# Supplementary material for: Organophotocatalytic selective deuterodehalogenation of aryl or alkyl chlorides
Source: Nat Commun. 2021 May 17;12:2894. doi: 10.1038/s41467-021-23255-0 (PMC8129137; doi:10.1038/s41467-021-23255-0)
Supplement: Supplementary file 1 — Supplementary Information [file 41467_2021_23255_MOESM1_ESM.pdf]

# Supplementary Information

## Organophotocatalytic selective deuterodehalogenation of aryl or alkyl chlorides

Yanjun Li, Ziqi Ye, Yu-Mei Lin, Yan Liu, Yumeng Zhang & Lei Gong*

# Table of Contents

**Supplementary Methods** ······························ S2

1. General Information ······························ S2
2. Synthesis of Substrates ······························ S3
3. Photocatalytic Deuterodehalogenation of Aryl and Alkyl Chlorides ······························ S16
   3.1 Optimization for Deuterodehalogenation of Aryl Chlorides ······························ S16
   3.2 Substrate Scope for Deuterodehalogenation of Aryl Chlorides ······························ S18
   3.3 Optimization for Deuterodehalogenation of Alkyl Chlorides ······························ S40
   3.4 Substrate Scope for Deuterodehalogenation of Alkyl Chlorides ······························ S42
   3.5 Site-Selective Deuterodechlorination of Polychlorides ······························ S58
   3.6 Gram-Scale Synthesis of Deuterated Zytiga *via* Selective Deuterodechlorination ·· S74
4. Mechanistic Investigations ······························ S77
   4.1 Cyclic Voltammetry Analysis ······························ S77
   4.2 UV/Vis-Absorption Spectra of the Photocatalysts ······························ S84
   4.3 Evaluation of Excited State Potentials of the Photocatalysts ······························ S85
   4.4 Estimating Lifetime of the Excited State of the Photocatalysts ······························ S87
   4.5 $CO_2$ Trapping Experiment ······························ S89
   4.6 Stern-Volmer Luminescence Quenching Studies ······························ S91
   4.7 Thiyl Radical Scrambling Experiment ······························ S92
   4.8 Calculation of Triplet State Energies of the Photocatalysts ······························ S93
   4.9 Calculation of BDE (C-Cl) of Dichlorides ······························ S96
5. Synthesis of Deuterated Drugs *via* Deuterodechlorination ······························ S98
6. Deuterodehalogenation of Halogen-Containing Bioactive Compounds and Drugs Derivatives ······························ S103
7. Application of the Methodology in Synthesis of Deuterated Drugs *via* H/D ExchangeS113
8. $^{1}H$, $^{2}H$ and $^{13}C$ NMR Spectra ······························ S121

**Supplementary References** ······························ S314

# Supplementary Methods

## 1. General Information

Visible-light-induced catalytic reactions were performed in 5 mL glass vials at the indicated temperature and under irradiation with a 50 W blue LEDs lamp ($\lambda_{max}$ = 400 nm; commercial supplier: Taiwan Epileds Co. Ltd., website: https://kiwilight.taobao.com). Photocatalysts **PC1**,[1] **PC3**,[2] **PC4**,[3] and substrates **3b**,[4] **3c**,[5] **3j**,[6] **5o**,[7] **5q**,[8] **5t**,[9] S11,[10] **S15**,[11] **S16**,[12] were synthesized according to the published procedures. DMSO (purity: 99.99%) were purchased from J&K. $D_2O$ were purchased from Aldrich (purity: 99.98%). All others reagents were purchased from commercial suppliers (TCI, Aldrich, Alfa, Macklin, Energy Chemical, Adamas-beta® and J&K) and used without further purification. Flash column chromatography was performed with silica gel (300–400 mesh, pH = 6.7–7.0). $^1$H NMR, $^2$H NMR and $^{13}$C NMR spectra were recorded on a Bruker AM (500 MHz) or Bruker AM (600 MHz) spectrometer at ambient temperature. NMR standards were used as follows: $CDCl_3$ = 7.26 ppm ($^1$H NMR), 77.0 ppm ($^{13}$C NMR); $(CD_3)_2CO$ = 2.05 ppm ($^1$H NMR), 29.7 and 206.4 ppm ($^{13}$C NMR); $(CD_3)_2SO$ = 2.49 ppm ($^1$H NMR), 39.6 ppm ($^{13}$C NMR); $D_2O$ = 4.80 ppm ($^1$H NMR). IR spectra were recorded on a Nicolet Avatar 330 FT-IR spectrophotometer. The luminescence decays were measured on an Edingburg FLS980 spectrometer. Fluorescece spectra were recorded on a Hitachi F-7000 Fluorescent Spectrophotometer. High-resolution mass spectra were performed on a Bruker micrOTOF-Q II mass spectrometer with a TOF analyzer. Cyclic voltammetry studies were carried out on a Shanghai Chen Hua CHI660D potentiostat offering compliance voltage up to ± 100 V (available at the counter electrode), scan range of ± 10 V and current range of ± 2 A.

## 2. Synthesis of Substrates

Substrates **3g–3i**, **5p**, **S13**, **S17**, **S20** and **S23** were prepared by a one-step synthesis.

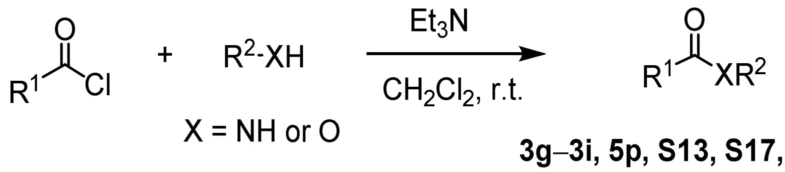


**3g–3i, 5p, S13, S17, S20 and S23**

***General procedure.*** To a solution of the amine, alcohol or phenol (1.0 eq), $Et_3N$ (2.0–3.0 eq) in $CH_2Cl_2$ (0.50 M) was added acyl chloride (1.10 eq) at room temperature. The reaction mixture was stirred for 1–15 h at room temperature, then quenched with $H_2O$ (20 mL) and extracted with $CH_2Cl_2$. The combined organic layers were dried over anhydrous $Na_2SO_4$ and concentrated under reduced pressure. The residue was subjected to silica gel chromatography (eluted with PE to PE:EtOAc = 3:1, PE = petroleum ether) to afford product.

**4-chlorobutyl [1,1'-biphenyl]-4-carboxylate (3g)**

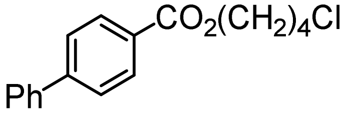


**3g**

According to the general procedure, 4-chlorobutan-1-ol (543 mg, 5.00 mmol) was converted to corresponding **3g** (1198 mg, 4.15 mmol, 83% yield) as a pale yellow oil.

$^{1}$H NMR (500 MHz, $CDCl_3$) δ 8.21 – 8.05 (m, 2H), 7.73 – 7.60 (m, 4H), 7.48 (dd, *J* = 10.3, 4.8 Hz, 2H), 7.41 (ddd, *J* = 7.4, 3.8, 1.2 Hz, 1H), 4.48 – 4.33 (m, 2H), 3.74 – 3.59 (m, 2H), 1.97 (dt, *J* = 6.1, 3.0 Hz, 4H).

$^{13}$C NMR (126 MHz, $CDCl_3$) δ 166.43, 145.72, 140.00, 130.11, 129.01, 128.97, 128.19, 127.29, 127.09, 64.15, 44.53, 29.34, 26.26.

IR (film): ν ($cm^{-1}$) 3059, 3032, 2959, 1937, 1709, 1609, 1488, 1449, 1406, 1291, 1179, 1112, 1008, 859, 745, 699, 653.

HRMS (ESI-TOF, m/z) calcd for $C_{17}H_{17}ClNaO_2$ $(M+Na)^+$: 311.0809, found: 311.0805.

**5-chloropentyl [1,1'-biphenyl]-4-carboxylate (3h)**

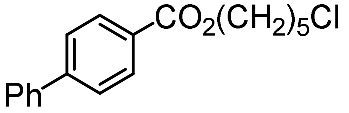


**3h**

According to the general procedure, 5-chloropentan-1-ol (613 mg, 5.00 mmol) was converted to corresponding **3h** (1453 mg, 4.80 mmol, 96% yield) as a pale yellow oil.

$^{1}$H NMR (500 MHz, $CDCl_3$) δ 8.13 (d, *J* = 8.1 Hz, 2H), 7.74 – 7.60 (m, 4H), 7.47 (t, *J* = 7.7 Hz, 2H), 7.43 – 7.36 (m, 1H), 4.37 (t, *J* = 6.5 Hz, 2H), 3.67 – 3.48 (m, 2H), 1.86 (qd, *J* = 14.2, 6.7 Hz, 4H), 1.64 (dt, *J* = 18.3, 7.7 Hz, 2H).

$^{13}$C NMR (126 MHz, $CDCl_3$) δ 166.49, 145.64, 140.02, 130.11, 129.15, 128.96, 128.17, 127.29, 127.07, 64.69, 44.79, 32.22, 28.11, 23.51.

IR (film): ν ($cm^{-1}$) 3059, 3032, 2955, 2868, 1936, 1807, 1718, 1609, 1488, 1450, 1406, 1388, 1267, 1179, 1112, 1021, 859, 752, 700, 650.

HRMS (ESI-TOF, m/z) calcd for $C_{18}H_{19}ClNaO_2$ $(M+Na)^+$: 325.0966, found: 325.0971.

**6-chlorohexyl [1,1'-biphenyl]-4-carboxylate (3i)**

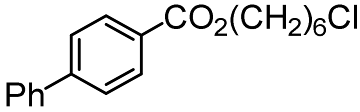


**3i**

According to the general procedure, 6-chlorohexan-1-ol (683.1 mg, 5.00 mmol) was converted to corresponding **3i** (1314 mg, 4.15 mmol, 83% yield) as a pale yellow oil.

$^{1}$H NMR (500 MHz, $CDCl_3$) δ 8.13 (d, *J* = 8.2 Hz, 2H), 7.72 – 7.60 (m, 4H), 7.47 (t, *J* = 7.7 Hz, 2H), 7.40 (t, *J* = 7.4 Hz, 1H), 4.36 (t, *J* = 6.6 Hz, 2H), 3.56 (t, *J* = 6.7 Hz, 2H), 1.88 – 1.75 (m, 4H), 1.59 – 1.44 (m, 4H).

$^{13}$C NMR (126 MHz, $CDCl_3$) δ 166.53, 145.61, 140.03, 130.10, 129.21, 128.96, 128.16, 127.29, 127.06, 64.89, 44.97, 32.52, 28.67, 26.62, 25.47.

IR (film): ν ($cm^{-1}$) 3059, 3032, 2940, 2861, 1937, 1807, 1714, 1609, 1488, 1450, 1406, 1270, 1179, 1120, 1021, 1008, 859, 753, 700.

HRMS (ESI-TOF, m/z) calcd for $C_{19}H_{21}ClNaO_2$ $(M+Na)^+$: 339.1122, found: 339.1119.

**6-chlorohexyl 6-chloronicotinate (5p)**

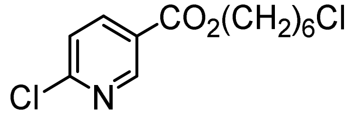


**5p**

According to the general procedure, 6-chlorohexan-1-ol (683 mg, 5.00 mmol) was converted to corresponding **5p** (1257 mg, 4.55 mmol, 91% yield) as a colorless oil.

$^{1}H$ NMR (500 MHz, $CDCl_3$) δ 9.02 – 8.89 (m, 1H), 8.20 (dd, *J* = 8.3, 2.4 Hz, 1H), 7.38 (dd, *J* = 8.3, 0.6 Hz, 1H), 4.32 (t, *J* = 6.7 Hz, 2H), 3.51 (t, *J* = 6.6 Hz, 2H), 1.81 – 1.71 (m, 4H), 1.54 – 1.39 (m, 4H).

$^{13}C$ NMR (126 MHz, $CDCl_3$) δ 164.39, 155.58, 151.11, 139.54, 125.24, 124.16, 65.55, 44.86, 32.38, 28.46, 26.47, 25.29.

IR (film): ν ($cm^{-1}$) 3093, 3066, 2941, 2861, 1733, 1587, 1566, 1456, 1389, 1366, 1289, 1124, 1021, 848, 768, 719.

HRMS (ESI-TOF, m/z) calcd for $C_{12}H_{15}Cl_2NNaO_2$ $(M+Na)^+$: 298.0372, found: 298.0361.

**2-butoxyethyl 2-chloronicotinate (S13)**

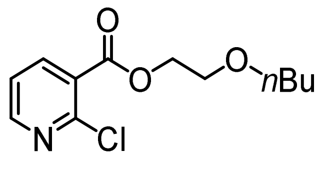


**S13**

According to the general procedure, 2-butoxyethan-1-ol (591 mg, 5.00 mmol) was converted to corresponding **S13** (1198 mg, 4.65 mmol, 93% yield) as a colorless oil.

$^1$H NMR (500 MHz, $CDCl_3$) δ 8.44 (dd, *J* = 4.8, 2.0 Hz, 1H), 8.11 (dd, *J* = 7.7, 2.0 Hz, 1H), 7.29 – 7.24 (m, 1H), 4.48 – 4.37 (m, 2H), 3.77 – 3.63 (m, 2H), 3.43 (t, *J* = 6.6 Hz, 2H), 1.50 (dt, *J* = 14.5, 6.7 Hz, 2H), 1.35 – 1.24 (m, 2H), 0.83 (t, *J* = 7.4 Hz, 3H).

$^{13}$C NMR (126 MHz, $CDCl_3$) δ 164.35, 151.82, 149.99, 140.28, 126.87, 122.05, 71.10, 68.21, 65.04, 31.61, 19.17, 13.80.

IR (film): ν ($cm^{-1}$) 2959, 1734, 1578, 1560, 1407, 1300, 1130, 1065, 821, 764.

HRMS (ESI-TOF, m/z) calcd for $C_{12}H_{16}ClNNaO_3$ $(M+Na)^+$: 280.0711, found: 280.0708.

**ethyl 4-(6-chloronicotinamido)butanoate (S17)**

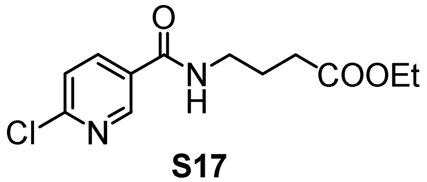

**S17**

According to the general procedure, ethyl 4-aminobutanoate hydrochloride (838 mg, 5.00 mmol) was converted to corresponding **S17** (1150 mg, 4.25 mmol, 85% yield) as a white solid.

$^1$H NMR (500 MHz, $CDCl_3$) δ 8.78 (d, *J* = 2.4 Hz, 1H), 8.10 (dd, *J* = 8.3, 2.5 Hz, 1H), 7.40 (d, *J* = 8.3 Hz, 1H), 7.11 (s, 1H), 4.13 (q, *J* = 7.1 Hz, 2H), 3.51 (dd, *J* = 12.1, 6.2 Hz, 2H), 2.47 (t, *J* = 6.7 Hz, 2H), 1.96 (p, *J* = 6.6 Hz, 2H), 1.24 (t, *J* = 7.1 Hz, 3H).

$^{13}$C NMR (151 MHz, $CDCl_3$) δ 173.82, 164.73, 153.84, 148.32, 137.99, 129.13, 124.15, 60.70, 39.85, 31.92, 24.12, 14.09.

IR (film): ν ($cm^{-1}$) 3294, 3051, 2983, 2945, 2914, 1737, 1636, 1547, 1462, 1328, 1107, 1018, 941, 750, 635.

HRMS (ESI-TOF, m/z) calcd for $C_{12}H_{15}ClN_2NaO_3$ $(M+Na)^+$: 293.0663, found: 293.0655.

**(*R*)-2,5,7,8-tetramethyl-2-((4*R*,8*R*)-4,8,12-trimethyltridecyl)chroman-6-yl-6-chloronicotinate (S20)**

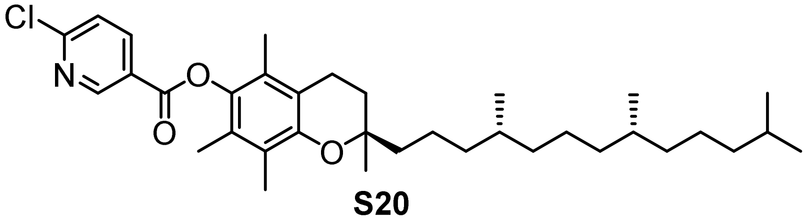

**S20**

According to the general procedure, Vitamin E (2154 mg, 5.00 mmol) was converted to corresponding **S20** (1150 mg, 4.25 mmol, 85% yield) as a grey oil.

$^1$H NMR (600 MHz, $CDCl_3$) δ 9.25 (d, *J* = 2.3 Hz, 1H), 8.43 (dd, *J* = 8.3, 2.3 Hz, 1H), 7.47 (d, *J* = 8.3 Hz, 1H), 2.65 (t, *J* = 6.7 Hz, 2H), 2.17 (s, 3H), 2.08 (s, 3H), 2.04 (s, 3H), 1.87 (dt, *J* = 13.7, 7.0 Hz, 1H), 1.61 – 1.40 (m, 8H), 1.40 – 1.28 (m, 11H), 1.20 – 1.08 (m, 6H), 0.94 – 0.89 (m, 12H).

$^{13}$C NMR (151 MHz, $CDCl_3$) δ 163.06, 156.23, 151.59, 149.82, 140.29, 140.05, 126.65, 124.93, 124.59, 124.46, 123.37, 117.63, 75.19, 39.45, 37.51, 37.36, 32.86, 32.74, 31.22, 28.03, 24.88, 24.52, 24.23, 23.69, 22.81, 22.71, 21.08, 20.69, 19.84, 19.75, 13.11, 12.27, 11.93.

IR (film): ν (cm$^{-1}$) 2928, 1743, 1587, 1456, 1379, 1241, 1101, 1015, 806, 764.

HRMS (ESI-TOF, m/z) calcd for $C_{35}H_{52}ClNNaO_3$ $(M+Na)^+$: 592.3528, found: 592.3516.

**(3a*S*,5*S*,6*R*,6a*S*)-5-((*S*)-2,2-dimethyl-1,3-dioxolan-4-yl)-2,2-dimethyltetrahydrofuro[2,3-d][1,3]dioxol-6-yl 4-(chloromethyl)benzoate (S23)**

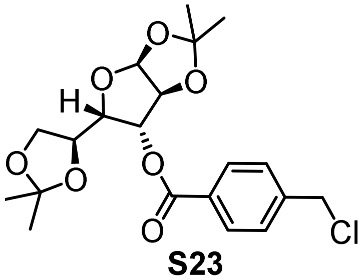

**S23**

According to the general procedure, Diacetone-d-Glucose (1301 mg, 5.00 mmol) was converted to corresponding **S23** (1651 mg, 4.0 mmol, 80% yield) as a white solid.

$^{1}H$ NMR (500 MHz, $CDCl_3$) δ 7.98 (d, *J* = 8.0 Hz, 2H), 7.44 (d, *J* = 8.0 Hz, 2H), 5.93 (d, *J* = 3.5 Hz, 1H), 5.47 (d, *J* = 2.2 Hz, 1H), 4.72 – 4.51 (m, 3H), 4.40 – 4.23 (m, 2H), 4.16 – 4.01 (m, 2H), 1.52 (s, 3H), 1.38 (s, 3H), 1.29 (s, 3H), 1.23 (s, 3H).

$^{13}C$ NMR (151 MHz, $CDCl_3$) δ 164.65, 142.90, 130.12, 129.45, 128.65, 112.35, 109.40, 105.14, 83.37, 79.94, 72.56, 67.26, 45.21, 26.84, 26.73, 26.21, 25.21.

IR (film): ν ($cm^{-1}$) 2989, 2938, 2898, 1733, 1614, 1415, 1374, 1269, 1165, 1103, 847, 713, 513.

HRMS (ESI-TOF, m/z) calcd for $C_{20}H_{25}ClNaO_7$ $(M+Na)^+$: 435.1181, found: 435.1177.

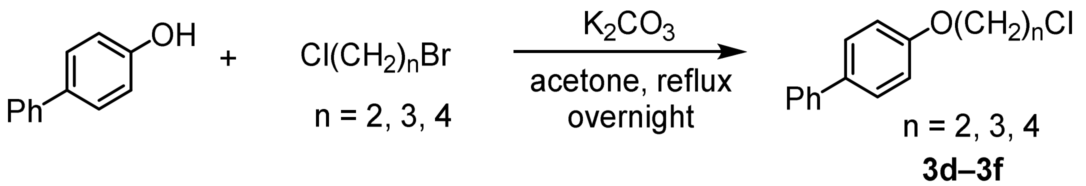


***General procedure.*** To a solution of 4-phenylphenol (5.0 mmol, 1.0 eq), $K_2CO_3$ (6.0 mmol, 1.2 eq) in acetone (0.50 M) was added 1-bromo-*n*-chloro alkane (1.0 eq). The reaction mixture was stirred for overnight at 70 °C, then concentrated and subjected to flash chromatography on silica gel (eluted with PE to PE:EtOAc = 20:1) to afford the desired product.

**4-(2-chloroethoxy)-1,1'-biphenyl (3d)**

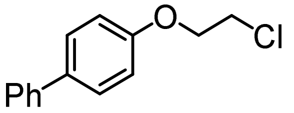


**3d**

According to the general procedure, 1-Bromo-2-Chloroethane (717 mg, 5.00 mmol) was converted to corresponding **3d** (384 mg, 4.55 mmol, 33% yield) as a white solid.

$^{1}H$ NMR (600 MHz, $CDCl_3$) δ 7.57 (dd, *J* = 12.0, 8.2 Hz, 4H), 7.45 (t, *J* = 7.6 Hz, 2H), 7.35 (t, *J* = 7.2 Hz, 1H), 7.02 (d, *J* = 8.5 Hz, 2H), 4.28 (t, *J* = 5.8 Hz, 2H), 3.85 (t, *J* = 5.8 Hz, 2H).

$^{13}C$ NMR (151 MHz, $CDCl_3$) δ 157.79, 140.67, 134.55, 128.82, 128.31, 126.87, 126.81, 115.07, 68.15, 41.97.

IR (film): ν (cm$^{-1}$) 2968, 1609, 1519, 1486, 1451, 1408, 1286, 1265, 1189, 1037, 830, 757, 742.

HRMS (ESI-TOF, m/z) calcd for $C_{14}H_{14}ClO$ $(M+H)^+$: 233.0728, found: 233.0733.

**4-(3-chloropropoxy)-1,1'-biphenyl (3e)**

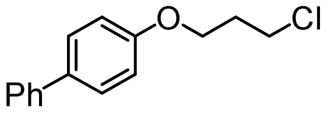


**3e**

According to the general procedure, 1-Bromo-3-Chloropropane (787 mg, 5.00 mmol) was converted to corresponding **3e** (814 mg, 3.30 mmol, 66% yield) as a white solid.

$^1$H NMR (500 MHz, $CDCl_3$) δ 7.59 – 7.50 (m, 4H), 7.42 (t, *J* = 7.7 Hz, 2H), 7.31 (t, *J* = 7.4 Hz, 1H), 7.02 – 6.96 (m, 2H), 4.17 (t, *J* = 5.8 Hz, 2H), 3.78 (t, *J* = 6.3 Hz, 2H), 2.27 (p, *J* = 6.1 Hz, 2H).

$^{13}$C NMR (126 MHz, $CDCl_3$) δ 158.29, 140.79, 134.06, 128.74, 128.21, 126.76, 126.71, 114.82, 64.39, 41.54, 32.32.

IR (film): ν (cm$^{-1}$) 2962, 1607, 1523, 1491, 1451, 1421, 1270, 1251, 1200, 1118, 1038, 948, 832, 762, 691, 655.

HRMS (ESI-TOF, m/z) calcd for $C_{15}H_{15}ClNaO$ $(M+Na)^+$: 269.0704, found: 269.0712.

**4-(4-chlorobutoxy)-1,1'-biphenyl (3f)**

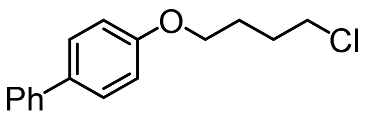


**3f**

According to the general procedure, 1-Bromo-4-Chlorobutane (857 mg, 5.00 mmol) was converted to corresponding **3f** (1069 mg, 4.10 mmol, 82% yield) as a white solid

$^1$H NMR (500 MHz, $CDCl_3$) δ 7.61 – 7.51 (m, 4H), 7.44 (dd, *J* = 10.6, 4.8 Hz, 2H), 7.33 (t, *J* = 7.4 Hz, 1H), 7.02 – 6.95 (m, 2H), 4.06 (t, *J* = 5.8 Hz, 2H), 3.65 (t, *J* = 6.2 Hz, 2H), 2.10 – 1.87 (m, 4H).

$^{13}C$ NMR (126 MHz, $CDCl_3$) δ 158.61, 140.88, 133.86, 128.90, 128.26, 126.82, 114.92, 67.17, 44.91, 29.49, 26.84.

IR (film): ν ($cm^{-1}$) 3036, 2948, 2876, 1985, 1609, 1524, 1490, 1397, 1287, 1272, 1203, 1048, 1023, 838, 733.

HRMS (ESI-TOF, m/z) calcd for $C_{16}H_{17}ClNaO$ $(M+Na)^+$: 283.0860, found: 283.0860.

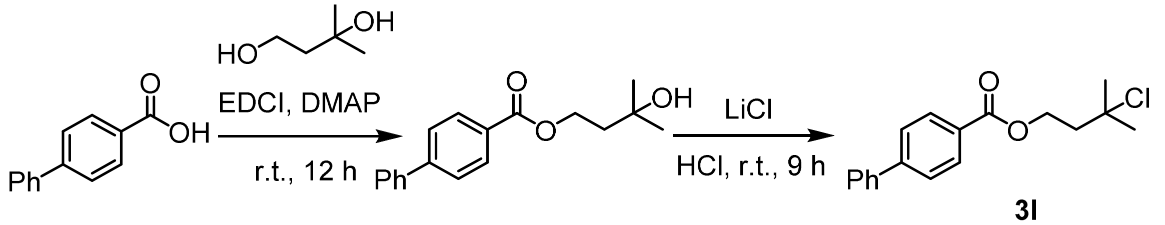

**3l**

To a solution of 4-biphenylcarboxylic acid (10.0 mmol, 1982 mg), EDC·HCl (1-(3-dimethylaminopropyl)-3-ethylcarbodiimide hydrochloride, 2300 mg, 12.0 mmol), DMAP (4-dimethylaminopyridine, 244 mg, 2.0 mmol) in $CH_2Cl_2$ (0.20 M, 50 mL) was added 3-methyl-1,3-butanediol (1600 mg, 15.0 mmol). The reaction mixture was stirred for 12 h at room temperature, then quenched with $H_2O$ (20 mL) and extracted with $CH_2Cl_2$. The combined organic layer was dried over anhydrous $Na_2SO_4$ and concentrated under reduced pressure. The resultant crude product alcohol were used in the next step without further purification.

A Schlenk tube was charged with LiCl (1.05 g, 25.0 mmol) in concentrated HCl (20 mL), and the crude alcohol (dissolved in a minimal amount of $CH_2Cl_2$). The reaction mixture was stirred for 9 h at room temperature, then extracted with $CH_2Cl_2$. The combined organic layer was dried over anhydrous $Na_2SO_4$ and concentrated under reduced pressure. The residue was subjected to silica gel chromatography (eluted with PE to PE:EtOAc = 40:1) to afford product **3l** (1302 mg, 4.30 mmol, 43% yield) as a white solid.

**3-chloro-3-methylbutyl [1,1'-biphenyl]-4-carboxylate (3l)**

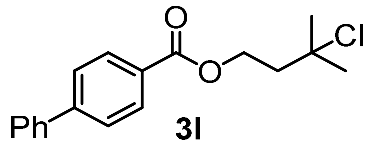


$^{1}$H NMR (500 MHz, $CDCl_3$) δ 8.20 – 8.06 (m, 2H), 7.72 – 7.56 (m, 4H), 7.48 (t, $J$ = 7.5 Hz, 2H), 7.40 (t, $J$ = 6.9 Hz, 1H), 4.60 (t, $J$ = 6.7 Hz, 2H), 2.29 (t, $J$ = 6.7 Hz, 2H), 1.71 (s, 6H).

$^{13}$C NMR (126 MHz, $CDCl_3$) δ 166.37, 145.76, 140.00, 130.10, 128.95, 128.18, 127.29, 127.10, 68.46, 62.09, 44.16, 32.96.

IR (film): ν ($cm^{-1}$) 3059, 3032, 2974, 2928, 1717, 1609, 1405, 1278, 1179, 1115, 1008, 859, 749, 699, 575.

HRMS (ESI-TOF, m/z) calcd for $C_{18}H_{19}ClNaO_2$ $(M+Na)^+$: 325.0966, found: 325.0966.

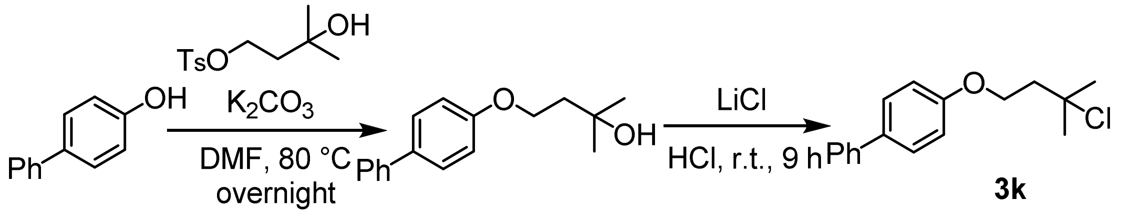


To a solution of 4-phenylphenol (1982 mg, 10.0 mmol), $K_2CO_3$ (1658 mg, 12.0 mmol) in DMF (20 mL) was added 3-hydroxy-3-methylbutyl 4-methylbenzenesulfonate (3100 mg, 12.0 mmol). The reaction mixture was stirred for overnight at 80 °C, then quenched with $H_2O$ (20 mL) and extracted with $CH_2Cl_2$. The combined organic layer was dried over anhydrous $Na_2SO_4$ and concentrated under reduced pressure. The resultant crude product alcohol were used in the next step without further purification.

A Schlenk tube was charged with LiCl (1050 mg, 25.0 mmol) in concentrated HCl (20 mL), and the crude alcohol (dissolved in a minimal amount of $CH_2Cl_2$). The reaction mixture was stirred for 9 h at room temperature, then extracted with $CH_2Cl_2$. The combined organic layer was dried over anhydrous $Na_2SO_4$ and concentrated under reduced pressure. The residue was subjected to silica gel chromatography (eluted with PE to PE:EtOAc = 40:1) to afford product **3k** (495 mg, 1.80 mmol, 18% yield) as a white solid.

**4-(3-chloro-3-methylbutoxy)-1,1'-biphenyl (3k)**

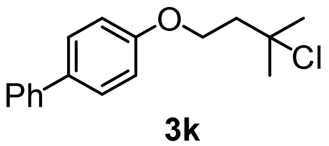


**3k**

$^{1}$H NMR (600 MHz, $CDCl_3$) δ 7.56 (dd, *J* = 15.2, 8.1 Hz, 4H), 7.44 (t, *J* = 7.6 Hz, 2H), 7.33 (t, *J* = 7.3 Hz, 1H), 7.01 (d, *J* = 8.5 Hz, 2H), 4.29 (t, *J* = 6.5 Hz, 2H), 2.31 (t, *J* = 6.5 Hz, 2H), 1.71 (s, 6H).

$^{13}$C NMR (151 MHz, $CDCl_3$) δ 158.23, 140.83, 133.93, 128.77, 128.23, 126.77, 126.72, 114.82, 69.22, 65.02, 44.57, 33.09.

IR (film): ν ($cm^{-1}$) 3032, 2926, 1609, 1520, 1490, 1451, 1290, 1248, 1176, 1132, 1043, 833, 763, 693, 552.

HRMS (ESI-TOF, m/z) calcd for $C_{17}H_{19}ClNaO$ $(M+Na)^+$: 297.1017, found: 297.1013.

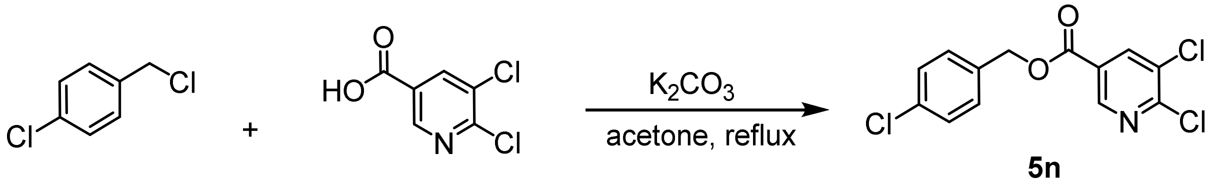


To a solution of 5,6-dichloronicotinic acid (5.0 mmol, 1.0 eq), $K_2CO_3$ (6.0 mmol, 1.2 eq) in acetone (0.50 M) was added 1-chloro-4-(chloromethyl)benzene (1.0 eq). The reaction mixture was stirred for overnight at 70 °C, then concentrated and subjected to flash chromatography on silica gel (eluted with PE to PE:EtOAc = 10:1) to afford the desired product **5n** (1203 mg, 3.8 mmol, 76% yield) as a white solid.

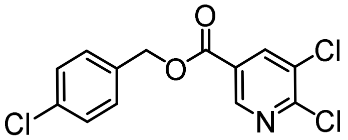


**5n**

$^{1}$H NMR (500 MHz, $CDCl_3$) δ 8.88 (d, *J* = 2.0 Hz, 1H), 8.33 (d, *J* = 2.0 Hz, 1H), 7.37 (s, 4H), 5.35 (s, 2H).

$^{13}C$ NMR (126 MHz, $CDCl_3$) δ 163.23, 153.54, 148.28, 139.36, 134.80, 133.45, 130.85, 129.96, 129.01, 125.98, 66.91.

IR (film): ν ($cm^{-1}$) 3066, 2962, 2878, 1897, 1727, 1580, 1492, 1374, 1290, 1218, 1123, 1043, 953, 804, 765, 721.

HRMS (ESI-TOF, m/z) calcd for $C_{13}H_9Cl_3NO_2$ $(M+H)^+$: 315.9693, found: 315.9693.

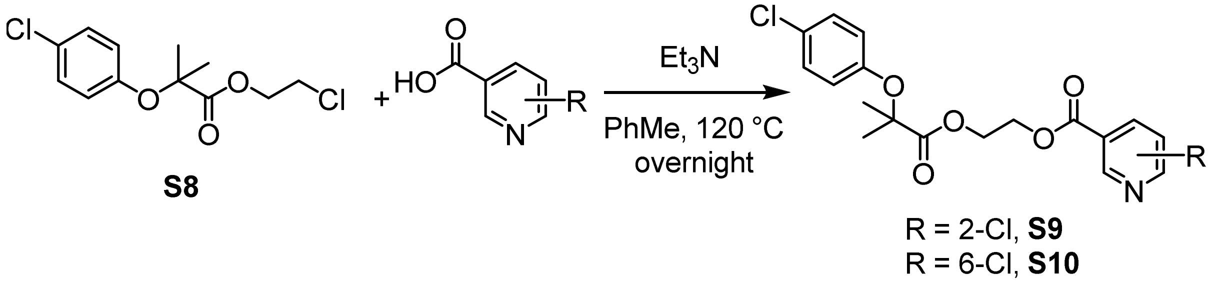


***General procedure.*** To a solution of **S8** (1386 mg, 5.00 mmol) and $Et_3N$ (1.40 mL, 10.0 mmol) in PhMe (10 mL) was added chloronicotinic acid (945 mg, 6.0 mmol). The mixture was stirred for overnight at 120 °C, and then concentrated under reduced pressure. The residue was subjected to silica gel chromatography (eluted with PE:EtOAc = 3:1) to afford products **S9** or **S10**.

**2-((2-(4-chlorophenoxy)-2-methylpropanoyl)oxy)ethyl 2-chloronicotinate (S9)**

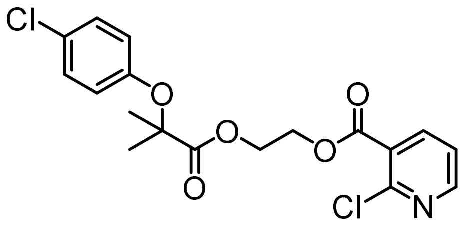

**S9**

According to the general procedure, 2-chloronicotinic acid was converted to corresponding **S9** (1712 mg, 4.3 mmol, 86% yield) as a grey oil.

$^1$H NMR (500 MHz, CDCl$_3$) δ 8.49 (dd, *J* = 4.7, 1.9 Hz, 1H), 7.94 (dd, *J* = 7.7, 2.0 Hz, 1H), 7.25 (dd, *J* = 7.7, 4.8 Hz, 1H), 7.13 – 7.04 (m, 2H), 6.83 – 6.68 (m, 2H), 4.58 – 4.41 (m, 4H), 1.56 (s, 6H).

$^{13}$C NMR (126 MHz, CDCl$_3$) δ 173.85, 163.95, 153.89, 152.16, 150.12, 140.33, 129.11, 127.21, 126.10, 122.14, 120.31, 79.33, 63.24, 62.92, 25.27.

IR (film): ν (cm$^{-1}$) 3640, 3463, 3075, 2993, 2957, 1729, 1579, 1485, 1408, 1339, 1062, 1011, 972, 829, 763, 670.

HRMS (ESI-TOF, m/z) calcd for $C_{18}H_{18}Cl_2NO_5$ (M+H)$^+$: 398.0557, found: 398.0553.

**2-((2-(4-chlorophenoxy)-2-methylpropanoyl)oxy)ethyl 6-chloronicotinate (S10)**

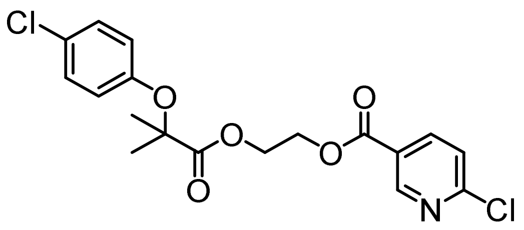


**S10**

According to the general procedure, 6-chloronicotinic acid was converted to corresponding **S10** (1692 mg, 4.25 mmol, 85% yield) as a grey oil.

$^1$H NMR (500 MHz, CDCl$_3$) δ 8.83 (d, *J* = 1.9 Hz, 1H), 8.00 (dd, *J* = 8.3, 2.3 Hz, 1H), 7.34 (d, *J* = 8.3 Hz, 1H), 7.04 (d, *J* = 8.9 Hz, 2H), 6.69 (d, *J* = 8.9 Hz, 2H), 4.61 – 4.36 (m, 4H), 1.54 (s, 6H).

$^{13}$C NMR (126 MHz, CDCl$_3$) δ 173.83, 163.94, 155.91, 153.91, 151.09, 139.44, 129.09, 127.10, 124.41, 124.23, 120.00, 79.25, 62.93, 62.86, 25.24.

IR (film): ν (cm$^{-1}$) 3068, 2994, 2958, 1888, 1733, 1588, 1489, 1385, 1368, 1270, 1241, 1176, 1131, 1020, 829, 790, 767.

HRMS (ESI-TOF, m/z) calcd for $C_{18}H_{18}Cl_2NO_5$ (M+H)$^+$: 398.0557, found: 398.0556.

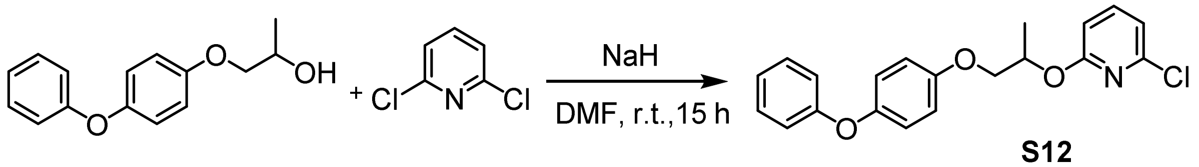

S12

To a solution of 1-(4-phenoxyphenoxy)propan-2-ol (1982 mg, 5.00 mmol) in anhydrous DMF (20 mL) was added NaH (60% dispersion in mineral oil, 240 mg, 6.0 mmol) at room temperature under argon atmosphere. The mixture was stirred for 30 min. 2,6-dichloropyridine (740 mg, 5.0 mmol) was then added to the mixture. The reaction was stirred at room temperature for 15 h, then quenched with $H_2O$ (50 mL) and extracted with EtOAc. The combined organic layer was dried over anhydrous $Na_2SO_4$ and concentrated under reduced pressure. The residue was subjected to silica gel chromatography (eluted with PE:EtOAc = 40:1) to afford product **S12** (1300 mg, 3.65 mmol, 73% yield) as a colorless oil.

**2-chloro-6-((1-(4-phenoxyphenoxy)propan-2-yl)oxy)pyridine (S12)**

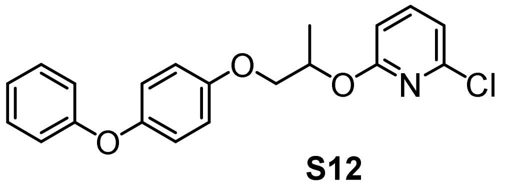

**S12**

$^1$H NMR (500 MHz, $CDCl_3$) δ 7.52 (t, *J* = 7.8 Hz, 1H), 7.33 (dd, *J* = 8.1, 7.7 Hz, 2H), 7.10 – 6.96 (m, 7H), 6.92 (d, *J* = 7.5 Hz, 1H), 6.70 (d, *J* = 8.2 Hz, 1H), 5.67 – 5.55 (m, 1H), 4.23 (dd, *J* = 10.1, 5.3 Hz, 1H), 4.10 (dd, *J* = 10.1, 4.8 Hz, 1H), 1.53 (d, *J* = 6.4 Hz, 3H).

$^{13}$C NMR (126 MHz, $CDCl_3$) δ 162.87, 158.56, 155.15, 150.46, 148.30, 140.86, 129.70, 122.54, 120.87, 117.71, 116.47, 115.92, 109.77, 70.82, 70.34, 16.91.

IR (film): ν (cm$^{-1}$) 3042, 2982, 2936, 2552, 2411, 1951, 1858, 1588, 1437, 1223, 962, 786, 692.

HRMS (ESI-TOF, m/z) calcd for $C_{20}H_{18}ClNNaO_3$ $(M+Na)^+$: 378.0867, found: 378.0860.

# 3. Photocatalytic Deuterodehalogenation of Aryl and Alkyl Chlorides

## 3.1 Optimization for Deuterodehalogenation of Aryl Chlorides

A dried 5 mL glass vial was charged with 4-chlorobiphenyl **1a** (0.10 mmol), photocatalyst **PC** (0.010 mmol), thiol or disulfide (0.030 mmol), additive (0.20 mmol), and the indicated solvent under air and then performed in a sealed vessel. The glass vial was positioned approximately 3 cm away from a 50 W blue LEDs lamp ($\lambda_{max}$ = 400 nm). The reactions were stirred at room temperature (~ 30 °C under irradiation) for the indicated time. The conversion was determined by $^{1}$H NMR analysis of the crude mixture. The reaction mixture was purified by silica gel chromatography (eluted with PE) to provide pure product **2a** for determining deuterium incorporation.

**Supplementary Table 1** Initial experiments for photocatalytic deuterodehalogenation of aryl chlorides[a].

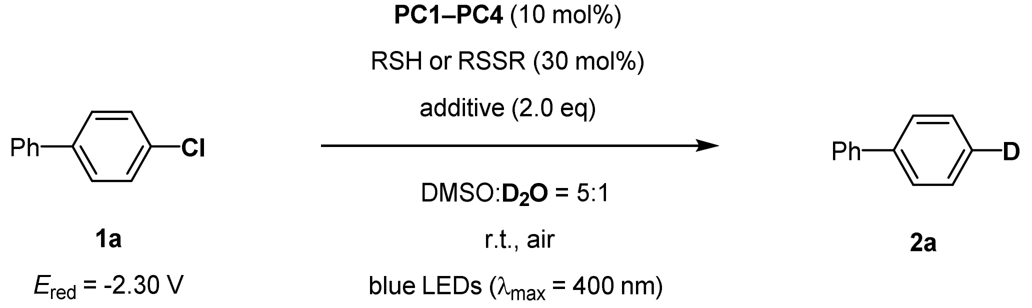


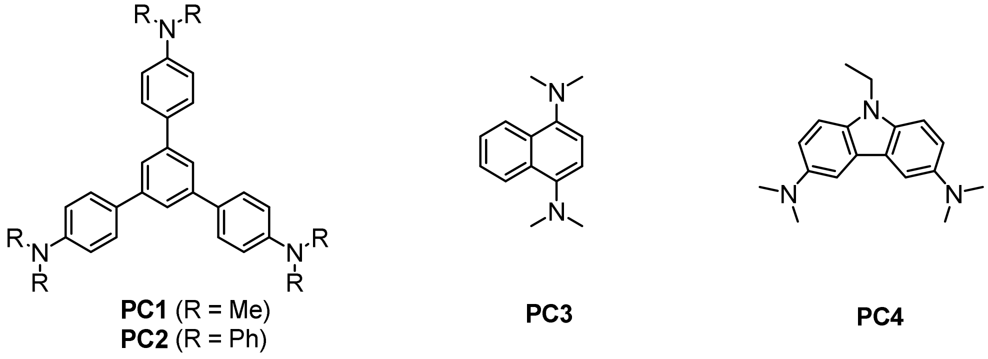


| entry | PC | RSH or RSSR | additive | t (h) | conv. (%)[b] | D-inc. (%)[c] |
|---|---|---|---|---|---|---|
| 1 | **PC1** | *n*PrSH | none | 15 | < 5 | n.d. |
| 2 | **PC1** | *n*PrSH | $Na_2CO_3$ | 15 | 77 | 20 |

| | | | | | | |
|---|---|---|---|---|---|---|
| 3 | **PC1** | *n*PrSH | NaOAc | 15 | 60 | 10 |
| 4 | **PC1** | *n*PrSH | $Na_3PO_4$ | 15 | 57 | 16 |
| 5 | **PC1** | *n*PrSH | Hantzsch ester | 15 | 0 | n.a. |
| 6 | **PC1** | *n*PrSH | $HCO_2Na$ | 15 | quant. | 85 |
| 7 | **PC1** | *n*PrSH | $HCO_2K$ | 15 | quant. | 84 |
| 8 | **PC1** | *n*PrSH | $HCO_2NH_4$ | 15 | quant. | 73 |
| 9 | **PC1** | ethyl 2-mercaptoacetate | $HCO_2Na$ | 9 | quant. | 53 |
| 10 | **PC1** | 4-MeOPhSH | $HCO_2Na$ | 9 | 90 | 24 |
| 11 | **PC1** | $(CyS)_2$ | $HCO_2Na$ | 9 | 69 | 6 |
| 12 | **PC1** | $(n\text{PrS})_2$ | $HCO_2Na$ | 9 | quant. | 91 |
| 13 | **PC1** | $(MeS)_2$ | $HCO_2Na$ | 9 | quant. | 92 |
| 14 | **PC1** | $(EtS)_2$ | $HCO_2Na$ | 9 | 85 | 92 |
| 15 | **PC1** | $(n\text{BuS})_2$ | $HCO_2Na$ | 9 | 66 | 9 |
| 16 | **PC2** | $(n\text{PrS})_2$ | $HCO_2Na$ | 9 | 26 | 92 |
| 17 | **PC3** | $(n\text{PrS})_2$ | $HCO_2Na$ | 9 | 36 | 84 |
| 18 | **PC4** | $(n\text{PrS})_2$ | $HCO_2Na$ | 9 | 51 | 81 |
| 19 | none | $(MeS)_2$ | $HCO_2Na$ | 25 | < 5 | n.d. |
| 20[d] | **PC1** | $(MeS)_2$ | $HCO_2Na$ | 25 | 0 | n.d. |
| 21 | **PC1** | none | $HCO_2Na$ | 25 | 90 | 10 |

[a]Reaction conditions: **1a** (0.10 mmol), $D_2O$ (0.10 mL), **PC1−PC4** (0.010 mmol), thiol or disulfide (0.030 mmol), additive (0.20 mmol), DMSO (0.50 mL), a 50 W blue LEDs lamp ($\lambda_{max}$ = 400 nm), room temperature (~ 30 °C under irradiation), in air.

[b]Degree of conversion determined by $^1$H-NMR.

[c]Deuterium incorporation (D-inc.) determined by $^1$H-NMR. n.d. = not determined. n.a. = not applicable.

[d]In the dark.

### 3.2 Substrate Scope for Deuterodehalogenation of Aryl Chlorides

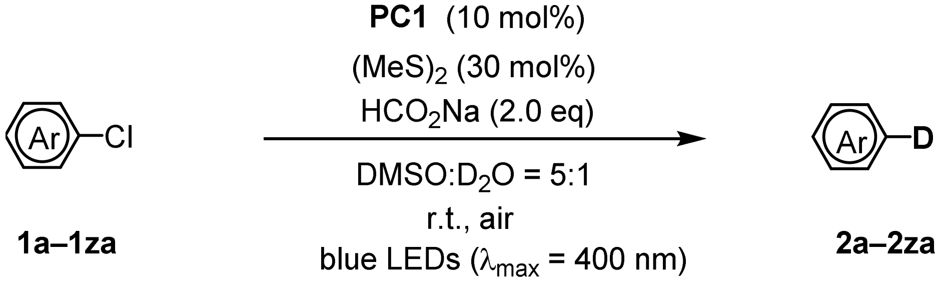


### 3.2.1 General Procedure

A dried 5 mL glass vial was charged with aryl chlorides **1a–1za** (0.20 mmol), photocatalyst **PC1** (8.7 mg, 0.020 mmol), $(MeS)_2$ (6 μL, 0.060 mmol), $HCO_2Na$ (27.2 mg, 0.40 mmol), $D_2O$ (200 μL) and DMSO (1.0 mL) under air and then performed in a sealed vessel. The glass vial was positioned approximately 3 cm away from a 50 W blue LEDs lamp ($\lambda_{max}$ = 400 nm). After being stirred at room temperature (~ 30 °C under irradiation) for the indicated time, the reaction mixture was purified by flash chromatography on silica gel to afford product **2a–2za**.

### 3.2.2 Experimental Details and Characterization Data

**1,1'-biphenyl-4-D (2a)**

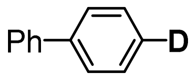


**2a**

A dried 5 mL glass vial was charged with aryl chloride **1a** (37.7 mg, 0.20 mmol), photocatalyst **PC1** (8.7 mg, 0.020 mmol), $(MeS)_2$ (6 μL, 0.060 mmol), $HCO_2Na$ (27.2 mg, 0.40 mmol), $D_2O$ (200 μL) and DMSO (1.0 mL) under air and then performed in a sealed vessel. The glass vial was positioned approximately 3 cm away from a 50 W blue LEDs lamp ($\lambda_{max}$ = 400 nm). After being stirred at room temperature (~ 30 °C under irradiation) for 12 h, the reaction mixture was purified by flash chromatography on silica gel (eluted with PE) to afford product **2a** (22.3 mg, 0.162 mmol, 81% yield, 92% D) as a white solid.

$^{1}H$ NMR (600 MHz, $CDCl_3$) δ 7.63 (d, $J$ = 7.7 Hz, 4H), 7.52 – 7.43 (m, 4H), 7.38 (t, $J$ = 7.4 Hz, 1.08H).

$^{13}C$ NMR (151 MHz, $CDCl_3$) δ 141.29, 128.79, 128.68, 127.29, 127.21.

$^{2}H$ NMR (92 MHz, $CH_2Cl_2$) δ 7.38 (s, 1D).

IR (film): ν ($cm^{-1}$) 3030, 2961, 2855, 1948, 1596, 1561, 1481, 1402, 1265, 1078, 1078, 1010, 857, 737, 608.

HRMS (ESI-TOF, m/z) calcd for $C_{12}H_9DNa$ $(M+Na)^+$: 178.0737, found: 178.0763.

**naphthalene-1-D (2b)**

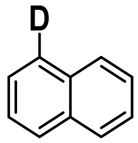

**2b**

A dried 5 mL glass vial was charged with aryl chloride **1b** (32.5 mg, 0.20 mmol), photocatalyst **PC1** (8.7 mg, 0.020 mmol), $(MeS)_2$ (6 μL, 0.060 mmol), $HCO_2Na$ (27.2 mg, 0.40 mmol), $D_2O$ (200 μL) and DMSO (1.0 mL) under air and then performed in a sealed vessel. The glass vial was positioned approximately 3 cm away from a 50 W blue LEDs lamp ($\lambda_{max}$ = 400 nm). After being stirred at room temperature (~ 30 °C under irradiation) for 12 h, the reaction mixture was purified by flash chromatography on silica gel (eluted with PE) to afford product **2b** (18.6 mg, 0.144 mmol, 72% yield, 97% D) as a white solid.

$^{1}H$ NMR (600 MHz, $CDCl_3$) δ 7.87 (dd, $J$ = 6.0, 3.4 Hz, 3.03H), 7.51 (dd, $J$ = 6.4, 3.0 Hz, 4H).

$^{13}C$ NMR (151 MHz, $CDCl_3$) δ 133.48, 133.41, 127.92, 127.87, 127.60 (t, $J$ = 24.2 Hz), 125.86, 125.76, 125.74.

$^{2}H$ NMR (92 MHz, $CH_2Cl_2$) δ 8.18 (s, 1D).

IR (film): ν ($cm^{-1}$) 3055, 1590,1264, 953, 868, 806.

HRMS (ESI-TOF, m/z) calcd for $C_{10}H_7DNa$ $(M+Na)^+$: 152.0581, found: 152.0582.

**phenanthrene-9-D (2c)**

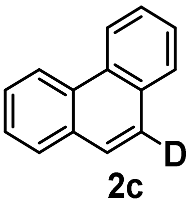

**2c**

A dried 5 mL glass vial was charged with aryl chloride **1c** (42.5 mg, 0.20 mmol), photocatalyst **PC1** (8.7 mg, 0.020 mmol), $(MeS)_2$ (6 μL, 0.060 mmol), $HCO_2Na$ (27.2 mg, 0.40 mmol), $D_2O$ (200 μL) and DMSO (1.0 mL) under air and then performed in a sealed vessel. The glass vial was positioned approximately 3 cm away from a 50 W blue LEDs lamp ($\lambda_{max}$ = 400 nm). After being stirred at room temperature (~ 30 °C under irradiation) for 12 h, the reaction mixture was purified by flash chromatography on silica gel (eluted with PE) to afford product **2c** (27.9 mg, 0.156 mmol, 78% yield, 91% D) as a white solid.

$^1$H NMR (500 MHz, $CDCl_3$) δ 8.73 (d, *J* = 8.2 Hz, 2H), 7.94 (d, *J* = 7.8 Hz, 2H), 7.78 (s, 1.09H), 7.67 (dt, *J* = 27.2, 7.4 Hz, 4H).

$^{13}$C NMR (151 MHz, $CDCl_3$) δ 132.13, 132.07, 130.38, 128.65, 128.60, 127.00, 126.87, 126.64, 126.52, 122.74.

$^2$H NMR (92 MHz, $CH_2Cl_2$) δ 7.71 (s, 1D).

IR (film): ν ($cm^{-1}$) 3052, 2954, 1714, 1277, 1114, 1038, 771,699, 610.

HRMS (ESI-TOF, m/z) calcd for $C_{14}H_{10}D$ $(M+H)^+$: 180.0918, found: 180.0910.

**methyl benzoate-4-D (2d)**

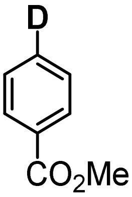

**2d**

A dried 5 mL glass vial was charged with aryl chloride **1d** (34.1 mg, 0.20 mmol), photocatalyst **PC1** (8.7 mg, 0.020 mmol), $(MeS)_2$ (6 μL, 0.060 mmol), $HCO_2Na$ (27.2 mg,

0.40 mmol), $D_2O$ (200 μL) and DMSO (1.0 mL) under air and then performed in a sealed vessel. The glass vial was positioned approximately 3 cm away from a 50 W blue LEDs lamp ($\lambda_{max}$ = 400 nm). After being stirred at room temperature (~ 30 °C under irradiation) for 10 h, the reaction mixture was purified by flash chromatography on silica gel (eluted with PE:EtOAc = 10:1) to afford product **2d** (18.4 mg, 0.134 mmol, 67% yield, 88% D) as a pale yellow oil.

$^1$H NMR (600 MHz, $CDCl_3$) δ 8.04 (d, $J$ = 8.2 Hz, 2H), 7.56 (t, 0.12H), 7.44 (d, $J$ = 8.0 Hz, 2H), 3.92 (s, 3H).

$^{13}$C NMR (151 MHz, $CDCl_3$) δ 167.16, 132.63 (t, $J$ = 24.3 Hz), 130.17, 129.59, 128.26, 52.13.

$^2$H NMR (92 MHz, $CH_2Cl_2$) δ 7.80 (s, 1D).

IR (film): ν (cm$^{-1}$) 2963, 1725, 1437, 1262, 1091, 1024, 801, 706

HRMS (ESI-TOF, m/z) calcd for $C_8H_8DO_2$ $(M+H)^+$: 138.0660, found: 138.0666.

***N,N*-dimethylbenzamide-4-D (2e)**

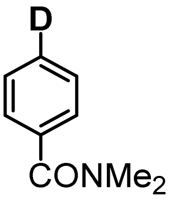

**2e**

A dried 5 mL glass vial was charged with aryl chloride **1e** (36.7 mg, 0.20 mmol), photocatalyst **PC1** (8.7 mg, 0.020 mmol), $(MeS)_2$ (6 μL, 0.060 mmol), $HCO_2Na$ (27.2 mg, 0.40 mmol), $D_2O$ (200 μL) and DMSO (1.0 mL) under air and then performed in a sealed vessel. The glass vial was positioned approximately 3 cm away from a 50 W blue LEDs lamp ($\lambda_{max}$ = 400 nm). After being stirred at room temperature (~ 30 °C under irradiation) for 10 h, the reaction mixture was purified by flash chromatography on silica gel (eluted with PE:EtOAc = 3:1) to afford product **2e** (25.5 mg, 0.170 mmol, 85% yield, 92% D) as a colorless oil.

$^1$H NMR (500 MHz, $CDCl_3$) δ 7.67 – 7.28 (m, 4.08H), 3.02 (d, $J$ = 50.5 Hz, 6H).

$^{13}C$ NMR (151 MHz, $CDCl_3$) δ 171.66, 136.31, 129.22 (t, $J$ = 24.1 Hz), 128.22, 127.02, 39.57, 35.33.

$^{2}H$ NMR (92 MHz, $CH_2Cl_2$) δ 7.61 (s, 1D).

IR (film): ν ($cm^{-1}$) 3027, 2931, 1632, 1507, 1478, 1394, 1265, 1217, 1083, 1026, 867, 617.

HRMS (ESI-TOF, m/z) calcd for $C_9H_{11}DNO$ $(M+H)^+$: 151.0976, found: 151.0980.

**diethyl (phenyl-4-D)phosphonate (2f)**

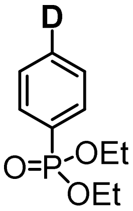

**2f**

A dried 5 mL glass vial was charged with aryl chloride **1f** (49.7 mg, 0.20 mmol), photocatalyst **PC1** (8.7 mg, 0.020 mmol), $(MeS)_2$ (6 μL, 0.060 mmol), $HCO_2Na$ (27.2 mg, 0.40 mmol), $D_2O$ (200 μL) and DMSO (1.0 mL) under air and then performed in a sealed vessel. The glass vial was positioned approximately 3 cm away from a 50 W blue LEDs lamp ($\lambda_{max}$ = 400 nm). After being stirred at room temperature (~ 30 °C under irradiation) for 10 h, the reaction mixture was purified by flash chromatography on silica gel (eluted with PE:EtOAc = 3:1) to afford product **2f** (37.0 mg, 0.172 mmol, 86% yield, 83% D) as a colorless oil.

$^{1}H$ NMR (500 MHz, $CDCl_3$) δ 7.78 (dd, $J$ = 13.3, 7.9 Hz, 2H), 7.51 (dd, $J$ = 7.2, 1.0 Hz, 0.17H), 7.43 (dd, $J$ = 7.8, 4.0 Hz, 2H), 4.20 – 3.98 (m, 4H), 1.29 (t, $J$ = 7.1 Hz, 6H).

$^{13}C$ NMR (126 MHz, $CDCl_3$) δ 132.36 (d, $J$ = 2.9 Hz), 131.73 (d, $J$ = 9.8 Hz), 128.37 (t, $J$ = 75.3 Hz), 128.33 (d, $J$ = 14.9 Hz), 62.09 (d, $J$ = 5.4 Hz), 16.29 (d, $J$ = 6.5 Hz).

$^{2}H$ NMR (92 MHz, $CH_2Cl_2$) δ 7.61 (s, 1D).

$^{31}P$ NMR (202 MHz, $CDCl_3$) δ 18.78.

IR (film): ν ($cm^{-1}$) 2984, 2931, 2908, 1479, 1394, 1247, 1164, 1133, 1053, 1024, 866, 769, 619.

HRMS (ESI-TOF, m/z) calcd for $C_{10}H_{14}DNaO_3P$ $(M+Na)^+$: 238.0714, found: 238.0711.

**anisole-4-D (2g)**

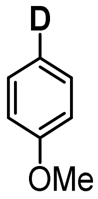


**2g**

A dried 10 mL Schlenk tube was charged with aryl chloride **1g** (28.5 mg, 0.20 mmol), photocatalyst **PC4** (11.2 mg, 0.040 mmol), ($n$PrS)$_2$ (10 µL, 0.060 mmol), $HCO_2Na$ (27.2 mg, 0.40 mmol), $D_2O$ (200 µL) and DMSO (1.0 mL). The mixture was degassed *via* three freeze-pump-thaw cycles. The Schlenk tube was positioned approximately 3 cm away from a 50 W blue LEDs lamp ($\lambda_{max}$ = 400 nm). After being stirred at room temperature (~ 30 °C under irradiation) for 72 h, the mixture was detected by crude $^1$H NMR analysis. The product **2g** was formed determined by $^1$H NMR (74% yield, 63% D)

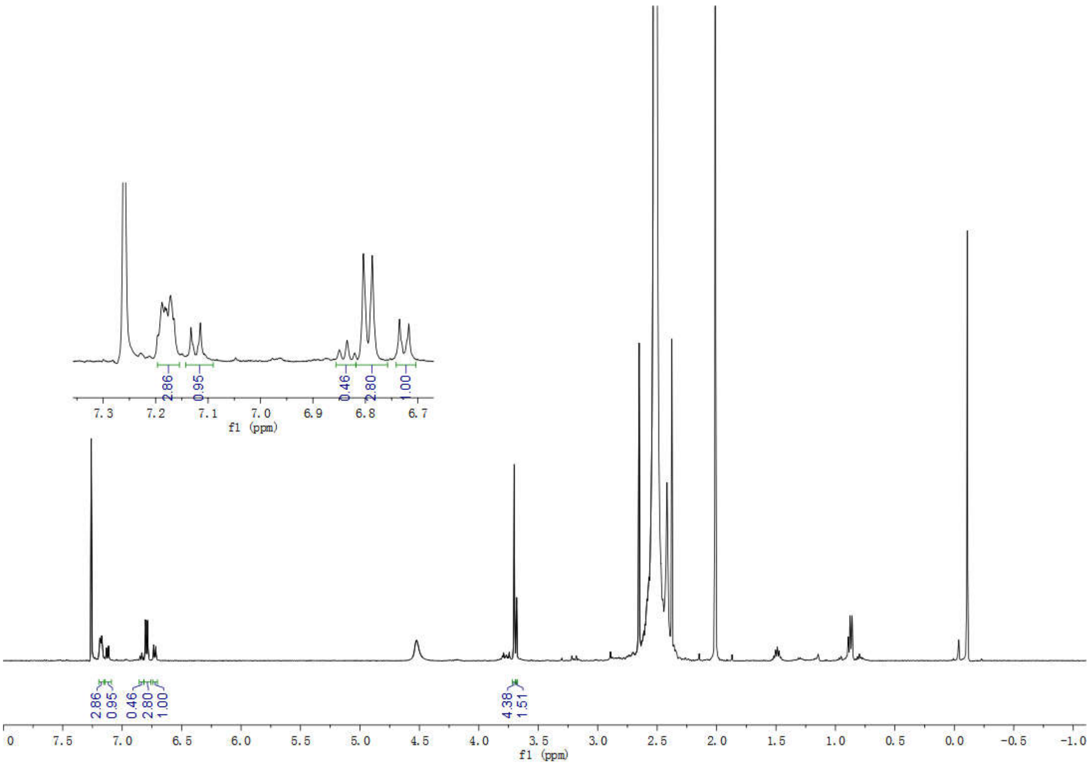


**Supplementary Fig. 1** Crude $^1$H NMR of **2g**.

**4-methoxy-1,1'-biphenyl-4'-D (2h)**

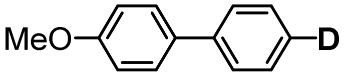

**2h**

A dried 5 mL glass vial was charged with aryl chloride **1h** (43.7 mg, 0.20 mmol), photocatalyst **PC1** (8.7 mg, 0.020 mmol), $(MeS)_2$ (6 μL, 0.060 mmol), $HCO_2Na$ (27.2 mg, 0.40 mmol), $D_2O$ (200 μL) and DMSO (1.0 mL) under air and then performed in a sealed vessel. The glass vial was positioned approximately 3 cm away from a 50 W blue LEDs lamp ($\lambda_{max}$ = 400 nm). After being stirred at room temperature (~ 30 °C under irradiation) for 15 h, the reaction mixture was purified by flash chromatography on silica gel (eluted with PE) to afford product **2h** (28.9 mg, 0.156 mmol, 78% yield, 85% D) as a white solid.

$^1H$ NMR (600 MHz, $CDCl_3$) δ 7.64 – 7.55 (m, 4H), 7.49 – 7.43 (m, 2H), 7.35 (dd, *J* = 10.5, 4.3 Hz, 0.15H), 7.06 – 6.99 (m, 2H), 3.88 (s, 3H).

$^{13}C$ NMR (151 MHz, $CDCl_3$) δ 159.23, 140.90, 133.84, 128.70, 128.23, 126.80, 126.37 (t, *J* = 24.3 Hz), 114.29, 55.39.

$^2H$ NMR (92 MHz, $CH_2Cl_2$) δ 7.51 (s, 1D).

IR (film): ν ($cm^{-1}$) 3047, 2961, 2936, 2836, 2048, 1609, 1518, 1485, 1290, 1265, 1246, 1183, 829, 738, 610.

**2-(benzyloxy)pyridine-6-D (2i)**

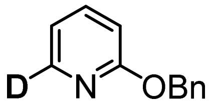

**2i**

A dried 10 mL Schlenk tube was charged with aryl chloride **1i** (43.9 mg, 0.20 mmol), photocatalyst **PC4** (11.2 mg, 0.040 mmol), $(nPrS)_2$ (10 μL, 0.060 mmol), $HCO_2Na$ (27.2 mg, 0.40 mmol), $D_2O$ (200 μL) and DMSO (1.0 mL). The mixture was degassed *via* three freeze-pump-thaw cycles. The Schlenk tube was positioned approximately 3 cm away from a 50 W blue LEDs lamp ($\lambda_{max}$ = 400 nm). After being stirred at room temperature (~ 30 °C under irradiation) for 48 h, the reaction mixture was purified by flash chromatography on silica gel

(eluted with PE:EtOAc = 20:1) to afford product **2i** (23.5 mg, 0.126 mmol, 63% yield, 84% D) as a colorless oil.

$^{1}$H NMR (500 MHz, $CDCl_3$) δ 8.10 (d, *J* = 3.7 Hz, 0.16H), 7.53 – 7.47 (m, 1H), 7.39 (d, *J* = 7.3 Hz, 2H), 7.30 (t, *J* = 7.4 Hz, 2H), 7.24 (t, *J* = 7.3 Hz, 1H), 6.81 (d, *J* = 7.0 Hz, 1H), 6.74 (d, *J* = 8.3 Hz, 1H), 5.31 (s, 2H).

$^{13}$C NMR (151 MHz, $CDCl_3$) δ 163.58, 146.40 (t, *J* = 27.3 Hz), 138.81, 137.28, 128.49, 127.97, 127.87, 116.81, 111.41, 67.67.

$^{2}$H NMR (92 MHz, $CH_2Cl_2$) δ 8.01 (s, 1D).

IR (film): ν ($cm^{-1}$) 2926, 1589, 1569, 1439, 1356, 1282, 1256, 992, 817.

HRMS (ESI-TOF, m/z) calcd for $C_{12}H_{11}DNO$ $(M+H)^+$: 187.0976, found: 187.0949.

**ethyl nicotinate-6-D (2j)**

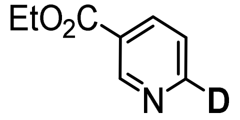


**2j**

A dried 5 mL glass vial was charged with aryl chloride **1j** (37.1 mg, 0.20 mmol), photocatalyst **PC1** (8.7 mg, 0.020 mmol), $(MeS)_2$ (6 μL, 0.060 mmol), $HCO_2Na$ (27.2 mg, 0.40 mmol), $D_2O$ (200 μL) and DMSO (1.0 mL) under air and then performed in a sealed vessel. The glass vial was positioned approximately 3 cm away from a 50 W blue LEDs lamp ($\lambda_{max}$ = 400 nm). After being stirred at room temperature (~ 30 °C under irradiation) for 12 h, the reaction mixture was purified by flash chromatography on silica gel (eluted with PE:EtOAc = 3:1) to afford product **2j** (23.4 mg, 0.154 mmol, 77% yield, 92% D) as a pale yellow oil.

$^{1}$H NMR (500 MHz, $CDCl_3$) δ 9.22 (d, *J* = 1.6 Hz, 1H), 8.76 (dd, *J* = 4.9, 1.7 Hz, 0.08H), 8.29 (dd, *J* = 7.9, 2.2 Hz, 1H), 7.38 (d, *J* = 7.9 Hz, 1H), 4.41 (q, *J* = 7.1 Hz, 2H), 1.40 (t, *J* = 7.1 Hz, 3H).

$^{13}$C NMR (151 MHz, $CDCl_3$) δ 165.25, 152.85 (t, *J* = 27.7 Hz), 150.80, 137.15, 126.42, 123.18, 61.49, 14.27.

$^2H$ NMR (92 MHz, $CH_2Cl_2$) δ 8.81 (s, 1D).

IR (film): ν ($cm^{-1}$) 2963, 1724, 1590, 1462, 1371, 1278, 1115, 1025, 801, 735,

HRMS (ESI-TOF, m/z) calcd for $C_8H_9DNO_2$ $(M+H)^+$: 153.0769, found: 153.0769.

**methyl isonicotinate-2-D (2k)**

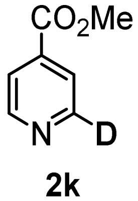


**2k**

A dried 5 mL glass vial was charged with aryl chloride **1k** (34.3 mg, 0.20 mmol), photocatalyst **PC1** (8.7 mg, 0.020 mmol), $(MeS)_2$ (6 μL, 0.060 mmol), $HCO_2Na$ (27.2 mg, 0.40 mmol), $D_2O$ (200 μL) and DMSO (1.0 mL) under air and then performed in a sealed vessel. The glass vial was positioned approximately 3 cm away from a 50 W blue LEDs lamp ($\lambda_{max}$ = 400 nm). After being stirred at room temperature (~ 30 °C under irradiation) for 31 h, the reaction mixture was purified by flash chromatography on silica gel (eluted with PE:EtOAc = 3:1) to afford product **2k** (19.9 mg, 0.144 mmol, 72% yield, 99% D) as a pale yellow oil.

$^1H$ NMR (600 MHz, $CDCl_3$) δ 8.79 (s, 1.01H), 7.87 (s, 2H), 3.96 (s, 3H).

$^{13}C$ NMR (151 MHz, $CDCl_3$) δ 165.45, 150.18, 149.85 (t, $J$ = 28.9 Hz), 137.70, 123.12, 122.97, 52.82.

$^2H$ NMR (92 MHz, $CH_2Cl_2$) δ 8.74 (s, 1D).

IR (film): ν ($cm^{-1}$) 2954, 2926, 2854, 1734, 1437, 1374, 1294, 1269, 1119, 1056, 782, 751.

HRMS (ESI-TOF, m/z) calcd for $C_7H_7DNO_2$ $(M+H)^+$: 139.0612, found: 139.0612.

**4-phenylpyridine-2-D (2l)**

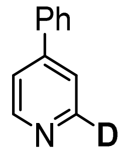


**2l**

A dried 5 mL glass vial was charged with aryl chloride **1l** (37.9 mg, 0.20 mmol), photocatalyst **PC1** (8.7 mg, 0.020 mmol), $(MeS)_2$ (6 μL, 0.060 mmol), $HCO_2Na$ (27.2 mg, 0.40 mmol), $D_2O$ (200 μL) and DMSO (1.0 mL) under air and then performed in a sealed vessel. The glass vial was positioned approximately 3 cm away from a 50 W blue LEDs lamp ($\lambda_{max}$ = 400 nm). After being stirred at room temperature (~ 30 °C under irradiation) for 31 h, the reaction mixture was purified by flash chromatography on silica gel (eluted with PE:EtOAc = 5:1) to afford product **2l** (24.7 mg, 0.158 mmol, 79% yield, 90% D) as a white solid.

$^1$H NMR (600 MHz, $CDCl_3$) δ 8.65 (d, *J* = 5.9 Hz, 1.1H), 7.63 (dd, *J* = 5.2, 3.3 Hz, 2H), 7.55 – 7.38 (m, 5H).

$^{13}$C NMR (151 MHz, $CDCl_3$) δ 150.24, 149.90 (t, *J* = 27.6 Hz), 148.39, 138.14, 129.14, 129.09, 127.01, 121.68, 121.54.

IR (film): ν (cm$^{-1}$) 3066, 2923, 2850, 2248, 1958, 1578, 1537, 1365, 1155, 1099, 898.

HRMS (ESI-TOF, m/z) calcd for $C_{11}H_9DN$ $(M+H)^+$: 157.0871, found: 187.0873.

**methyl nicotinate-2-D (2m)**

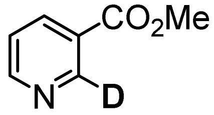


**2m**

A dried 5 mL glass vial was charged with aryl chloride **1m** (34.3 mg, 0.20 mmol), photocatalyst **PC1** (8.7 mg, 0.020 mmol), $(MeS)_2$ (6 μL, 0.060 mmol), $HCO_2Na$ (27.2 mg, 0.40 mmol), $D_2O$ (200 μL) and DMSO (1.0 mL) under air and then performed in a sealed vessel. The glass vial was positioned approximately 3 cm away from a 50 W blue LEDs lamp

($\lambda_{max}$ = 400 nm). After being stirred at room temperature (~ 30 °C under irradiation) for 5 h, the reaction mixture was purified by flash chromatography on silica gel (eluted with PE:EtOAc = 3:1) to afford product **2m** (18.8 mg, 0.136 mmol, 68% yield, 91% D) as a pale yellow oil.

$^1$H NMR (500 MHz, $CDCl_3$) δ 9.20 (s, 1H), 8.76 (s, 0.09H), 8.28 (d, $J$ = 7.9 Hz, 1H), 7.38 (dd, $J$ = 7.7, 4.9 Hz, 1H), 3.93 (s, 3H).

$^{13}$C NMR (126 MHz, $CDCl_3$) δ 165.69, 153.27, 150.45 (t, $J$ = 28.6 Hz), 137.14, 126.01, 123.37, 52.42.

$^2$H NMR (92 MHz, $CH_2Cl_2$) δ 8.90 (s, 1D).

IR (film): ν (cm$^{-1}$) 2955, 1727, 1582, 1460, 1438, 1408, 1218, 1144, 1144, 814, 616.

HRMS (ESI-TOF, m/z) calcd for $C_7H_7DNO_2$ $(M+H)^+$: 139.0612, found: 139.0613.

***N,N*-dimethylnicotinamide-6-D (2n)**

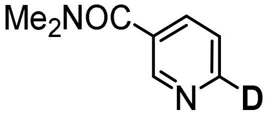


**2n**

A dried 5 mL glass vial was charged with aryl chloride **1n** (36.9 mg, 0.20 mmol), photocatalyst **PC1** (8.7 mg, 0.020 mmol), $(MeS)_2$ (6 μL, 0.060 mmol), $HCO_2Na$ (27.2 mg, 0.40 mmol), $D_2O$ (200 μL) and DMSO (1.0 mL) under air and then performed in a sealed vessel. The glass vial was positioned approximately 3 cm away from a 50 W blue LEDs lamp ($\lambda_{max}$ = 400 nm). After being stirred at room temperature (~ 30 °C under irradiation) for 10 h, the reaction mixture was purified by flash chromatography on silica gel (eluted with PE:EtOAc = 1:1) to afford product **2n** (22.7 mg, 0.150 mmol, 75% yield, 92% D) as a pale yellow oil.

$^1$H NMR (600 MHz, $CDCl_3$) δ 8.64 (s, 1H), 8.61 (d, $J$ = 4.6 Hz, 0.08H), 7.73 (dd, $J$ = 7.8, 2.2 Hz, 1H), 7.32 (d, $J$ = 7.8 Hz, 1H), 3.09 (s, 3H), 2.97 (s, 3H).

$^{13}$C NMR (151 MHz, $CDCl_3$) δ 168.96, 150.23 (t, $J$ = 26.9 Hz), 147.96, 134.98, 132.14, 123.24, 39.53, 35.43.

$^{2}H$ NMR (92 MHz, $CH_2Cl_2$) δ 8.97 (s, 1D).

IR (film): ν ($cm^{-1}$) 2934, 1625, 1507, 1449, 1401, 1268, 1096, 1036, 918, 742, 703.

HRMS (ESI-TOF, m/z) calcd for $C_8H_{10}DN_2O$ $(M+H)^+$: 152.0929, found: 152.0929.

**morpholino(pyridin-3-yl-6-D)methanone (2o)**

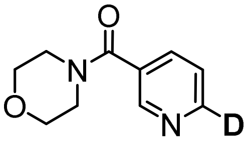

**2o**

A dried 5 mL glass vial was charged with aryl chloride **1o** (45.3 mg, 0.20 mmol), photocatalyst **PC1** (8.7 mg, 0.020 mmol), $(MeS)_2$ (6 μL, 0.060 mmol), $HCO_2Na$ (27.2 mg, 0.40 mmol), $D_2O$ (200 μL) and DMSO (1.0 mL) under air and then performed in a sealed vessel. The glass vial was positioned approximately 3 cm away from a 50 W blue LEDs lamp ($\lambda_{max}$ = 400 nm). After being stirred at room temperature (~ 30 °C under irradiation) for 6 h, the reaction mixture was purified by flash chromatography on silica gel (eluted with PE:EtOAc = 1:2) to afford product **2o** (33.4 mg, 0.188 mmol, 94% yield, 93% D) as a pale yellow oil.

$^{1}H$ NMR (600 MHz, $CDCl_3$) δ 8.60 (s, 1.07H), 7.77 – 7.65 (m, 1H), 7.32 (d, $J$ = 7.8 Hz, 1H), 3.79 – 3.35 (m, 8H).

$^{13}C$ NMR (151 MHz, $CDCl_3$) δ 167.66, 150.39 (t, $J$ = 27.6 Hz), 147.76, 135.30, 131.23, 123.48, 66.73, 48.21, 42.61.

$^{2}H$ NMR (92 MHz, $CH_2Cl_2$) δ 8.55 (s, 1D).

IR (film): ν ($cm^{-1}$) 2969, 2924, 2859, 1629, 1301, 1280, 1115, 1013, 938, 842, 646.

HRMS (ESI-TOF, m/z) calcd for $C_{10}H_{12}DN_2O_2$ $(M+H)^+$: 194.1034, found: 194.1034.

**isoquinoline-1-D (2p)**

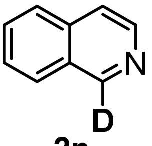

**2p**

A dried 5 mL glass vial was charged with aryl chloride **1p** (32.7 mg, 0.20 mmol), photocatalyst **PC1** (8.7 mg, 0.020 mmol), $(MeS)_2$ (6 μL, 0.060 mmol), $HCO_2Na$ (27.2 mg, 0.40 mmol), $D_2O$ (200 μL) and DMSO (1.0 mL) under air and then performed in a sealed vessel. The glass vial was positioned approximately 3 cm away from a 50 W blue LEDs lamp ($\lambda_{max}$ = 400 nm). After being stirred at room temperature (~ 30 °C under irradiation) for 10 h, the reaction mixture was purified by flash chromatography on silica gel (eluted with PE:EtOAc = 10:1) to afford product **2p** (19.0 mg, 0.146 mmol, 73% yield, 93% D) as a pale yellow oil.

$^1$H NMR (500 MHz, $CDCl_3$) δ 9.26 (s, 0.07H), 8.53 (d, *J* = 5.8 Hz, 1H), 7.98 (d, *J* = 8.2 Hz, 1H), 7.83 (d, *J* = 8.2 Hz, 1H), 7.73 – 7.68 (m, 1H), 7.65 (d, *J* = 5.7 Hz, 1H), 7.63 – 7.58 (m, 1H).

$^{13}$C NMR (151 MHz, $CDCl_3$) δ 151.98 (t, *J* = 26.6 Hz), 142.67, 135.86, 130.53, 128.55, 127.67, 127.36, 126.50, 120.62.

$^2$H NMR (92 MHz, $CH_2Cl_2$) δ 9.45 (s, 1D).

IR (film): ν ($cm^{-1}$) 3054, 2925, 2852, 1676, 1625, 1587, 1561, 1497, 1382, 1320, 1260, 829.

HRMS (ESI-TOF, m/z) calcd for $C_9H_7DN$ $(M+H)^+$: 131.0714, found: 131.0714.

**2-phenylpyrimidine-4-D (2q)**

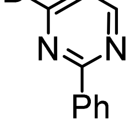

**2q**

A dried 5 mL glass vial was charged with aryl chloride **1q** (38.1 mg, 0.20 mmol), photocatalyst **PC1** (8.7 mg, 0.020 mmol), $(MeS)_2$ (6 μL, 0.060 mmol), $HCO_2Na$ (27.2 mg,

0.40 mmol), $D_2O$ (200 μL) and DMSO (1.0 mL) under air and then performed in a sealed vessel. The glass vial was positioned approximately 3 cm away from a 50 W blue LEDs lamp ($\lambda_{max}$ = 400 nm). After being stirred at room temperature (~ 30 °C under irradiation) for 12 h, the reaction mixture was purified by flash chromatography on silica gel (eluted with PE:EtOAc = 3:1) to afford product **2q** (17.3 mg, 0.110 mmol, 55% yield, 98% D) as a pale yellow oil.

$^1$H NMR (600 MHz, $CDCl_3$) δ 8.80 (d, *J* = 4.8 Hz, 1.02H), 8.51 – 8.38 (m, 2H), 7.55 – 7.45 (m, 3H), 7.17 (d, *J* = 4.8 Hz, 1H).

$^{13}$C NMR (151 MHz, $CDCl_3$) δ 164.76, 157.29, 156.91 (t, *J* = 27.4 Hz), 137.56, 130.82, 128.64, 128.16, 118.98.

$^2$H NMR (92 MHz, $CH_2Cl_2$) δ 8.76 (s, 1D).

IR (film): ν (cm$^{-1}$) 3064, 3035, 2926, 2254, 1557, 1420, 1377, 1174, 1070, 1026, 728, 693.

HRMS (ESI-TOF, m/z) calcd for $C_{10}H_8DN_2$ $(M+H)^+$: 158.0823, found: 158.0823.

**2,4-diphenylpyrimidine-6-D (2r)**

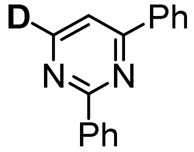


**2r**

A dried 5 mL glass vial was charged with aryl chloride **1r** (53.3 mg, 0.20 mmol), photocatalyst **PC1** (8.7 mg, 0.020 mmol), $(MeS)_2$ (6 μL, 0.060 mmol), $HCO_2Na$ (27.2 mg, 0.40 mmol), $D_2O$ (200 μL) and DMSO (1.0 mL) under air and then performed in a sealed vessel. The glass vial was positioned approximately 3 cm away from a 50 W blue LEDs lamp ($\lambda_{max}$ = 400 nm). After being stirred at room temperature (~ 30 °C under irradiation) for 31 h, the reaction mixture was purified by flash chromatography on silica gel (eluted with PE:EtOAc = 3:1) to afford product **2r** (41.1 mg, 0.176 mmol, 88% yield, 94% D) as a white solid.

$^{1}$H NMR (500 MHz, $CDCl_3$) δ 8.83 (d, $J$ = 5.2 Hz, 0.06H), 8.66 – 8.56 (m, 2H), 8.30 – 8.19 (m, 2H), 7.59 (s, 1H), 7.57 – 7.46 (m, 6H).

$^{13}$C NMR (151 MHz, $CDCl_3$) δ 164.62, 163.90, 157.52 (t, $J$ = 27.2 Hz), 137.91, 136.99, 130.99, 130.75, 128.97, 128.58, 128.33, 127.24, 114.41.

$^{2}$H NMR (92 MHz, $CH_2Cl_2$) δ 8.87 (s, 1D).

IR (film): ν ($cm^{-1}$) 3066, 2964, 2257, 1774, 1541, 1491, 1371, 1313, 1180, 936, 888, 735, 689.

HRMS (ESI-TOF, m/z) calcd for $C_{16}H_{12}DN_2$ $(M+H)^+$: 234.1136, found: 234.1136.

**6,7-dimethoxyquinazoline-4-D (2s)**

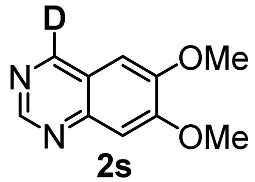


**2s**

A dried 10 mL Schlenk tube was charged with aryl chloride **1s** (44.9 mg, 0.20 mmol), photocatalyst **PC4** (11.2 mg, 0.040 mmol), ($n$PrS)$_2$ (10 μL, 0.060 mmol), $HCO_2Na$ (27.2 mg, 0.40 mmol), $D_2O$ (200 μL) and DMSO (1.0 mL). The mixture was degassed *via* three freeze-pump-thaw cycles. The Schlenk tube was positioned approximately 3 cm away from a 50 W blue LEDs lamp ($\lambda_{max}$ = 400 nm). After being stirred at room temperature (~ 30 °C under irradiation) for 70 h, the reaction mixture was purified by flash chromatography on silica gel (eluted with PE:EtOAc = 10:1) to afford product **2s** (19.5 mg, 0.102 mmol, 51% yield, 90% D) as a white solid.

$^{1}$H NMR (500 MHz, $CDCl_3$) δ 9.12 (s, 1.1H), 7.29 (s, 1H), 7.08 (s, 1H), 4.04 (s, 3H), 4.02 (s, 3H).

$^{13}$C NMR (126 MHz, $CDCl_3$) δ 156.70, 156.29, 154.06, 150.71, 148.04, 120.97, 106.54, 103.76, 56.45, 56.25.

$^{2}$H NMR (92 MHz, $CH_2Cl_2$) δ 9.32 (s, 1D).

IR (film): ν ($cm^{-1}$) 2968, 2921, 2850, 2246, 1615, 1560, 1506, 1435, 1233, 1149, 1008, 848, 582.

HRMS (ESI-TOF, m/z) calcd for $C_{10}H_{10}DN_2O_2$ $(M+H)^+$: 192.0878, found: 192.0878.

**9-(tetrahydro-2H-pyran-2-yl)-9H-purine-6-D (2t)**

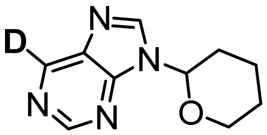

**2t**

A dried 10 mL Schlenk tube was charged with aryl chloride **1t** (47.7 mg, 0.20 mmol), photocatalyst **PC4** (11.2 mg, 0.040 mmol), ($n$PrS)$_2$ (10 μL, 0.060 mmol), $HCO_2Na$ (27.2 mg, 0.40 mmol), $D_2O$ (200 μL) and DMSO (1.0 mL). The mixture was degassed *via* three freeze-pump-thaw cycles. The Schlenk tube was positioned approximately 3 cm away from a 50 W blue LEDs lamp ($\lambda_{max}$ = 400 nm). After being stirred at room temperature (~ 30 °C under irradiation) for 48 h, the reaction mixture was purified by flash chromatography on silica gel (eluted with PE:EtOAc = 2:1) to afford product **2t** (22.2 mg, 0.108 mmol, 54% yield, 85% D) as a grey solid.

$^1$H NMR (500 MHz, $CDCl_3$) δ 9.10 (s, 0.15H), 8.95 (s, 1H), 8.29 (s, 1H), 5.78 (dd, $J$ = 10.3, 2.5 Hz, 1H), 4.23 – 4.03 (m, 1H), 3.83 – 3.66 (m, 1H), 2.15 – 1.99 (m, 3H), 1.80 – 1.60 (m, 3H).

$^{13}$C NMR (151 MHz, $CDCl_3$) δ 152.59, 150.64, 148.29 (t, $J$ = 28.6 Hz), 143.13, 134.02, 81.88, 68.84, 31.74, 24.82, 22.72.

$^2$H NMR (92 MHz, $CH_2Cl_2$) δ 9.42 (s, 1D).

IR (film): ν (cm$^{-1}$) 3107, 2945, 2856, 2263, 1584, 1492, 1332, 1211, 1085, 1058, 1045, 914, 644.

HRMS (ESI-TOF, m/z) calcd for $C_{10}H_{11}DN_4NaO$ $(M+Na)^+$: 228.0966, found: 228.0966.

**9-benzyl-9H-purine-6-D (2u)**

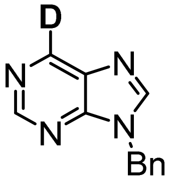

**2u**

A dried 10 mL Schlenk tube was charged with aryl chloride **1u** (48.9 mg, 0.20 mmol), photocatalyst **PC4** (11.2 mg, 0.040 mmol), (*n*PrS)$_2$ (10 μL, 0.060 mmol), $HCO_2Na$ (27.2 mg, 0.40 mmol), $D_2O$ (200 μL) and DMSO (1.0 mL). The mixture was degassed *via* three freeze-pump-thaw cycles. The Schlenk tube was positioned approximately 3 cm away from a 50 W blue LEDs lamp ($\lambda_{max}$ = 400 nm). After being stirred at room temperature (~ 30 °C under irradiation) for 48 h, the reaction mixture was purified by flash chromatography on silica gel (eluted with PE:EtOAc = 2:1) to afford product **2u** (16.9 mg, 0.080 mmol, 40% yield, 80% D) as a pale yellow oil.

$^1$H NMR (500 MHz, $CDCl_3$) δ 9.15 (s, 0.2H), 9.02 (s, 1H), 8.06 (s, 1H), 7.41 – 7.25 (m, 5H), 5.45 (s, 2H).

$^{13}$C NMR (126 MHz, $CDCl_3$) δ 152.66, 151.55, 148.10 (t, $J$ = 29.5 Hz), 145.24, 134.89, 133.83, 129.21, 128.72, 127.94, 47.26.

$^2$H NMR (92 MHz, $CH_2Cl_2$) δ 9.35 (s, 1D).

IR (film): ν (cm$^{-1}$) 1576, 1498, 1456, 1402, 1330, 1216, 727, 698.

HRMS (ESI-TOF, m/z) calcd for $C_{12}H_{10}DN_4$ $(M+H)^+$: 212.1041, found: 212.1041.

**thieno[3,2-d]pyrimidine-4-D (2v)**

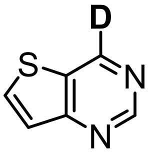

**2v**

A dried 5 mL glass vial was charged with aryl chloride **1v** (34.1 mg, 0.20 mmol), photocatalyst **PC1** (8.7 mg, 0.020 mmol), $(MeS)_2$ (6 μL, 0.060 mmol), $HCO_2Na$ (27.2 mg,

0.40 mmol), $D_2O$ (200 μL) and DMSO (1.0 mL) under air and then performed in a sealed vessel. The glass vial was positioned approximately 3 cm away from a 50 W blue LEDs lamp ($\lambda_{max}$ = 400 nm). After being stirred at room temperature (~ 30 °C under irradiation) for 12 h, the reaction mixture was purified by flash chromatography on silica gel (eluted with PE:EtOAc = 10:1) to afford product **2v** (21.9 mg, 0.160 mmol, 80% yield, 94% D) as a grey solid.

$^1$H NMR (500 MHz, $CDCl_3$) δ 9.29 (s, 0.06H), 9.22 (s, 1H), 8.03 (d, *J* = 5.5 Hz, 1H), 7.57 (d, *J* = 5.5 Hz, 1H).

$^{13}$C NMR (151 MHz, $CDCl_3$) δ 160.66, 154.30, 151.28 (t, *J* = 29.0 Hz), 136.79, 131.16, 124.45.

$^2$H NMR (92 MHz, $CH_2Cl_2$) δ 9.33 (s, 1D).

IR (film): ν ($cm^{-1}$) 3112, 2927, 2851, 1655, 1581, 1539, 1520, 1461, 1433, 1224, 1082, 1050, 814, 765, 573.

HRMS (ESI-TOF, m/z) calcd for $C_6H_4DN_2S$ $(M+H)^+$: 138.0231, found: 138.0233.

**6-(*tert*-butyl)thieno[2,3-d]pyrimidine-4-D (2w)**

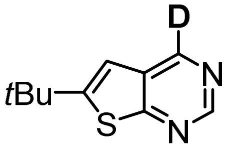


**2w**

A dried 5 mL glass vial was charged with aryl chloride **1w** (45.3 mg, 0.20 mmol), photocatalyst **PC1** (8.7 mg, 0.020 mmol), $(MeS)_2$ (6 μL, 0.060 mmol), $HCO_2Na$ (27.2 mg, 0.40 mmol), $D_2O$ (200 μL) and DMSO (1.0 mL) under air and then performed in a sealed vessel. The glass vial was positioned approximately 3 cm away from a 50 W blue LEDs lamp ($\lambda_{max}$ = 400 nm). After being stirred at room temperature (~ 30 °C under irradiation) for 18 h, the reaction mixture was purified by flash chromatography on silica gel (eluted with PE:EtOAc = 10:1) to afford product **2w** (15.1 mg, 0.078 mmol, 39% yield, 90% D) as a pale yellow oil.

$^1$H NMR (500 MHz, $CDCl_3$) δ 8.98 (s, 1H), 8.96 (s, 0.1H), 7.01 (s, 1H), 1.45 (s, 9H).

$^{13}C$ NMR (151 MHz, $CDCl_3$) δ 168.29, 160.14, 152.43, 149.68 (t, *J* = 28.4 Hz), 131.65, 113.24, 35.43, 31.85.

$^{2}H$ NMR (92 MHz, $CH_2Cl_2$) δ 8.99 (s, 1D).

IR (film): ν ($cm^{-1}$) 3042, 2964, 2868, 2248, 1878, 1541, 1504, 1464, 1357, 1254, 1147, 1120, 872, 716.

HRMS (ESI-TOF, m/z) calcd for $C_{10}H_{12}DN_2S$ $(M+H)^+$: 194.0857, found: 194.0859.

**5,6-dimethylthieno[2,3-d]pyrimidine-4-D (2x)**

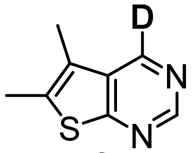


**2x**

A dried 5 mL glass vial was charged with aryl chloride **1x** (39.7 mg, 0.20 mmol), photocatalyst **PC1** (8.7 mg, 0.020 mmol), $(MeS)_2$ (6 μL, 0.060 mmol), $HCO_2Na$ (27.2 mg, 0.40 mmol), $D_2O$ (200 μL) and DMSO (1.0 mL) under air and then performed in a sealed vessel. The glass vial was positioned approximately 3 cm away from a 50 W blue LEDs lamp ($\lambda_{max}$ = 400 nm). After being stirred at room temperature (~ 30 °C under irradiation) for 13 h, the reaction mixture was purified by flash chromatography on silica gel (eluted with PE:EtOAc = 10:1) to afford product **2x** (23.1 mg, 0.140 mmol, 70% yield, 96% D) as a pale yellow oil.

$^{1}H$ NMR (500 MHz, $CDCl_3$) δ 8.99 (s, 1H), 8.88 (s, 0.04H), 2.51 (s, 3H), 2.33 (s, 3H).

$^{13}C$ NMR (151 MHz, $CDCl_3$) δ 167.68, 152.81, 148.51 (t, *J* = 27.1 Hz), 135.42, 132.31, 124.02, 13.77, 10.64.

$^{2}H$ NMR (92 MHz, $CH_2Cl_2$) δ 8.71 (s, 1D).

IR (film): ν ($cm^{-1}$) 2962, 2923, 2855, 2262, 1921, 1672, 1574, 1542, 1389, 1262, 1158, 1095, 1024, 965, 809, 713.

HRMS (ESI-TOF, m/z) calcd for $C_8H_8DN_2S$ $(M+H)^+$: 166.0544, found: 166.0544.

***Substrate scope of aryl bromides and iodides***

**phenanthrene-9-D (2c)**

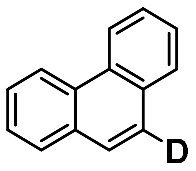


**2c**, X= Br

A dried 5 mL glass vial was charged with 9-bromophenanthrene (51.4 mg, 0.20 mmol), photocatalyst **PC1** (8.7 mg, 0.020 mmol), $(MeS)_2$ (6 μL, 0.060 mmol), $HCO_2Na$ (27.2 mg, 0.40 mmol), $D_2O$ (200 μL) and DMSO (1.0 mL) under air and then performed in a sealed vessel. The glass vial was positioned approximately 3 cm away from a 50 W blue LEDs lamp ($\lambda_{max}$ = 400 nm). After being stirred at room temperature (~ 30 °C under irradiation) for 12 h, the reaction mixture was purified by flash chromatography on silica gel (eluted with PE) to afford product **2c** (29.4 mg, 0.164 mmol, 82% yield, 89% D) as a white solid.

***N*,*N*-dimethylbenzamide-4-D (2e)**

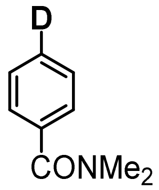


**2e**, X = Br

A dried 5 mL glass vial was charged with 4-bromo-*N*,*N*-dimethylbenzamide (45.6 mg, 0.20 mmol), photocatalyst **PC1** (8.7 mg, 0.020 mmol), $(MeS)_2$ (6 μL, 0.060 mmol), $HCO_2Na$ (27.2 mg, 0.40 mmol), $D_2O$ (200 μL) and DMSO (1.0 mL) under air and then performed in a sealed vessel. The glass vial was positioned approximately 3 cm away from a 50 W blue LEDs lamp ($\lambda_{max}$ = 400 nm). After being stirred at room temperature (~ 30 °C under irradiation) for 10 h, the reaction mixture was purified by flash chromatography on silica gel (eluted with PE:EtOAc = 3:1) to afford product **2e** (23.7 mg, 0.158 mmol, 79% yield, 92% D) as a colorless oil.

**2-(2-methyl-1,3-dioxolan-2-yl) pyridine-6-D (2y)**

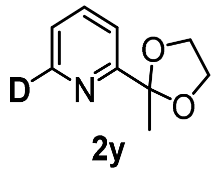

**2y**

A dried 5 mL glass vial was charged with 2-bromo-6-(2-methyl-1,3-dioxolan-2-yl)pyridine **1y** (48.8 mg, 0.20 mmol), photocatalyst **PC1** (8.7 mg, 0.020 mmol), $(MeS)_2$ (6 μL, 0.060 mmol), $HCO_2Na$ (27.2 mg, 0.40 mmol), $D_2O$ (200 μL) and DMSO (1.0 mL) under air and then performed in a sealed vessel. The glass vial was positioned approximately 3 cm away from a 50 W blue LEDs lamp ($\lambda_{max}$ = 400 nm). After being stirred at room temperature (~ 30 °C under irradiation) for 72 h, the reaction mixture was purified by flash chromatography on silica gel (eluted with PE:EtOAc = 3:1) to afford product **2y** (28.6 mg, 0.172 mmol, 86% yield, 85% D) as a pale yellow oil.

$^1$H NMR (500 MHz, $CDCl_3$) δ 8.64 (d, $J$ = 4.5 Hz, 0.15H), 7.69 (d, $J$ = 7.7 Hz, 1H), 7.56 (d, $J$ = 7.8 Hz, 1H), 7.22 (d, $J$ = 7.5 Hz, 1H), 4.14 – 4.05 (m, 2H), 3.92 – 3.85 (m, 2H), 1.74 (s, 3H).

$^{13}$C NMR (151 MHz, $CDCl_3$) δ 160.96, 149.07 (t, $J$ = 28.2 Hz), 136.59, 122.73, 119.45, 108.57, 64.91, 25.39.

$^2$H NMR (92 MHz, $CH_2Cl_2$) δ 8.65 (s, 1D).

IR (film): ν ($cm^{-1}$) 3062, 2988, 2937, 2887, 2250, 1582, 1435, 1370, 1280, 1201, 1040, 950, 873, 834, 729, 574.

HRMS (ESI-TOF, m/z) calcd for $C_9H_{11}DNO_2$ $(M+H)^+$: 167.0925, found: 167.0927.

**9-phenyl-9H-carbazole-3-D (2z)**

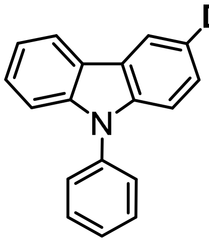

**2z**

A dried 5 mL glass vial was charged with 3-iodo-9-phenyl-9H-carbazole **1z** (73.8 mg, 0.20 mmol), photocatalyst **PC1** (8.7 mg, 0.020 mmol), $(MeS)_2$ (6 μL, 0.060 mmol), $HCO_2Na$ (27.2 mg, 0.40 mmol), $D_2O$ (200 μL) and DMSO (1.0 mL) under air and then performed in a sealed vessel. The glass vial was positioned approximately 3 cm away from a 50 W blue LEDs lamp ($\lambda_{max}$ = 400 nm). After being stirred at room temperature (~ 30 °C under irradiation) for 18 h, the reaction mixture was purified by flash chromatography on silica gel (eluted with PE:EtOAc = 10:1) to afford product **2z** (61.1 mg, 0.180 mmol, 90% yield, 87% D) as a pale yellow oil.

$^1$H NMR (600 MHz, $CDCl_3$) δ 8.38 – 8.15 (m, 2H), 7.71 – 7.58 (m, 4H), 7.56 – 7.40 (m, 5H), 7.39 – 7.33 (m, 1.13H).

$^{13}$C NMR (151 MHz, $CDCl_3$) δ 140.98, 137.79, 129.95, 127.52, 127.22, 126.01, 125.90, 123.44, 120.39, 120.28, 119.98, 119.72 (t, $J$ = 24.9 Hz), 109.85.

$^2$H NMR (92 MHz, $CH_2Cl_2$) δ 7.49 (s, 1D).

IR (film): ν ($cm^{-1}$) 3089, 2927, 2853, 2675, 2268, 1892, 1597, 1470, 1449, 1331, 1231, 1179, 1029, 749, 698, 647.

HRMS (ESI-TOF, m/z) calcd for $C_{18}H_{13}DN$ $(M+H)^+$: 245.1184, found: 245.1184.

**9,9-dimethyl-9H-fluorene-2-D (2za)**

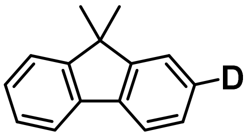

**2za**

A dried 5 mL glass vial was charged with 2-bromo-9,9-dimethyl-9H-fluorene **1za** (54.6 mg, 0.20 mmol), photocatalyst **PC1** (8.7 mg, 0.020 mmol), $(MeS)_2$ (6 μL, 0.060 mmol), $HCO_2Na$ (27.2 mg, 0.40 mmol), $D_2O$ (200 μL) and DMSO (1.0 mL) under air and then performed in a sealed vessel. The glass vial was positioned approximately 3 cm away from a 50 W blue LEDs lamp ($\lambda_{max}$ = 400 nm). After being stirred at room temperature (~ 30 °C under irradiation) for 15 h, the reaction mixture was purified by flash chromatography on silica gel (eluted with PE) to afford product **2za** (29.2 mg, 0.150 mmol, 75% yield, 95% D) as a white solid.

$^1$H NMR (500 MHz, $CDCl_3$) δ 7.79 (d, $J$ = 7.8 Hz, 2H), 7.55 – 7.47 (m, 2H), 7.39 (pd, $J$ = 7.3, 1.4 Hz, 3.05H), 1.55 (s, 6H).

$^{13}$C NMR (126 MHz, $CDCl_3$) δ 153.66, 139.27, 127.30, 127.01, 126.90, 122.66, 122.55, 120.06, 46.89, 27.23.

$^2$H NMR (92 MHz, $CH_2Cl_2$) δ 7.31 (s, 1D).

IR (film): ν (cm$^{-1}$) 3061, 3014, 2961, 2922, 2863, 2263, 1948, 1604, 1479, 1444, 1413, 1026, 783, 761,737,665.

HRMS (ESI-TOF, m/z) calcd for $C_{15}H_{14}D$ $(M+H)^+$: 196.1231, found: 196.1238.

**3.3 Optimization for Deuterodehalogenation of Alkyl Chlorides**

A dried 5 mL glass vial was charged with **3a** (19.1 mg 0.10 mmol), photocatalyst **PC** (0.020 mmol), $(RS)_2$ (0.060 mmol), $HCO_2Na$ (13.6 mg, 0.20 mmol), $D_2O$ (100 μL) and DMSO (500 μL) under air and then performed in a sealed vessel. The glass vial was positioned approximately 3 cm away from a 50 W blue LEDs lamp ($\lambda_{max}$ = 400 nm). The reactions were

stirred at room temperature (~ 30 °C under irradiation) for 15 h. The conversion was determined by $^1$H NMR analysis of the crude mixture. The reaction mixture was purified by silica gel chromatography (eluted with PE) to provide pure product **4a** for determining deuterium incorporation.

**Supplementary Table 2** Optimization for deuterodehalogenation of alkyl chlorides[a].

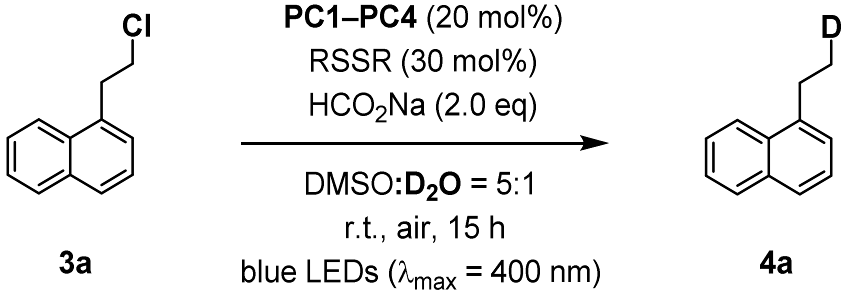


| entry | **PC** | RSSR | conv. (%)[b] | D-inc. (%)[c] |
|---|---|---|---|---|
| 1 | **PC1** | $(MeS)_2$ | quant. | 85 |
| 2 | **PC2** | $(MeS)_2$ | < 10 | n.a. |
| 3 | **PC3** | $(MeS)_2$ | quant. | 93 |
| 4 | **PC4** | $(MeS)_2$ | quant. | 93 |
| 5 | **PC4** | $(CyS)_2$ | quant. | 63 |
| 6 | **PC4** | $(n\text{PrS})_2$ | quant. | 94 |

[a]Reaction conditions: **3a** (0.10 mmol), $D_2O$ (0.10 mL), **PC1−PC4** (0.020 mmol), $(RS)_2$ (0.030 mmol), $HCO_2Na$ (0.20 mmol), DMSO (0.50 mL), 50 W blue LEDs lamp ($\lambda_{max}$ = 400 nm), under air and then performed in a sealed vessel.

[b]Conversion determined by $^1$H-NMR.

[c]Deuterium incorporation (D-inc.) determined by $^1$H-NMR, n.a. = not applicable.

### 3.4 Substrate Scope for Deuterodehalogenation of Alkyl Chlorides

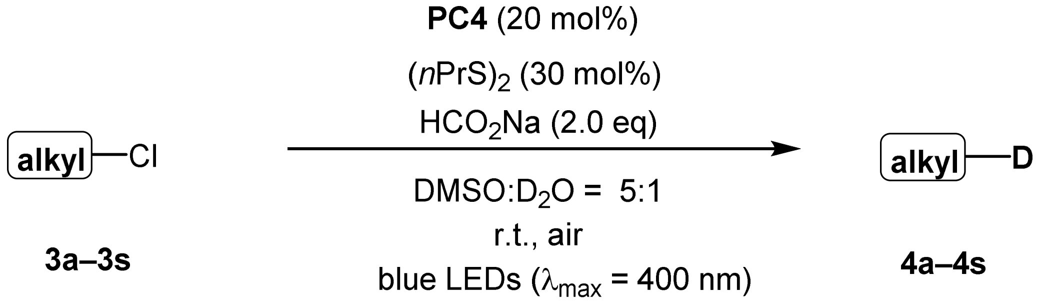


#### 3.4.1 General Procedure

A dried 5 mL glass vial was charged with alkyl chlorides **3a–3s** (0.20 mmol), photocatalyst **PC4** (11.2 mg, 0.040 mmol), (*n*PrS)$_2$ (10 μL, 0.060 mmol), $HCO_2Na$ (27.2 mg, 0.40 mmol), $D_2O$ (200 μL) and DMSO (1.0 mL) under air and then performed in a sealed vessel. The glass vial was positioned approximately 3 cm away from a 50 W blue LEDs lamp ($\lambda_{max}$ = 400 nm). After being stirred at room temperature (~ 30 °C under irradiation) for the indicated time, the reaction mixture was purified by flash chromatography on silica gel to afford product **4a–4s**.

#### 3.4.2 Experimental Details and Characterization Data

**1-(ethyl-2-D)naphthalene (4a)**

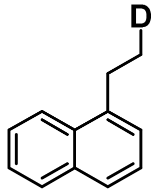


**4a**

A dried 5 mL glass vial was charged with alkyl chlorides **3a** (38.1 mg, 0.20 mmol), photocatalyst **PC4** (11.2 mg, 0.040 mmol), (*n*PrS)$_2$ (10 μL, 0.060 mmol), $HCO_2Na$ (27.2 mg, 0.40 mmol), $D_2O$ (200 μL) and DMSO (1.0 mL) under air and then performed in a sealed vessel. The glass vial was positioned approximately 3 cm away from a 50 W blue LEDs lamp ($\lambda_{max}$ = 400 nm). After being stirred at room temperature (~ 30 °C under irradiation) for 12 h,

the reaction mixture was purified by flash chromatography on silica gel (eluted with PE) to afford product **4a** (23.9 mg, 0.152 mmol, 76% yield, 94% D) as a colorless oil.

$^1$H NMR (600 MHz, $CDCl_3$) δ 8.07 (d, $J$ = 8.3 Hz, 1H), 7.87 (d, $J$ = 8.0 Hz, 1H), 7.72 (d, $J$ = 8.1 Hz, 1H), 7.50 (dt, $J$ = 23.4, 7.3 Hz, 2H), 7.42 (t, $J$ = 7.4 Hz, 1H), 7.36 (d, $J$ = 6.9 Hz, 1H), 3.13 (t, $J$ = 7.5 Hz, 2H), 1.43 – 1.36 (m, 2.06H).

$^{13}$C NMR (151 MHz, $CDCl_3$) δ 140.30, 133.84, 131.80, 128.76, 126.40, 125.69, 125.41, 124.86, 123.76, 25.84, 14.77 (t, $J$ = 20.0 Hz).

$^2$H NMR (92 MHz, $CH_2Cl_2$) δ 1.26 (s, 1D).

IR (film): ν (cm$^{-1}$) 3048, 2962, 2931, 2871, 2174, 1925, 1694, 1596, 1509, 1463, 1394, 1260, 794, 775.

HRMS (ESI-TOF, m/z) calcd for $C_{12}H_{11}DNa$ $(M+Na)^+$: 180.0894, found: 180.0894.

**4-(ethyl-2-D)-1,1'-biphenyl (4b)**

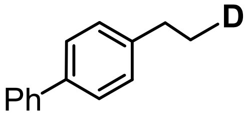


**4b**

A dried 5 mL glass vial was charged with alkyl chlorides **3b** (43.3 mg, 0.20 mmol), photocatalyst **PC4** (11.2 mg, 0.040 mmol), ($n$PrS)$_2$ (10 μL, 0.060 mmol), $HCO_2Na$ (27.2 mg, 0.40 mmol), $D_2O$ (200 μL) and DMSO (1.0 mL) under air and then performed in a sealed vessel. The glass vial was positioned approximately 3 cm away from a 50 W blue LEDs lamp ($\lambda_{max}$ = 400 nm). After being stirred at room temperature (~ 30 °C under irradiation) for 12 h, the reaction mixture was purified by flash chromatography on silica gel (eluted with PE) to afford product **4b** (28.8 mg, 0.158 mmol, 79% yield, 96% D) as a colorless oil.

$^1$H NMR (500 MHz, $CDCl_3$) δ 7.71 – 7.65 (m, 2H), 7.61 (d, $J$ = 8.1 Hz, 2H), 7.51 (t, $J$ = 7.6 Hz, 2H), 7.41 (t, $J$ = 7.3 Hz, 1H), 7.36 (d, $J$ = 8.0 Hz, 2H), 2.78 (t, $J$ = 7.5 Hz, 2H), 1.39 – 1.33 (m, 2.04H).

$^{13}$C NMR (151 MHz, $CDCl_3$) δ 143.47, 141.30, 138.72, 128.81, 128.40, 127.19, 127.11, 127.07, 28.55, 15.41 (t, $J$ = 39.3 Hz).

$^2$H NMR (92 MHz, $CH_2Cl_2$) δ 1.19 (s, 1D).

IR (film): ν ($cm^{-1}$) 3028, 2960, 2932, 2857, 2177, 1487, 1450, 1117, 1009, 830, 762, 696.

HRMS (ESI-TOF, m/z) calcd for $C_{14}H_{14}D$ $(M+H)^+$: 184.1231, found: 184.1232.

**4-(propyl-3-D)-1,1'-biphenyl (4c)**

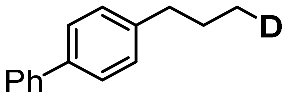


**4c**

A dried 5 mL glass vial was charged with alkyl chlorides **3c** (46.1 mg, 0.20 mmol), photocatalyst **PC4** (11.2 mg, 0.040 mmol), $(n\text{PrS})_2$ (10 μL, 0.060 mmol), $HCO_2Na$ (27.2 mg, 0.40 mmol), $D_2O$ (200 μL) and DMSO (1.0 mL) under air and then performed in a sealed vessel. The glass vial was positioned approximately 3 cm away from a 50 W blue LEDs lamp ($\lambda_{max}$ = 400 nm). After being stirred at room temperature (~ 30 °C under irradiation) for 12 h, the reaction mixture was purified by flash chromatography on silica gel (eluted with PE) to afford product **4c** (35.8 mg, 0.166 mmol, 83% yield, 90% D) as a colorless oil.

$^1$H NMR (500 MHz, $CDCl_3$) δ 7.54 (d, *J* = 7.4 Hz, 2H), 7.47 (d, *J* = 6.9 Hz, 2H), 7.38 (t, *J* = 6.9 Hz, 2H), 7.27 (t, *J* = 7.4 Hz, 1H), 7.21 (d, *J* = 7.1 Hz, 2H), 2.59 (t, *J* = 7.6 Hz, 2H), 1.73 – 1.58 (m, 2H), 0.97 – 0.88 (m, 2.1H).

$^{13}$C NMR (126 MHz, $CDCl_3$) δ 141.88, 141.24, 138.63, 128.92, 128.73, 128.62, 127.03, 127.00, 37.71, 24.51, 13.63 (t, *J* = 19.6 Hz).

$^2$H NMR (92 MHz, $CH_2Cl_2$) δ 0.86 (s, 1D).

IR (film): ν ($cm^{-1}$) 3027, 2955, 2927, 2854, 2177, 1487, 1261, 1076, 1009, 761, 698, 610, 503.

HRMS (ESI-TOF, m/z) calcd for $C_{15}H_{15}DNa$ $(M+Na)^+$: 220.1207, found: 220.1212.

**4-(ethoxy-2-D)-1,1'-biphenyl (4d)**

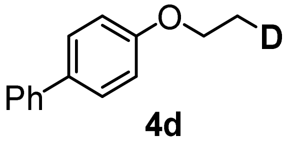


**4d**

A dried 5 mL glass vial was charged with alkyl chlorides **3d** (46.4 mg, 0.20 mmol), photocatalyst **PC4** (11.2 mg, 0.040 mmol), (*n*PrS)$_2$ (10 μL, 0.060 mmol), $HCO_2Na$ (27.2 mg, 0.40 mmol), $D_2O$ (200 μL) and DMSO (1.0 mL) under air and then performed in a sealed vessel. The glass vial was positioned approximately 3 cm away from a 50 W blue LEDs lamp ($\lambda_{max}$ = 400 nm). After being stirred at room temperature (~ 30 °C under irradiation) for 48 h, the reaction mixture was purified by flash chromatography on silica gel (eluted with PE:EtOAc = 40:1) to afford product **4d** (25.9 mg, 0.130 mmol, 65% yield, 90% D) as a white solid.

$^1$H NMR (600 MHz, $CDCl_3$) δ 7.65 – 7.48 (m, 4H), 7.45 – 7.38 (m, 2H), 7.31 (t, $J$ = 7.4 Hz, 1H), 7.04 – 6.91 (m, 2H), 4.08 (t, $J$ = 6.8 Hz, 2H), 1.47 – 1.42 (m, 2.1H).

$^{13}$C NMR (151 MHz, $CDCl_3$) δ 158.54, 140.90, 133.62, 128.73, 128.62, 128.15, 126.74, 126.63, 114.78, 63.49, 14.64 (t, $J$ = 19.9 Hz).

$^2$H NMR (92 MHz, $CH_2Cl_2$) δ 1.44 (s, 1D)

IR (film): ν (cm$^{-1}$) 3055, 2973, 2925, 2885, 2190, 1604, 1519, 1487, 1286, 1269, 1251, 1059, 833, 762.

HRMS (ESI-TOF, m/z) calcd for $C_{14}H_{13}DNaO$ $(M+Na)^+$: 222.1000, found: 222.1000.

**4-(propoxy-3-D)-1,1'-biphenyl (4e)**

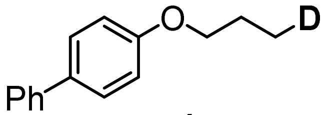


**4e**

A dried 5 mL glass vial was charged with alkyl chlorides **3e** (49.3 mg, 0.20 mmol), photocatalyst **PC4** (11.2 mg, 0.040 mmol), (*n*PrS)$_2$ (10 μL, 0.060 mmol), $HCO_2Na$ (27.2 mg, 0.40 mmol), $D_2O$ (200 μL) and DMSO (1.0 mL) under air and then performed in a sealed vessel. The glass vial was positioned approximately 3 cm away from a 50 W blue LEDs lamp

($\lambda_{max}$ = 400 nm). After being stirred at room temperature (~ 30 °C under irradiation) for 60 h, the reaction mixture was purified by flash chromatography on silica gel (eluted with PE:EtOAc = 40:1) to afford product **4e** (29.0 mg, 0.136 mmol, 68% yield, 97% D) as a white solid.

$^1$H NMR (600 MHz, $CDCl_3$) δ 7.68 – 7.51 (m, 4H), 7.44 (dd, *J* = 10.6, 4.9 Hz, 2H), 7.38 – 7.28 (m, 1H), 7.10 – 6.93 (m, 2H), 3.99 (t, *J* = 6.6 Hz, 2H), 1.95 – 1.75 (m, 2H), 1.11 – 1.05 (m, 2.03H).

$^{13}$C NMR (151 MHz, $CDCl_3$) δ 158.77, 140.94, 133.59, 128.75, 128.15, 126.75, 126.64, 114.82, 69.61, 22.59, 10.32 (t, *J* = 19.4 Hz).

$^2$H NMR (92 MHz, $CH_2Cl_2$) δ 1.06 (s, 1D).

IR (film): ν (cm$^{-1}$) 3032, 2939, 2876, 2181, 1608, 1518, 1390, 1288, 1266, 1246, 1175, 834, 745.

HRMS (ESI-TOF, m/z) calcd for $C_{15}H_{16}DO$ $(M+H)^+$: 214.1337, found: 214.1340.

**4-(butoxy-4-D)-1,1'-biphenyl (4f)**

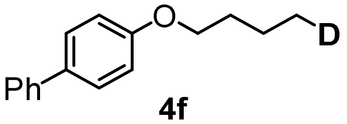

**4f**

A dried 5 mL glass vial was charged with alkyl chlorides **3f** (52.2 mg, 0.20 mmol), photocatalyst **PC4** (11.2 mg, 0.040 mmol), ($n$PrS)$_2$ (10 μL, 0.060 mmol), $HCO_2Na$ (27.2 mg, 0.40 mmol), $D_2O$ (200 μL) and DMSO (1.0 mL) under air and then performed in a sealed vessel. The glass vial was positioned approximately 3 cm away from a 50 W blue LEDs lamp ($\lambda_{max}$ = 400 nm). After being stirred at room temperature (~ 30 °C under irradiation) for 58 h, the reaction mixture was purified by flash chromatography on silica gel (eluted with PE:EtOAc = 40:1) to afford product **4f** (27.7 mg, 0.122 mmol, 61% yield, 98% D) as a white solid.

$^1$H NMR (500 MHz, $CDCl_3$) δ 7.64 – 7.51 (m, 4H), 7.43 (dd, *J* = 10.1, 5.3 Hz, 2H), 7.32 (t, *J* = 7.4 Hz, 1H), 7.00 (d, *J* = 8.6 Hz, 2H), 4.03 (t, *J* = 6.5 Hz, 2H), 1.87 – 1.77 (m, 2H), 1.60 – 1.48 (m, 2H), 1.04 – 0.97 (m, 2.02H).

$^{13}C$ NMR (126 MHz, $CDCl_3$) δ 158.78, 140.94, 133.58, 128.73, 128.13, 126.74, 126.62, 114.82, 67.82, 31.38, 19.23, 13.61 (t, *J* = 19.6 Hz).

$^{2}H$ NMR (92 MHz, $CH_2Cl_2$) δ 0.81 (s, 1D).

IR (film): ν ($cm^{-1}$) 3031, 2952, 2917, 2872, 2170, 1887, 1608, 1521, 1489, 1451, 1286, 1271, 1070, 827, 759, 689.

HRMS (ESI-TOF, m/z) calcd for $C_{16}H_{18}DO$ $(M+H)^+$: 228.1493, found: 228.1493.

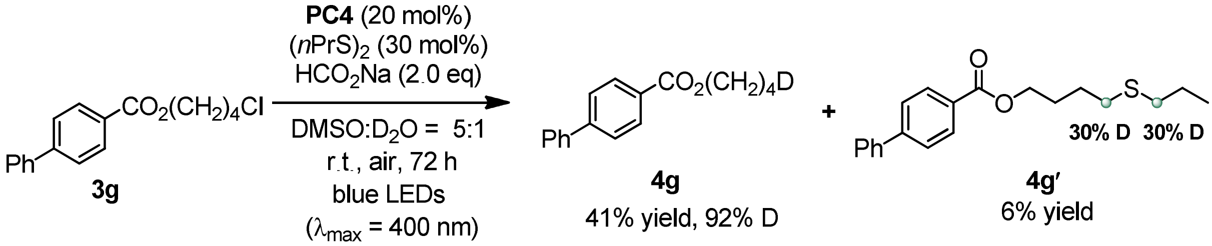


A dried 5 mL glass vial was charged with alkyl chlorides **3g** (57.8 mg, 0.20 mmol), photocatalyst **PC4** (11.2 mg, 0.040 mmol), (*n*PrS)$_2$ (10 μL, 0.060 mmol), $HCO_2Na$ (27.2 mg, 0.40 mmol), $D_2O$ (200 μL) and DMSO (1.0 mL) under air and then performed in a sealed vessel. The glass vial was positioned approximately 3 cm away from a 50 W blue LEDs lamp ($\lambda_{max}$ = 400 nm). After being stirred at room temperature (~ 30 °C under irradiation) for 72 h, the reaction mixture was purified by flash chromatography on silica gel (eluted with PE:EtOAc = 40:1) to afford product **4g** (21.0 mg, 0.082 mmol, 41% yield, 92% D) as a pale yellow oil and byproduct **4g'** (4.2 mg, 0.012 mmol, 6% yield) as a grey oil.

**butyl-4-D [1,1'-biphenyl]-4-carboxylate (4g)**

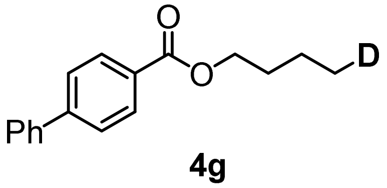


**4g**

$^{1}H$ NMR (600 MHz, $CDCl_3$) δ 8.12 (d, *J* = 8.2 Hz, 2H), 7.65 (dd, *J* = 21.5, 7.9 Hz, 4H), 7.47 (t, *J* = 7.6 Hz, 2H), 7.40 (t, *J* = 7.3 Hz, 1H), 4.36 (t, *J* = 6.6 Hz, 2H), 1.82 – 1.75 (m, 2H), 1.54 – 1.46 (m, 2H), 1.02 – 0.96 (m, 2.08H).

$^{13}C$ NMR (151 MHz, $CDCl_3$) δ 166.63, 145.56, 140.09, 130.08, 129.29, 128.94, 128.13, 127.30, 127.04, 64.90, 30.81, 19.24, 13.52 (t, *J* = 19.6 Hz).

$^{2}H$ NMR (92 MHz, $CH_2Cl_2$) δ 1.00 (s, 1D).

IR (film): ν ($cm^{-1}$) 3059, 3032, 2957, 2934, 2871, 2364, 2172, 1937, 1717, 1610, 1488, 1406, 1278, 1179, 1103, 859, 748, 698.

HRMS (ESI-TOF, m/z) calcd for $C_{17}H_{17}DNaO_2$ $(M+Na)^+$: 278.1262, found: 278.1263.

**4-(propylthio)butyl [1,1'-biphenyl]-4-carboxylate (4g', side product)**

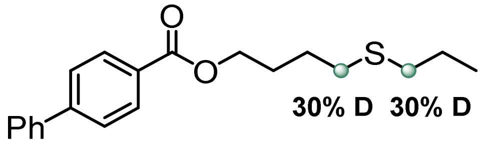


**4g′**

$^{1}H$ NMR (600 MHz, $CDCl_3$) δ 8.11 (d, *J* = 8.3 Hz, 2H), 7.64 (dd, *J* = 21.9, 7.9 Hz, 4H), 7.47 (t, *J* = 7.6 Hz, 2H), 7.40 (t, *J* = 7.3 Hz, 1H), 4.37 (t, *J* = 6.5 Hz, 2H), 2.60 (t, *J* = 7.3 Hz, 1.4H), 2.55 – 2.48 (m, 1.4H), 1.90 (dd, *J* = 14.8, 6.6 Hz, 2H), 1.78 (dd, *J* = 15.0, 7.6 Hz, 2H), 1.63 (dt, *J* = 14.7, 7.3 Hz, 2H), 0.99 (t, *J* = 7.3 Hz, 3H).

$^{13}C$ NMR (151 MHz, $CDCl_3$) δ 166.53, 145.66, 140.05, 130.09, 129.09, 128.95, 128.16, 127.30, 127.07, 64.56, 34.24, 31.71, 27.98, 26.24, 23.02, 13.55.

**pentyl-5-D [1,1'-biphenyl]-4-carboxylate (4h)**

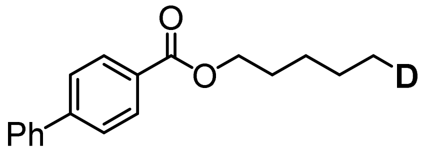


**4h**

A dried 5 mL glass vial was charged with alkyl chlorides **3h** (60.6 mg, 0.20 mmol), photocatalyst **PC4** (11.2 mg, 0.040 mmol), $(nPrS)_2$ (10 μL, 0.060 mmol), $HCO_2Na$ (27.2 mg, 0.40 mmol), $D_2O$ (200 μL) and DMSO (1.0 mL) under air and then performed in a sealed vessel. The glass vial was positioned approximately 3 cm away from a 50 W blue LEDs lamp ($\lambda_{max}$ = 400 nm). After being stirred at room temperature (~ 30 °C under irradiation) for 72 h,

the reaction mixture was purified by flash chromatography on silica gel (eluted with PE:EtOAc = 40:1) to afford product **4h** (20.5 mg, 0.076 mmol, 38% yield, 90% D) as a pale yellow oil.

$^1$H NMR (600 MHz, $CDCl_3$) δ 8.18 – 8.04 (m, 2H), 7.70 – 7.59 (m, 4H), 7.52 – 7.42 (m, 2H), 7.42 – 7.35 (m, 1H), 4.34 (t, $J$ = 6.7 Hz, 2H), 1.84 – 1.75 (m, 2H), 1.48 – 1.37 (m, 4H), 0.96 – 0.91 (m, 2.1H).

$^{13}$C NMR (151 MHz, $CDCl_3$) δ 166.63, 145.55, 140.09, 130.08, 129.30, 128.94, 128.13, 127.30, 127.04, 65.19, 28.49, 28.22, 22.32, 13.74 (t, $J$ = 18.7 Hz).

$^2$H NMR (92 MHz, $CH_2Cl_2$) δ 0.98 (s, 1D).

IR (film): ν (cm$^{-1}$) 3068, 3035, 2962, 2920, 2894, 2853, 2247, 1585, 1445, 1375, 1313, 1249, 1122, 982, 809, 776, 730.

HRMS (ESI-TOF, m/z) calcd for $C_{18}H_{19}DNaO_2$ $(M+Na)^+$: 292.1418, found: 292.1418.

**hexyl-6-D [1,1'-biphenyl]-4-carboxylate (4i)**

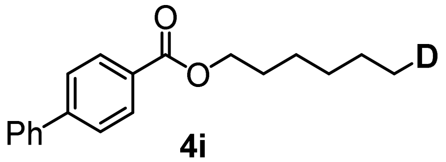

**4i**

A dried 5 mL glass vial was charged with alkyl chlorides **3i** (63.4 mg, 0.20 mmol), photocatalyst **PC4** (11.2 mg, 0.040 mmol), ($n$PrS)$_2$ (10 μL, 0.060 mmol), $HCO_2Na$ (27.2 mg, 0.40 mmol), $D_2O$ (200 μL) and DMSO (1.0 mL) under air and then performed in a sealed vessel. The glass vial was positioned approximately 3 cm away from a 50 W blue LEDs lamp ($\lambda_{max}$ = 400 nm). After being stirred at room temperature (~ 30 °C under irradiation) for 72 h, the reaction mixture was purified by flash chromatography on silica gel (eluted with PE:EtOAc = 40:1) to afford product **4i** (16.4 mg, 0.058 mmol, 29% yield, 88% D) as a pale yellow oil.

$^1$H NMR (600 MHz, $CDCl_3$) δ 8.18 – 7.98 (m, 2H), 7.72 – 7.58 (m, 4H), 7.47 (t, $J$ = 7.7 Hz, 2H), 7.40 (t, $J$ = 7.4 Hz, 1H), 4.34 (t, $J$ = 6.7 Hz, 2H), 1.82 – 1.75 (m, 2H), 1.50 – 1.43 (m, 2H), 1.39 – 1.33 (m, 4H), 0.92 – 0.88 (m, 2.12H).

$^{13}C$ NMR (151 MHz, $CDCl_3$) δ 166.63, 145.55, 140.09, 130.07, 129.29, 128.94, 128.12, 127.30, 127.04, 65.20, 31.48, 28.74, 25.76, 22.50, 13.75 (t, $J$ = 19.2 Hz).

$^{2}H$ NMR (92 MHz, $CH_2Cl_2$) δ 0.84 (s, 1D).

IR (film): ν ($cm^{-1}$) 3058, 3032, 2963, 285, 2187, 1936, 1717, 1609, 1487, 1104, 1008, 859, 746.

HRMS (ESI-TOF, m/z) calcd for $C_{19}H_{21}DNaO_2$ $(M+Na)^+$: 306.1575, found: 306.1569.

**(propane-1,3-diyl-2-D)dibenzene (4j)**

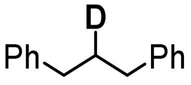


**4j**

A dried 5 mL glass vial was charged with alkyl chlorides **3j** (46.1 mg, 0.20 mmol), photocatalyst **PC4** (11.2 mg, 0.040 mmol), $(n\text{PrS})_2$ (10 μL, 0.060 mmol), $HCO_2Na$ (27.2 mg, 0.40 mmol), $D_2O$ (200 μL) and DMSO (1.0 mL) under air and then performed in a sealed vessel. The glass vial was positioned approximately 3 cm away from a 50 W blue LEDs lamp ($\lambda_{max}$ = 400 nm). After being stirred at room temperature (~ 30 °C under irradiation) for 72 h, the reaction mixture was purified by flash chromatography on silica gel (eluted with PE) to afford product **4j** (23.6 mg, 0.120 mmol, 60% yield, 90% D) as a colorless oil.

$^{1}H$ NMR (600 MHz, $CDCl_3$) δ 7.31 (dd, $J$ = 9.9, 5.2 Hz, 4H), 7.21 (dd, $J$ = 9.2, 4.0 Hz, 6H), 2.69 (t, $J$ = 7.8 Hz, 4H), 2.03 – 1.95 (m, 1.10H).

$^{13}C$ NMR (151 MHz, $CDCl_3$) δ 142.34, 128.49, 128.35, 125.78, 35.39, 32.60 (t, $J$ = 19.2 Hz).

$^{2}H$ NMR (92 MHz, $CH_2Cl_2$) δ 1.89 (s, 1D).

IR (film): ν ($cm^{-1}$) 3027, 2926, 2854, 1603, 1497, 1454, 1081, 1030, 743, 699, 666.

HRMS (ESI-TOF, m/z) calcd for $C_{15}H_{16}D$ $(M+H)^+$: 198.1388, found: 198.1367.

**4-(3-methylbutoxy-3-D)-1,1'-biphenyl (4k)**

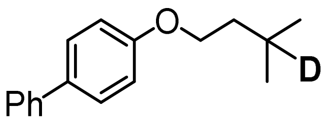

**4k**

A dried 5 mL glass vial was charged with alkyl chlorides **3k** (55.0 mg, 0.20 mmol), photocatalyst **PC4** (11.2 mg, 0.040 mmol), (*n*PrS)$_2$ (10 μL, 0.060 mmol), $HCO_2Na$ (27.2 mg, 0.40 mmol), $D_2O$ (200 μL) and DMSO (1.0 mL) under air and then performed in a sealed vessel. The glass vial was positioned approximately 3 cm away from a 50 W blue LEDs lamp ($\lambda_{max}$ = 400 nm). After being stirred at room temperature (~ 30 °C under irradiation) for 72 h, the reaction mixture was purified by flash chromatography on silica gel (eluted with PE:EtOAc = 40:1) to afford product **4k** (26.5 mg, 0.110 mmol, 55% yield, 68% D) as a colorless oil.

$^1$H NMR (500 MHz, $CDCl_3$) δ 7.62 – 7.50 (m, 4H), 7.43 (dd, $J$ = 10.6, 4.8 Hz, 2H), 7.31 (t, $J$ = 7.4 Hz, 1H), 7.03 – 6.93 (m, 2H), 4.04 (t, $J$ = 6.7 Hz, 2H), 1.88 (dt, $J$ = 13.4, 6.7 Hz, 0.32H), 1.71 (t, $J$ = 6.6 Hz, 2H), 0.99 (d, $J$ = 7.1 Hz, 6H).

$^{13}$C NMR (126 MHz, $CDCl_3$) δ 158.75, 140.93, 133.57, 128.71, 128.12, 126.73, 126.60, 114.80, 66.46, 37.95, 24.64 (t, $J$ = 19.6 Hz), 22.50.

$^2$H NMR (92 MHz, $CH_2Cl_2$) δ 1.12 (s, 1D).

IR (film): ν (cm$^{-1}$) 2952, 2932, 1606, 1522, 1465, 1268, 1250, 1054, 831, 760, 691, 604.

HRMS (ESI-TOF, m/z) calcd for $C_{17}H_{20}DO$ $(M+H)^+$: 242.1650, found: 242.1656.

**3-methylbutyl-3-D [1,1'-biphenyl]-4-carboxylate (4l)**

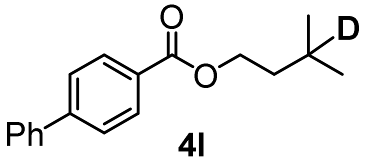

**4l**

A dried 5 mL glass vial was charged with alkyl chlorides **3l** (60.6 mg, 0.20 mmol), photocatalyst **PC4** (11.2 mg, 0.040 mmol), (*n*PrS)$_2$ (10 μL, 0.060 mmol), $HCO_2Na$ (27.2 mg, 0.40 mmol), $D_2O$ (200 μL) and DMSO (1.0 mL) under air and then performed in a sealed

vessel. The glass vial was positioned approximately 3 cm away from a 50 W blue LEDs lamp ($\lambda_{max}$ = 400 nm). After being stirred at room temperature (~ 30 °C under irradiation) for 72 h, the reaction mixture was purified by flash chromatography on silica gel (eluted with PE:EtOAc = 40:1) to afford product **4l** (23.7 mg, 0.088 mmol, 44% yield, 61% D) as a colorless oil.

$^1$H NMR (500 MHz, $CDCl_3$) δ 8.11 (d, $J$ = 8.3 Hz, 2H), 7.68 – 7.60 (m, 4H), 7.47 (t, $J$ = 7.7 Hz, 2H), 7.44 – 7.34 (m, 1H), 4.39 (t, $J$ = 6.8 Hz, 2H), 1.85 – 1.79 (m, 0.39H), 1.69 (t, $J$ = 6.7 Hz, 2H), 1.00 (d, $J$ = 6.8 Hz, 6H).

$^{13}$C NMR (126 MHz, $CDCl_3$) δ 166.60, 145.56, 140.09, 130.07, 129.29, 128.93, 128.12, 127.29, 127.04, 63.67, 37.37, 24.80 (t, $J$ = 20.4 Hz), 22.43.

$^2$H NMR (92 MHz, $CH_2Cl_2$) δ 1.81 (s, 1D).

IR (film): ν (cm$^{-1}$) 2957, 2926, 1717, 1610, 1277, 1113, 858, 748, 700.

HRMS (ESI-TOF, m/z) calcd for $C_{18}H_{20}DO_2$ $(M+H)^+$: 270.1599, found: 270.1599.

***<u>Substrate scope for benzyl, α-carbonyl or α-hetero chorides</u>***

**4-(methyl-D)-1,1'-biphenyl (4m)**

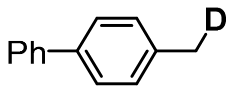

**4m**

A dried 5 mL glass vial was charged with alkyl chloride **3m** (40.5 mg, 0.20 mmol), photocatalyst **PC4** (5.6 mg, 0.020 mmol), $(n\text{PrS})_2$ (10 μL, 0.060 mmol), $HCO_2Na$ (27.2 mg, 0.40 mmol), $D_2O$ (200 μL) and DMSO (1.0 mL) under air and then performed in a sealed vessel. The glass vial was positioned approximately 3 cm away from a 50 W blue LEDs lamp ($\lambda_{max}$ = 400 nm). After being stirred at room temperature (~ 30 °C under irradiation) for 10 h, the reaction mixture was purified by flash chromatography on silica gel (eluted with PE) to afford product **4m** (27.4 mg, 0.162 mmol, 81% yield, 97% D) as a colorless oil.

$^1$H NMR (500 MHz, $CDCl_3$) δ 7.61 (d, $J$ = 7.3 Hz, 2H), 7.53 (d, $J$ = 8.0 Hz, 2H), 7.45 (t, $J$ = 7.7 Hz, 2H), 7.35 (t, $J$ = 7.4 Hz, 1H), 7.28 (d, $J$ = 7.9 Hz, 2H), 2.42 (d, $J$ = 7.9 Hz, 2.03H).

$^{13}C$ NMR (151 MHz, $CDCl_3$) δ 141.21, 138.40, 137.05, 137.02, 129.52, 128.75, 127.03, 127.01, 20.86 (t, *J* = 19.1 Hz).

$^{2}H$ NMR (92 MHz, $CH_2Cl_2$) δ 2.18 (s, 1D).

IR (film): ν ($cm^{-1}$) 3027, 2925, 2855, 1685, 1602, 1489, 1450, 1009, 755, 697.

HRMS (ESI-TOF, m/z) calcd for $C_{13}H_{11}DNa$ $(M+Na)^+$: 192.0894, found: 192.0893.

**1-(methyl-D)naphthalene (4n)**

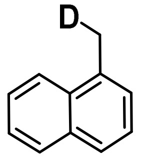

**4n**

A dried 5 mL glass vial was charged with alkyl chloride **3n** (35.3 mg, 0.20 mmol), photocatalyst **PC4** (5.6 mg, 0.020 mmol), $(n\text{PrS})_2$ (10 μL, 0.060 mmol), $HCO_2Na$ (27.2 mg, 0.40 mmol), $D_2O$ (200 μL) and DMSO (1.0 mL) under air and then performed in a sealed vessel. The glass vial was positioned approximately 3 cm away from a 50 W blue LEDs lamp ($\lambda_{max}$ = 400 nm). After being stirred at room temperature (~ 30 °C under irradiation) for 10 h, the reaction mixture was purified by flash chromatography on silica gel (eluted with PE) to afford product **4n** (19.7 mg, 0.138 mmol, 69% yield, 93% D) as a colorless oil.

$^{1}H$ NMR (600 MHz, $CDCl_3$) δ 8.03 (d, *J* = 8.3 Hz, 1H), 7.88 (d, *J* = 7.9 Hz, 1H), 7.74 (d, *J* = 8.2 Hz, 1H), 7.53 (dt, *J* = 14.7, 6.9 Hz, 2H), 7.46 – 7.30 (m, 2H), 2.72 (d, *J* = 9.6 Hz, 2.07H).

$^{13}C$ NMR (151 MHz, $CDCl_3$) δ 134.27, 133.58, 132.66, 128.56, 126.59, 126.40, 125.74, 125.61, 125.57, 124.15, 19.15 (t, *J* = 19.7 Hz).

$^{2}H$ NMR (92 MHz, $CH_2Cl_2$) δ 2.88 (s, 1D).

IR (film): ν ($cm^{-1}$) 3044, 2963, 2925, 2199, 1685, 1596, 1508, 1462, 1397, 1261, 1165, 1019, 787, 771.

HRMS (ESI-TOF, m/z) calcd for $C_{11}H_{10}D$ $(M+H)^+$: 144.0918, found: 144.0921.

**4,4'-bis(methyl-D$_2$)-1,1'-biphenyl (4o)**

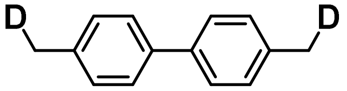

**4o**

A dried 5 mL glass vial was charged with alkyl chloride **3o** (50.2 mg, 0.20 mmol), photocatalyst **PC4** (5.6 mg, 0.020 mmol), (*n*PrS)$_2$ (10 μL, 0.060 mmol), $HCO_2Na$ (27.2 mg, 0.40 mmol), $D_2O$ (200 μL) and DMSO (1.0 mL) under air and then performed in a sealed vessel. The glass vial was positioned approximately 3 cm away from a 50 W blue LEDs lamp ($\lambda_{max}$ = 400 nm). After being stirred at room temperature (~ 30 °C under irradiation) for 10 h, the reaction mixture was purified by flash chromatography on silica gel (eluted with PE) to afford product **4o** (29.2 mg, 0.172 mmol, 86% yield, 95% D) as a white solid.

$^1$H NMR (500 MHz, $CDCl_3$) δ 7.49 (d, *J* = 8.1 Hz, 4H), 7.25 (d, *J* = 8.1 Hz, 4H), 2.41 – 2.37 (m, 4.11H).

$^{13}$C NMR (151 MHz, $CDCl_3$) δ 138.31, 136.68, 129.45, 126.83, 20.83 (t, *J* = 39.1 Hz).

$^2$H NMR (92 MHz, $CH_2Cl_2$) δ 2.42 (s, 2D).

IR (film): ν (cm$^{-1}$) 3025, 2963, 2924, 2307, 1676, 1500, 1420, 1260, 1104, 1015, 790, 741.

HRMS (ESI-TOF, m/z) calcd for $C_{14}H_{12}D_2Na$ $(M+Na)^+$: 207.1113, found: 207.1111.

**2-(methyl-D)-1-phenyl-1H-benzo[d]imidazole (4p)**

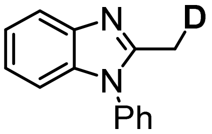

**4p**

A dried 5 mL glass vial was charged with alkyl chloride **3p** (48.5 mg, 0.20 mmol), photocatalyst **PC4** (5.6 mg, 0.020 mmol), (*n*PrS)$_2$ (10 μL, 0.060 mmol), $HCO_2Na$ (27.2 mg, 0.40 mmol), $D_2O$ (200 μL) and DMSO (1.0 mL) under air and then performed in a sealed vessel. The glass vial was positioned approximately 3 cm away from a 50 W blue LEDs lamp ($\lambda_{max}$ = 400 nm). After being stirred at room temperature (~ 30 °C under irradiation) for 10 h,

the reaction mixture was purified by flash chromatography on silica gel (eluted with PE:EtOAc = 10:1) to afford product **4p** (25.1 mg, 0.120 mmol, 60% yield, 99% D) as a colorless oil.

$^1$H NMR (600 MHz, $CDCl_3$) δ 7.73 (d, $J$ = 8.0 Hz, 1H), 7.56 (dd, $J$ = 10.4, 4.8 Hz, 2H), 7.50 (t, $J$ = 7.5 Hz, 1H), 7.34 (d, $J$ = 7.3 Hz, 2H), 7.24 (d, $J$ = 7.2 Hz, 1H), 7.17 (t, $J$ = 7.6 Hz, 1H), 7.11 (d, $J$ = 8.0 Hz, 1H), 2.49 (dd, $J$ = 6.6, 4.4 Hz, 2.01H).

$^{13}$C NMR (151 MHz, $CDCl_3$) δ 151.52, 142.42, 136.41, 136.02, 129.94, 128.84, 127.07, 122.65, 122.45, 118.94, 109.97, 14.17 (t, $J$ = 19.8 Hz).

$^2$H NMR (92 MHz, $CH_2Cl_2$) δ 2.42 (s, 1D).

IR (film): ν (cm$^{-1}$) 3058, 2925, 1597, 1500, 1457, 1391, 1330, 1188, 760, 743, 557.

HRMS (ESI-TOF, m/z) calcd for $C_{14}H_{11}DN_2Na$ $(M+Na)^+$: 232.0955, found: 232.0955.

***N*-phenylacetamide-2-D (4q)**

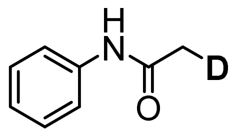


**4q**

A dried 5 mL glass vial was charged with alkyl chloride **3q** (33.9 mg, 0.20 mmol), photocatalyst **PC4** (11.2 mg, 0.040 mmol), $(n\text{PrS})_2$ (10 μL, 0.060 mmol), $HCO_2Na$ (27.2 mg, 0.40 mmol), $D_2O$ (200 μL) and DMSO (1.0 mL) under air and then performed in a sealed vessel. The glass vial was positioned approximately 3 cm away from a 50 W blue LEDs lamp ($\lambda_{max}$ = 400 nm). After being stirred at room temperature (~ 30 °C under irradiation) for 20 h, the reaction mixture was purified by flash chromatography on silica gel (eluted with PE:EtOAc = 3:1) to afford product **4q** (14.1 mg, 0.104 mmol, 52% yield, 95% D) as a grey solid.

$^1$H NMR (600 MHz, $CDCl_3$) δ 7.49 (d, $J$ = 7.9 Hz, 3H), 7.30 (t, $J$ = 7.8 Hz, 2H), 7.10 (t, $J$ = 7.4 Hz, 1H), 2.15 (dd, $J$ = 5.9, 3.9 Hz, 2.05H).

$^{13}$C NMR (151 MHz, $CDCl_3$) δ 168.58, 137.90, 129.00, 124.35, 119.97, 24.34 (t, $J$ = 19.8 Hz).

$^2$H NMR (92 MHz, $CDCl_3$) δ 2.07 (s, 1D).

IR (film): ν ($cm^{-1}$) 3250, 3194, 3136, 3082, 2924, 1658, 1596, 1554, 1500, 1325, 760, 694.

HRMS (ESI-TOF, m/z) calcd for $C_8H_9DNO$ $(M+H)^+$: 137.0820, found: 137.0822.

**ethyl 4-(acetamido-2-D)benzoate (4r)**

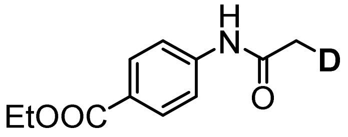


**4r**

A dried 5 mL glass vial was charged with alkyl chloride **3r** (48.3 mg, 0.20 mmol), photocatalyst **PC4** (5.6 mg, 0.020 mmol), $(n\text{PrS})_2$ (10 μL, 0.060 mmol), $HCO_2Na$ (27.2 mg, 0.40 mmol), $D_2O$ (200 μL) and DMSO (1.0 mL) under air and then performed in a sealed vessel. The glass vial was positioned approximately 3 cm away from a 50 W blue LEDs lamp ($\lambda_{max}$ = 400 nm). After being stirred at room temperature (~ 30 °C under irradiation) for 48 h, the reaction mixture was purified by flash chromatography on silica gel (eluted with PE:EtOAc = 3:1) to afford product **4r** (31.2 mg, 0.150 mmol, 75% yield, 96% D) as a grey solid.

$^1$H NMR (500 MHz, $CDCl_3$) δ 8.38 (s, 1H), 7.96 (d, *J* = 8.6 Hz, 2H), 7.61 (d, *J* = 8.5 Hz, 2H), 4.33 (q, *J* = 7.1 Hz, 2H), 2.17 (dd, *J* = 5.2, 3.3 Hz, 2.04H), 1.36 (t, *J* = 7.1 Hz, 3H).

$^{13}$C NMR (126 MHz, $CDCl_3$) δ 169.19, 166.37, 142.36, 130.69, 125.74, 118.93, 60.95, 24.38 (t, *J* = 19.5 Hz), 14.32.

$^2$H NMR (92 MHz, $CH_2Cl_2$) δ 1.98 (s, 1D).

IR (film): ν ($cm^{-1}$) 3322, 3118, 2983, 1683, 1600, 1538, 1410, 1278, 1175, 1108, 1021, 857, 771, 699.

HRMS (ESI-TOF, m/z) calcd for $C_{11}H_{12}DNNaO_3$ $(M+Na)^+$: 231.0850, found: 231.0850.

**1-(methyl-D)-1H-benzo[d][1,2,3]triazole (4s)**

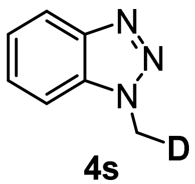

**4s**

A dried 5 mL glass vial was charged with alkyl chloride **3s** (33.5 mg, 0.20 mmol), photocatalyst **PC4** (11.2 mg, 0.040 mmol), ($n$PrS)$_2$ (10 μL, 0.060 mmol), $HCO_2Na$ (27.2 mg, 0.40 mmol), $D_2O$ (200 μL) and DMSO (1.0 mL) under air and then performed in a sealed vessel. The glass vial was positioned approximately 3 cm away from a 50 W blue LEDs lamp ($\lambda_{max}$ = 400 nm). After being stirred at room temperature (~ 30 °C under irradiation) for 10 h, the reaction mixture was purified by flash chromatography on silica gel (eluted with PE:EtOAc = 10:1) to afford product **4s** (11.5 mg, 0.086 mmol, 43% yield, 96% D) as a grey solid.

$^1$H NMR (600 MHz, $CDCl_3$) δ 8.05 (d, $J$ = 8.3 Hz, 1H), 7.60 – 7.42 (m, 2H), 7.36 (t, $J$ = 7.4 Hz, 1H), 4.28 (dd, $J$ = 6.1, 4.2 Hz, 2.04H).

$^{13}$C NMR (151 MHz, $CDCl_3$) δ 145.93, 133.50, 127.33, 123.88, 119.93, 109.14, 34.01 (t, $J$ = 21.0 Hz).

$^2$H NMR (92 MHz, $CH_2Cl_2$) δ 4.08 (s, 1D).

IR (film): ν (cm$^{-1}$) 2925, 1497, 1458, 1269, 1167, 1001, 744, 584.

HRMS (ESI-TOF, m/z) calcd for $C_7H_7DN_3$ (M+H)$^+$: 135.0776, found: 135.0776.

**4-(methyl-D)-1,1'-biphenyl (4m)**

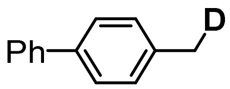

**4m**

X = F

A dried 5 mL glass vial was charged with 4-(fluoromethyl)-1,1'-biphenyl (33.5 mg, 0.20 mmol), photocatalyst **PC4** (5.6 mg, 0.020 mmol), ($n$PrS)$_2$ (10 μL, 0.060 mmol), $HCO_2Na$ (27.2 mg, 0.40 mmol), $D_2O$ (200 μL) and DMSO (1.0 mL) under air and then performed in a sealed vessel. The glass vial was positioned approximately 3 cm away from a 50 W blue

LEDs lamp ($\lambda_{max}$ = 400 nm). After being stirred at room temperature (~ 30 °C under irradiation) for 48 h, the reaction mixture was purified by flash chromatography on silica gel (eluted with PE) to afford product **4m** (26.3 mg, 0.156 mmol, 78% yield, 90% D) as a grey solid.

### 3.5 Site-Selective Deuterodechlorination of Polychlorides

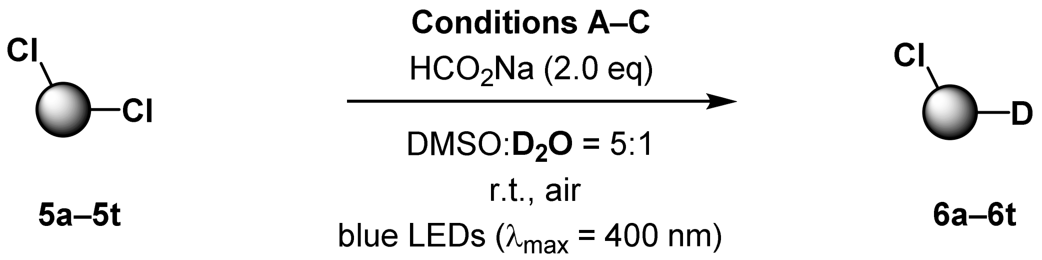


**Conditions A: PC1** (10 mol%), $(MeS)_2$ (30 mol%). **Conditions B: PC2** (10 mol%), $(n\text{PrS})_2$ (30 mol%). **Conditions C: PC4** (20 mol%), $(n\text{PrS})_2$ (30 mol%).

**2-chloropyridine-6-D (6a)**

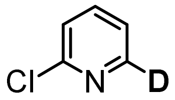


**6a**

**Condition B:** a dried 5 mL glass vial was charged with **5a** (29.6 mg, 0.20 mmol), photocatalyst **PC2** (16.2 mg, 0.020 mmol), $(n\text{PrS})_2$ (10 μL, 0.060 mmol), $HCO_2Na$ (27.2 mg, 0.40 mmol), $D_2O$ (200 μL) and DMSO (1.0 mL) under air and then performed in a sealed vessel. The glass vial was positioned approximately 3 cm away from a 50 W blue LEDs lamp ($\lambda_{max}$ = 400 nm). After being stirred at room temperature (~ 30 °C under irradiation) for 48 h, the reaction mixture was purified by flash chromatography on silica gel (eluted with PE:EtOAc = 20:1) to afford product **6a** (14.0 mg, 0.122 mmol, 61% yield, 98% D) as a yellow oil.

$^1$H NMR (500 MHz, $CDCl_3$) δ 8.39 (d, $J$ = 3.4 Hz, 0.02H), 7.65 (t, $J$ = 7.7 Hz, 1H), 7.33 (dd, $J$ = 8.0, 0.8 Hz, 1H), 7.22 (d, $J$ = 7.4 Hz, 1H).

$^{13}$C NMR (151 MHz, $CDCl_3$) δ 151.61, 149.50 (t, $J$ = 27.5 Hz), 138.73, 124.50, 122.11.

$^2$H NMR (92 MHz, $CH_2Cl_2$) δ 8.31 (s, 1D).

IR (film): ν (cm$^{-1}$) 3069, 2926, 2262, 1569, 1544, 1415, 1172, 1082, 1068, 989.

HRMS (ESI-TOF, m/z) calcd for $C_5H_4DClN$ $(M+H)^+$: 115.0168, found: 115.0165.

**Condition A:** a dried 5 mL glass vial was charged with **5a** (29.6 mg, 0.20 mmol), photocatalyst **PC1** (8.7 mg, 0.020 mmol), $(MeS)_2$ (6 μL, 0.060 mmol), $HCO_2Na$ (27.2 mg, 0.40 mmol), $D_2O$ (200 μL) and DMSO (1.0 mL) under air and then performed in a sealed vessel. The glass vial was positioned approximately 3 cm away from a 50 W blue LEDs lamp ($\lambda_{max}$ = 400 nm). After being stirred at room temperature (~ 30 °C under irradiation) for 52 h, the reaction mixture was purified by flash chromatography on silica gel (eluted with PE:EtOAc = 20:1) to afford product **6a** (12.4 mg, 0.108 mmol, 54% yield, 82% D) as a yellow oil.

**3-chloropyridine-6-D (6b)**

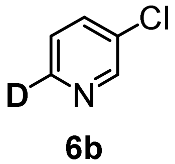

**6b**

**Condition B:** a dried 5 mL glass vial was charged with **5b** (29.6 mg, 0.20 mmol), photocatalyst **PC2** (16.2 mg, 0.020 mmol), $(n\text{PrS})_2$ (10 μL, 0.060 mmol), $HCO_2Na$ (27.2 mg, 0.40 mmol), $D_2O$ (200 μL) and DMSO (1.0 mL) under air and then performed in a sealed vessel. The glass vial was positioned approximately 3 cm away from a 50 W blue LEDs lamp ($\lambda_{max}$ = 400 nm). After being stirred at room temperature (~ 30 °C under irradiation) for 40 h, the reaction mixture was purified by flash chromatography on silica gel (eluted with PE:EtOAc = 20:1) to afford product **6b** (14.5 mg, 0.126 mmol, 63% yield, 90% D) as a yellow oil.

$^{1}H$ NMR (600 MHz, $CDCl_3$) δ 8.55 (d, *J* = 2.2 Hz, 1H), 8.45 (d, *J* = 3.6 Hz, 0.1H), 7.63 (dd, *J* = 8.2, 2.4 Hz, 1H), 7.21 (d, *J* = 8.2 Hz, 1H).

$^{13}C$ NMR (151 MHz, $CDCl_3$) δ 148.88, 147.19 (t, *J* = 27.8 Hz), 135.81, 132.17, 124.18.

$^{2}H$ NMR (92 MHz, $CH_2Cl_2$) δ 8.43 (s, 1D).

IR (film): ν ($cm^{-1}$) 3047, 2926, 2261, 1560, 1450, 1360, 1286, 1148, 1108, 857, 724, 629.

HRMS (ESI-TOF, m/z) calcd for $C_5H_4DClN$ $(M+H)^+$: 115.0168, found: 115.0168.

**2-chloro-4-phenylpyridine-6-D (6c)**

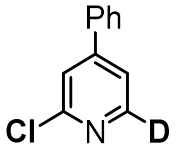

**6c**

**Condition A:** a dried 5 mL glass vial was charged with **5c** (44.8 mg, 0.20 mmol), photocatalyst **PC1** (8.7 mg, 0.020 mmol), $(MeS)_2$ (6 μL, 0.060 mmol), $HCO_2Na$ (27.2 mg, 0.40 mmol), $D_2O$ (200 μL) and DMSO (1.0 mL) under air and then performed in a sealed vessel. The glass vial was positioned approximately 3 cm away from a 50 W blue LEDs lamp ($\lambda_{max}$ = 400 nm). After being stirred at room temperature (~ 30 °C under irradiation) for 15 h, the reaction mixture was purified by flash chromatography on silica gel (eluted with PE:EtOAc = 10:1) to afford product **6c** (28.9 mg, 0.152 mmol, 76% yield, 93% D) as a pale yellow oil.

$^{1}H$ NMR (600 MHz, $CDCl_3$) δ 8.42 (d, *J* = 5.2 Hz, 0.07H), 7.60 (d, *J* = 6.8 Hz, 2H), 7.57 – 7.35 (m, 5H).

$^{13}C$ NMR (151 MHz, $CDCl_3$) δ 152.22, 151.60, 149.69 (t, *J* = 27.8 Hz), 136.83, 129.71, 129.29, 127.06, 122.08, 120.38.

$^{2}H$ NMR (92 MHz, $CH_2Cl_2$) δ 8.38 (s, 1D).

IR (film): ν ($cm^{-1}$) 3058, 1605, 1585, 1368, 11800, 1086, 988, 867.

HRMS (ESI-TOF, m/z) calcd for $C_{11}H_8DClN$ $(M+H)^+$: 191.0481, found: 191.0483.

**(2-chloropyridin-4-yl-6-D)(piperidin-1-yl) methanone (6d)**

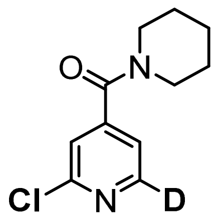

**6d**

**Condition A:** a dried 5 mL glass vial was charged with **5d** (51.8 mg, 0.20 mmol), photocatalyst **PC1** (8.7 mg, 0.020 mmol), $(MeS)_2$ (6 μL, 0.060 mmol), $HCO_2Na$ (27.2 mg, 0.40 mmol), $D_2O$ (200 μL) and DMSO (1.0 mL) under air and then performed in a sealed vessel. The glass vial was positioned approximately 3 cm away from a 50 W blue LEDs lamp ($\lambda_{max}$ = 400 nm). After being stirred at room temperature (~ 30 °C under irradiation) for 18 h, the reaction mixture was purified by flash chromatography on silica gel (eluted with PE:EtOAc = 3:1) to afford product **6d** (41.6 mg, 0.184 mmol, 92% yield, 90% D) as a pale yellow oil.

$^1$H NMR (600 MHz, $CDCl_3$) δ 8.40 (d, *J* = 4.9 Hz, 0.1H), 7.27 (d, *J* = 1.3 Hz, 1H), 7.16 (s, 1H), 3.75 – 3.57 (m, 2H), 3.32 – 3.12 (m, 2H), 1.74 – 1.60 (m, 4H), 1.56 – 1.44 (m, 2H).

$^{13}$C NMR (151 MHz, $CDCl_3$) δ 166.06, 152.00, 149.84 (t, *J* = 28.2 Hz), 147.12, 121.82, 119.67, 48.49, 43.08, 26.47, 25.42, 24.30.

$^2$H NMR (92 MHz, $CH_2Cl_2$) δ 8.30 (s, 1D).

IR (film): ν ($cm^{-1}$) 3061, 2995, 2940, 2858, 2262, 1635, 1586, 1532, 1447, 1351, 1274, 1182, 1120, 1013, 873, 780, 745.

HRMS (ESI-TOF, m/z) calcd for $C_{11}H_{13}DClN_2O$ $(M+H)^+$: 226.0852, found: 226.0853.

**methyl 5-chloronicotinate-2-D (6e)**

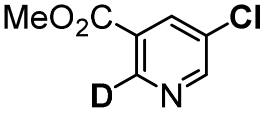

**6e**

**Condition B:** a dried 5 mL glass vial was charged with **5e** (41.2 mg, 0.20 mmol), photocatalyst **PC2** (16.2 mg, 0.020 mmol), (*n*PrS)$_2$ (10 μL, 0.060 mmol), $HCO_2Na$ (27.2 mg, 0.40 mmol), $D_2O$ (200 μL) and DMSO (1.0 mL) under air and then performed in a sealed vessel. The glass vial was positioned approximately 3 cm away from a 50 W blue LEDs lamp ($\lambda_{max}$ = 400 nm). After being stirred at room temperature (~ 30 °C under irradiation) for 16 h, the reaction mixture was purified by flash chromatography on silica gel (eluted with PE:EtOAc = 10:1) to afford product **6e** (18.3 mg, 0.130 mmol, 53% yield, 90% D) as a white solid.

$^1$H NMR (500 MHz, $CDCl_3$) δ 9.06 (d, *J* = 1.6 Hz, 0.1H), 8.71 (d, *J* = 2.4 Hz, 1H), 8.25 (d, *J* = 2.4 Hz, 1H), 3.95 (s, 3H).

$^{13}$C NMR (151 MHz, $CDCl_3$) δ 164.58, 152.38, 148.15 (t, *J* = 28.1 Hz), 136.67, 132.18, 126.92, 52.75.

$^2$H NMR (92 MHz, $CH_2Cl_2$) δ 9.18 (s, 1D).

IR (film): ν ($cm^{-1}$) 3051, 2026, 2853, 2232, 1729, 1620, 1568, 1489, 1397, 1218, 1141, 979, 904, 767.

HRMS (ESI-TOF, m/z) calcd for $C_7H_6DClNO_2$ $(M+H)^+$: 173.0223, found: 173.0221.

**methyl 4-chloropicolinate-6-D (6f)**

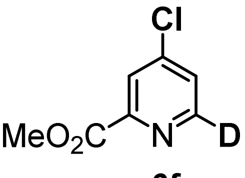

**6f**

**Condition B:** a dried 5 mL glass vial was charged with **5f** (41.2 mg, 0.20 mmol), photocatalyst **PC2** (16.2 mg, 0.020 mmol), (*n*PrS)$_2$ (10 μL, 0.060 mmol), $HCO_2Na$ (27.2 mg,

0.40 mmol), $D_2O$ (200 μL) and DMSO (1.0 mL) under air and then performed in a sealed vessel. The glass vial was positioned approximately 3 cm away from a 50 W blue LEDs lamp ($\lambda_{max}$ = 400 nm). After being stirred at room temperature (~ 30 °C under irradiation) for 12 h, the reaction mixture was purified by flash chromatography on silica gel (eluted with PE:EtOAc = 10:1) to afford product **6f** (19.7 mg, 0.114 mmol, 57% yield, 90% D) as a white solid.

$^1$H NMR (600 MHz, $CDCl_3$) δ 8.05 (s, 1H), 7.81 (t, *J* = 7.8 Hz, 0.1H), 7.51 (s, 1H), 3.98 (s, 3H).

$^{13}$C NMR (151 MHz, $CDCl_3$) δ 164.53, 151.61, 148.24, 139.34 (t, *J* = 25.5 Hz), 128.00, 123.62, 53.17.

$^2$H NMR (92 MHz, $CH_2Cl_2$) δ 8.31 (s, 1D).

IR (film): ν ($cm^{-1}$) 3022, 2963, 2296, 1720, 1571, 1551, 1441, 1291, 1250, 1128, 1070, 988, 907, 740, 691.

HRMS (ESI-TOF, m/z) calcd for $C_7H_6DClNO_2$ $(M+H)^+$: 173.0223, found: 173.0223.

**ethyl 5-chloronicotinate-6-D (6g)**

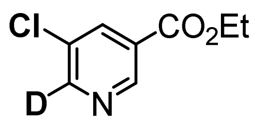


**6g**

**Condition B:** a dried 5 mL glass vial was charged with **5g** (44.0 mg, 0.20 mmol), photocatalyst **PC2** (16.2 mg, 0.020 mmol), $(n\text{PrS})_2$ (10 μL, 0.060 mmol), $HCO_2Na$ (27.2 mg, 0.40 mmol), $D_2O$ (200 μL) and DMSO (1.0 mL) under air and then performed in a sealed vessel. The glass vial was positioned approximately 3 cm away from a 50 W blue LEDs lamp ($\lambda_{max}$ = 400 nm). After being stirred at room temperature (~ 30 °C under irradiation) for 12 h, the reaction mixture was purified by flash chromatography on silica gel (eluted with PE:EtOAc = 10:1) to afford product **6g** (18.3 mg, 0.098 mmol, 49% yield, 96% D) as a gray solid.

$^1$H NMR (500 MHz, $CDCl_3$) δ 9.08 (d, *J* = 1.2 Hz, 1H), 8.72 (d, *J* = 2.2 Hz, 0.04H), 8.26 (d, *J* = 1.4 Hz, 1H), 4.42 (q, *J* = 7.1 Hz, 2H), 1.41 (t, *J* = 7.1 Hz, 3H).

$^{13}C$ NMR (151 MHz, $CDCl_3$) δ 164.11, 151.92 (t, $J$ = 28.1 Hz), 148.49, 136.65, 132.02, 127.36, 61.92, 14.23.

$^{2}H$ NMR (92 MHz, $CH_2Cl_2$) δ 8.60 (s, 1D).

IR (film): ν ($cm^{-1}$) 3068, 2983, 2937, 1728, 1581, 1430, 1367, 1290, 1216, 1120, 1021, 894, 760, 640.

HRMS (ESI-TOF, m/z) calcd for $C_8H_8DClNO_2$ $(M+H)^+$: 187.0379, found: 187.0378.

**5-chloronicotinamide-2-D (6h)**

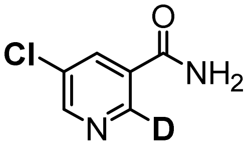

**6h**

**Condition B:** a dried 5 mL glass vial was charged with **5h** (38.2 mg, 0.20 mmol), photocatalyst **PC2** (16.2 mg, 0.020 mmol), $(n\text{PrS})_2$ (10 μL, 0.060 mmol), $HCO_2Na$ (27.2 mg, 0.40 mmol), $D_2O$ (200 μL) and DMSO (1.0 mL) under air and then performed in a sealed vessel. The glass vial was positioned approximately 3 cm away from a 50 W blue LEDs lamp ($\lambda_{max}$ = 400 nm). After being stirred at room temperature (~ 30 °C under irradiation) for 15 h, the reaction mixture was purified by flash chromatography on silica gel (eluted with PE:EtOAc = 1:1) to afford product **6h** (22.7 mg, 0.144 mmol, 72% yield, 91% D) as a white solid.

$^{1}H$ NMR (600 MHz, $(CD_3)_2SO$) δ 8.95 (d, $J$ = 1.7 Hz, 0.09H), 8.75 (d, $J$ = 2.3 Hz, 1H), 8.28 (d, $J$ = 2.3 Hz, 1H), 8.24 (s, 1H), 7.75 (s, 1H).

$^{13}C$ NMR (151 MHz, $(CD_3)_2SO$) δ 165.19, 150.57, 146.81 (t, $J$ = 27.7 Hz), 134.93, 131.19, 131.01.

$^{2}H$ NMR (92 MHz, DMSO) δ 9.61 (s, 1D).

IR (film): ν ($cm^{-1}$) 3410, 3041, 2917, 2140, 1653, 1436, 1405, 1316, 1023, 953, 707, 634.

HRMS (ESI-TOF, m/z) calcd for $C_6H_5DClN_2O$ $(M+H)^+$: 158.0226, found: 158.0225.

**8-chloroquinoline-2-D (6i)**

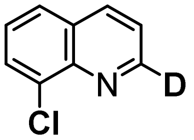

**6i**

**Condition B:** a dried 5 mL glass vial was charged with **5i** (39.6 mg, 0.20 mmol), photocatalyst **PC2** (16.2 mg, 0.020 mmol), (*n*PrS)$_2$ (10 μL, 0.060 mmol), $HCO_2Na$ (27.2 mg, 0.40 mmol), $D_2O$ (200 μL) and DMSO (1.0 mL) under air and then performed in a sealed vessel. The glass vial was positioned approximately 3 cm away from a 50 W blue LEDs lamp ($\lambda_{max}$ = 400 nm). After being stirred at room temperature (~ 30 °C under irradiation) for 10 h, the reaction mixture was purified by flash chromatography on silica gel (eluted with PE:EtOAc = 10:1) to afford product **6i** (17.1 mg, 0.104 mmol, 52% yield, 90% D) as a pale yellow oil.

$^{1}$H NMR (500 MHz, $CDCl_3$) δ 9.02 (s, 0.1H), 8.15 (dd, *J* = 8.2, 4.9 Hz, 1H), 7.93 – 7.76 (m, 1H), 7.72 (dd, *J* = 8.0, 4.5 Hz, 1H), 7.56 – 7.36 (m, 2H).

$^{13}$C NMR (151 MHz, $CDCl_3$) δ 150.67 (t, *J* = 27.4 Hz), 144.45, 136.58, 133.45, 129.63, 127.01, 126.52, 121.94, 121.82.

$^{2}$H NMR (92 MHz, $CH_2Cl_2$) δ 9.58 (s, 1D).

IR (film): ν (cm$^{-1}$) 3060, 2252, 1610, 1591, 1551, 1488, 1438, 1301, 1211, 1099, 979, 855, 761.

HRMS (ESI-TOF, m/z) calcd for $C_9H_6DClN$ $(M+H)^+$: 165.0324, found: 165.0324.

**6-chloroisoquinoline-1-D (6j)**

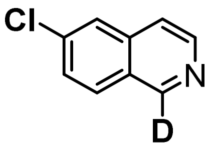

**6j**

**Condition B:** a dried 5 mL glass vial was charged with **5j** (39.6 mg, 0.20 mmol), photocatalyst **PC2** (16.2 mg, 0.020 mmol), (*n*PrS)$_2$ (10 μL, 0.060 mmol), $HCO_2Na$ (27.2 mg,

0.40 mmol), $D_2O$ (200 μL) and DMSO (1.0 mL) under air and then performed in a sealed vessel. The glass vial was positioned approximately 3 cm away from a 50 W blue LEDs lamp ($\lambda_{max}$ = 400 nm). After being stirred at room temperature (~ 30 °C under irradiation) for 20 h, the reaction mixture was purified by flash chromatography on silica gel (eluted with PE:EtOAc = 10:1) to afford product **6j** (14.5 mg, 0.088 mmol, 44% yield, 97% D) as a white solid.

$^1$H NMR (600 MHz, $CDCl_3$) δ 9.22 (s, 0.03H), 8.54 (d, $J$ = 5.6 Hz, 1H), 7.91 (d, $J$ = 8.7 Hz, 1H), 7.80 (s, 1H), 7.55 (dd, $J$ = 14.8, 7.2 Hz, 2H).

$^{13}$C NMR (151 MHz, $CDCl_3$) δ 151.92 (t, $J$ = 27.8 Hz), 144.02, 136.61, 136.46, 129.29, 128.43, 126.73, 125.39, 119.59.

$^2$H NMR (92 MHz, $CH_2Cl_2$) δ 9.69 (s, 1D).

IR (film): ν ($cm^{-1}$) 3055, 2923, 2244, 1623, 1560, 1450, 1320, 1213, 1162, 1072, 908, 880, 822, 636.

HRMS (ESI-TOF, m/z) calcd for $C_9H_6DClN(M+H)^+$: 165.0324, found: 165.0324.

**3-chloroisoquinoline-1-D (6k)**

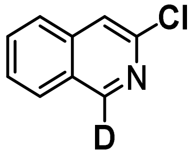


**6k**

**Condition B:** a dried 5 mL glass vial was charged with **5k** (39.6 mg, 0.20 mmol), photocatalyst photocatalyst **PC2** (16.2 mg, 0.020 mmol), ($n$PrS)$_2$ (10 μL, 0.060 mmol), $HCO_2Na$ (27.2 mg, 0.40 mmol), $D_2O$ (200 μL) and DMSO (1.0 mL) under air and then performed in a sealed vessel. The glass vial was positioned approximately 3 cm away from a 50 W blue LEDs lamp ($\lambda_{max}$ = 400 nm). After being stirred at room temperature (~ 30 °C under irradiation) for 15 h, the reaction mixture was extracted with EtOAc. The combined organic layer was dried over anhydrous $Na_2SO_4$ and concentrated under reduced pressure. The residue was subjected to silica gel chromatography (eluted with PE:EtOAc = 20:1) to afford product **6k** (21.4 mg, 0.130 mmol, 65% yield, 91% D) as a white solid.

$^{1}$H NMR (500 MHz, $CDCl_3$) δ 9.07 (s, 0.09H), 8.01 – 7.93 (m, 1H), 7.79 – 7.68 (m, 3H), 7.60 (ddd, *J* = 8.0, 6.8, 1.1 Hz, 1H).

$^{13}$C NMR (151 MHz, $CDCl_3$) δ 152.15 (t, *J* = 27.7 Hz), 145.63, 137.71, 131.30, 127.54, 127.49, 127.24, 125.73, 119.67.

$^{2}$H NMR (92 MHz, $CH_2Cl_2$) δ 9.07 (s, 1D).

IR (film): ν (cm$^{-1}$) 3059, 2926, 2850, 2244, 1624, 1577, 1560, 1487, 1429, 1375, 1294, 1137, 1088, 1000, 854.

HRMS (ESI-TOF, m/z) calcd for $C_9H_6DClN$ $(M+H)^+$: 165.0324, found: 165.0322.

**4-chloroisoquinoline-1-D (6l)**

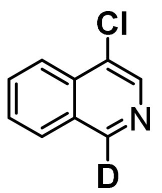

**6l**

**Condition B:** a dried 5 mL glass vial was charged with **5l** (39.6 mg, 0.20 mmol), photocatalyst **PC2** (16.2 mg, 0.020 mmol), $(n\mathrm{PrS})_2$ (10 μL, 0.060 mmol), $HCO_2Na$ (27.2 mg, 0.40 mmol), $D_2O$ (200 μL) and DMSO (1.0 mL) under air and then performed in a sealed vessel. The glass vial was positioned approximately 3 cm away from a 50 W blue LEDs lamp ($\lambda_{max}$ = 400 nm). After being stirred at room temperature (~ 30 °C under irradiation) for 20 h, the reaction mixture was purified by flash chromatography on silica gel (eluted with PE:EtOAc = 10:1) to afford product **6l** (16.8 mg, 0.102 mmol, 51% yield, 92% D) as a pale yellow oil.

$^{1}$H NMR (500 MHz, $CDCl_3$) δ 9.13 (s, 0.08H), 8.57 (s, 1H), 8.18 (d, *J* = 8.5 Hz, 1H), 7.98 (d, *J* = 8.2 Hz, 1H), 7.81 (t, *J* = 7.7 Hz, 1H), 7.67 (t, *J* = 7.5 Hz, 1H).

$^{13}$C NMR (151 MHz, $CDCl_3$) δ 150.68 (t, *J* = 27.7 Hz), 141.72, 133.55, 131.50, 129.28, 128.49, 128.22, 127.73, 123.31.

$^{2}$H NMR (92 MHz, $CH_2Cl_2$) δ 9.22 (s, 1D).

IR (film): ν ($cm^{-1}$) 3055, 3022, 1723, 1573, 1445, 1418, 1282, 1246, 1192, 1126, 1108, 924, 741.

HRMS (ESI-TOF, m/z) calcd for $C_9H_6DClN$ $(M+H)^+$: 165.0324, found: 165.0324.

**5-(4-chlorophenyl)thieno[2,3-d]pyrimidine-4-D (6m)**

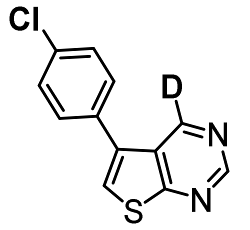

**6m**

**Condition B:** a dried 5 mL glass vial was charged with **5m** (56.2 mg, 0.20 mmol), photocatalyst **PC2** (16.2 mg, 0.020 mmol), (*n*PrS)$_2$ (10 μL, 0.060 mmol), $HCO_2Na$ (27.2 mg, 0.40 mmol), $D_2O$ (200 μL) and DMSO (1.0 mL) under air and then performed in a sealed vessel. The glass vial was positioned approximately 3 cm away from a 50 W blue LEDs lamp ($\lambda_{max}$ = 400 nm). After being stirred at room temperature (~ 30 °C under irradiation) for 15 h, the reaction mixture was purified by flash chromatography on silica gel (eluted with PE:EtOAc = 10:1) to afford product **6m** (26.8 mg, 0.108 mmol, 54% yield, 96% D) as a white solid.

$^1$H NMR (500 MHz, $CDCl_3$) δ 9.21 (s, 0.04H), 9.14 (s, 1H), 7.56 – 7.46 (m, 5H).

$^{13}$C NMR (151 MHz, $CDCl_3$) δ 169.45, 153.67, 151.01 (t, *J* = 28.3 Hz), 134.63, 133.90, 132.29, 129.50, 129.47, 128.91, 123.97.

$^2$H NMR (92 MHz, $CH_2Cl_2$) δ 9.33 (s, 1D).

IR (film): ν ($cm^{-1}$) 3081, 2923, 2854, 2335, 1958, 1617, 1505, 1401, 1364, 1202, 1091, 1015, 806, 739.

HRMS (ESI-TOF, m/z) calcd for $C_{12}H_7DClN_2S$ $(M+H)^+$: 248.0154, found: 248.0154.

**4-chlorobenzyl 5-chloronicotinate-6-D (6n)**

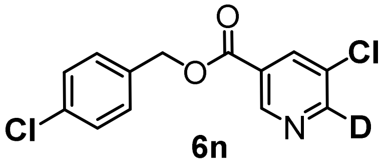

6n

**Condition A:** a dried 5 mL glass vial was charged with **5n** (63.3 mg, 0.20 mmol), photocatalyst **PC1** (8.7 mg, 0.020 mmol), $(MeS)_2$ (6 μL, 0.060 mmol), $HCO_2Na$ (27.2 mg, 0.40 mmol), $D_2O$ (200 μL) and DMSO (1.0 mL) under air and then performed in a sealed vessel. The glass vial was positioned approximately 3 cm away from a 50 W blue LEDs lamp ($\lambda_{max}$ = 400 nm). After being stirred at room temperature (~ 30 °C under irradiation) for 28 h, the reaction mixture was purified by flash chromatography on silica gel (eluted with PE:EtOAc = 10:1) to afford product **6n** (39.0 mg, 0.138 mmol, 69% yield, 78% D) as a white solid.

$^1$H NMR (500 MHz, $CDCl_3$) δ 9.10 (s, 1H), 8.74 (d, $J$ = 2.1 Hz, 0.22H), 8.26 (s, 1H), 7.38 (s, 4H), 5.35 (s, 2H).

$^{13}$C NMR (151 MHz, $CDCl_3$) δ 163.91, 152.26 (t, $J$ = 27.9 Hz), 148.60, 136.72, 134.70, 133.61, 132.12, 129.91, 128.99, 126.88, 66.74.

$^2$H NMR (92 MHz, $CH_2Cl_2$) δ 8.94 (s, 1D).

IR (film): ν (cm$^{-1}$) 3070, 3026, 2939, 2261, 1897, 1717, 1580, 1494, 1431, 1376, 1297, 1242, 1131, 997, 898, 804, 753, 642.

HRMS (ESI-TOF, m/z) calcd for $C_{13}H_9DCl_2NO_2$ $(M+H)^+$: 283.0146, found: 283.0147.

**4-chlorophenyl benzoate-4-D (6o)**

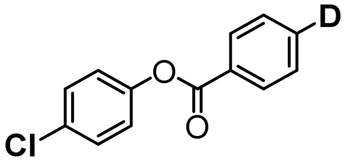

6o

**Condition B:** a dried 5 mL glass vial was charged with **5o** (53.4 mg, 0.20 mmol), photocatalyst **PC2** (16.2 mg, 0.020 mmol), $(nPrS)_2$ (10 μL, 0.060 mmol), $HCO_2Na$ (27.2 mg, 0.40 mmol), $D_2O$ (200 μL) and DMSO (1.0 mL) under air and then performed in a sealed

vessel. The glass vial was positioned approximately 3 cm away from a 50 W blue LEDs lamp ($\lambda_{max}$ = 400 nm). After being stirred at room temperature (~ 30 °C under irradiation) for 8 h, the reaction mixture was purified by flash chromatography on silica gel (eluted with PE:EtOAc = 20:1) to afford product **6o** (31.3 mg, 0.134 mmol, 67% yield, 90% D) as a grey solid.

$^1$H NMR (500 MHz, $CDCl_3$) δ 8.20 (d, *J* = 8.2 Hz, 2H), 7.64 (d, *J* = 7.5 Hz, 0.1H), 7.52 (d, *J* = 8.1 Hz, 2H), 7.40 (d, *J* = 8.8 Hz, 2H), 7.18 (t, *J* = 5.9 Hz, 2H).

$^{13}$C NMR (151 MHz, $CDCl_3$) δ 164.99, 149.44, 133.52 (t, *J* = 24.7 Hz), 131.30, 130.23, 129.57, 129.21, 128.55, 123.14.

$^2$H NMR (92 MHz, $CH_2Cl_2$) δ 7.63 (s, 1D).

IR (film): ν (cm$^{-1}$) 3089, 3062, 2928, 1904, 1734, 1586, 1488, 1409, 1263, 1200, 1092, 1014, 877, 824.

HRMS (ESI-TOF, m/z) calcd for $C_{13}H_9DClO_2(M+H)^+$: 234.0427, found: 234.0427.

**6-chlorohexyl nicotinate-6-D (6p)**

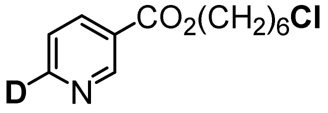


**6p**

**Condition B:** a dried 5 mL glass vial was charged with **5p** (55.2 mg, 0.20 mmol), photocatalyst **PC2** (16.2 mg, 0.020 mmol), ($n$PrS)$_2$ (10 μL, 0.060 mmol), $HCO_2Na$ (27.2 mg, 0.40 mmol), $D_2O$ (200 μL) and DMSO (1.0 mL) under air and then performed in a sealed vessel. The glass vial was positioned approximately 3 cm away from a 50 W blue LEDs lamp ($\lambda_{max}$ = 400 nm). After being stirred at room temperature (~ 30 °C under irradiation) for 36 h, the reaction mixture was purified by flash chromatography on silica gel (eluted with PE:EtOAc = 3:1) to afford product **6p** (38.3 mg, 0.158 mmol, 79% yield, 93% D) as a pale yellow oil.

$^1$H NMR (600 MHz, $CDCl_3$) δ 9.19 (d, *J* = 1.2 Hz, 1H), 8.75 – 8.74 (m, 0.07H), 8.28 (dd, *J* = 7.9, 2.1 Hz, 1H), 7.38 (d, *J* = 7.9 Hz, 1H), 4.34 (t, *J* = 6.6 Hz, 2H), 3.52 (t, *J* = 6.6 Hz, 2H), 1.82 – 1.74 (m, 4H), 1.53 – 1.42 (m, 4H).

$^{13}C$ NMR (151 MHz, $CDCl_3$) δ 165.25, 152.90 (t, $J$ = 27.2 Hz), 150.76, 137.14, 126.33, 123.21, 65.32, 44.91, 32.41, 28.50, 26.51, 25.34.

$^{2}H$ NMR (92 MHz, $CH_2Cl_2$) δ 8.74 (s, 1D).

IR (film): ν ($cm^{-1}$) 2938, 2861, 2259, 1724, 1589, 1461, 1378, 1278, 1121, 1024, 735, 636.

HRMS (ESI-TOF, m/z) calcd for $C_{12}H_{16}DClNO_2$ $(M+H)^+$: 243.1005, found: 243.1005.

**ethyl 3-chloro-4-(2-chloroacetamido-2-D) benzoate (6q)**

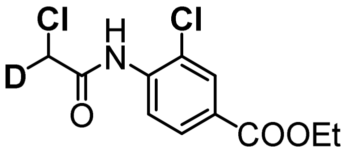


**6q**

**Condition B:** a dried 5 mL glass vial was charged with **5q** (62.1 mg, 0.20 mmol), photocatalyst **PC2** (16.2 mg, 0.020 mmol), $(n\text{PrS})_2$ (10 μL, 0.060 mmol), $HCO_2Na$ (27.2 mg, 0.40 mmol), $D_2O$ (200 μL) and DMSO (1.0 mL) under air and then performed in a sealed vessel. The glass vial was positioned approximately 3 cm away from a 50 W blue LEDs lamp ($\lambda_{max}$ = 400 nm). After being stirred at room temperature (~ 30 °C under irradiation) for 5 h, the reaction mixture was purified by flash chromatography on silica gel (eluted with PE:EtOAc = 4:1) to afford product **6q** (22.8 mg, 0.082 mmol, 41% yield, 94% D) as a white solid.

$^{1}H$ NMR (600 MHz, $CDCl_3$) δ 9.11 (s, 1H), 8.55 – 8.47 (m, 1H), 8.09 (d, $J$ = 1.7 Hz, 1H), 7.97 (dd, $J$ = 8.6, 1.6 Hz, 1H), 4.37 (q, $J$ = 7.1 Hz, 2H), 4.24 (d, $J$ = 7.9 Hz, 1.06H), 1.39 (t, $J$ = 7.1 Hz, 3H).

$^{13}C$ NMR (151 MHz, $CDCl_3$) δ 164.92, 164.13, 137.38, 130.44, 129.36, 127.35, 122.94, 120.06, 61.39, 42.92 (t, $J$ = 23.8 Hz), 14.31.

$^{2}H$ NMR (92 MHz, $CH_2Cl_2$) δ 4.57 (s, 1D).

IR (film): ν ($cm^{-1}$) 3369, 2984, 1703, 1660, 1522, 1479, 1391, 1272, 1150, 1051, 907, 769.

HRMS (ESI-TOF, m/z) calcd for $C_{11}H_{11}DCl_2NO_3$ $(M+H)^+$: 277.0252, found: 277.0252.

**N-(4-chlorophenyl)acetamide-2-D (6r)**

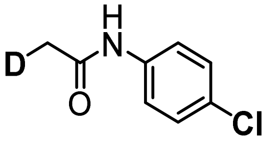


**6r**

**Condition C:** a dried 5 mL glass vial was charged with **5r** (40.8 mg, 0.20 mmol), photocatalyst **PC4** (11.2 mg, 0.040 mmol), (*n*PrS)$_2$ (10 μL, 0.060 mmol), $HCO_2Na$ (27.2 mg, 0.40 mmol), $D_2O$ (200 μL) and DMSO (1.0 mL) under air and then performed in a sealed vessel. The glass vial was positioned approximately 3 cm away from a 50 W blue LEDs lamp ($\lambda_{max}$ = 400 nm). After being stirred at room temperature (~ 30 °C under irradiation) for 15 h, the reaction mixture was purified by flash chromatography on silica gel (eluted with PE:EtOAc = 5:1) to afford product **6r** (11.9 mg, 0.078 mmol, 39% yield, 96% D) as a white solid.

$^1$H NMR (600 MHz, $(CD_3)_2CO$) δ 9.31 (s, 1H), 7.66 (d, *J* = 8.7 Hz, 2H), 7.29 (d, *J* = 8.8 Hz, 2H), 2.07 (s, 2.04H).

$^{13}$C NMR (151 MHz, $(CD_3)_2CO$) δ 168.20, 138.52, 128.51, 127.29, 120.47, 23.10 (t, *J* = 19.9 Hz).

$^2$H NMR (92 MHz, Acetone) δ 2.88 (s, 1D).

IR (film): ν (cm$^{-1}$) 3285, 1656, 1604, 1551, 1489, 1392, 1093, 828, 666.

HRMS (ESI-TOF, m/z) calcd for $C_8H_8DClNO$ $(M+H)^+$: 171.0430, found: 171.0430.

**ethyl-2-D 2-(4-chlorophenoxy)-2-methylpropanoate (6s)**

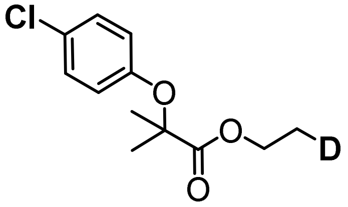


**6s**

**Condition C:** a dried 5 mL glass vial was charged with **5s** (55.4 mg, 0.20 mmol), photocatalyst **PC4** (11.2 mg, 0.040 mmol), (*n*PrS)$_2$ (10 μL, 0.060 mmol), $HCO_2Na$ (27.2 mg,

0.40 mmol), $D_2O$ (200 μL) and DMSO (1.0 mL) under air and then performed in a sealed vessel. The glass vial was positioned approximately 3 cm away from a 50 W blue LEDs lamp ($\lambda_{max}$ = 400 nm). After being stirred at room temperature (~ 30 °C under irradiation) for 72 h, the reaction mixture was purified by flash chromatography on silica gel (eluted with PE:EtOAc = 30:1) to afford product **6s** (17.5 mg, 0.072 mmol, 36% yield, 96% D) as a colorless oil.

$^1$H NMR (600 MHz, $CDCl_3$) δ 7.19 (d, $J$ = 8.7 Hz, 2H), 6.78 (d, $J$ = 8.7 Hz, 2H), 4.22 (t, $J$ = 7.0 Hz, 2H), 1.57 (s, 6H), 1.24 (dt, $J$ = 8.7, 7.2 Hz, 2.04H).

$^{13}$C NMR (151 MHz, $CDCl_3$) δ 174.00, 154.05, 129.11, 127.21, 120.53, 79.49, 61.50, 25.29, 13.81 (t, $J$ = 19.7 Hz).

$^2$H NMR (92 MHz, $CH_2Cl_2$) δ 1.42 (s, 1D).

IR (film): ν (cm$^{-1}$) 2090, 2940, 2186, 1876, 1732, 1594, 1489, 1386, 1284, 1239, 1142, 1092, 968, 828, 670.

HRMS (ESI-TOF, m/z) calcd for $C_{12}H_{15}DClO_3$ $(M+H)^+$: 244.0845, found: 244.0845.

**1-chloro-4-(propoxy-3-d)benzene (6t)**

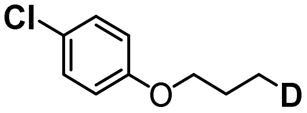


**6t**

**Condition C:** a dried 5 mL glass vial was charged with **5t** (41.0 mg, 0.20 mmol), photocatalyst **PC4** (11.2 mg, 0.040 mmol), $(n\text{PrS})_2$ (10 μL, 0.060 mmol), $HCO_2Na$ (27.2 mg, 0.40 mmol), $D_2O$ (200 μL) and DMSO (1.0 mL) under air and then performed in a sealed vessel. The glass vial was positioned approximately 3 cm away from a 50 W blue LEDs lamp ($\lambda_{max}$ = 400 nm). After being stirred at room temperature (~ 30 °C under irradiation) for 72 h, the reaction mixture was purified by flash chromatography on silica gel (eluted with PE) to afford product **6t** (16.8 mg, 0.098 mmol, 49% yield, 86% D) as a colorless oil.

$^1$H NMR (600 MHz, $CDCl_3$) δ 7.22 (d, $J$ = 8.8 Hz, 2H), 6.82 (d, $J$ = 8.8 Hz, 2H), 3.88 (t, $J$ = 6.6 Hz, 2H), 1.80 (dd, $J$ = 14.1, 7.0 Hz, 2H), 1.03 – 0.99 (m, 2.14H).

$^{13}C$ NMR (151 MHz, $CDCl_3$) δ 157.76, 129.26, 125.28, 115.77, 69.80, 22.45, 10.21 (t, $J$ = 19.1 Hz).

$^{2}H$ NMR (92 MHz, $CH_2Cl_2$) δ 0.97 (s, 1D).

IR (film): ν ($cm^{-1}$) 2963, 2872, 2182, 1596, 1492, 1471, 1392, 1285, 1244, 1169, 1092, 956, 823, 663.

HRMS (ESI-TOF, m/z) calcd for $C_9H_{10}DClNaO$ $(M+H)^+$: 194.0453, found: 194.0443.

### 3.6 Gram-Scale Synthesis of Deuterated Zytiga *via* Selective Deuterodechlorination

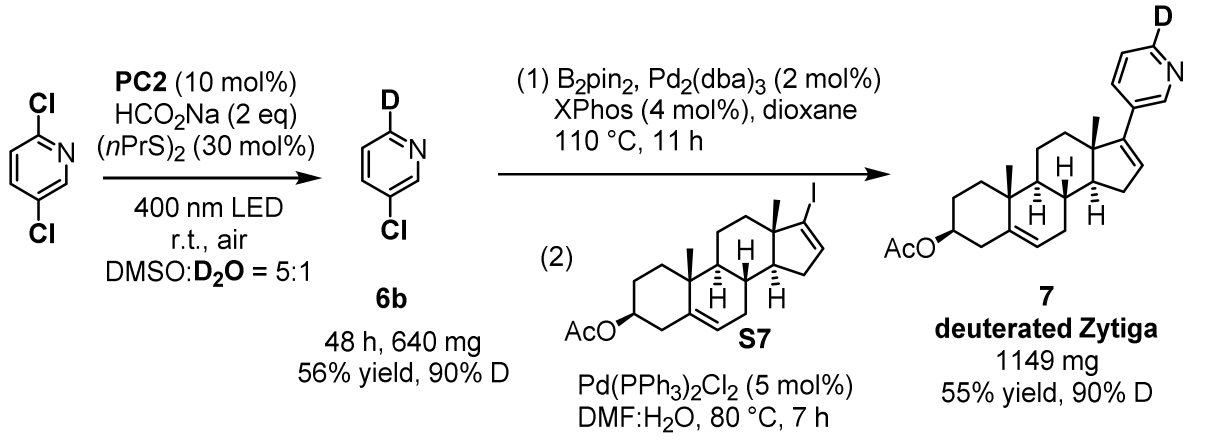


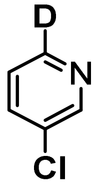

**6b**

A dried 100 mL flask was charged with 2,5-dichloropyridine **5b** (1480.0 mg, 10.0 mmol), photocatalyst **PC2** (808 mg, 1.0 mmol), ($n$PrS)$_2$ (500 μL, 3.0 mmol), $HCO_2Na$ (1360 mg, 20.0 mmol), $D_2O$ (10 mL) and DMSO (50 mL) under air and then performed in a sealed vessel. The flask was positioned approximately 3 cm away from a 50 W blue LEDs lamp ($\lambda_{max}$ = 400 nm). After being stirred at room temperature (~ 30 °C under irradiation) for 48 h, the reaction mixture was extracted with $Et_2O$. The combined organic layer was dried over

anhydrous $Na_2SO_4$ and concentrated under reduced pressure. The residue was subjected to silica gel chromatography (eluted with pentane:$Et_2O$ = 20:1) to afford product **6b** (640 mg, 5.6 mmol, 56% yield, 90% D) as a pale yellow oil.

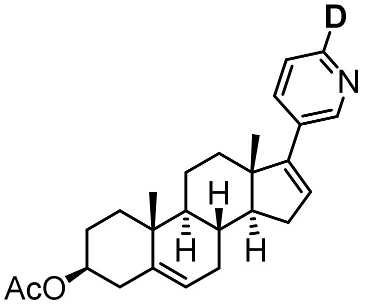

**7**

A 250 mL flask was charged with bis(pinacolato)diboron (4268 mg, 16.8 mmol), KOAc (1653 mg, 16.8 mmol), $Pd_2(dba)_3$ (51.7 mg, 0.056 mmol), XPhos (107.8 mg, 0.112 mmol) and 1,4-dioxane (20 mL) under argon. The reaction mixture was stirred at 110 °C. **6b** (640 mg, 5.6 mmol) in 1,4-dioxane (20 mL) was added over 1 h. After continuing stirring for additional 11 h at 110 °C, the reaction mixture was extracted with EtOAc. The combined organic layer was dried over anhydrous $Na_2SO_4$ and concentrated under reduced pressure. The residue was purified via precipitation (hexane:EtOAc) to afford crude product 3-(4,4,5,5-tetramethyl-1,3,2-dioxaborolan-2-yl)pyridine as a yellow solid.

A 100 mL flask was charged with 3-(4,4,5,5-tetramethyl-1,3,2-dioxaborolan-2-yl)pyridine (obtained above), **S7** (2343 mg, 5.32 mmol), $K_2CO_3$ (828 mg, 6.0 mmol), $Pd(PPh_3)_2Cl_2$ (187 mg, 0.266 mmol), DMF (25 mL) and $H_2O$ (5 mL) under argon. The reaction mixture was stirred for 7 h at 80 °C, then extracted with EtOAc. The combined organic layer was dried over anhydrous $Na_2SO_4$ and concentrated under reduced pressure. The residue was subjected to silica gel chromatography (eluted with PE:EtOAc = 5:1) to afford deuterated Zytiga **7** (1149 mg, 55% yield, 1.92 mmol, 90% D) as a white solid.

$^1$H NMR (600 MHz, $CDCl_3$) δ 8.60 (s, 1H), 8.44 (d, $J$ = 3.6 Hz, 0.1H), 7.63 (d, $J$ = 7.8 Hz, 1H), 7.20 (d, $J$ = 7.8 Hz, 1H), 5.97 (s, 1H), 5.40 (d, $J$ = 3.9 Hz, 1H), 4.63 – 4.56 (m, 1H), 2.35 – 2.25 (m, 3H), 2.04 – 1.99 (m, 5H), 1.85 (d, $J$ = 10.0 Hz, 2H), 1.73 – 1.47 (m, 8H), 1.12 – 1.01 (m, 8H).

$^{13}C$ NMR (151 MHz, $CDCl_3$) δ 170.53, 151.64, 147.86, 147.48 (t, *J* = 25.9 Hz), 140.02, 133.74, 133.00, 129.25, 122.91, 122.29, 73.86, 57.46, 50.25, 47.32, 38.13, 36.91, 36.78, 35.20, 31.80, 31.50, 30.40, 27.74, 21.44, 20.82, 19.26, 16.58.

$^{2}H$ NMR (92 MHz, $CH_2Cl_2$) δ 8.34 (s, 1D).

IR (film): ν ($cm^{-1}$) 3039, 2936, 2852, 2258, 1731, 1599, 1463, 1438, 1373, 1246, 1136, 1033, 904, 737, 634.

HRMS (ESI-TOF, m/z) calcd for $C_{26}H_{33}DNO_2$ $(M+H)^+$: 393.2647, found: 393.2647.

# 4. Mechanistic Investigations

## 4.1 Cyclic Voltammetry Analysis

Voltammetric experiments were conducted with a computer-controlled Shanghai Chen Hua CHI660D containing glassy carbon electrode serving as the working electrode, saturated calomel reference electrode, Pt wire auxiliary electrode.

All solutions used for the voltammetric experiments were deoxygenated by purging with high purity argon gas and measurements were performed in a Faraday cage at room temperature (22 ± 2 °C). The supporting electrolyte, tetrabutylammonium hexafluorophosphate ($n$Bu$_4$NPF$_6$), was purchased from commercial suppliers Aldrich.

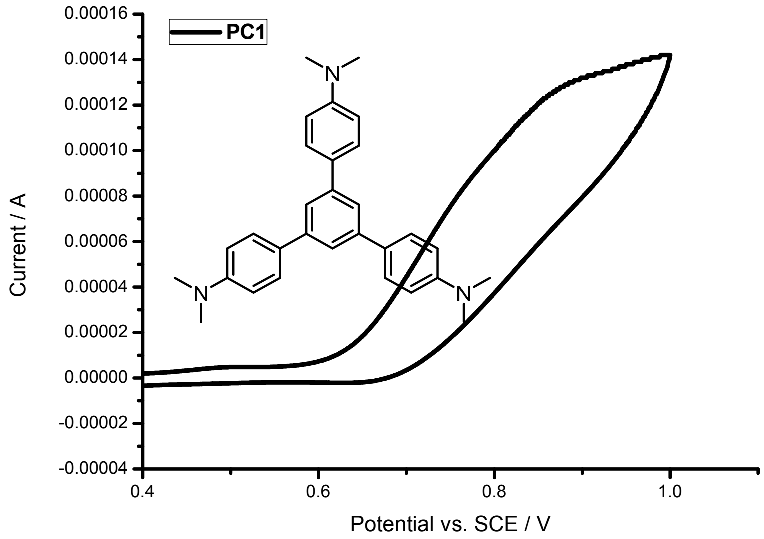


**Supplementary Fig. 2** Cyclic voltammograms of **PC1** in DMSO (1.0 mM) containing 0.1 M $n$Bu$_4$NPF$_6$. Scan rate: 0.1 V/s. $E^{ox}$ = +0.87 V.

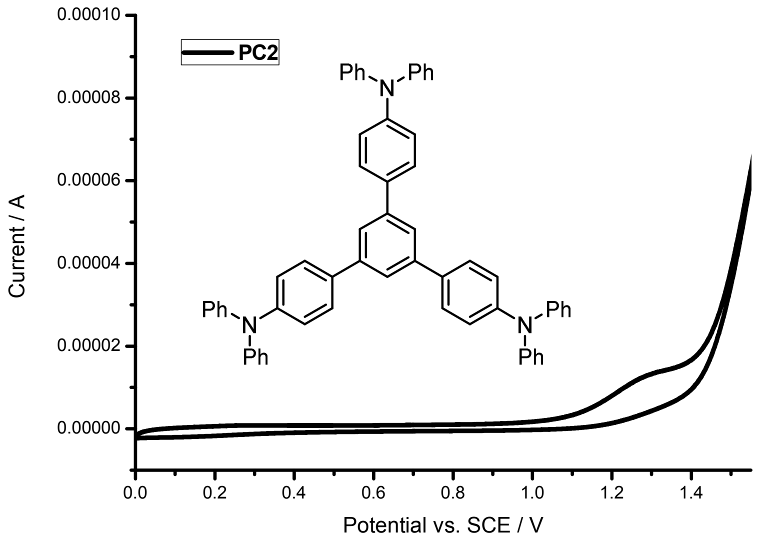


**Supplementary Fig. 3** Cyclic voltammograms of **PC2** in DMSO (1.0 mM) containing 0.1 M $n\mathrm{Bu_4NPF_6}$. Scan rate: 0.1 V/s. $E^{ox}$ = +1.30 V.

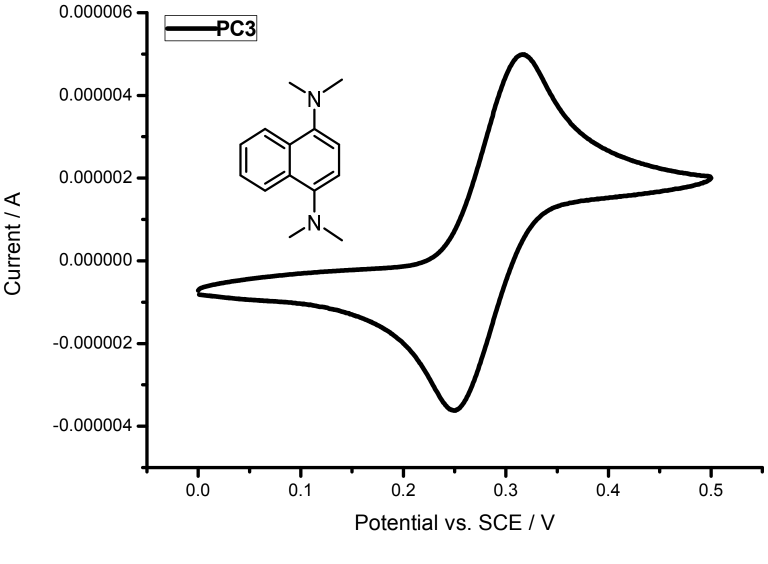


**Supplementary Fig. 4** Cyclic voltammograms of **PC3** in DMSO (1.0 mM) containing 0.1 M $n\mathrm{Bu_4NPF_6}$. Scan rate: 0.1 V/s. $E_{1/2}^{ox}$ = +0.29 V.

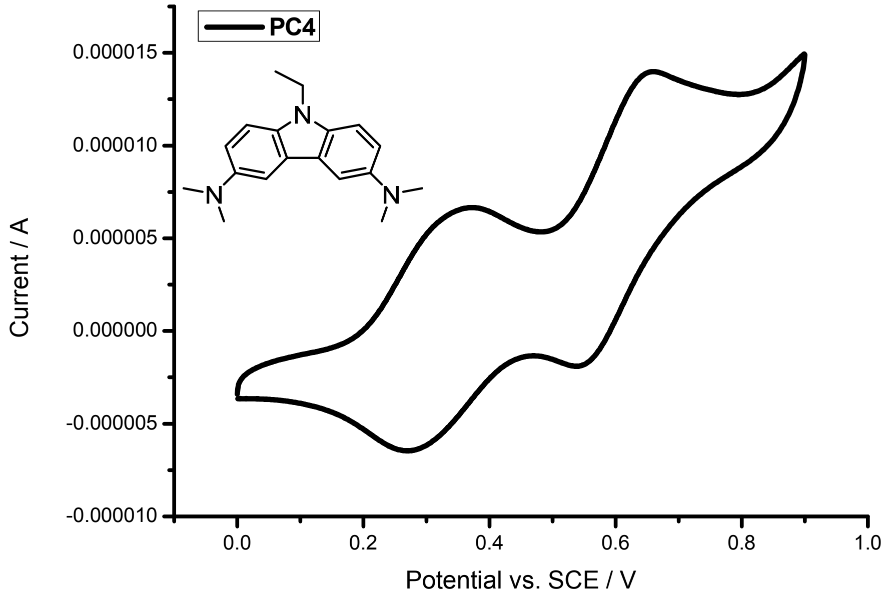


**Supplementary Fig. 5** Cyclic voltammograms of **PC4** in DMSO (1.0 mM) containing 0.1 M $n\mathrm{Bu_4NPF_6}$. Scan rate: 0.1 V/s. $E_{1/2}^{\mathrm{ox}}$ = +0.32 V.

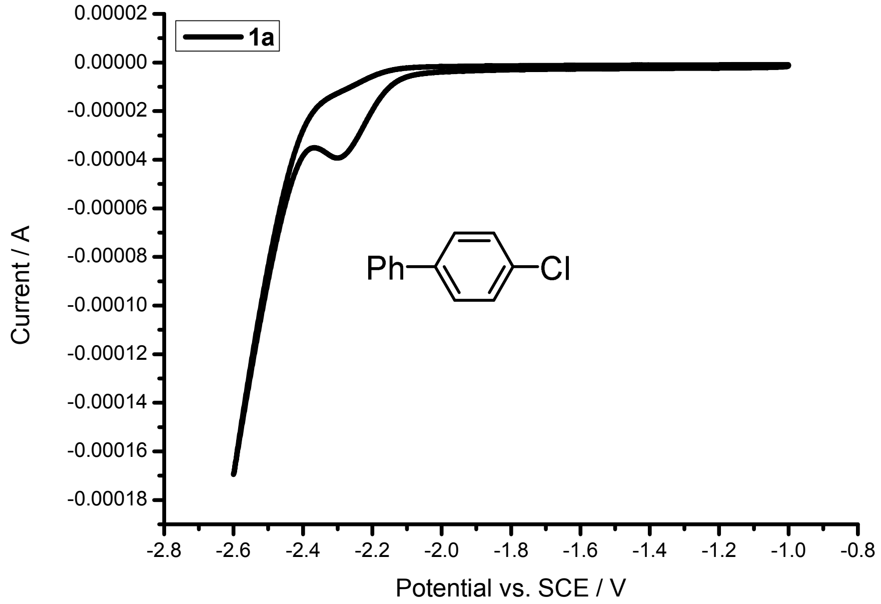


**Supplementary Fig. 6** Cyclic voltammograms of **1a** in DMSO (2.0 mM) containing 0.1 M $n\mathrm{Bu_4NPF_6}$. Scan rate: 0.1 V/s. $E_{red}$ = -2.30 V.

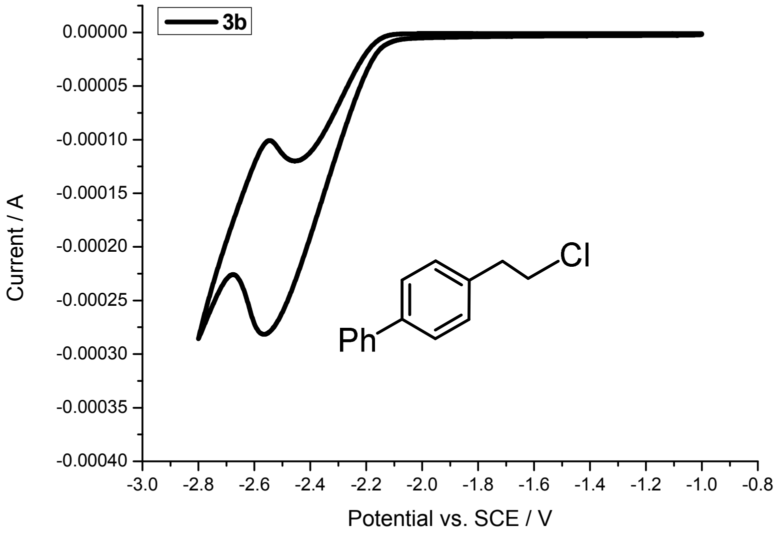


**Supplementary Fig. 7** Cyclic voltammograms of **3b** in DMSO (2.0 mM) containing 0.1 M *n*$Bu_4NPF_6$. Scan rate: 0.1 V/s. $E_{red}$ = -2.60 V.

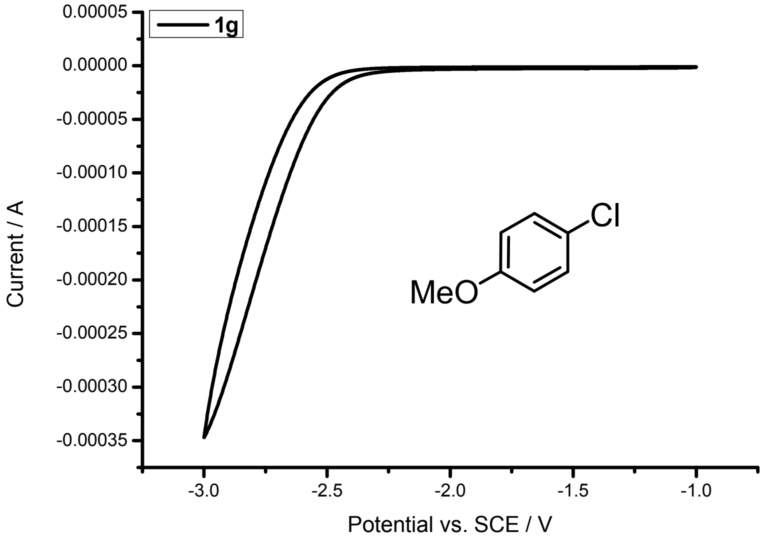


**Supplementary Fig. 8** Cyclic voltammograms of **1g** in DMSO (2.0 mM) containing 0.1 M *n*$Bu_4NPF_6$. Scan rate: 0.1 V/s. $E_{red}$ ≈ -2.90 V.

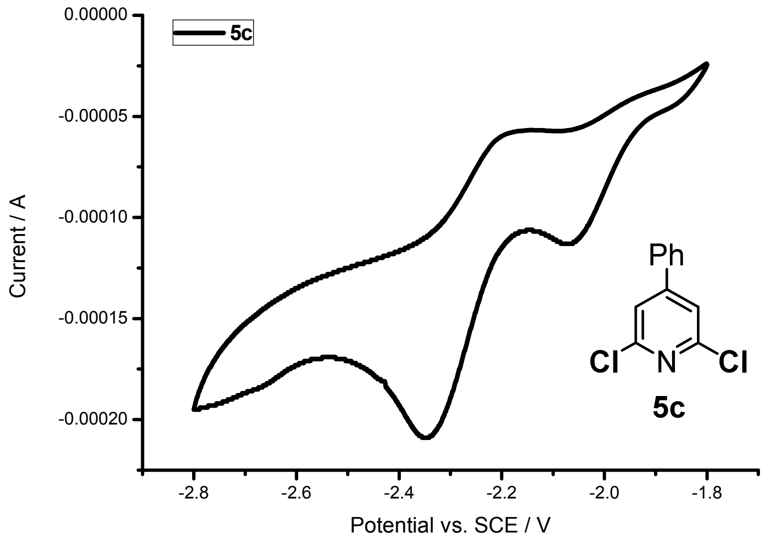


**Supplementary Fig. 9** Cyclic voltammograms of **5c** in $CH_3CN$ (2.0 mM) containing 0.1 M $n$$Bu_4NPF_6$. Scan rate: 0.1 V/s. $E_{red,1}$ = -2.3 V, $E_{red,2}$ = -2.1 V.

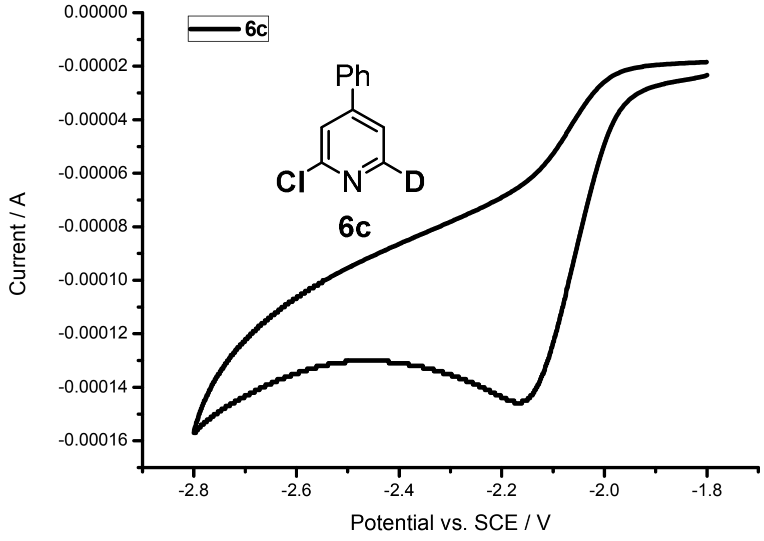


**Supplementary Fig. 10** Cyclic voltammograms of **6c** in $CH_3CN$ (0.20 mM) containing 0.1 M $n$$Bu_4NPF_6$. Scan rate: 0.1 V/s. $E_{red}$ = -2.2 V.

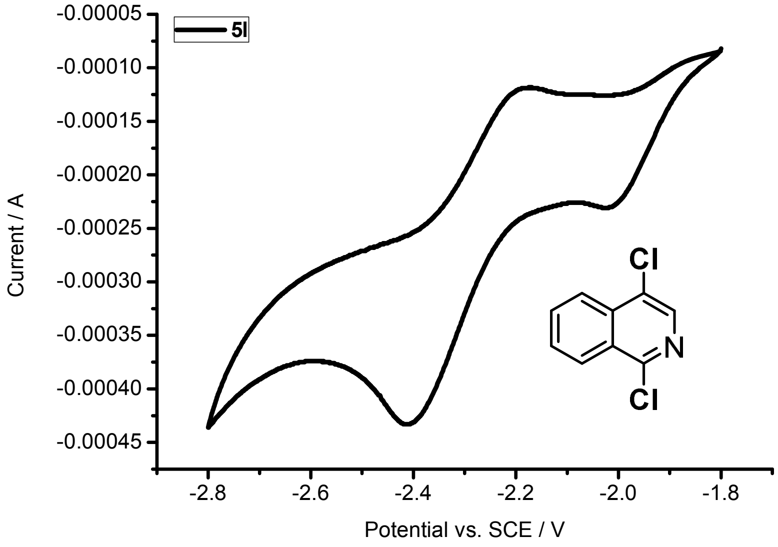


**Supplementary Fig. 11** Cyclic voltammograms of **5l** in $CH_3CN$ (2.0 mM) containing 0.1 M $n$$Bu_4NPF_6$. Scan rate: 0.1 V/s. $E_{red,1}$ = -2.3 V, $E_{red,2}$ = -2.0 V.

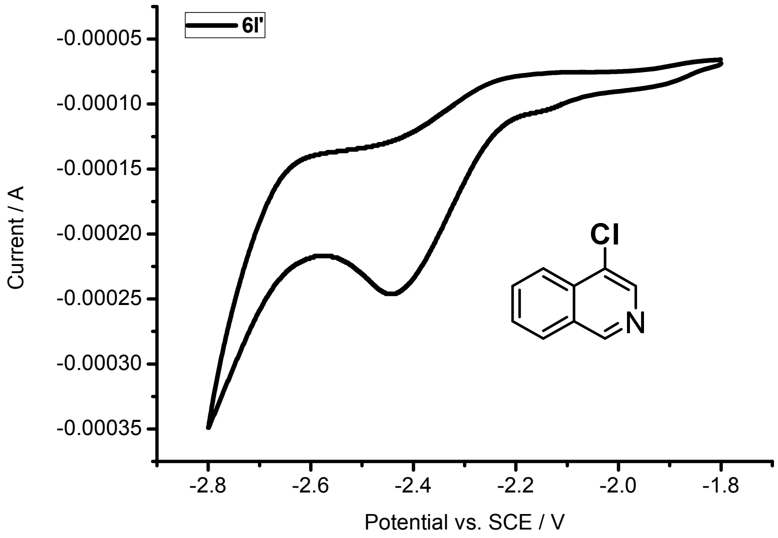


**Supplementary Fig. 12** Cyclic voltammograms of **6l'** in $CH_3CN$ (2.0 mM) containing 0.1 M $n$$Bu_4NPF_6$. Scan rate: 0.1 V/s. $E_{red}$ = -2.4 V.

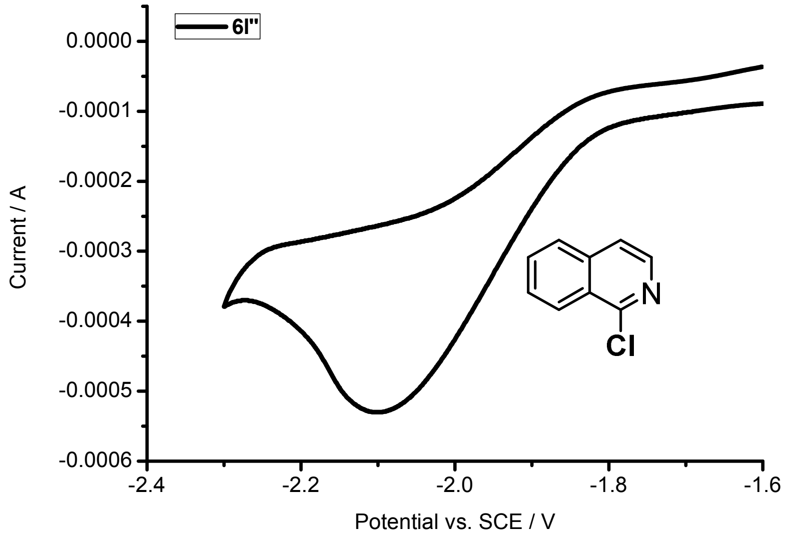


**Supplementary Fig. 13** Cyclic voltammograms of **6l''** in $CH_3CN$ (2.0 mM) containing 0.1 M $n$Bu$_4$NPF$_6$. Scan rate: 0.1 V/s. $E_{red}$ = -2.1 V.

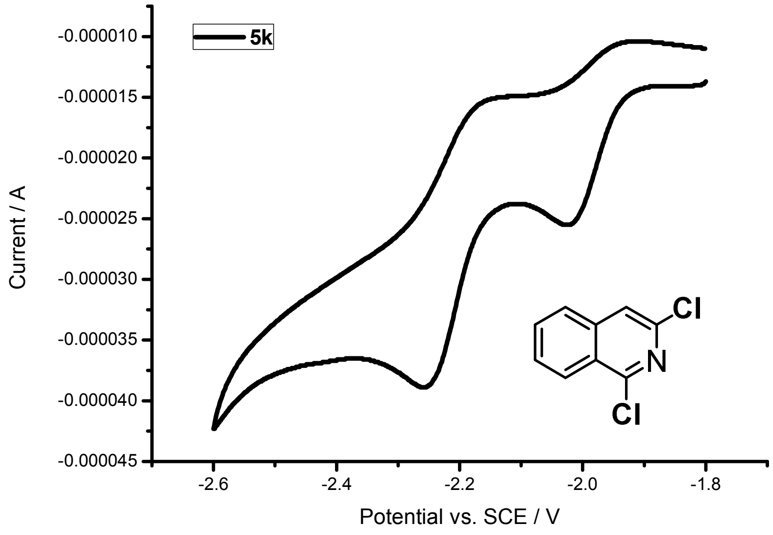


**Supplementary Fig. 14** Cyclic voltammograms of **5k** in $CH_3CN$ (2.0 mM) containing 0.1 M $n$Bu$_4$NPF$_6$. Scan rate: 0.1 V/s.

## 4.2 UV/Vis-Absorption Spectra of the Photocatalysts

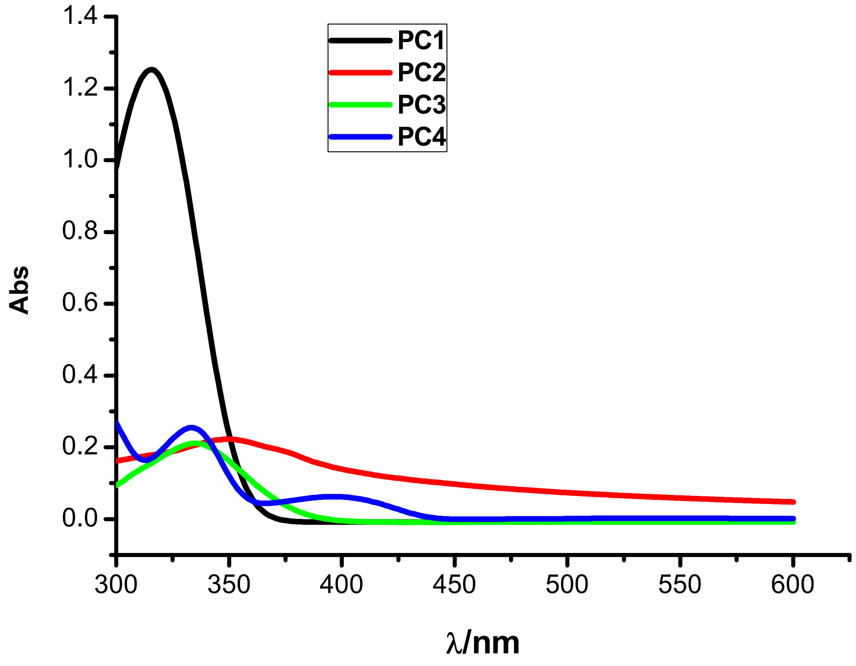


**Supplementary Fig. 15** UV-Vis spectra of **PC1−PC4**. The sample was prepared as a 0.10 mM solution in DMSO and used freshly for the measurement.

### 4.3 Evaluation of Excited State Potentials of the Photocatalysts

Fluorescence spectra were recorded on a Hitachi F-7000 Fluorescent Spectrophotometer in a 10.0 mm quartz cuvette. The sample **PC1−PC4** was prepared as a 0.10 mM solution in DMSO and used freshly for the measurement.

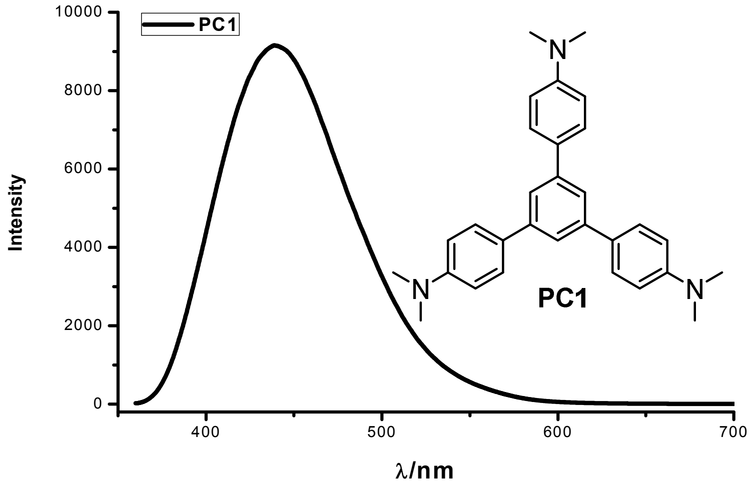


**Supplementary Fig. 16** Fluorescence emission spectrum of **PC1** (excited at 360 nm). The $\lambda_{onset,em}$ was estimated to be 366 nm. The $\lambda_{max,em}$ was estimated to be 440 nm.

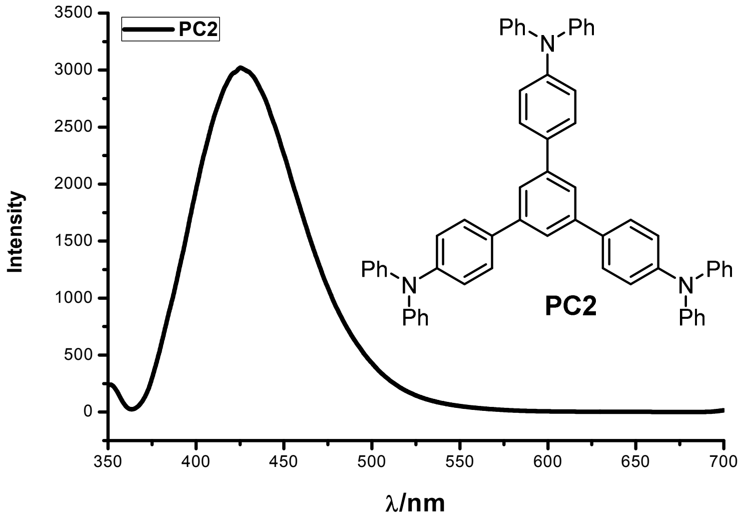


**Supplementary Fig. 17** Fluorescence emission spectrum of **PC2** (excited at 350 nm). The $\lambda_{onset,em}$ was estimated to be 367 nm. The $\lambda_{max,em}$ was estimated to be 426 nm.

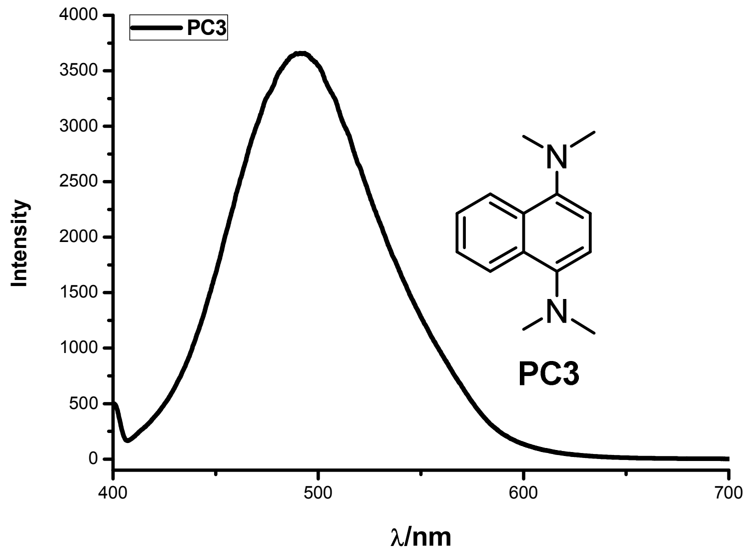


**Supplementary Fig. 18** Fluorescence emission spectrum of **PC3** (excited at 400 nm). The $\lambda_{onset,em}$ was estimated to be 414 nm. The $\lambda_{max,em}$ was estimated to be 491 nm.

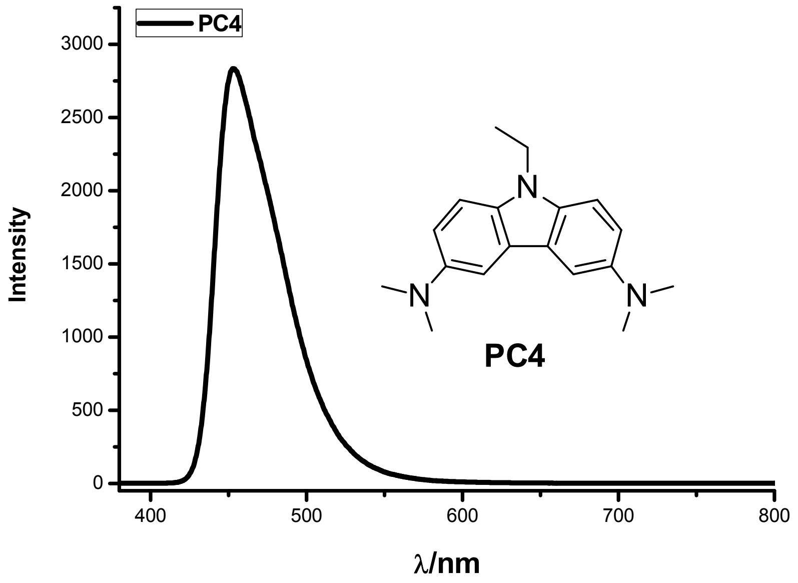


**Supplementary Fig. 19** Fluorescence emission spectrum of **PC4** (excited at 370 nm). The $\lambda_{onset,em}$ was estimated to be 425 nm. The $\lambda_{max,em}$ was estimated to be 453 nm.

***Remarks:*** Using $\lambda_{onset,em}$, $\lambda_{max,em}$ and $E^{ox}$, the excited state reduction potential was determined for $E$*(**PC**$^{+\cdot}$/**PC***) according to the following equations: $E$*(**PC**$^{+\cdot}$/**PC***) = $E^{ox}$ - $E^{0,0}$, where $E^{0,0}$= $hc/\lambda$ = 1240 nm/λ.

$E$*(**PC1**$^{+\cdot}$/**PC1***) = -2.52 ~ -1.95 V

$E$*(**PC2**$^{+\cdot}$/**PC2***) = -2.08 ~ -1.61 V

$E$*(**PC3**$^{+\cdot}$/**PC3***) = -2.71 ~ -2.24 V

$E$*(**PC4**$^{+\cdot}$/**PC4***) = -2.60 ~ -2.42 V

### 4.4 Estimating Lifetime of the Excited State of the Photocatalysts

Estimating lifetime of excited state of the photocatalysts was based on the ultrafast transient absorption spectroscopic techniques. The luminescence decays were measured on an Edingburg FLS980 spectrometer. The sample compartment was home-built and designed as 10x10 mm cuvettes in 90° geometry between excitation and detection. The solution of **PC1**-**PC4** in DMSO (0.10 mM) was excited at 378 nm. All decay traces were fitted by iterative reconvolution with an experimental instrument response function recorded directly after decay acquisition.

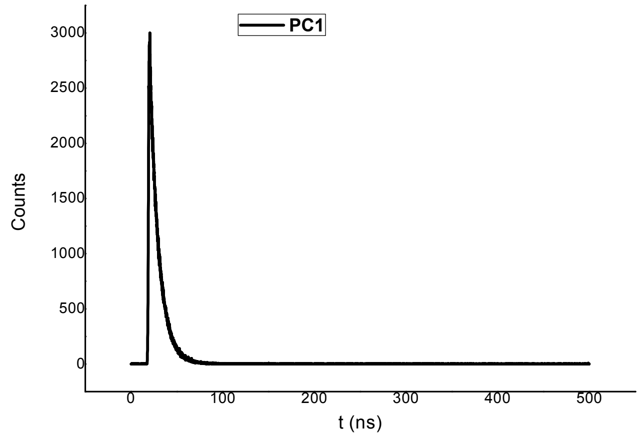


**Supplementary Fig. 20** Luminescence decays of **PC1** in DMSO detected at 25 °C after excitation at 378 nm. τ =10 ns.

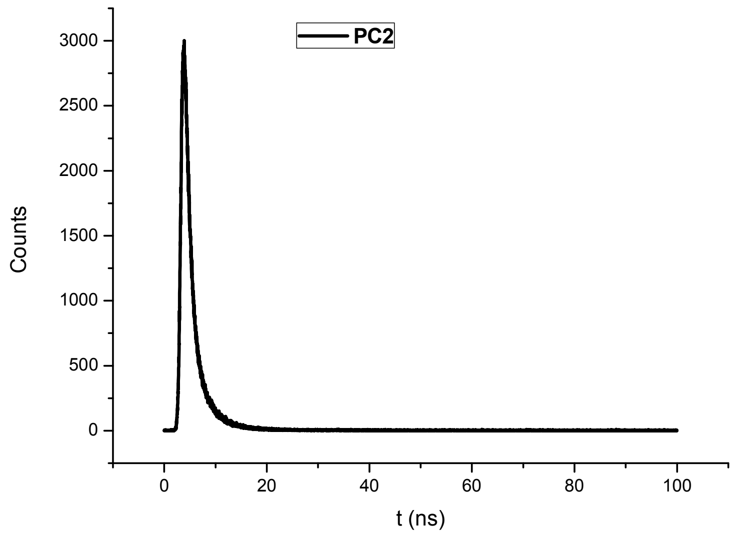


**Supplementary Fig. 21** Luminescence decays of **PC2** in DMSO detected at 25 °C after excitation at 378 nm. τ = 2 ns.

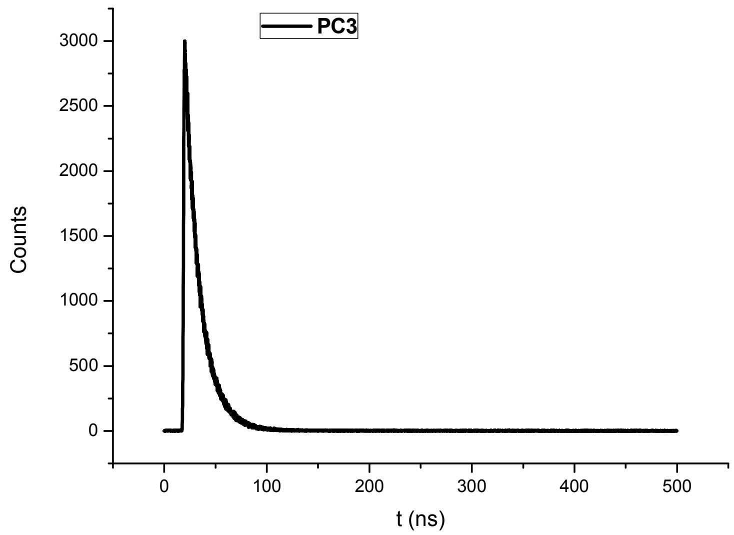


**Supplementary Fig. 22** Luminescence decays of **PC3** in DMSO detected at 25 °C after excitation at 378 nm. τ =15 ns.

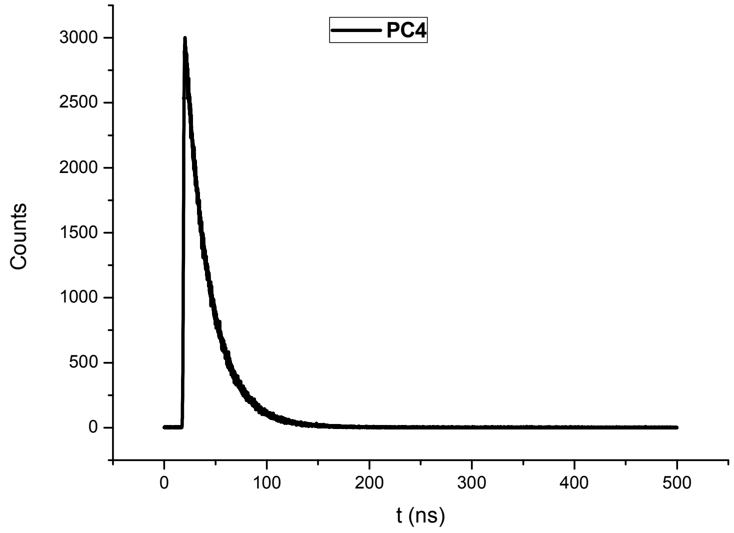


**Supplementary Fig. 23** Luminescence decays of **PC4** in DMSO detected at 25 °C after excitation at 378 nm. $\tau$ = 24 ns.

## 4.5 $CO_2$ Trapping Experiment

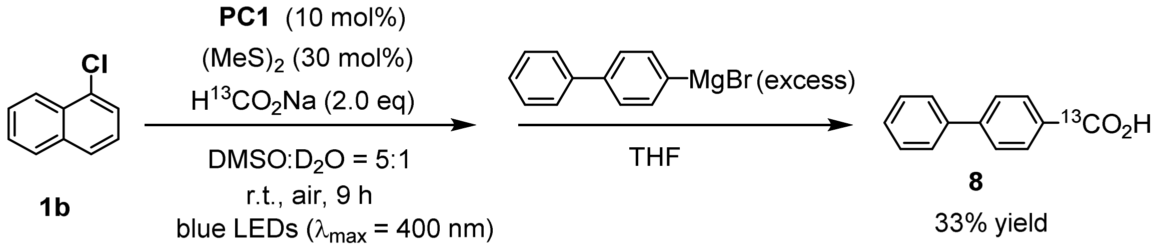


A dried 25 mL Schlenk tube (**A**) was charged with aryl chloride **1b** (162.6 mg, 1.0 mmol), photocatalyst **PC1** (43.6 mg, 0.10 mmol), $(MeS)_2$ (30 μL, 0.30 mol), $H^{13}CO_2Na$ (138.0 mg, 2.0 mmol), $D_2O$ (1.0 mL) and DMSO (5.0 mL). The Schlenk tube (**A**) was positioned approximately 3 cm away from a 50 W blue LEDs lamp ($\lambda_{max}$ = 400 nm). After being stirred at room temperature (~ 30 °C under irradiation) for 9 h, the Schlenk tube (**A**) was bubbled with argon, and the resulting gas flow was incorporated into a Schlenk flask (**B**) containing (1,1'-biphenyl)-4-ylmagnesium bromide (30 mL, 0.5 M in THF) with protection of an argon balloon (Supplementary Fig. 24). After being stirred for 30 min, the excess Grignard reagents were quenched with $H_2O$ at -40 °C. Diluted HCl was added until the solution was acidic and

transparent. The solution was extracted with $CH_2Cl_2$ for three times. The combined organic layer was dried over anhydrous $Na_2SO_4$ and concentrated under reduced pressure. The residue was subjected to silica gel chromatography (eluted with to $CH_2Cl_2$:EtOAc = 5:1) to afford product **8** (66.6 mg, 0.33 mmol, 33% yield) as a white solid.

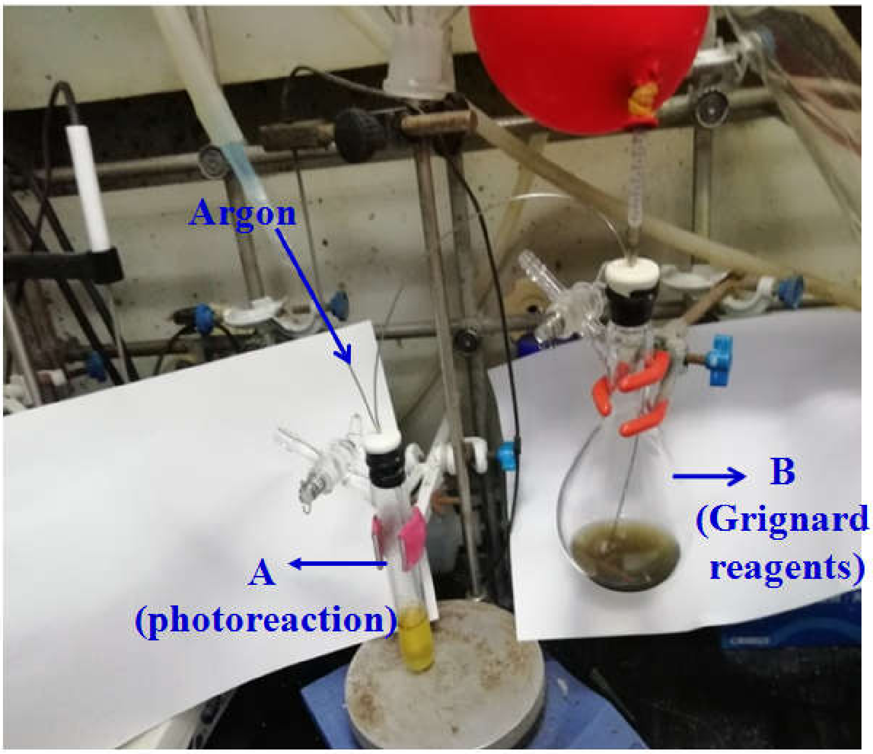


**Supplementary Fig. 24** Reaction set-up of $CO_2$ trapping experiment.

**[1,1'-biphenyl]-4-carboxylic-$^{13}$C acid (8)**

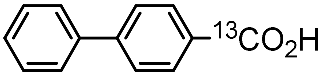

**8**

$^{1}$H NMR (600 MHz, $(CD_3)_2SO$) δ 12.98 (s, 1H), 8.07 – 7.97 (m, 2H), 7.80 (d, *J* = 8.1 Hz, 2H), 7.77 – 7.70 (m, 2H), 7.50 (t, *J* = 7.7 Hz, 2H), 7.43 (t, *J* = 7.4 Hz, 1H).

$^{13}$C NMR (151 MHz, $(CD_3)_2SO$) δ 167.63 (**$^{13}$C**), 144.77, 139.50, 130.44, 129.56, 128.77, 127.44, 127.30, 127.27.

IR (film): ν (cm$^{-1}$) 3445, 1634, 1485, 1448, 1407, 1272, 1191, 1116, 1007, 860, 732, 695, 670.

HRMS (ESI-TOF, m/z) calcd for $C_{12}{}^{13}CH_{10}NaO_2$ $(M+Na)^+$: 222.0607, found: 222.0606.

### 4.6 Stern-Volmer Luminescence Quenching Studies

Stern-Volmer luminescence quenching studies were carried out using a $1\times10^{-4}$ M solution of **PC** and different concentrations of 1-chloronaphthalene, biphenylethyl chloride, $(RS)_2$ in DMSO and $HCO_2Na$ in DMSO:$H_2O$ (2:1) at room temperature. The samples were prepared in 4 mL quartz cuvettes, equipped with PTFE stoppers. After degassing with an argon stream for 1 minutes, the emission intensity was collected.

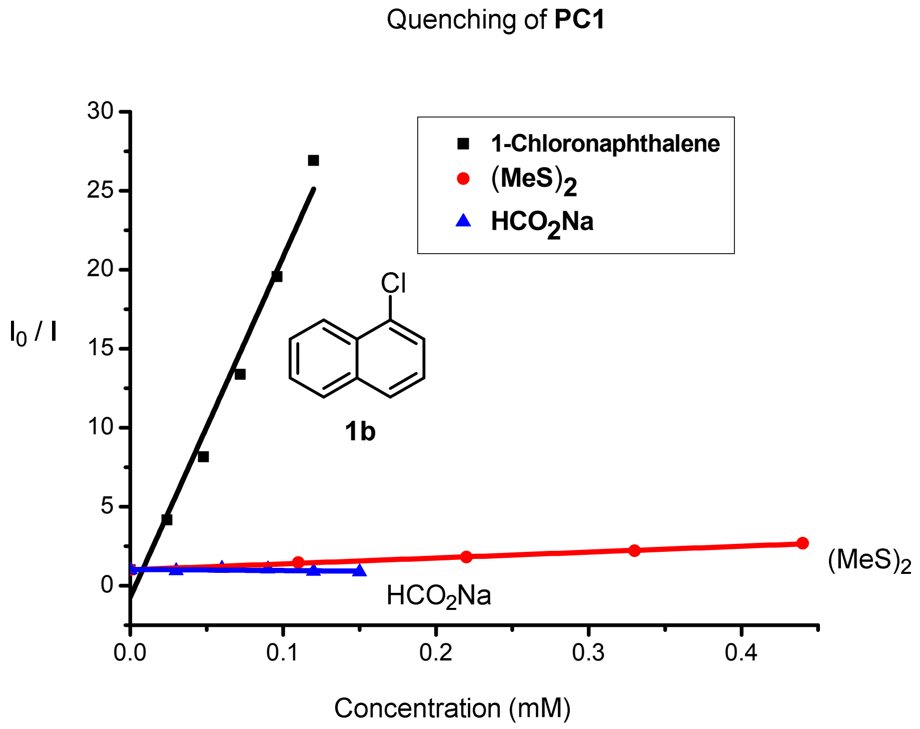


**Supplementary Fig. 25** Stern-Volmer luminescence quenching analysis for 1-chloronaphthalene using **PC1** ($1\times10^{-4}$ M). The solutions were irradiated at 360 nm and the luminescence was measured at 440 nm. $I_0$ = emission intensity of the photocatalyst in isolation at the specified wavelength; I = observed intensity as a function of the quencher concentration.

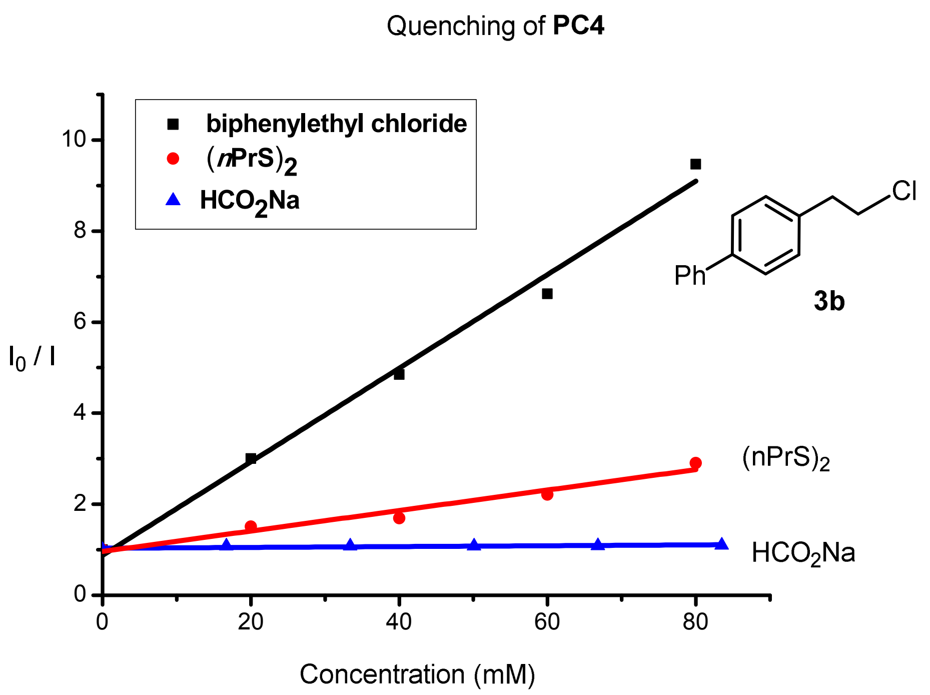


**Supplementary Fig. 26** Stern-Volmer luminescence quenching analysis for biphenylethyl iodide, biphenylethyl chloride, ($n$PrS)$_2$ and $HCO_2Na$ using **PC4** ($1\times10^{-4}$ M). $I_0$ = emission intensity of the photocatalyst in isolation at the specified wavelength; I = observed intensity as a function of the quencher concentration. The solutions were irradiated at 370 nm and the luminescence was measured at 453 nm.

## 4.7 Thiyl Radical Scrambling Experiment

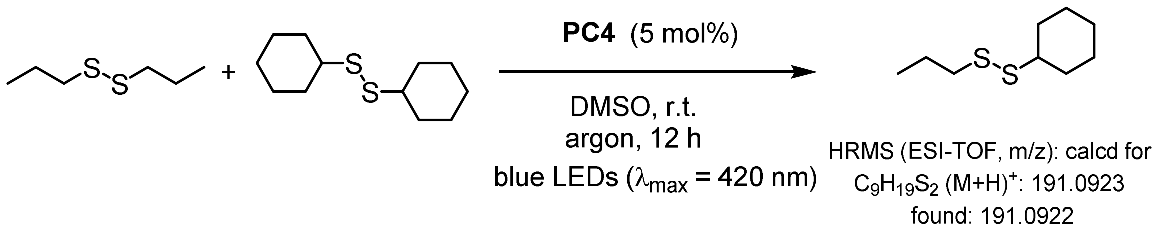


A dried 10 mL Schlenk tube was charged with dipropyl disulfide (15.0 mg, 0.10 mmol), photocatalyst **PC4** (1.4 mg, 0.0050 mmol), dicyclohexyl disulfide (23.0 mg, 0.10 mmol) DMSO (500 μL). The mixture was degassed *via* three freeze-pump-thaw cycles and the tube was finally backfilled with argon. The glass vial was positioned approximately 3 cm away

from a 50 W blue LEDs lamp ($\lambda_{max}$ = 400 nm). After being stirred at room temperature (~ 30 °C under irradiation) for 12 h, the reaction mixture was analysed by HRMS. HRMS (ESI-TOF, m/z): calcd for $C_9H_{19}S_2$ $(M+H)^+$: 191.0923, found: 191.0922.

### 4.8 Calculation of Triplet State Energies of the Photocatalysts

Computational Details

All density functional theory (DFT) calculations were performed using Gaussian 09 program.[13] The adiabatic $S_0$–$T_1$ gap for 9-ethyl-$N^3$,$N^3$,$N^6$,$N^6$-tetramethyl-9H-carbazole-3,6-diamine (**PC4**) were determined via the energy difference of the optimized singlet ground state structure and the first excited triplet structure. Geometries were optimized with M06 method[14] and 6-31G(d) basis set.[15-17] Frequency analysis was performed to ensure all geometries were minimal. Single-point energies were calculated using the 6-311+G(2d,p) basis set.[18] The solvent energies were calculated by SMD implicit solvent model.[19]

Coordinates and Energies

9-ethyl-$N^3$,$N^3$,$N^6$,$N^6$-tetramethyl-9H-carbazole-3,6-diamine (**PC4**)

Energy of $T_1$ : -1146.811047 Hartree

Energy of $S_0$ : -1146.907962 Hartree

$S_0$–$T_1$ Energy: 0.104354 Hartree (65.5 kcal mol$^{-1}$)

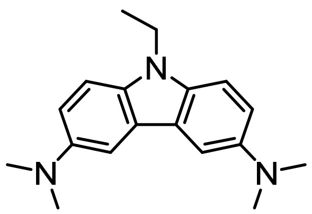

**PC4**

**Optimized structure for $S_0$:**

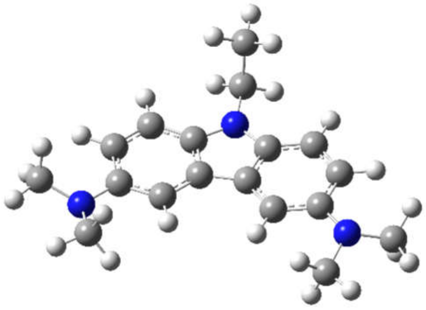

**M062X /6-311+G(2d,p) energy: -1146.907962 Hartree**

**Optimized structure for $T_1$:**

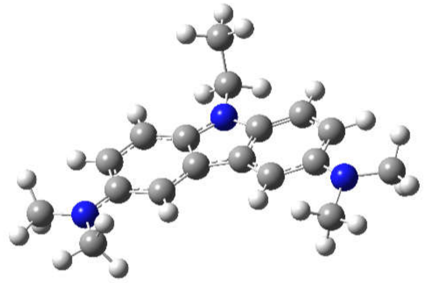

**M062X/6-311+G(2d,p) energy: -1146.811047 Hartree**

**$S_0$–$T_1$ Energy (6-311+G(2d,p)): 0.104354 Hartree** (65.5 kcal $mol^{-1}$)

**Supplementary Table 3** Standard orientation $S_0$ (coordinates)

| Atom | X | Y | Z |
|---|---|---|---|
| C | -1.11872300 | 1.31935000 | 0.18965300 |
| C | -0.72708000 | -0.03308700 | 0.08444800 |
| C | -1.68269900 | -1.04590800 | -0.04920400 |
| C | -3.04351900 | -0.71538400 | -0.10734300 |
| C | -3.40863200 | 0.65349100 | 0.00308000 |
| C | -2.47085000 | 1.66531300 | 0.14570100 |
| H | -1.35188600 | -2.07643300 | -0.11075200 |

| | | | |
|---|---|---|---|
| H | -4.45441500 | 0.93460100 | -0.02788700 |
| H | -2.79463500 | 2.69924200 | 0.22188900 |
| C | 0.72094300 | -0.04316300 | 0.15191200 |
| C | 1.11994800 | 1.30407800 | 0.29008900 |
| C | 1.67141700 | -1.06756300 | 0.08650200 |
| C | 2.47576600 | 1.63345100 | 0.34566400 |
| C | 3.03580100 | -0.75699900 | 0.16575400 |
| H | 1.33310800 | -2.09085900 | -0.03019900 |
| C | 3.40868400 | 0.60935400 | 0.28093800 |
| H | 2.80595600 | 2.66493600 | 0.42687400 |
| H | 4.45726800 | 0.87855500 | 0.32017900 |
| N | 0.00171600 | 2.11953500 | 0.34155500 |
| C | 0.01192300 | 3.57060500 | 0.33331100 |
| H | 0.87351200 | 3.90204900 | 0.92039800 |
| H | -0.88414700 | 3.91459500 | 0.85837300 |
| C | 0.06557900 | 4.14441200 | -1.07802700 |
| H | 0.97006200 | 3.80918700 | -1.59544800 |
| H | -0.80469300 | 3.82114200 | -1.65793500 |
| H | 0.07160200 | 5.23808300 | -1.04504900 |
| C | 3.59426600 | -3.07702700 | -0.28995300 |
| H | 4.45525000 | -3.74712100 | -0.27105000 |
| H | 2.84152300 | -3.48664100 | 0.38993400 |
| H | 3.17221600 | -3.07435800 | -1.30810000 |
| C | 5.35702300 | -1.37317900 | -0.26016400 |
| H | 5.80405000 | -0.68392200 | 0.46151200 |
| H | 5.98401900 | -2.26586200 | -0.28913800 |
| H | 5.37960500 | -0.89669600 | -1.25292300 |
| C | -5.38177500 | -1.37170700 | 0.11842400 |
| H | -5.79630100 | -0.56996500 | -0.49917500 |
| H | -6.01117300 | -2.25148900 | -0.02491600 |
| H | -5.44606400 | -1.06290500 | 1.17369500 |
| C | -3.62400400 | -3.07463900 | -0.07257800 |
| H | -4.48892400 | -3.72272500 | -0.22237400 |
| H | -2.85808700 | -3.37328400 | -0.79465300 |
| H | -3.22592600 | -3.24724700 | 0.94048400 |
| N | -4.02594600 | -1.69739500 | -0.29278200 |
| N | 4.01618300 | -1.75946400 | 0.14937300 |

**Supplementary Table 4** Standard orientation $T_1$ (coordinates)

| Atom | X | Y | Z |
|---|---|---|---|
| C | 1.12256700 | 1.28743300 | -0.17892000 |
| C | 0.71604800 | -0.07358600 | -0.04140400 |
| C | 1.70574700 | -1.07102600 | 0.07934100 |
| C | 3.07394900 | -0.70066300 | 0.08150600 |
| C | 3.43288500 | 0.65514900 | -0.06224900 |

| | | | |
|---|---|---|---|
| C | 2.44191600 | 1.66315100 | -0.18703500 |
| H | 1.40742100 | -2.10795100 | 0.17183700 |
| H | 4.47228800 | 0.95382900 | -0.07853500 |
| H | 2.73711300 | 2.70376100 | -0.28998800 |
| C | -0.70493300 | -0.08818600 | -0.06464200 |
| C | -1.14188800 | 1.28859100 | -0.21520000 |
| C | -1.67652500 | -1.09836900 | 0.03053200 |
| C | -2.48911800 | 1.65153500 | -0.25079900 |
| C | -3.06352700 | -0.73173400 | -0.02011600 |
| H | -1.38095100 | -2.13323600 | 0.14118500 |
| C | -3.45822900 | 0.63769900 | -0.15499300 |
| H | -2.78922500 | 2.68972100 | -0.34619800 |
| H | -4.50532600 | 0.90369700 | -0.18234500 |
| N | -0.04045200 | 2.07437700 | -0.30213900 |
| C | -0.03896300 | 3.52427600 | -0.40231800 |
| H | -0.93280700 | 3.82251600 | -0.95609000 |
| H | 0.83013300 | 3.81241000 | -1.00031700 |
| C | 0.00047500 | 4.17989100 | 0.97274100 |
| H | -0.88074100 | 3.89503100 | 1.55591700 |
| H | 0.89631000 | 3.87054000 | 1.51995200 |
| H | 0.01409500 | 5.26870000 | 0.87067400 |
| C | -3.63432400 | -3.10031400 | 0.19802300 |
| H | -4.53916900 | -3.70304000 | 0.24086000 |
| H | -3.03342100 | -3.42442200 | -0.65678800 |
| H | -3.05969600 | -3.25883900 | 1.11532400 |
| C | -5.42525500 | -1.35841800 | 0.00782700 |
| H | -5.66627300 | -0.85930500 | -0.93555000 |
| H | -6.01011100 | -2.27282300 | 0.08278000 |
| H | -5.69417700 | -0.69674500 | 0.83664700 |
| C | 5.42694600 | -1.32363200 | -0.08367600 |
| H | 5.79050100 | -0.52882500 | 0.57474100 |
| H | 6.06410900 | -2.19608100 | 0.06893500 |
| H | 5.54086300 | -0.98316600 | -1.12482200 |
| C | 3.68938600 | -3.06473600 | 0.02831000 |
| H | 4.57329900 | -3.68960800 | 0.16535900 |
| H | 2.94184500 | -3.38730900 | 0.75902800 |
| H | 3.28430800 | -3.24671000 | -0.97930200 |
| N | 4.05848200 | -1.67747800 | 0.24468900 |
| N | -4.00707700 | -1.69611600 | 0.06103900 |

### 4.9 Calculation of BDE (C-Cl) of Dichlorides

The calculated results of bond dissociation energies (BDE (C-Cl)) of dichlorides **5k** and **5l** were below.

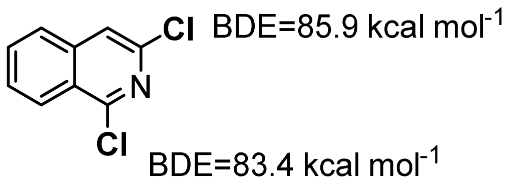
Cl
N
Cl
BDE=85.9 kcal mol-1
BDE=83.4 kcal mol-1

**5k**

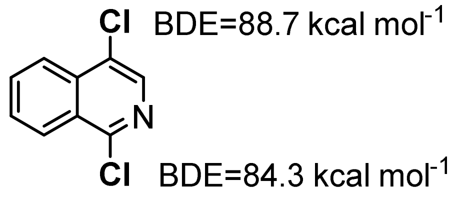
Cl
N
Cl
BDE=88.7 kcal mol-1
BDE=84.3 kcal mol-1

**5l**

## 5. Synthesis of Deuterated Drugs *via* Deuterodechlorination

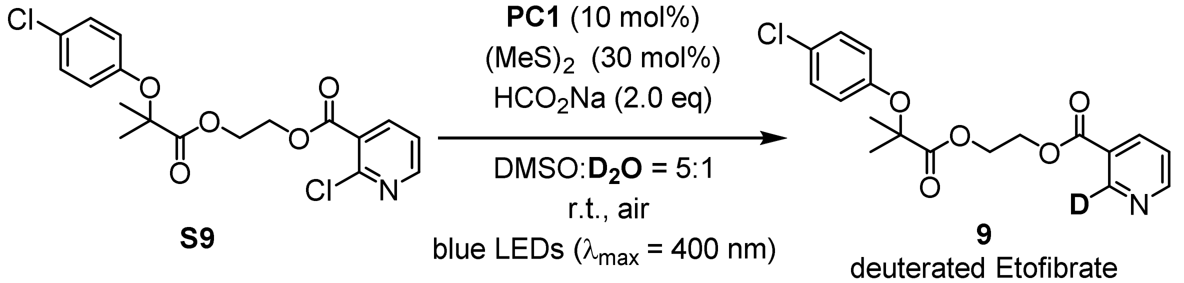


**S9**

**9**
deuterated Etofibrate
18 h, 88% yield
91% D

A dried 5 mL glass vial was charged with **S9** (79.6 mg, 0.20 mmol), photocatalyst **PC1** (8.7 mg, 0.020 mmol), $(MeS)_2$ (6 μL, 0.060 mmol), $HCO_2Na$ (27.2 mg, 0.40 mmol), $D_2O$ (200 μL) and DMSO (1.0 mL) under air and then performed in a sealed vessel. The glass vial was positioned approximately 3 cm away from a 50 W blue LEDs lamp ($\lambda_{max}$ = 400 nm). After being stirred at room temperature (~ 30 °C under irradiation) for 18 h, the reaction mixture was purified by flash chromatography on silica gel (eluted with PE:EtOAc = 3:1) to afford product deuterated Etofibrate **9** (64.2 mg, 0.176 mmol, 88% yield, 91% D) as a colorless oil.

$^1$H NMR (500 MHz, $CDCl_3$) δ 9.11 (d, *J* = 1.2 Hz, 0.09H), 8.75 (dd, *J* = 4.8, 1.4 Hz, 1H), 8.10 (dd, *J* = 7.9, 1.5 Hz, 1H), 7.34 (dd, *J* = 7.9, 4.9 Hz, 1H), 7.06 (d, *J* = 8.9 Hz, 2H), 6.72 (d, *J* = 8.9 Hz, 2H), 4.51 (td, *J* = 5.8, 1.8 Hz, 4H), 1.56 (s, 6H).

$^{13}$C NMR (151 MHz, $CDCl_3$) δ 173.88, 164.78, 153.90, 153.56, 150.42 (t, *J* = 28.3 Hz), 137.09, 129.11, 127.15, 125.39, 123.41, 120.13, 79.30, 62.95, 62.75, 25.27.

$^2$H NMR (92 MHz, $CH_2Cl_2$) δ 9.00 (s, 1D).

IR (film): ν ($cm^{-1}$) 3051, 2993, 2961, 2270, 1733, 1583, 1490, 1285, 1239, 1176, 1138, 1101, 829, 734, 671, 649.

HRMS (ESI-TOF, m/z) calcd for $C_{18}H_{18}DClNO_5$ $(M+H)^+$: 365.1009, found: 365.1006.

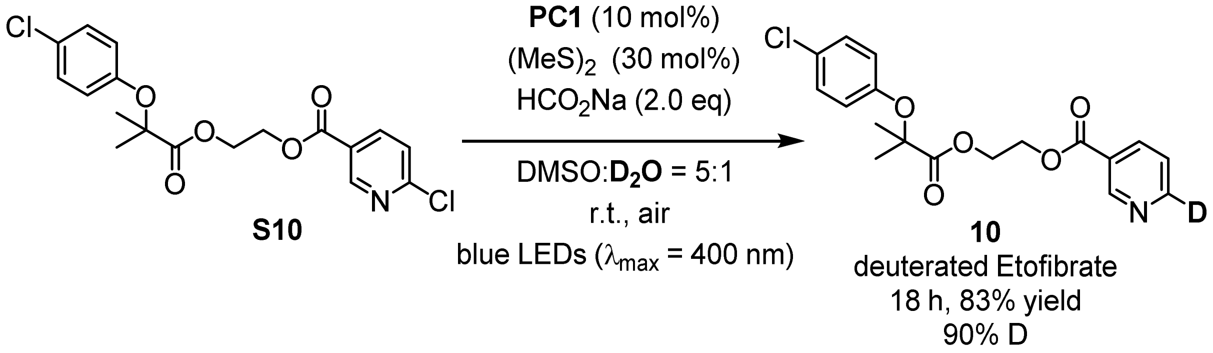

A dried 5 mL glass vial was charged with **S10** (79.6 mg, 0.20 mmol), photocatalyst **PC1** (8.7 mg, 0.020 mmol), $(MeS)_2$ (6 μL, 0.060 mmol), $HCO_2Na$ (27.2 mg, 0.40 mmol), $D_2O$ (200 μL) and DMSO (1.0 mL) under air and then performed in a sealed vessel. The glass vial was positioned approximately 3 cm away from a 50 W blue LEDs lamp ($\lambda_{max}$ = 400 nm). After being stirred at room temperature (~ 30 °C under irradiation) for 18 h, the reaction mixture was purified by flash chromatography on silica gel (eluted with PE:EtOAc = 3:1) to afford product deuterated Etofibrate **10** (60.5 mg, 0.166 mmol, 83% yield, 90% D) as a colorless oil.

$^1$H NMR (500 MHz, $CDCl_3$) δ 9.11 (s, 1H), 8.75 (d, $J$ = 4.8 Hz, 0.1H), 8.26 – 8.04 (m, 1H), 7.35 (d, $J$ = 7.9 Hz, 1H), 7.16 – 6.99 (m, 2H), 6.72 (dd, $J$ = 8.8, 1.2 Hz, 2H), 4.51 (dtd, $J$ = 6.1, 3.8, 2.4 Hz, 4H), 1.56 (d, $J$ = 1.2 Hz, 6H).

$^{13}$C NMR (126 MHz, $CDCl_3$) δ 173.87, 164.78, 153.91, 153.19 (t, $J$ = 28.8 Hz), 150.74, 137.12, 129.11, 127.19, 125.53, 123.25, 120.16, 79.32, 62.95, 62.75, 25.27.

$^2$H NMR (92 MHz, $CH_2Cl_2$) δ 8.60 (s, 1D).

IR (film): ν (cm$^{-1}$) 2994, 2959, 2260, 1736, 1590, 1490, 1385, 1274, 1239, 1151, 1024, 829, 735, 671, 636.

HRMS (ESI-TOF, m/z) calcd for $C_{18}H_{17}DClNNaO_5$ $(M+Na)^+$: 387.0828, found: 387.0832.

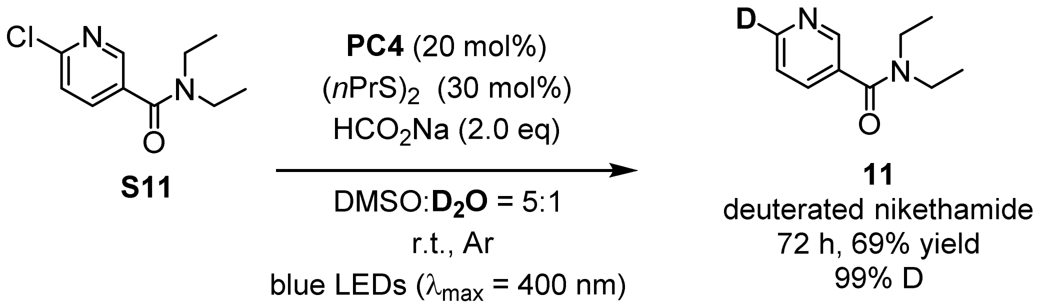


A dried 10 mL Schlenk tube was charged with **S11** (42.5 mg, 0.20 mmol), photocatalyst **PC4** (11.2 mg, 0.040 mmol), (*n*PrS)$_2$ (10 μL, 0.060 mmol), $HCO_2Na$ (27.2 mg, 0.40 mmol), $D_2O$ (200 μL) and DMSO (1.0 mL) under air and then performed in a sealed vessel. The mixture was degassed *via* three freeze-pump-thaw cycles. The Schlenk tube was positioned approximately 3 cm away from a 50 W blue LEDs lamp ($\lambda_{max}$ = 400 nm). After being stirred at room temperature (~ 30 °C under irradiation) for 72 h, the reaction mixture was purified by flash chromatography on silica gel (eluted with PE:EtOAc = 1:1) to afford product deuterated Nikethamide **11** (24.7 mg, 0.138 mmol, 69% yield, 99% D) as a yellow oil.

$^1$H NMR (600 MHz, $CDCl_3$) δ 8.69 (s, 1.01H), 7.73 (d, *J* = 7.6 Hz, 1H), 7.37 (d, *J* = 7.3 Hz, 1H), 3.55 (d, *J* = 3.6 Hz, 2H), 3.26 (s, 2H), 1.28 – 1.10 (m, 6H).

$^{13}$C NMR (151 MHz, $CDCl_3$) δ 168.53, 150.00 (t, *J* = 27.1 Hz), 147.08, 134.41, 133.06, 123.32, 43.45, 39.58, 14.29, 12.85.

$^2$H NMR (92 MHz, $CH_2Cl_2$) δ 8.50 (s, 1D).

IR (film): ν (cm$^{-1}$) 2974, 2934, 2262, 1633, 1481, 1433, 1384, 1364, 1316, 1291, 1105, 1025, 868, 791, 745, 641.

HRMS (ESI-TOF, m/z) calcd for $C_{10}H_{14}DN_2O$ $(M+H)^+$: 180.1242, found: 180.1242.

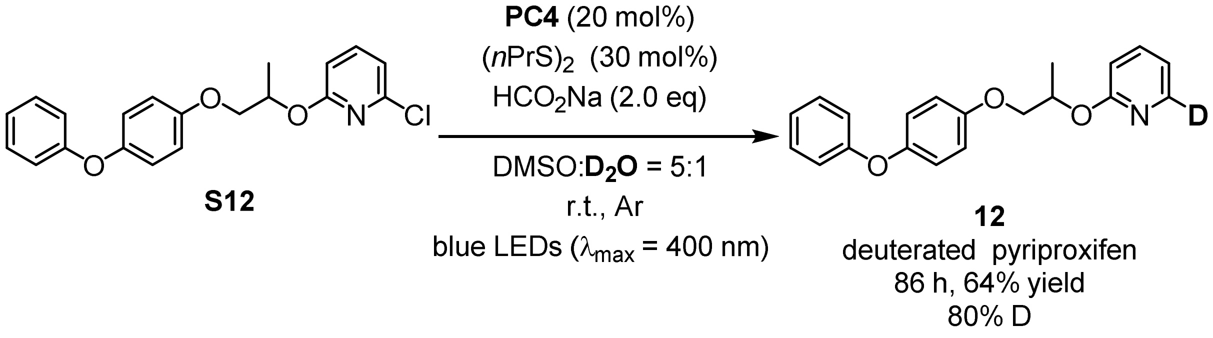


A dried 10 mL Schlenk tube was charged with **S12** (71.2 mg, 0.20 mmol), photocatalyst **PC4** (11.2 mg, 0.040 mmol), $(n\text{PrS})_2$ (10 μL, 0.060 mmol), $HCO_2Na$ (27.2 mg, 0.40 mmol), $D_2O$ (200 μL) and DMSO (1.0 mL) under air and then performed in a sealed vessel. The mixture was degassed *via* three freeze-pump-thaw cycles. The Schlenk tube was positioned approximately 3 cm away from a 50 W blue LEDs lamp ($\lambda_{max}$ = 400 nm). After being stirred at room temperature (~ 30 °C under irradiation) for 86 h, the reaction mixture was purified by flash chromatography on silica gel (eluted with PE:EtOAc = 20:1) to afford product deuterated Pyriproxifen **12** (41.3 mg, 0.128 mmol, 64% yield, 86% D) as a colorless oil.

$^1$H NMR (600 MHz, $CDCl_3$) δ 8.16 (dd, *J* = 5.0, 1.4 Hz, 0.14H), 7.58 (dd, *J* = 8.2, 7.2 Hz, 1H), 7.34 – 7.26 (m, 2H), 7.04 (t, *J* = 7.4 Hz, 1H), 7.02 – 6.89 (m, 6H), 6.87 (d, *J* = 7.0 Hz, 1H), 6.76 (d, *J* = 8.3 Hz, 1H), 5.70 – 5.49 (m, 1H), 4.20 (dd, *J* = 9.9, 5.3 Hz, 1H), 4.09 (dd, *J* = 9.9, 4.8 Hz, 1H), 1.50 (d, *J* = 6.4 Hz, 3H).

$^{13}$C NMR (151 MHz, $CDCl_3$) δ 163.14, 158.51, 155.24, 150.29, 146.39 (t, *J* = 27.5 Hz), 138.83, 129.63, 122.45, 120.79, 117.63, 116.66, 115.82, 111.76, 71.08, 69.41, 17.03.

$^2$H NMR (92 MHz, $CH_2Cl_2$) δ 8.01 (s, 1D).

IR (film): ν (cm$^{-1}$) 3062, 2979, 2933, 2873, 2258, 1589, 1505, 1489, 1440, 1282, 1224, 1074, 1046, 819, 692.

HRMS (ESI-TOF, m/z) calcd for $C_{20}H_{18}DNNaO_3$ $(M+Na)^+$: 345.1320, found: 345.1304.

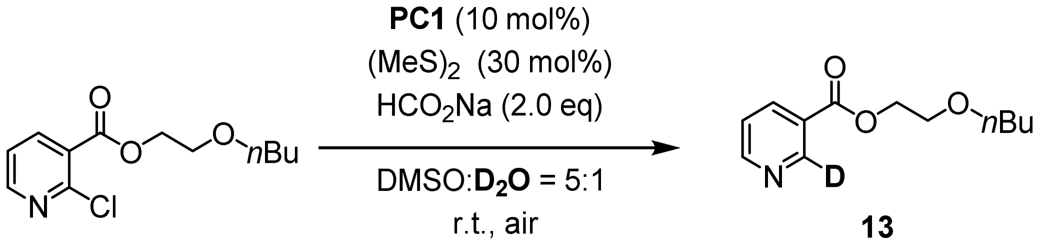

S13

13
deuterated nicoboxil
8 h, 86% yield
90% D

A dried 5 mL glass vial was charged with **S13** (51.4 mg, 0.20 mmol), photocatalyst **PC1** (8.7 mg, 0.020 mmol), $(MeS)_2$ (6 μL, 0.060 mmol), $HCO_2Na$ (27.2 mg, 0.40 mmol), $D_2O$ (200 μL) and DMSO (1.0 mL) under air and then performed in a sealed vessel. The glass vial was positioned approximately 3 cm away from a 50 W blue LEDs lamp ($\lambda_{max}$ = 400 nm). After being stirred at room temperature (~ 30 °C under irradiation) for 8 h, the reaction mixture was purified by flash chromatography on silica gel (eluted with PE:EtOAc = 10:1) to afford product deuterated Nicoboxil **13** (38.6 mg, 0.172 mmol, 86% yield, 90% D) as a colorless oil.

$^1$H NMR (600 MHz, $CDCl_3$) δ 9.19 (s, 0.1H), 8.72 (d, *J* = 4.5 Hz, 1H), 8.26 (d, *J* = 7.9 Hz, 1H), 7.34 (dd, *J* = 6.9, 5.2 Hz, 1H), 4.44 (dd, *J* = 4.7, 3.7 Hz, 2H), 3.71 (dd, *J* = 4.7, 3.7 Hz, 2H), 3.46 (dd, *J* = 9.5, 3.6 Hz, 2H), 1.57 – 1.46 (m, 2H), 1.40 – 1.25 (m, 2H), 0.96 – 0.76 (m, 3H).

$^{13}$C NMR (151 MHz, $CDCl_3$) δ 165.18, 153.32, 150.55 (t, *J* = 28.3 Hz), 137.16, 125.97, 123.29, 71.17, 68.39, 64.59, 31.61, 19.20, 13.83.

$^2$H NMR (92 MHz, $CH_2Cl_2$) δ 9.10 (s, 1D).

IR (film): ν (cm$^{-1}$) 3051, 2959, 2871, 2270, 1947, 1733, 1583, 1459, 1417, 1297, 1149, 908, 814, 735, 649.

HRMS (ESI-TOF, m/z) calcd for $C_{12}H_{17}DNO_3$ $(M+H)^+$: 225.1344, found: 225.1341.

## 6. Deuterodehalogenation of Halogen-Containing Bioactive Compounds and Drugs Derivatives

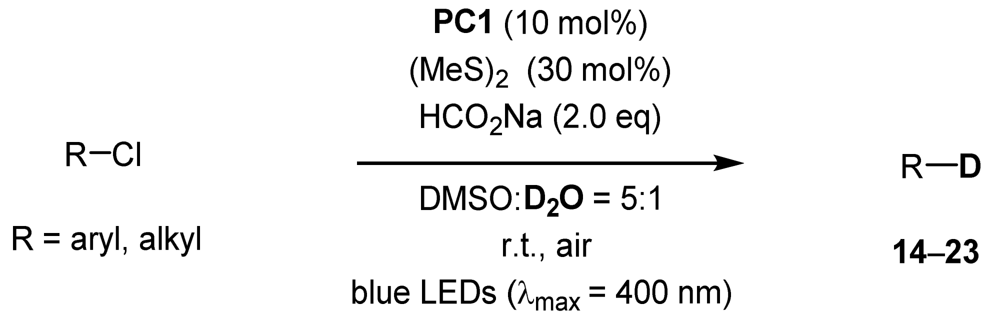


***N*-(4'-chloro-[1,1'-biphenyl]-2-yl)nicotinamide-2-D (14)**

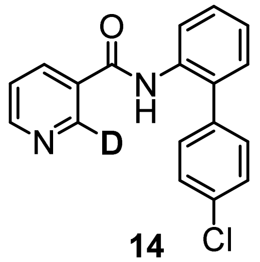


**14**
from boscalid

A dried 5 mL glass vial was charged with boscalid **S14** (68.6 mg, 0.20 mmol), photocatalyst **PC1** (8.7 mg, 0.020 mmol), $(MeS)_2$ (6 μL, 0.060 mmol), $HCO_2Na$ (27.2 mg, 0.40 mmol), $D_2O$ (200 μL) and DMSO (1.0 mL) under air and then performed in a sealed vessel. The glass vial was positioned approximately 3 cm away from a 50 W blue LEDs lamp ($\lambda_{max}$ = 400 nm). After being stirred at room temperature (~ 30 °C under irradiation) for 10 h, the reaction mixture was purified by flash chromatography on silica gel (eluted with PE:EtOAc = 1:2) to afford product **14** (55.3 mg, 0.178 mmol, 89% yield, 88% D) as a white solid.

$^1$H NMR (600 MHz, $CDCl_3$) δ 8.80 (s, 0.12H), 8.67 (dd, *J* = 4.7, 1.6 Hz, 1H), 8.27 (d, *J* = 7.5 Hz, 1H), 8.14 (s, 1H), 8.02 (d, *J* = 7.9 Hz, 1H), 7.43 (ddd, *J* = 10.8, 6.7, 2.1 Hz, 3H), 7.37 (ddd, *J* = 8.4, 7.3, 3.3 Hz, 3H), 7.32 – 7.26 (m, 2H).

$^{13}$C NMR (151 MHz, $CDCl_3$) δ 163.51, 152.31, 147.25 (t, *J* = 27.4 Hz), 136.45, 135.34, 134.37, 134.15, 132.43, 132.39, 130.55, 130.38, 130.28, 130.24, 129.41, 128.90, 125.52, 123.77, 122.82.

$^2$H NMR (92 MHz, $CH_2Cl_2$) δ 8.90 (s, 1D).

IR (film): ν ($cm^{-1}$) 3256, 3054, 2926, 2854, 1653, 1583, 1522, 1477, 1446, 1312, 1090, 1020, 915, 831, 760, 648.

HRMS (ESI-TOF, m/z) calcd for $C_{18}H_{13}DClN_2O$ $(M+H)^+$: 310.0852, found: 310.0848.

**(1*R*,2*S*,5*R*)-2-isopropyl-5-methylcyclohexyl benzoate-4-D (15)**

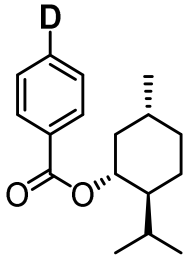


**15**
from L-Menthol derivative

A dried 5 mL glass vial was charged with L-Menthol derivative **S15** (59.0 mg, 0.20 mmol), photocatalyst **PC1** (8.7 mg, 0.020 mmol), $(MeS)_2$ (6 μL, 0.060 mmol), $HCO_2Na$ (27.2 mg, 0.40 mmol), $D_2O$ (200 μL) and DMSO (1.0 mL) under air and then performed in a sealed vessel. The glass vial was positioned approximately 3 cm away from a 50 W blue LEDs lamp ($\lambda_{max}$ = 400 nm). After being stirred at room temperature (~ 30 °C under irradiation) for 12 h, the reaction mixture was purified by flash chromatography on silica gel (eluted with PE:EtOAc = 10:1) to afford product **15** (43.4 mg, 0.170 mmol, 85% yield, 86% D) as a pale yellow oil.

$^1$H NMR (500 MHz, $CDCl_3$) δ 8.07 (t, *J* = 16.2 Hz, 2H), 7.55 (s, 0.14H), 7.51 – 7.38 (m, 2H), 4.94 (td, *J* = 10.9, 4.4 Hz, 1H), 2.17 – 2.10 (m, 1H), 1.97 (dtd, *J* = 14.0, 7.0, 2.7 Hz, 1H), 1.78 – 1.67 (m, 2H), 1.63 – 1.51 (m, 2H), 1.19 – 1.06 (m, 2H), 0.99 – 0.89 (m, 7H), 0.80 (d, *J* = 7.0 Hz, 3H).

$^{13}$C NMR (151 MHz, $CDCl_3$) δ 166.12, 132.41 (t, *J* = 19.2 Hz), 130.89, 129.56, 128.19, 74.84, 47.30, 40.99, 34.35, 31.46, 26.51, 23.65, 22.06, 20.79, 16.53.

$^2$H NMR (92 MHz, $CH_2Cl_2$) δ 7.44 (s, 1D).

IR (film): ν ($cm^{-1}$) 2956, 2928, 2870, 1716, 1456, 1370, 1274, 1174, 1115, 1025, 982, 963, 877.

HRMS (ESI-TOF, m/z) calcd for $C_{17}H_{23}DNaO_2$ $(M+Na)^+$: 284.1731, found: 284.1729.

**methyl 2-(4-(2-(benzamido-4-D)ethyl)phenoxy)-2-methylpropanoate (16)**

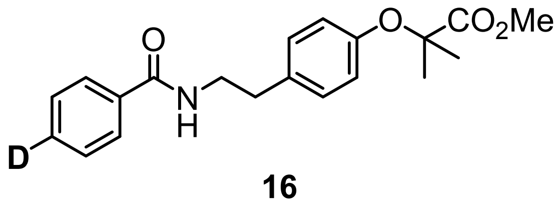

**16**
from bezafibrate derivative

A dried 5 mL glass vial was charged with bezafibrate derivative **S16** (75.2 mg, 0.20 mmol), photocatalyst **PC1** (8.7 mg, 0.020 mmol), $(MeS)_2$ (6 μL, 0.060 mmol), $HCO_2Na$ (27.2 mg, 0.40 mmol), $D_2O$ (200 μL) and DMSO (1.0 mL) under air and then performed in a sealed vessel. The glass vial was positioned approximately 3 cm away from a 50 W blue LEDs lamp ($\lambda_{max}$ = 400 nm). After being stirred at room temperature (~ 30 °C under irradiation) for 12 h, the reaction mixture was purified by flash chromatography on silica gel (eluted with PE:EtOAc = 3:1) to afford product **16** (47.9 mg, 0.140 mmol, 70% yield, 82% D) as a white solid.

$^1$H NMR (600 MHz, $CDCl_3$) δ 7.68 (d, *J* = 8.2 Hz, 2H), 7.43 (t, *J* = 7.4 Hz, 0.18H), 7.42 – 7.27 (m, 2H), 7.06 (d, *J* = 8.4 Hz, 2H), 6.76 (d, *J* = 8.5 Hz, 2H), 6.49 (s, 1H), 3.73 (s, 3H), 3.61 (dd, *J* = 13.1, 6.8 Hz, 2H), 2.82 (t, *J* = 7.1 Hz, 2H), 1.56 (s, 6H).

$^{13}$C NMR (151 MHz, $CDCl_3$) δ 174.84, 167.57, 153.93, 134.65, 132.77, 131.07 (t, *J* = 24.0 Hz), 129.55, 128.40, 126.88, 119.53, 79.15, 52.49, 41.24, 34.80, 25.35.

$^2$H NMR (92 MHz, $CH_2Cl_2$) δ 7.42 (s, 1D).

IR (film): ν ($cm^{-1}$) 3323, 2994, 2949, 1736, 1641, 1539, 1509, 1289, 1235, 1175, 1141, 964, 831.

HRMS (ESI-TOF, m/z) calcd for $C_{20}H_{23}DNO_4$ $(M+H)^+$: 343.1763, found: 343.1756.

**ethyl 4-(nicotinamido-6-D) butanoate (17)**

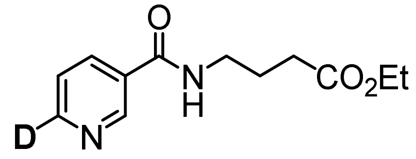


**17**

deuterated pikamilone ester

A dried 5 mL glass vial was charged with pikamilone ester **S17** (54.1 mg, 0.20 mmol), photocatalyst **PC1** (8.7 mg, 0.020 mmol), $(MeS)_2$ (6 μL, 0.060 mmol), $HCO_2Na$ (27.2 mg, 0.40 mmol), $D_2O$ (200 μL) and DMSO (1.0 mL) under air and then performed in a sealed vessel. The glass vial was positioned approximately 3 cm away from a 50 W blue LEDs lamp ($\lambda_{max}$ = 400 nm). After being stirred at room temperature (~ 30 °C under irradiation) for 48 h, the reaction mixture was purified by flash chromatography on silica gel (eluted with PE:EtOAc = 3:1) to afford product **17** (30.8 mg, 0.130 mmol, 65% yield, 89% D) as a white solid.

$^1$H NMR (500 MHz, $CDCl_3$) δ 8.98 (d, *J* = 1.8 Hz, 1H), 8.63 (dd, *J* = 4.8, 1.4 Hz, 0.11H), 8.11 (dd, *J* = 7.9, 2.2 Hz, 1H), 7.42 (s, 1H), 7.32 (d, *J* = 7.9 Hz, 1H), 4.07 (q, *J* = 7.1 Hz, 2H), 3.46 (dd, *J* = 12.4, 6.7 Hz, 2H), 2.40 (t, *J* = 7.0 Hz, 2H), 1.92 (p, *J* = 6.9 Hz, 2H), 1.19 (t, *J* = 7.1 Hz, 3H).

$^{13}$C NMR (126 MHz, $CDCl_3$) δ 173.89, 165.65, 151.53 (t, *J* = 24.5 Hz), 147.97, 135.26, 130.30, 123.34, 60.72, 39.83, 32.01, 24.23, 14.14.

$^2$H NMR (92 MHz, $CH_2Cl_2$) δ 8.94 (s, 1D).

IR (film): ν ($cm^{-1}$) 3354, 2921, 2851, 1731, 1648, 1592, 1544, 1458, 1180, 1026, 667.

HRMS (ESI-TOF, m/z) calcd for $C_{12}H_{16}DN_2O_3$ $(M+H)^+$: 238.1296, found: 238.1296.

**$N$-(3-chloro-4-((3-fluorobenzyl)oxy)phenyl)quinazolin-6-D-4-amine (18)**

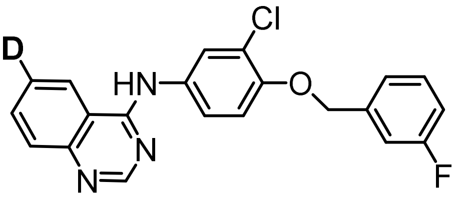


**18**
synthetic intermediate
of lapatinib ditosylate
**Y = I**

A dried 5 mL glass vial was charged with synthetic intermediate of lapatinib ditosylate **S18** (101.1 mg, 0.20 mmol), photocatalyst **PC1** (8.7 mg, 0.020 mmol), $(MeS)_2$ (6 μL, 0.060 mmol), $HCO_2Na$ (27.2 mg, 0.40 mmol), $D_2O$ (200 μL) and DMSO (1.0 mL) under air and then performed in a sealed vessel. The glass vial was positioned approximately 3 cm away from a 50 W blue LEDs lamp ($\lambda_{max}$ = 400 nm). After being stirred at room temperature (~ 30 °C under irradiation) for 12 h, the reaction mixture was purified by flash chromatography on silica gel (eluted with PE:EtOAc = 2:1) to afford product **18** (57.0 mg, 0.156 mmol, 78% yield, 73% D) as a white solid.

$^1$H NMR (600 MHz, $(CD_3)_2CO$) δ 9.14 (s, 1H), 8.65 (s, 1H), 8.38 (t, $J$ = 4.0 Hz, 1H), 8.16 (dd, $J$ = 7.2, 2.6 Hz, 1H), 7.86 – 7.78 (m, 3H), 7.60 – 7.57 (m, 0.27H), 7.46 (td, $J$ = 7.9, 6.0 Hz, 1H), 7.35 (dd, $J$ = 24.1, 8.8 Hz, 2H), 7.24 – 7.18 (m, 1H), 7.11 (td, $J$ = 8.5, 2.4 Hz, 1H), 5.27 (s, 2H).

$^{13}$C NMR (151 MHz, $(CD_3)_2CO$) δ 162.90 (d, $J$ = 244.1 Hz), 157.79, 154.51, 150.28 (d, $J$ = 1.9 Hz), 140.05 (d, $J$ = 7.4 Hz), 133.65, 132.64, 130.39 (d, $J$ = 8.3 Hz), 128.43, 126.04 (t, $J$ = 25.6 Hz), 124.04, 123.03 (d, $J$ = 2.8 Hz), 122.06, 121.82, 121.72, 121.61, 115.36, 114.51 (d, $J$ = 21.2 Hz), 114.21, 113.90 (d, $J$ = 22.3 Hz), 69.84 (d, $J$ = 1.7 Hz).

$^2$H NMR (92 MHz, Acetone) δ 7.87 (s, 1D).

IR (film): ν (cm$^{-1}$) 3297, 2925, 1592, 1572, 1526, 1489, 1415, 1261, 1215, 1060, 934, 784, 683.

HRMS (ESI-TOF, m/z) calcd for $C_{21}H_{14}DClFN_3NaO$ $(M+Na)^+$: 403.0843, found: 403.0829.

**3-(cyclopropylmethoxy)-4-(difluoromethoxy)-*N*-(pyridin-4-yl-3,5-$D_2$)benzamide (19)**

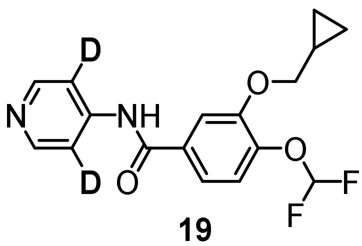

**19**
from roflumilast

A dried 5 mL glass vial was charged with roflumilast **S19** (80.6 mg, 0.20 mmol), photocatalyst **PC1** (8.7 mg, 0.020 mmol), $(MeS)_2$ (6 μL, 0.060 mmol), $HCO_2Na$ (27.2 mg, 0.40 mmol), $D_2O$ (200 μL) and DMSO (1.0 mL) under air and then performed in a sealed vessel. The glass vial was positioned approximately 3 cm away from a 50 W blue LEDs lamp ($\lambda_{max}$ = 400 nm). After being stirred at room temperature (~ 30 °C under irradiation) for 48 h, the reaction mixture was purified by flash chromatography on silica gel (eluted with PE:EtOAc = 1:1) to afford product **19** (41.0 mg, 0.122 mmol, 61% yield, 72% D) as a yellow oil.

$^1$H NMR (600 MHz, $CDCl_3$) δ 9.44 (d, *J* = 24.3 Hz, 1H), 8.42 (s, 2H), 7.67 (t, *J* = 6.1 Hz, 0.56H), 7.48 (s, 1H), 7.38 (dd, *J* = 8.3, 1.7 Hz, 1H), 7.10 (dd, *J* = 8.2, 2.0 Hz, 1H), 6.67 (t, *J* = 74.9 Hz, 1H), 3.82 (dd, *J* = 6.9, 2.2 Hz, 2H), 1.30 – 1.18 (m, 1H), 0.61 (q, *J* = 5.3 Hz, 2H), 0.29 (q, *J* = 4.7 Hz, 2H).

$^{13}$C NMR (151 MHz, $CDCl_3$) δ 165.93, 150.59, 150.11, 150.04, 145.92, 145.86, 143.32 (t, *J* = 2.9 Hz), 132.24, 121.84, 119.76, 115.83 (t, *J* = 260.6 Hz), 113.97, 113.90, 74.09, 9.95, 3.24.

$^2$H NMR (92 MHz, $CH_2Cl_2$) δ 7.74 (s, 2D).

IR (film): ν (cm$^{-1}$) 3249, 1685, 1583, 1499, 1407, 1312, 1200, 1053, 1006, 836, 762.

HRMS (ESI-TOF, m/z) calcd for $C_{17}H_{15}D_2F_2N_2O_3$ $(M+H)^+$: 337.1327, found: 337.1320.

**(*R*)-2,5,7,8-tetramethyl-2-((4*R*,8*R*)-4,8,12-trimethyltridecyl)chroman-6-yl nicotinate-6-D (20)**

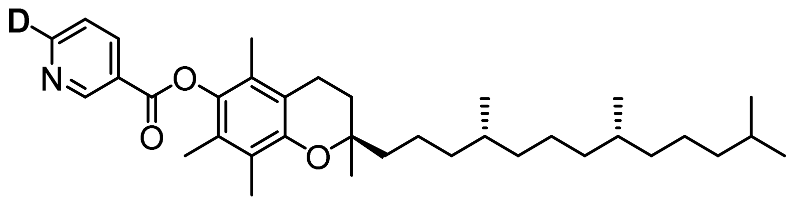

**20**
from Vitamin E derivative

A dried 5 mL glass vial was charged with Vitamin E derivative **S20** (114.1 mg, 0.20 mmol), photocatalyst **PC1** (8.7 mg, 0.020 mmol), $(MeS)_2$ (6 μL, 0.060 mmol), $HCO_2Na$ (27.2 mg, 0.40 mmol), $D_2O$ (200 μL) and DMSO (1.0 mL) under air and then performed in a sealed vessel. The glass vial was positioned approximately 3 cm away from a 50 W blue LEDs lamp ($\lambda_{max}$ = 400 nm). After being stirred at room temperature (~ 30 °C under irradiation) for 48 h, the reaction mixture was purified by flash chromatography on silica gel (eluted with PE:EtOAc = 10:1) to afford product **20** (81.6 mg, 0.152 mmol, 76% yield, 83% D) as a yellow oil.

$^1H$ NMR (500 MHz, $CDCl_3$) δ 9.44 (d, *J* = 20.3 Hz, 1H), 8.86 (d, *J* = 4.2 Hz, 0.17H), 8.50 (d, *J* = 7.9 Hz, 1H), 7.48 (d, *J* = 7.9 Hz, 1H), 2.63 (t, *J* = 6.7 Hz, 2H), 2.14 (s, 3H), 2.07 (s, 3H), 2.03 (s, 2H), 1.64 – 1.48 (m, 4H), 1.46 – 1.22 (m, 16H), 1.18 – 1.01 (m, 7H), 0.91 – 0.83 (m, 12H).

$^{13}C$ NMR (151 MHz, $CDCl_3$) δ 163.89, 153.80, 151.30, 149.73, 140.32, 137.70, 126.73, 125.70, 125.00, 123.43, 123.32, 117.62, 75.20, 40.47, 39.61, 39.40, 37.47, 37.31, 32.82, 31.21, 31.01, 28.00, 24.83, 24.37, 23.69, 22.75, 22.65, 21.05, 20.66, 19.78, 19.69, 13.09, 12.25, 11.89.

$^2H$ NMR (92 MHz, $CH_2Cl_2$) δ 8.86 (s, 1D).

IR (film): ν ($cm^{-1}$) 2927, 2868, 2259, 1743, 1590, 1463, 1418, 1378, 1334, 1277, 1239, 1159, 1099, 1022, 734, 636.

HRMS (ESI-TOF, m/z) calcd for $C_{35}H_{52}DNNaO_3$ $(M+Na)^+$: 559.3980, found: 559.3975.

**(2*R*,3*S*,4*R*,5*R*)-2-(hydroxymethyl)-5-(9H-purin-9-yl-6-D)tetrahydrofuran-3,4-diol (21)**

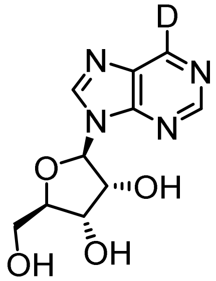

**21**

from 6-Chloropurine riboside

A dried 5 mL glass vial was charged with 6-Chloropurine riboside **S21** (57.3 mg, 0.20 mmol), photocatalyst **PC1** (8.7 mg, 0.020 mmol), $(MeS)_2$ (6 μL, 0.060 mmol), $HCO_2Na$ (27.2 mg, 0.40 mmol), $D_2O$ (200 μL) and DMSO (1.0 mL) under air and then performed in a sealed vessel. The glass vial was positioned approximately 3 cm away from a 50 W blue LEDs lamp ($\lambda_{max}$ = 400 nm). After being stirred at room temperature (~ 30 °C under irradiation) for 72 h, the reaction mixture was purified by flash chromatography on silica gel (eluted with $CH_2Cl_2$:MeOH = 4:1) to afford product **21** (31.8 mg, 0.126 mmol, 63% yield, 83% D) as a white solid.

$^1$H NMR (600 MHz, $D_2O$) δ 9.06 (s, 1H), 8.89 (s, 0.17H), 8.69 (s, 1H), 6.18 (d, *J* = 5.6 Hz, 1H), 4.84 (t, *J* = 5.4 Hz, 1H), 4.51 – 4.44 (m, 1H), 4.31 (dd, *J* = 7.0, 3.8 Hz, 1H), 3.95 (dd, *J* = 12.8, 2.9 Hz, 1H), 3.88 (dd, *J* = 12.8, 4.0 Hz, 1H).

$^{13}$C NMR (151 MHz, $D_2O$) δ 151.79, 150.46, 147.65 (t, *J* = 28.4 Hz), 145.74, 133.56, 88.32, 85.57, 73.76, 70.38, 61.30.

$^2$H NMR (92 MHz, $H_2O$) δ 9.00 (s, 1D).

IR (film): ν (cm$^{-1}$) 3359, 3192, 2922, 2851, 1660, 1633, 1468, 1410, 1377, 1260, 1082, 965, 712.

HRMS (ESI-TOF, m/z) calcd for $C_{10}H_{12}DN_4O_4$ $(M+H)^+$: 254.0994, found: 254.0996.

**((3a*R*,4*R*,6*R*,6a*R*)-2,2-dimethyl-6-(9H-purin-9-yl-6-d)tetrahydrofuro[3,4-d][1,3]dioxol-4-yl)methanol (22)**

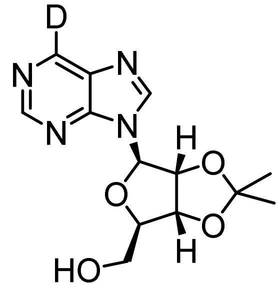

**22**

from nucleoside analog

A dried 5 mL glass vial was charged with nucleoside analog **S22** (65.3 mg, 0.20 mmol), photocatalyst **PC1** (8.7 mg, 0.020 mmol), $(MeS)_2$ (6 μL, 0.060 mmol), $HCO_2Na$ (27.2 mg, 0.40 mmol), $D_2O$ (200 μL) and DMSO (1.0 mL) under air and then performed in a sealed vessel. The glass vial was positioned approximately 3 cm away from a 50 W blue LEDs lamp ($\lambda_{max}$ = 400 nm). After being stirred at room temperature (~ 30 °C under irradiation) for 15 h, the reaction mixture was purified by flash chromatography on silica gel (eluted with PE:EtOAc = 10:1) to afford product **22** (31.1 mg, 0.106 mmol, 53% yield, 85% D) as a pale yellow oil.

$^1$H NMR (600 MHz, $CDCl_3$) δ 9.12 (s, 0.15H), 8.91 (s, 1H), 8.23 (s, 1H), 5.99 (d, *J* = 4.4 Hz, 1H), 5.25 – 5.13 (m, 1H), 5.11 – 4.99 (m, 1H), 4.50 (d, *J* = 1.1 Hz, 1H), 3.92 (dd, *J* = 12.6, 1.6 Hz, 1H), 3.78 (dd, *J* = 12.6, 2.2 Hz, 1H), 2.56 (s, 1H), 1.60 (s, 3H), 1.34 (s, 3H).

$^{13}$C NMR (151 MHz, $CDCl_3$) δ 152.12, 150.21, 149.12 (t, *J* = 27.2 Hz), 144.97, 135.29, 114.31, 93.56, 86.35, 83.26, 81.55, 63.09, 27.51, 25.21.

$^2$H NMR (92 MHz, $CH_2Cl_2$) δ 9.00 (s, 1D).

IR (film): ν (cm$^{-1}$) 3363, 2989, 2935, 1586, 1496, 1384, 1334, 1214, 1081, 850, 645, 565.

HRMS (ESI-TOF, m/z) calcd for $C_{13}H_{15}DN_4NaO_4$ $(M+Na)^+$: 316.1127, found: 316.1125.

**(3a*S*,5*S*,6a*S*)-5-((*S*)-2,2-dimethyl-1,3-dioxolan-4-yl)-2,2-dimethyltetrahydrofuro[2,3-d][1,3]dioxol-6-yl 4-(methyl-d)benzoate (23)**

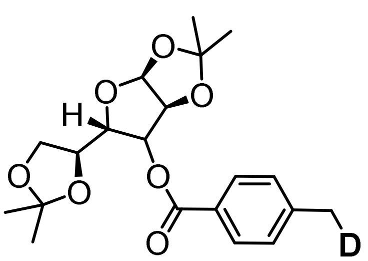

**23**

from diacetone-d-glucose derivative

A dried 5 mL glass vial was charged with diacetone-d-glucose derivative **S23** (82.6 mg, 0.20 mmol), photocatalyst **PC1** (8.7 mg, 0.020 mmol), $(MeS)_2$ (6 μL, 0.060 mmol), $HCO_2Na$ (27.2 mg, 0.40 mmol), $D_2O$ (200 μL) and DMSO (1.0 mL) under air and then performed in a sealed vessel. The glass vial was positioned approximately 3 cm away from a 50 W blue LEDs lamp ($\lambda_{max}$ = 400 nm). After being stirred at room temperature (~ 30 °C under irradiation) for 10 h, the reaction mixture was purified by flash chromatography on silica gel (eluted with PE:EtOAc = 10:1) to afford product **23** (55.8 mg, 0.130 mmol, 65% yield, 94% D) as a colorless oil.

$^1$H NMR (600 MHz, $CDCl_3$) δ 7.90 (d, *J* = 8.2 Hz, 2H), 7.24 (d, *J* = 8.1 Hz, 2H), 5.94 (d, *J* = 3.6 Hz, 1H), 5.48 (d, *J* = 2.7 Hz, 1H), 4.62 (d, *J* = 3.6 Hz, 1H), 4.39 – 4.30 (m, 2H), 4.09 (qd, *J* = 8.6, 5.4 Hz, 2H), 2.40 (d, *J* = 9.2 Hz, 2.06H), 1.55 (s, 3H), 1.41 (s, 3H), 1.31 (s, 3H), 1.26 (s, 3H).

$^{13}$C NMR (151 MHz, $CDCl_3$) δ 165.27, 144.28, 129.75, 129.26, 126.78, 112.34, 109.36, 105.15, 83.42, 79.99, 76.44, 72.62, 67.21, 26.82, 26.76, 26.22, 25.22, 21.45 (t, *J* = 19.2 Hz).

$^2$H NMR (92 MHz, $CH_2Cl_2$) δ 2.56 (s, 1D).

IR (film): ν (cm$^{-1}$) 2989, 2937, 1727, 1613, 1456, 1374, 1268, 1217, 1097, 1076, 1021, 846, 745.

HRMS (ESI-TOF, m/z) calcd for $C_{20}H_{25}DNaO_7$ $(M+Na)^+$: 402.1634, found: 402.1634.

## 7. Application of the Methodology in Synthesis of Deuterated Drugs *via* H/D Exchange

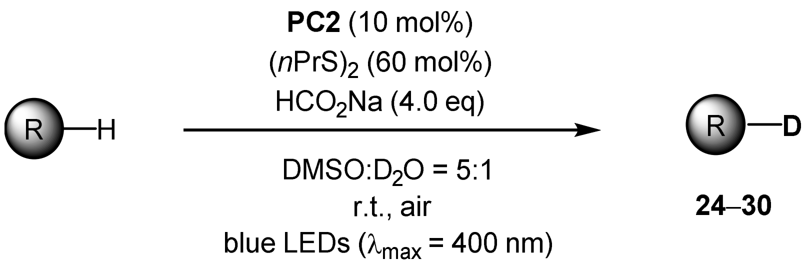


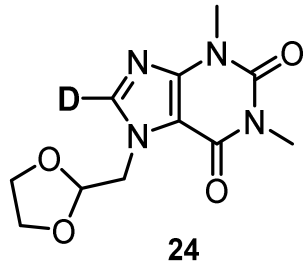


**24**
deuterated Doxofylline

A dried 5 mL glass vial was charged with Doxofylline (53.3 mg, 0.20 mmol), photocatalyst **PC2** (16.2 mg, 0.020 mmol), $(n\text{PrS})_2$ (20 μL, 0.060 mmol), $HCO_2Na$ (54.4 mg, 0.80 mmol), $D_2O$ (400 μL) and DMSO (2.0 mL) under air and then performed in a sealed vessel. The glass vial was positioned approximately 3 cm away from a 50 W blue LEDs lamp ($\lambda_{max}$ = 400 nm). After being stirred at room temperature (~ 30 °C under irradiation) for 56 h, the reaction mixture was purified by flash chromatography on silica gel (eluted with PE:EtOAc = 1:2) to afford product **24** (45.4 mg, 0.170 mmol, 85% yield, 93% D) as a white solid.

$^1$H NMR (500 MHz, $CDCl_3$) δ 7.61 (s, 0.07H), 5.17 (t, $J$ = 3.1 Hz, 1H), 4.53 (d, $J$ = 3.1 Hz, 2H), 3.88 – 3.74 (m, 4H), 3.54 (s, 3H), 3.35 (s, 3H).

$^{13}$C NMR (151 MHz, $CDCl_3$) δ 155.29, 151.63, 148.32, 142.02 (t, $J$ = 31.8 Hz), 107.27, 100.82, 65.42, 47.91, 29.74, 27.93.

$^2$H NMR (92 MHz, $CH_2Cl_2$) δ 8.09 (s, 1D).

IR (film): ν ($cm^{-1}$) 3110, 2953, 2897, 1704, 1604, 1549, 1477, 1435, 1406, 1375, 1190, 973, 892.

HRMS (ESI-TOF, m/z) calcd for $C_{11}H_{14}DN_4O_4$ $(M+H)^+$: 268.1151, found: 268.1172.

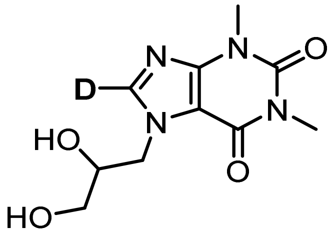

**25**
deuterated Diprophylline

A dried 5 mL glass vial was charged with Diprophylline (50.8 mg, 0.20 mmol), photocatalyst **PC2** (16.2 mg, 0.020 mmol), $(n\mathrm{PrS})_2$ (20 μL, 0.060 mmol), $HCO_2Na$ (54.4 mg, 0.80 mmol), $D_2O$ (400 μL) and DMSO (2.0 mL) under air and then performed in a sealed vessel. The glass vial was positioned approximately 3 cm away from a 50 W blue LEDs lamp ($\lambda_{max}$ = 400 nm). After being stirred at room temperature (~ 30 °C under irradiation) for 24 h, the reaction mixture was purified by flash chromatography on silica gel (eluted with EtOAc) to afford product **25** (31.7 mg, 0.124 mmol, 62% yield, 90% D) as a white solid.

$^1$H NMR (600 MHz, $(CD_3)_2SO$) δ 7.94 (s, 0.1H), 5.01 (s, 1H), 4.77 (s, 1H), 4.40 (dd, $J$ = 13.6, 3.6 Hz, 1H), 4.06 (dd, $J$ = 13.6, 8.7 Hz, 1H), 3.79 (d, $J$ = 3.9 Hz, 1H), 3.41 (s, 3H), 3.35 (d, $J$ = 4.8 Hz, 2H), 3.20 (s, 3H).

$^{13}$C NMR (151 MHz, $(CD_3)_2SO$) δ 154.97, 151.46, 148.75, 143.62 (t, $J$ = 30.3 Hz), 106.51, 70.39, 63.96, 50.02, 29.88, 28.01.

$^2$H NMR (92 MHz, DMSO) δ 7.97 (s, 1D).

HRMS (ESI-TOF, m/z) calcd for $C_{10}H_{14}DN_4O_4$ $(M+H)^+$: 256.1151, found: 256.1147.

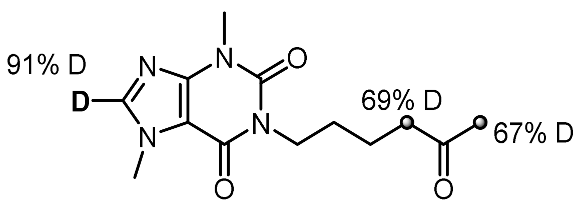


**26**

deuterated Pentoxifylline

A dried 5 mL glass vial was charged with Pentoxifylline (50.8 mg, 0.20 mmol), photocatalyst **PC2** (16.2 mg, 0.020 mmol), ($n$PrS)$_2$ (20 μL, 0.060 mmol), $HCO_2Na$ (54.4 mg, 0.80 mmol), $D_2O$ (400 μL) and DMSO (2.0 mL) under air and then performed in a sealed vessel. The glass vial was positioned approximately 3 cm away from a 50 W blue LEDs lamp ($\lambda_{max}$ = 400 nm). After being stirred at room temperature (~ 30 °C under irradiation) for 48 h, the reaction mixture was purified by flash chromatography on silica gel (eluted with PE:EtOAc = 1:2) to afford product **26** (20.1 mg, 0.070 mmol, 35% yield) as a white solid.

$^1$H NMR (600 MHz, $CDCl_3$) δ 7.49 (s, 0.09H, 91% D), 3.98 (t, 2H), 3.96 (s, 3H), 3.54 (s, 3H), 2.49 – 2.43 (m, 0.62H, 69% D), 2.13 – 2.07 (m, 0.99H, 67% D), 1.66 – 1.57 (m, 4H).

$^{13}$C NMR (151 MHz, $CDCl_3$) δ 209.32 – 208.67 (m), 155.27, 151.46, 148.76, 141.33 (t, $J$ = 37.7 Hz), 107.61, 43.29 – 42.06 (m), 40.80, 33.56, 29.95, 29.75 – 29.00 (m), 27.39, 21.00 – 20.78 (m).

IR (film): ν (cm$^{-1}$) 3114, 2951, 1652, 1604, 1549, 1455, 1358, 1235, 1052, 969, 763.

HRMS (ESI-TOF, m/z) calcd for $C_{13}H_{18}DN_4O_3$ $(M+H)^+$: 285.1828, found: 285.1826.

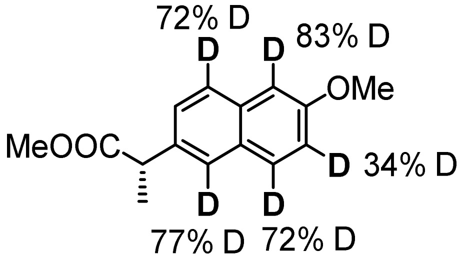


**27**
deuterated Naproxen ester

A dried 5 mL glass vial was charged with Naproxen ester **S27** (48.8 mg, 0.20 mmol), photocatalyst **PC2** (16.2 mg, 0.020 mmol), ($n$PrS)$_2$ (20 μL, 0.060 mmol), $HCO_2Na$ (54.4 mg, 0.80 mmol), $D_2O$ (400 μL) and DMSO (2.0 mL) under air and then performed in a sealed vessel. The glass vial was positioned approximately 3 cm away from a 50 W blue LEDs lamp ($\lambda_{max}$ = 400 nm). After being stirred at room temperature (~ 30 °C under irradiation) for 48 h, the reaction mixture was purified by flash chromatography on silica gel (eluted with PE:EtOAc = 10:1) to afford product **27** (20.9 mg, 0.084 mmol, 42% yield) as a white solid.

$^1$H NMR (500 MHz, $CDCl_3$) δ 7.71 (d, $J$ = 8.3 Hz, 0.56H), 7.67 (s, 0.23H), 7.41 (s, 1H), 7.14 (s, 0.66H), 7.12 (s, 0.17H), 3.91 (s, 3H), 3.87 (q, $J$ = 7.1 Hz, 1H), 3.67 (s, 3H), 1.58 (d, $J$ = 7.2 Hz, 3H).

$^{13}$C NMR (151 MHz, $CDCl_3$) δ 175.18, 157.60 (t, $J$ = 5.2 Hz), 135.59 (t, $J$ = 11.3 Hz), 134.12 – 133.50 (m), 129.50 – 129.04 (m), 128.98 – 128.50 (m), 127.24 – 126.61 (m), 126.20 – 125.74 (m), 125.90 – 125.25 (m), 118.97 (d, $J$ = 17.7 Hz), 105.59 – 105.06 (m), 55.33, 52.07, 45.32, 18.61.

IR (film): ν (cm$^{-1}$) 2974, 1739, 1592, 1440, 1331, 1262, 1200, 1175, 1093, 851.

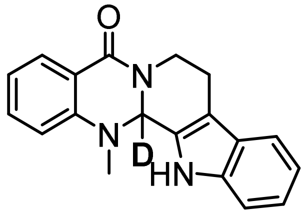

**28**
deuterated Evodiamine

A dried 5 mL glass vial was charged with Evodiamine (48.8 mg, 0.20 mmol), photocatalyst **PC2** (16.2 mg, 0.020 mmol), ($n$PrS)$_2$ (20 μL, 0.060 mmol), $HCO_2Na$ (54.4 mg, 0.80 mmol), $D_2O$ (400 μL) and DMSO (2.0 mL) under air and then performed in a sealed vessel. The glass vial was positioned approximately 3 cm away from a 50 W blue LEDs lamp ($\lambda_{max}$ = 400 nm). After being stirred at room temperature (~ 30 °C under irradiation) for 24 h, the reaction mixture was purified by flash chromatography on silica gel (eluted with PE:EtOAc = 3:1) to afford product **28** (21.9 mg, 0.072 mmol, 36% yield, 75% D) as a yellow solid.

$^1$H NMR (600 MHz, $(CD_3)_2SO$) δ 11.07 (d, $J$ = 6.3 Hz, 1H), 7.80 (d, $J$ = 7.5 Hz, 1H), 7.48 (d, $J$ = 7.1 Hz, 2H), 7.37 (d, $J$ = 8.1 Hz, 1H), 7.20 – 6.91 (m, 4H), 6.13 (s, 0.25H, 75% D), 4.64 (dd, $J$ = 12.4, 4.4 Hz, 1H), 3.27 – 3.15 (m, 1H), 3.02 – 2.84 (m, 4H), 2.80 (d, $J$ = 15.2 Hz, 1H).

$^{13}$C NMR (151 MHz, $(CD_3)_2SO$) δ 164.74, 149.19, 136.94, 133.94, 131.11, 128.46, 126.44, 122.32, 120.68, 119.64, 119.37, 118.68, 117.85, 112.14, 111.98, 69.85 (t, $J$ = 22.2 Hz), 41.39, 36.90, 19.94.

IR (film): ν (cm$^{-1}$) 2920, 2847, 1626, 1506, 1456, 1393, 1302, 1197, 1137, 1125, 1097,745.

HRMS (ESI-TOF, m/z) calcd for $C_{19}H_{17}DN_3O$ $(M+H)^+$: 305.1507, found: 305.1505.

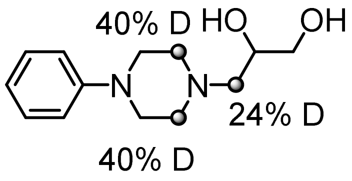


**29**

deuterated Dropropizine

A dried 5 mL glass vial was charged with Dropropizine (47.3 mg, 0.20 mmol), photocatalyst **PC2** (16.2 mg, 0.020 mmol), (*n*PrS)$_2$ (20 μL, 0.060 mmol), $HCO_2Na$ (54.4 mg, 0.80 mmol), $D_2O$ (400 μL) and DMSO (2.0 mL) under air and then performed in a sealed vessel. The glass vial was positioned approximately 3 cm away from a 50 W blue LEDs lamp ($\lambda_{max}$ = 400 nm). After being stirred at room temperature (~ 30 °C under irradiation) for 56 h, the reaction mixture was purified by flash chromatography on silica gel (eluted with EtOAc) to afford product **29** (28.3 mg, 0.116 mmol, 58% yield) as a white solid.

$^1$H NMR (600 MHz, $CDCl_3$) δ 7.26 – 7.21 (m, 2H), 6.90 (d, *J* = 7.9 Hz, 2H), 6.85 (t, *J* = 7.2 Hz, 1H), 3.86 (dd, *J* = 8.5, 4.0 Hz, 1H), 3.71 (dd, *J* = 11.4, 3.2 Hz, 1H), 3.60 – 3.48 (m, 3H), 3.16 (dd, *J* = 28.6, 14.2 Hz, 2H), 2.76 (ddd, *J* = 18.5, 12.1, 6.1 Hz, 1.2H), 2.58 (dt, *J* = 18.8, 11.9 Hz, 2H), 2.39 (dd, *J* = 12.5, 3.4 Hz, 0.76H).

$^{13}$C NMR (151 MHz, $CDCl_3$) δ 151.12, 129.19, 120.25 – 119.70 (m), 116.50 – 115.95 (m), 67.25, 65.00, 60.49, 53.54 – 52.86 (m), 49.33 – 48.35 (m).

HRMS (ESI-TOF, m/z) calcd for $C_{13}H_{15}D_6N_2O_2$ $(M+H)^+$: 243.1974, found: 243.1976.

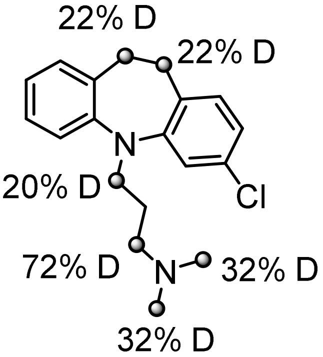


**30**
deuterated Clomipramine

A dried 5 mL glass vial was charged with Clomipramine (63.0 mg, 0.20 mmol), photocatalyst **PC2** (16.2 mg, 0.020 mmol), ($n$PrS)$_2$ (20 μL, 0.060 mmol), $HCO_2Na$ (54.4 mg, 0.80 mmol), $D_2O$ (400 μL) and DMSO (2.0 mL) under air and then performed in a sealed vessel. The glass vial was positioned approximately 3 cm away from a 50 W blue LEDs lamp ($\lambda_{max}$ = 400 nm). After being stirred at room temperature (~ 30 °C under irradiation) for 24 h, the reaction mixture was purified by flash chromatography on silica gel (eluted with PE:EtOAc = 1:3) to afford product **30** (41.4 mg, 0.126 mmol, 63% yield) as a colorless oil.

$^1$H NMR (600 MHz, $CDCl_3$) δ 7.12 (ddd, $J$ = 24.9, 15.1, 7.3 Hz, 4H), 6.98 (dd, $J$ = 13.3, 7.5 Hz, 2H), 6.87 (d, $J$ = 8.0 Hz, 1H), 3.75 (dd, $J$ = 8.5, 4.4 Hz, 1.6H), 3.14 (dd, $J$ = 23.6, 13.1 Hz, 3.56H), 2.28 (dd, $J$ = 16.6, 10.1 Hz, 0.56H), 2.14 (t, $J$ = 14.6 Hz, 4.11H), 1.71 (t, $J$ = 6.7 Hz, 2H).

$^{13}$C NMR (151 MHz, $CDCl_3$) δ 149.12, 147.92, 135.13, 135.09, 131.55, 131.20, 129.45, 126.59, 123.27, 122.00, 120.60, 119.91, 57.56 – 56.51 (m), 49.19 – 47.99 (m), 45.61 – 44.63 (m), 32.35 – 31.64 (m), 31.62 – 30.75 (m), 26.19 – 24.60 (m).

IR (film): ν (cm$^{-1}$) 3063, 2940, 2838, 2791, 2176, 2053, 1590, 1484, 1456, 1406, 1227, 1100, 927, 747.

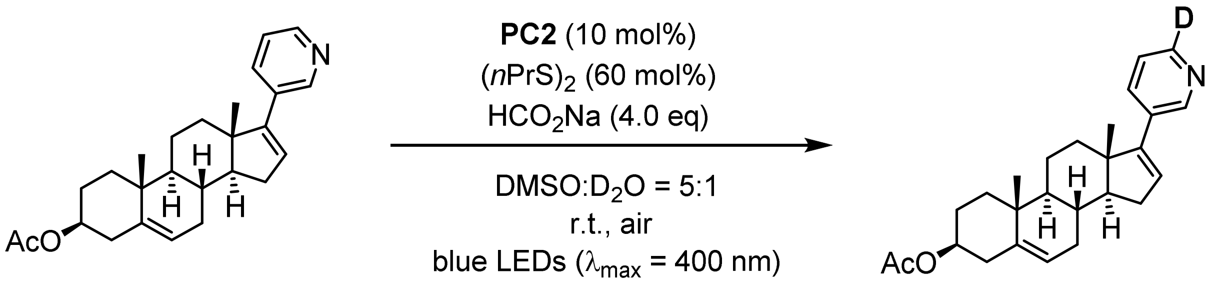


**Zytiga**

**7**
**deuterated Zytiga**
0% yield

A dried 5 mL glass vial was charged with Zytiga (63.0 mg, 0.20 mmol), photocatalyst **PC2** (16.2 mg, 0.020 mmol), ($n$PrS)$_2$ (20 μL, 0.060 mmol), $HCO_2Na$ (54.4 mg, 0.80 mmol), $D_2O$ (400 μL) and DMSO (2.0 mL) under air and then performed in a sealed vessel. The glass vial was positioned approximately 3 cm away from a 50 W blue LEDs lamp ($\lambda_{max}$ = 400 nm). After being stirred at room temperature (~ 30 °C under irradiation) for 24 h, compound **7** was not formed (detected by $^1$H NMR analysis).

# 8. $^1$H, $^2$H and $^{13}$C NMR Spectra

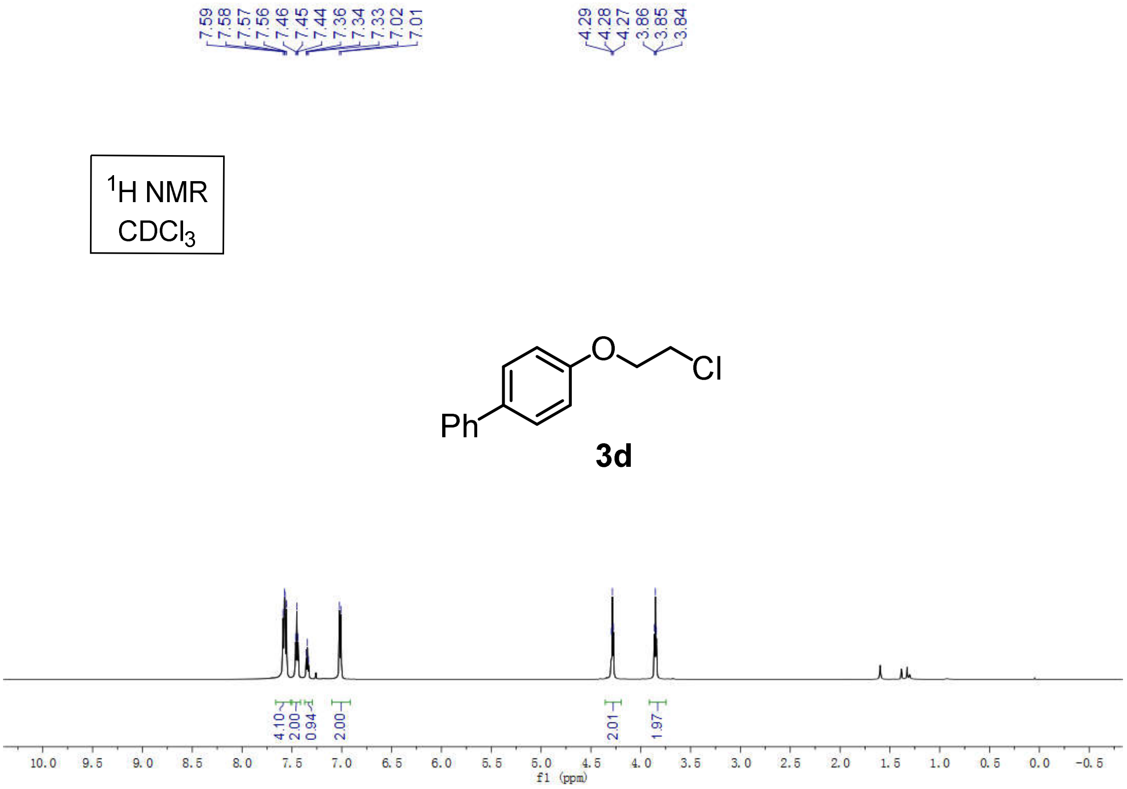


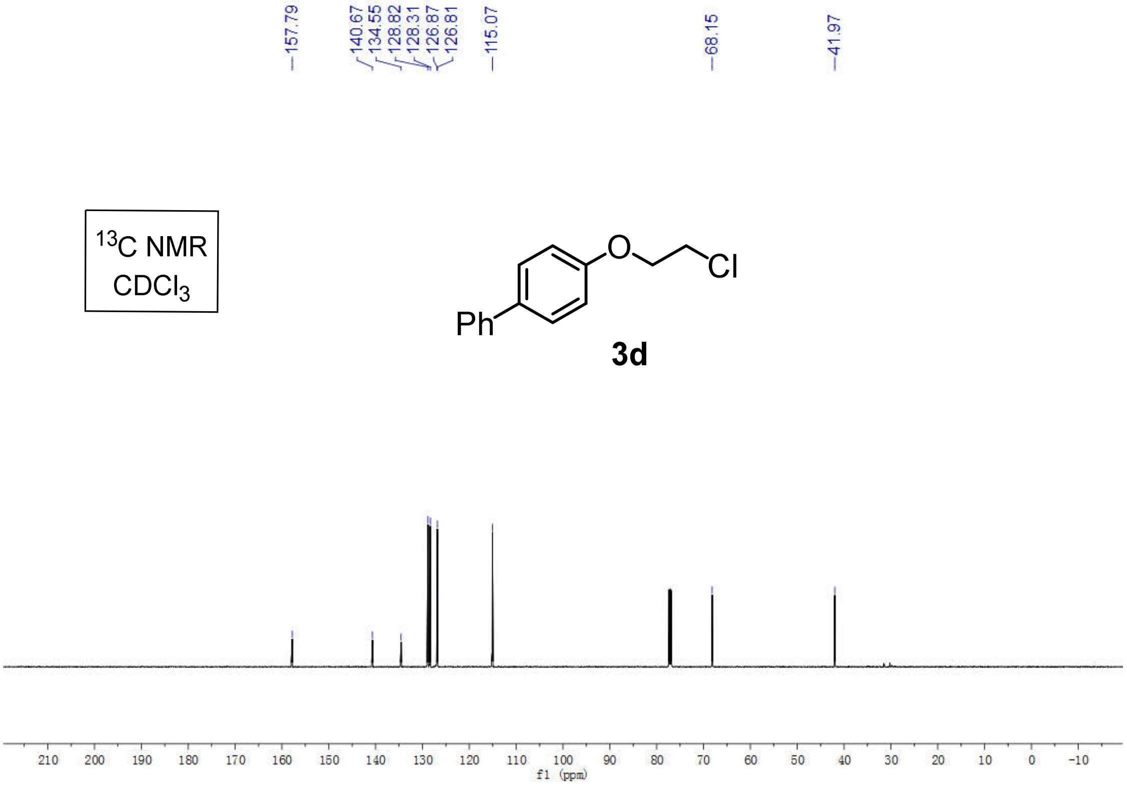


**Supplementary Fig. 27** $^1$H NMR and $^{13}$C NMR spectra of the compound **3d**.

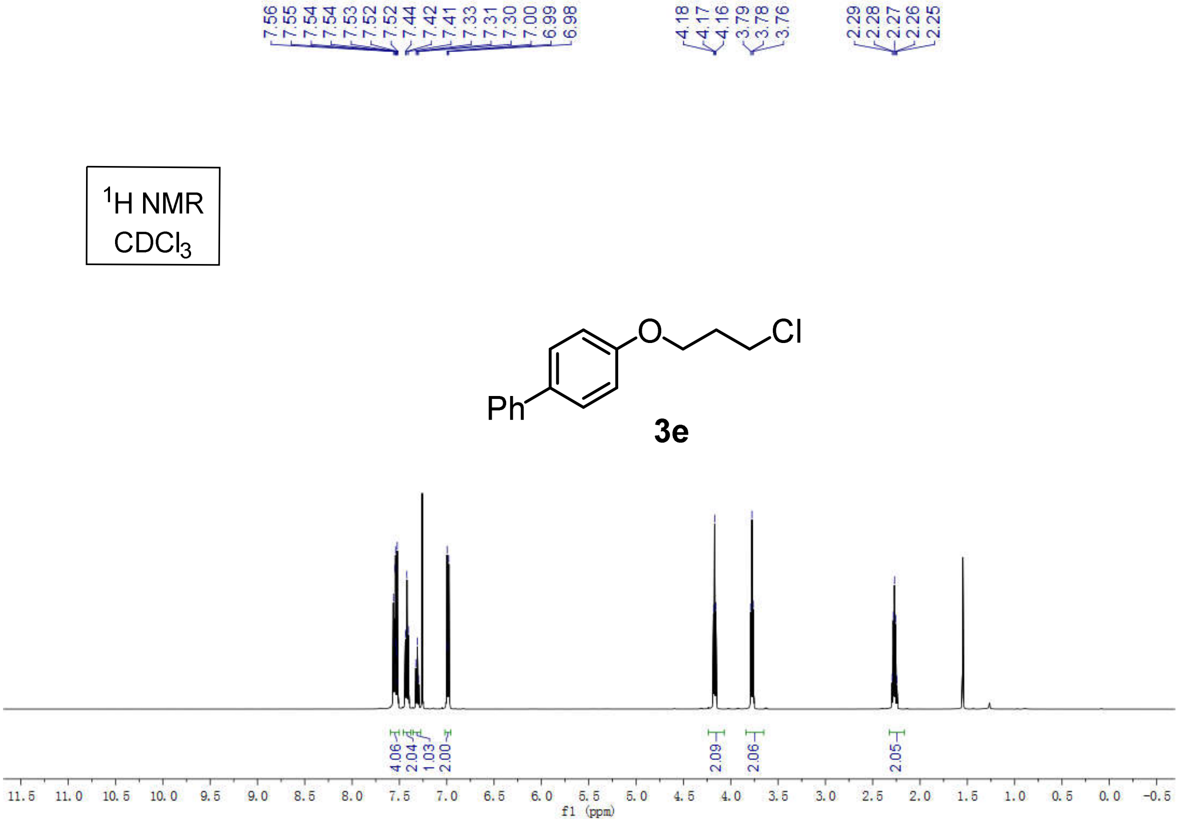


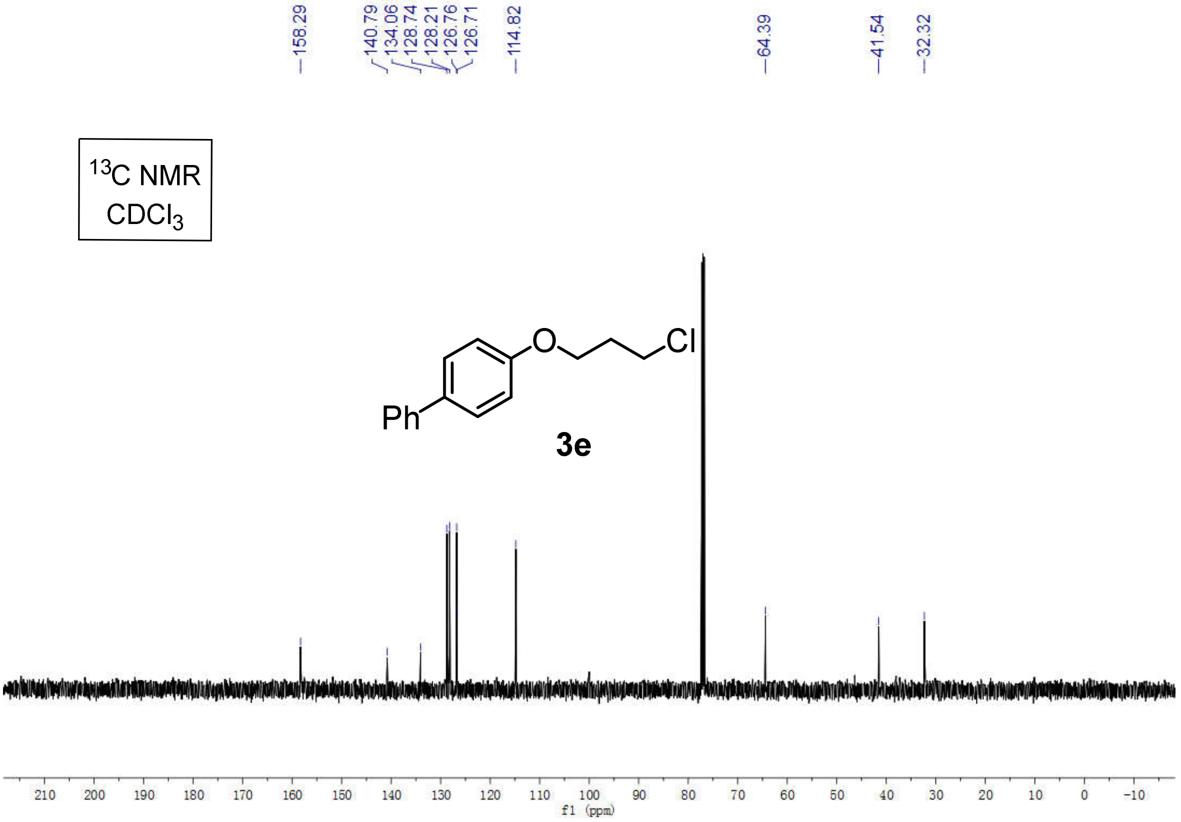


**Supplementary Fig. 28** $^{1}$H NMR and $^{13}$C NMR spectra of the compound **3e**.

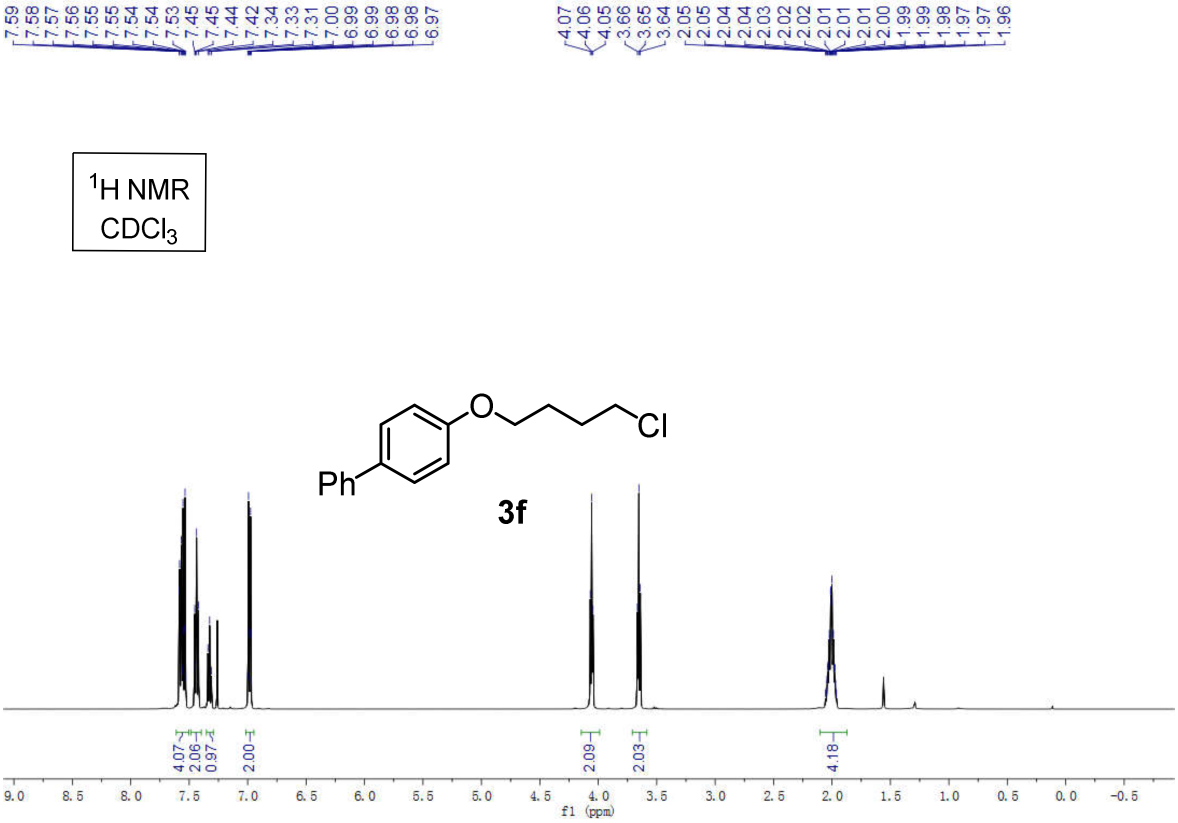


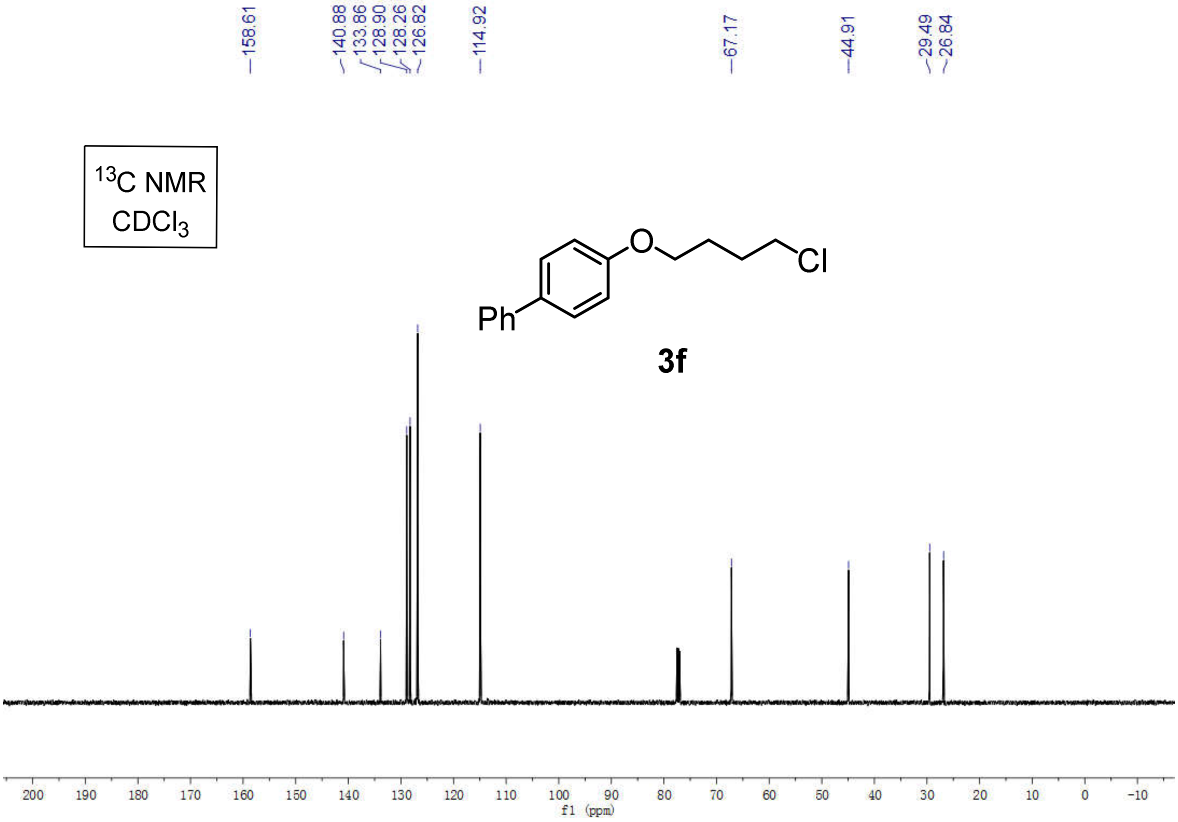


**Supplementary Fig. 29** $^{1}$H NMR and $^{13}$C NMR spectra of the compound **3f**.

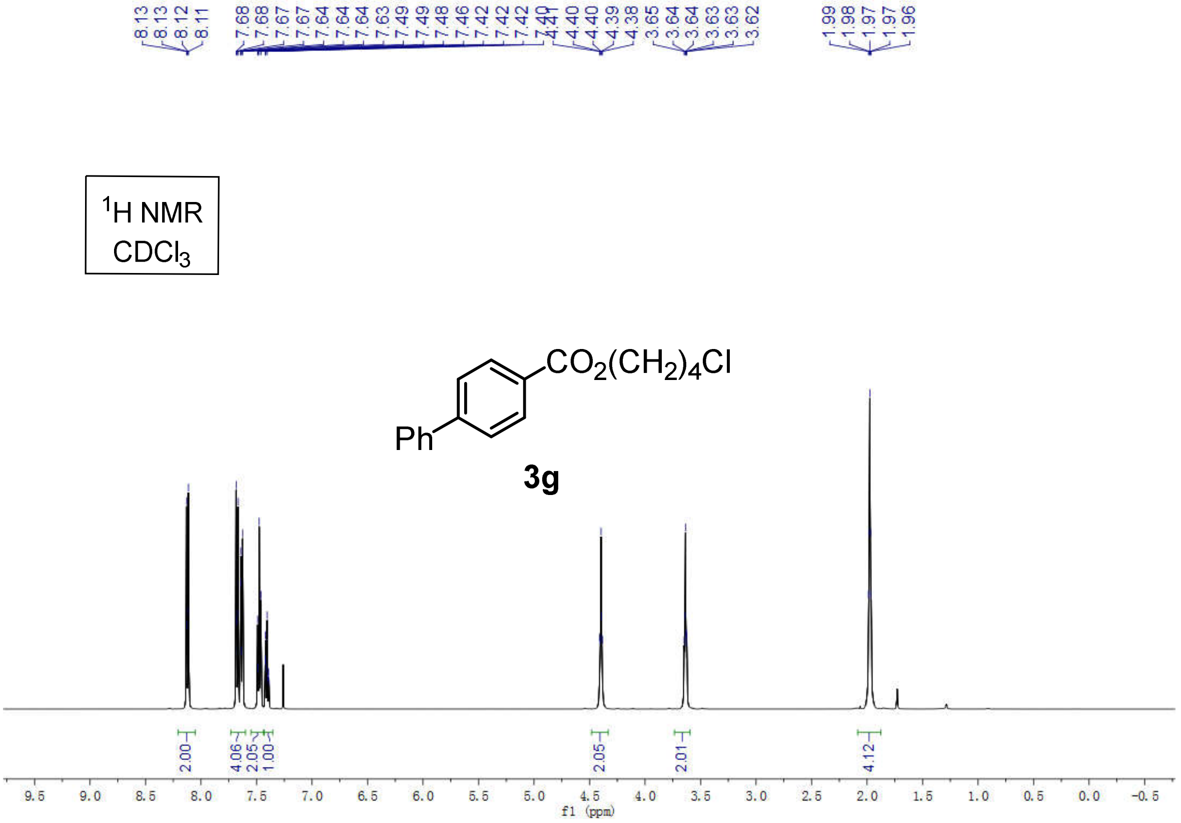


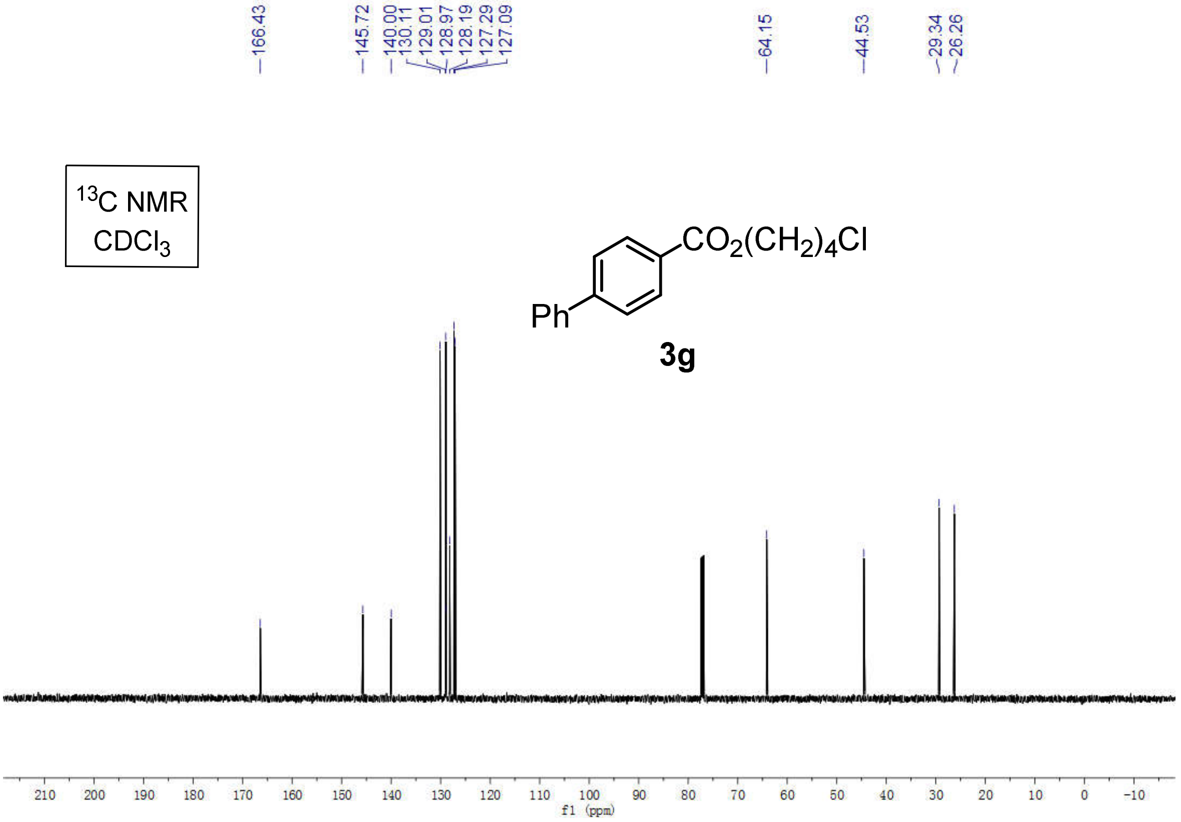


**Supplementary Fig. 30** $^{1}$H NMR and $^{13}$C NMR spectra of the compound **3g**.

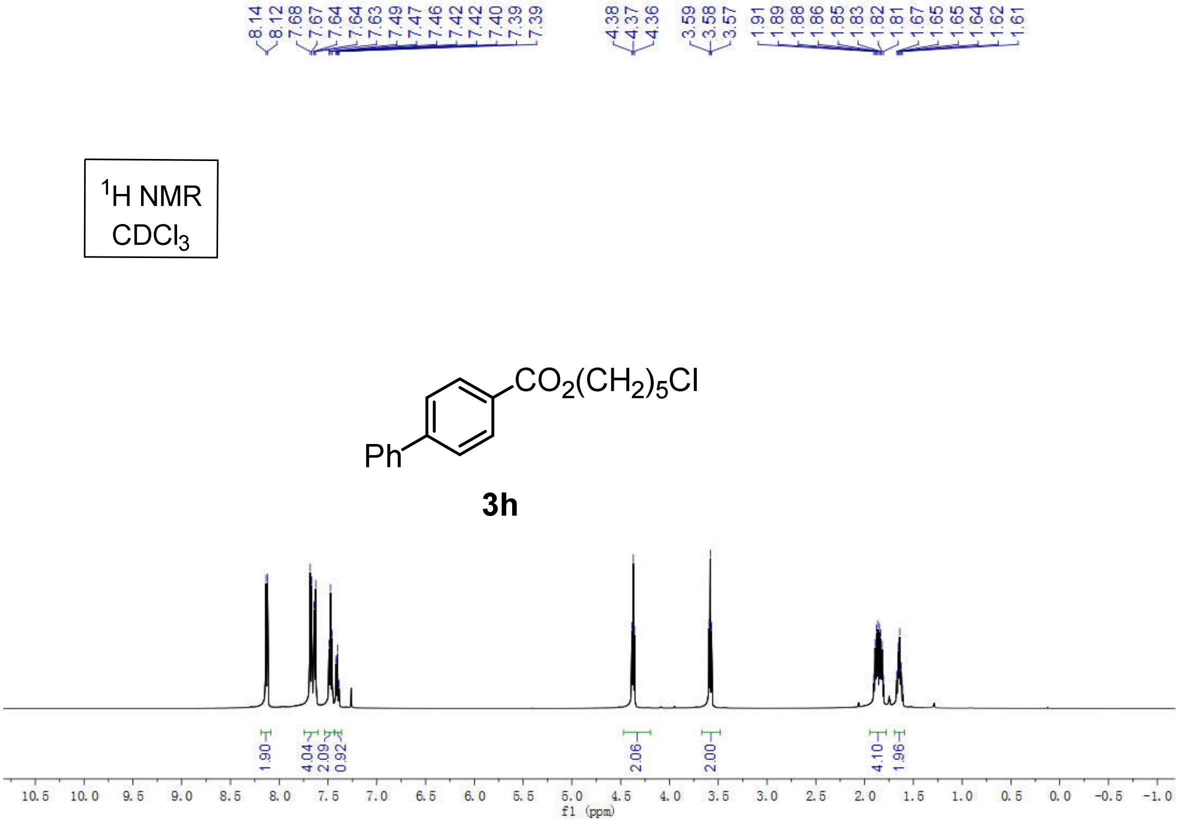


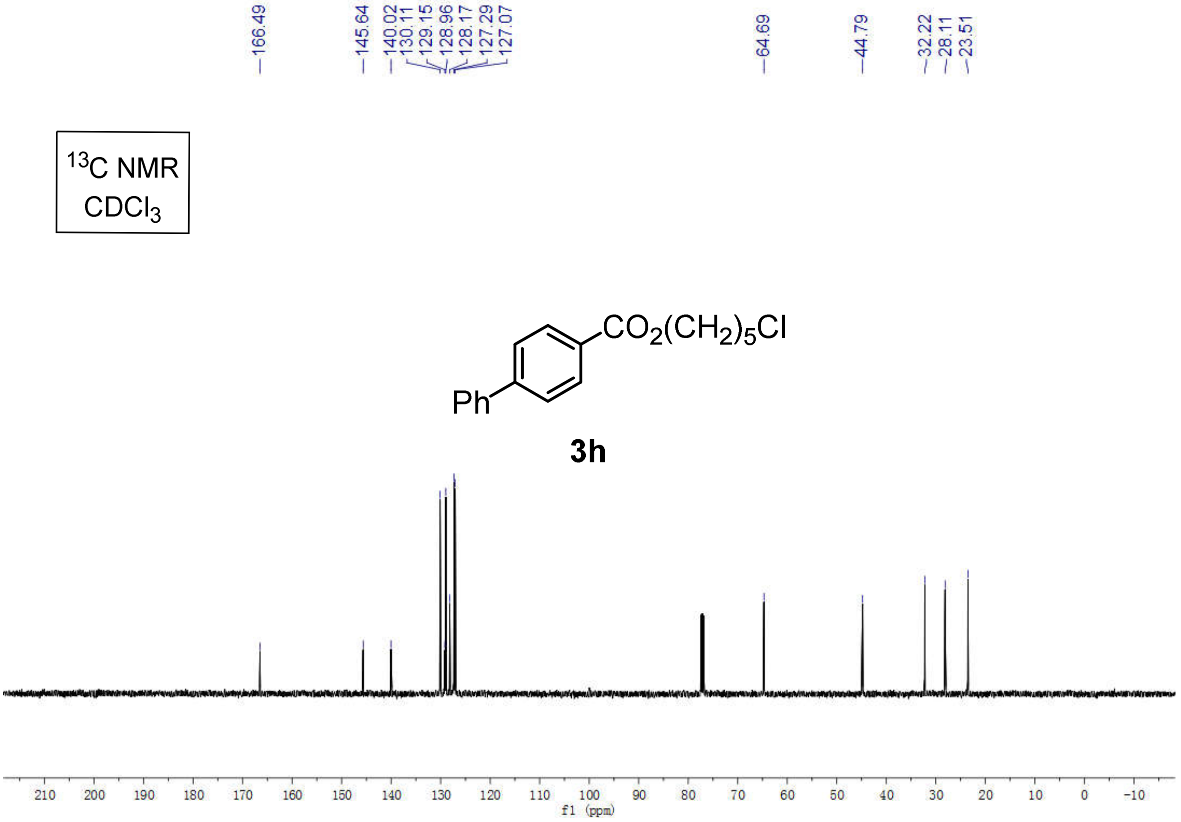


**Supplementary Fig. 31** $^{1}$H NMR and $^{13}$C NMR spectra of the compound **3h**.

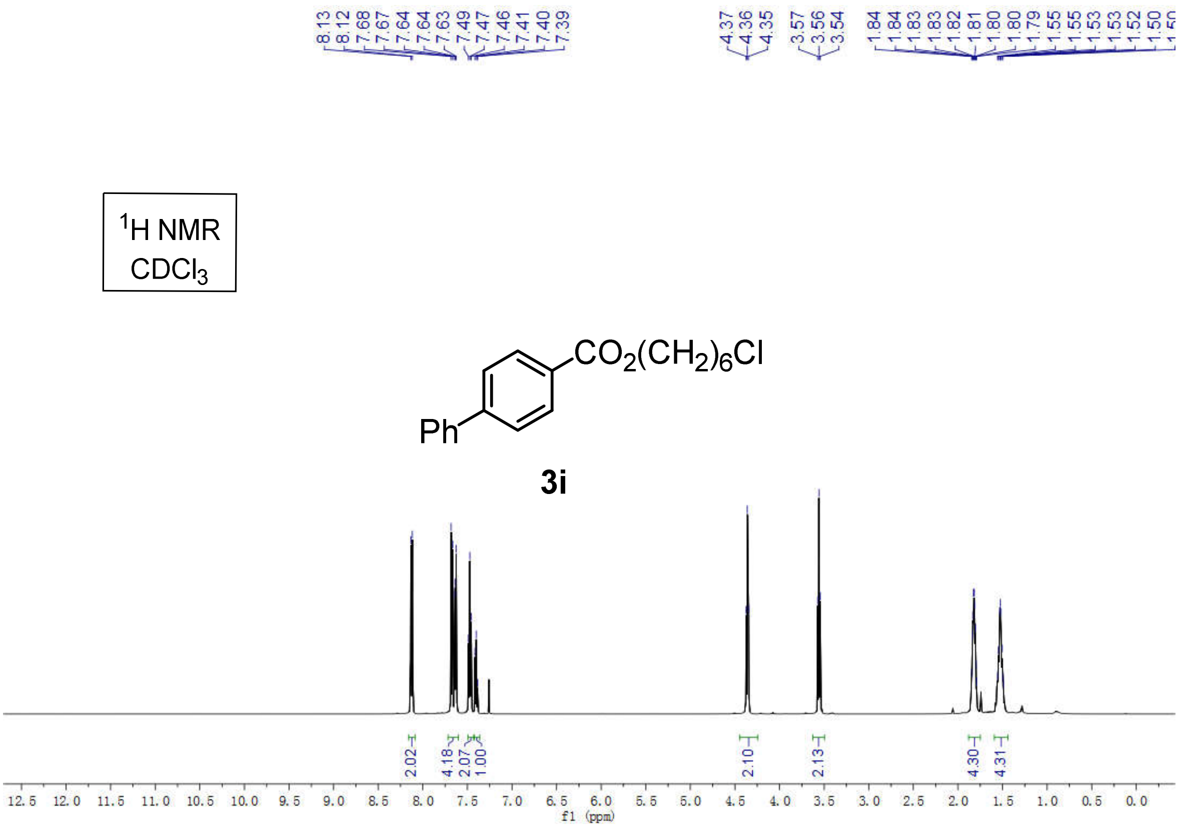

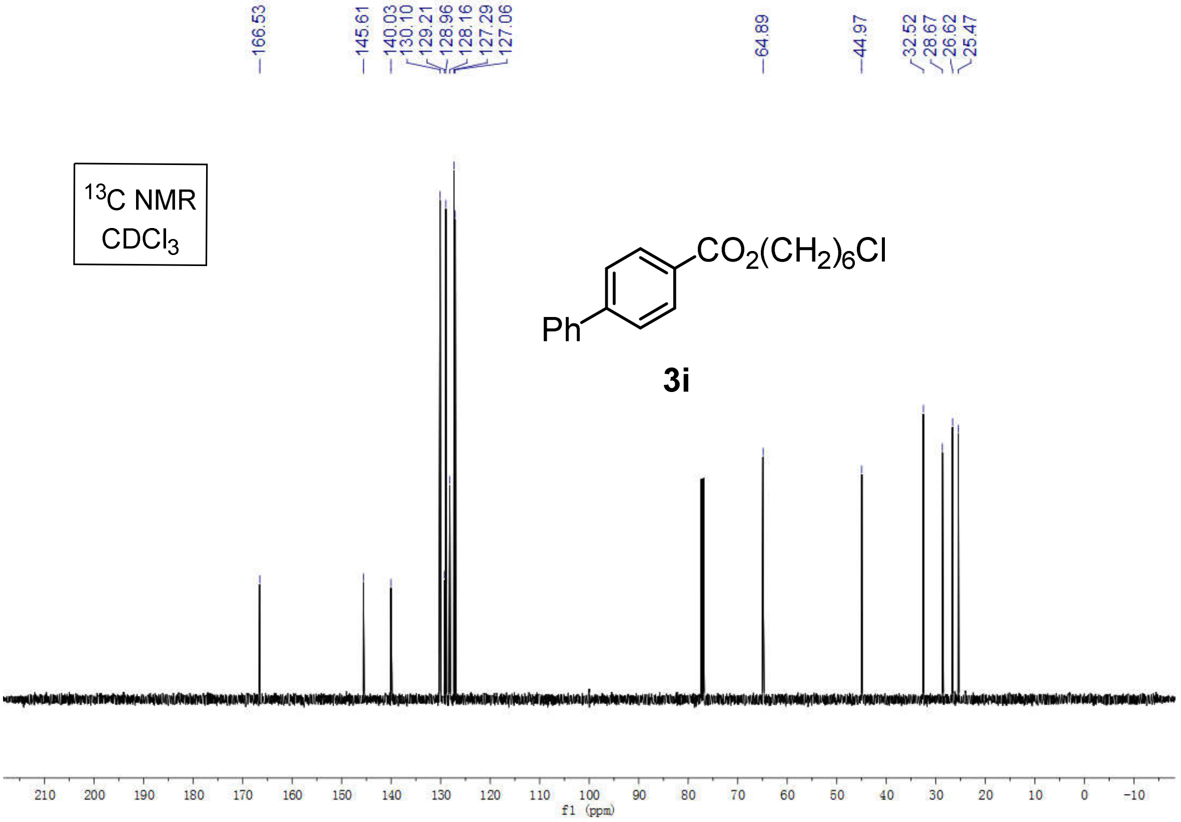

**Supplementary Fig. 32** $^{1}$H NMR and $^{13}$C NMR spectra of the compound **3i**.

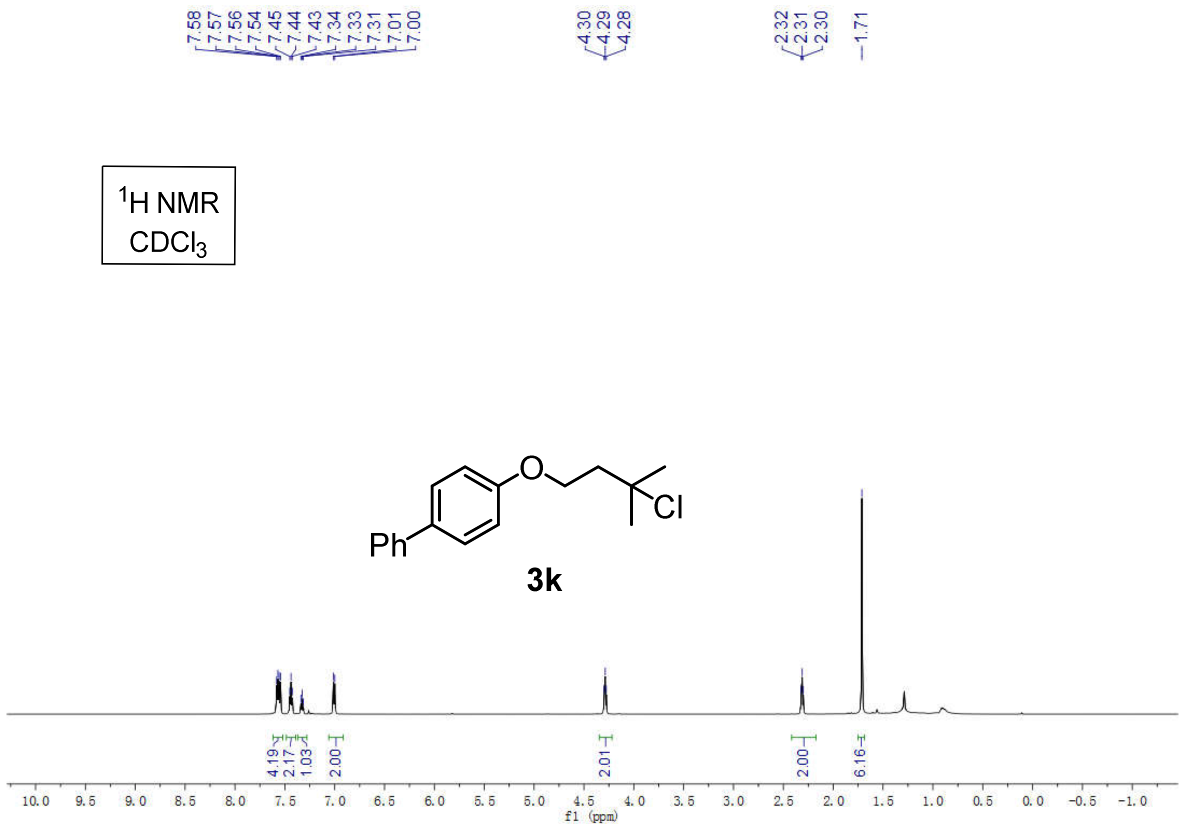


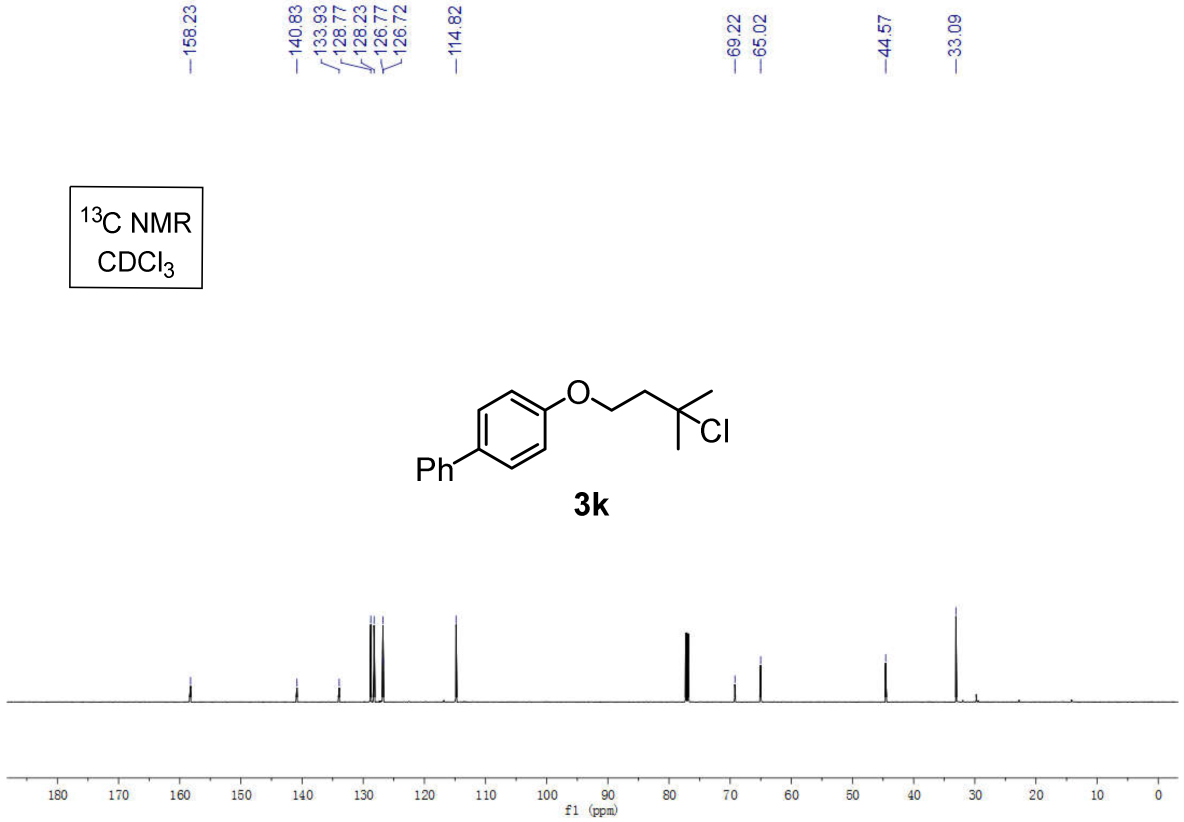


**Supplementary Fig. 33** $^{1}$H NMR and $^{13}$C NMR spectra of the compound **3k**.

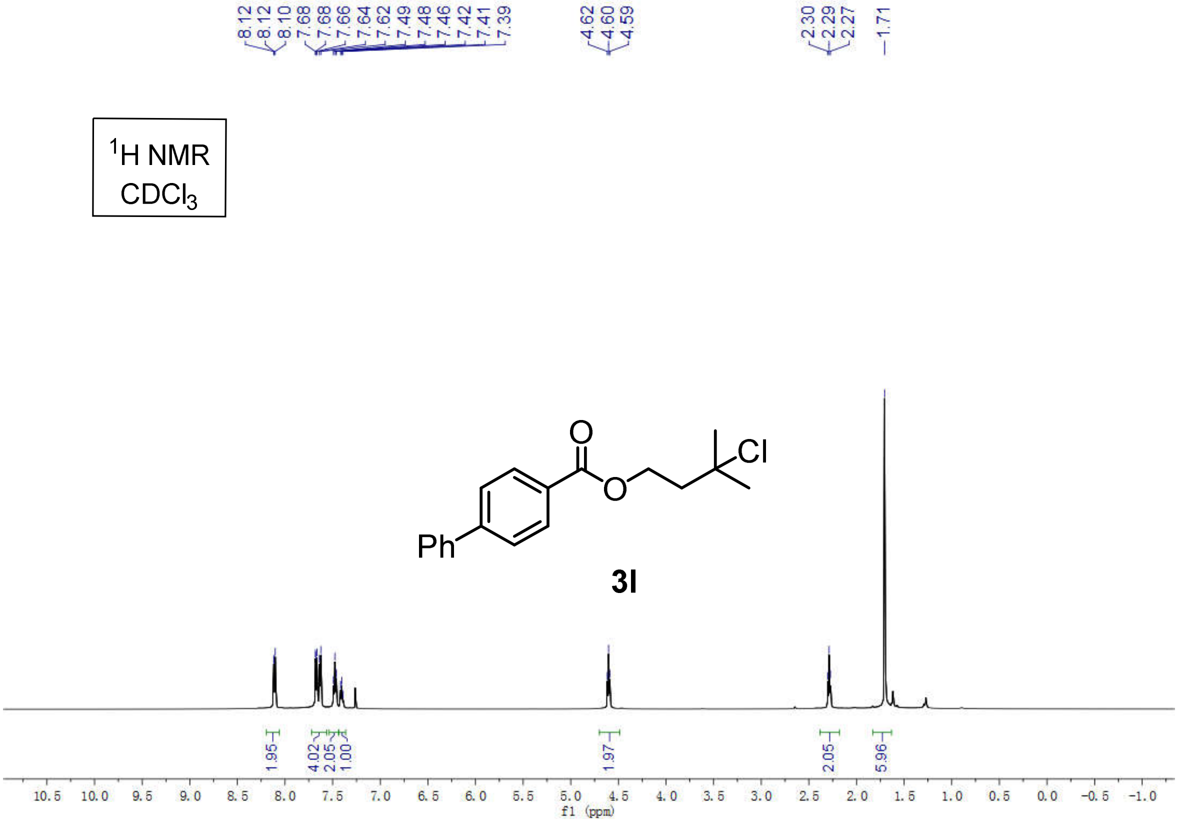


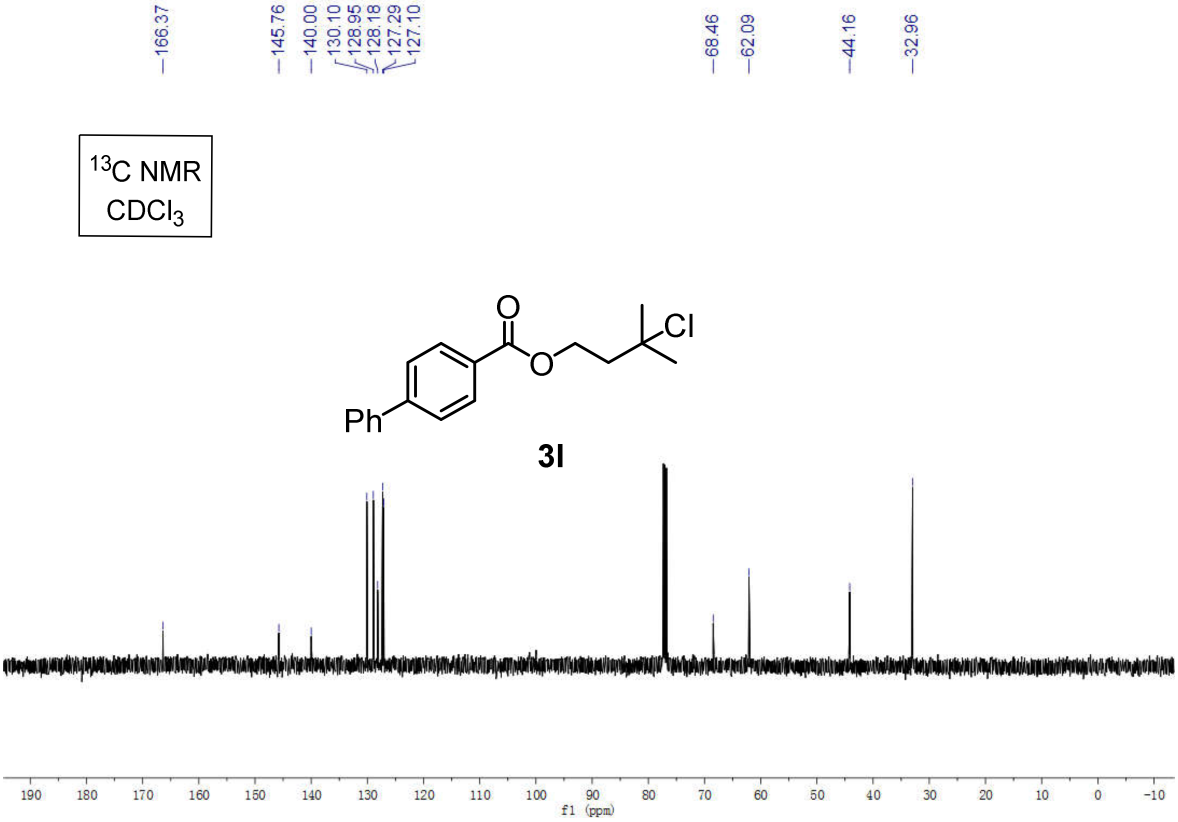


**Supplementary Fig. 34** $^{1}$H NMR and $^{13}$C NMR spectra of the compound **3l**.

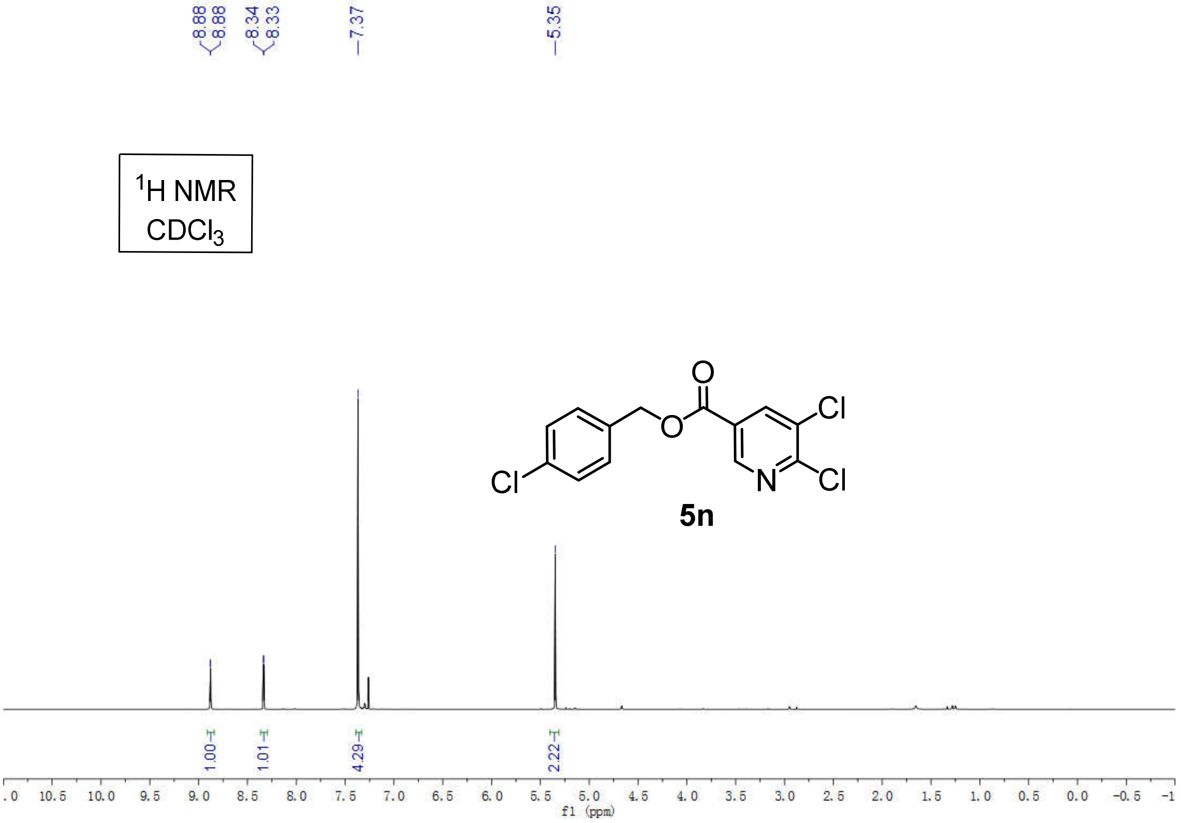


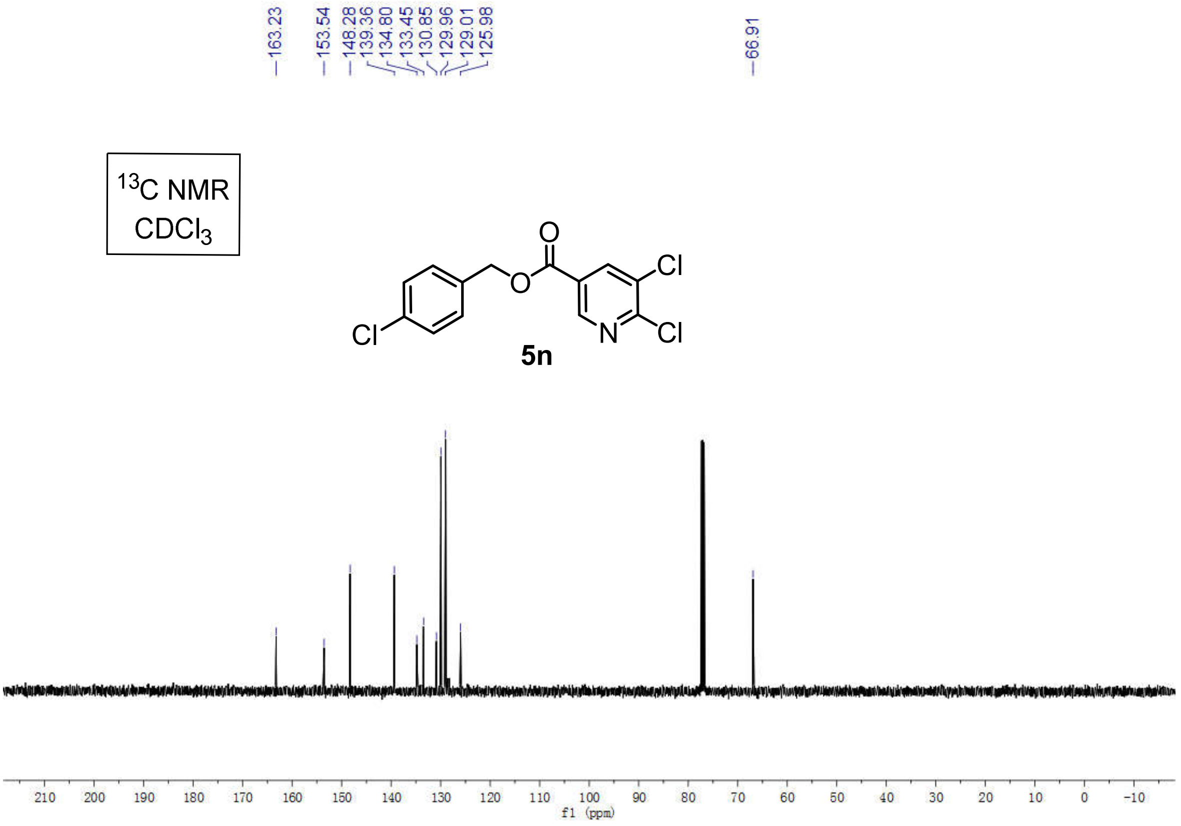


**Supplementary Fig. 35** $^{1}$H NMR and $^{13}$C NMR spectra of the compound **5n**.

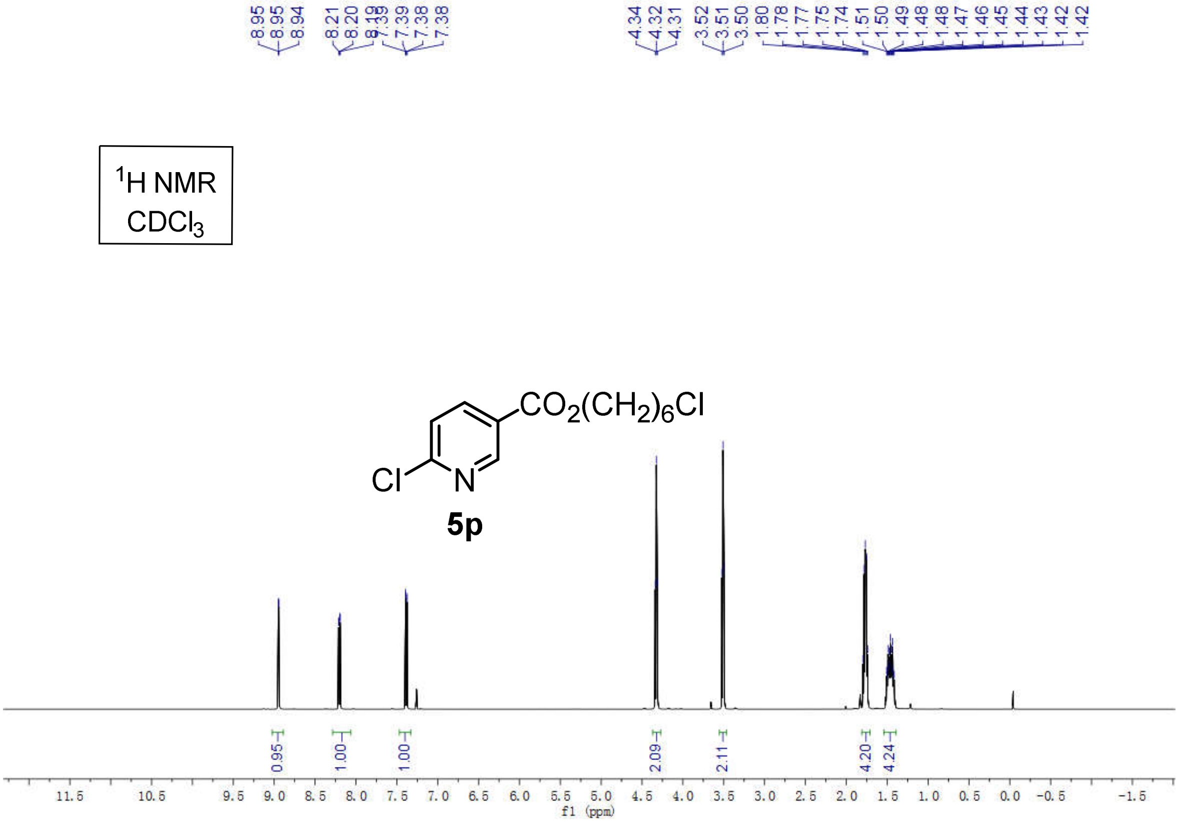

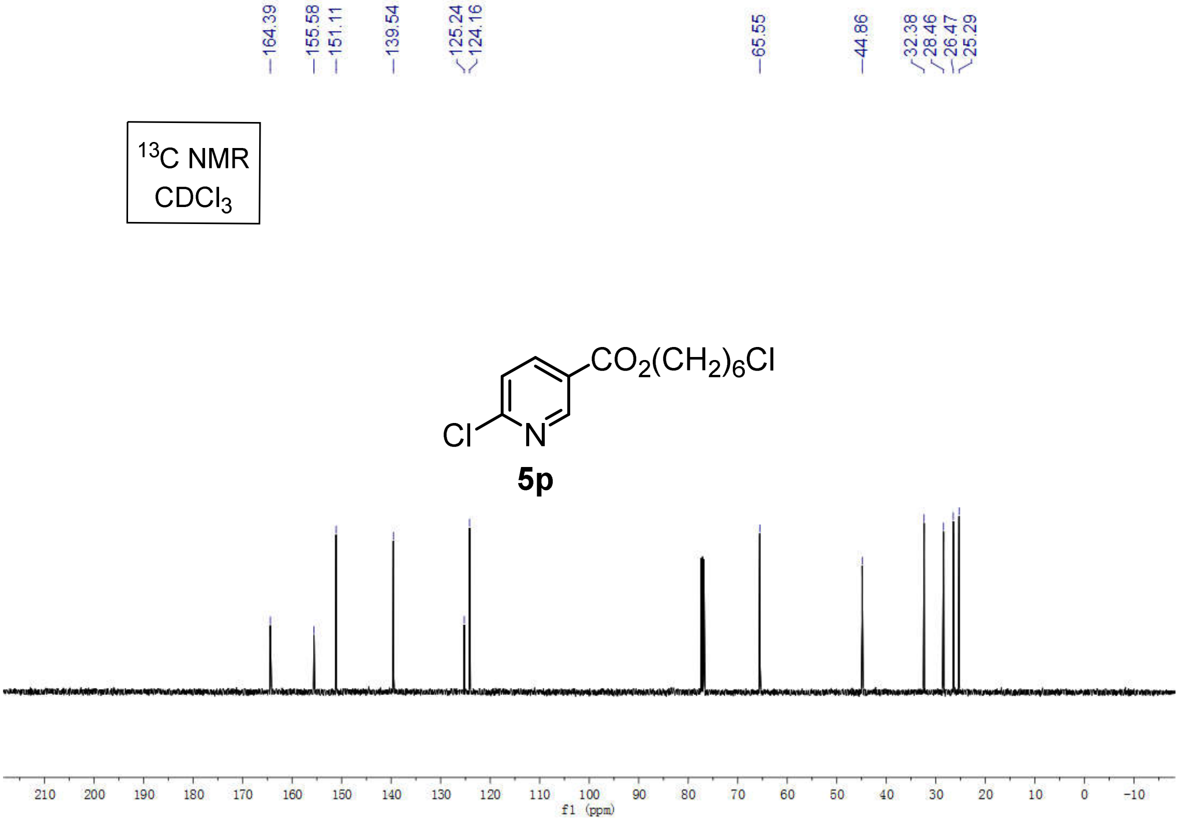

**Supplementary Fig. 36** $^1$H NMR and $^{13}$C NMR spectra of the compound **5p**.

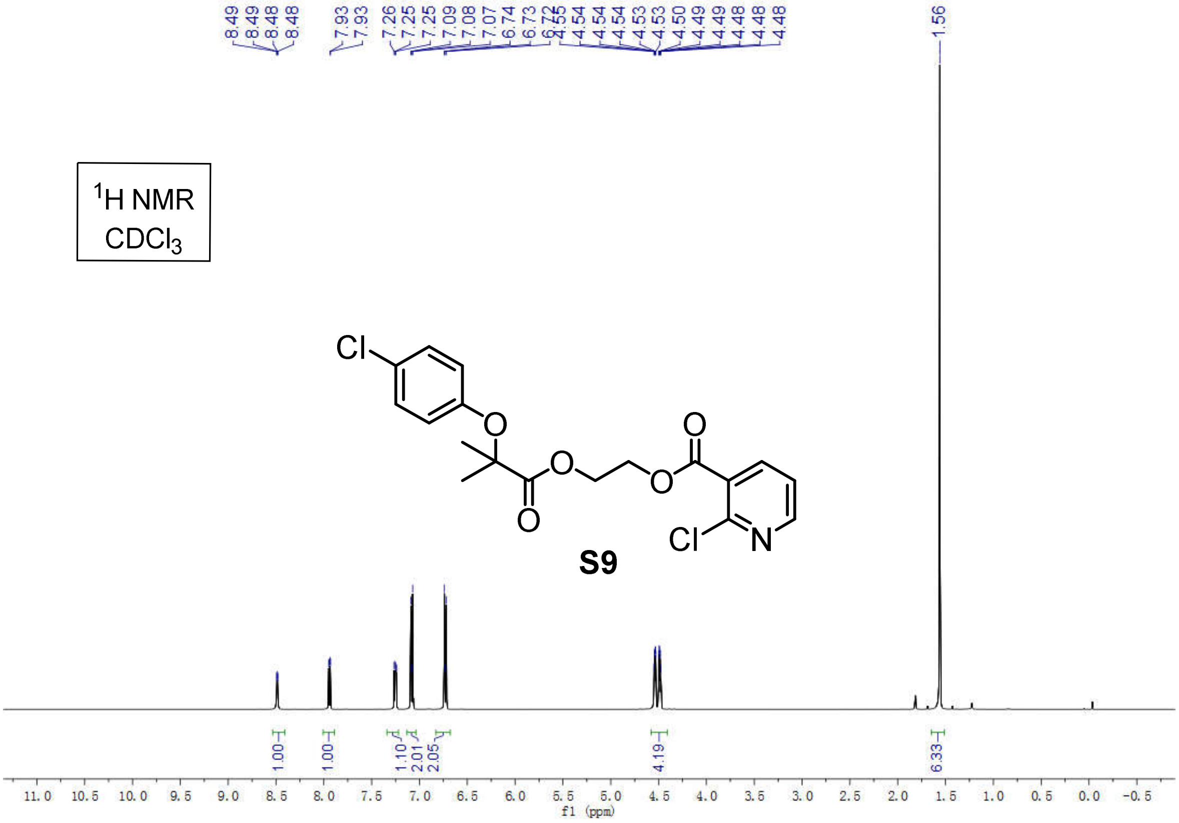


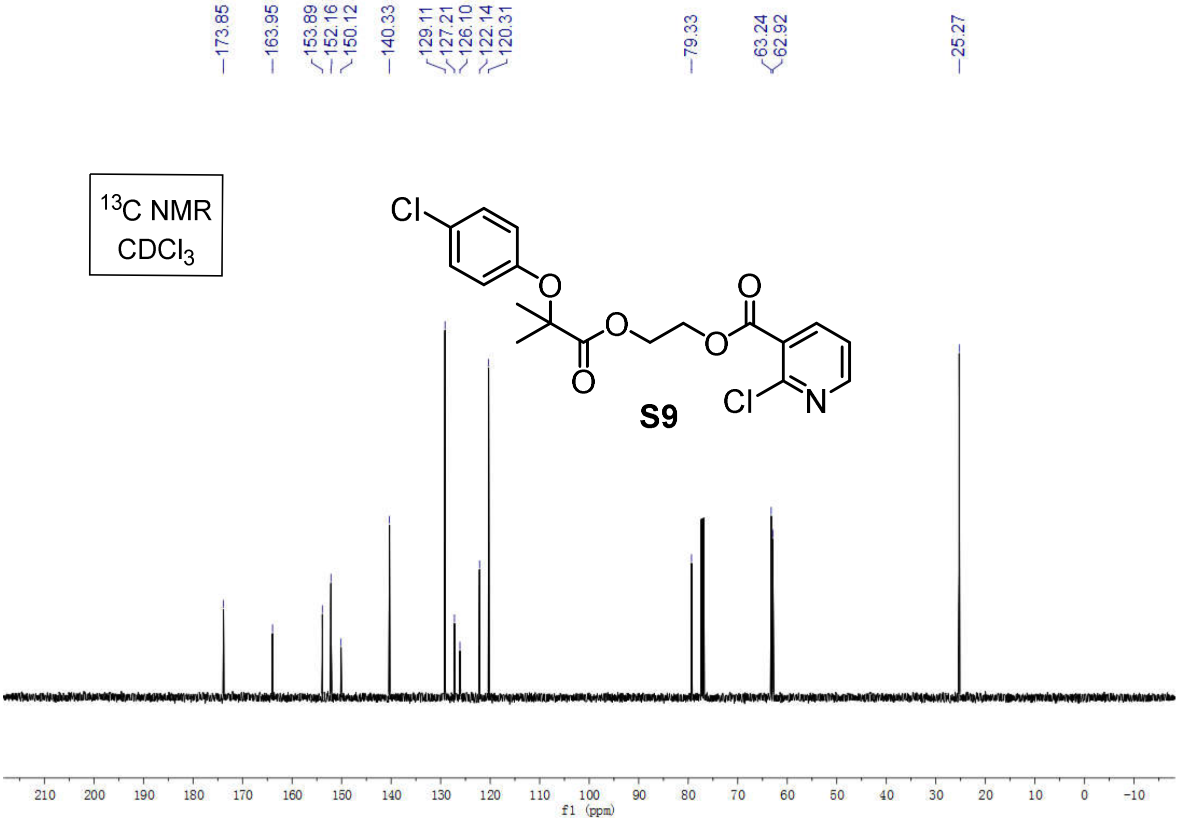


**Supplementary Fig. 37** $^1$H NMR and $^{13}$C NMR spectra of the compound **S9**.

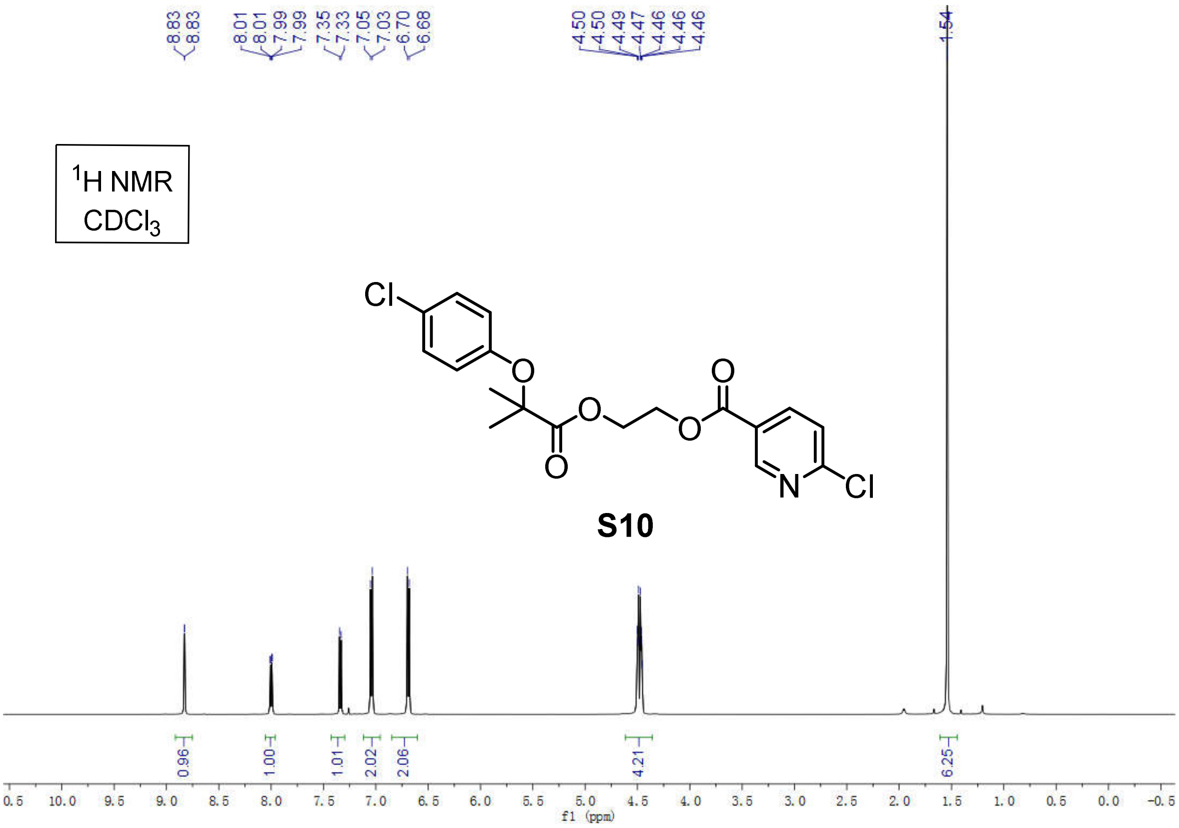


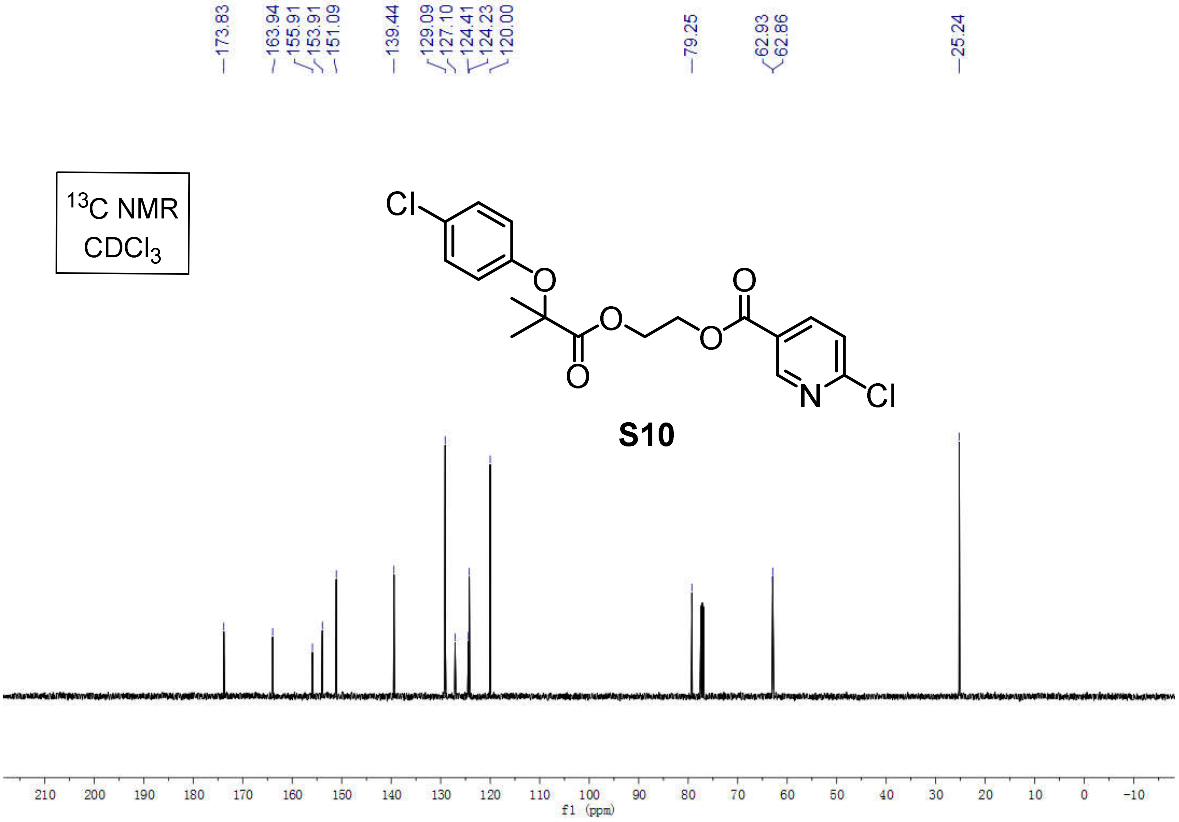


**Supplementary Fig. 38** $^{1}$H NMR and $^{13}$C NMR spectra of the compound **S10**.

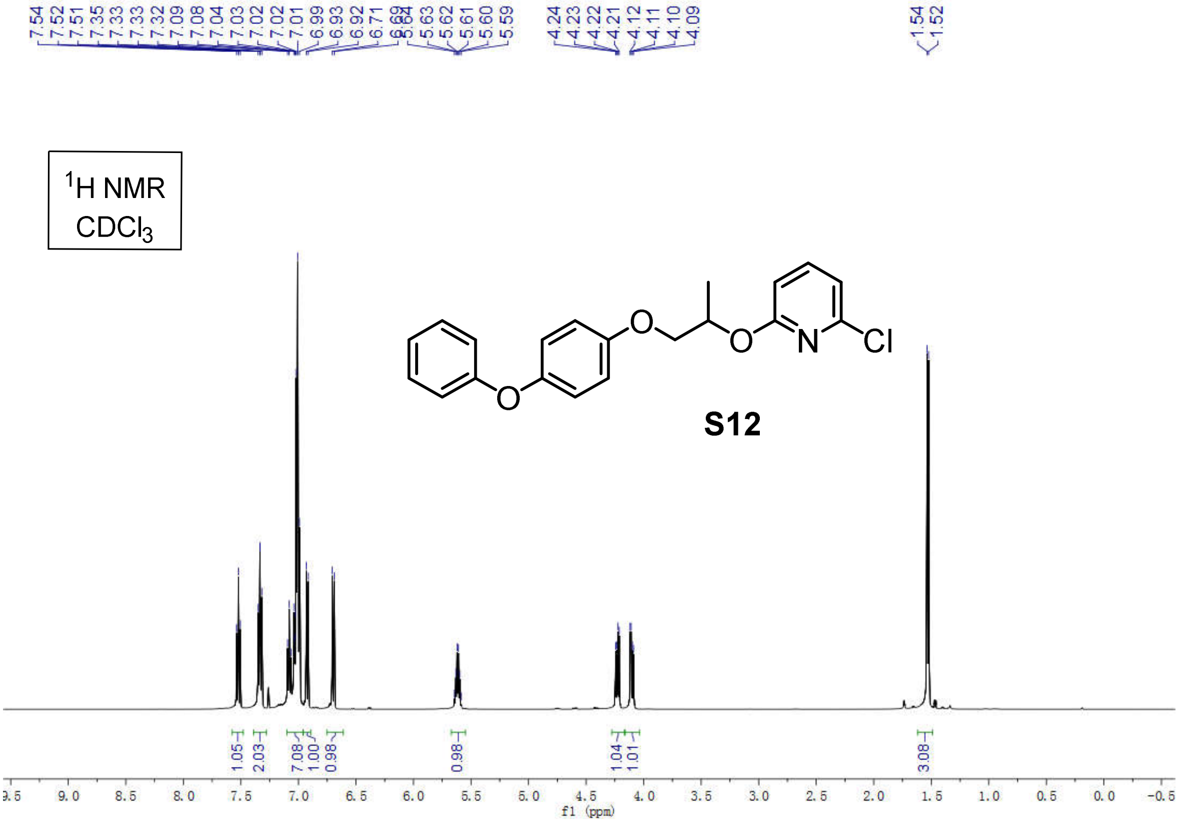


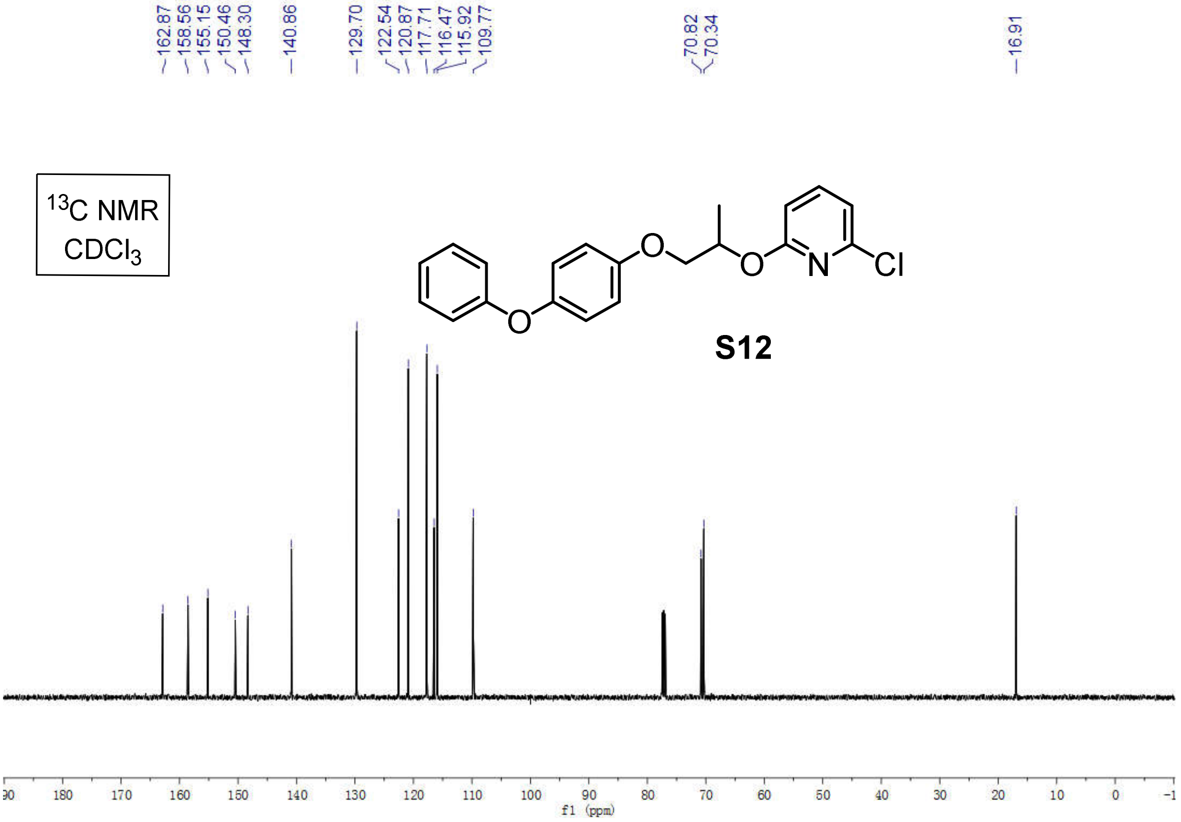


**Supplementary Fig. 39** $^{1}$H NMR and $^{13}$C NMR spectra of the compound **S10**.

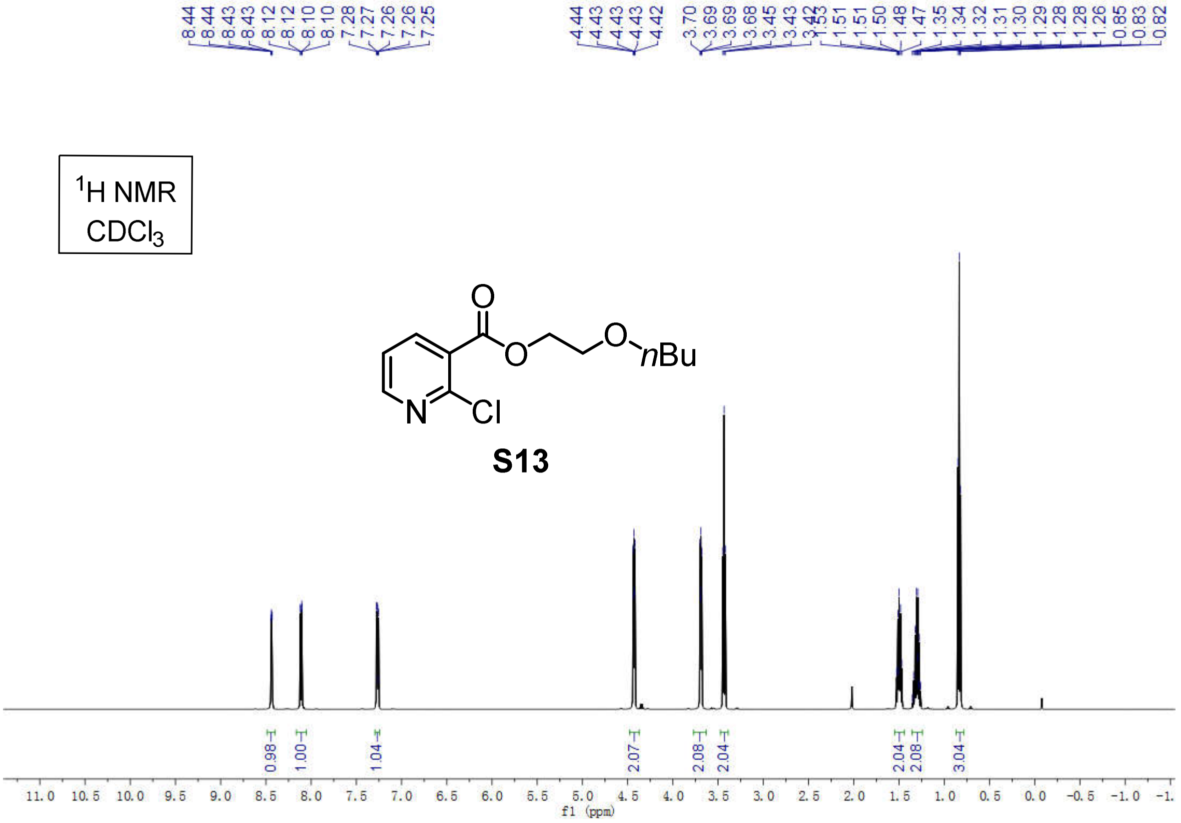

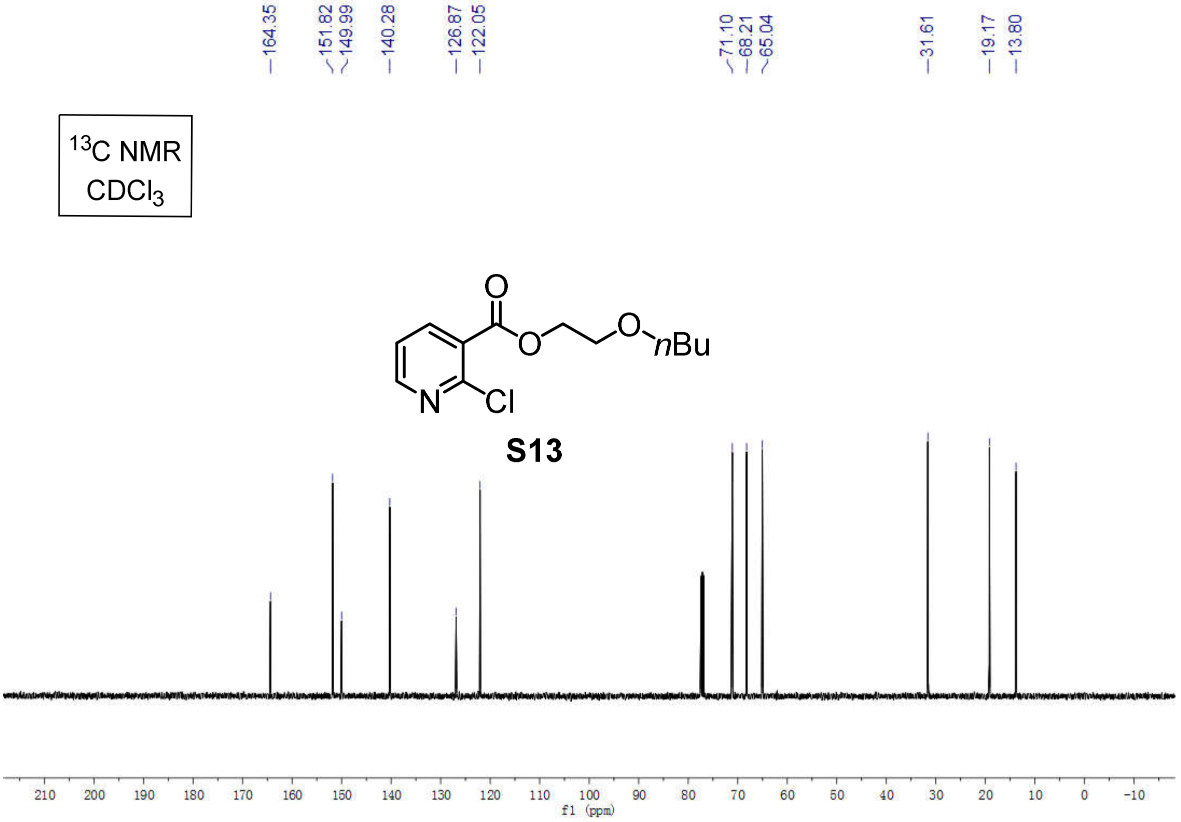

**Supplementary Fig. 40** $^{1}$H NMR and $^{13}$C NMR spectra of the compound **S13**.

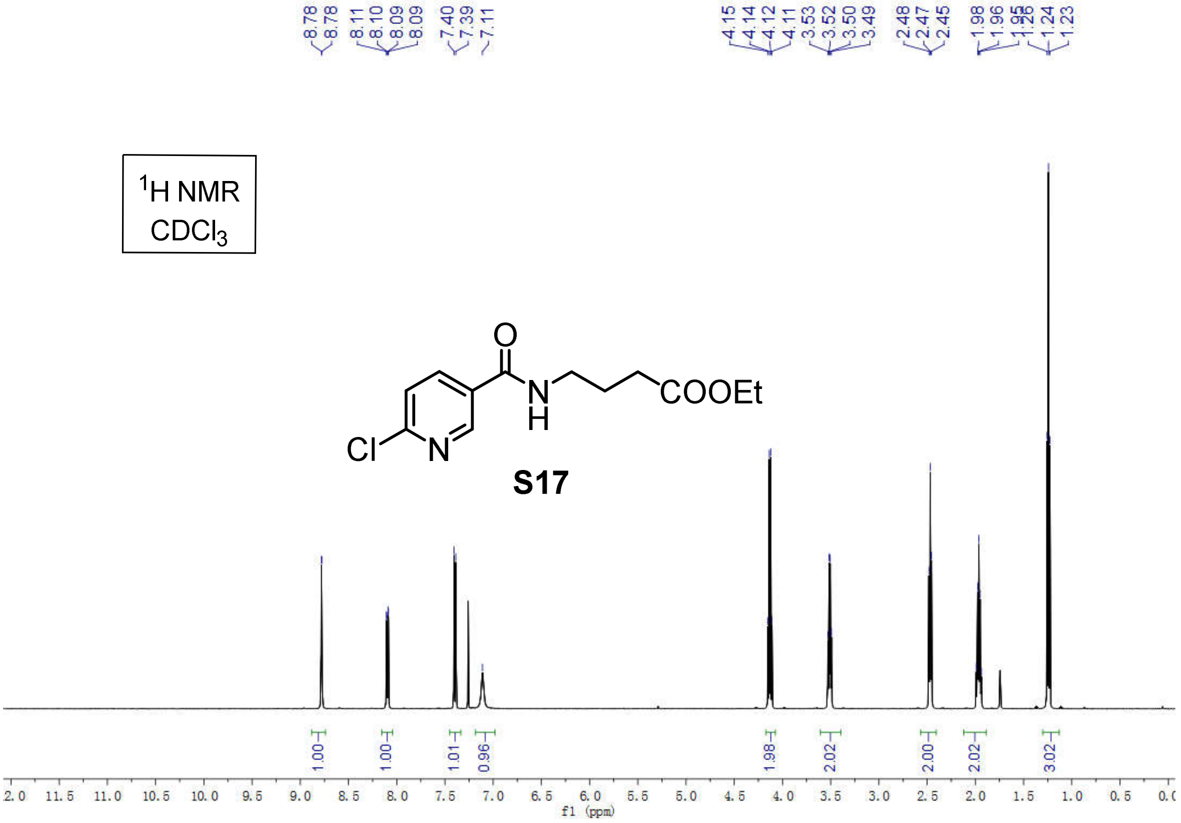


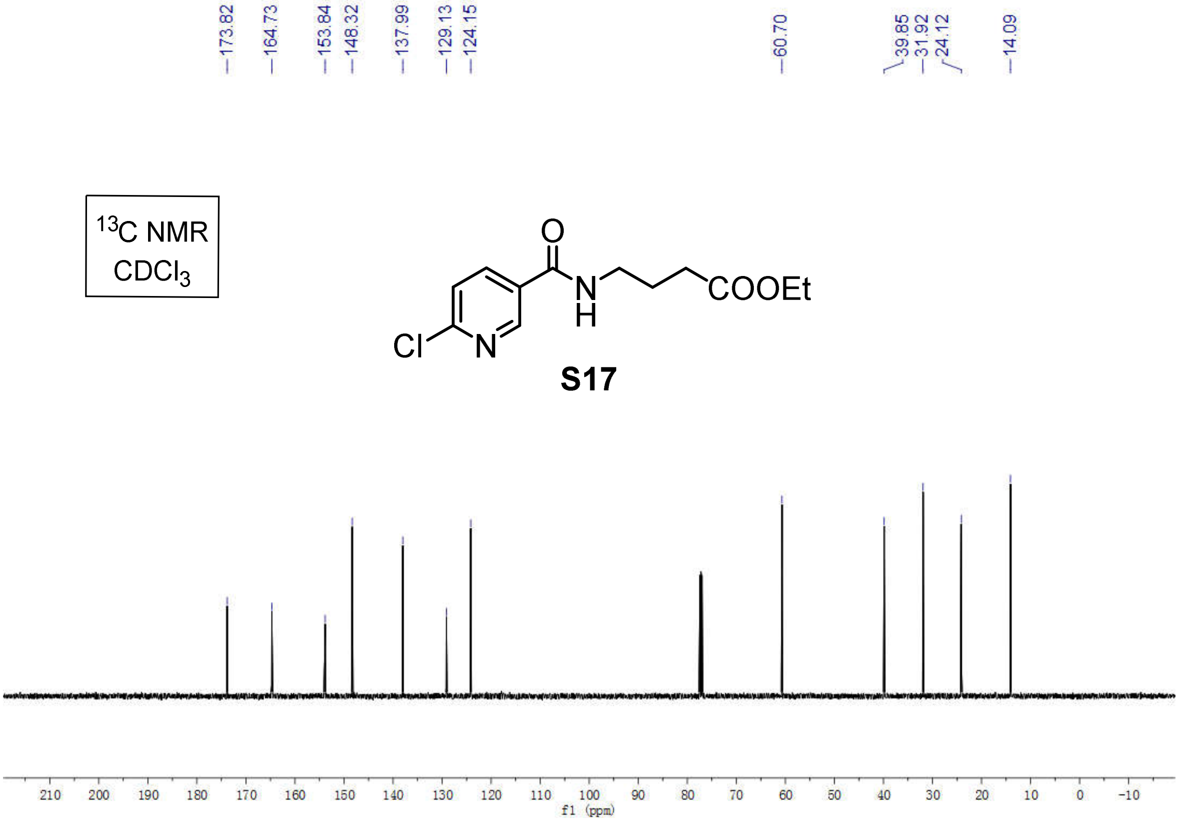


**Supplementary Fig. 41** $^{1}$H NMR and $^{13}$C NMR spectra of the compound **S17**.

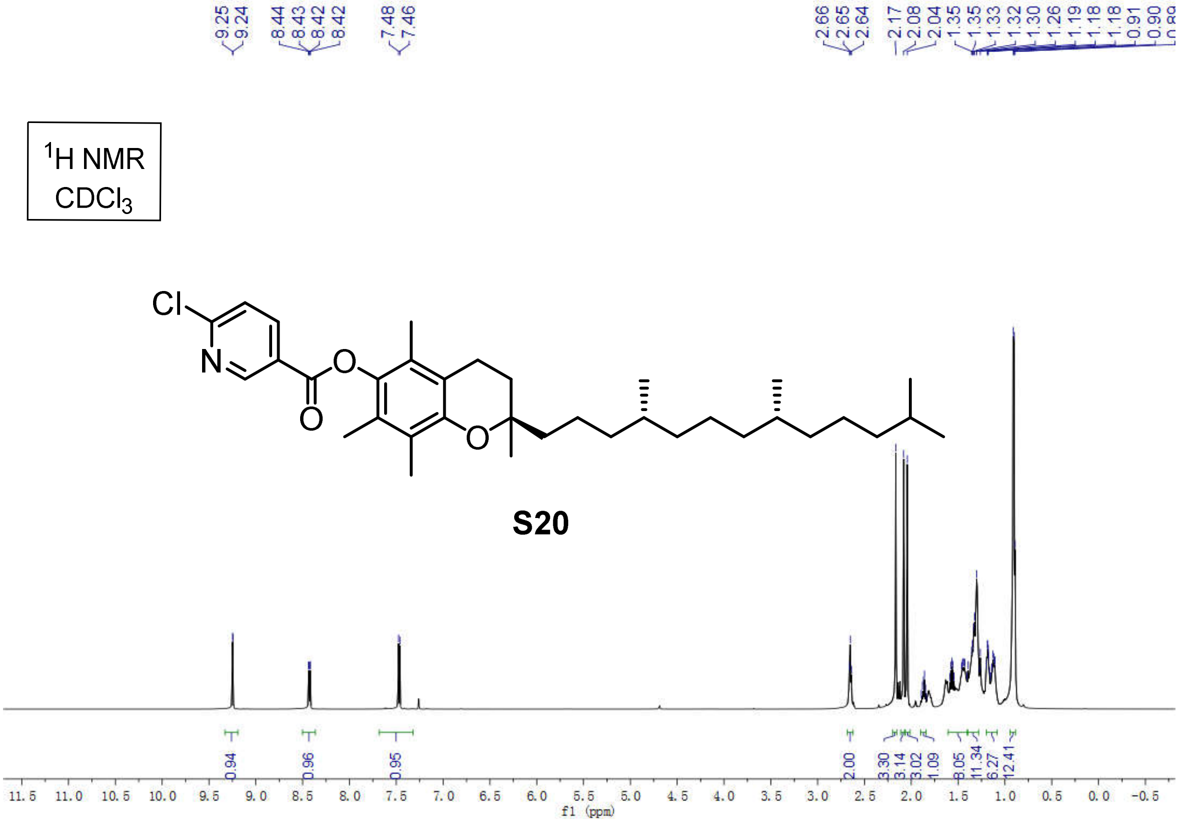


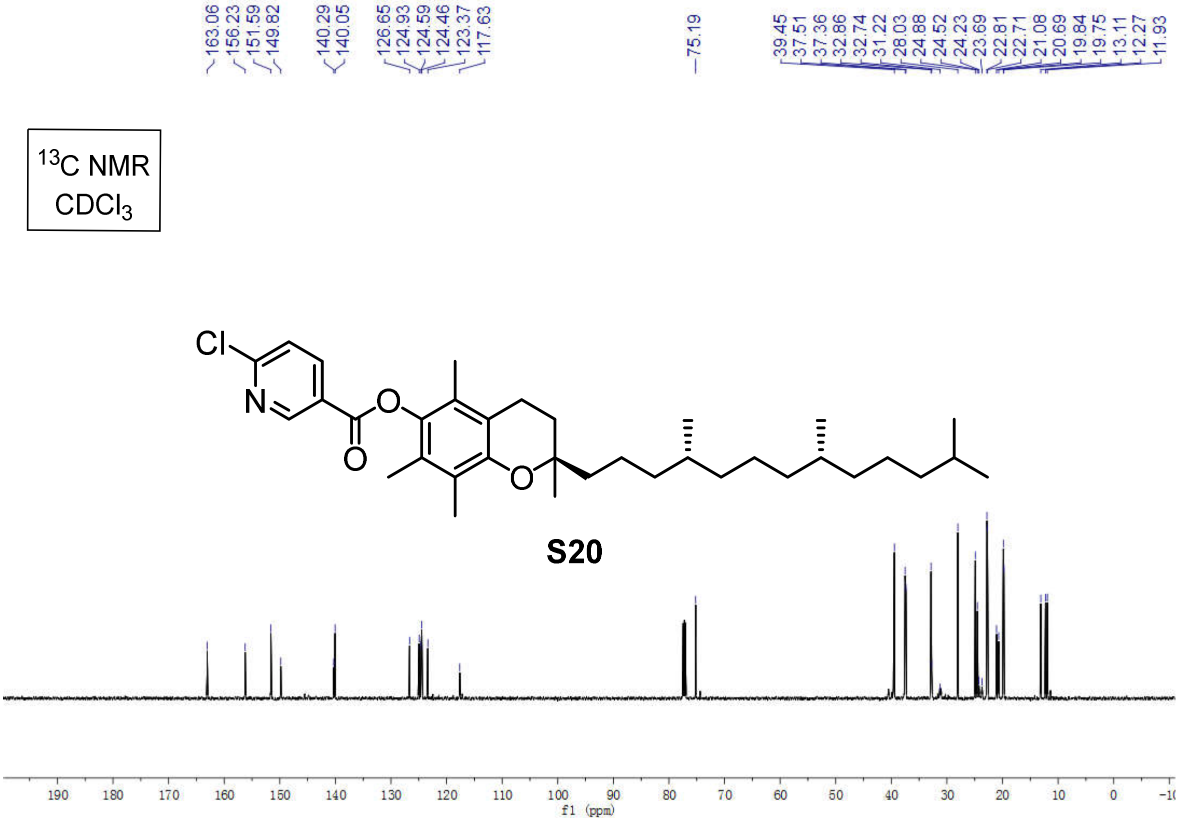


**Supplementary Fig. 42** $^{1}$H NMR and $^{13}$C NMR spectra of the compound **S20**.

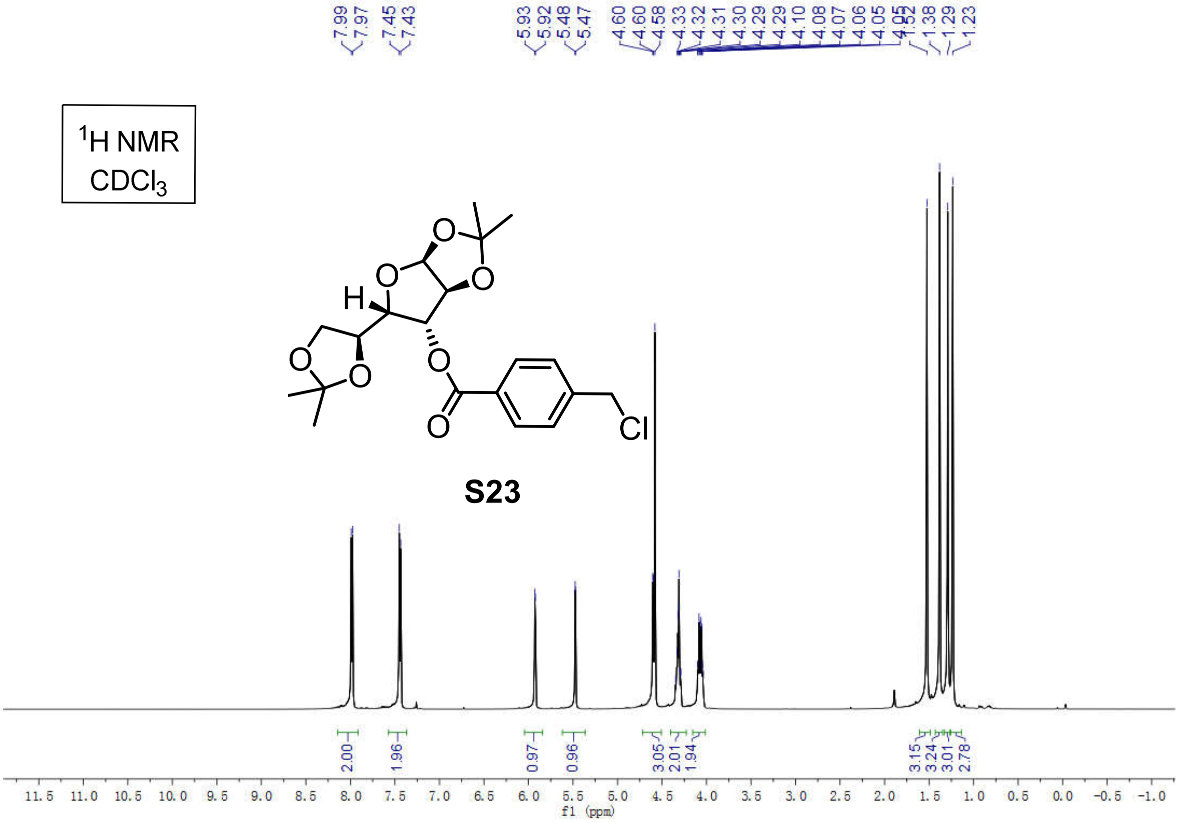


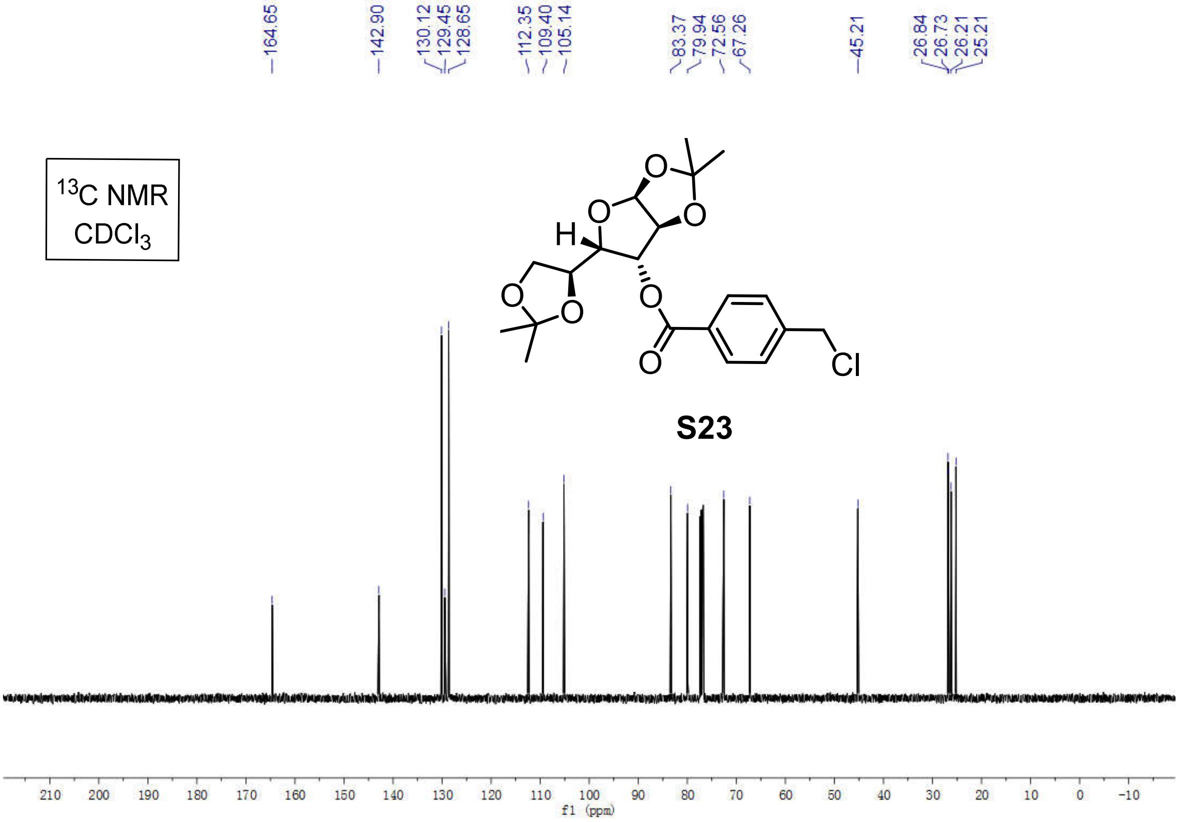


**Supplementary Fig. 43** $^{1}$H NMR and $^{13}$C NMR spectra of the compound **S23**.

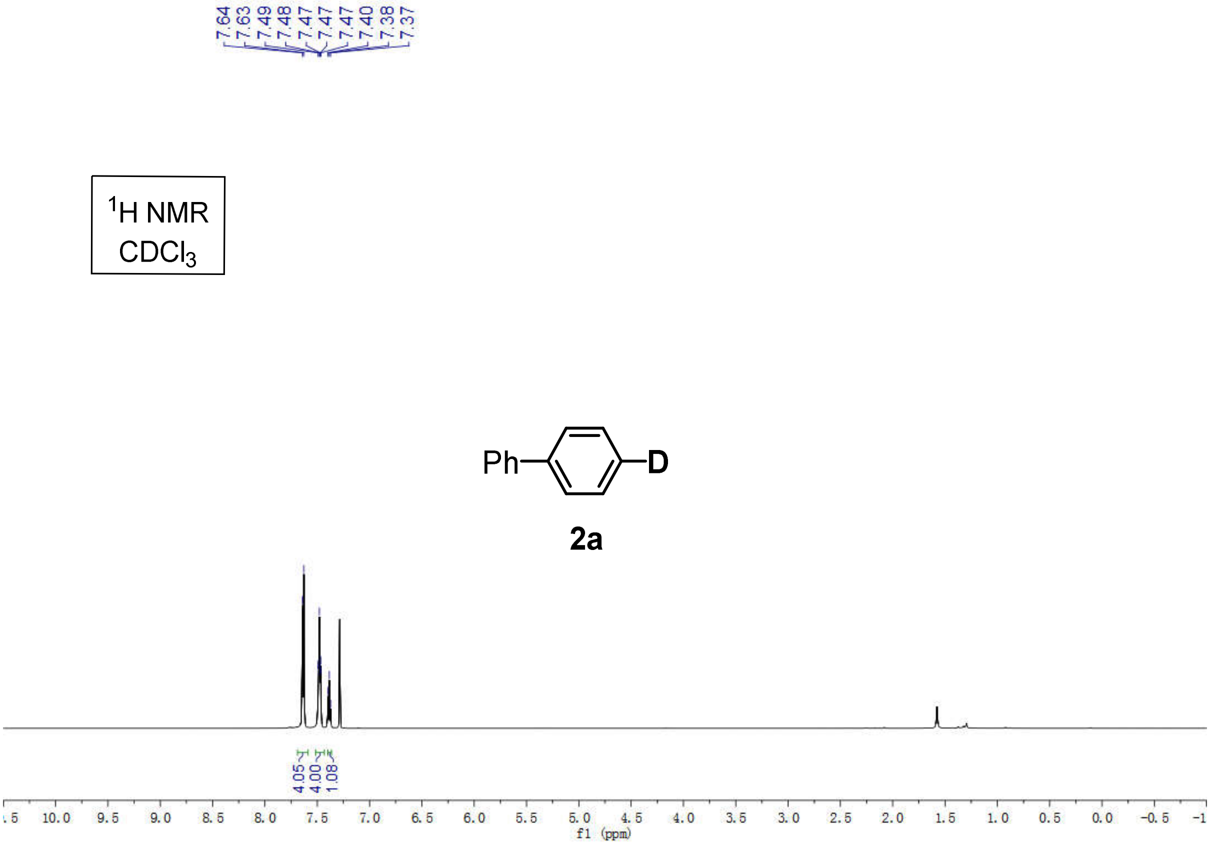
7.64
7.63
7.49
7.48
7.47
7.47
7.47
7.40
7.38
7.37
1H NMR
CDCl3
Ph
D
2a
4.05
4.00
1.08
10.0 9.5 9.0 8.5 8.0 7.5 7.0 6.5 6.0 5.5 5.0 4.5 4.0 3.5 3.0 2.5 2.0 1.5 1.0 0.5 0.0 -0.5 -1
f1 (ppm)

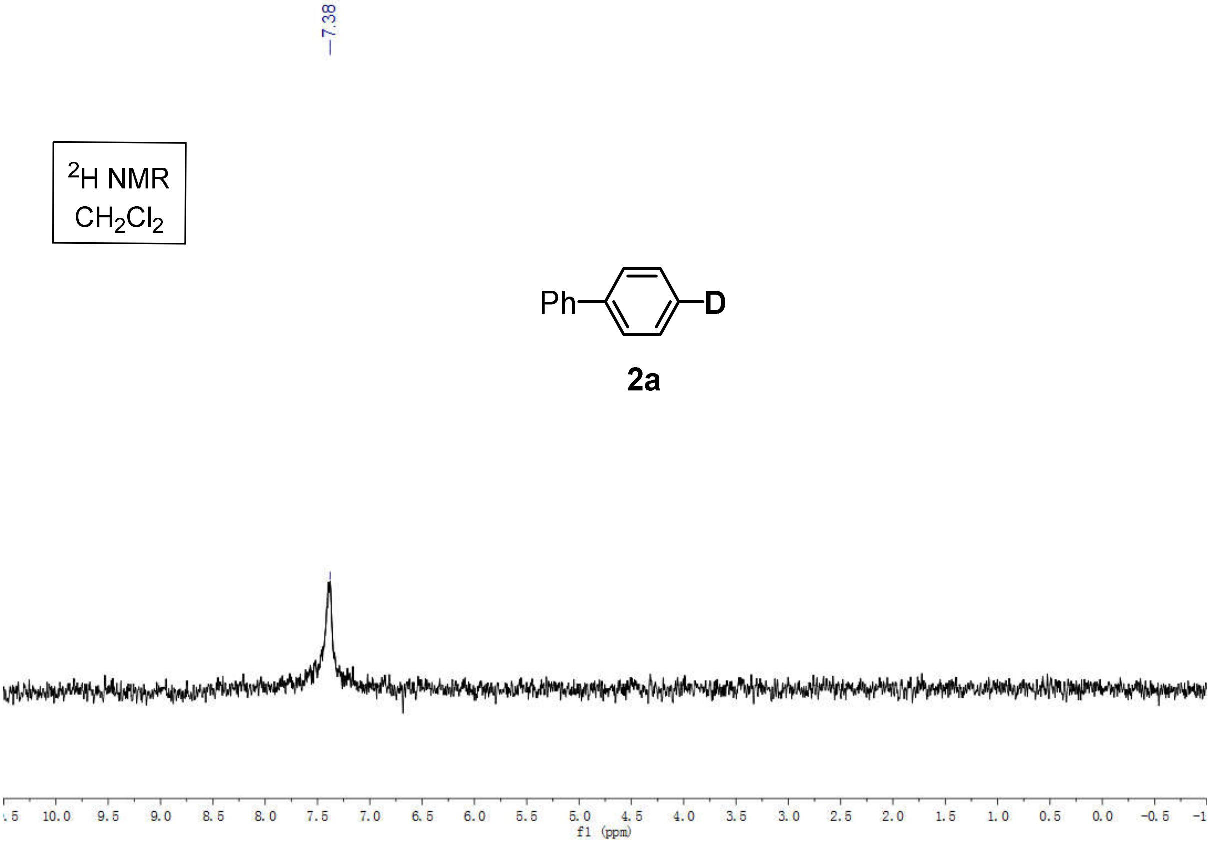
7.38
2H NMR
CH2Cl2
Ph
D
2a
10.0 9.5 9.0 8.5 8.0 7.5 7.0 6.5 6.0 5.5 5.0 4.5 4.0 3.5 3.0 2.5 2.0 1.5 1.0 0.5 0.0 -0.5 -1
f1 (ppm)

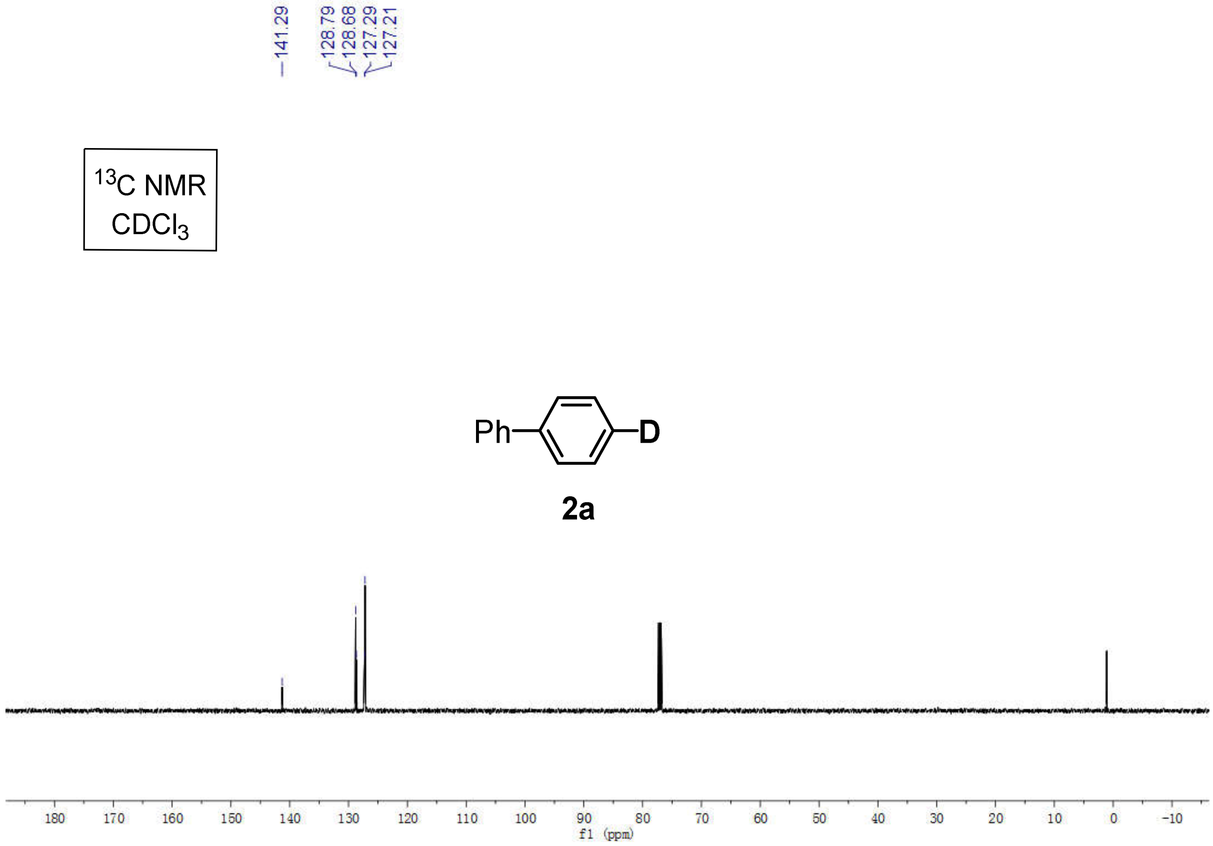


**Supplementary Fig. 44** $^{1}$H NMR, $^{2}$H NMR and $^{13}$C NMR spectra of the compound **2a**.

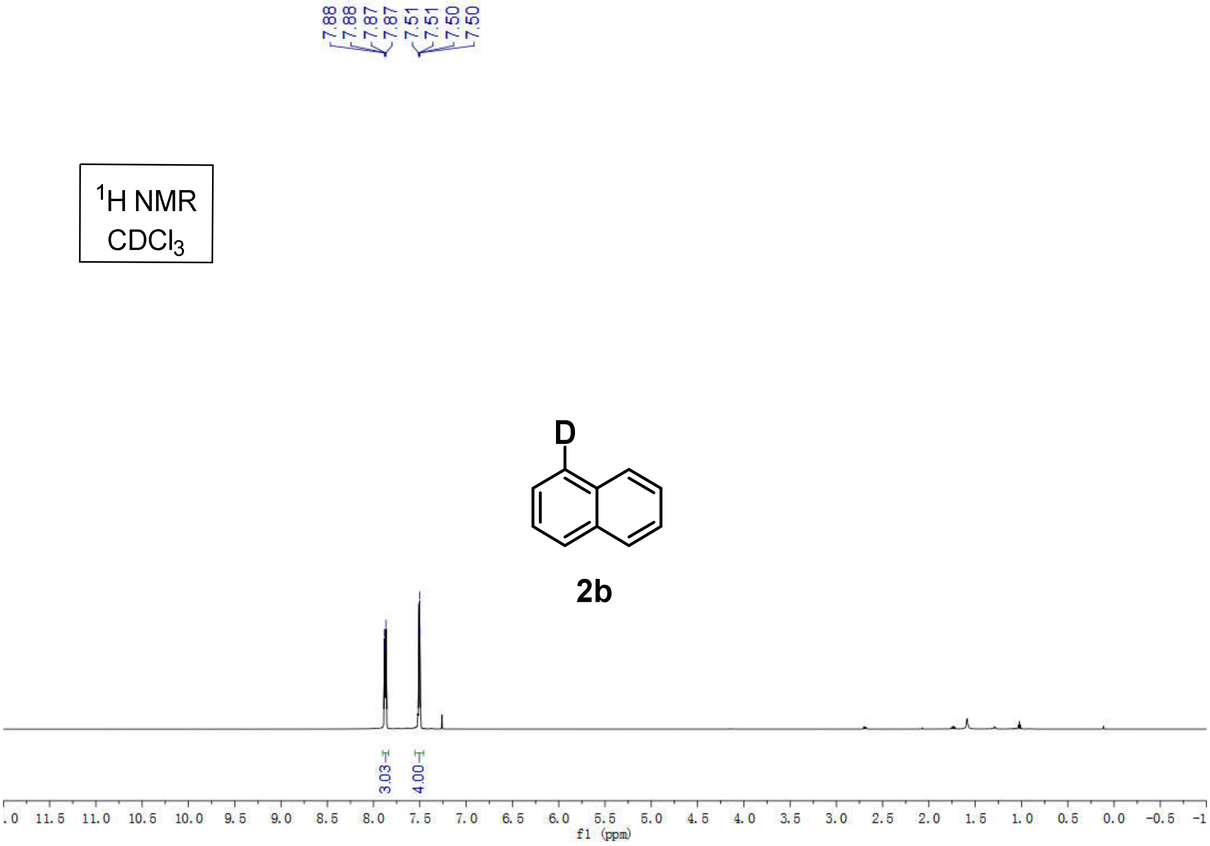

7.88
7.88
7.87
7.87
7.51
7.51
7.50
7.50
1H NMR
CDCl3
D
2b
3.03
4.00
f1 (ppm)


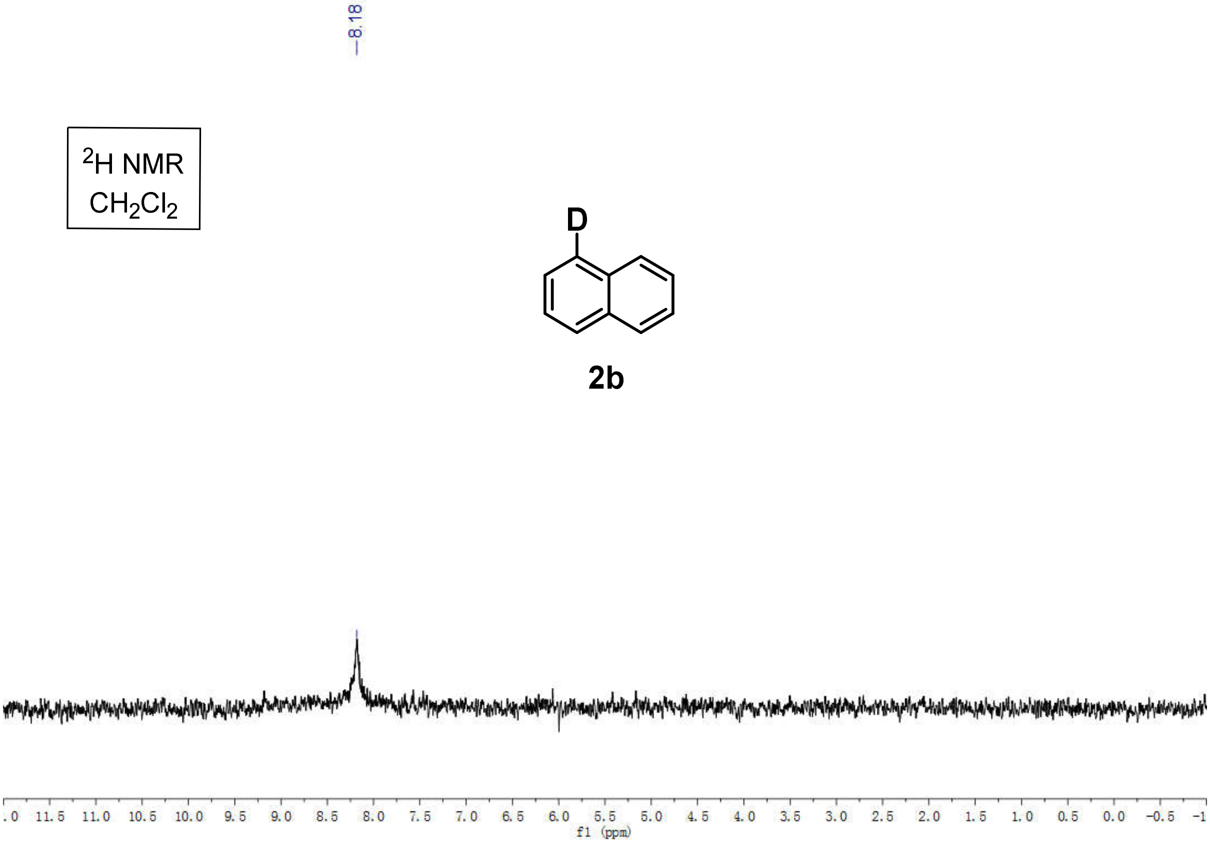

8.18
2H NMR
CH2Cl2
D
2b
f1 (ppm)

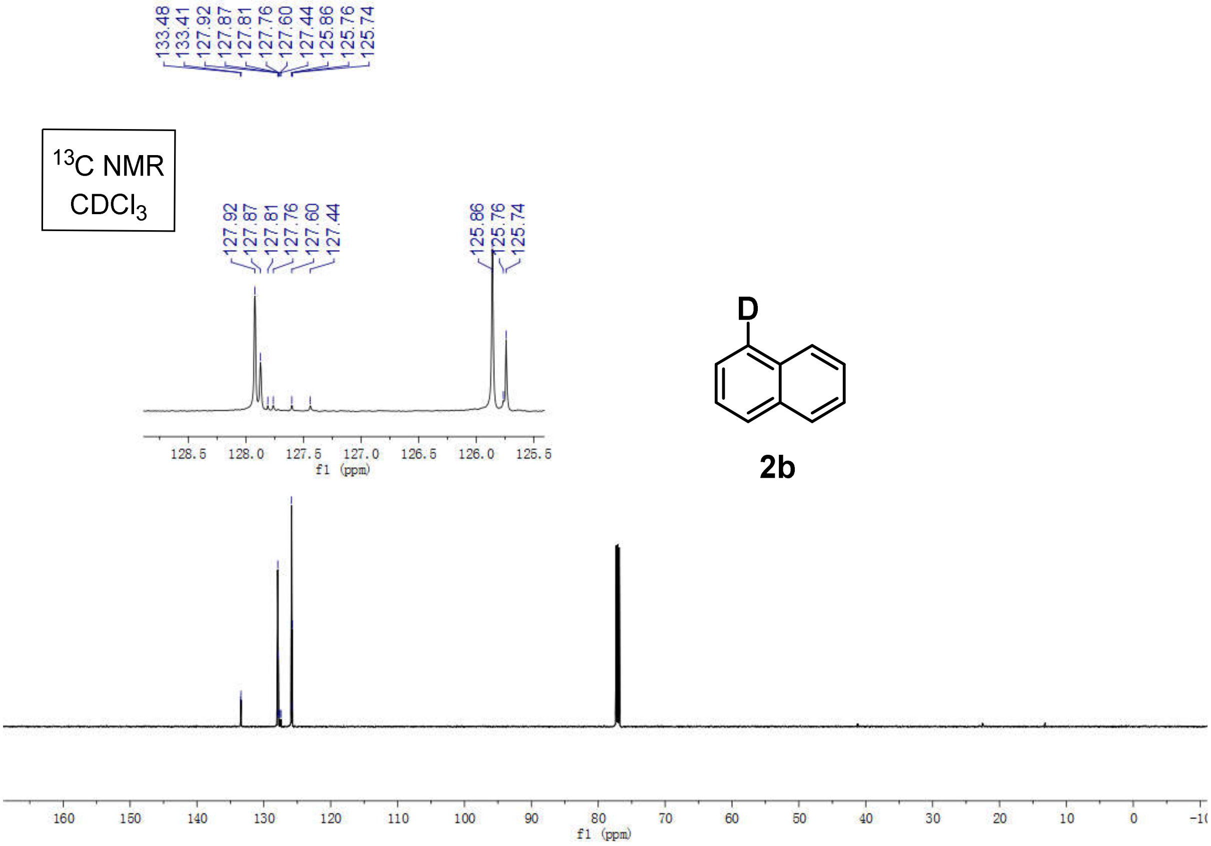


**2b**

**Supplementary Fig. 45** $^{1}$H NMR, $^{2}$H NMR and $^{13}$C NMR spectra of the compound **2b**.

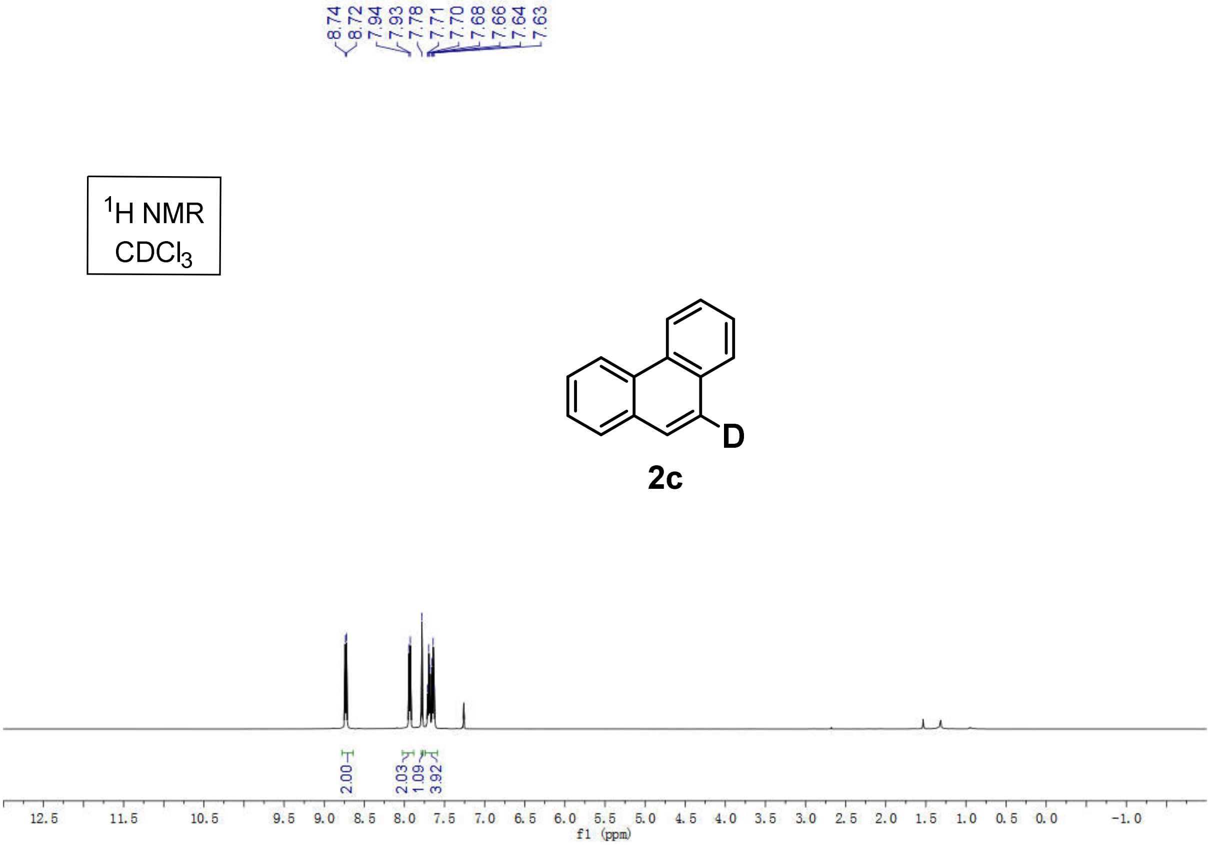
8.74
8.72
7.94
7.93
7.78
7.71
7.70
7.68
7.66
7.64
7.63
$^1$H NMR
$CDCl_3$
D
2c
2.00
2.03
1.09
3.92
12.5 11.5 10.5 9.5 9.0 8.5 8.0 7.5 7.0 6.5 6.0 5.5 5.0 4.5 4.0 3.5 3.0 2.5 2.0 1.5 1.0 0.5 0.0 -1.0
f1 (ppm)

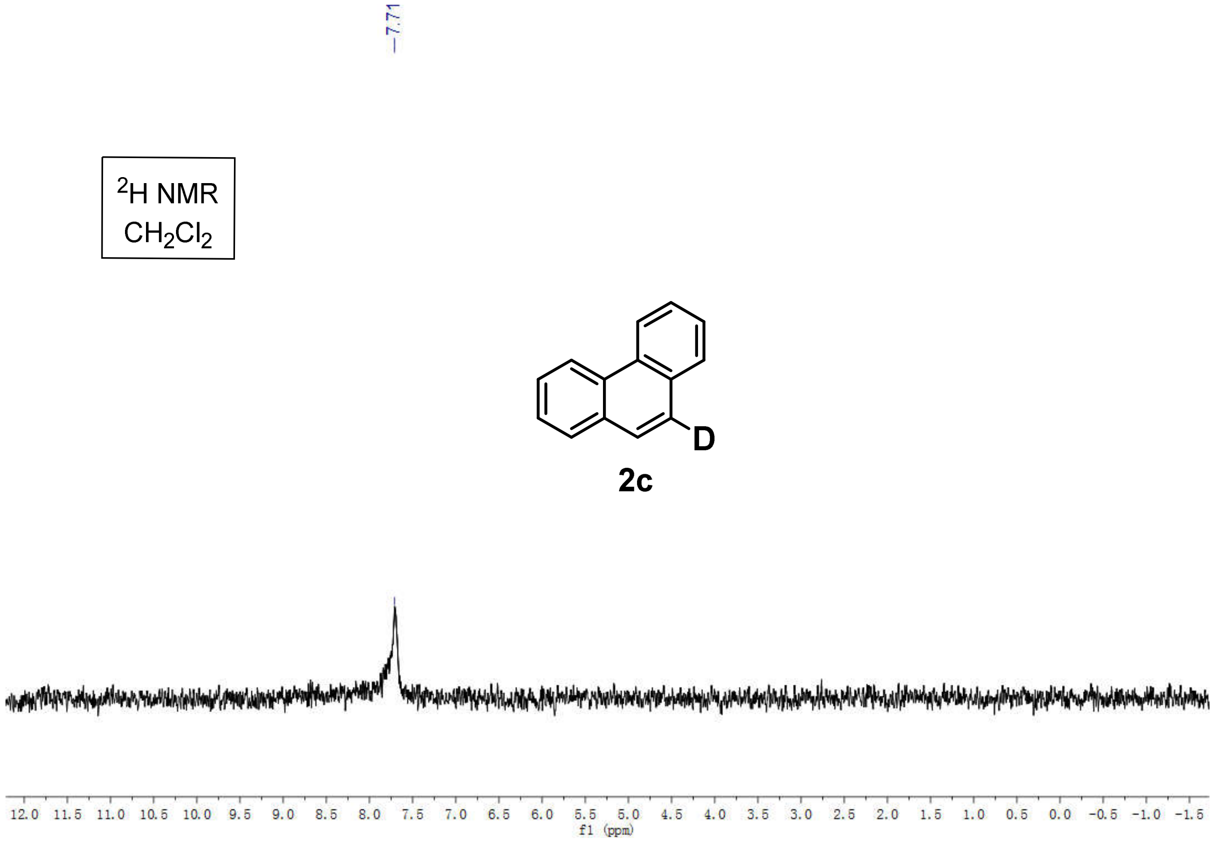
7.71
$^2$H NMR
$CH_2Cl_2$
D
2c
12.0 11.5 11.0 10.5 10.0 9.5 9.0 8.5 8.0 7.5 7.0 6.5 6.0 5.5 5.0 4.5 4.0 3.5 3.0 2.5 2.0 1.5 1.0 0.5 0.0 -0.5 -1.0 -1.5
f1 (ppm)

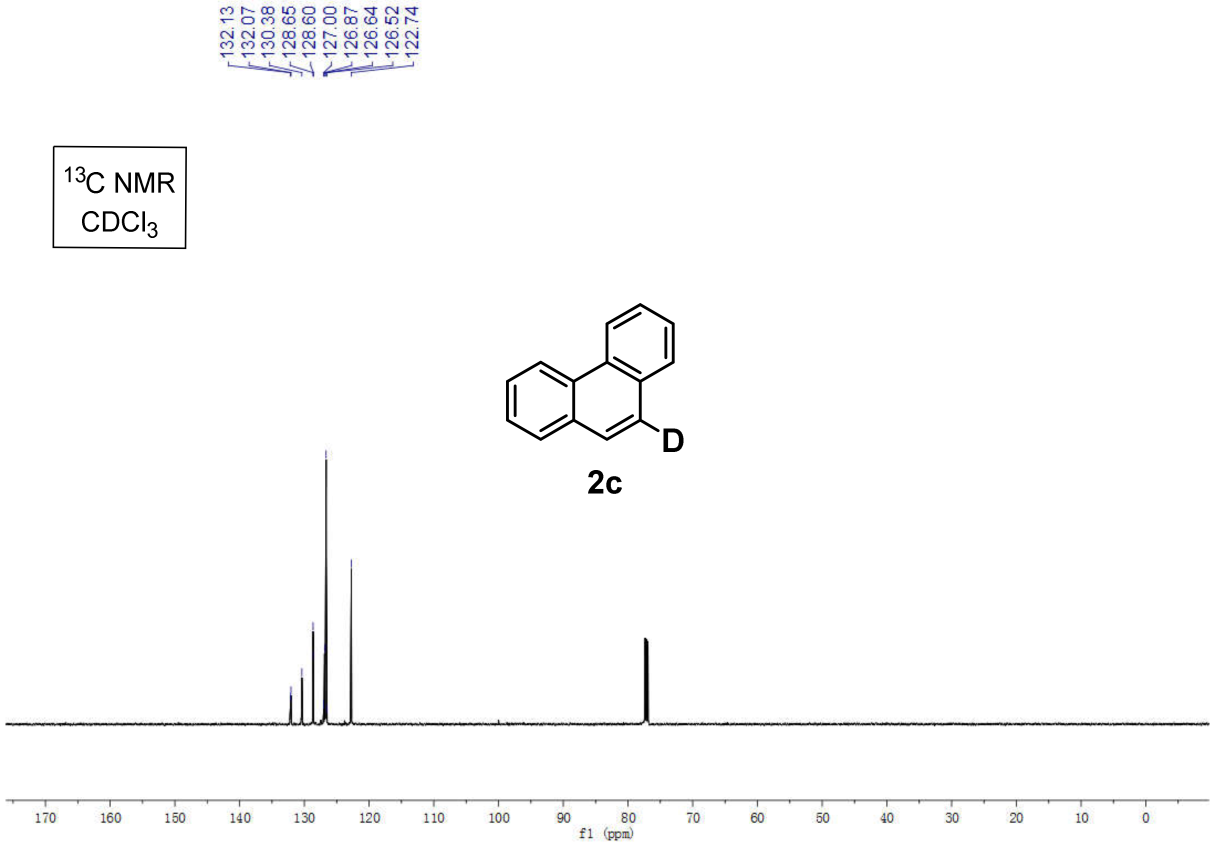


**Supplementary Fig. 46** $^{1}$H NMR, $^{2}$H NMR and $^{13}$C NMR spectra of the compound **2c**.

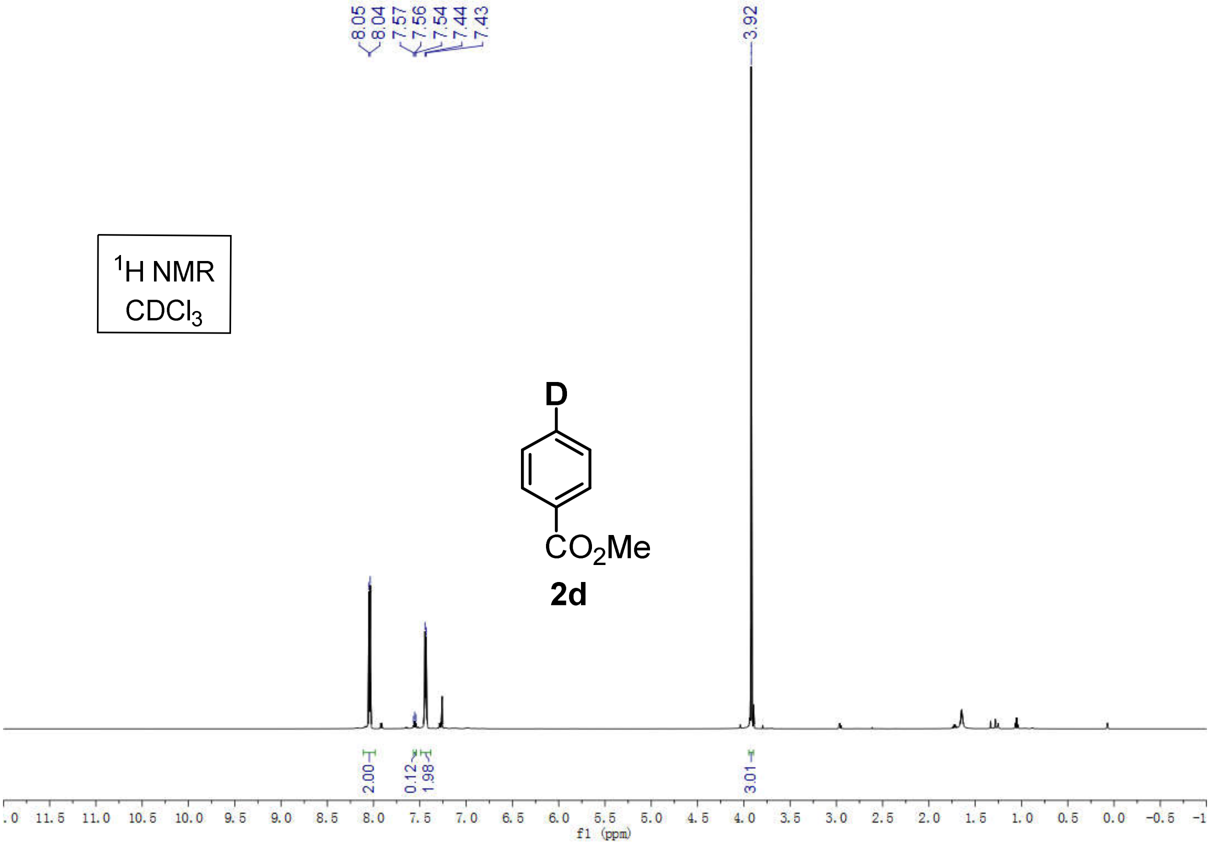
8.05
8.04
7.57
7.56
7.54
7.44
7.43
3.92
1H NMR
CDCl3
D
CO2Me
2d
2.00
0.12
1.98
3.01
f1 (ppm)

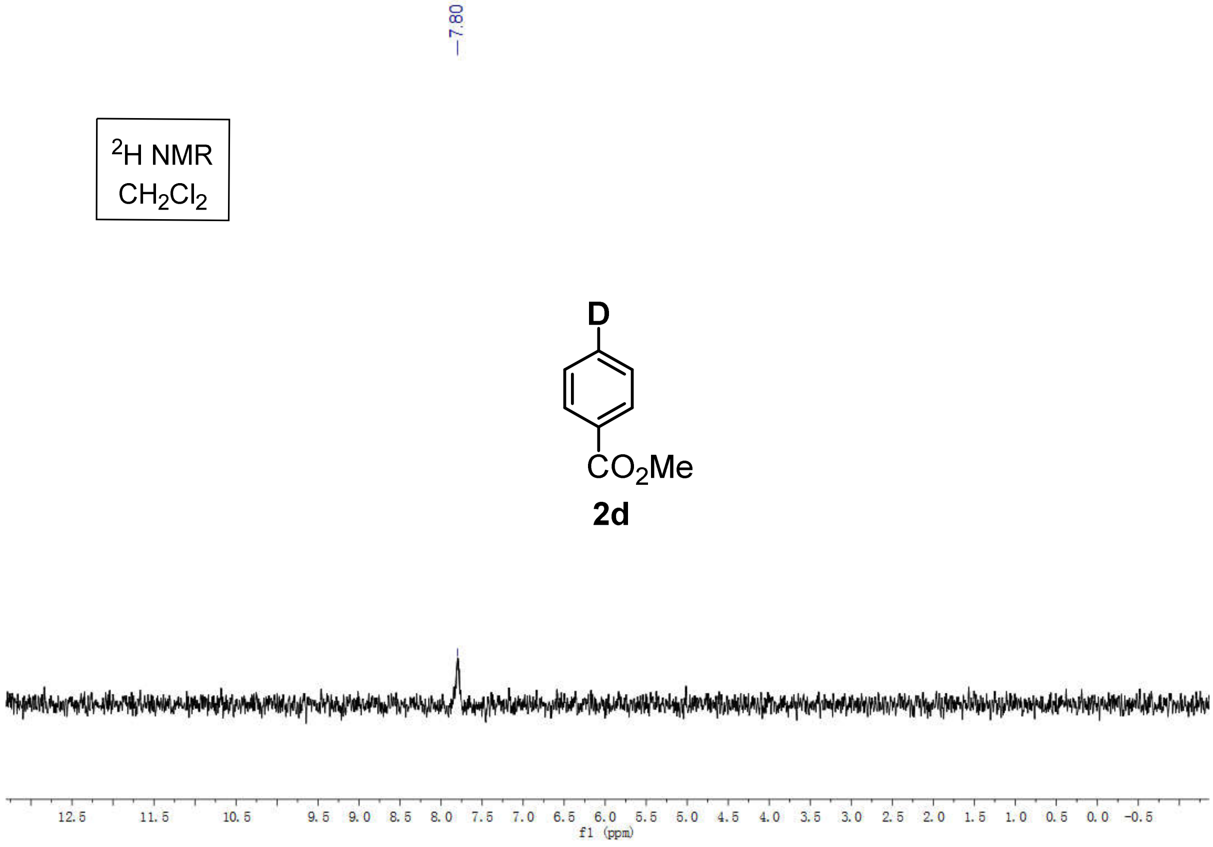
7.80
2H NMR
CH2Cl2
D
CO2Me
2d
f1 (ppm)

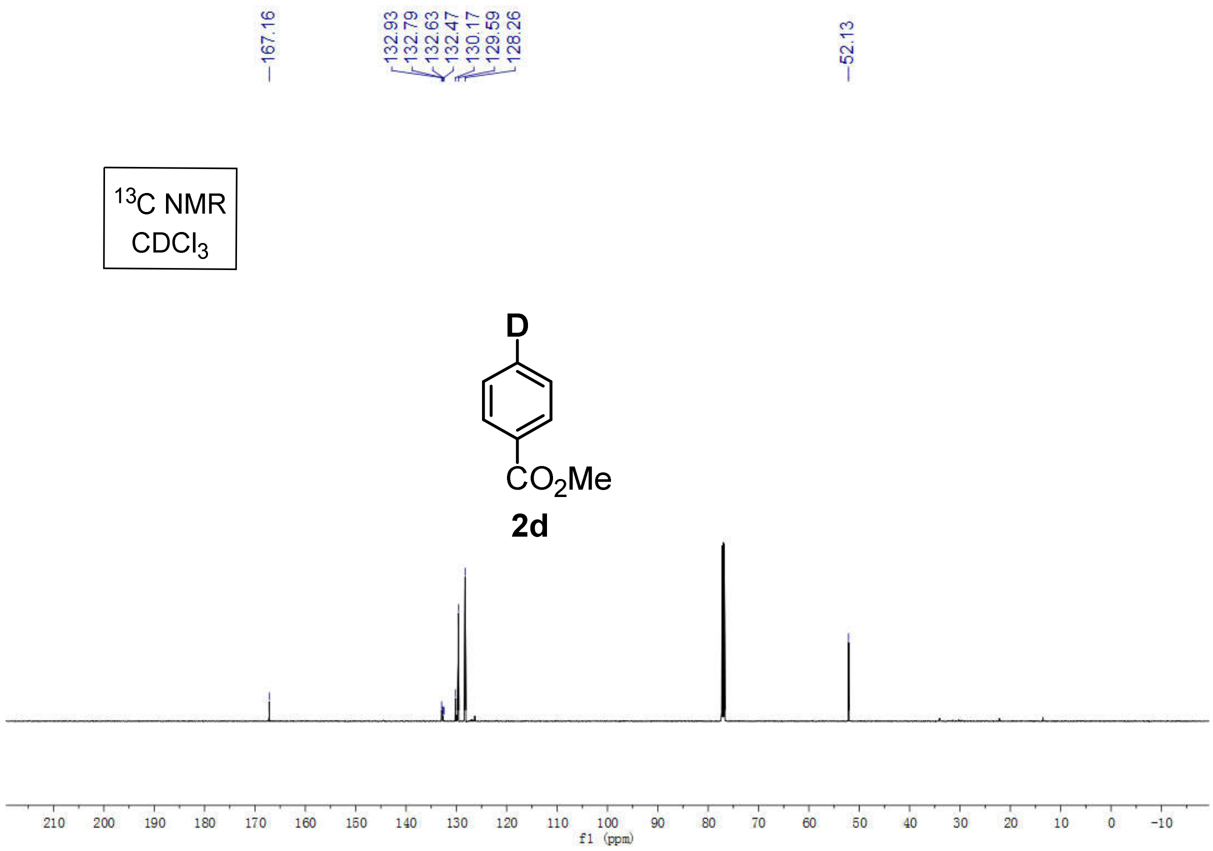


**Supplementary Fig. 47** $^{1}$H NMR, $^{2}$H NMR and $^{13}$C NMR spectra of the compound **2d**.

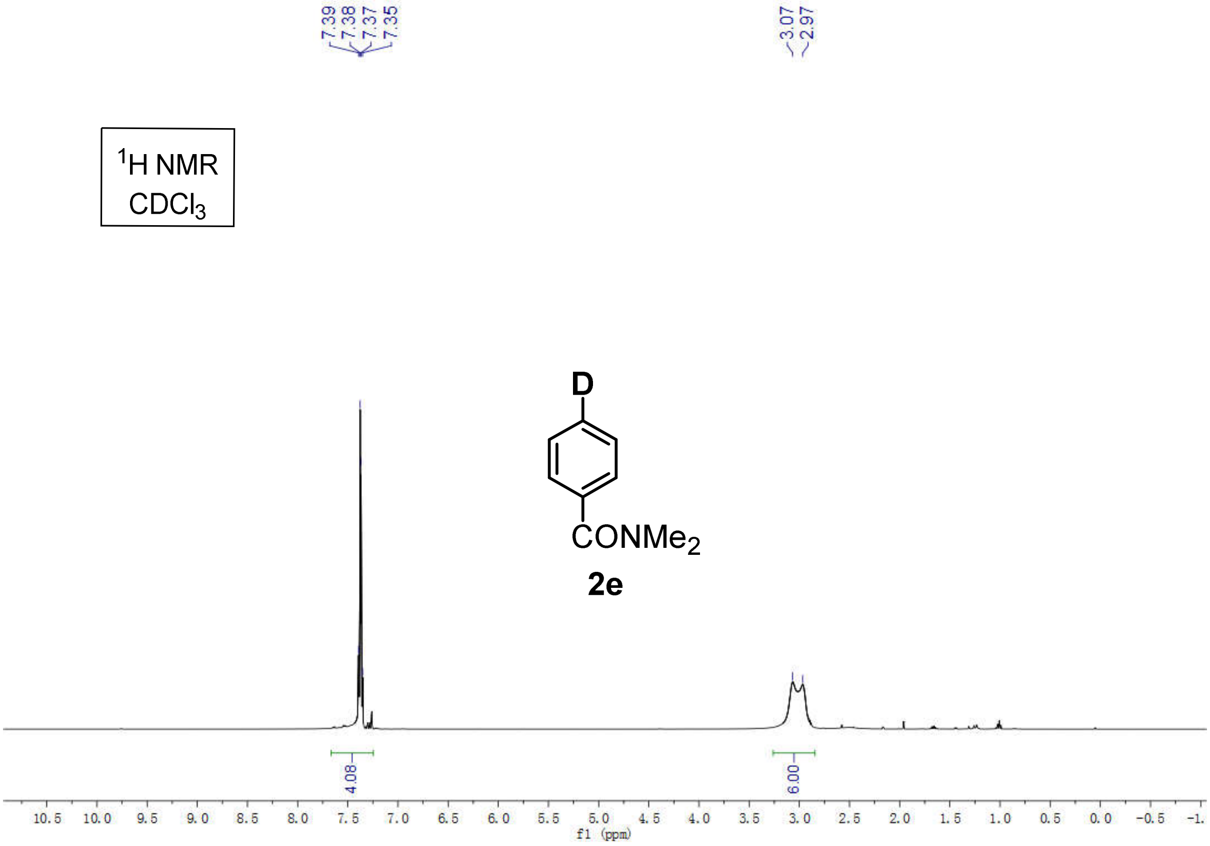
7.39
7.38
7.37
7.35
3.07
2.97
1H NMR
CDCl3
D
CONMe2
2e
4.08
6.00
f1 (ppm)

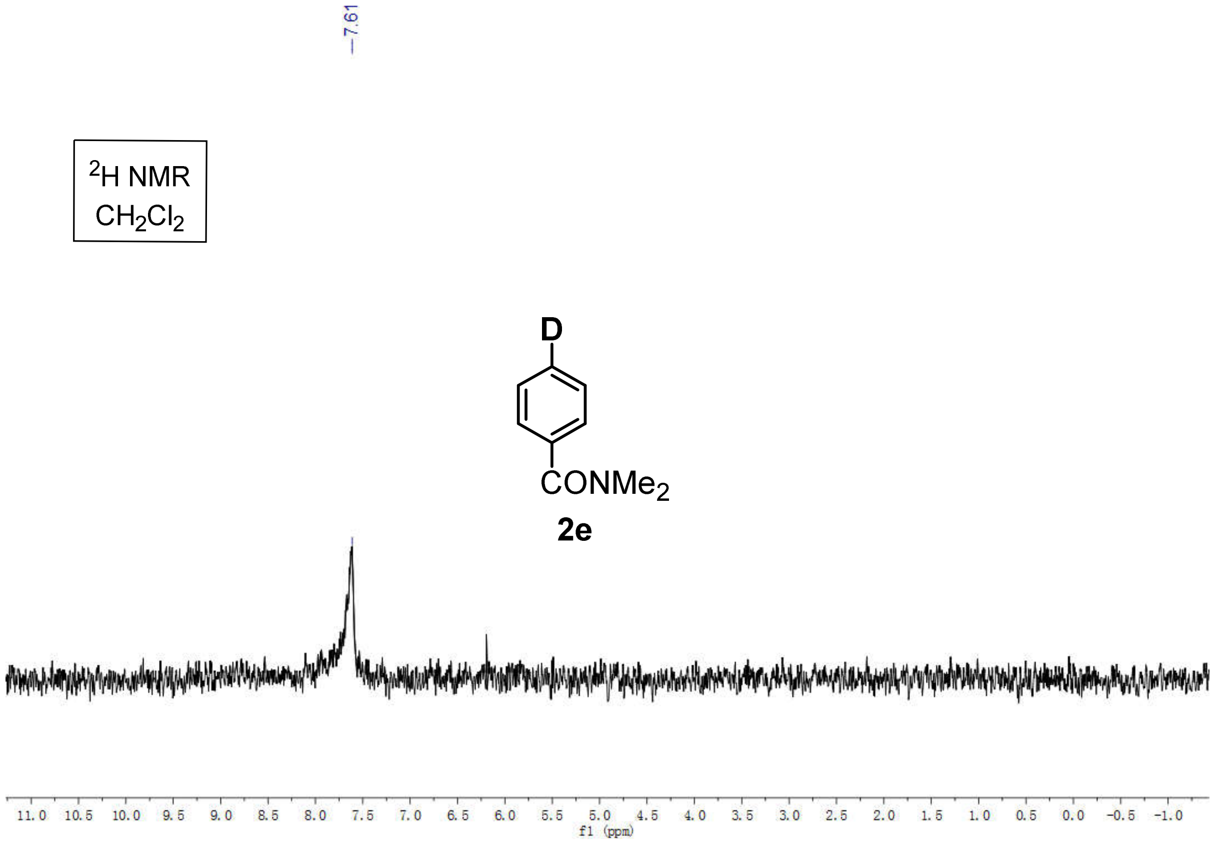
7.61
2H NMR
CH2Cl2
D
CONMe2
2e
f1 (ppm)

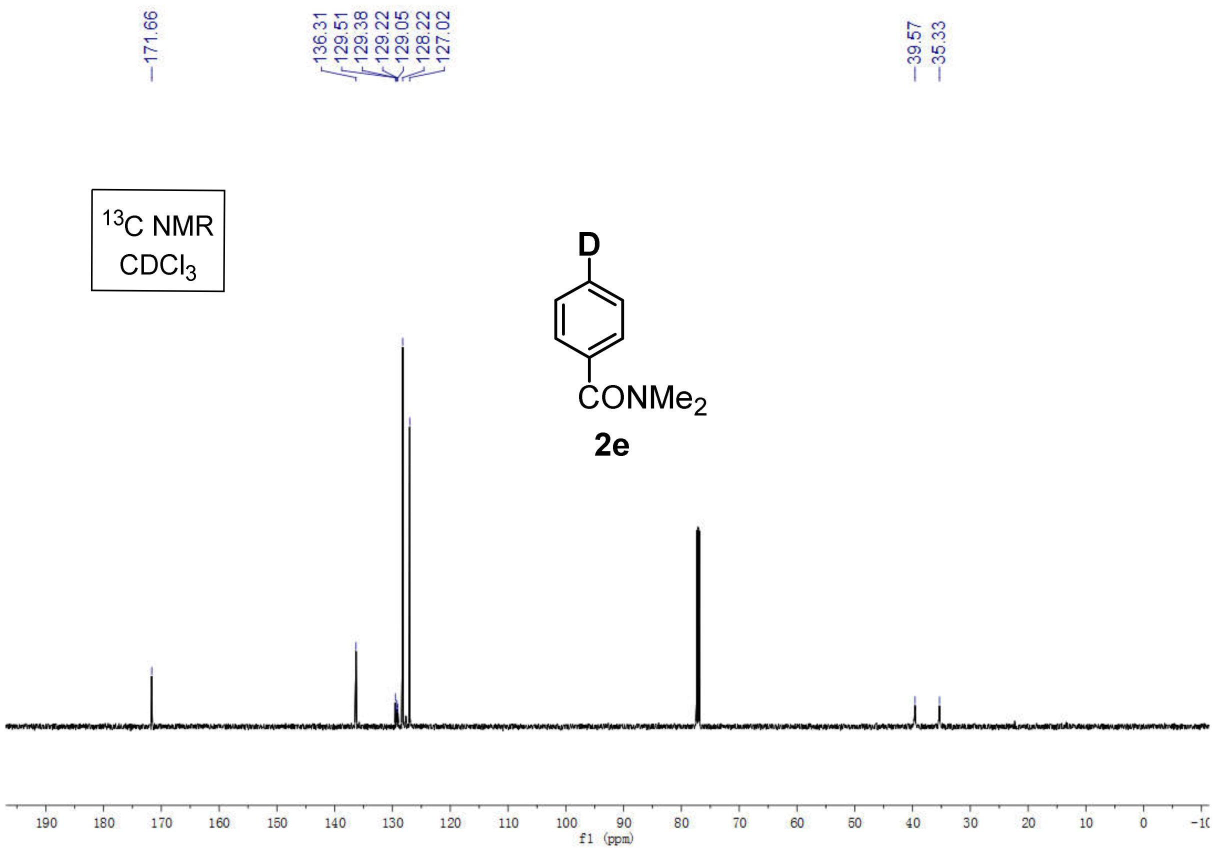


**Supplementary Fig. 48** $^{1}$H NMR, $^{2}$H NMR and $^{13}$C NMR spectra of the compound **2e**.

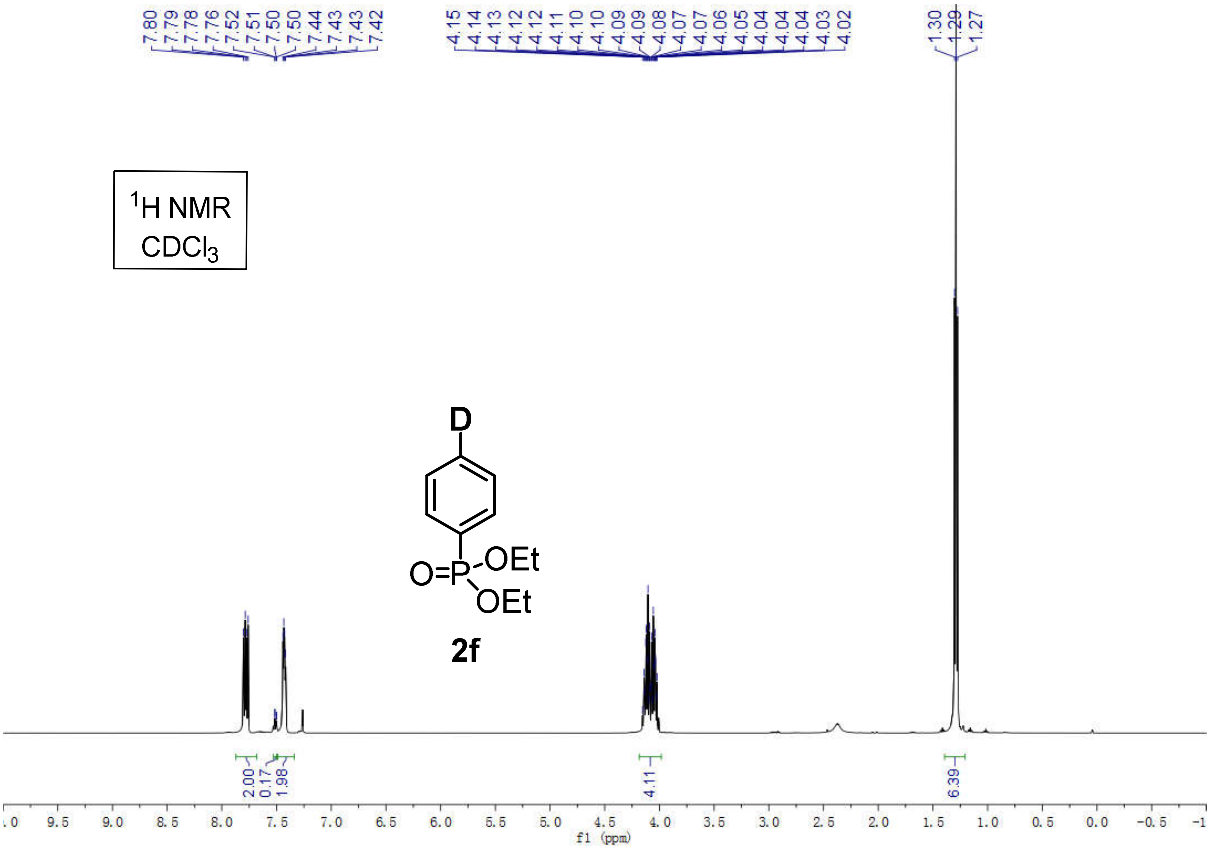

7.80
7.79
7.78
7.76
7.52
7.51
7.50
7.50
7.44
7.43
7.43
7.42
4.15
4.14
4.13
4.12
4.12
4.11
4.10
4.10
4.09
4.09
4.08
4.07
4.07
4.06
4.05
4.04
4.04
4.04
4.03
4.02
1.30
1.29
1.27
1H NMR
CDCl3
D
O=P
OEt
OEt
2f
2.00
0.17
1.98
4.11
6.39
f1 (ppm)


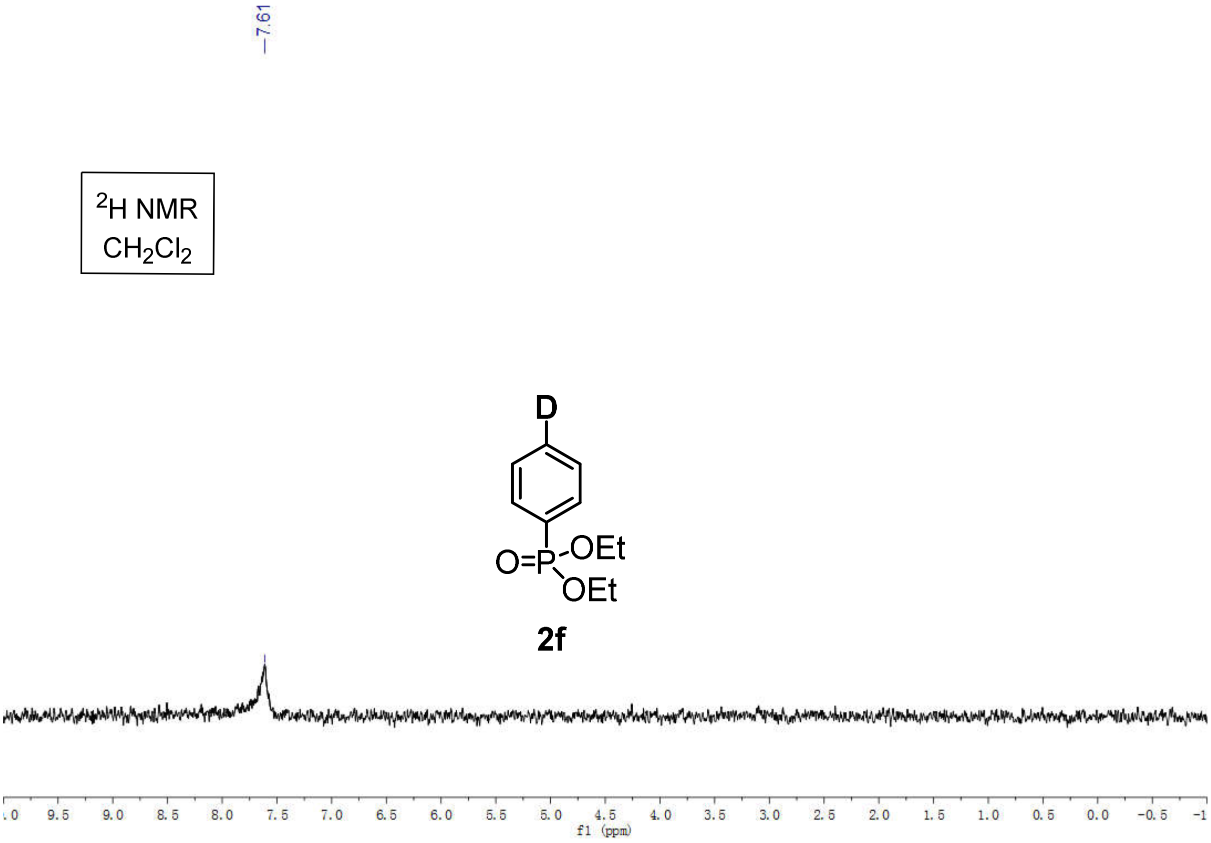

7.61
2H NMR
CH2Cl2
D
O=P
OEt
OEt
2f
f1 (ppm)

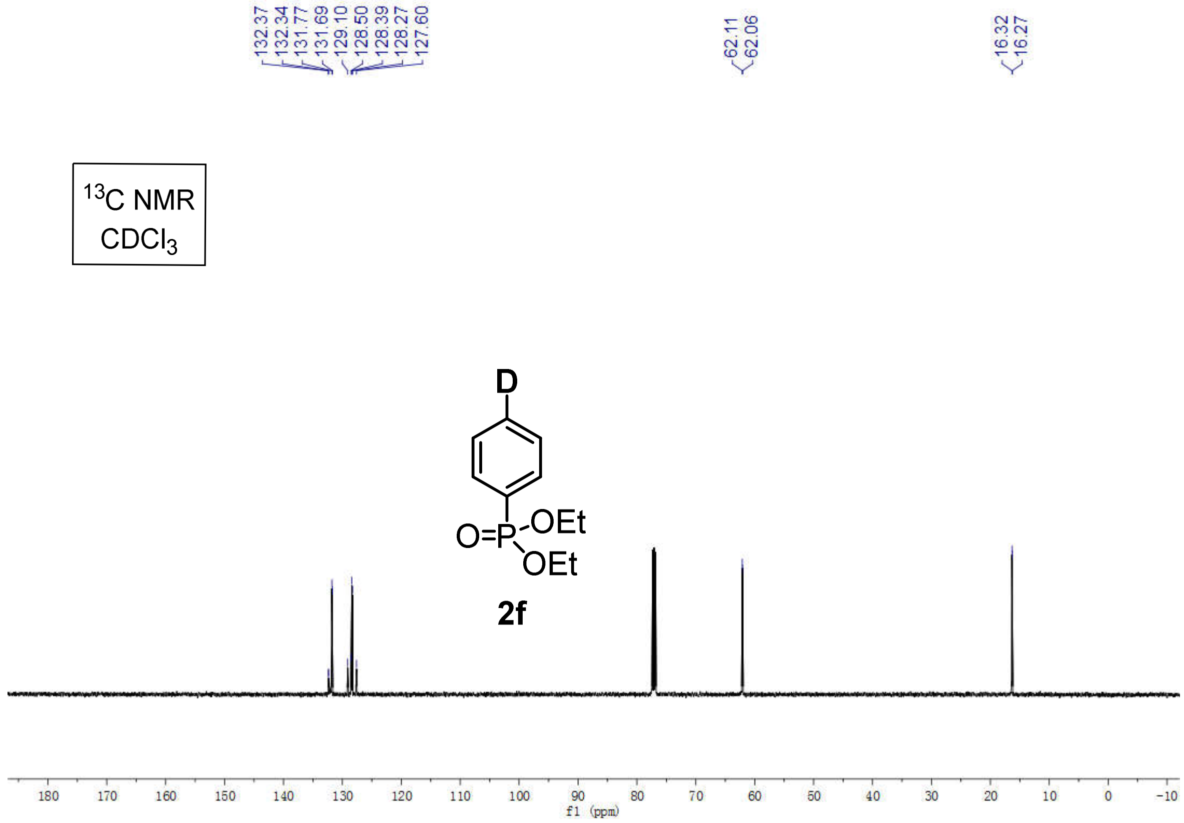


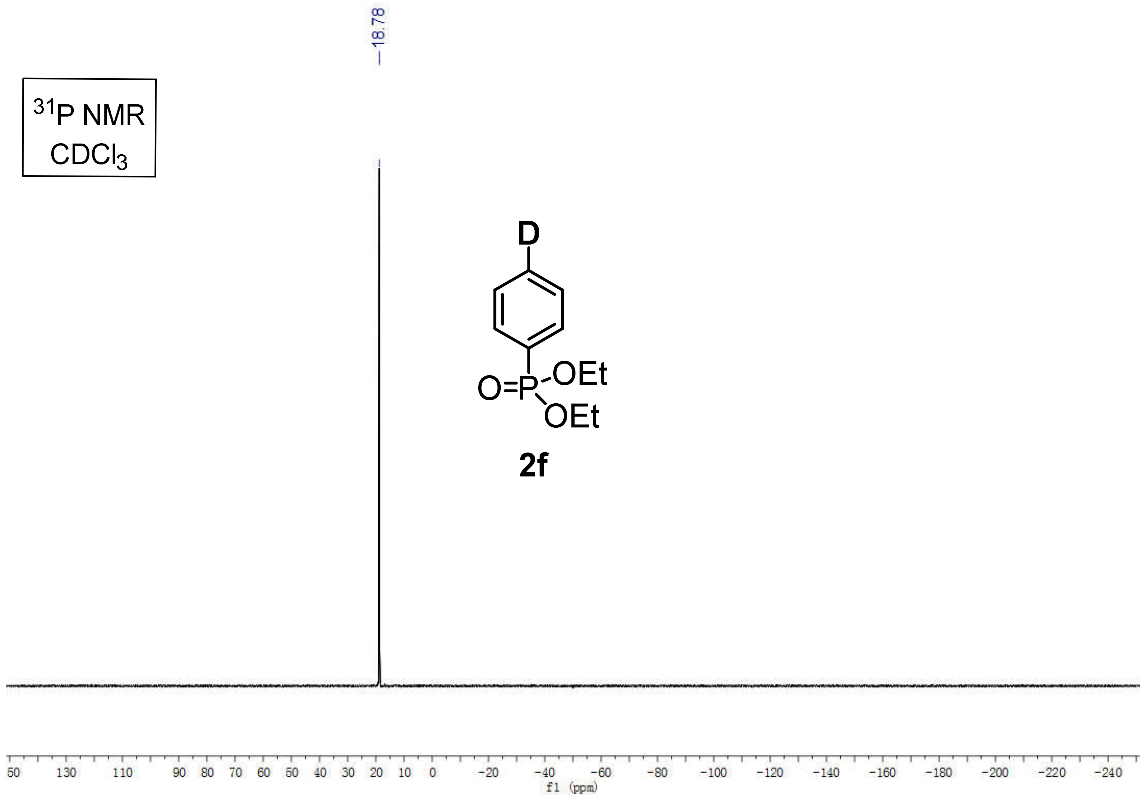


**Supplementary Fig. 49** $^{1}$H NMR, $^{2}$H NMR, $^{13}$C NMR and $^{31}$P NMR spectra of the compound **2f**.

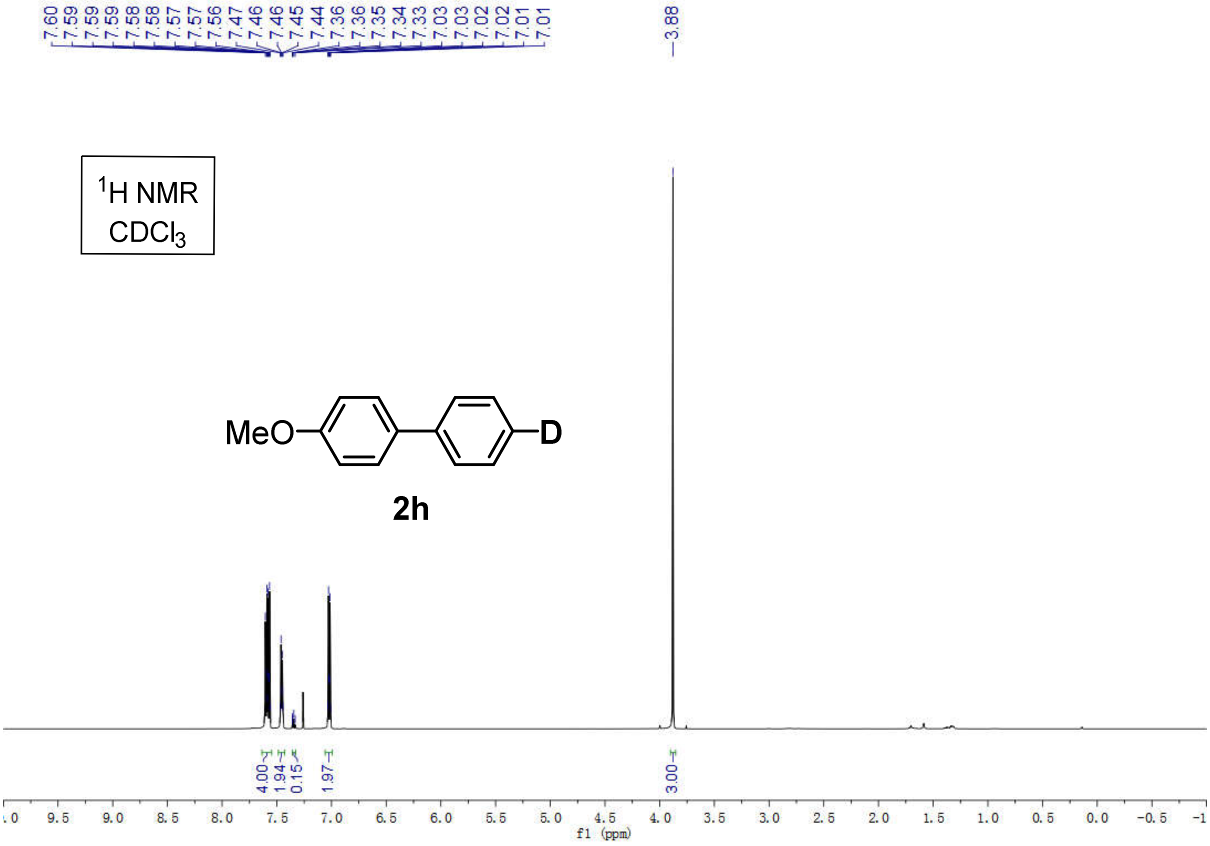

7.60
7.59
7.59
7.59
7.58
7.58
7.57
7.57
7.56
7.47
7.46
7.46
7.45
7.44
7.36
7.36
7.35
7.34
7.33
7.03
7.03
7.02
7.02
7.01
7.01
3.88
1H NMR
CDCl3
MeO
D
2h
4.00
1.94
0.15
1.97
3.00
f1 (ppm)


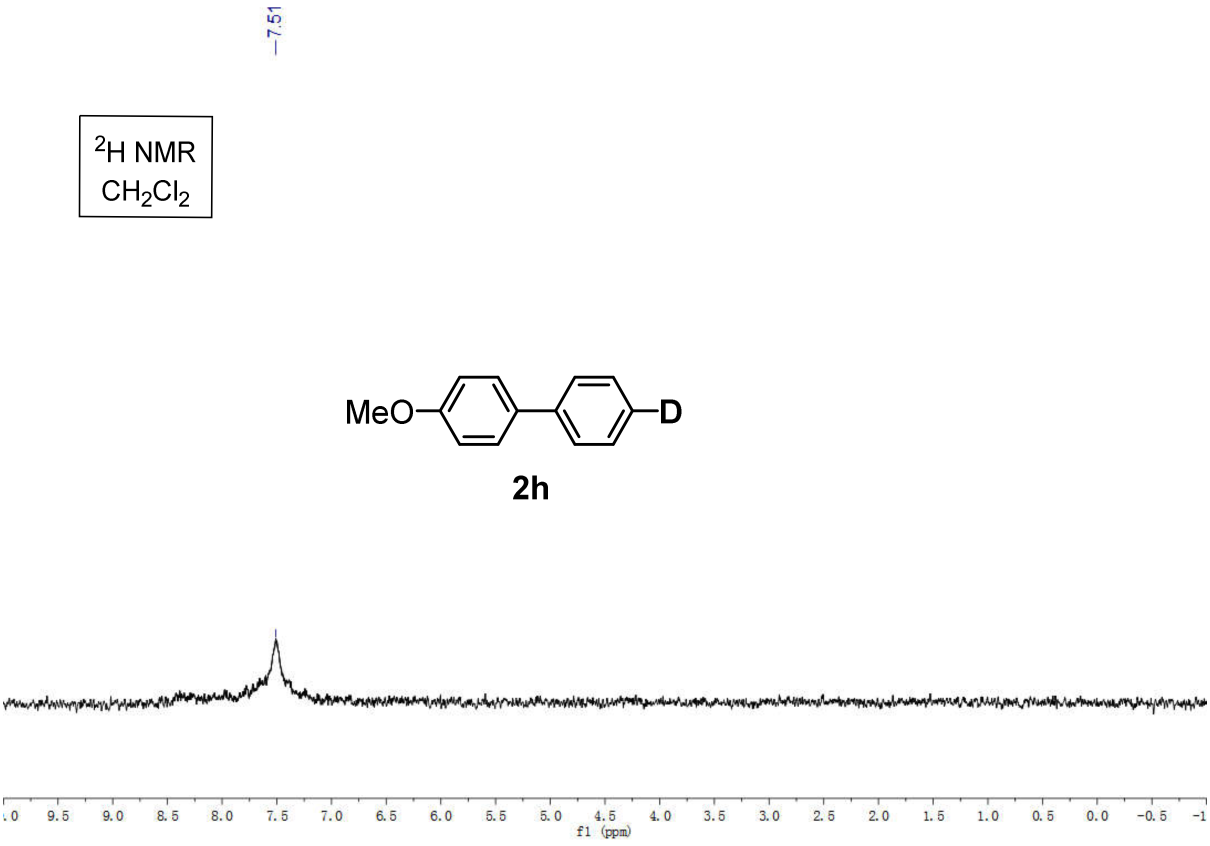

7.51
2H NMR
CH2Cl2
MeO
D
2h
f1 (ppm)

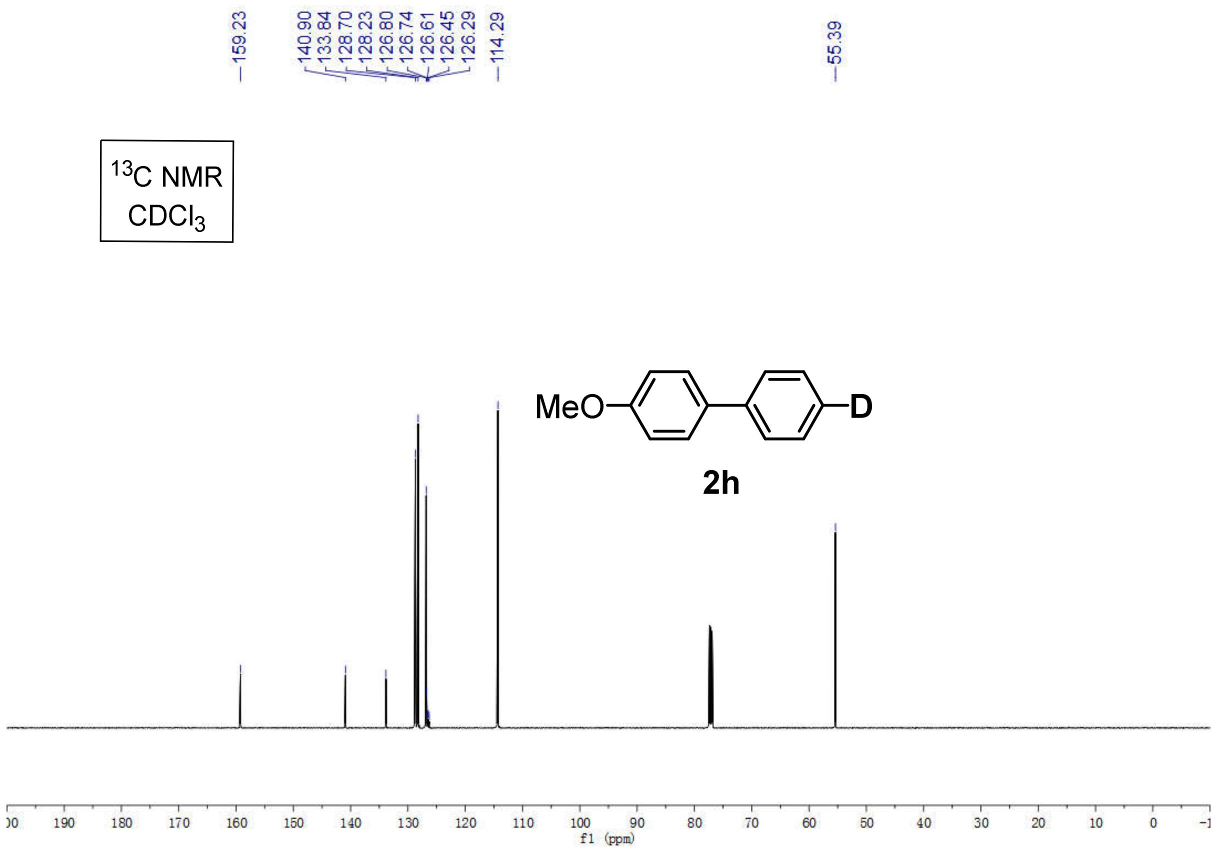


**Supplementary Fig. 50** $^{1}$H NMR, $^{2}$H NMR and $^{13}$C NMR spectra of the compound **2h**.

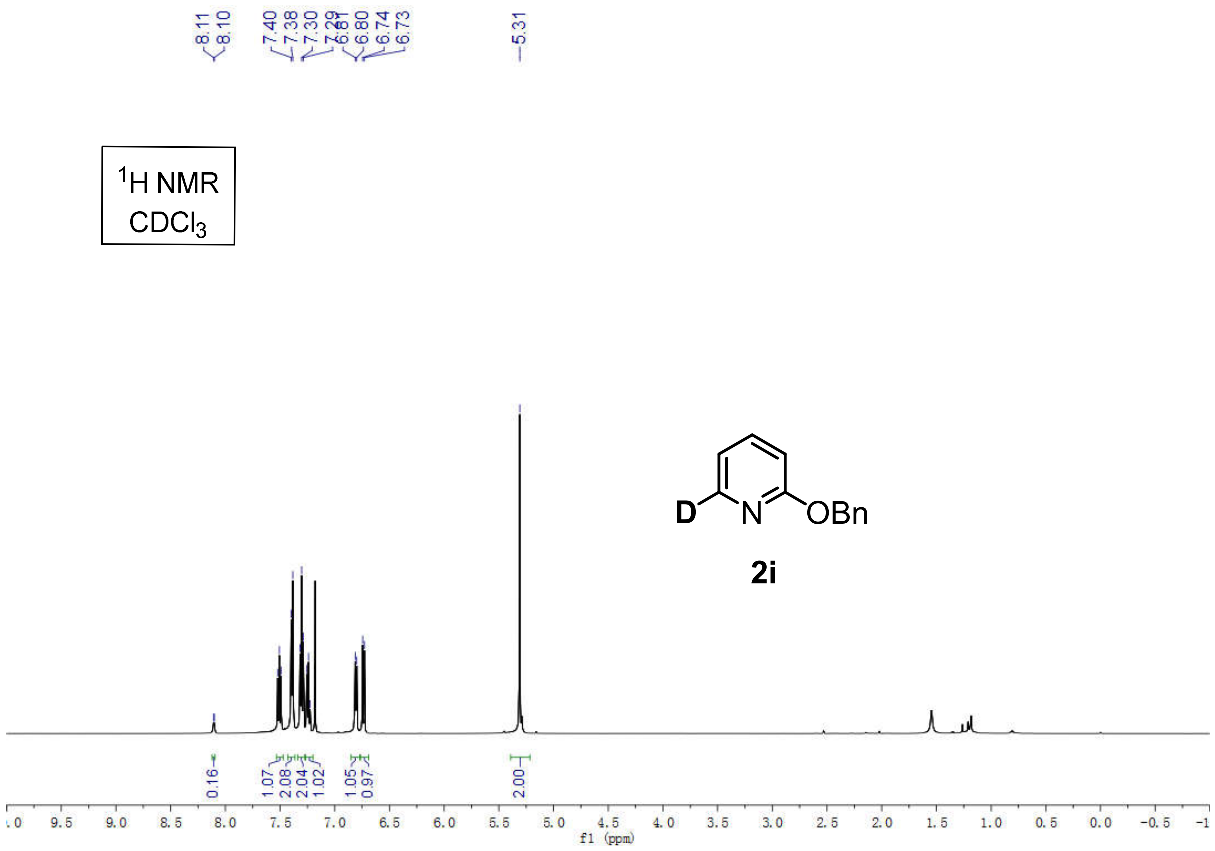
8.11
8.10
7.40
7.38
7.30
7.29
6.81
6.80
6.74
6.73
5.31
$^1H$ NMR
$CDCl_3$
D
N
OBn
2i
0.16
1.07
2.08
2.04
1.02
1.05
0.97
2.00
f1 (ppm)

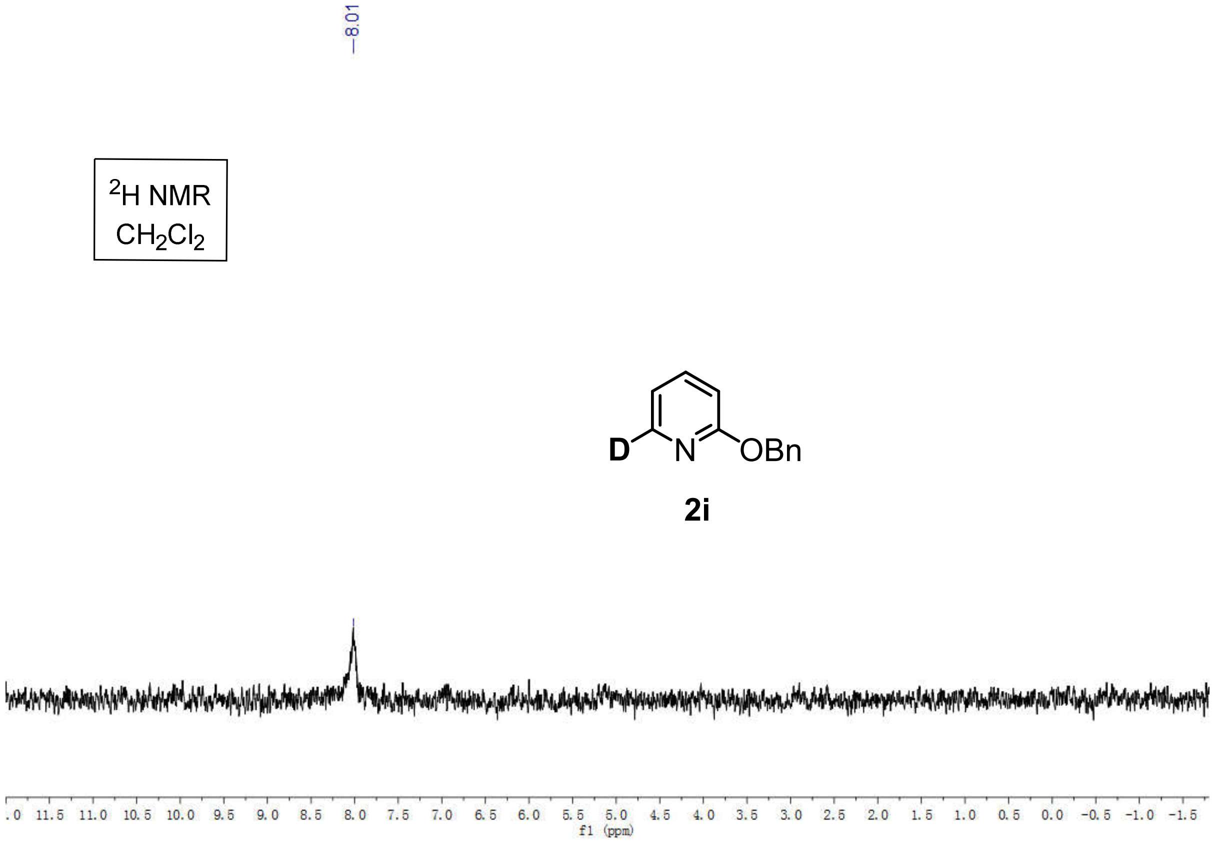
8.01
$^2H$ NMR
$CH_2Cl_2$
D
N
OBn
2i
f1 (ppm)

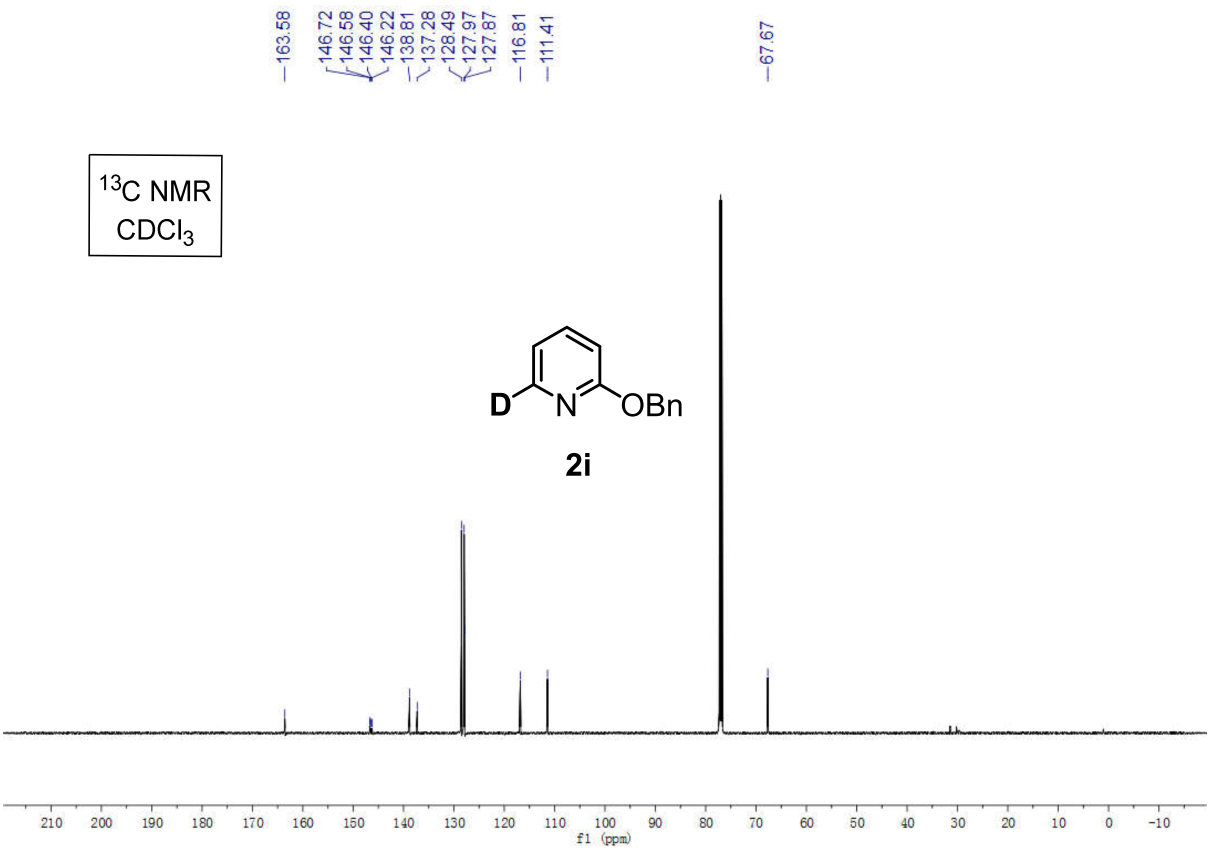


**Supplementary Fig. 51** $^{1}$H NMR, $^{2}$H NMR and $^{13}$C NMR spectra of the compound **2i**.

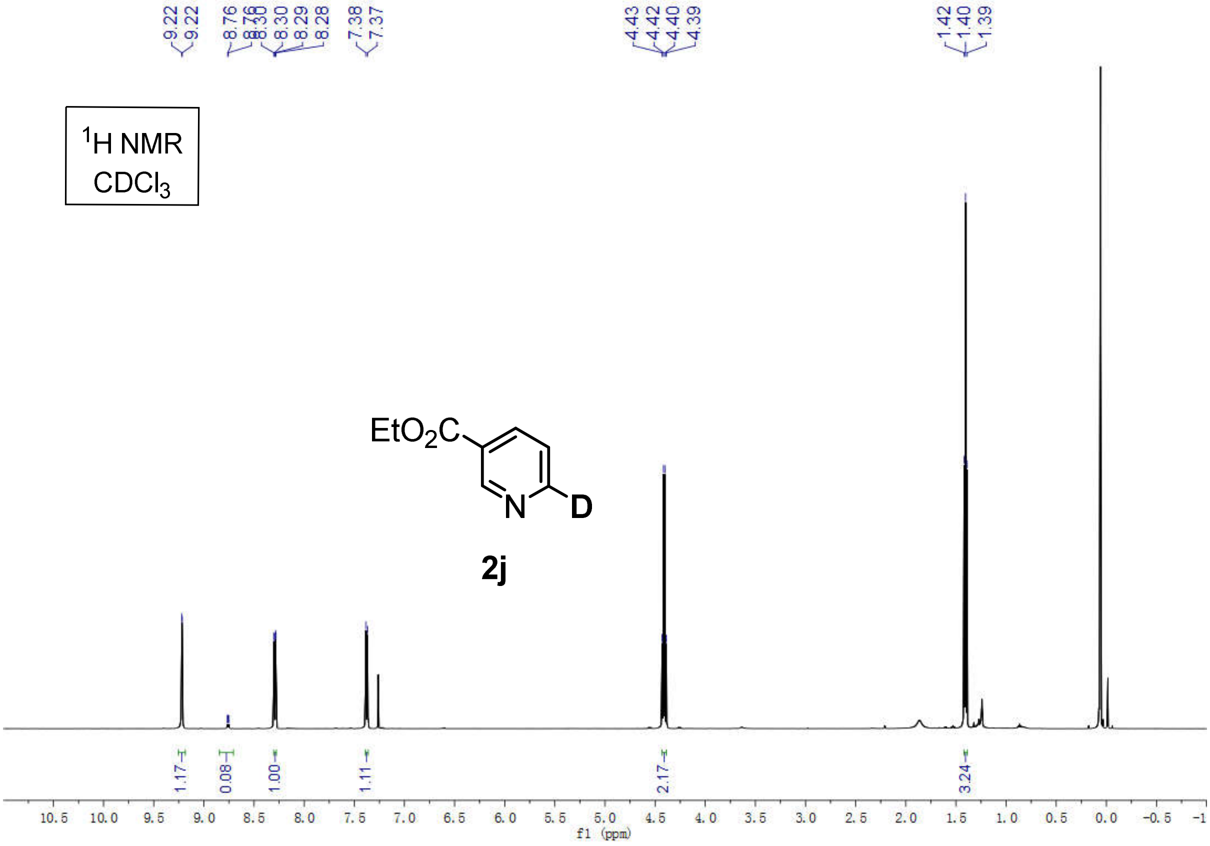
9.22
9.22
8.76
8.76
8.30
8.30
8.29
8.28
7.38
7.37
4.43
4.42
4.40
4.39
1.42
1.40
1.39
1H NMR
CDCl3
EtO2C
N
D
2j
1.17
0.08
1.00
1.11
2.17
3.24
f1 (ppm)

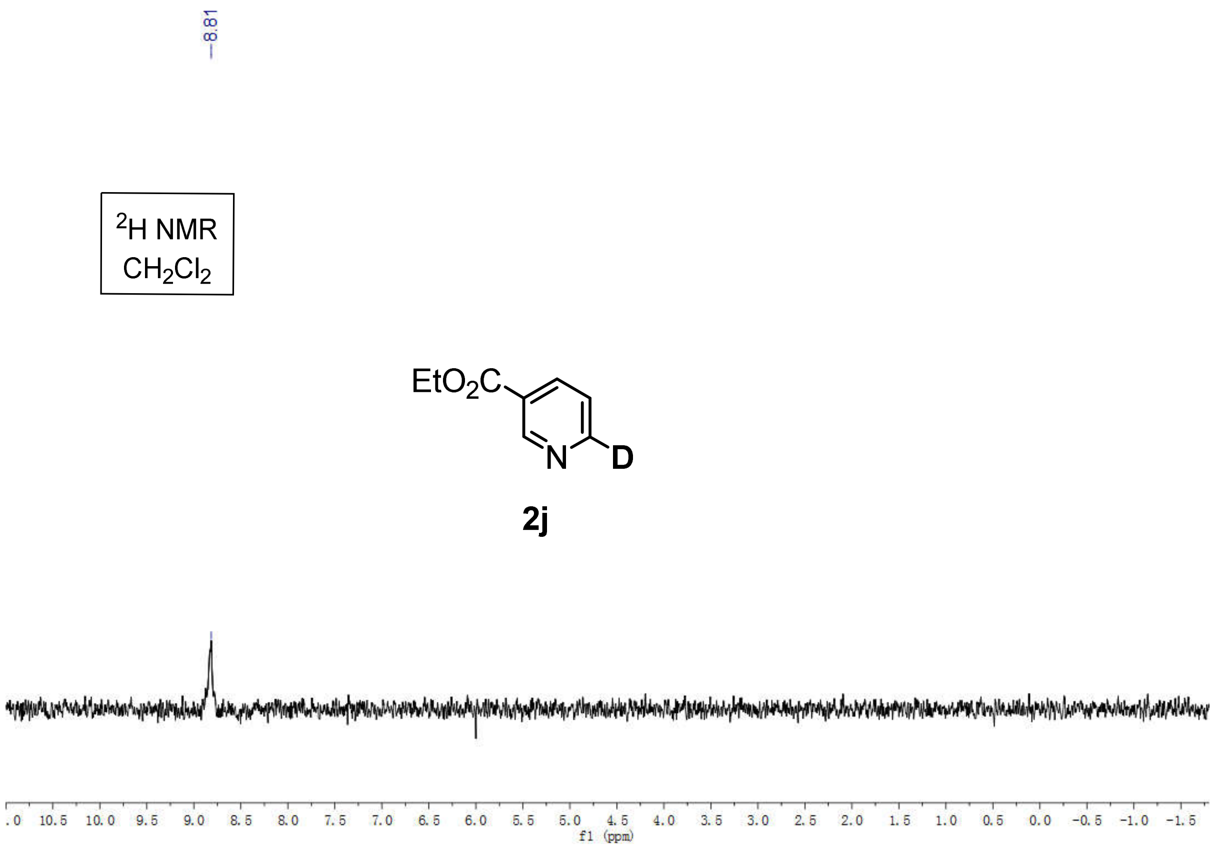
8.81
2H NMR
CH2Cl2
EtO2C
N
D
2j
f1 (ppm)

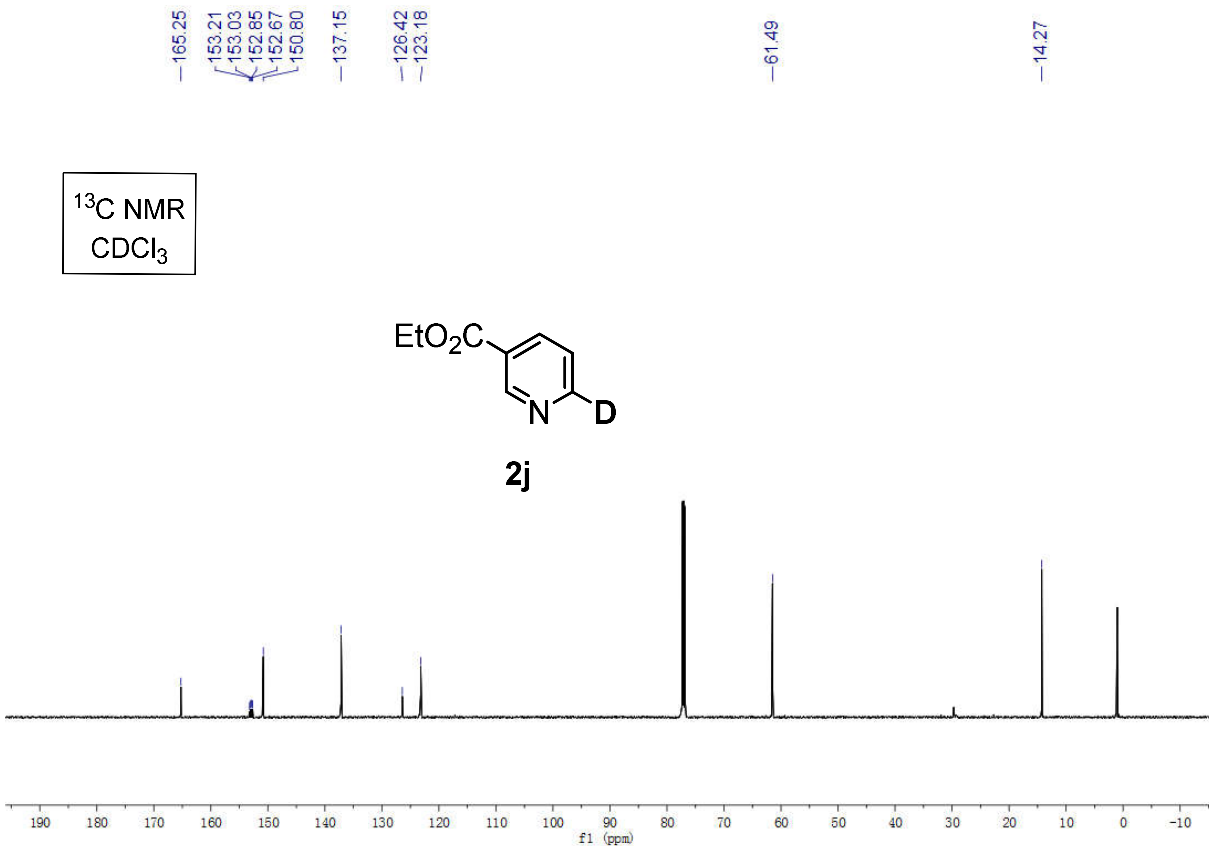


**Supplementary Fig. 52** $^{1}$H NMR, $^{2}$H NMR and $^{13}$C NMR spectra of the compound **2j**.

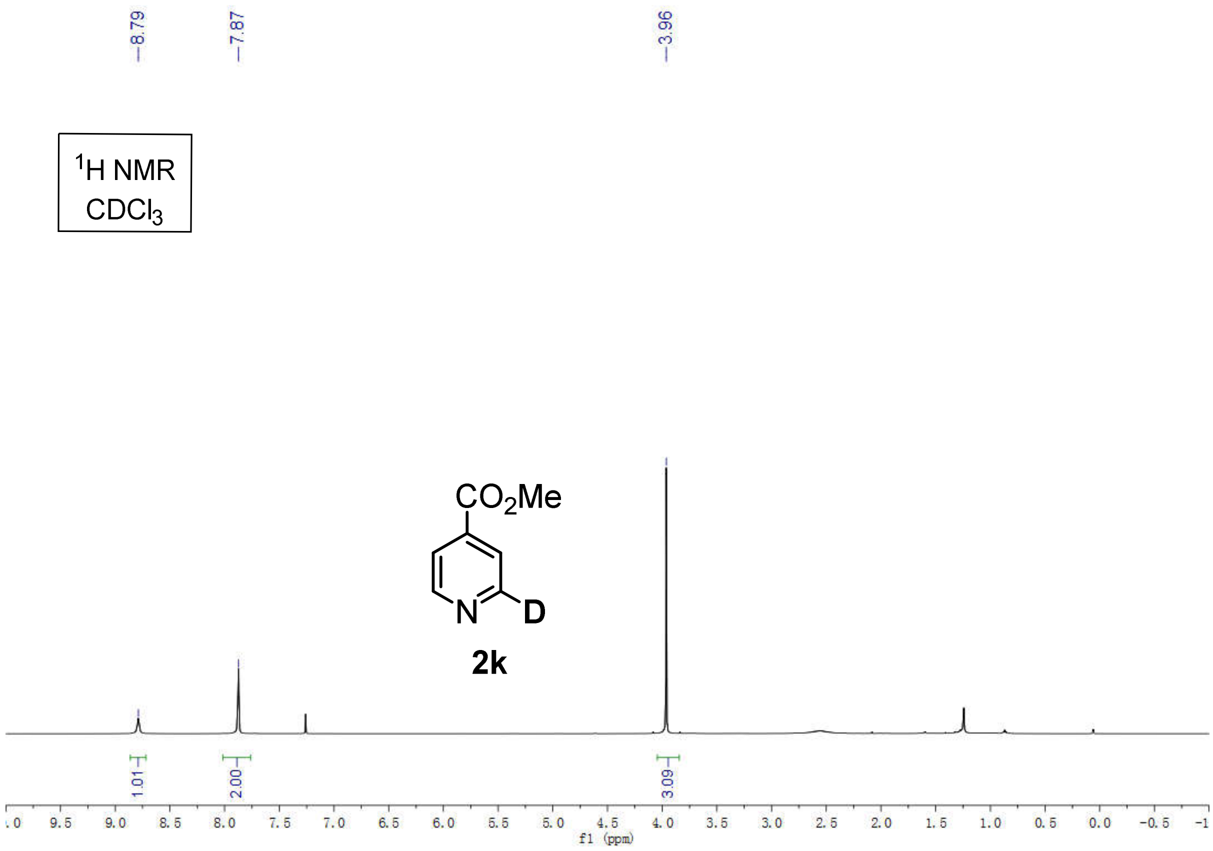
8.79
7.87
3.96
1H NMR
CDCl3
CO2Me
N
D
2k
1.01
2.00
3.09
f1 (ppm)

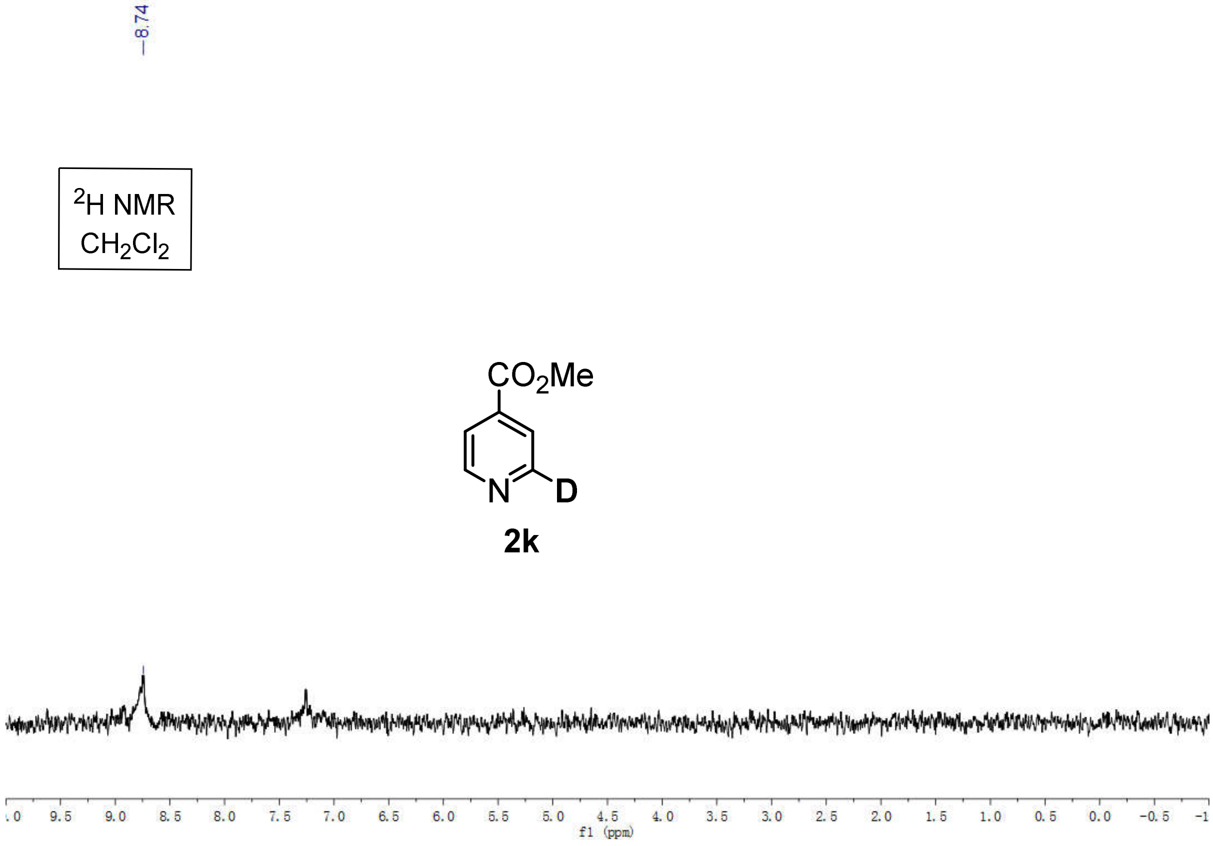
8.74
2H NMR
CH2Cl2
CO2Me
N
D
2k
f1 (ppm)

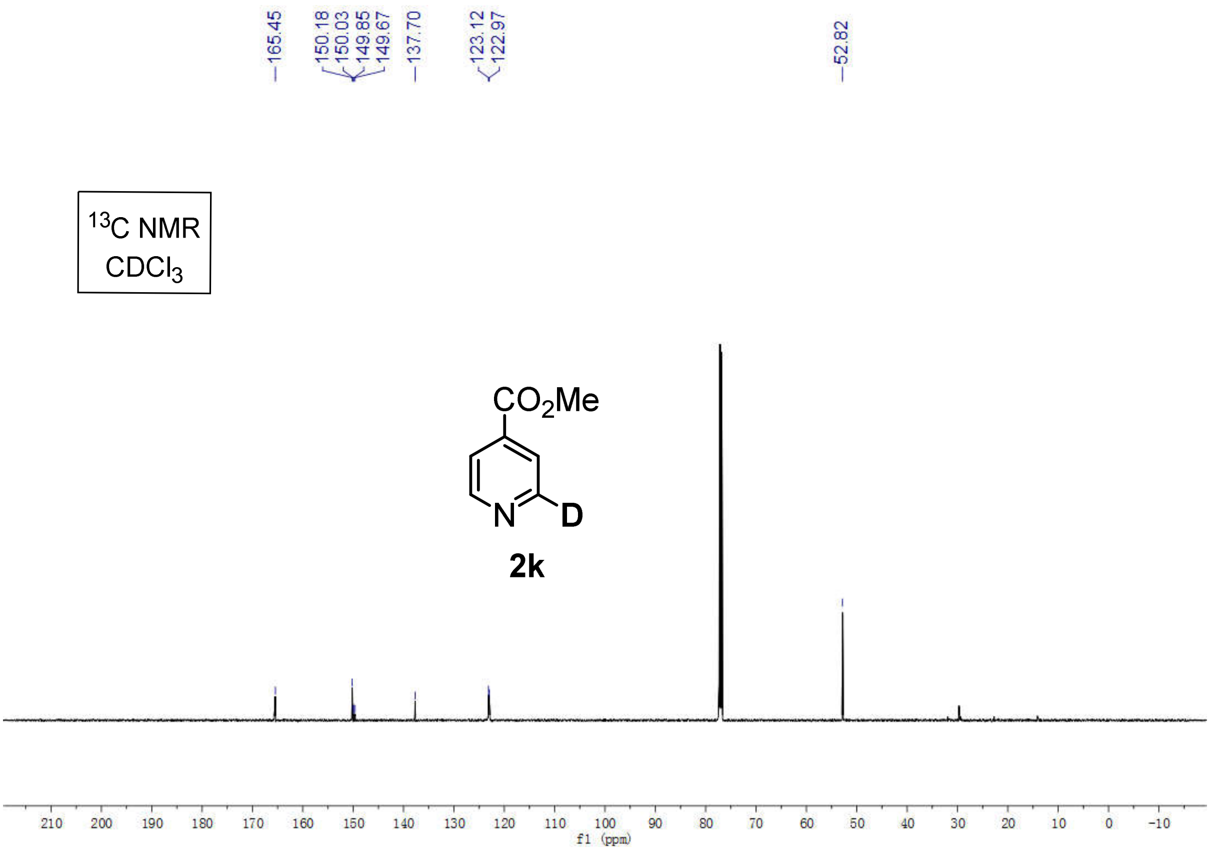


**Supplementary Fig. 53** $^{1}$H NMR, $^{2}$H NMR and $^{13}$C NMR spectra of the compound **2k**.

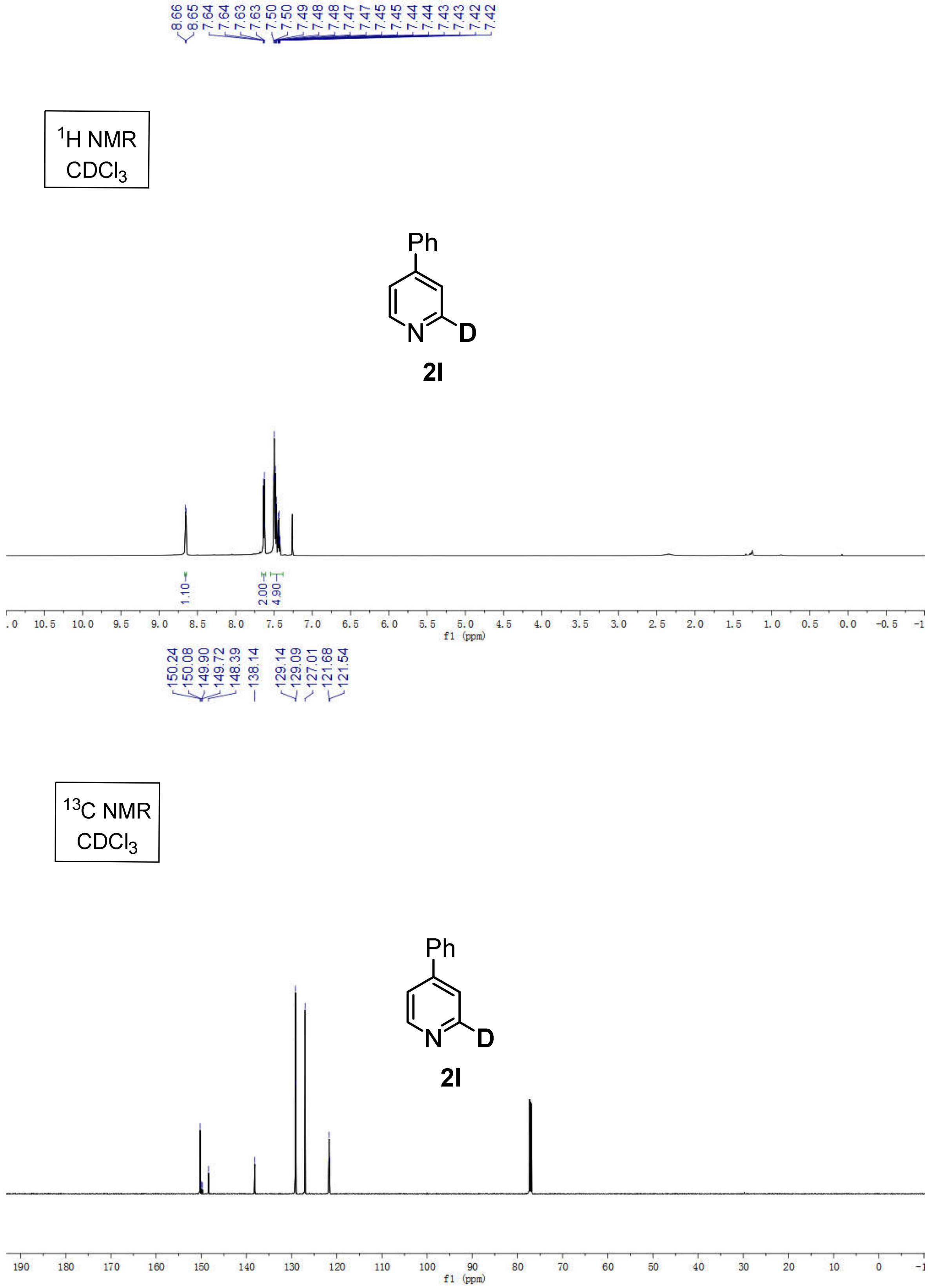


**Supplementary Fig. 54** $^{1}H$ NMR and $^{13}C$ NMR spectra of the compound **2l**.

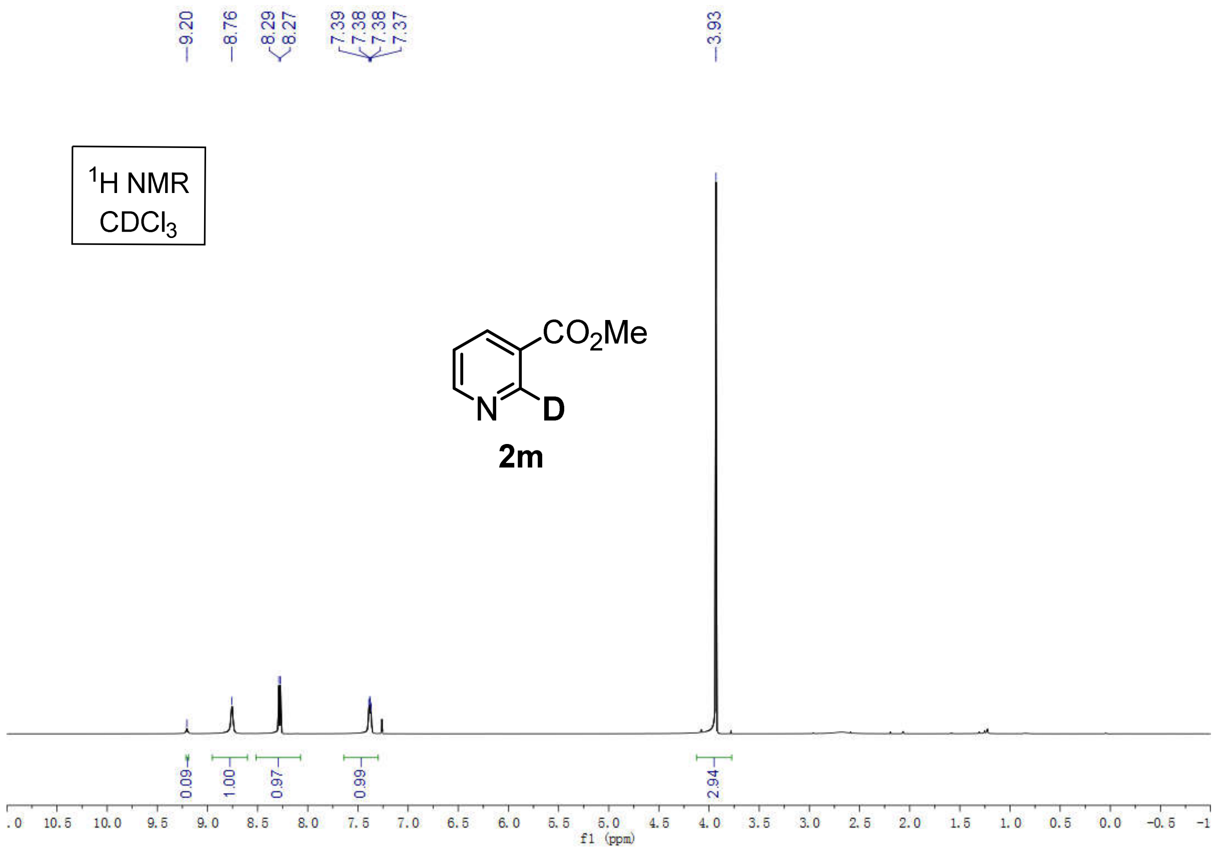

9.20
8.76
8.29
8.27
7.39
7.38
7.38
7.37
3.93
$^1$H NMR
$CDCl_3$
$CO_2Me$
N
D
2m
0.09
1.00
0.97
0.99
2.94
f1 (ppm)


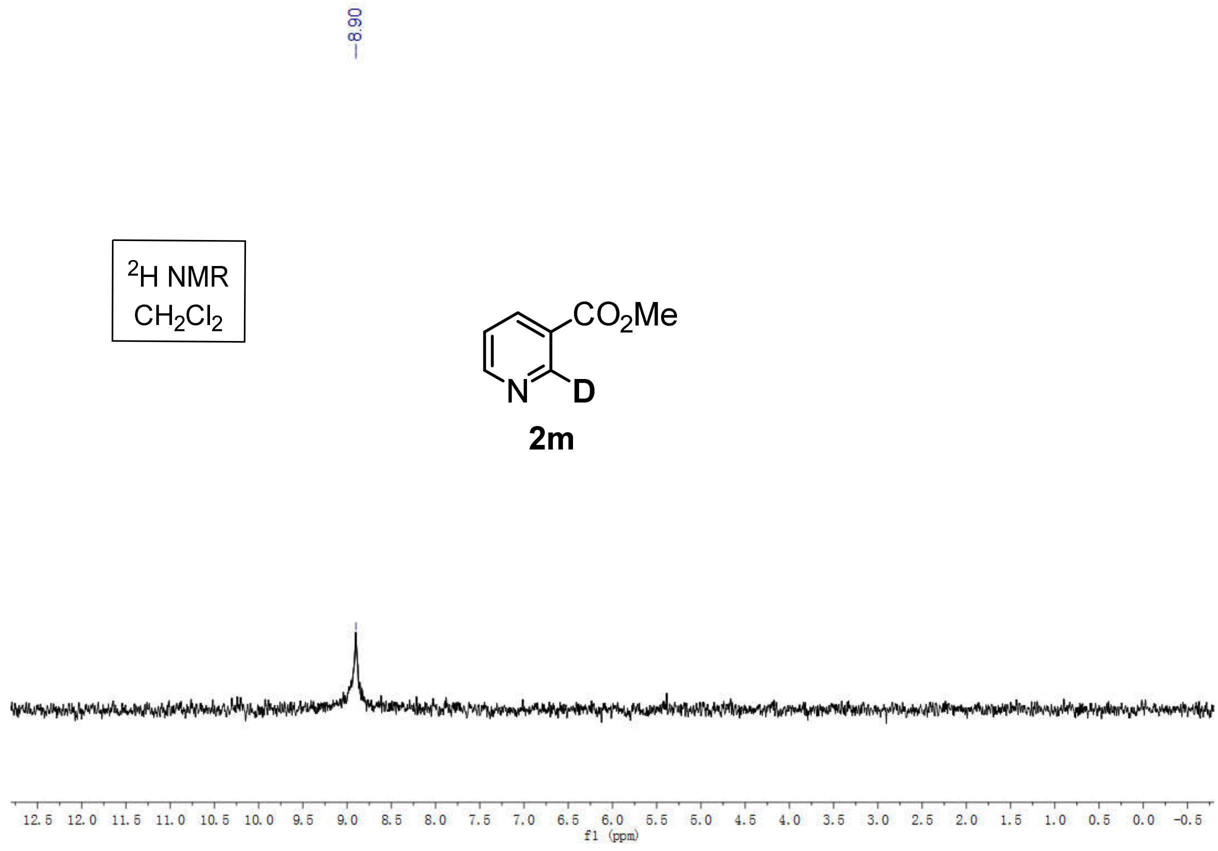

8.90
$^2$H NMR
$CH_2Cl_2$
$CO_2Me$
N
D
2m
f1 (ppm)

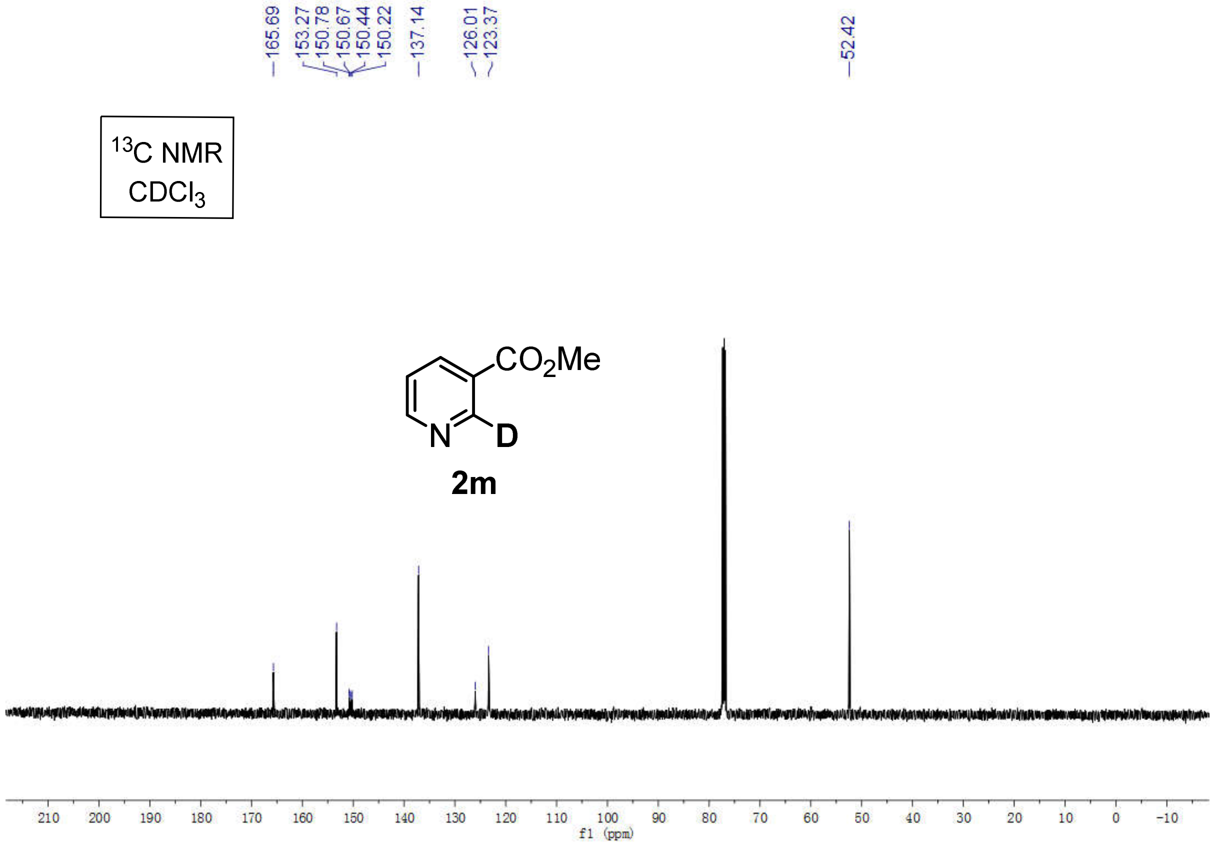


**Supplementary Fig. 55** $^{1}$H NMR, $^{2}$H NMR and $^{13}$C NMR spectra of the compound **2m**.

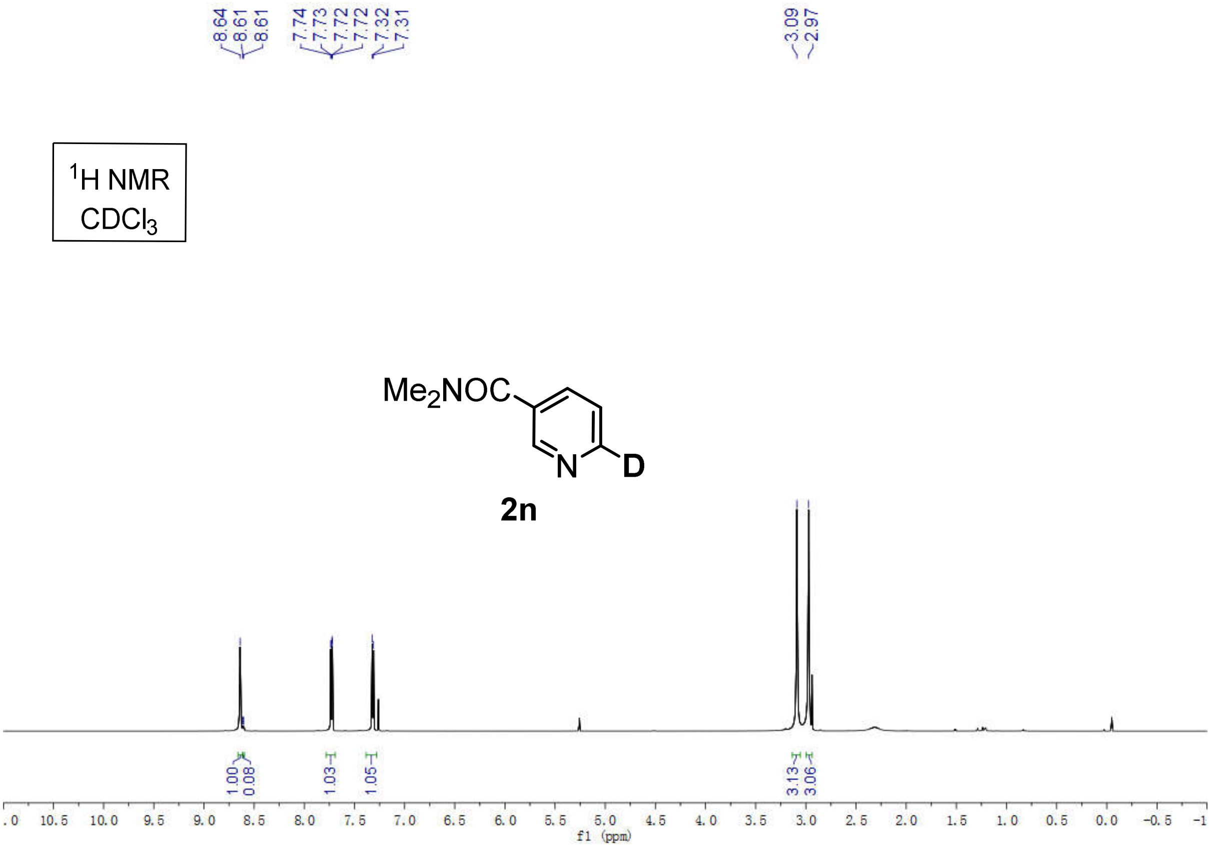
8.64
8.61
8.61
7.74
7.73
7.72
7.72
7.32
7.31
3.09
2.97
1H NMR
CDCl3
Me2NOC
N
D
2n
1.00
0.08
1.03
1.05
3.13
3.06
f1 (ppm)

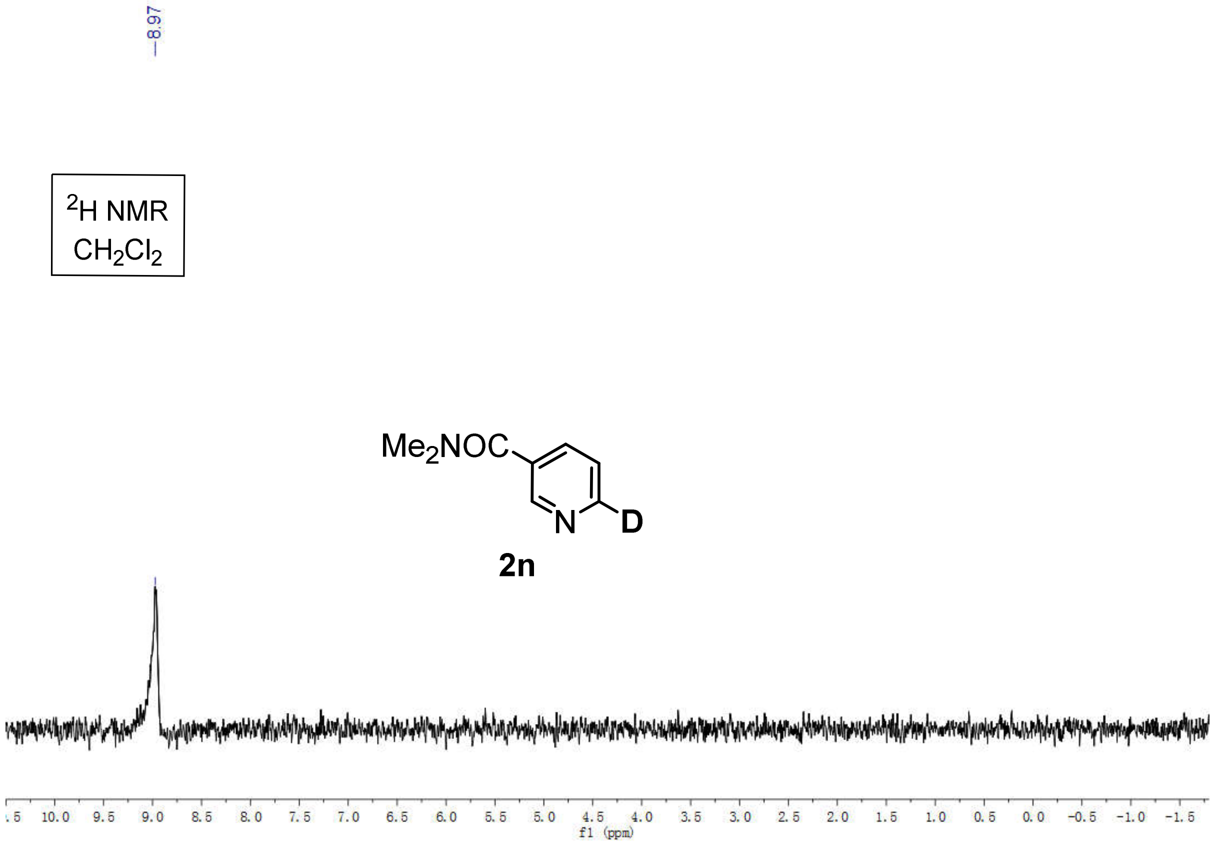
8.97
2H NMR
CH2Cl2
Me2NOC
N
D
2n
f1 (ppm)

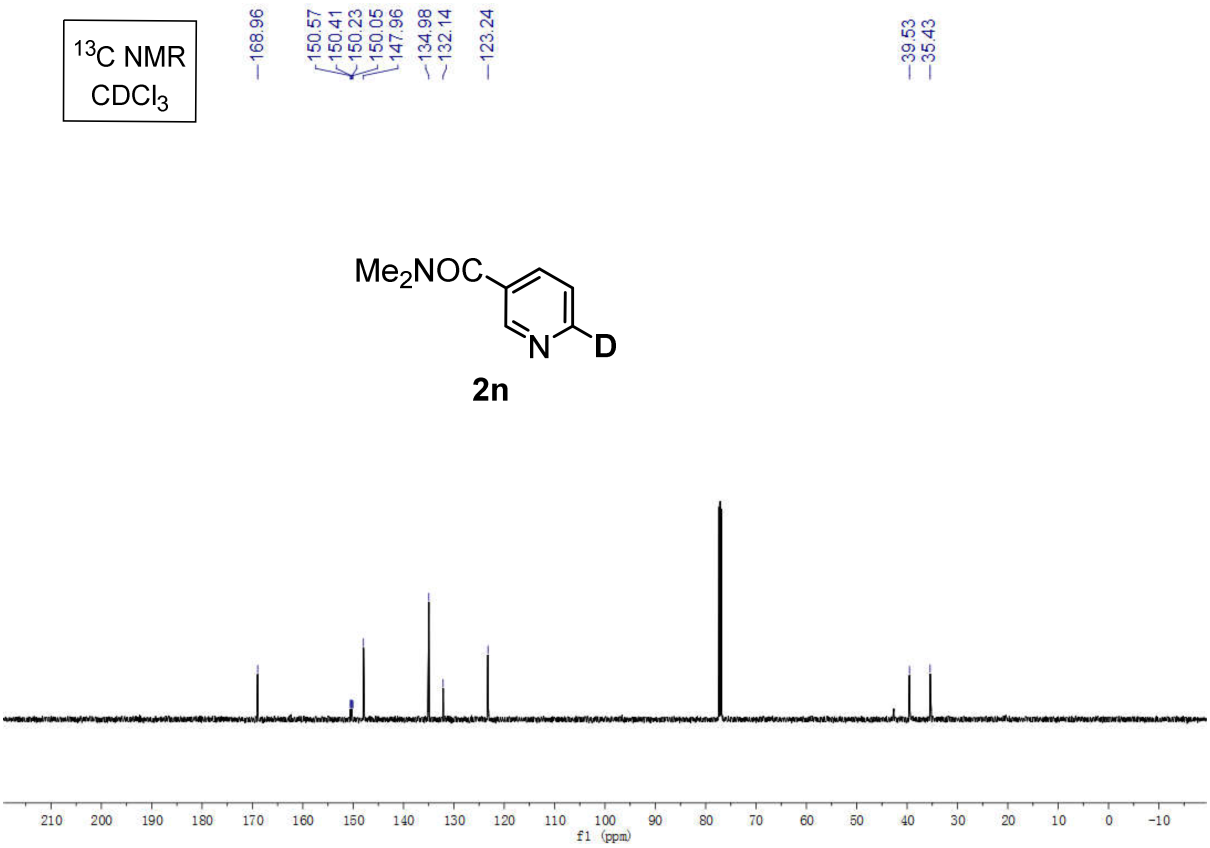


**Supplementary Fig. 56** $^{1}$H NMR, $^{2}$H NMR and $^{13}$C NMR spectra of the compound **2n**.

$^1H$ NMR
$CDCl_3$

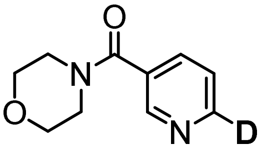

**2o**

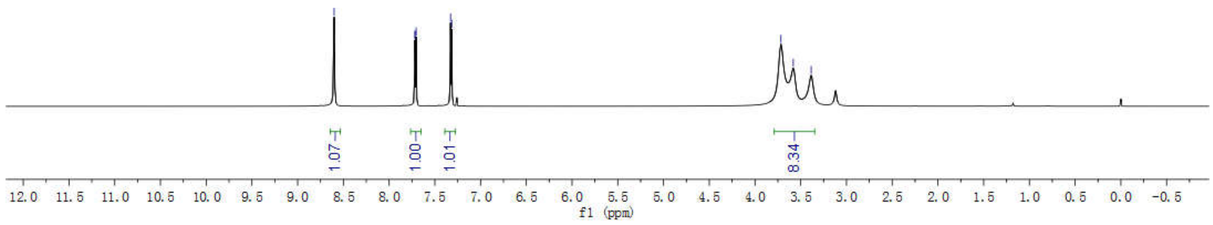


$^2H$ NMR
$CH_2Cl_2$

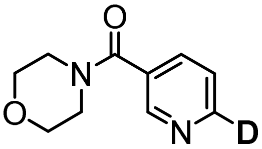

**2o**

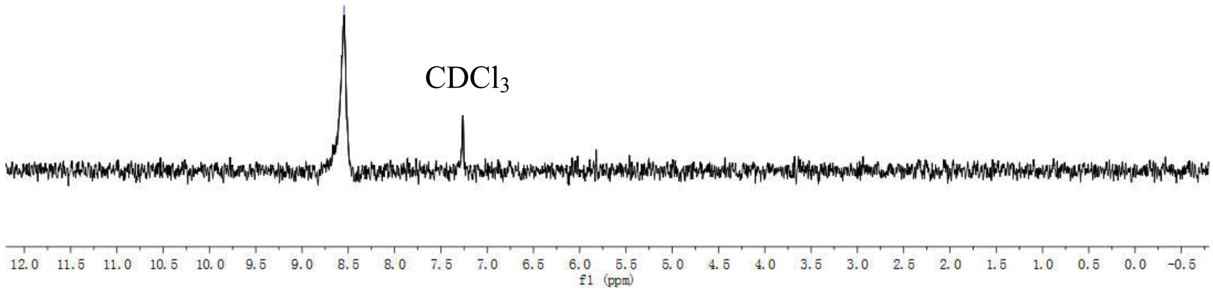

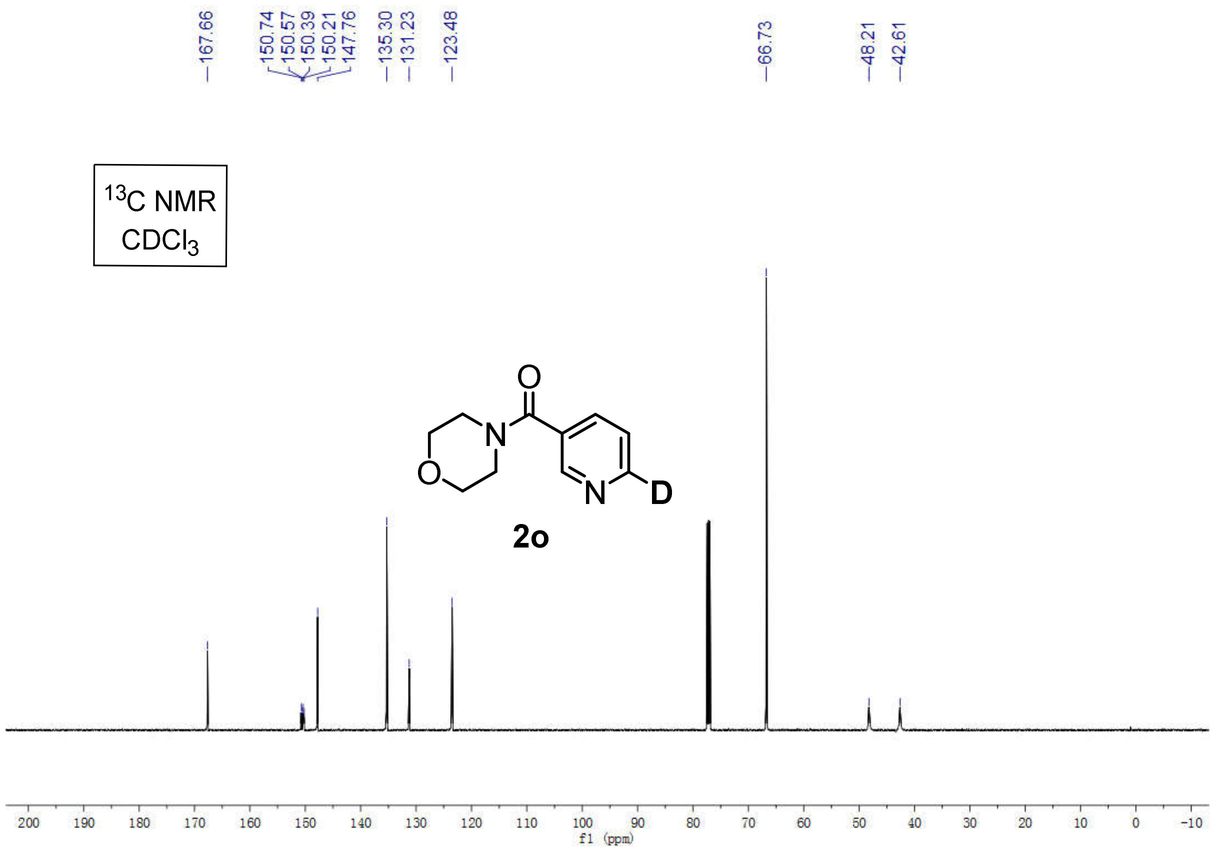


**Supplementary Fig. 57** $^{1}$H NMR, $^{2}$H NMR and $^{13}$C NMR spectra of the compound **2o**.

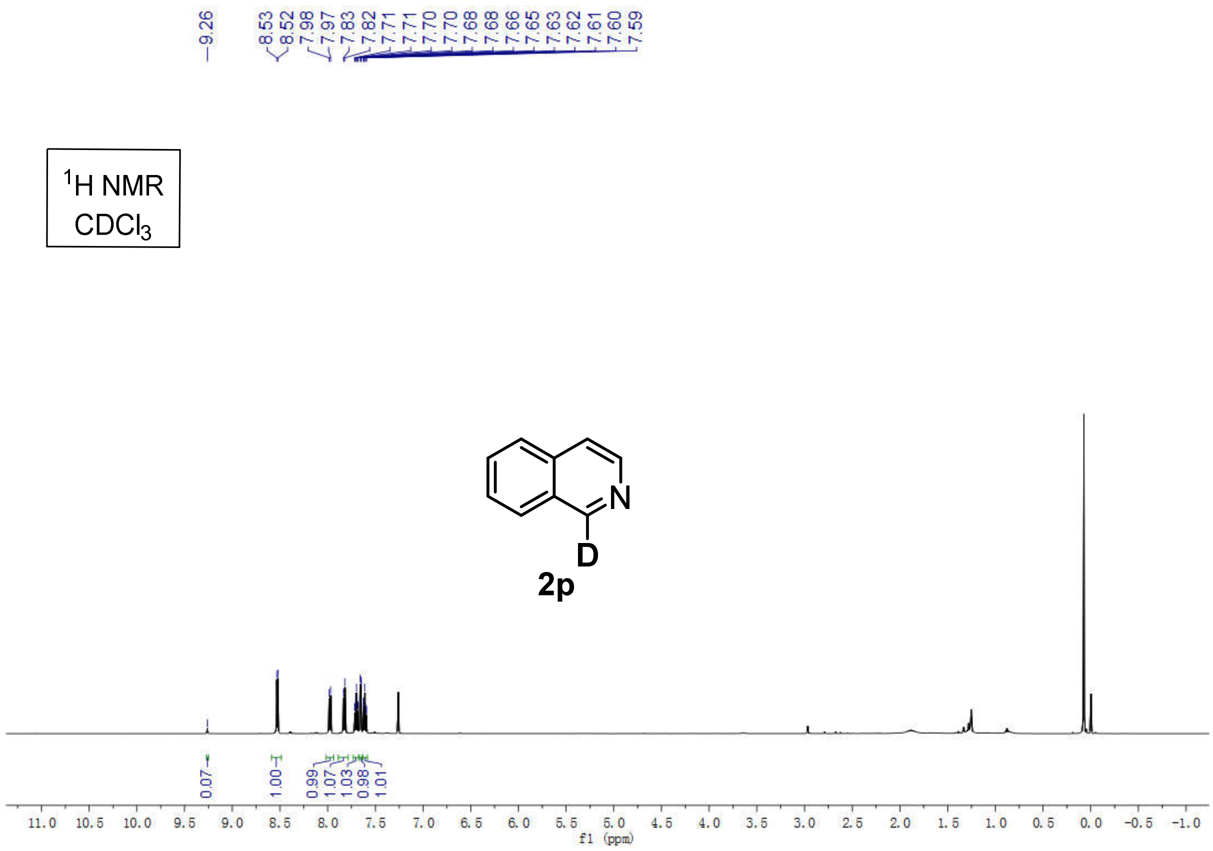

9.26
8.53
8.52
7.98
7.97
7.83
7.82
7.71
7.71
7.70
7.70
7.68
7.68
7.66
7.65
7.63
7.62
7.61
7.60
7.59
$^1$H NMR
$CDCl_3$
N
D
2p
0.07
1.00
0.99
1.07
1.03
0.98
1.01
f1 (ppm)


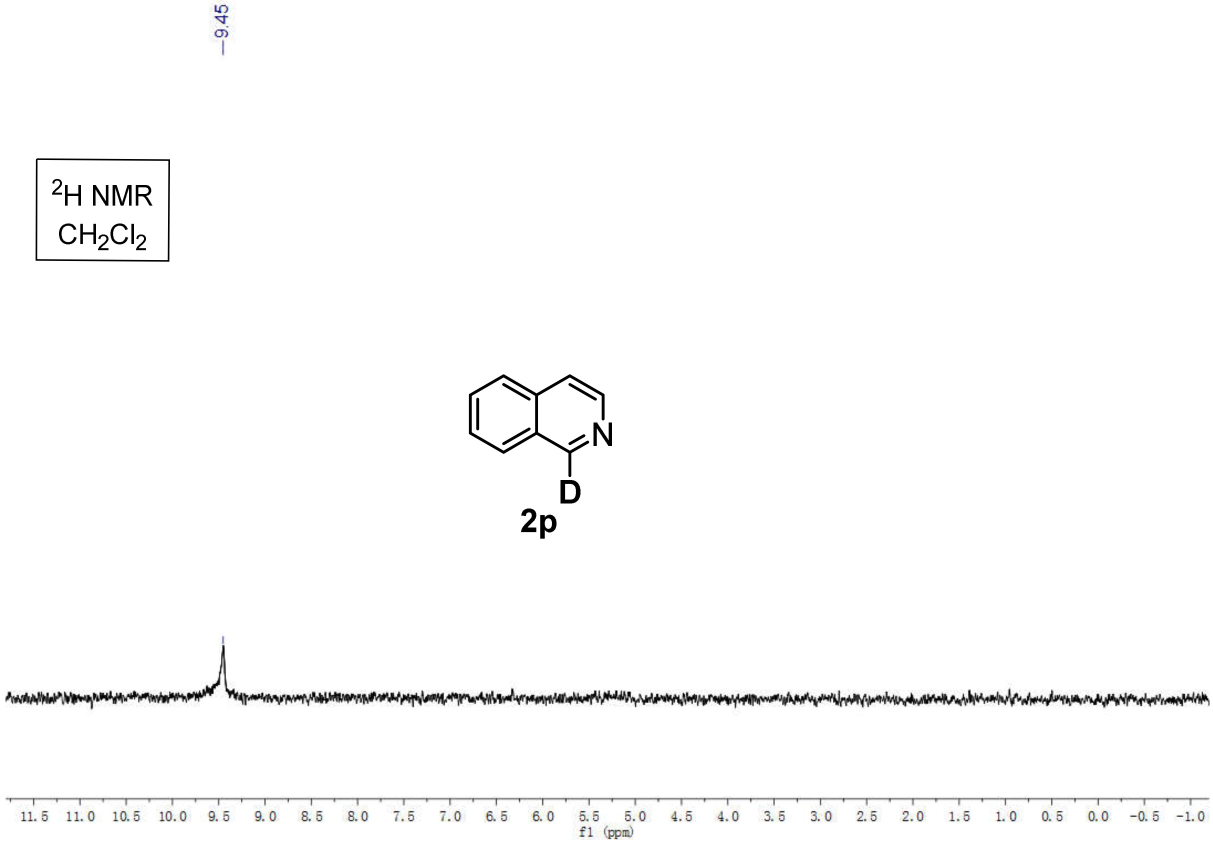

9.45
$^2$H NMR
$CH_2Cl_2$
N
D
2p
f1 (ppm)

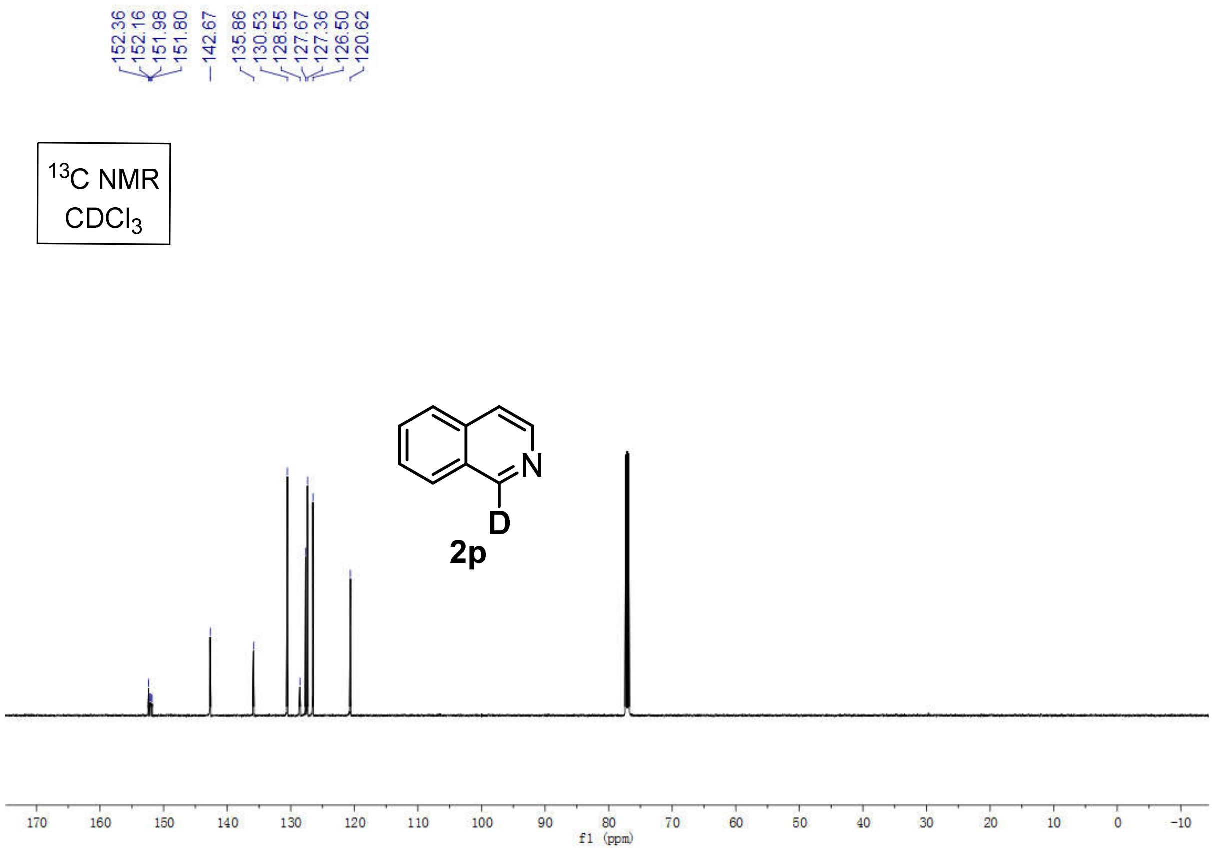


**Supplementary Fig. 58** $^{1}$H NMR, $^{2}$H NMR and $^{13}$C NMR spectra of the compound **2p**.

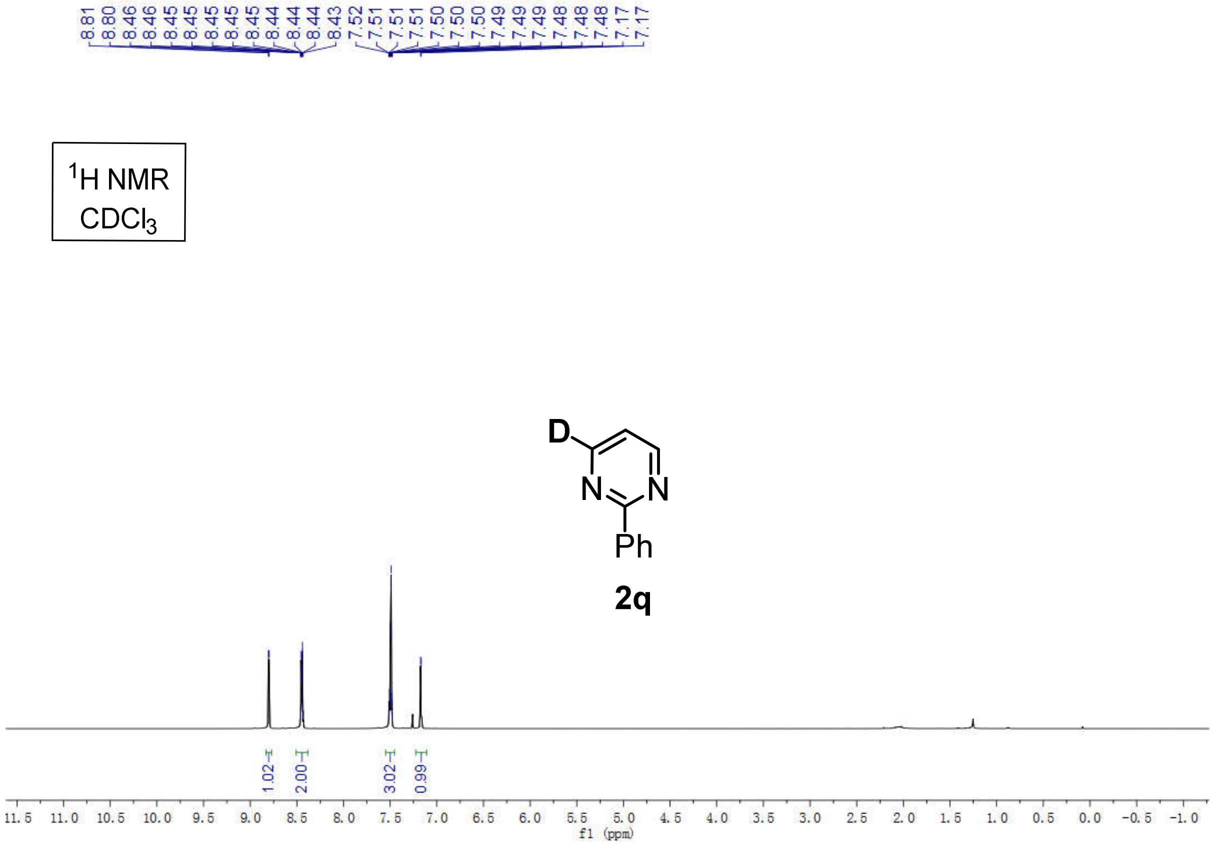

8.81
8.80
8.46
8.46
8.45
8.45
8.45
8.45
8.45
8.44
8.44
8.44
8.43
7.52
7.51
7.51
7.51
7.50
7.50
7.49
7.49
7.48
7.48
7.48
7.17
7.17
$^1$H NMR
$CDCl_3$
D
N
N
Ph
2q
1.02
2.00
3.02
0.99
f1 (ppm)


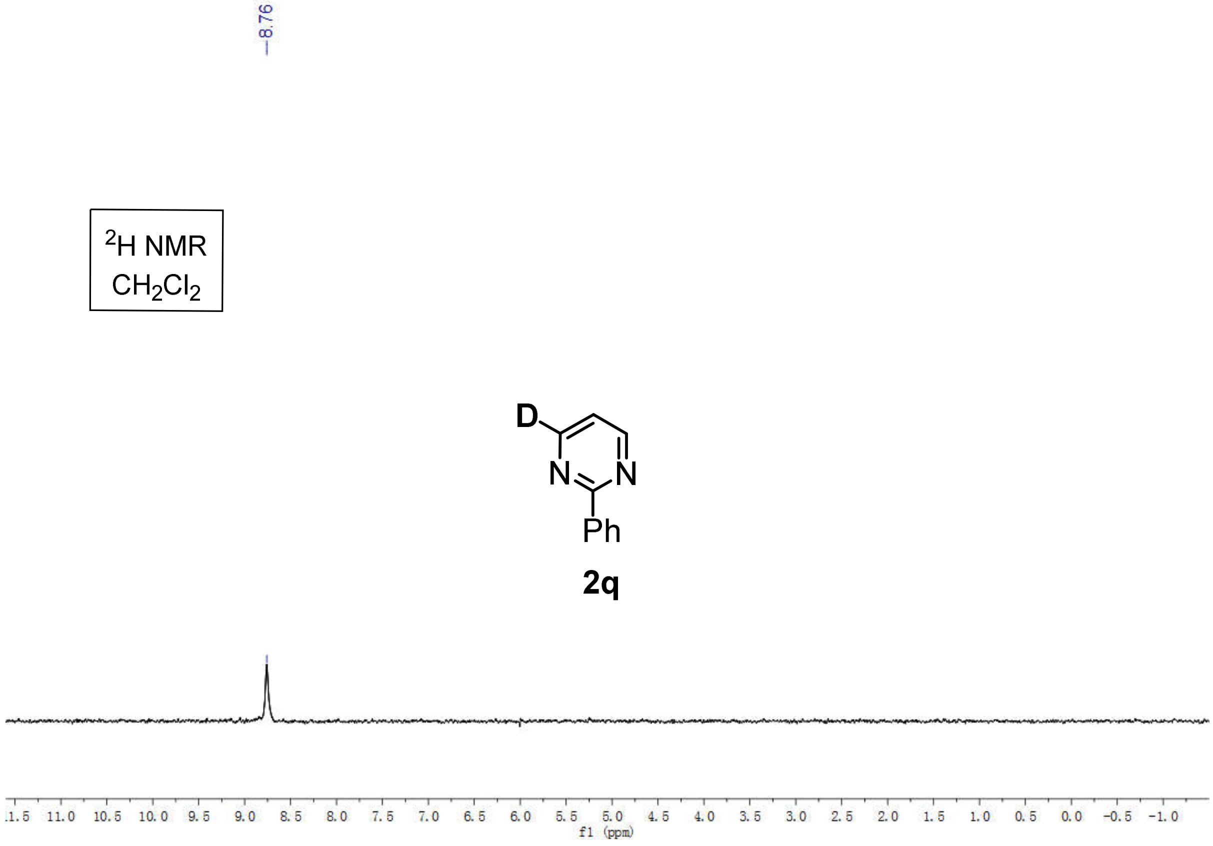

8.76
$^2$H NMR
$CH_2Cl_2$
D
N
N
Ph
2q
f1 (ppm)

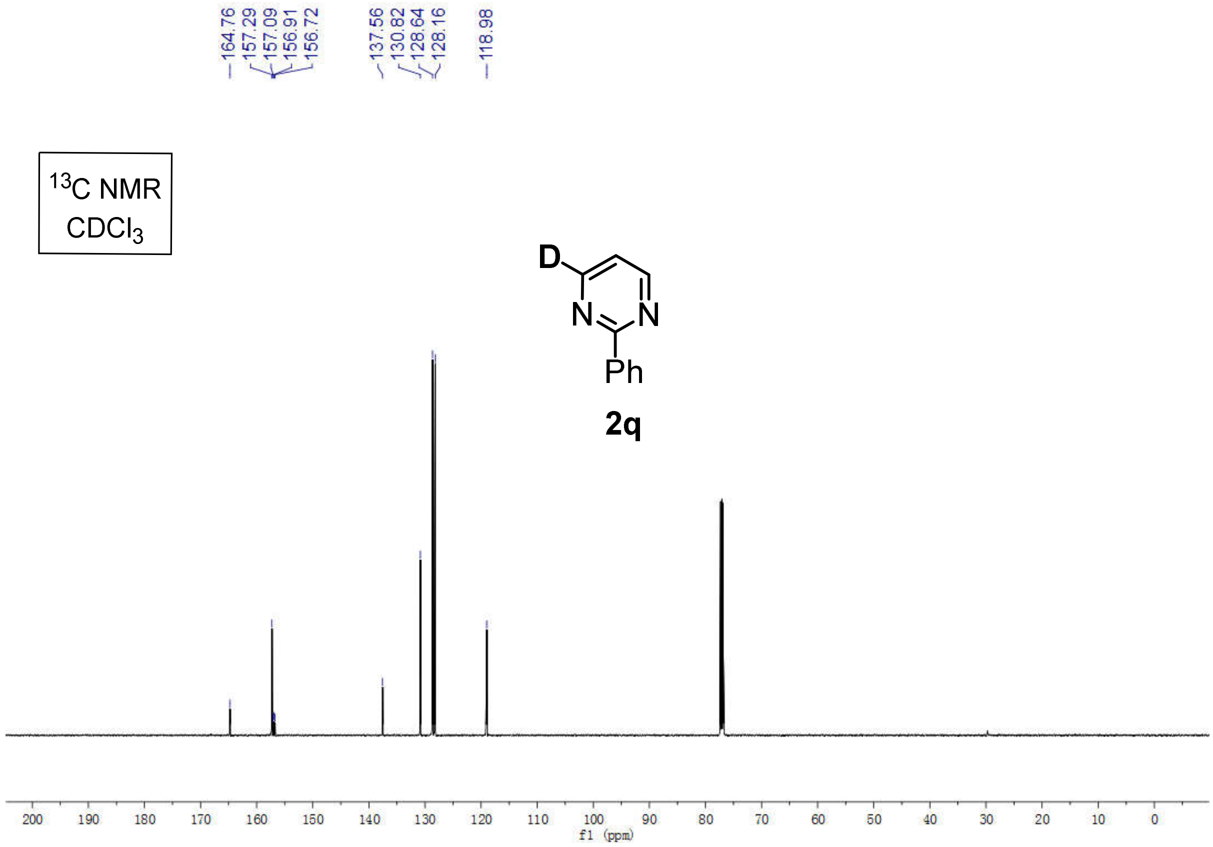


**Supplementary Fig. 59** $^{1}$H NMR, $^{2}$H NMR and $^{13}$C NMR spectra of the compound **2q**.

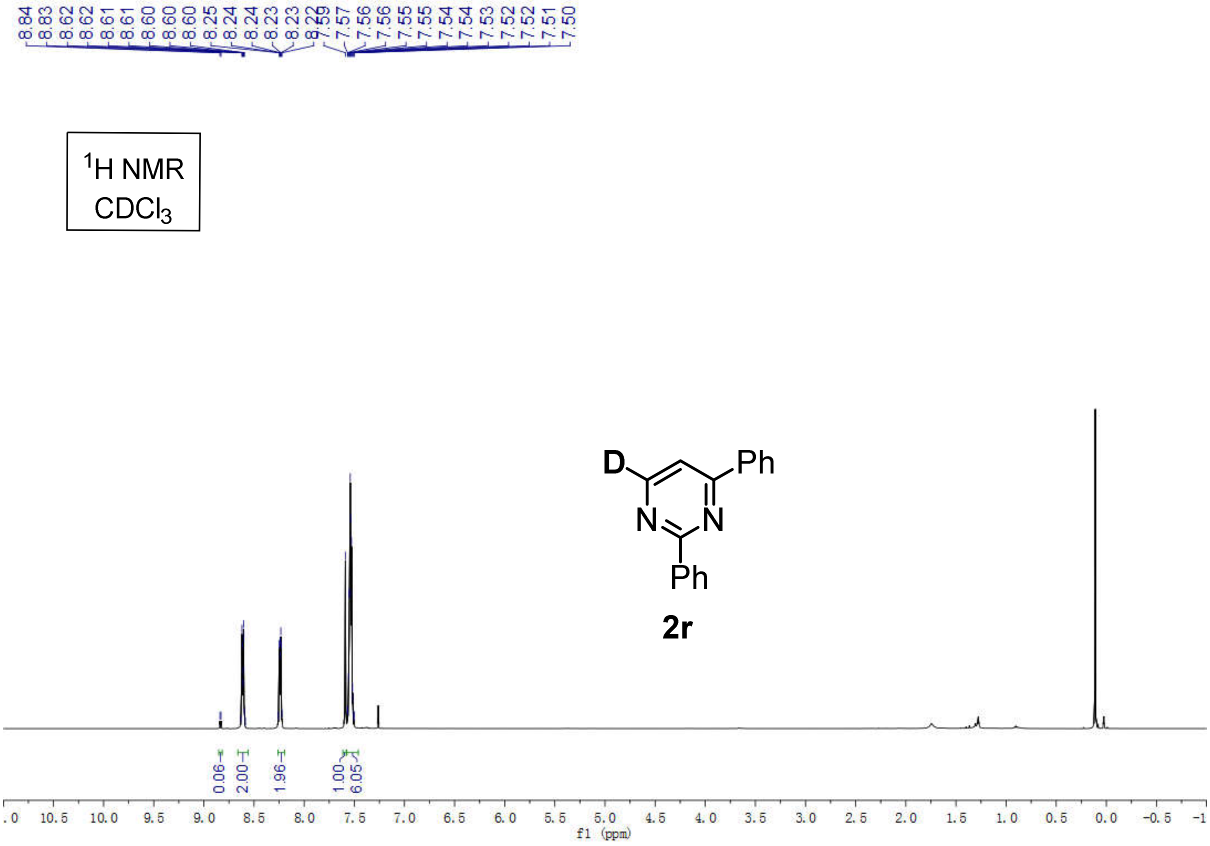
8.84
8.83
8.62
8.62
8.61
8.61
8.60
8.60
8.60
8.25
8.24
8.24
8.23
8.23
8.22
7.59
7.57
7.56
7.56
7.55
7.55
7.54
7.54
7.53
7.52
7.52
7.51
7.50
$^{1}$H NMR
$CDCl_3$
D
Ph
N
N
Ph
2r
0.06
2.00
1.96
1.00
6.05
f1 (ppm)

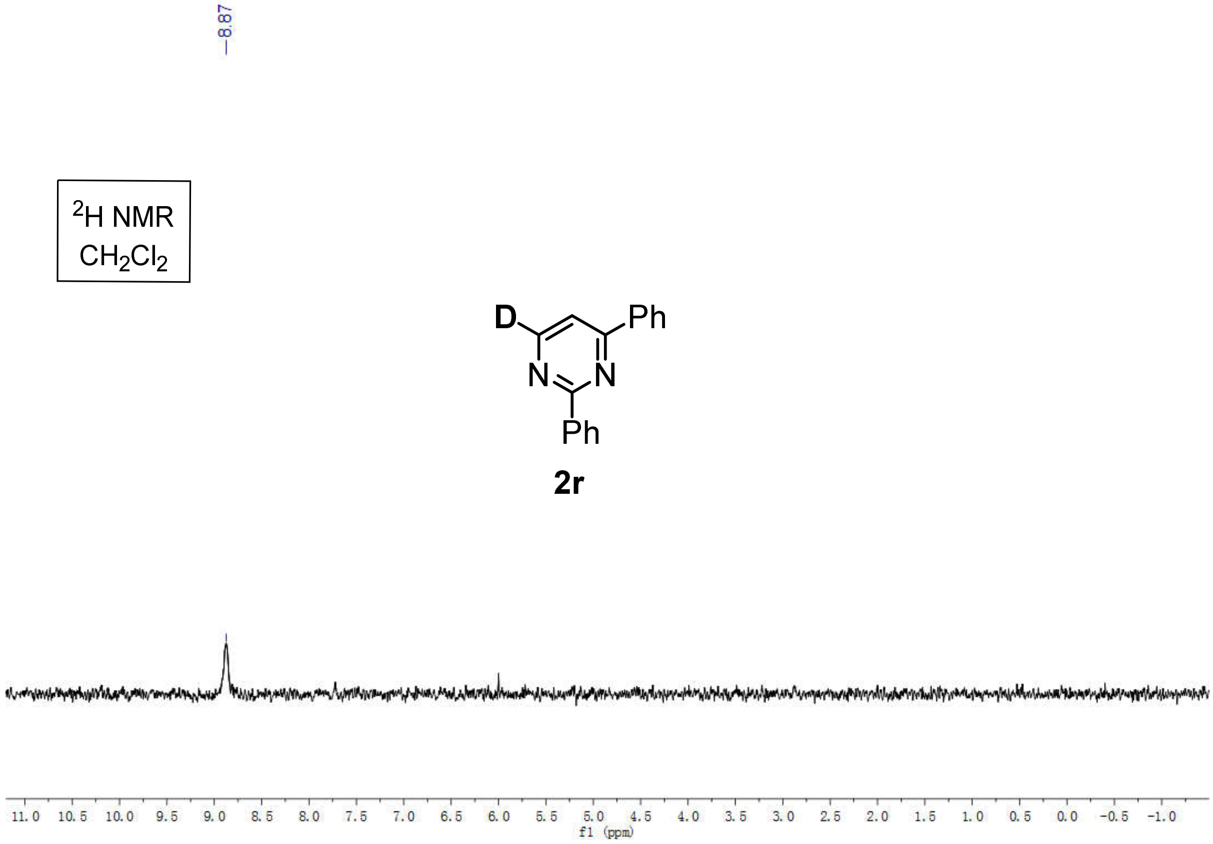
8.87
$^{2}$H NMR
$CH_2Cl_2$
D
Ph
N
N
Ph
2r
f1 (ppm)

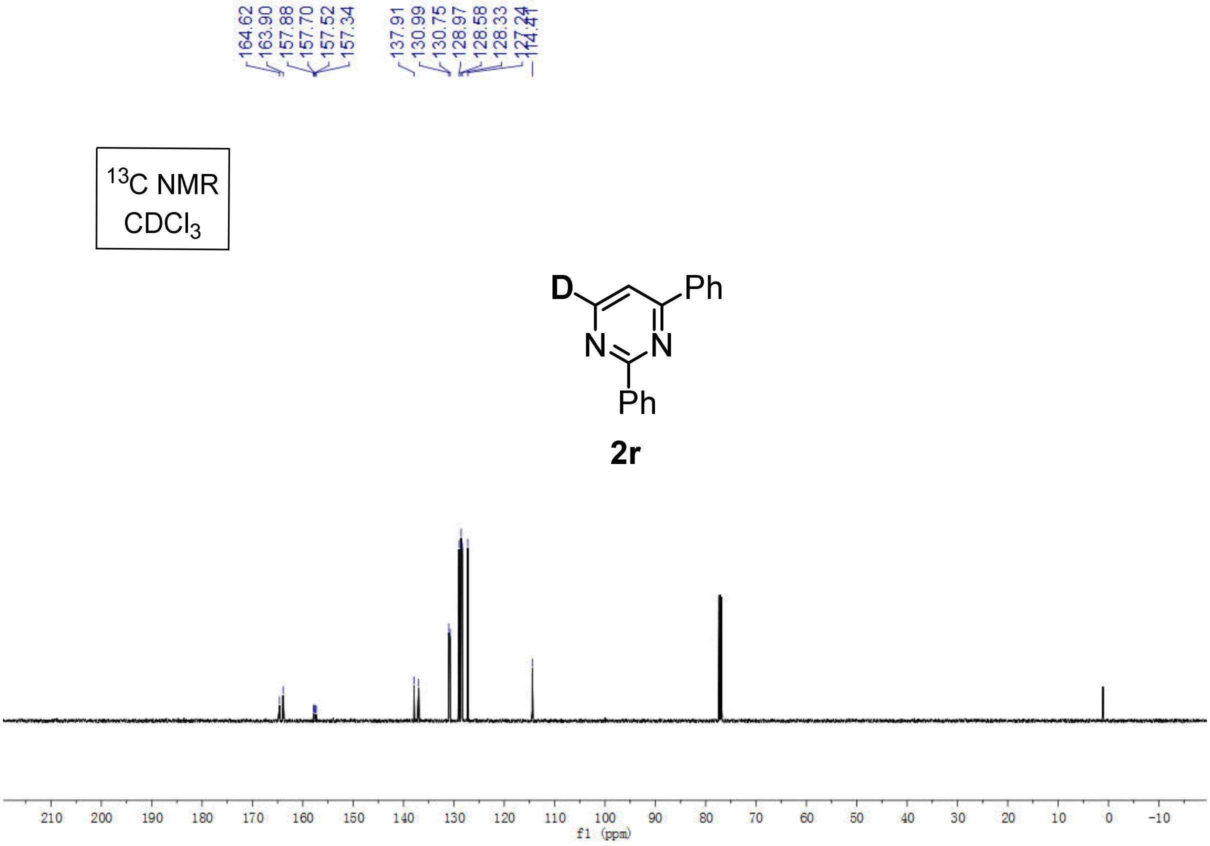


**Supplementary Fig. 60** $^1$H NMR, $^2$H NMR and $^{13}$C NMR spectra of the compound **2r**.

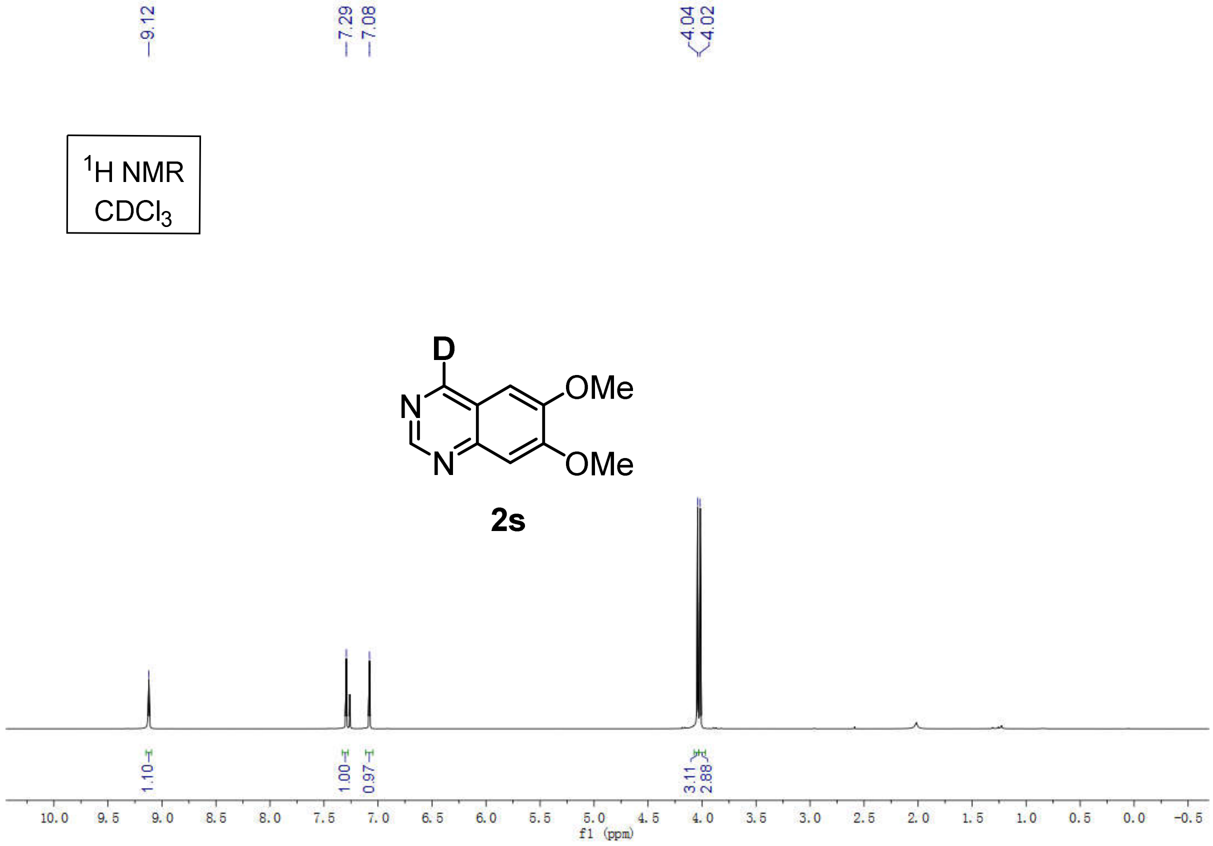
9.12
7.29
7.08
4.04
4.02
1H NMR
CDCl3
D
N
N
OMe
OMe
2s
1.10
1.00
0.97
3.11
2.88
10.0 9.5 9.0 8.5 8.0 7.5 7.0 6.5 6.0 5.5 5.0 4.5 4.0 3.5 3.0 2.5 2.0 1.5 1.0 0.5 0.0 -0.5
f1 (ppm)

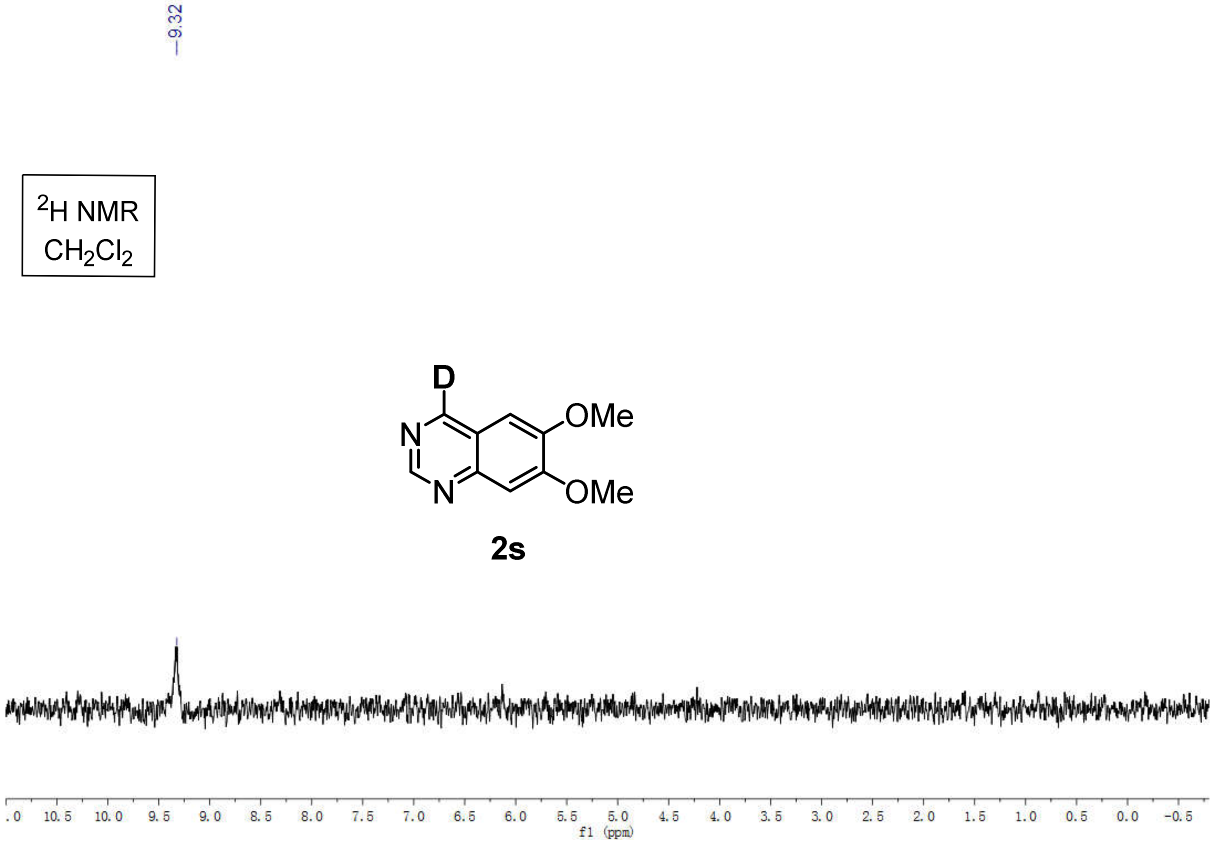
9.32
2H NMR
CH2Cl2
D
N
N
OMe
OMe
2s
.0 10.5 10.0 9.5 9.0 8.5 8.0 7.5 7.0 6.5 6.0 5.5 5.0 4.5 4.0 3.5 3.0 2.5 2.0 1.5 1.0 0.5 0.0 -0.5
f1 (ppm)

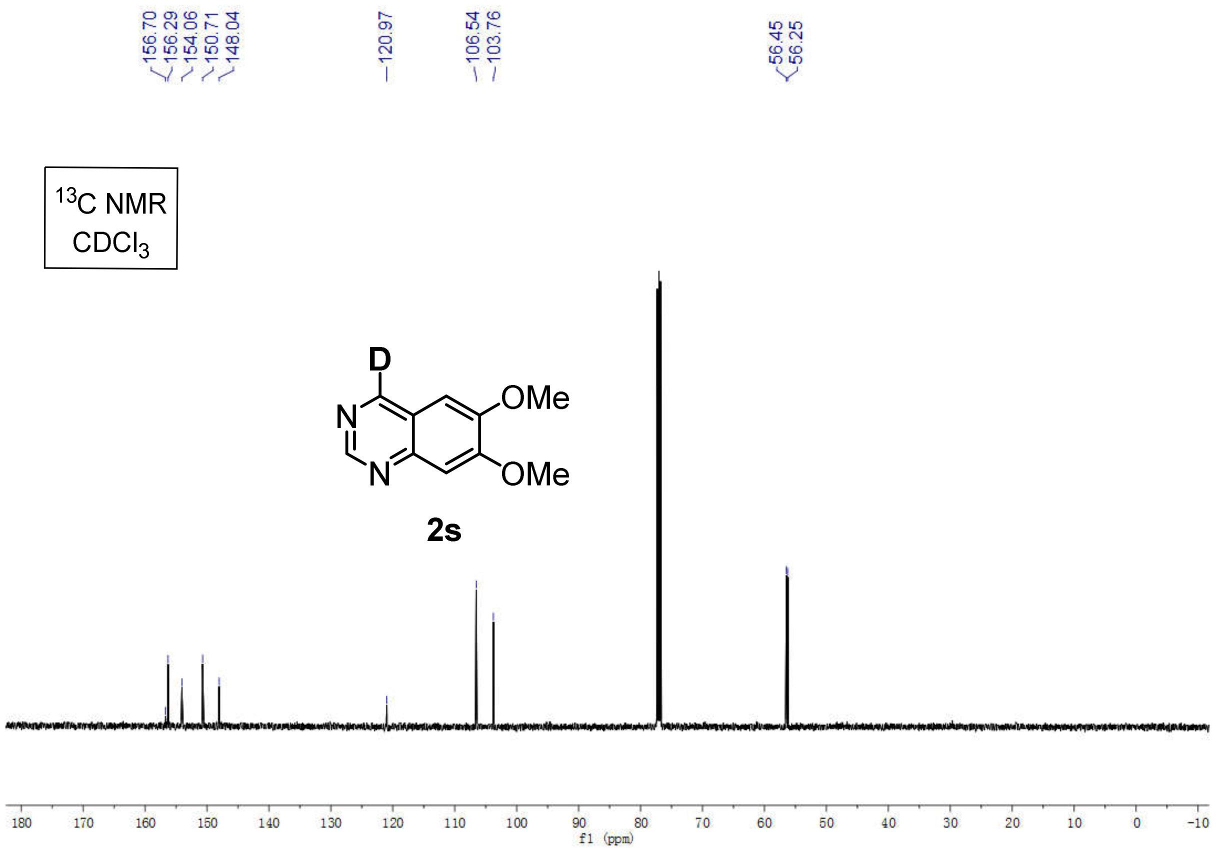


**Supplementary Fig. 61** $^{1}$H NMR, $^{2}$H NMR and $^{13}$C NMR spectra of the compound **2s**.

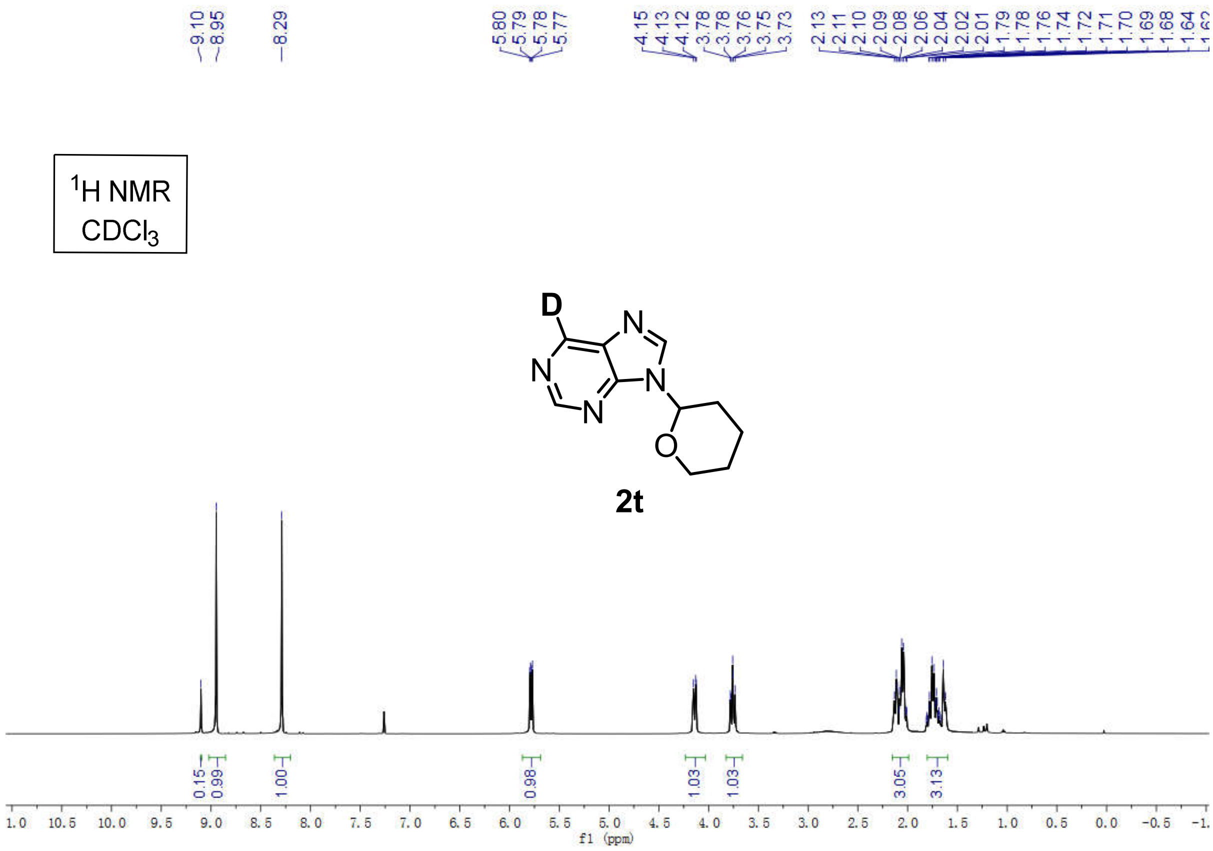

$^1$H NMR
$CDCl_3$
2t
f1 (ppm)


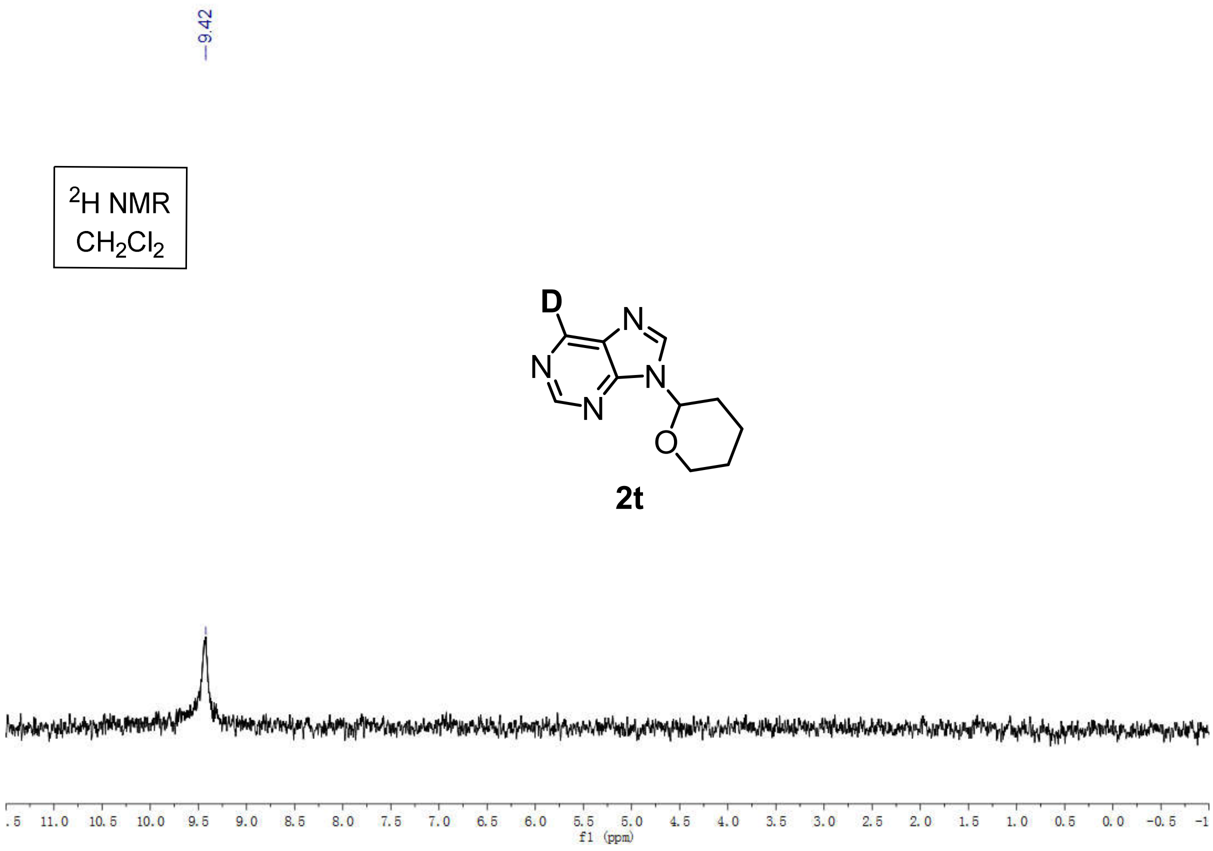

$^2$H NMR
$CH_2Cl_2$
2t
f1 (ppm)

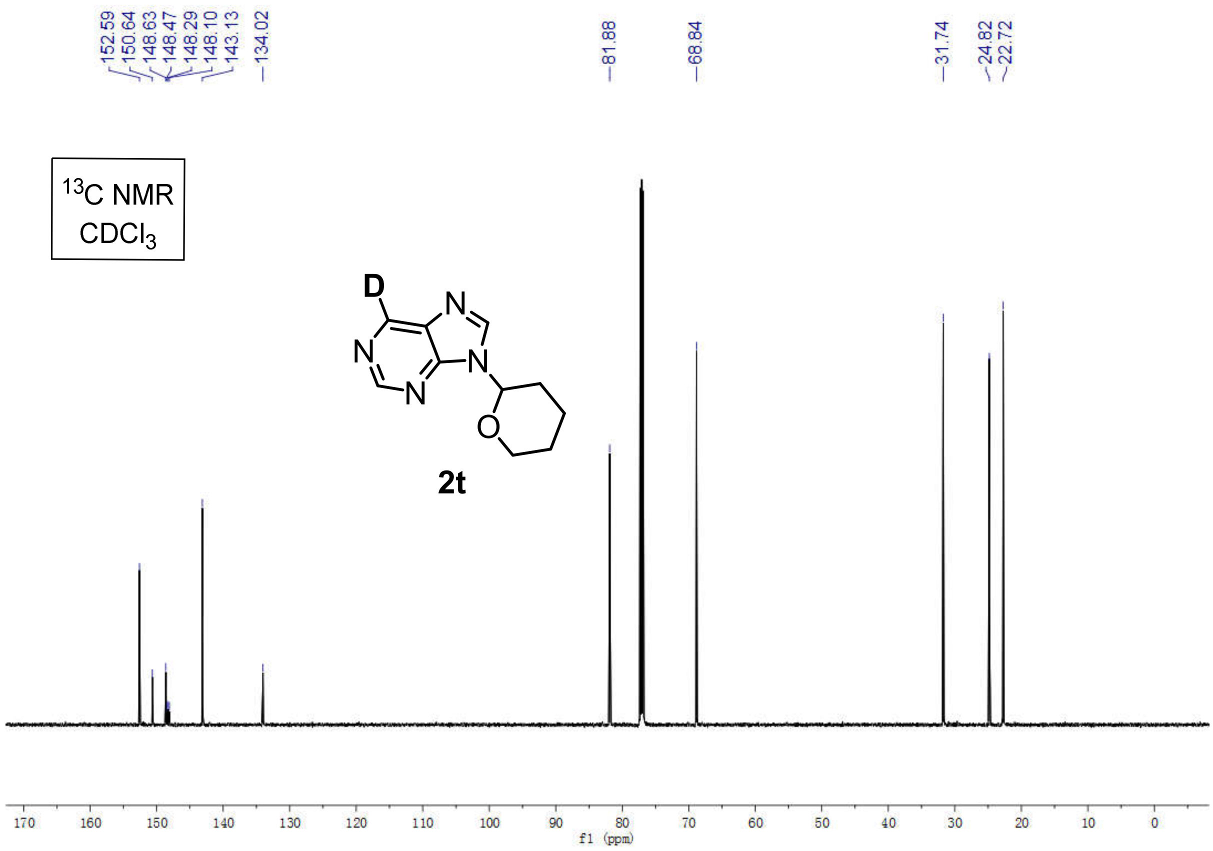


**Supplementary Fig. 62** $^1$H NMR, $^2$H NMR and $^{13}$C NMR spectra of the compound **2t**.

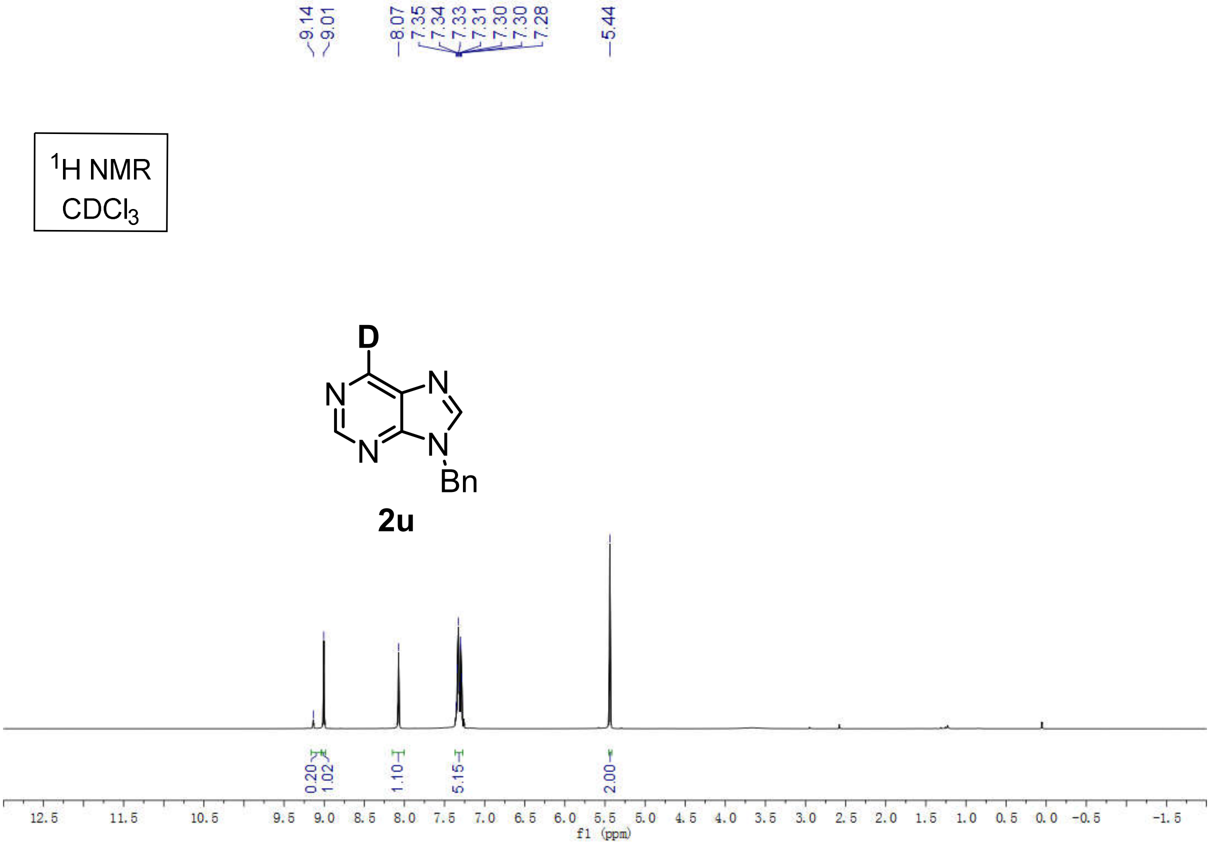
9.14
9.01
8.07
7.35
7.34
7.33
7.31
7.30
7.30
7.28
5.44
1H NMR
CDCl3
D
N
N
N
N
Bn
2u
0.20
1.02
1.10
5.15
2.00
f1 (ppm)

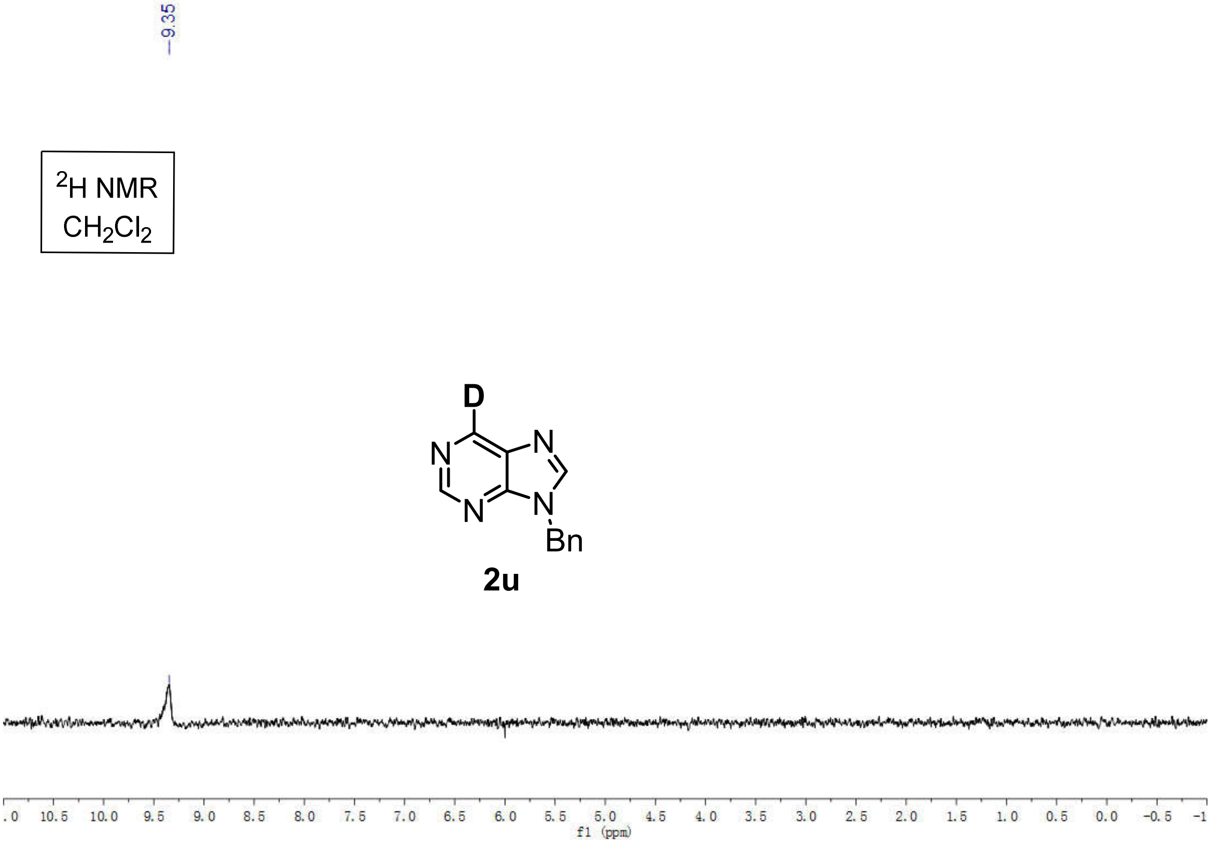
9.35
2H NMR
CH2Cl2
D
N
N
N
N
Bn
2u
f1 (ppm)

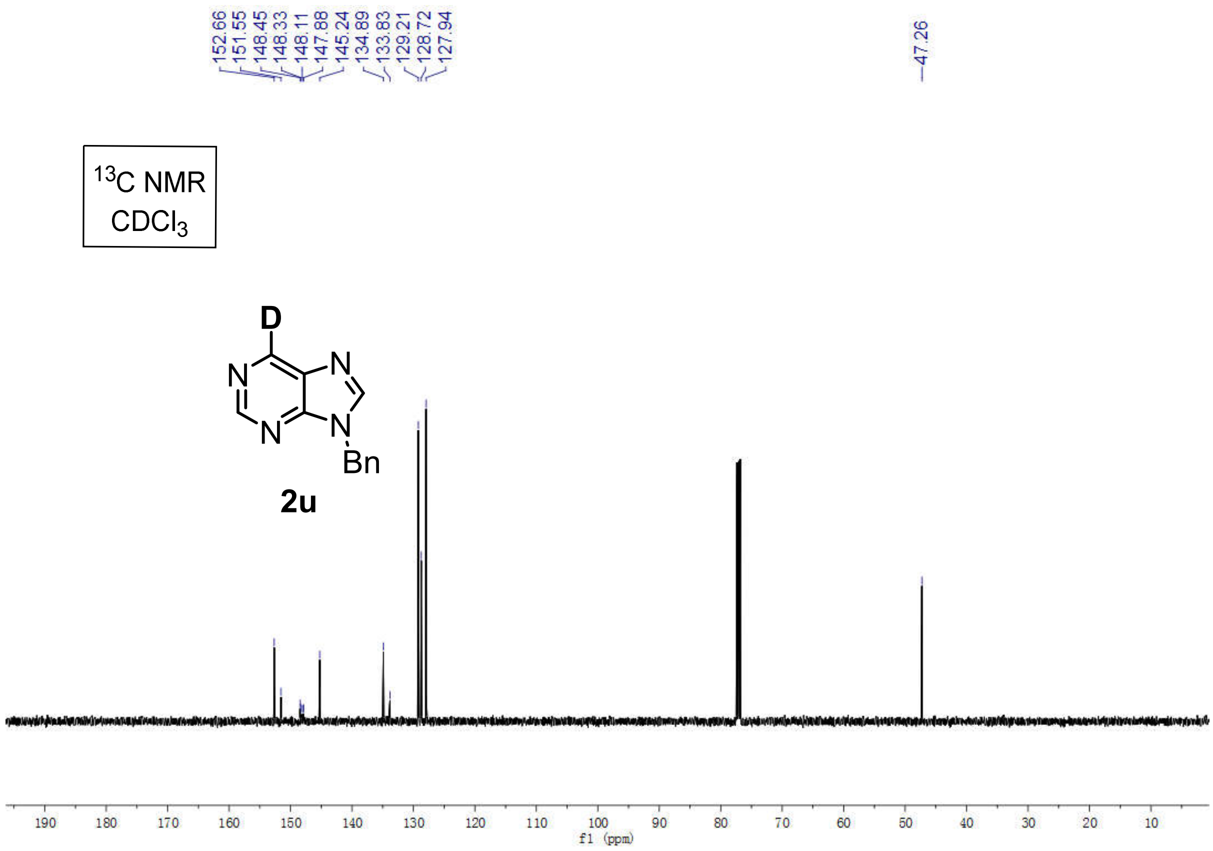


**Supplementary Fig. 63** $^{1}$H NMR, $^{2}$H NMR and $^{13}$C NMR spectra of the compound **2u**.

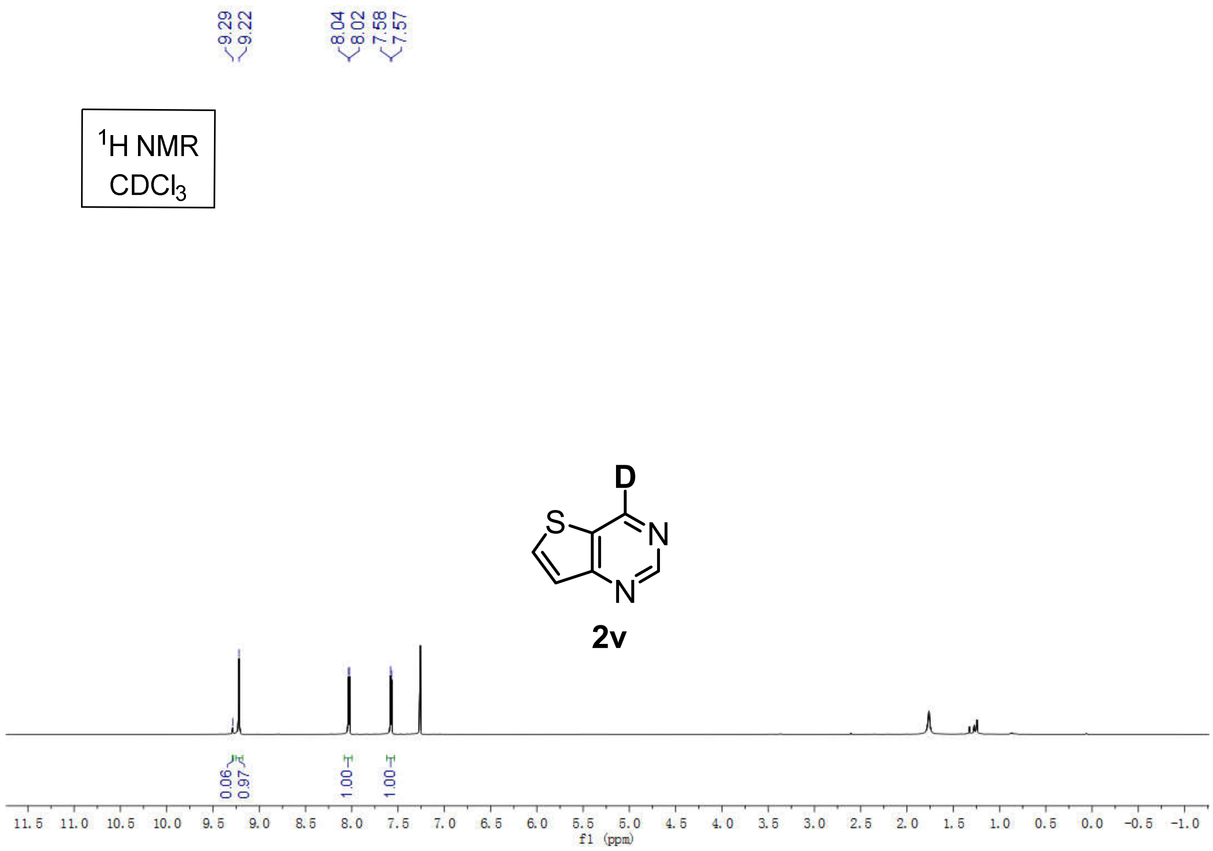
9.29
9.22
8.04
8.02
7.58
7.57
$^1$H NMR
$CDCl_3$
D
S
N
N
2v
0.06
0.97
1.00
1.00
11.5 11.0 10.5 10.0 9.5 9.0 8.5 8.0 7.5 7.0 6.5 6.0 5.5 5.0 4.5 4.0 3.5 3.0 2.5 2.0 1.5 1.0 0.5 0.0 -0.5 -1.0
f1 (ppm)

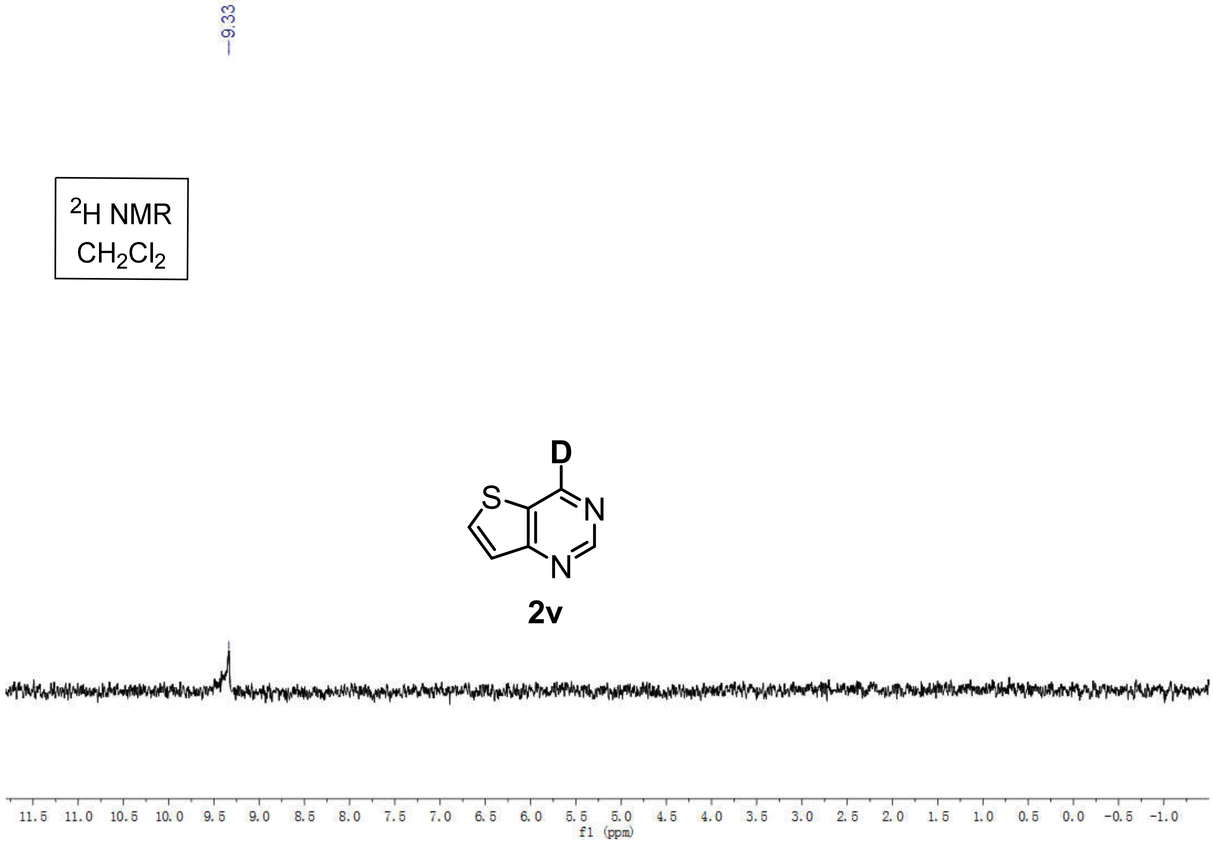
9.33
$^2$H NMR
$CH_2Cl_2$
D
S
N
N
2v
11.5 11.0 10.5 10.0 9.5 9.0 8.5 8.0 7.5 7.0 6.5 6.0 5.5 5.0 4.5 4.0 3.5 3.0 2.5 2.0 1.5 1.0 0.5 0.0 -0.5 -1.0
f1 (ppm)

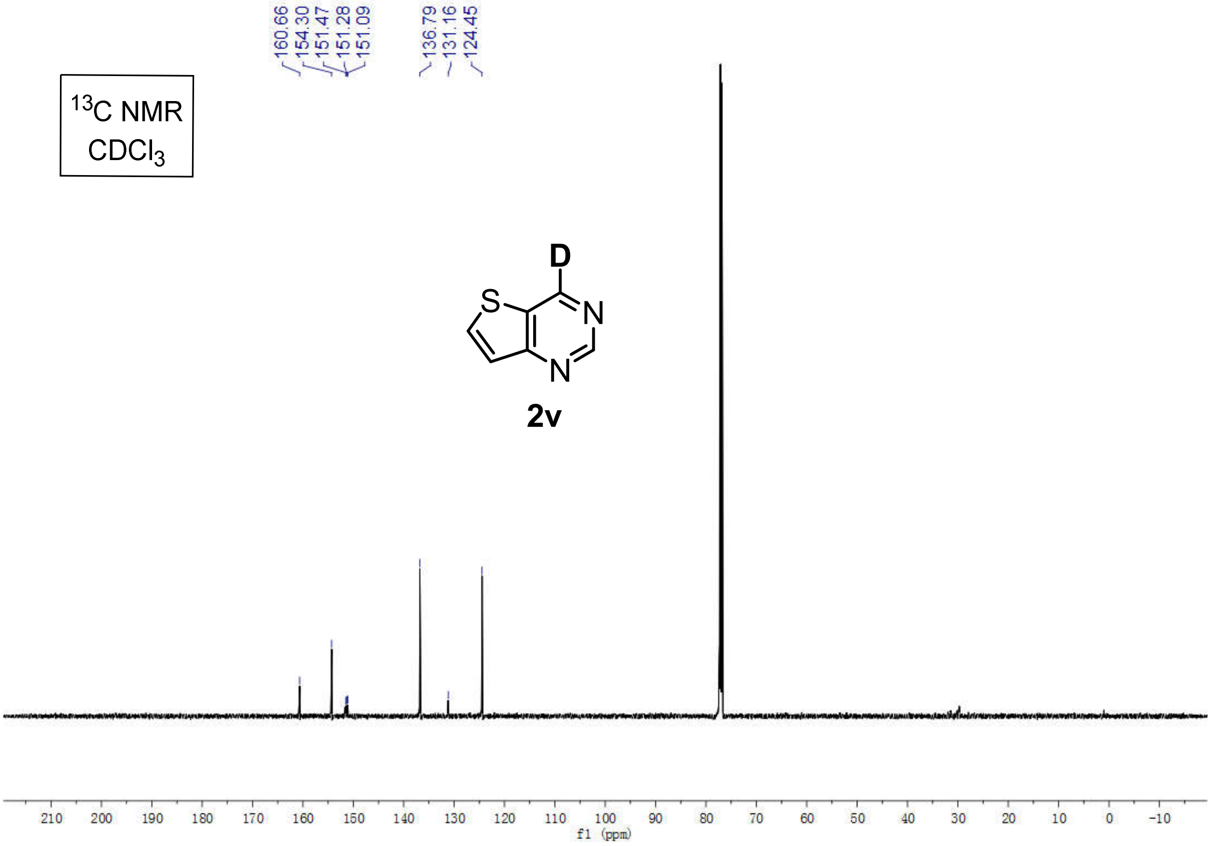


**Supplementary Fig. 64** $^{1}H$ NMR, $^{2}H$ NMR and $^{13}C$ NMR spectra of the compound **2v**.

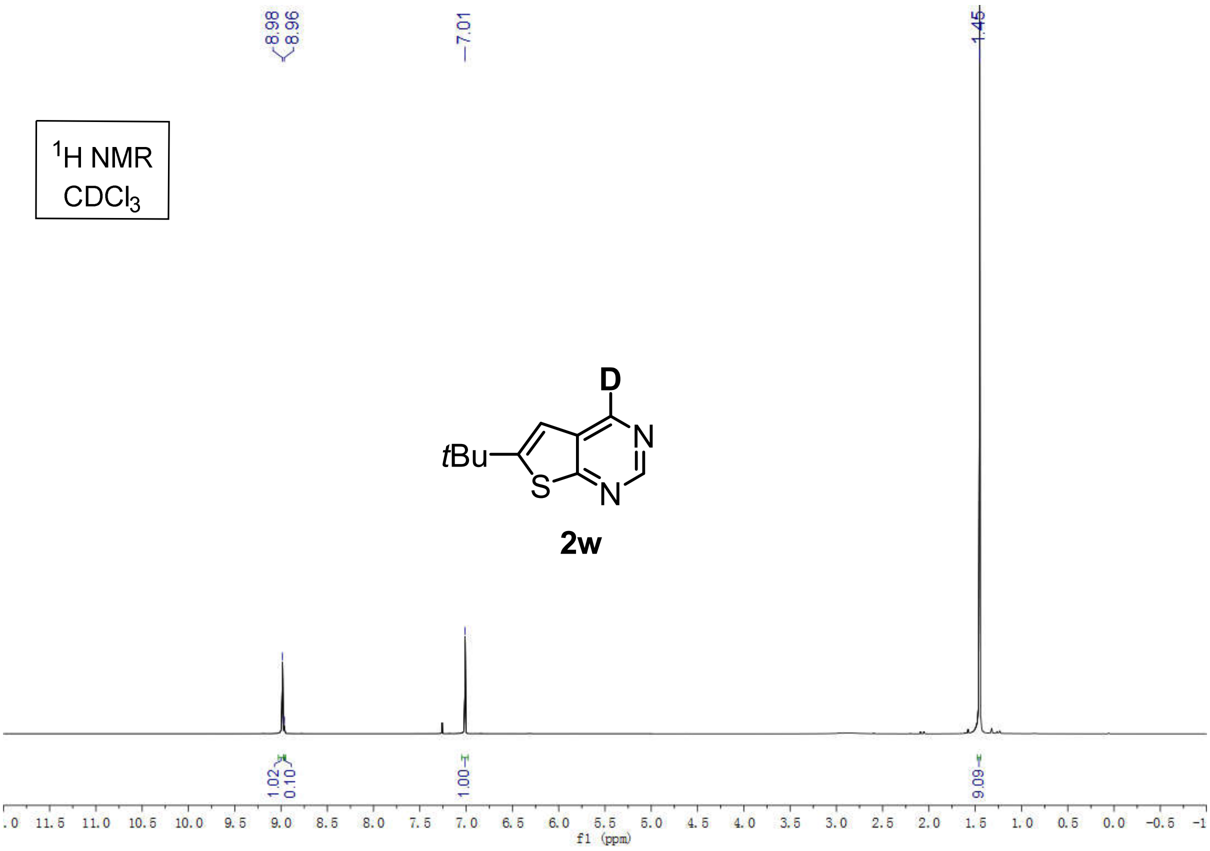

8.98
8.96
7.01
1.45
1H NMR
CDCl3
D
tBu
N
S
N
2w
1.02
0.10
1.00
9.09
f1 (ppm)


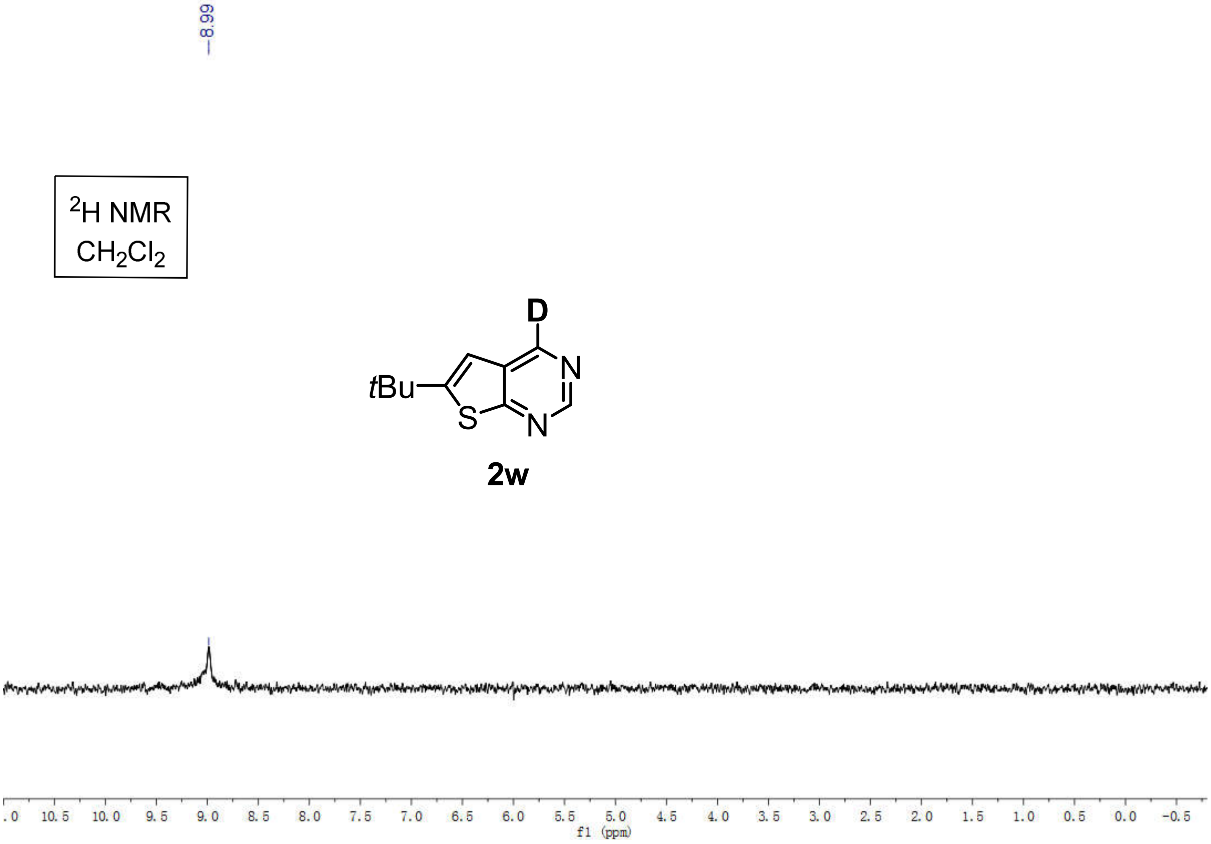

8.99
2H NMR
CH2Cl2
D
tBu
N
S
N
2w
f1 (ppm)

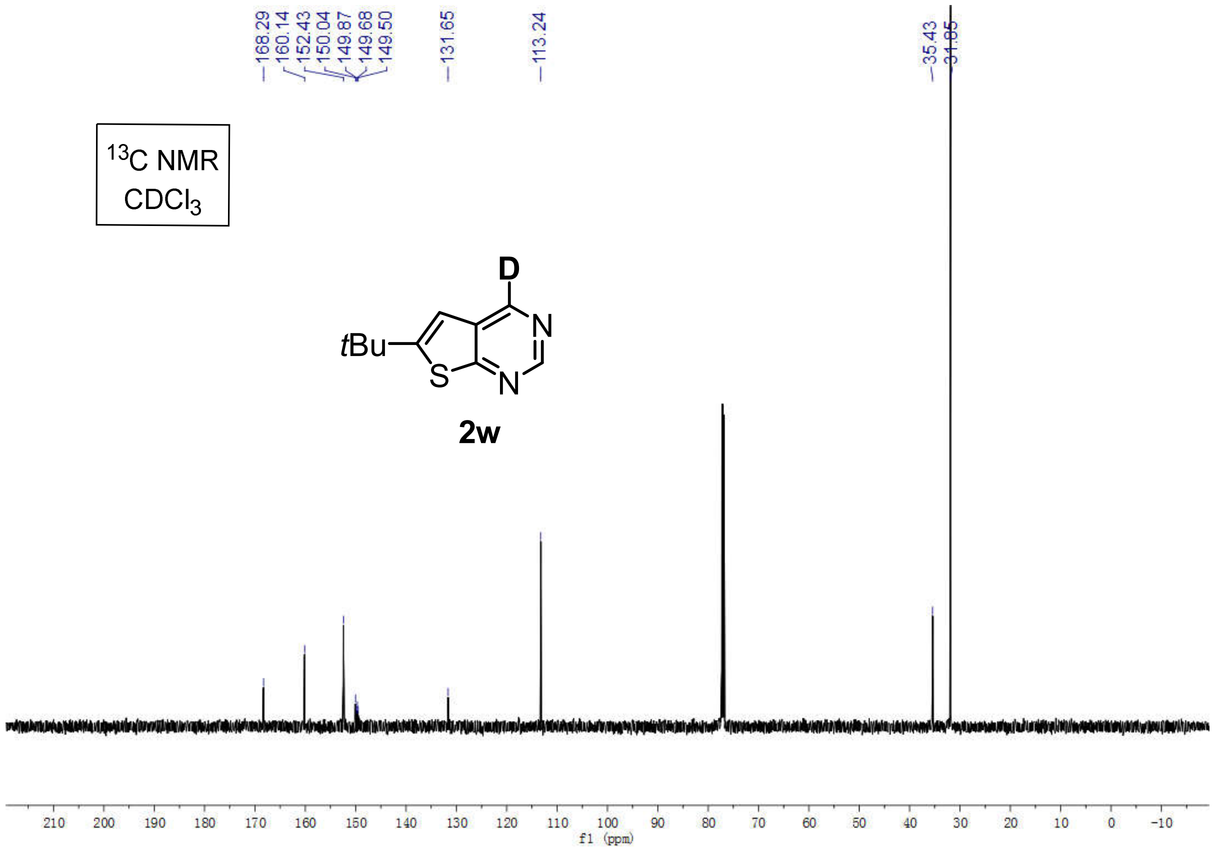


**Supplementary Fig. 65** $^{1}H$ NMR, $^{2}H$ NMR and $^{13}C$ NMR spectra of the compound **2w**.

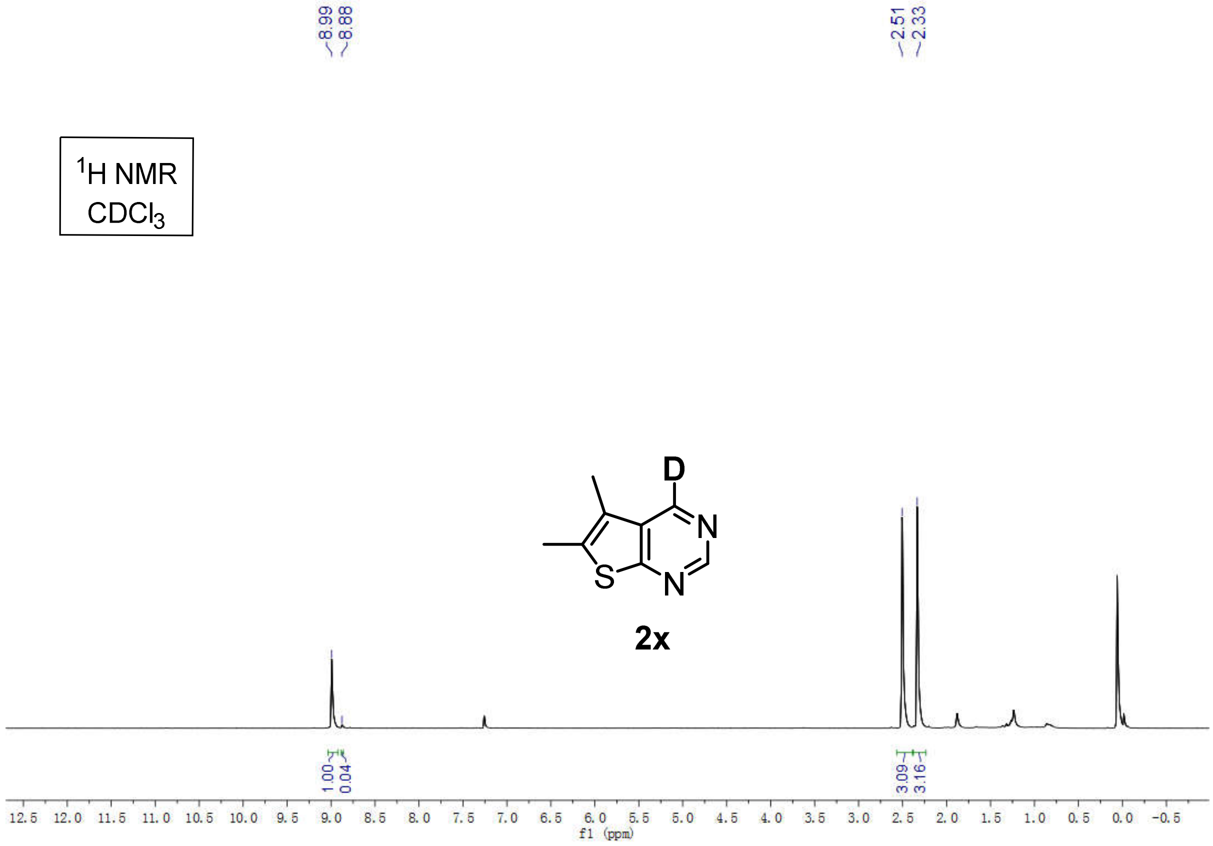

8.99
8.88
2.51
2.33
$^1$H NMR
$CDCl_3$
D
N
S
N
2x
1.00
0.04
3.09
3.16
12.5 12.0 11.5 11.0 10.5 10.0 9.5 9.0 8.5 8.0 7.5 7.0 6.5 6.0 5.5 5.0 4.5 4.0 3.5 3.0 2.5 2.0 1.5 1.0 0.5 0.0 -0.5
f1 (ppm)


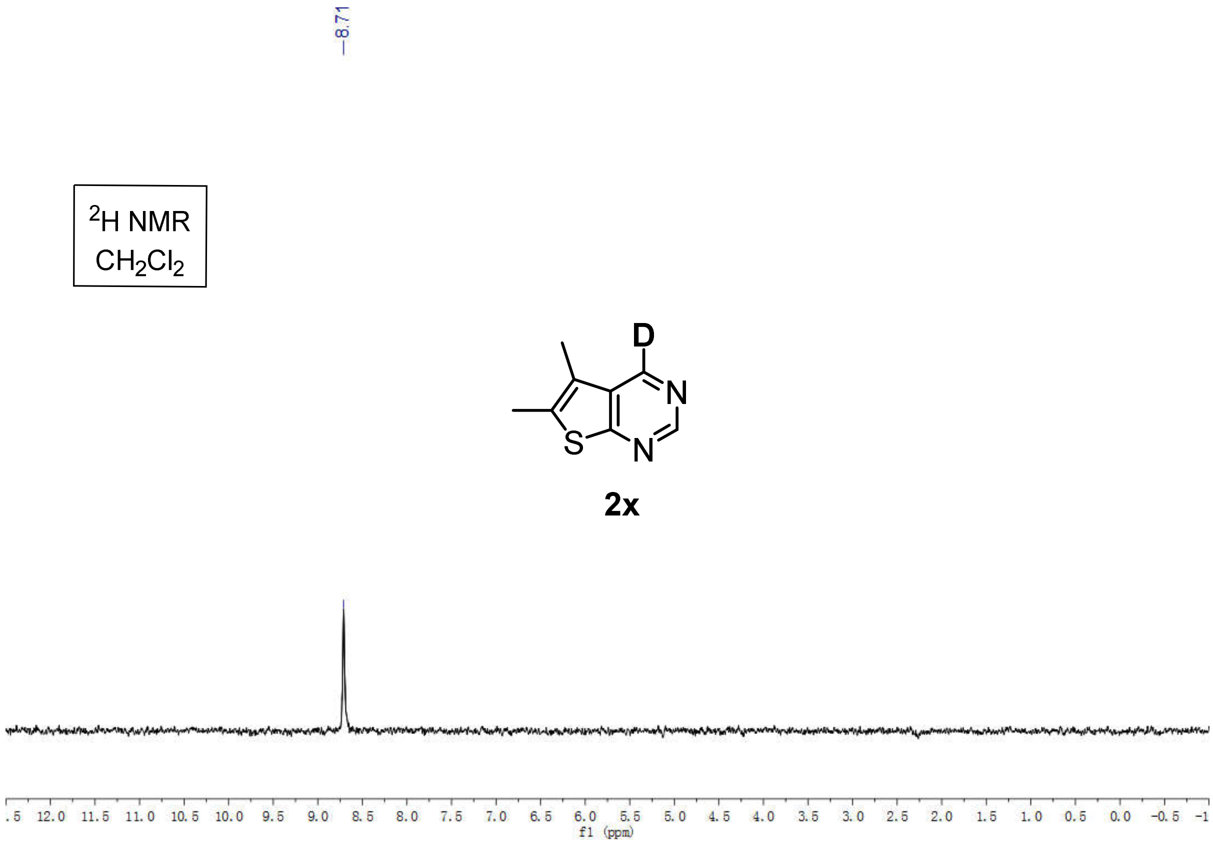

8.71
$^2$H NMR
$CH_2Cl_2$
D
N
S
N
2x
.5 12.0 11.5 11.0 10.5 10.0 9.5 9.0 8.5 8.0 7.5 7.0 6.5 6.0 5.5 5.0 4.5 4.0 3.5 3.0 2.5 2.0 1.5 1.0 0.5 0.0 -0.5 -1
f1 (ppm)

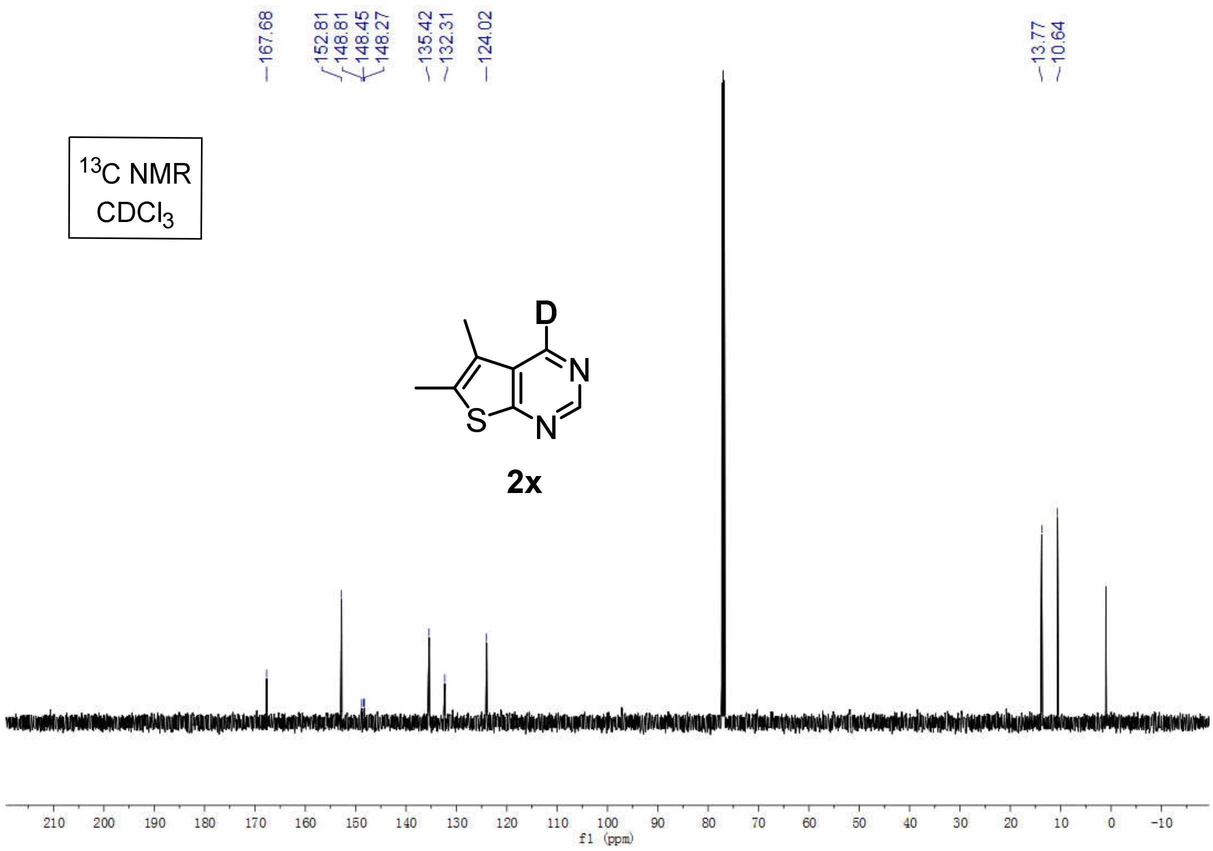


**Supplementary Fig. 66** $^{1}$H NMR, $^{2}$H NMR and $^{13}$C NMR spectra of the compound **2x**.

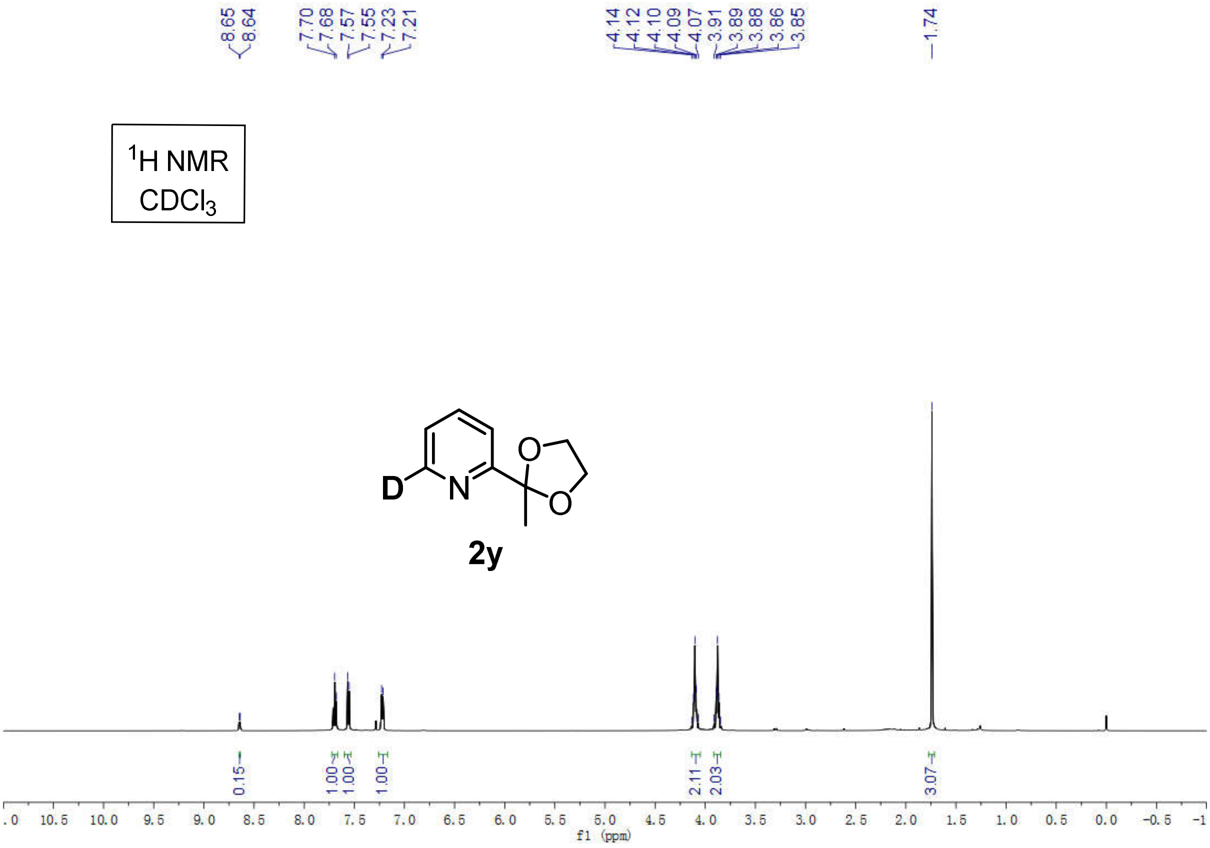
1H NMR
CDCl3
2y
f1 (ppm)

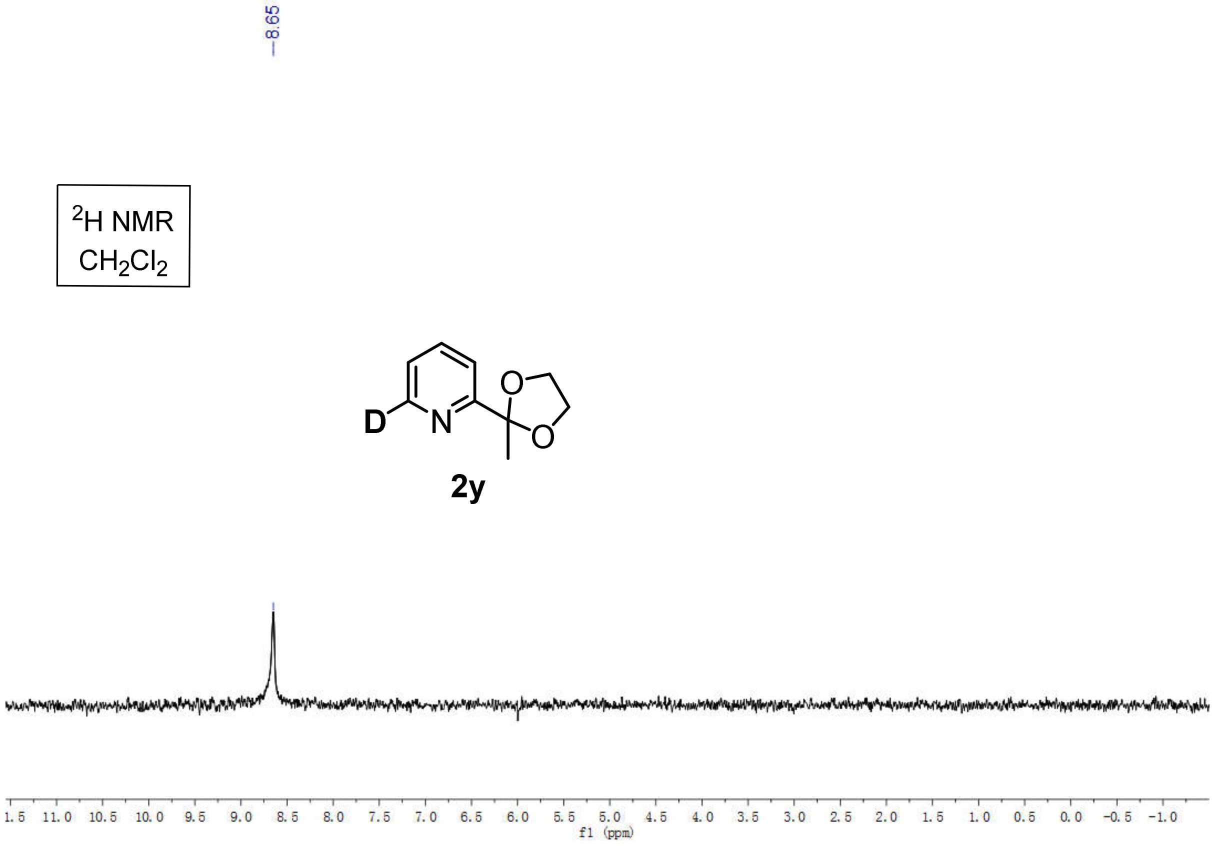
2H NMR
CH2Cl2
2y
f1 (ppm)

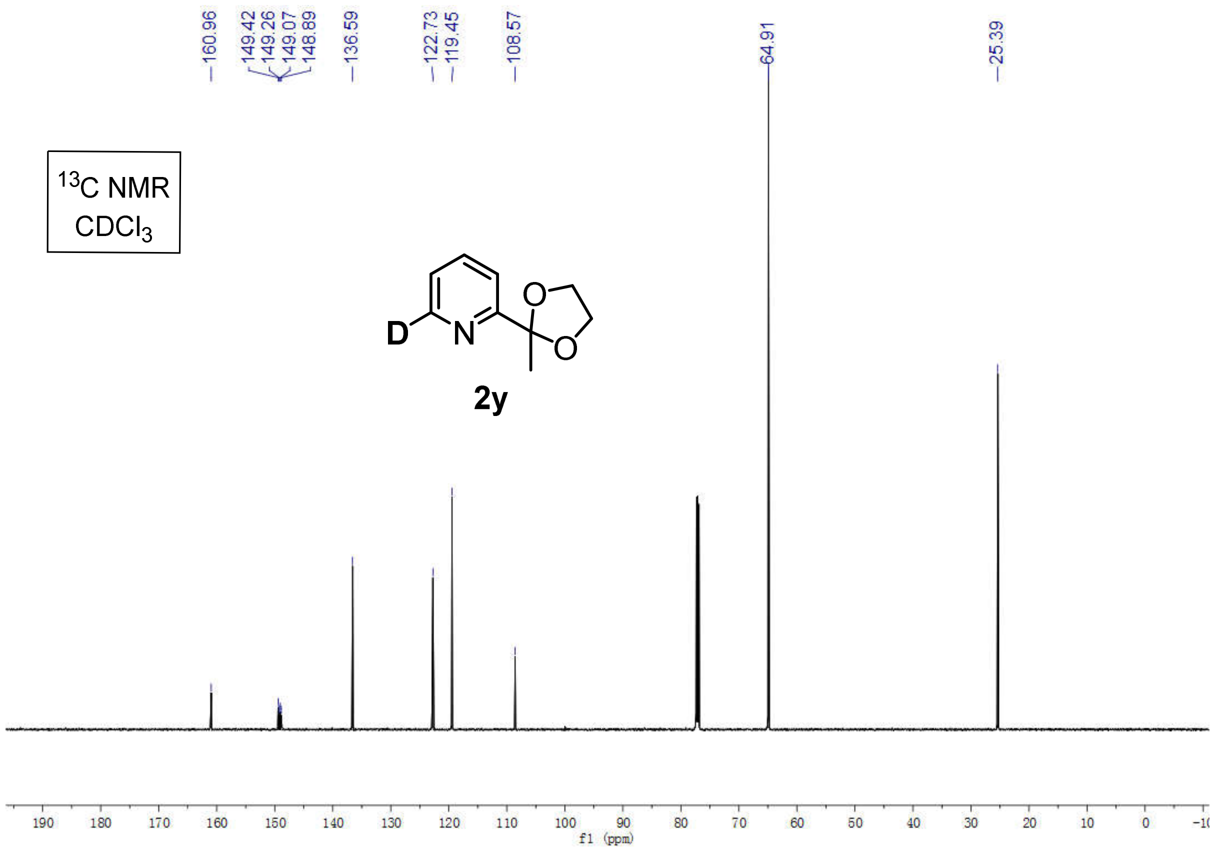


**Supplementary Fig. 67** $^{1}$H NMR, $^{2}$H NMR and $^{13}$C NMR spectra of the compound **2y**.

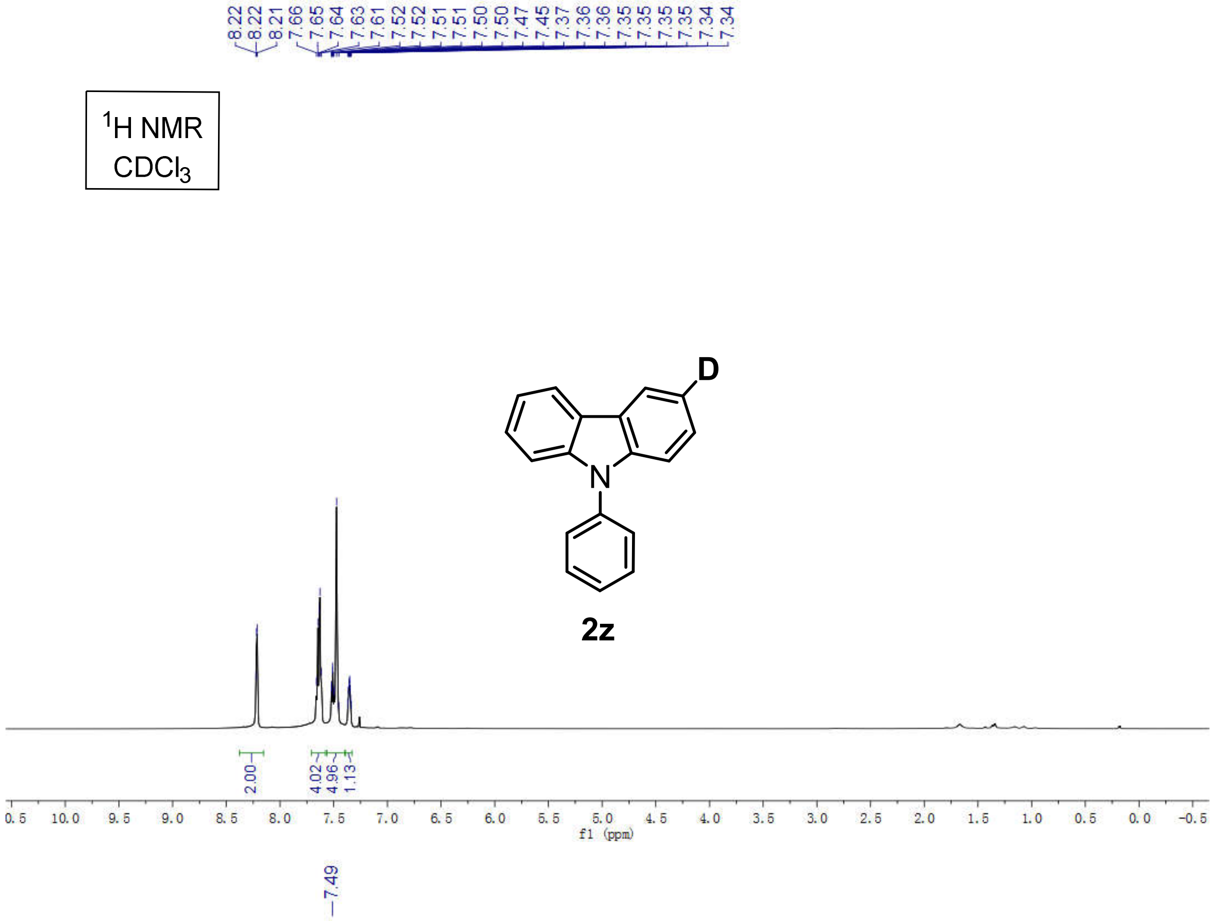
8.22
8.22
8.21
7.66
7.65
7.64
7.63
7.61
7.52
7.52
7.51
7.51
7.50
7.50
7.47
7.45
7.37
7.36
7.36
7.35
7.35
7.35
7.35
7.34
7.34
$^1$H NMR
$CDCl_3$
D
N
2z
2.00
4.02
4.96
1.13
f1 (ppm)
7.49

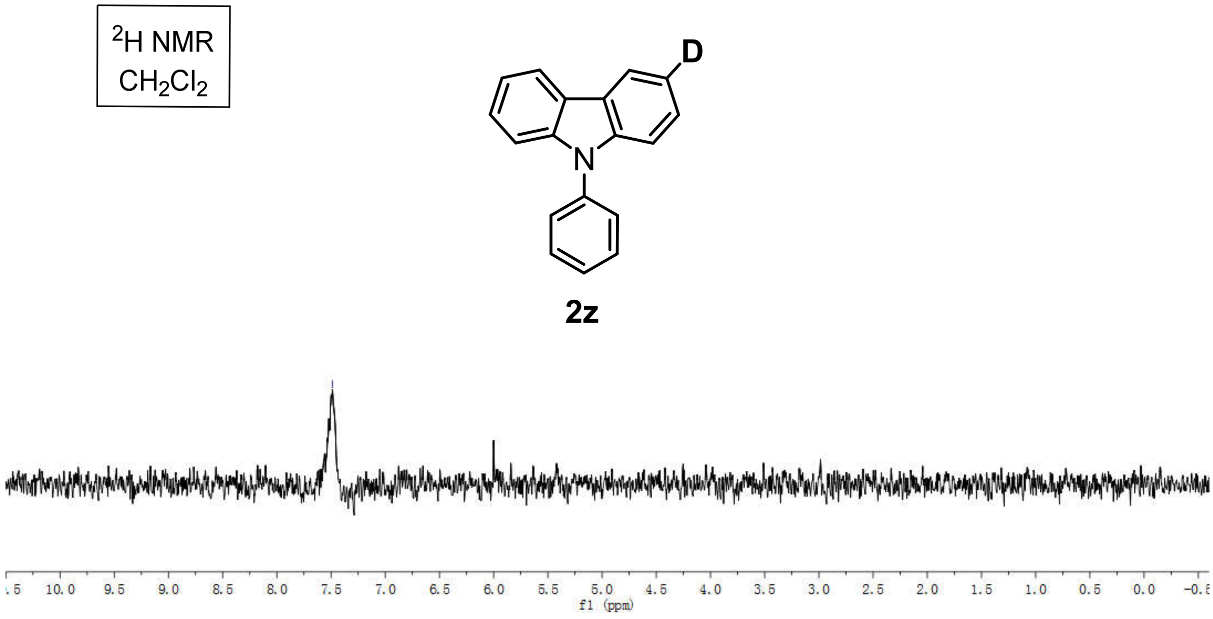
$^2$H NMR
$CH_2Cl_2$
D
N
2z
f1 (ppm)

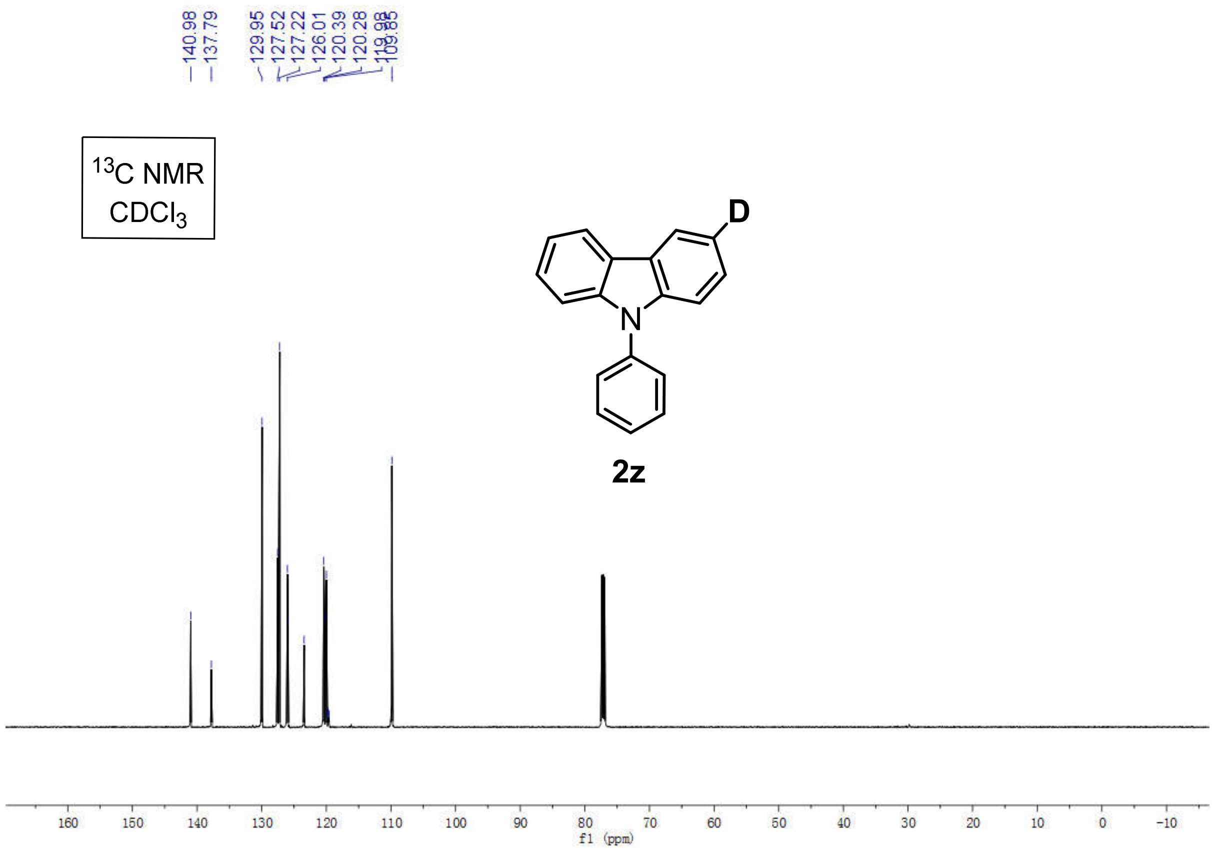


**Supplementary Fig. 68** $^{1}$H NMR, $^{2}$H NMR and $^{13}$C NMR spectra of the compound **2z**.

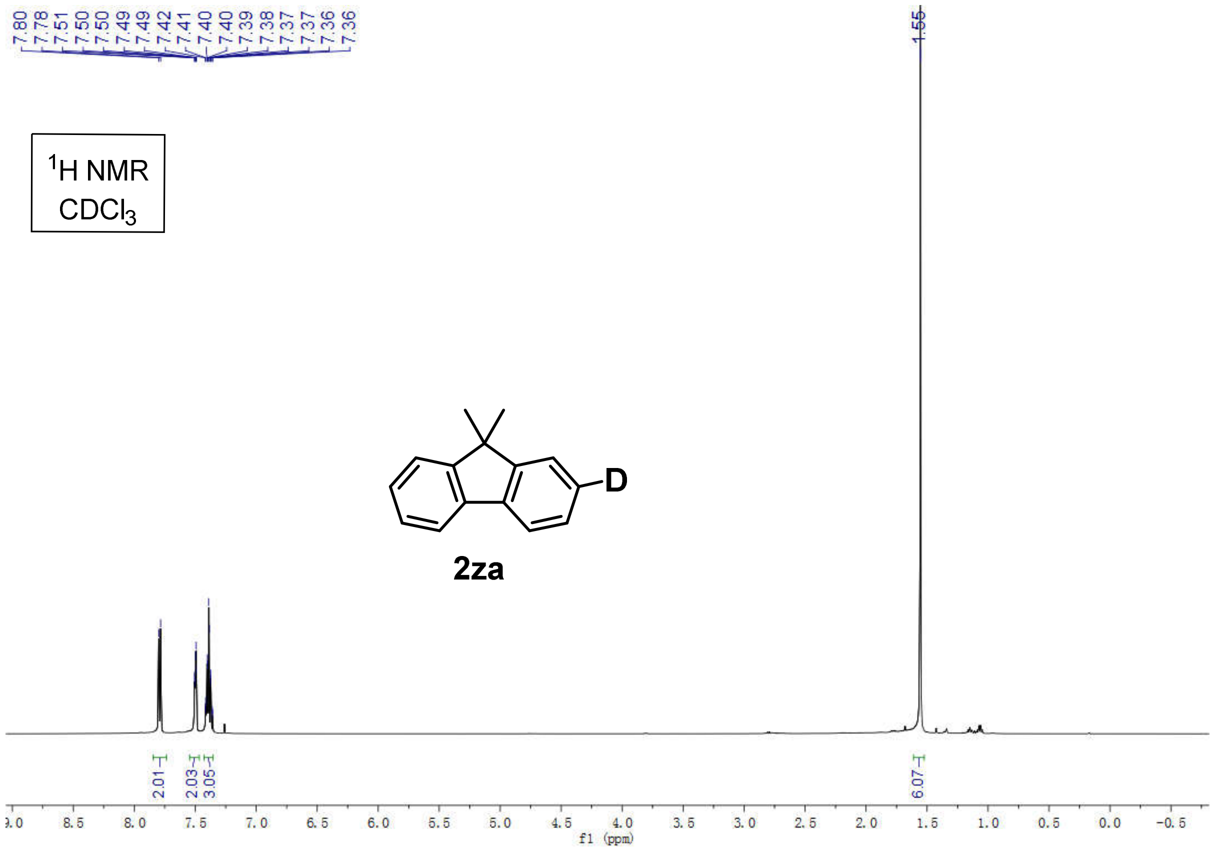
7.80 7.78 7.51 7.50 7.50 7.49 7.49 7.42 7.41 7.40 7.40 7.39 7.38 7.37 7.37 7.36 7.36
1.55
1H NMR
CDCl3
D
2za
2.01
2.03
3.05
6.07
9.0 8.5 8.0 7.5 7.0 6.5 6.0 5.5 5.0 4.5 4.0 3.5 3.0 2.5 2.0 1.5 1.0 0.5 0.0 -0.5
f1 (ppm)

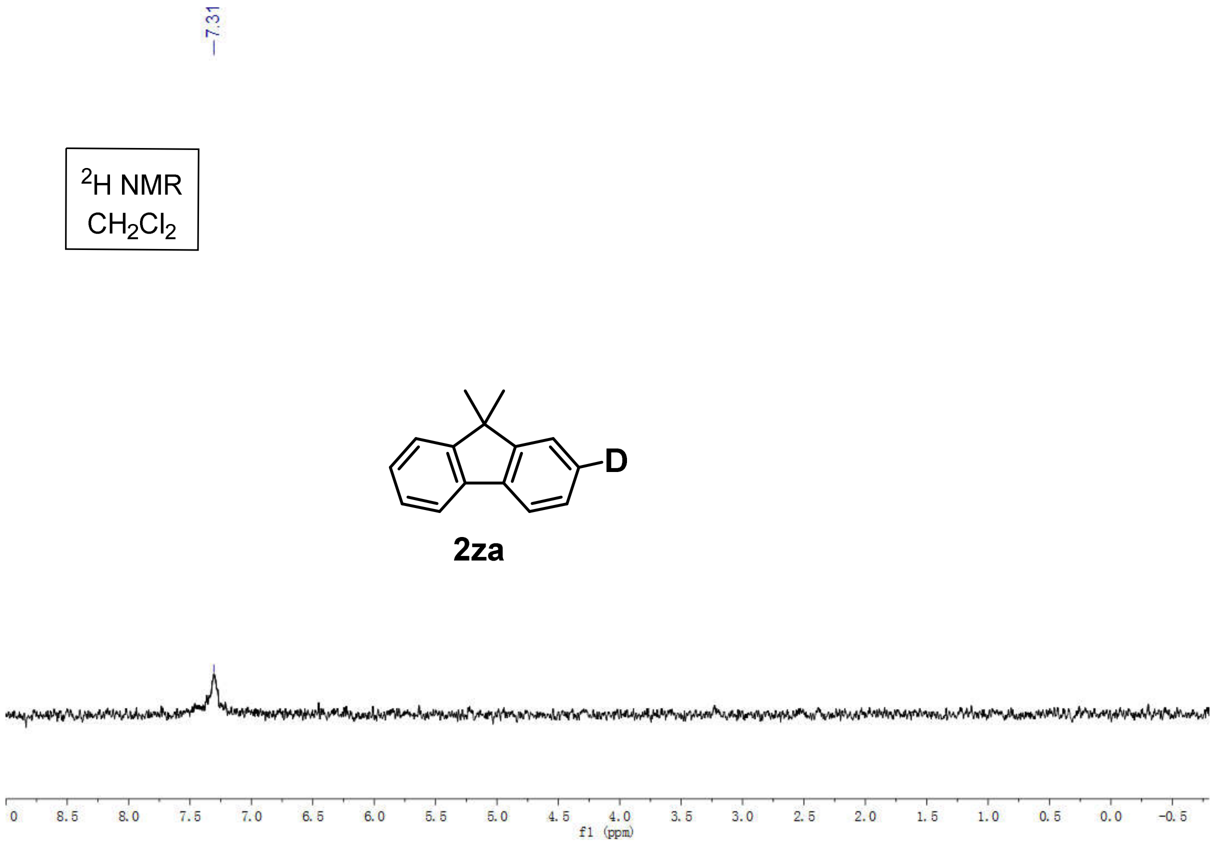
7.31
2H NMR
CH2Cl2
D
2za
0 8.5 8.0 7.5 7.0 6.5 6.0 5.5 5.0 4.5 4.0 3.5 3.0 2.5 2.0 1.5 1.0 0.5 0.0 -0.5
f1 (ppm)

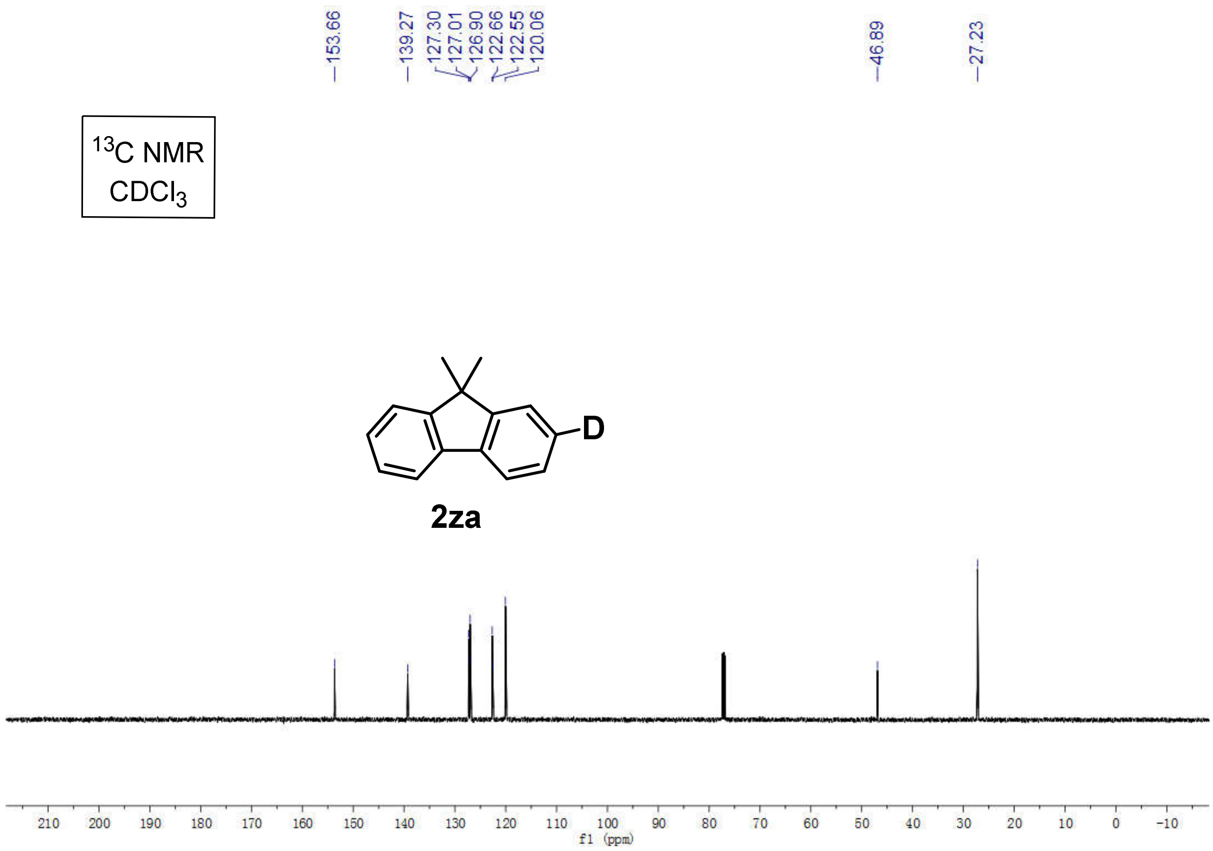


**Supplementary Fig. 69** $^{1}$H NMR, $^{2}$H NMR and $^{13}$C NMR spectra of the compound **2za**.

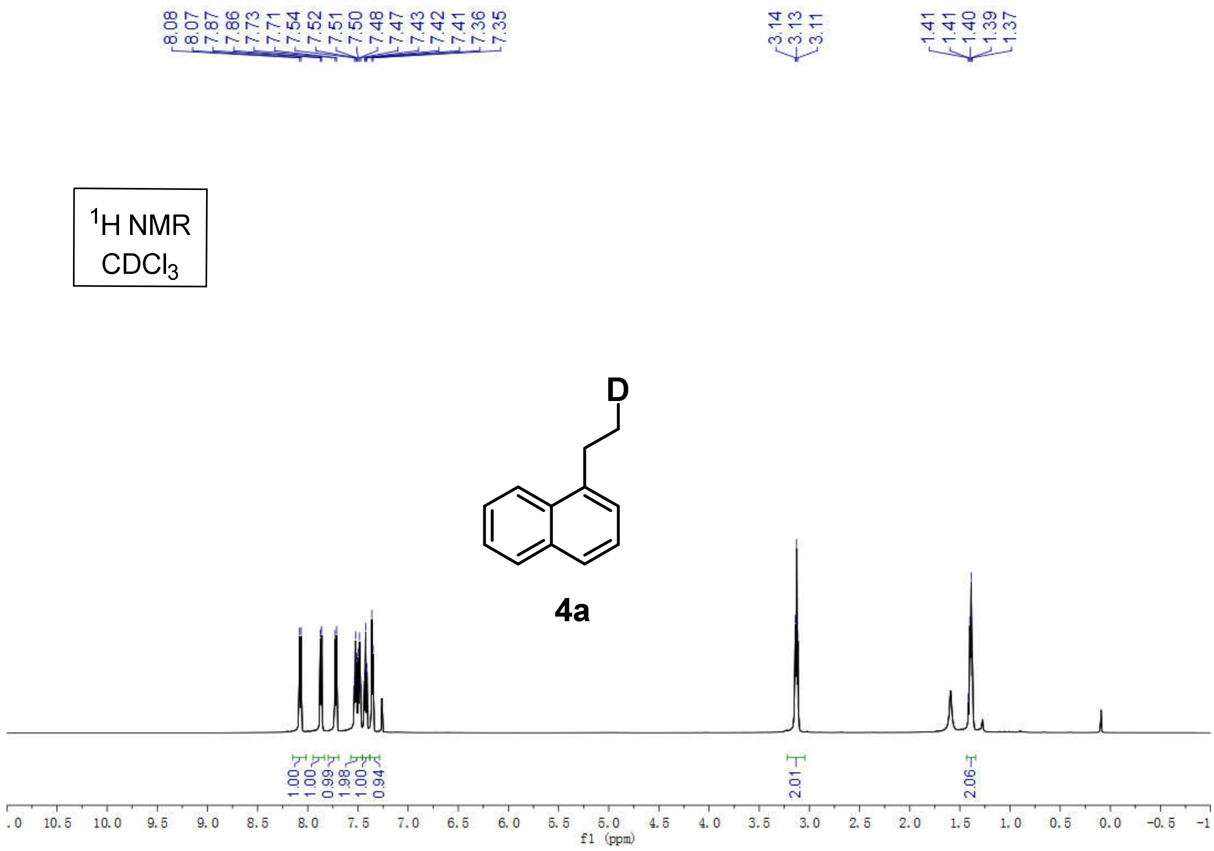
8.08
8.07
7.87
7.86
7.73
7.71
7.54
7.52
7.51
7.50
7.48
7.47
7.43
7.42
7.41
7.36
7.35
3.14
3.13
3.11
1.41
1.41
1.40
1.39
1.37
1H NMR
CDCl3
D
4a
1.00
1.00
0.99
0.99
1.98
1.00
0.94
2.01
2.06
f1 (ppm)

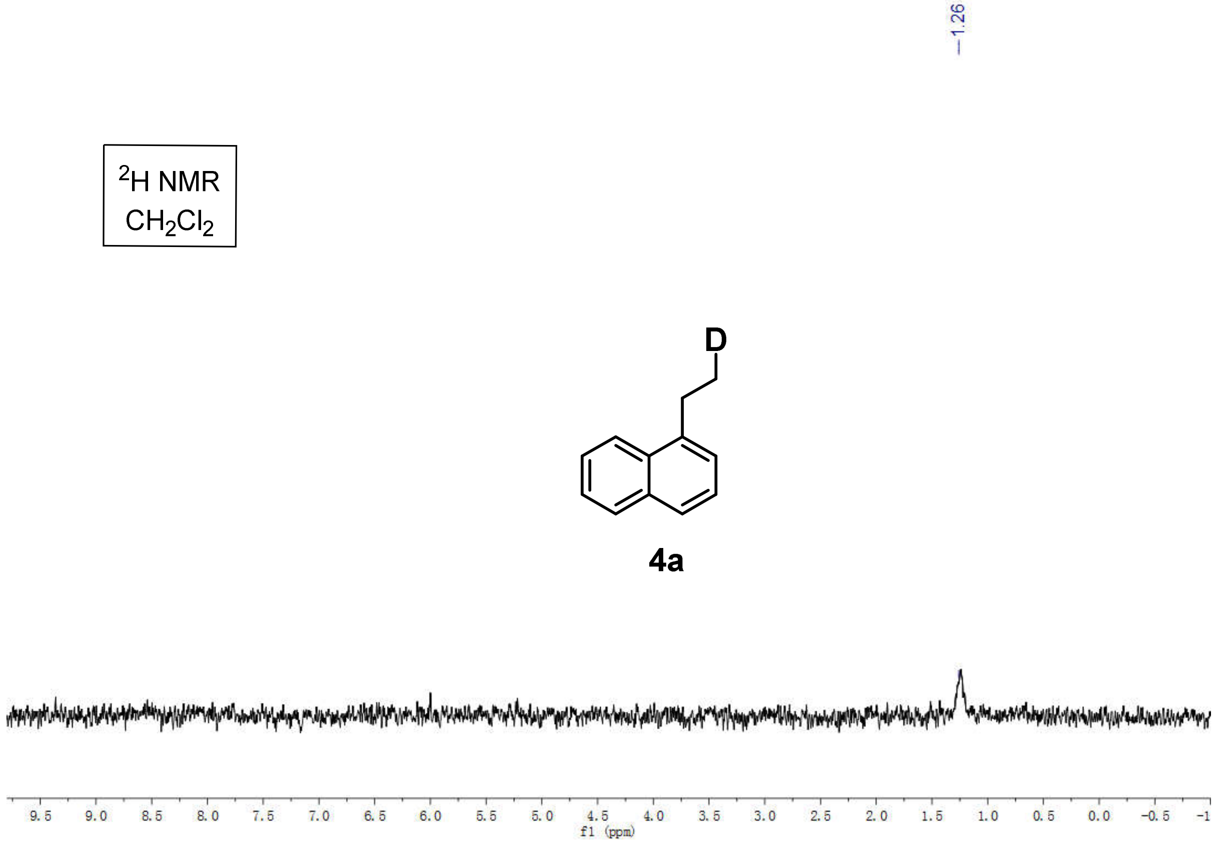
1.26
2H NMR
CH2Cl2
D
4a
f1 (ppm)

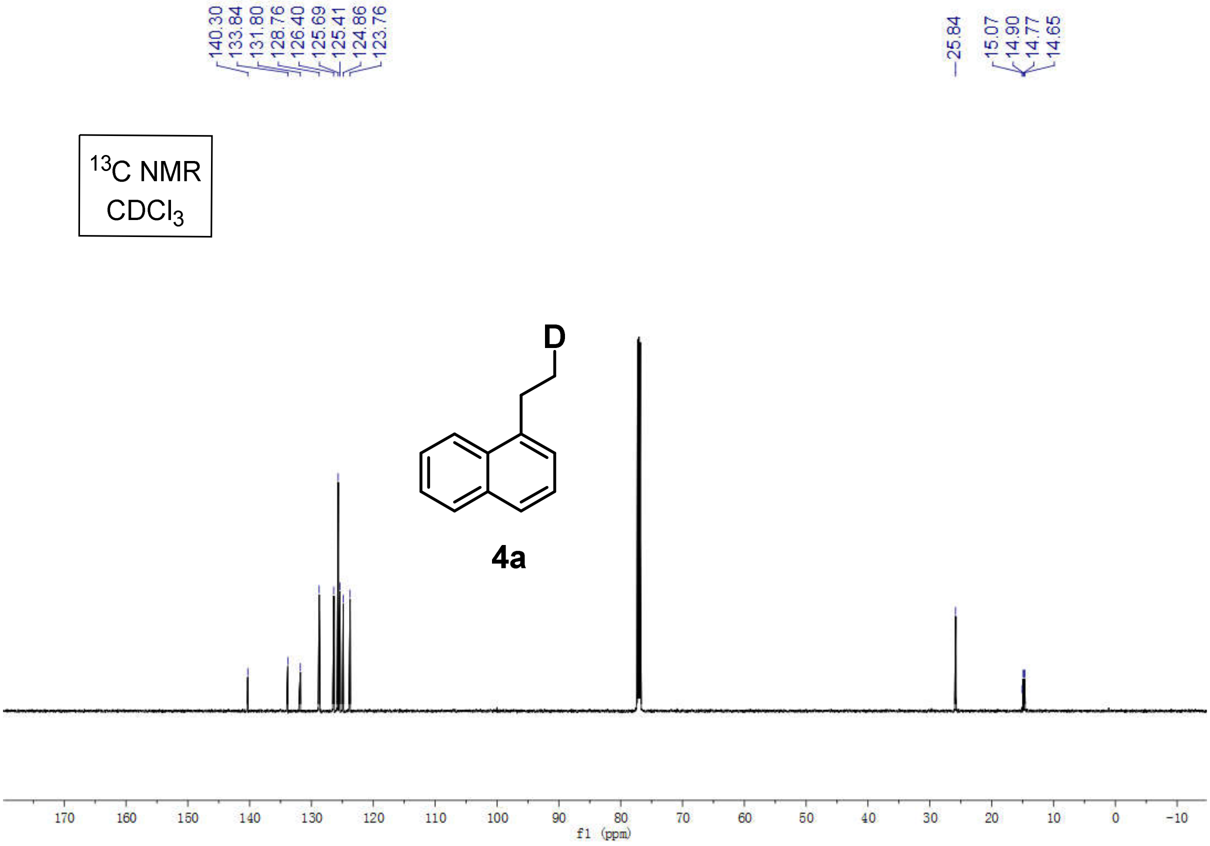


**Supplementary Fig. 70** $^{1}$H NMR, $^{2}$H NMR and $^{13}$C NMR spectra of the compound **4a**.

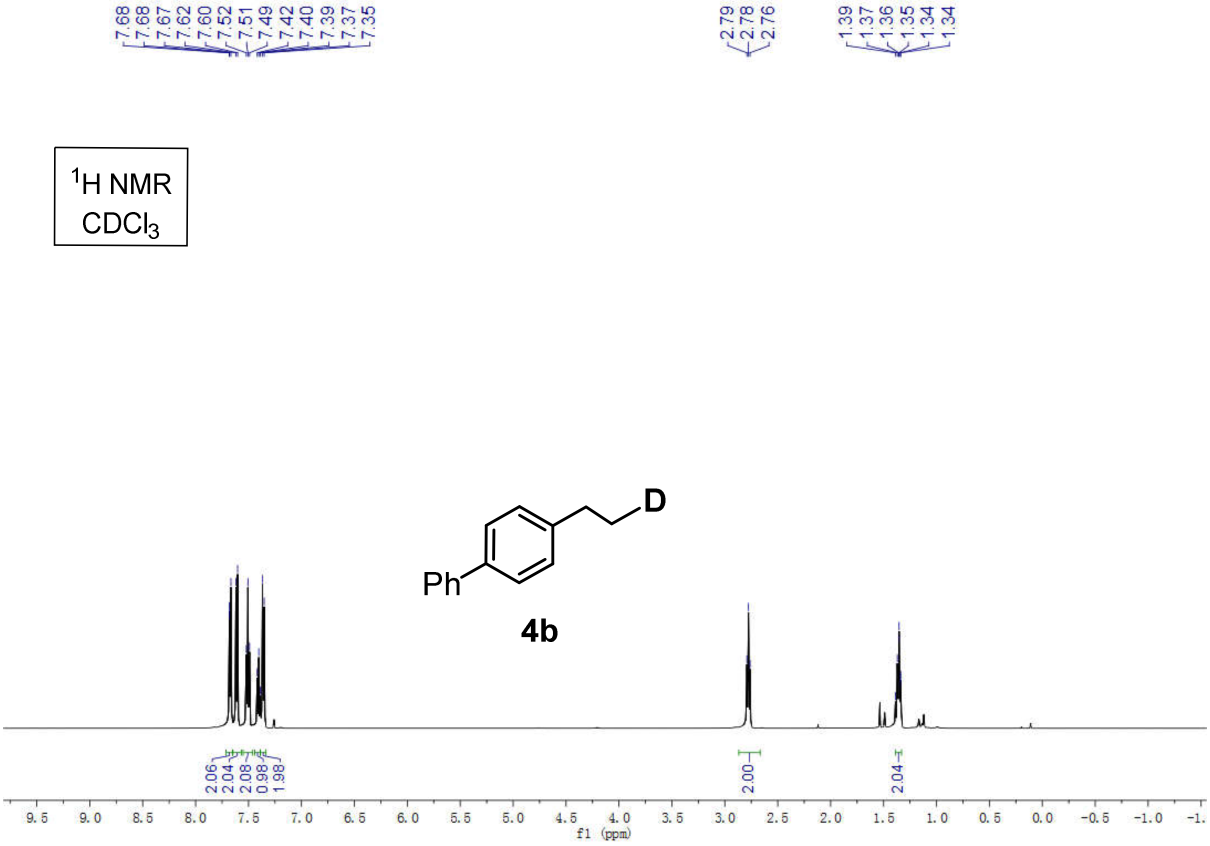
7.68
7.68
7.67
7.62
7.60
7.52
7.51
7.49
7.42
7.40
7.39
7.37
7.35
2.79
2.78
2.76
1.39
1.37
1.36
1.35
1.34
1.34
$^1$H NMR
$CDCl_3$
D
Ph
4b
2.06
2.04
2.08
0.98
1.98
2.00
2.04
9.5 9.0 8.5 8.0 7.5 7.0 6.5 6.0 5.5 5.0 4.5 4.0 3.5 3.0 2.5 2.0 1.5 1.0 0.5 0.0 -0.5 -1.0 -1.
f1 (ppm)

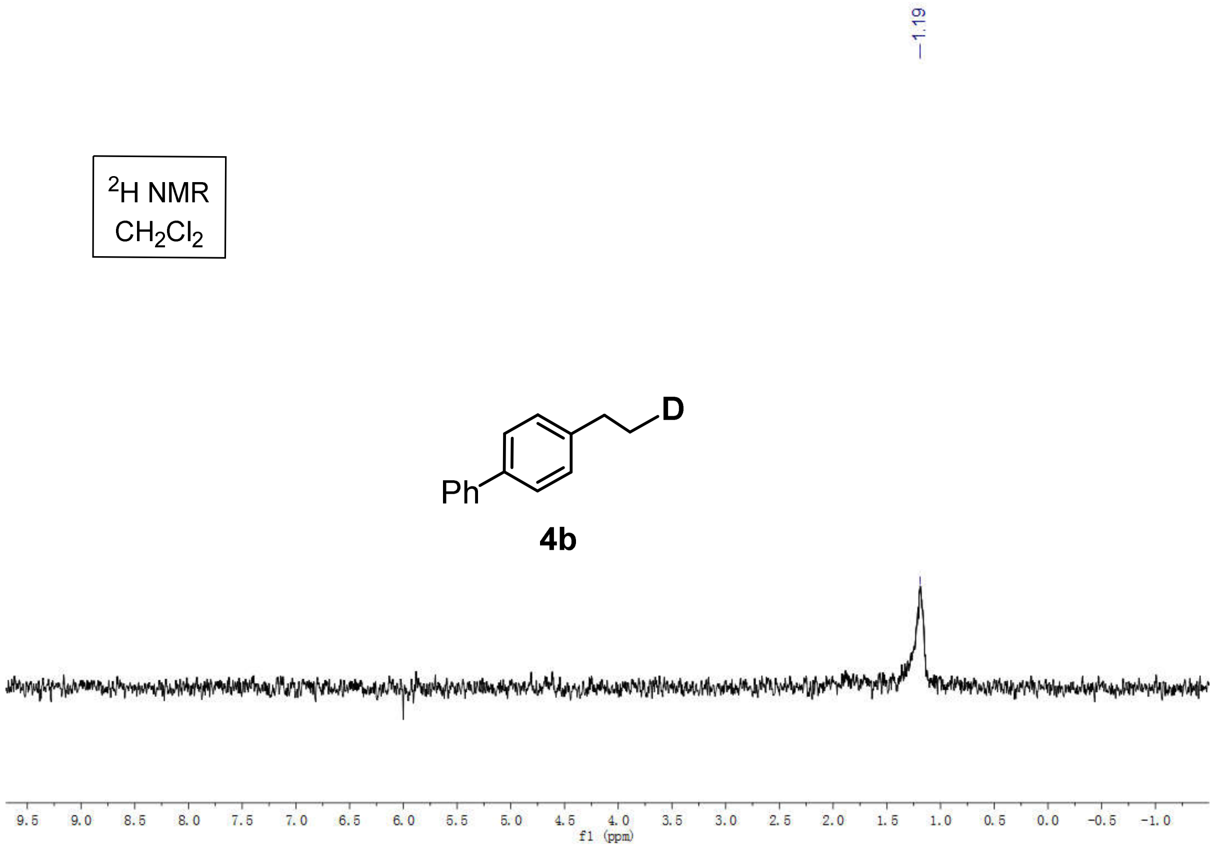
1.19
$^2$H NMR
$CH_2Cl_2$
D
Ph
4b
9.5 9.0 8.5 8.0 7.5 7.0 6.5 6.0 5.5 5.0 4.5 4.0 3.5 3.0 2.5 2.0 1.5 1.0 0.5 0.0 -0.5 -1.0
f1 (ppm)

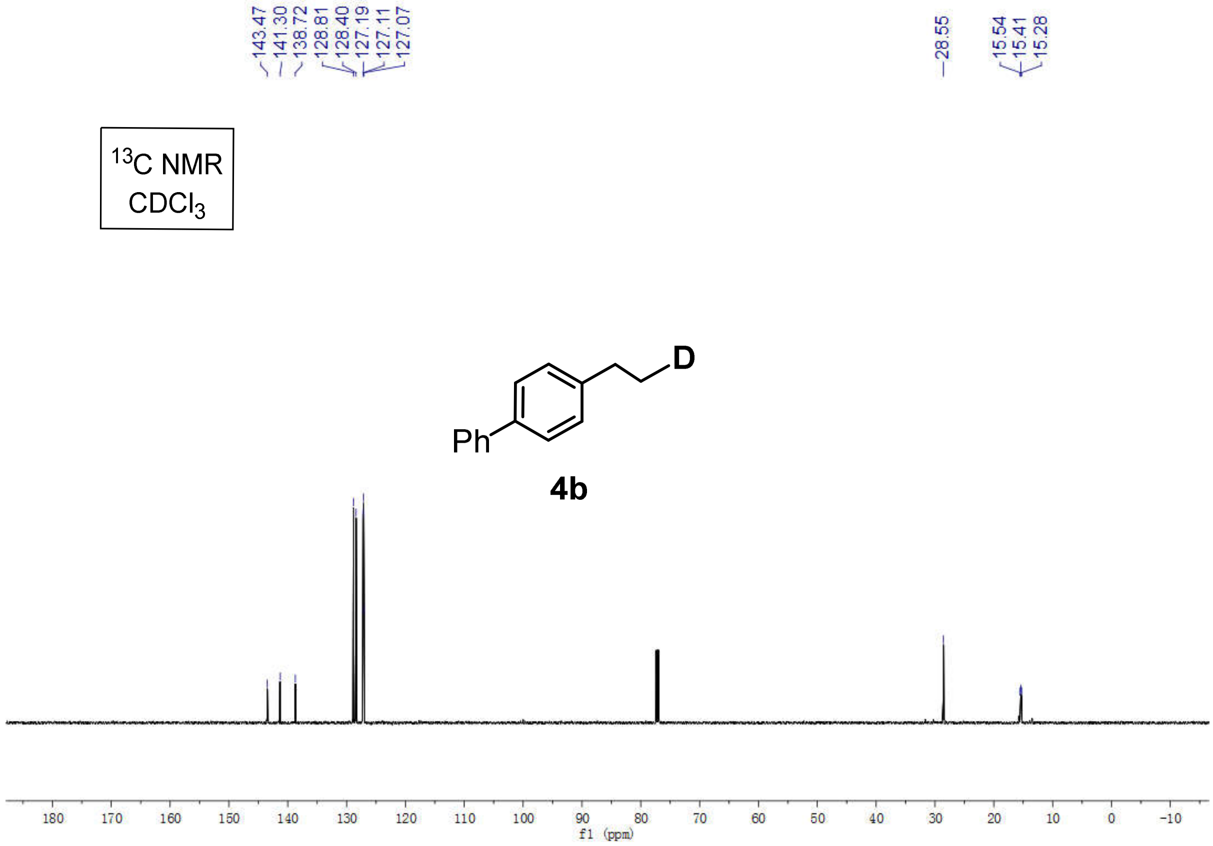


**Supplementary Fig. 71** $^{1}$H NMR, $^{2}$H NMR and $^{13}$C NMR spectra of the compound **4b**.

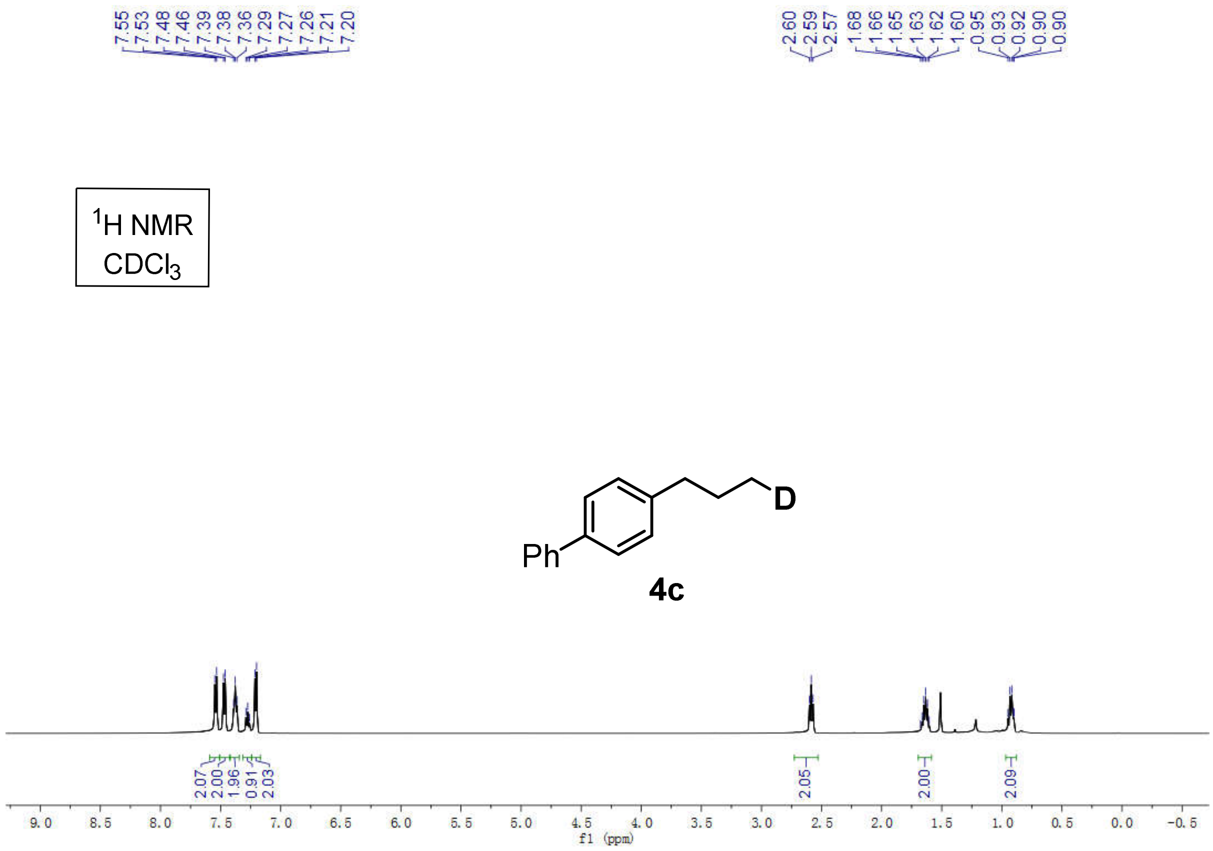
7.55
7.53
7.48
7.46
7.39
7.38
7.36
7.29
7.27
7.26
7.21
7.20
2.60
2.59
2.57
1.68
1.66
1.65
1.63
1.62
1.60
0.95
0.93
0.92
0.90
0.90
1H NMR
CDCl3
D
Ph
4c
2.07
2.00
1.96
0.91
2.03
2.05
2.00
2.09
9.0 8.5 8.0 7.5 7.0 6.5 6.0 5.5 5.0 4.5 4.0 3.5 3.0 2.5 2.0 1.5 1.0 0.5 0.0 -0.5
f1 (ppm)

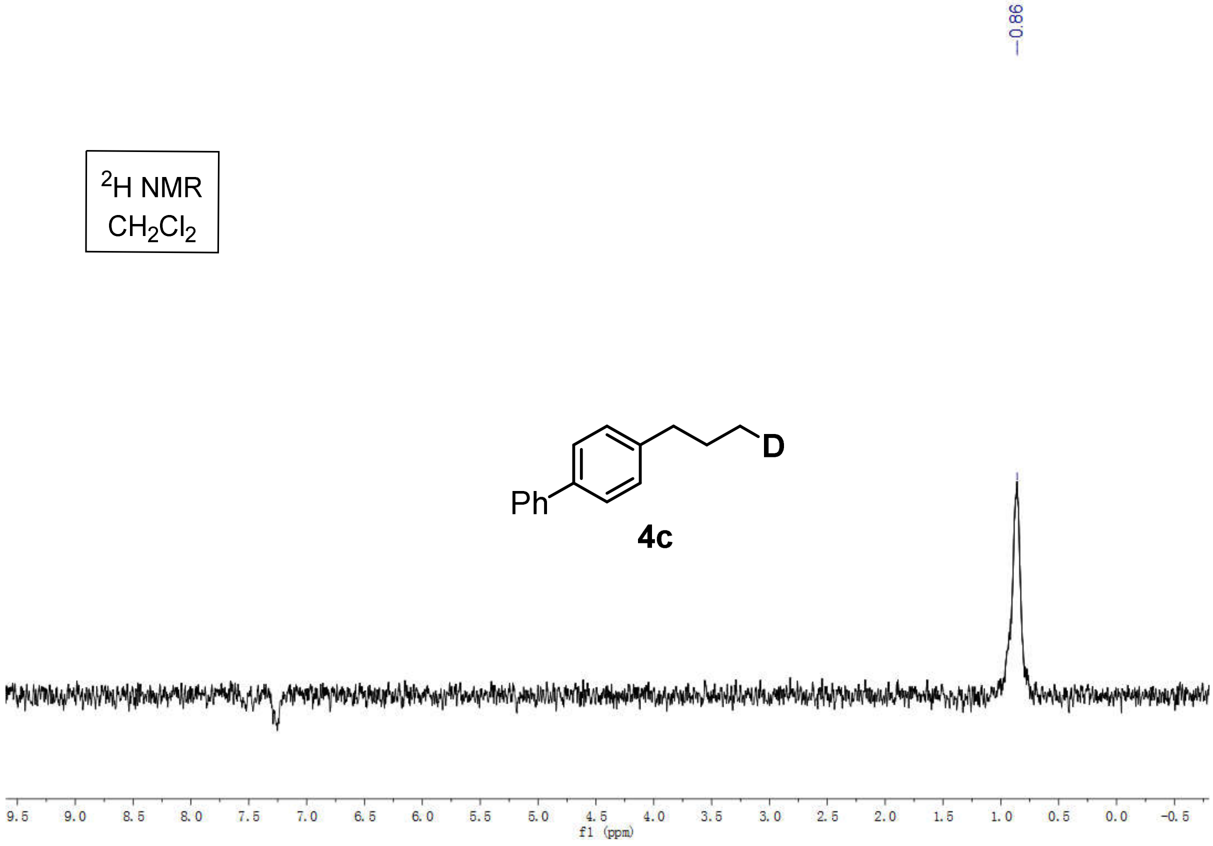
0.86
2H NMR
CH2Cl2
D
Ph
4c
9.5 9.0 8.5 8.0 7.5 7.0 6.5 6.0 5.5 5.0 4.5 4.0 3.5 3.0 2.5 2.0 1.5 1.0 0.5 0.0 -0.5
f1 (ppm)

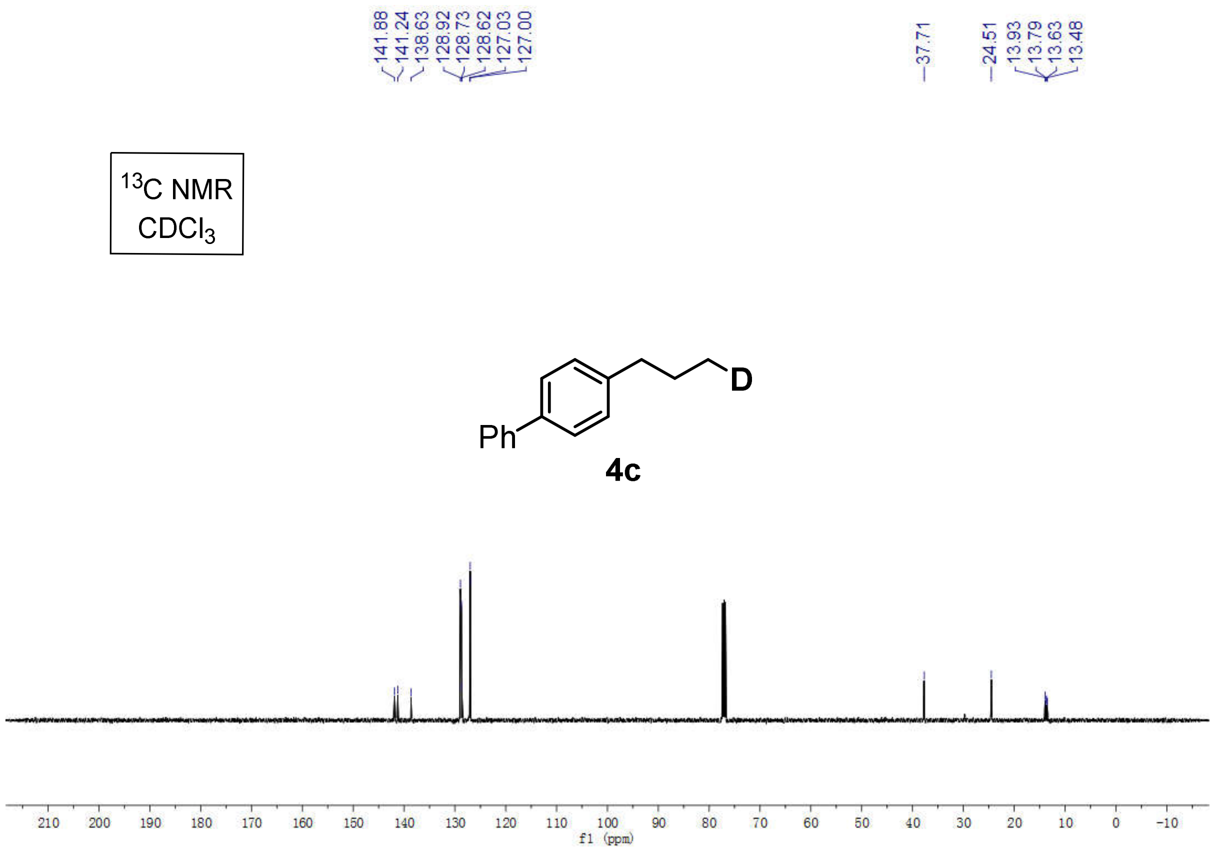


**Supplementary Fig. 72** $^{1}$H NMR, $^{2}$H NMR and $^{13}$C NMR spectra of the compound **4c**.

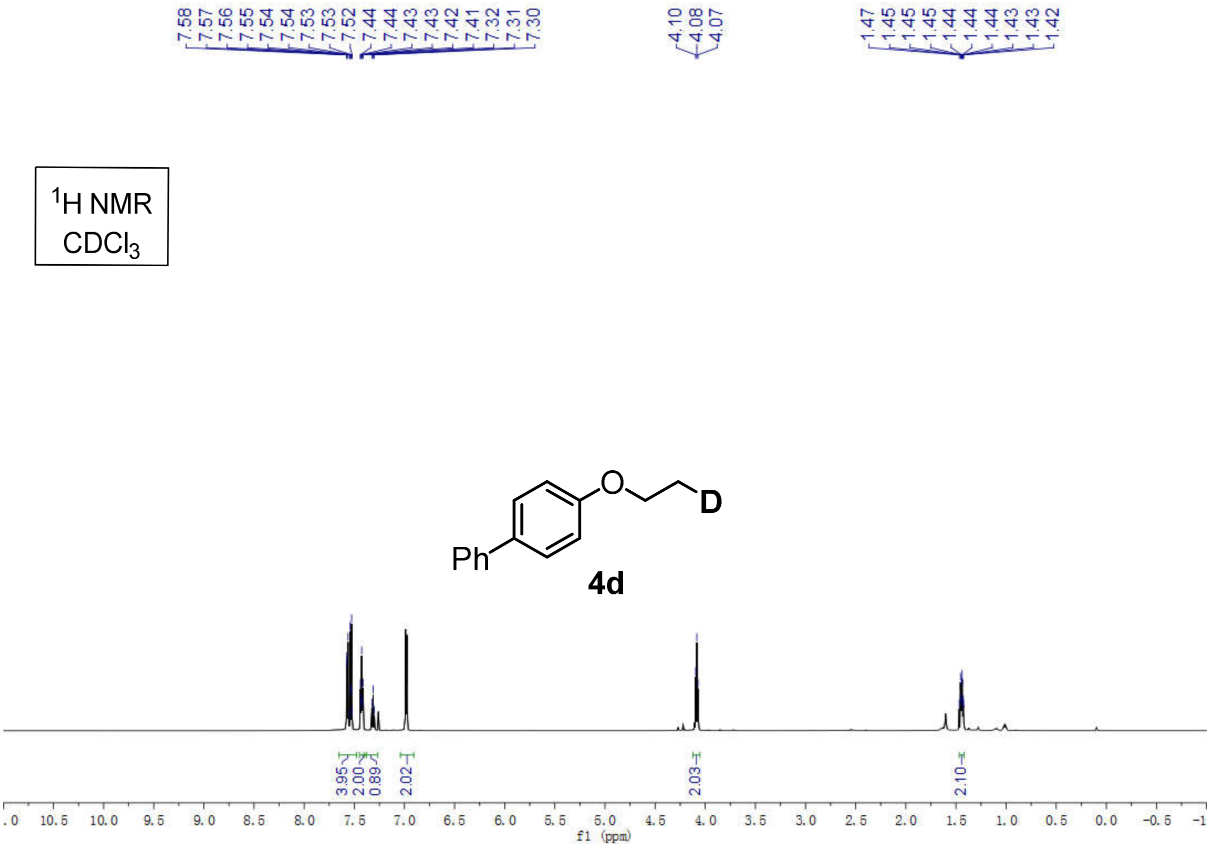
7.58 7.57 7.56 7.55 7.54 7.54 7.53 7.53 7.52 7.44 7.44 7.43 7.43 7.42 7.41 7.32 7.31 7.30
4.10 4.08 4.07
1.47 1.45 1.45 1.45 1.44 1.44 1.44 1.44 1.43 1.43 1.42
1H NMR
CDCl3
Ph
O
D
4d
3.95 2.00 0.89 2.02 2.03 2.10
f1 (ppm)

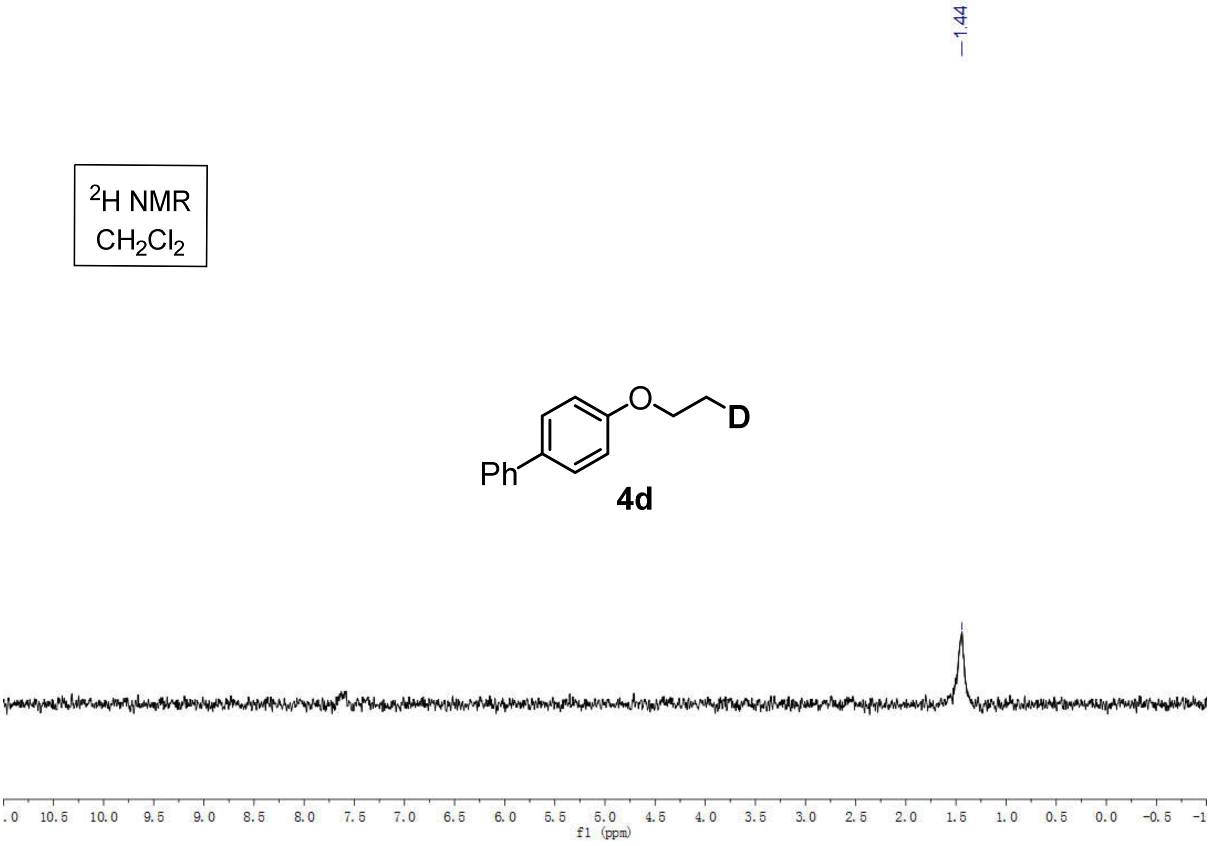
1.44
2H NMR
CH2Cl2
Ph
O
D
4d
f1 (ppm)

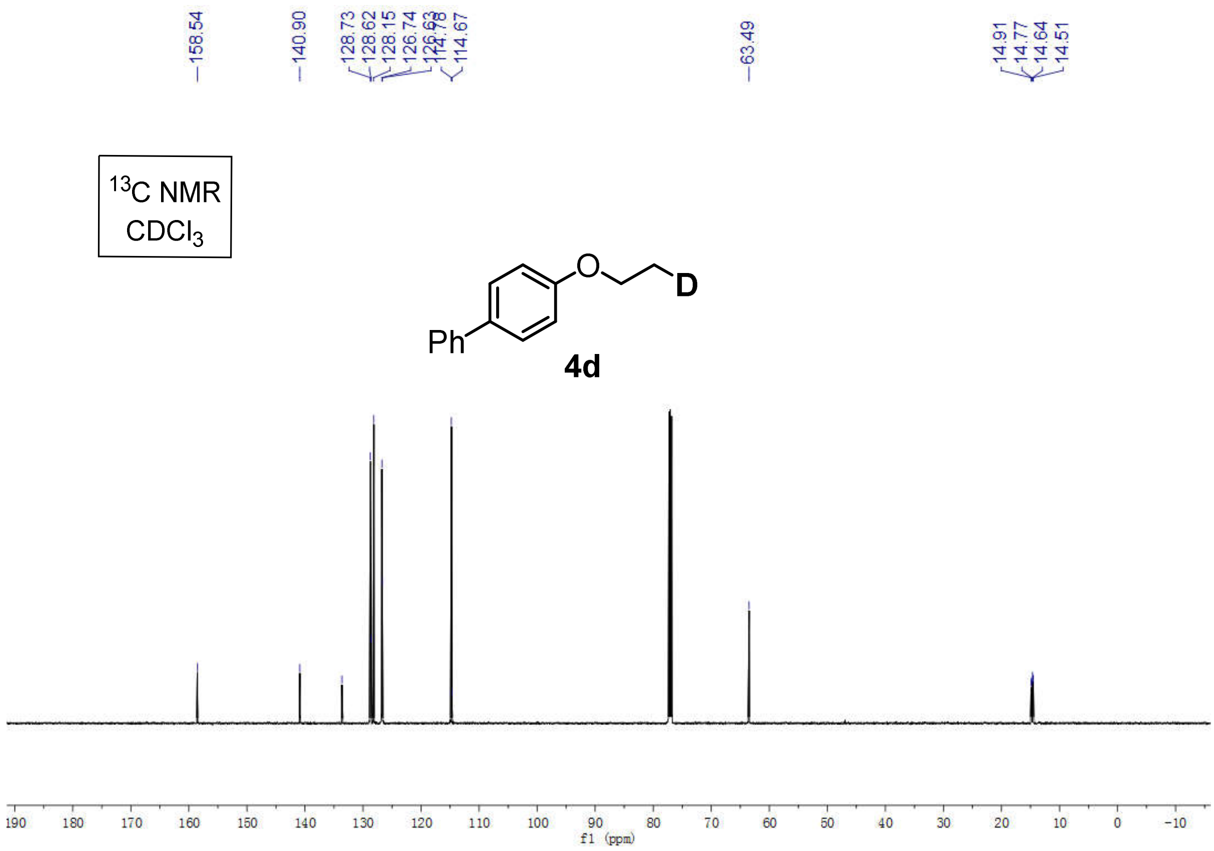


**Supplementary Fig. 73** $^{1}$H NMR, $^{2}$H NMR and $^{13}$C NMR spectra of the compound **4d**.

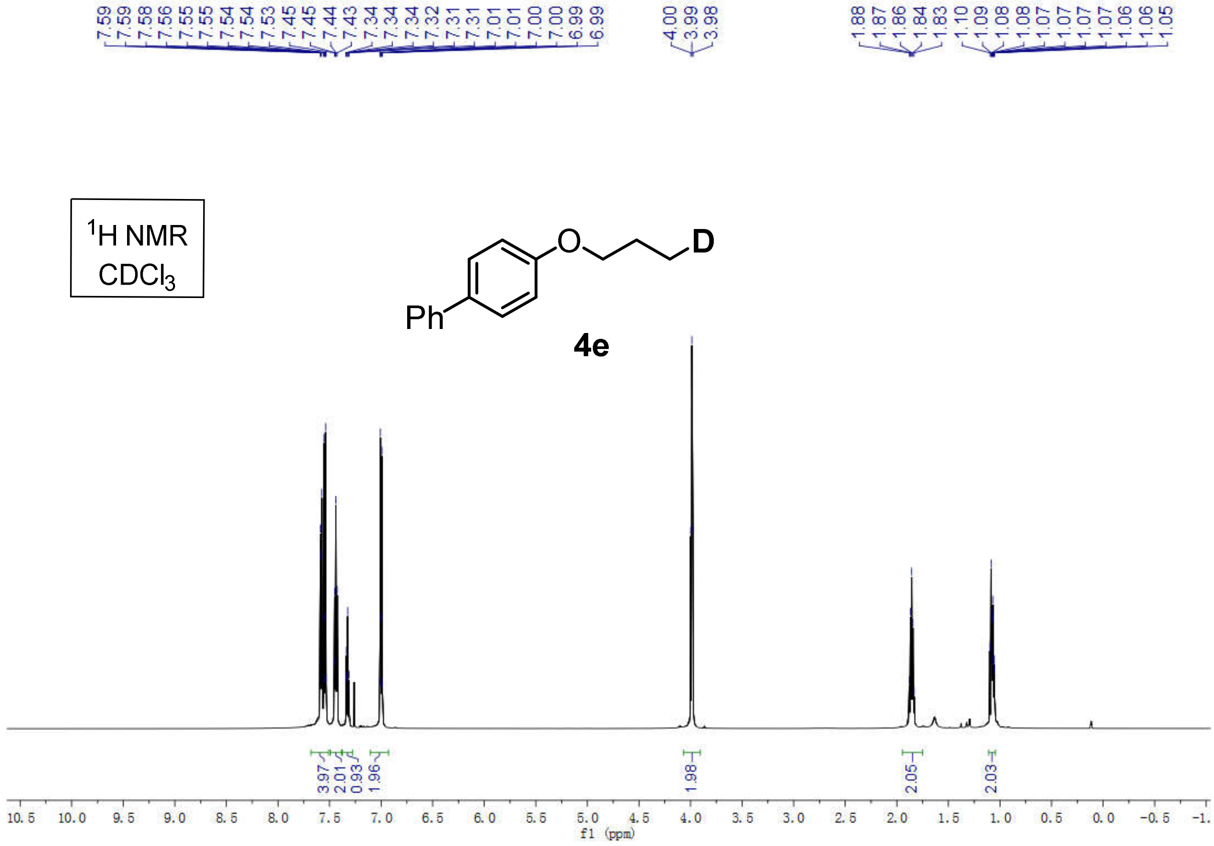
1H NMR
CDCl3
Ph
O
D
4e
f1 (ppm)

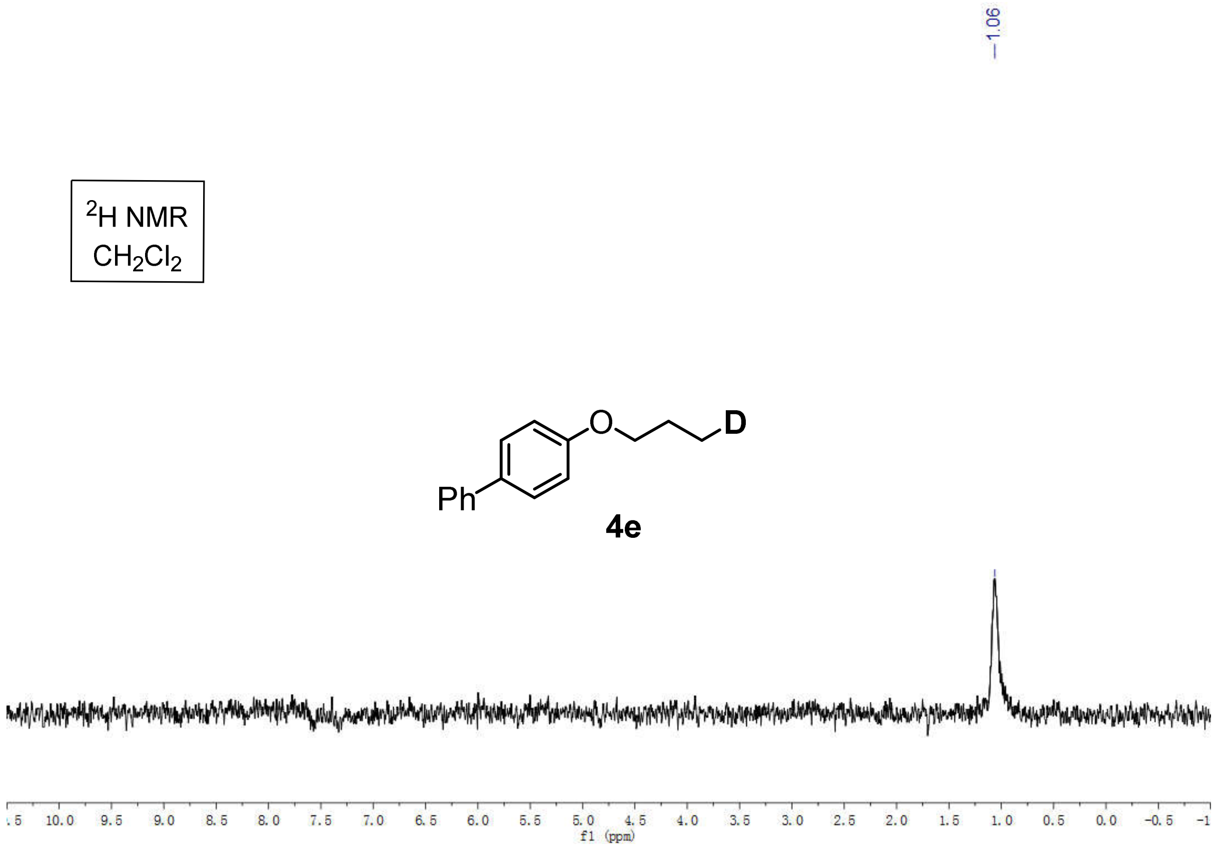
2H NMR
CH2Cl2
Ph
O
D
4e
f1 (ppm)

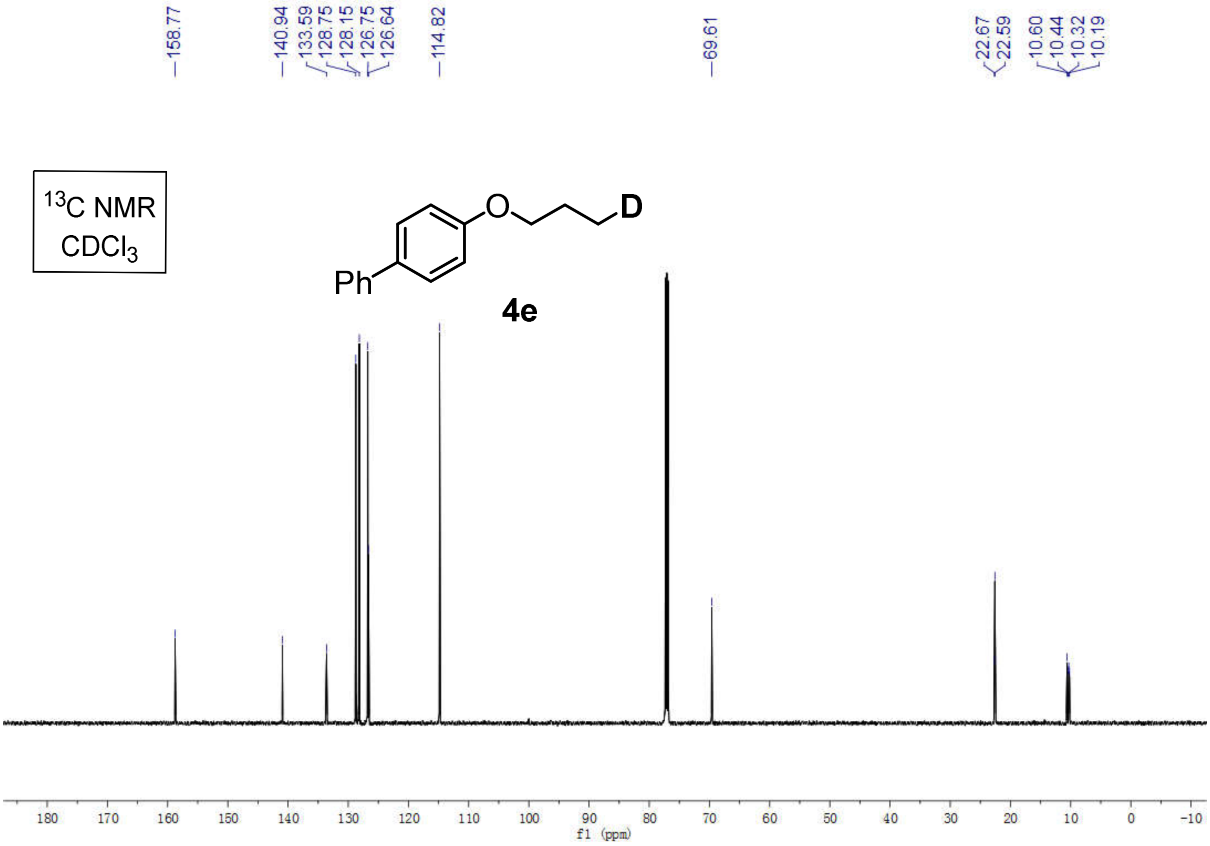


**Supplementary Fig. 74** $^{1}$H NMR, $^{2}$H NMR and $^{13}$C NMR spectra of the compound **4e**.

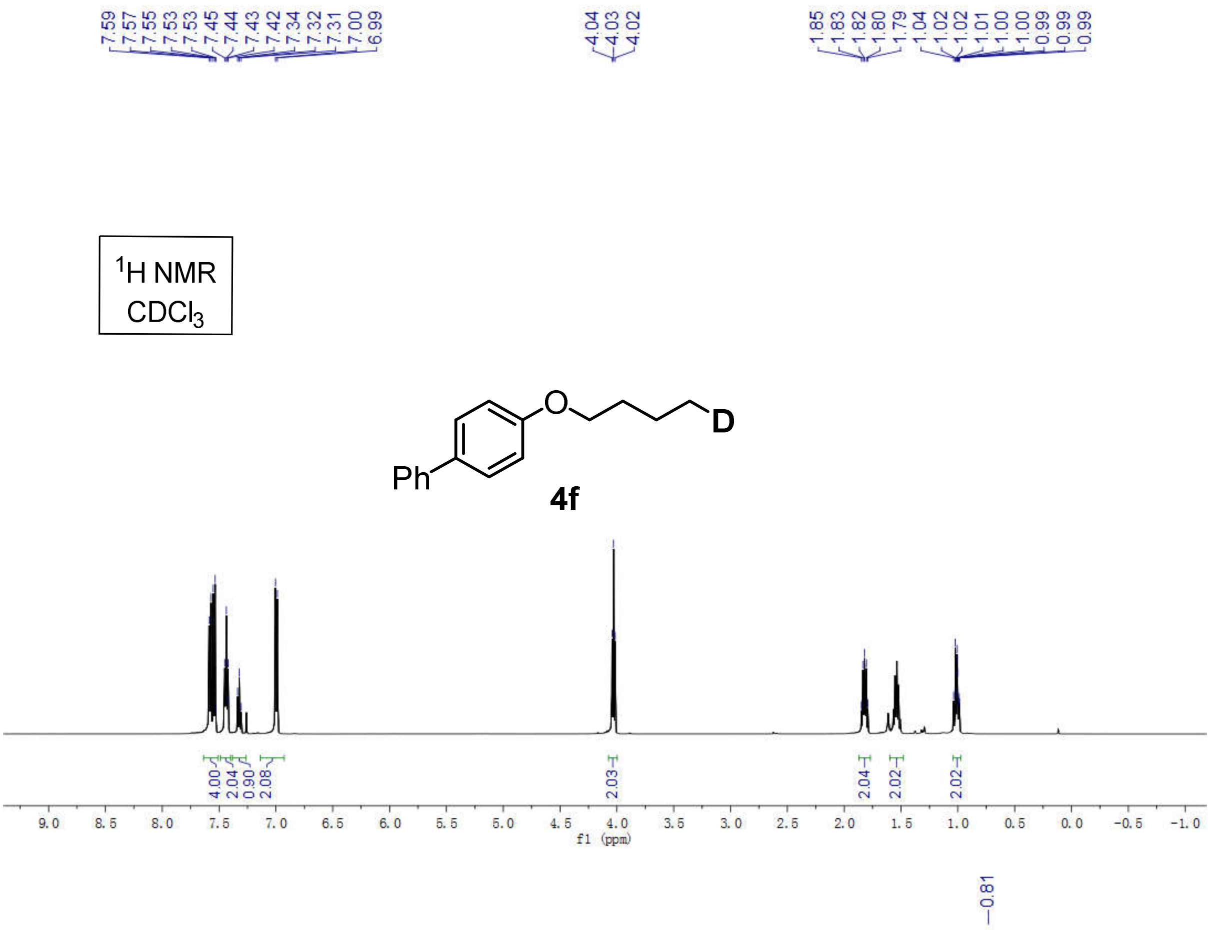
7.59
7.57
7.55
7.53
7.53
7.45
7.44
7.43
7.42
7.34
7.32
7.31
7.00
6.99
4.04
4.03
4.02
1.85
1.83
1.82
1.80
1.79
1.04
1.02
1.02
1.01
1.00
1.00
0.99
0.99
0.99
$^1$H NMR
$CDCl_3$
O
D
Ph
4f
4.00
2.04
0.90
2.08
2.03
2.04
2.02
2.02
f1 (ppm)

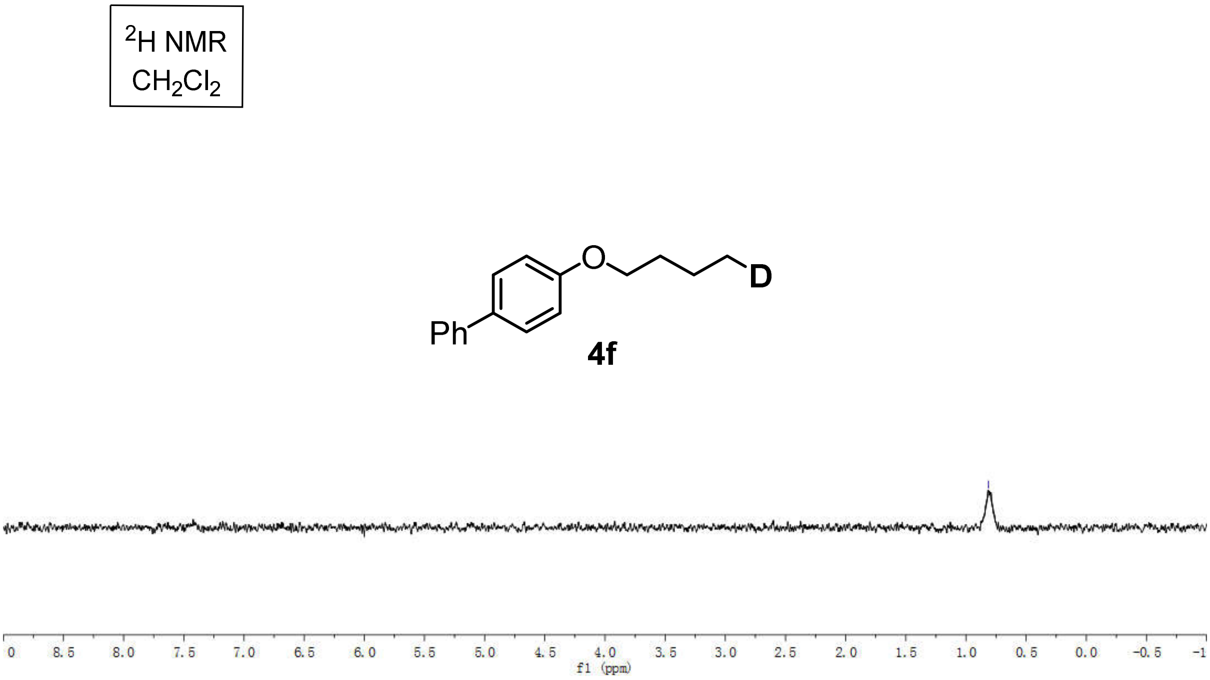
0.81
$^2$H NMR
$CH_2Cl_2$
O
D
Ph
4f
f1 (ppm)

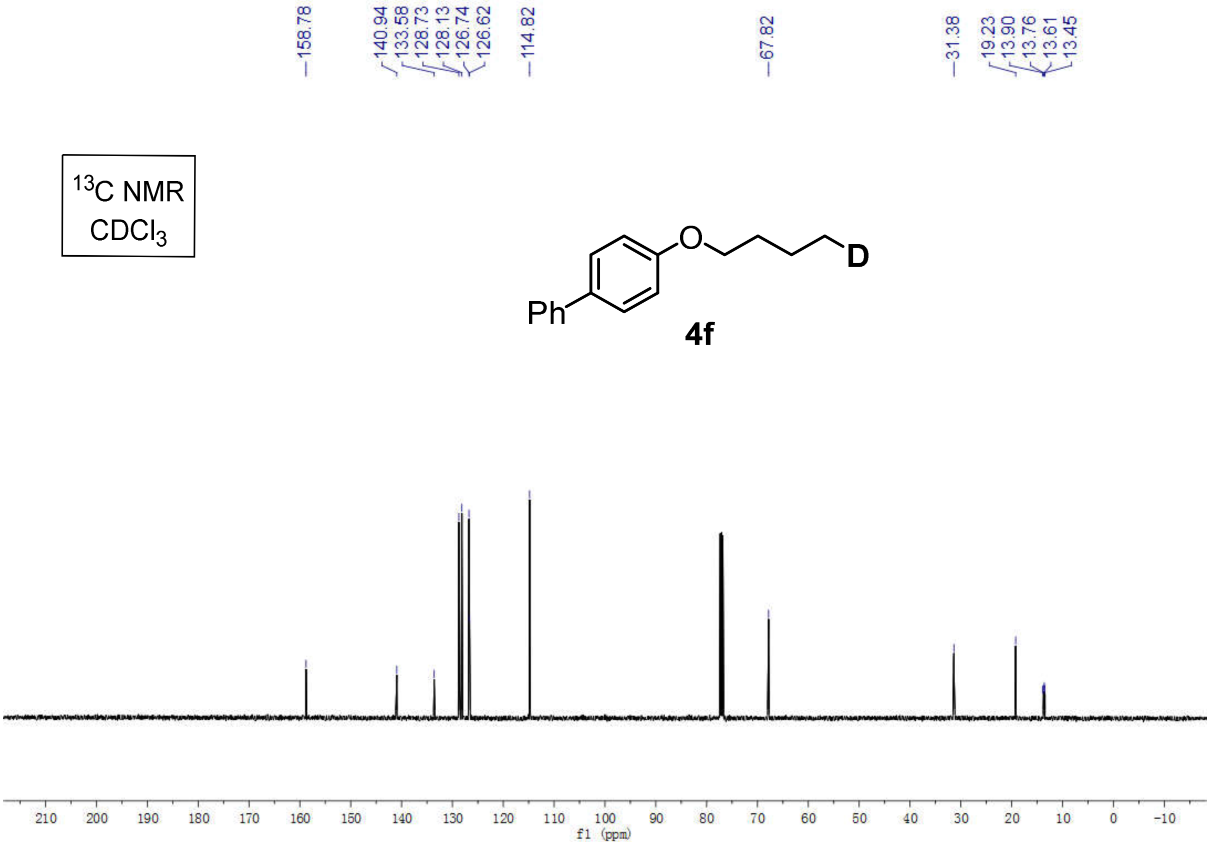


**Supplementary Fig. 75** $^{1}$H NMR, $^{2}$H NMR and $^{13}$C NMR spectra of the compound **4f**.

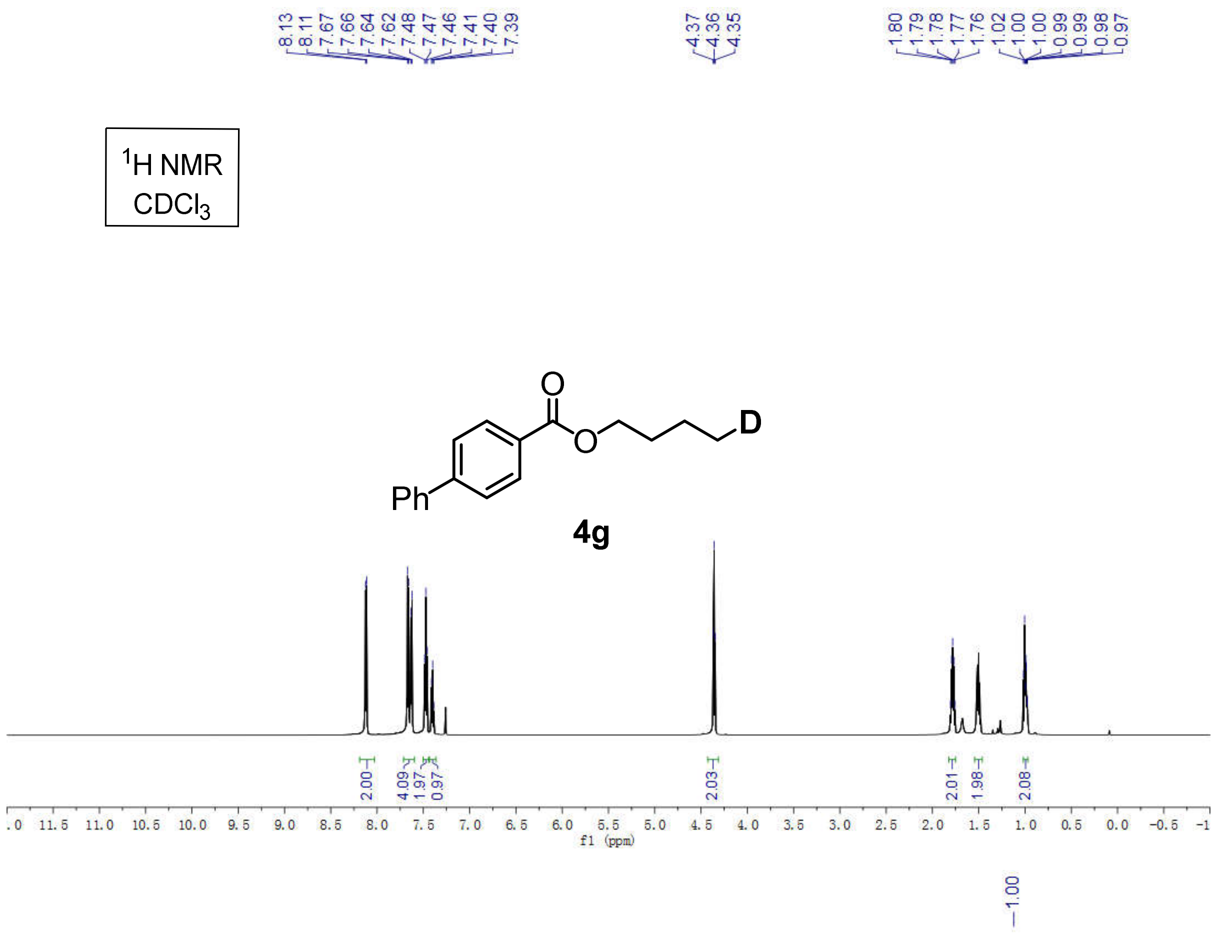
8.13
8.11
7.67
7.66
7.64
7.62
7.48
7.47
7.46
7.41
7.40
7.39
4.37
4.36
4.35
1.80
1.79
1.78
1.77
1.76
1.02
1.00
0.99
0.99
0.98
0.97
$^1$H NMR
$CDCl_3$
O
O
D
Ph
4g
2.00
4.09
1.97
0.97
2.03
2.01
1.98
2.08
f1 (ppm)

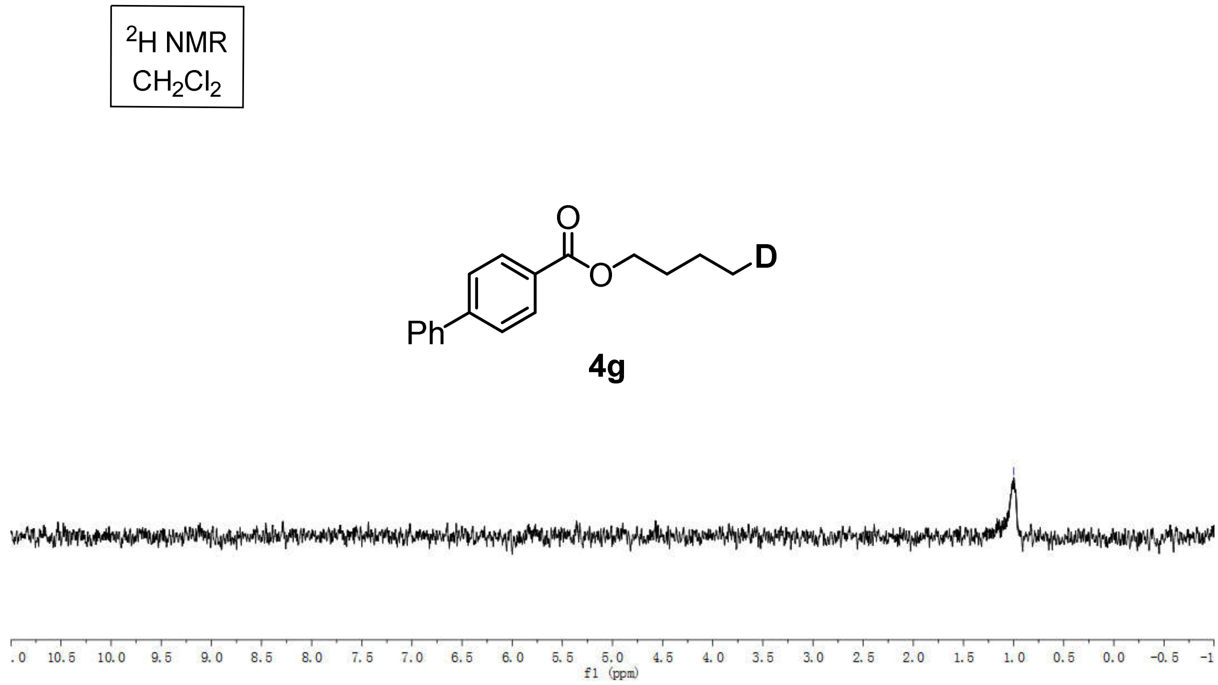
1.00
$^2$H NMR
$CH_2Cl_2$
O
O
D
Ph
4g
f1 (ppm)

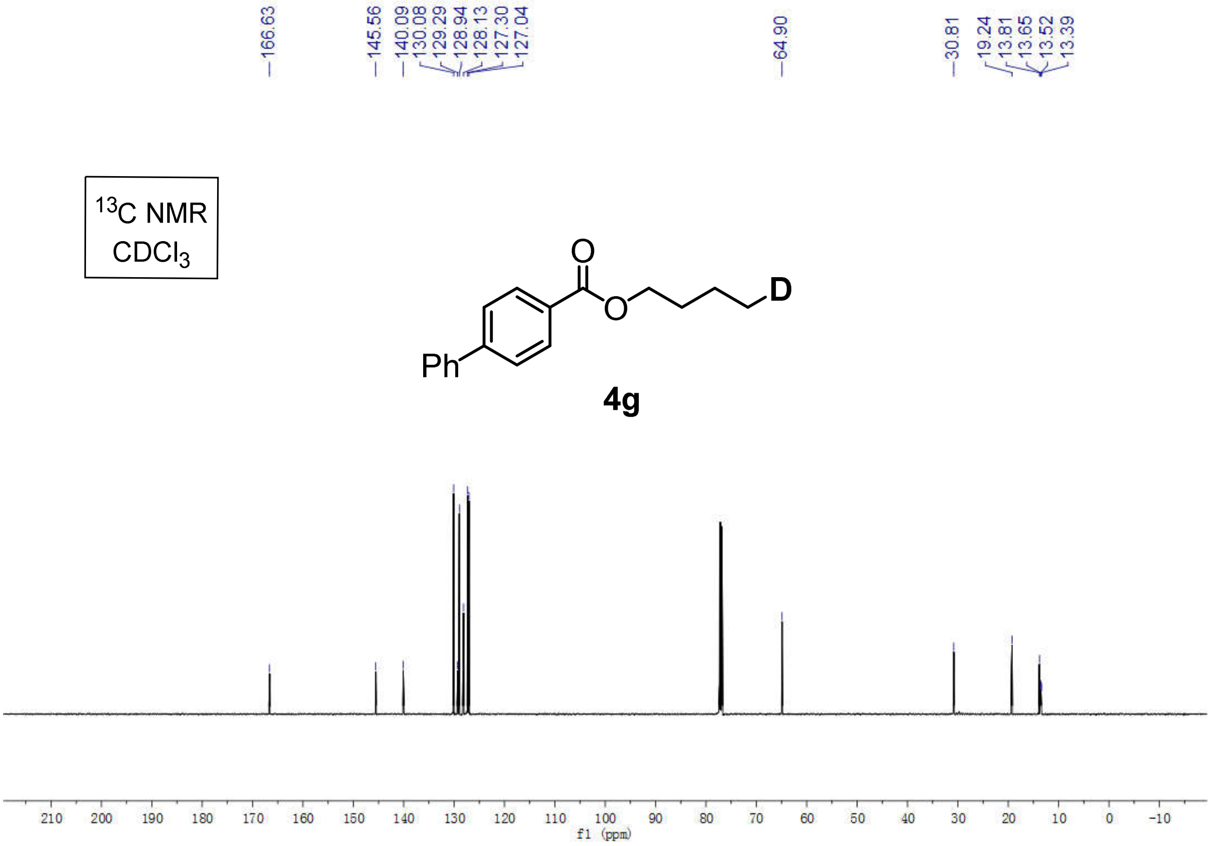


**Supplementary Fig. 76** $^{1}$H NMR, $^{2}$H NMR and $^{13}$C NMR spectra of the compound **4g**.

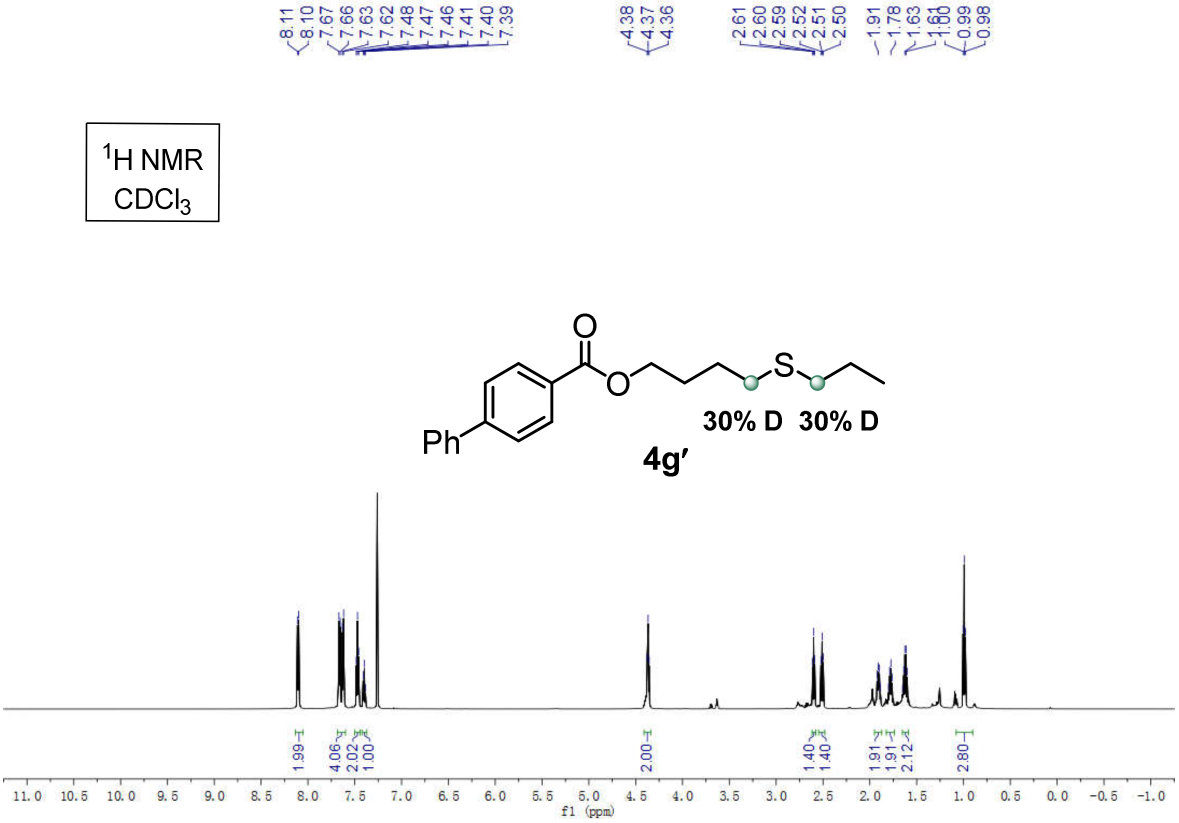


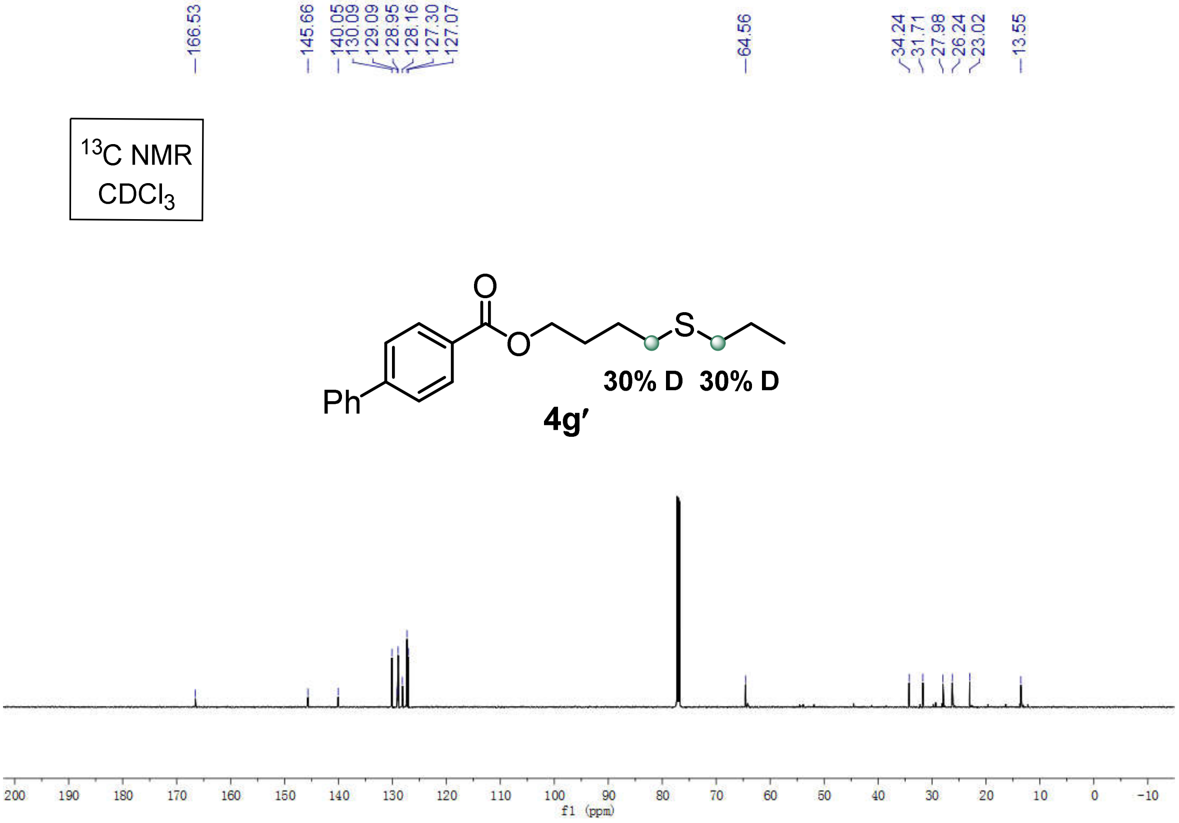


**Supplementary Fig. 77** $^1$H NMR, $^2$H NMR and $^{13}$C NMR spectra of the compound **4g'**.

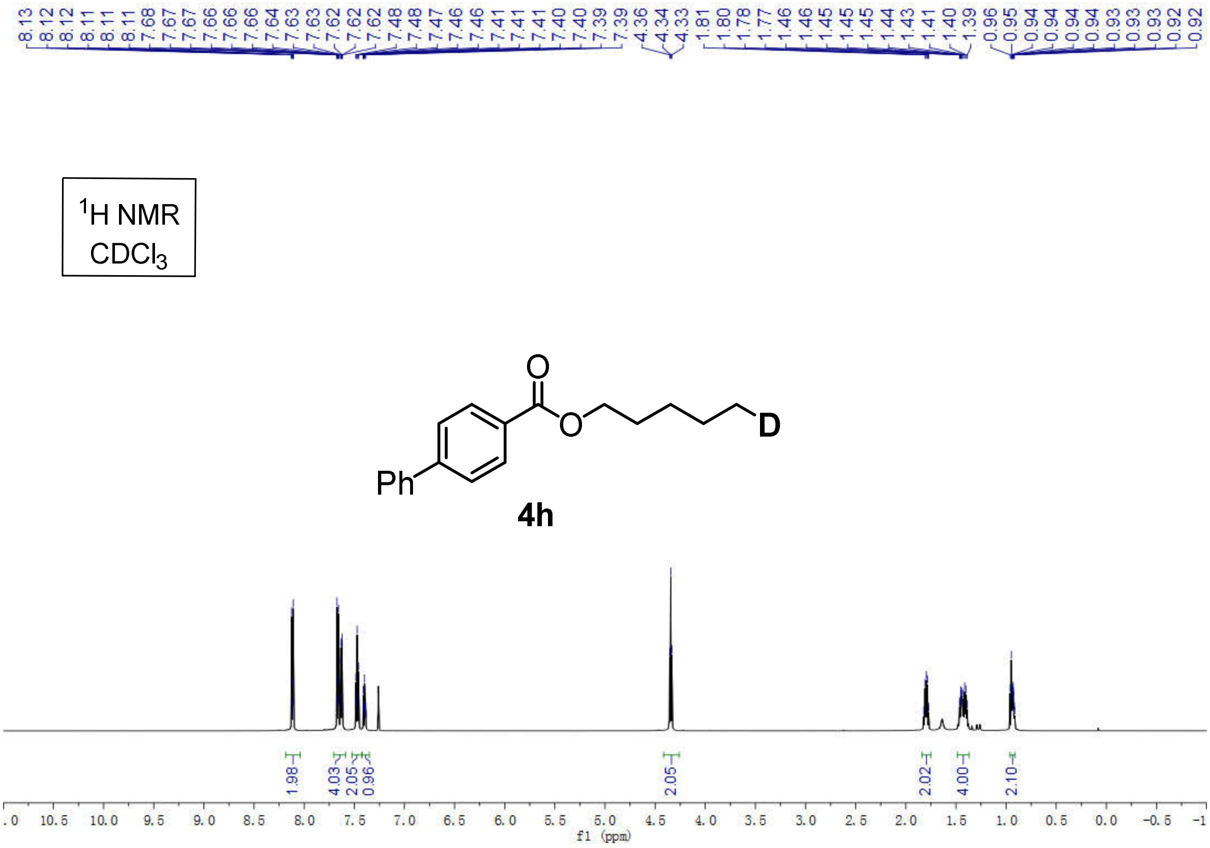
$^1$H NMR
$CDCl_3$
Ph
O
O
D
4h
1.98
4.03
2.05
0.96
2.05
2.02
4.00
2.10
f1 (ppm)

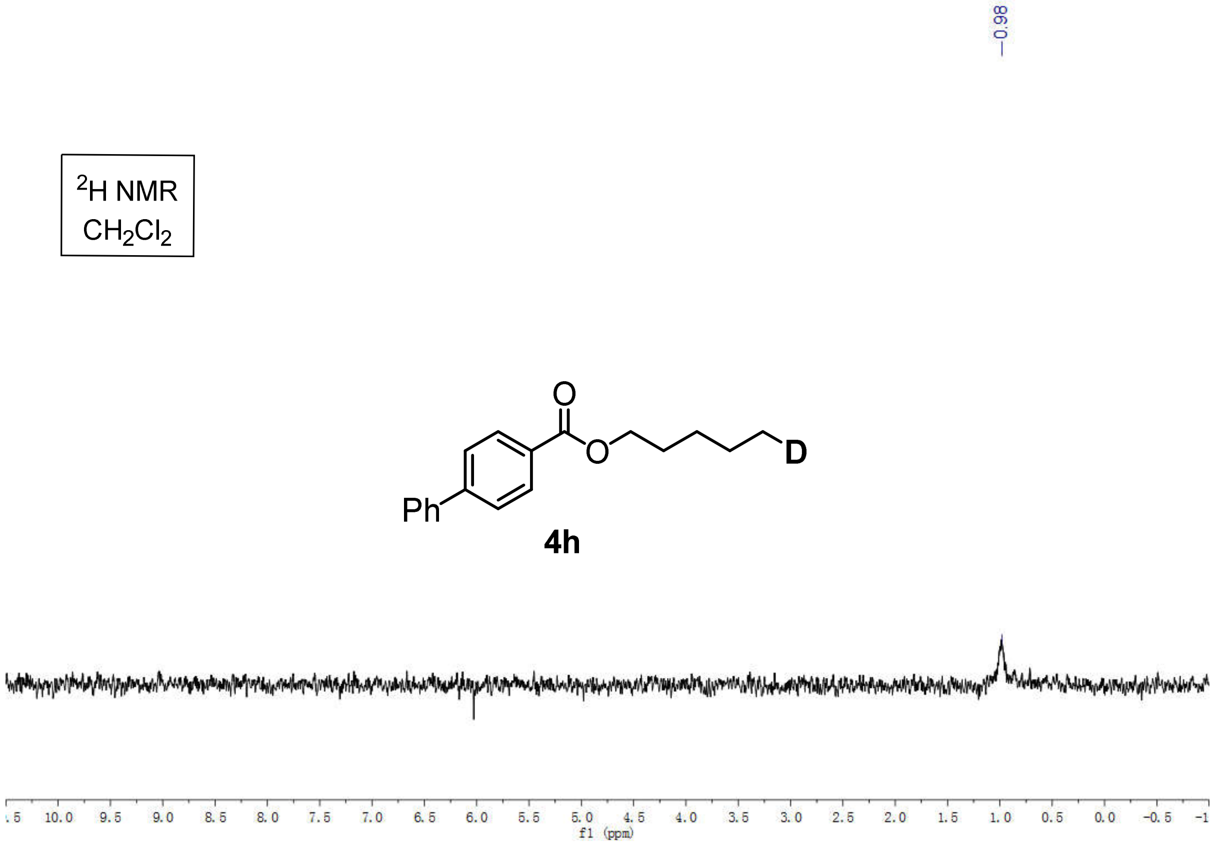
-0.98
$^2$H NMR
$CH_2Cl_2$
Ph
O
O
D
4h
f1 (ppm)

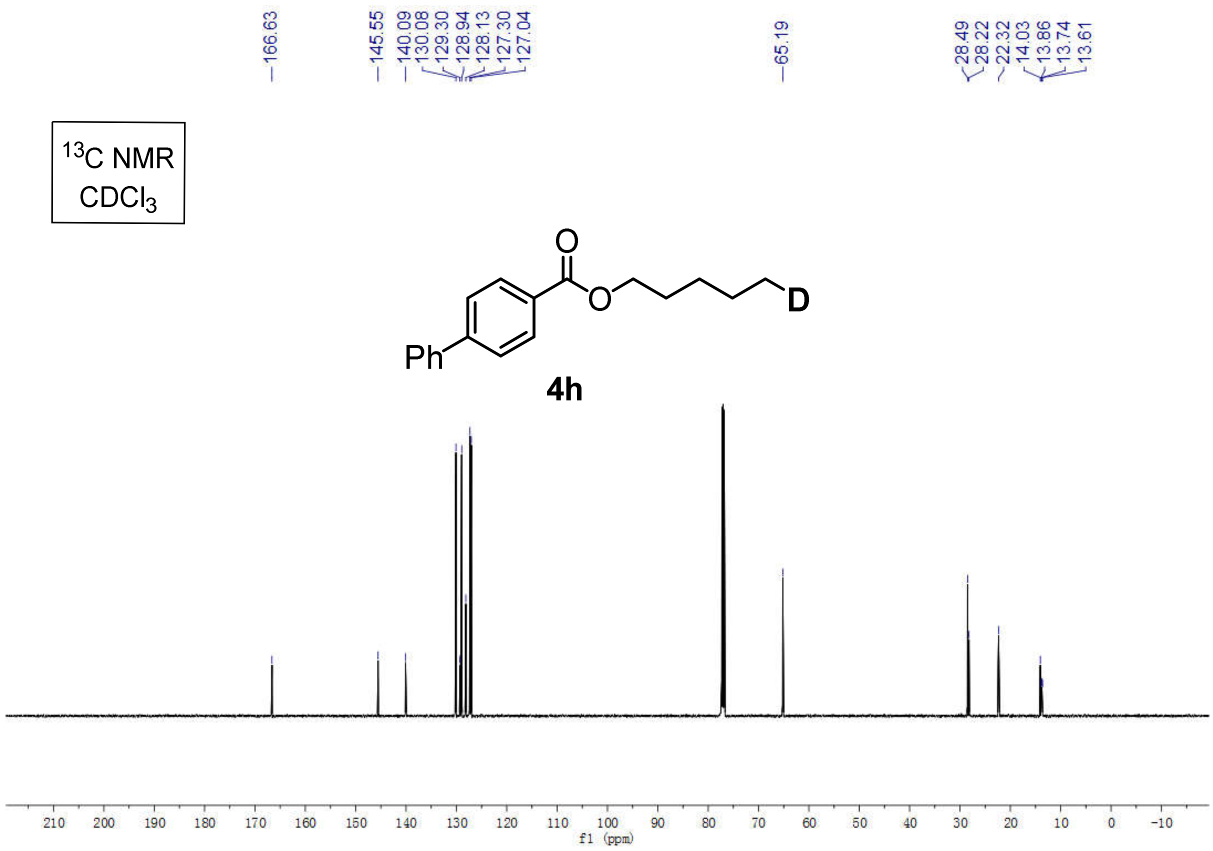


**Supplementary Fig. 78** $^{1}$H NMR, $^{2}$H NMR and $^{13}$C NMR spectra of the compound **4h**.

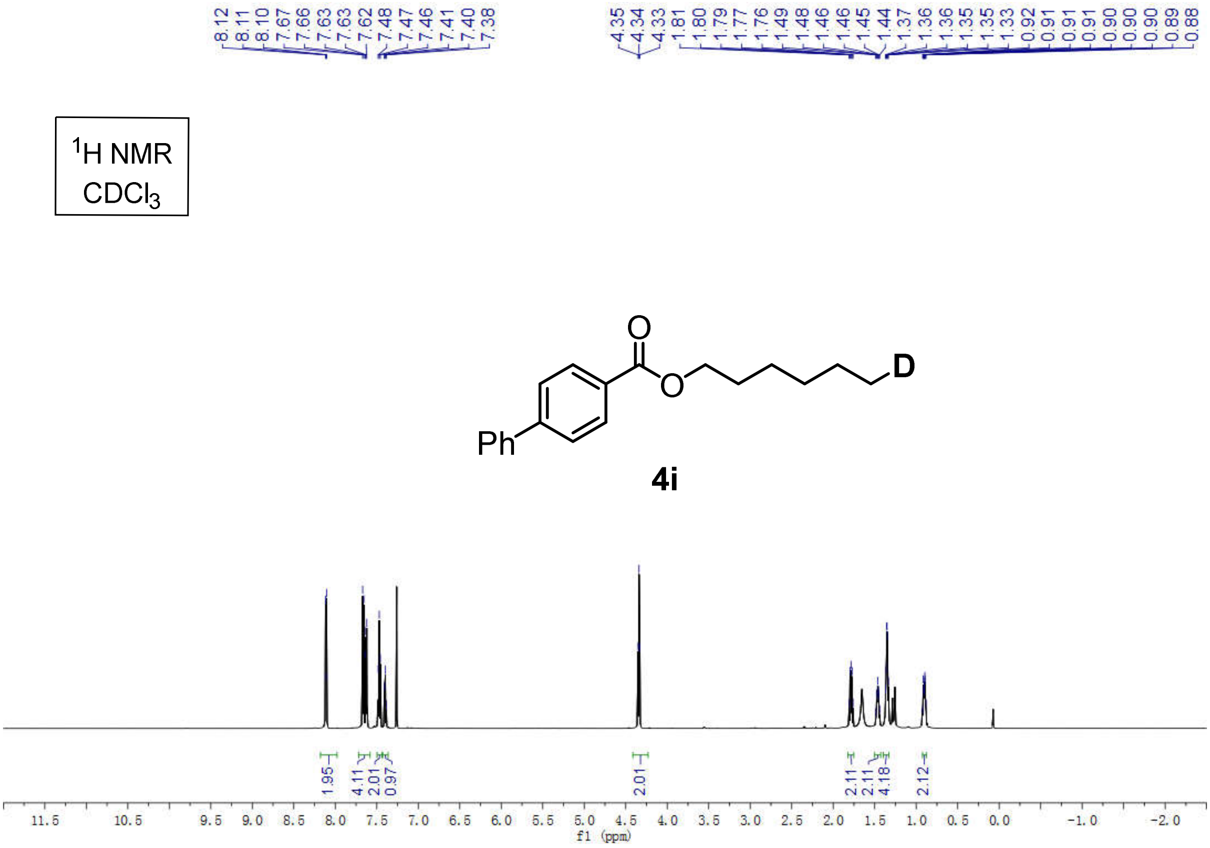

8.12
8.11
8.10
7.67
7.66
7.63
7.63
7.62
7.48
7.47
7.46
7.41
7.40
7.38
4.35
4.34
4.33
1.81
1.80
1.79
1.77
1.76
1.49
1.48
1.46
1.46
1.45
1.44
1.37
1.36
1.35
1.35
1.33
0.92
0.91
0.91
0.90
0.90
0.90
0.89
0.88
$^1$H NMR
$CDCl_3$
O
O
D
Ph
4i
1.95
4.11
2.01
0.97
2.01
2.11
2.11
4.18
2.12
f1 (ppm)


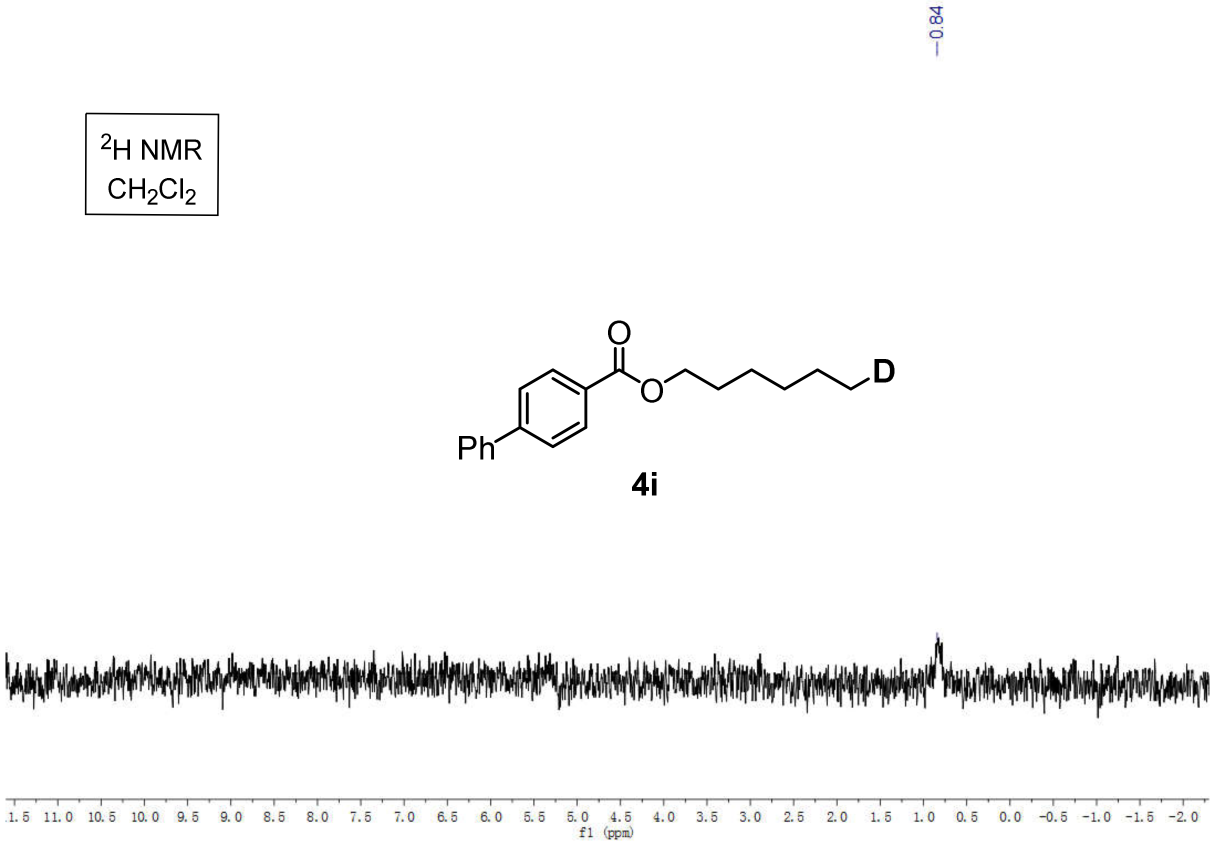

0.84
$^2$H NMR
$CH_2Cl_2$
O
O
D
Ph
4i
f1 (ppm)

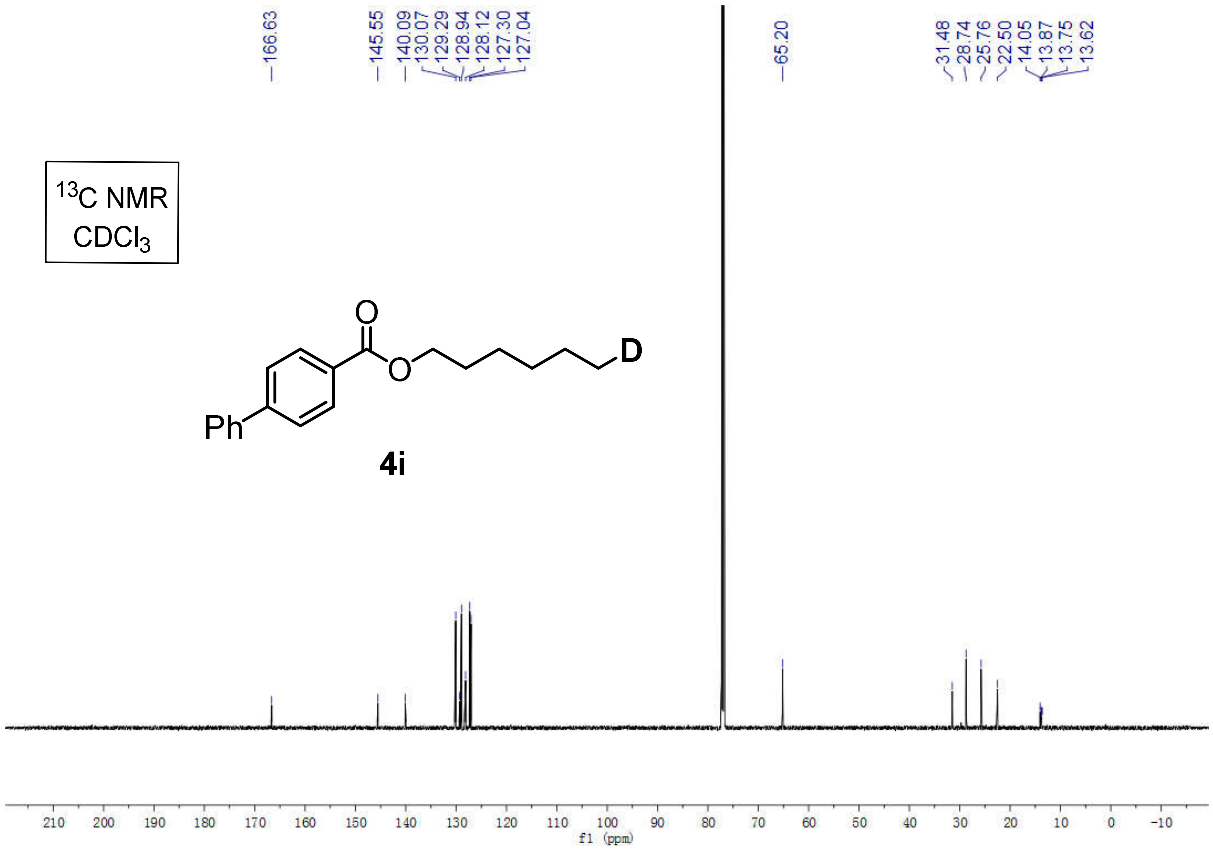


**Supplementary Fig. 79** $^{1}$H NMR, $^{2}$H NMR and $^{13}$C NMR spectra of the compound **4i**.

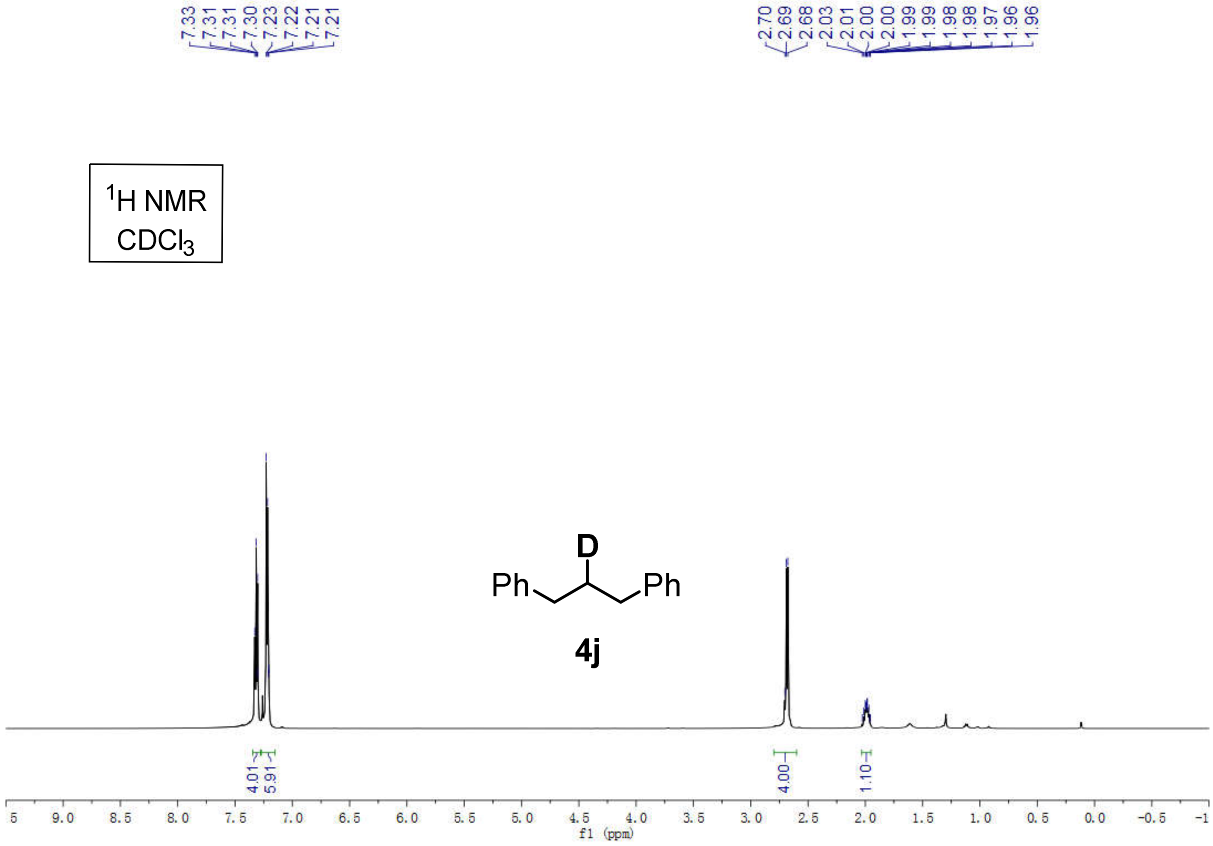
7.33 7.31 7.31 7.30 7.23 7.22 7.21 7.21
2.70 2.69 2.68 2.03 2.01 2.00 2.00 1.99 1.99 1.98 1.98 1.97 1.96 1.96
$^{1}$H NMR
$CDCl_3$
D
Ph Ph
4j
4.01 5.91 4.00 1.10
f1 (ppm)

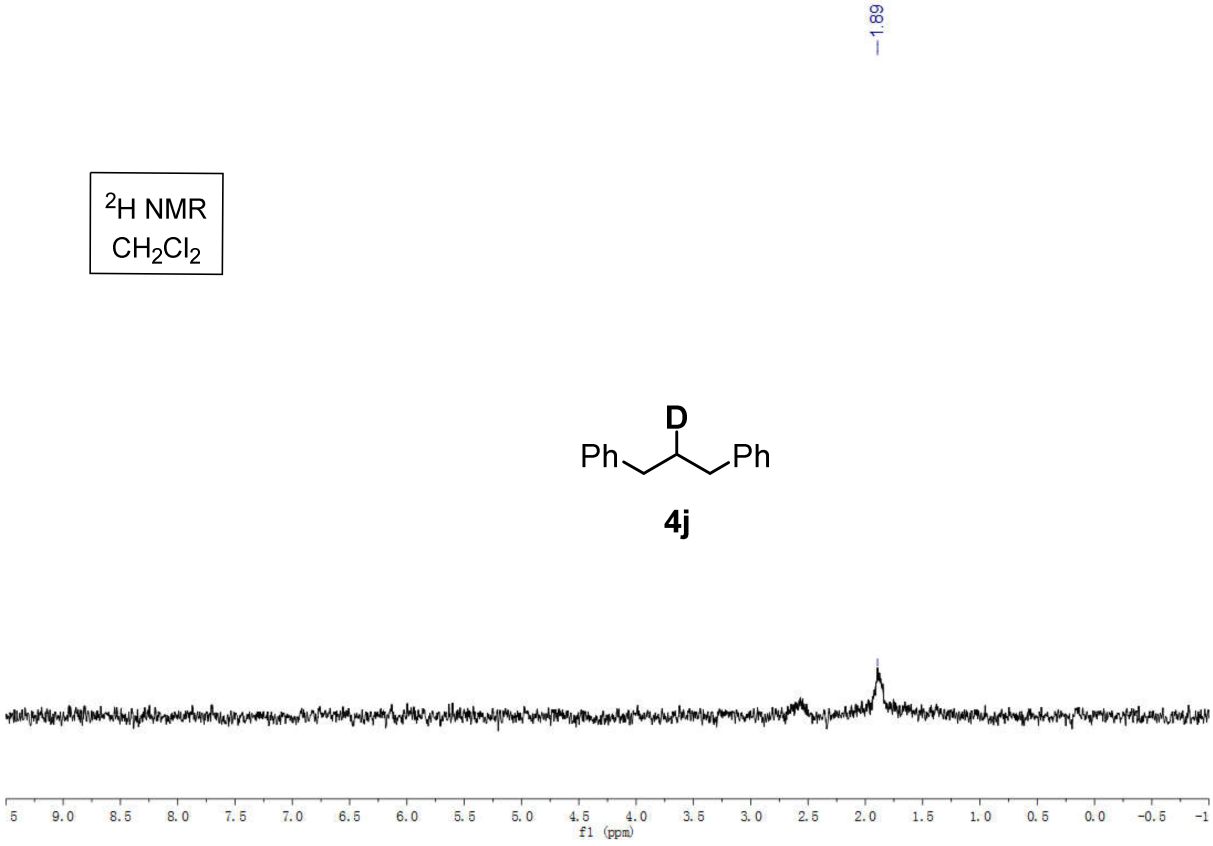
1.89
$^{2}$H NMR
$CH_2Cl_2$
D
Ph Ph
4j
f1 (ppm)

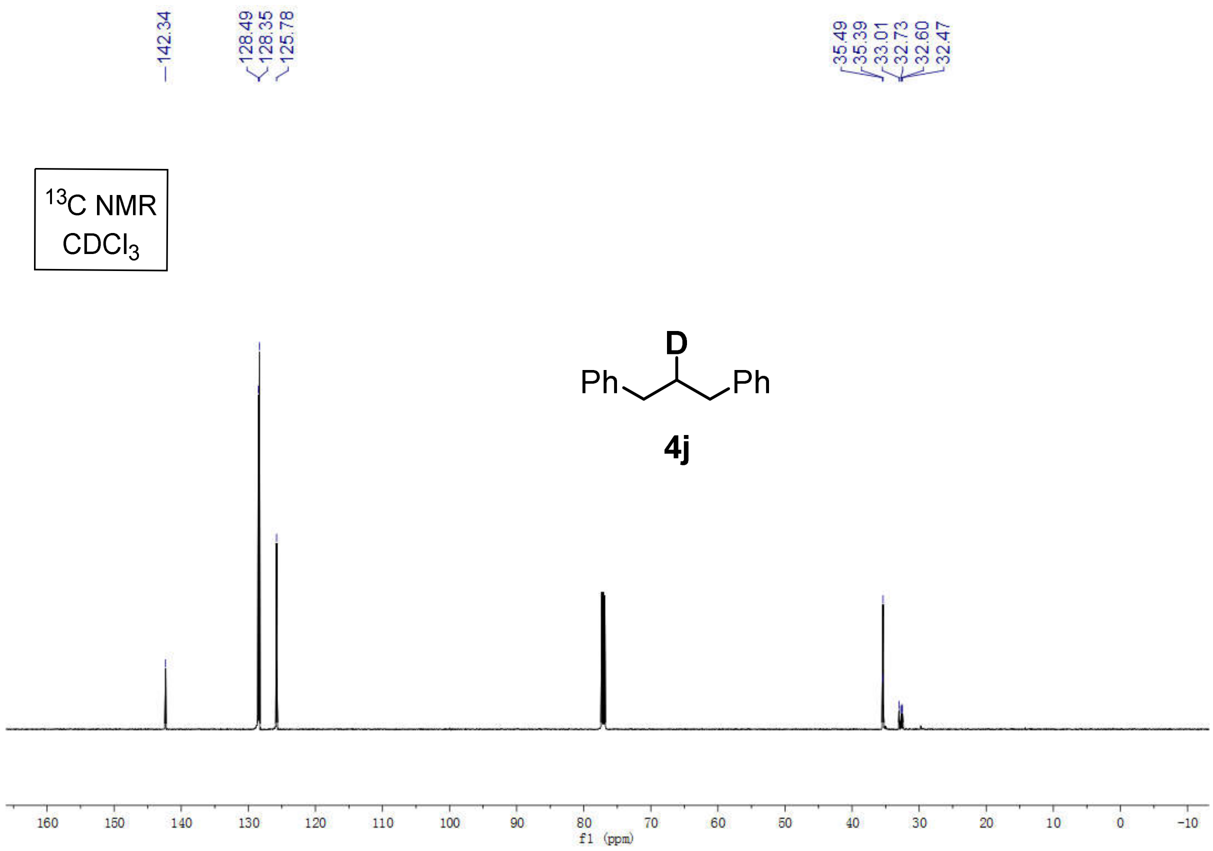


**Supplementary Fig. 80** $^{1}$H NMR, $^{2}$H NMR and $^{13}$C NMR spectra of the compound **4j**.

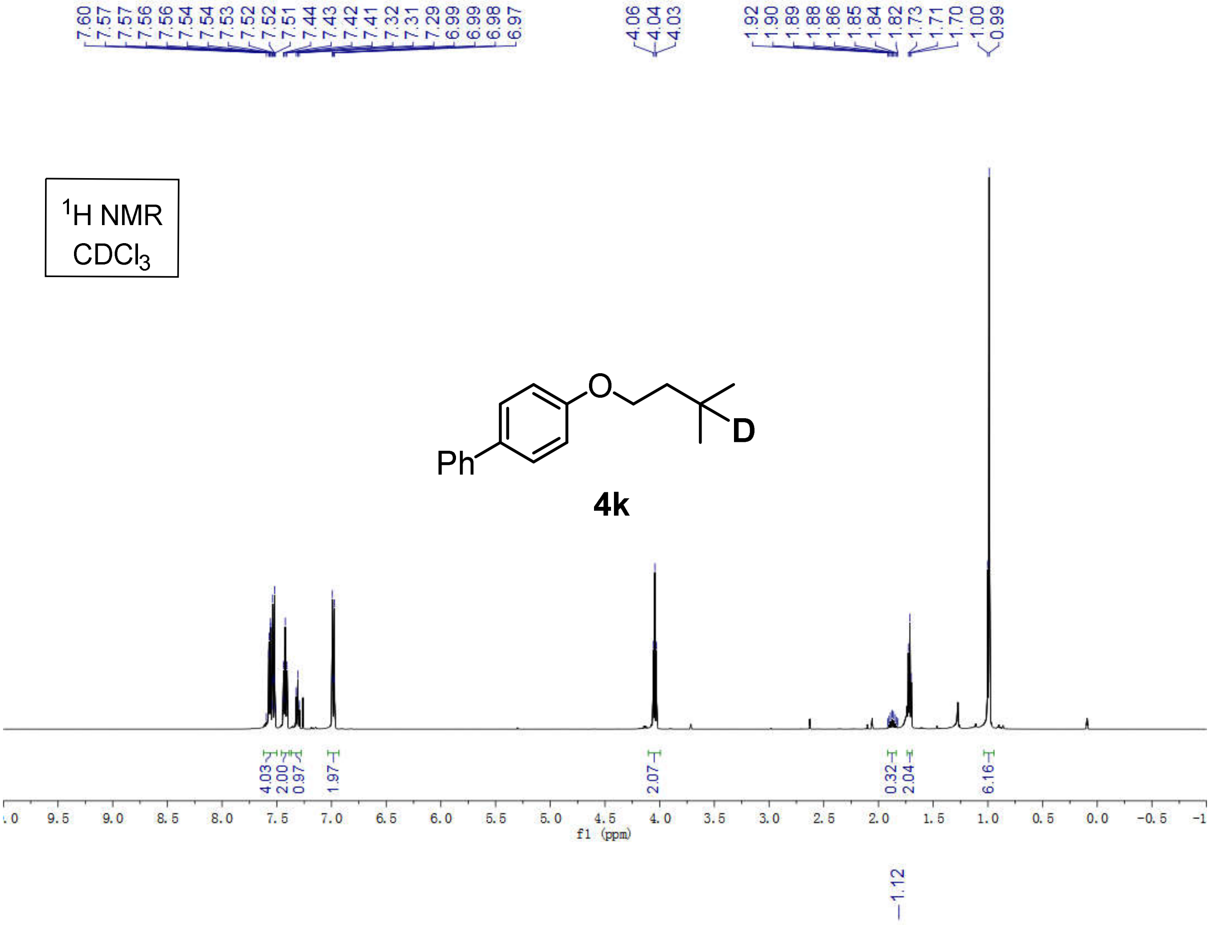
7.60 7.57 7.57 7.56 7.56 7.54 7.54 7.53 7.52 7.52 7.51 7.44 7.43 7.42 7.41 7.32 7.31 7.29 6.99 6.99 6.98 6.97
4.06 4.04 4.03
1.92 1.90 1.89 1.88 1.86 1.85 1.84 1.82 1.73 1.71 1.70 1.00 0.99
$^1$H NMR
$CDCl_3$
O
D
Ph
4k
4.03 2.00 0.97 1.97 2.07 0.32 2.04 6.16
f1 (ppm)

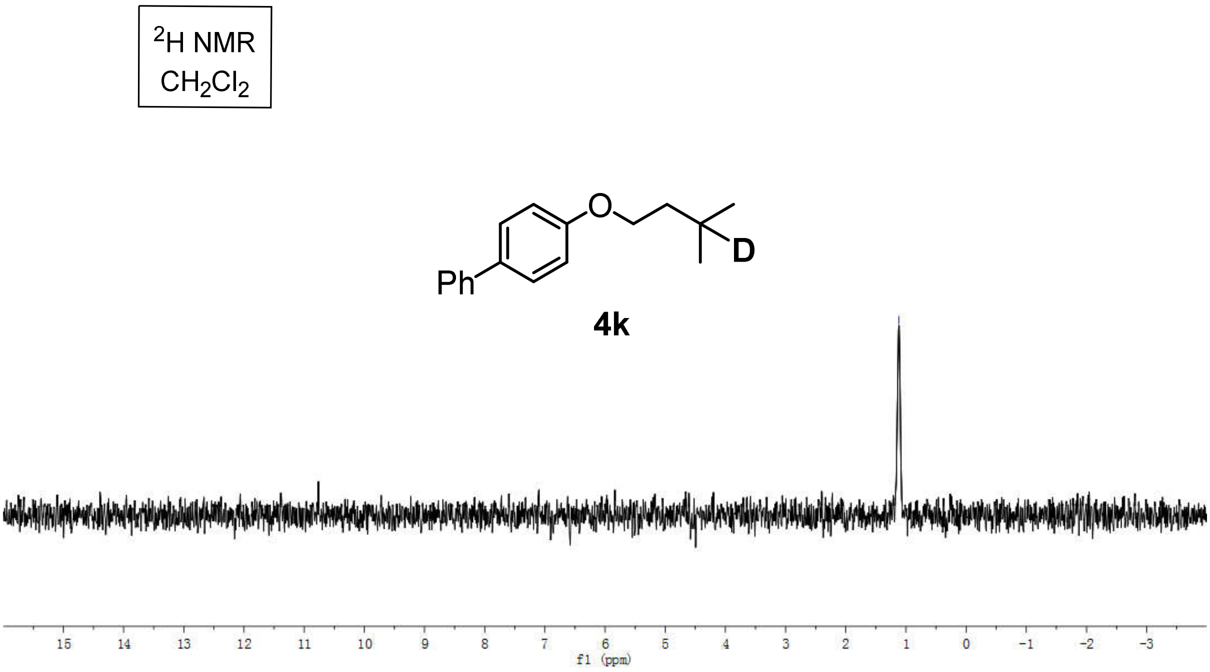
1.12
$^2$H NMR
$CH_2Cl_2$
O
D
Ph
4k
f1 (ppm)

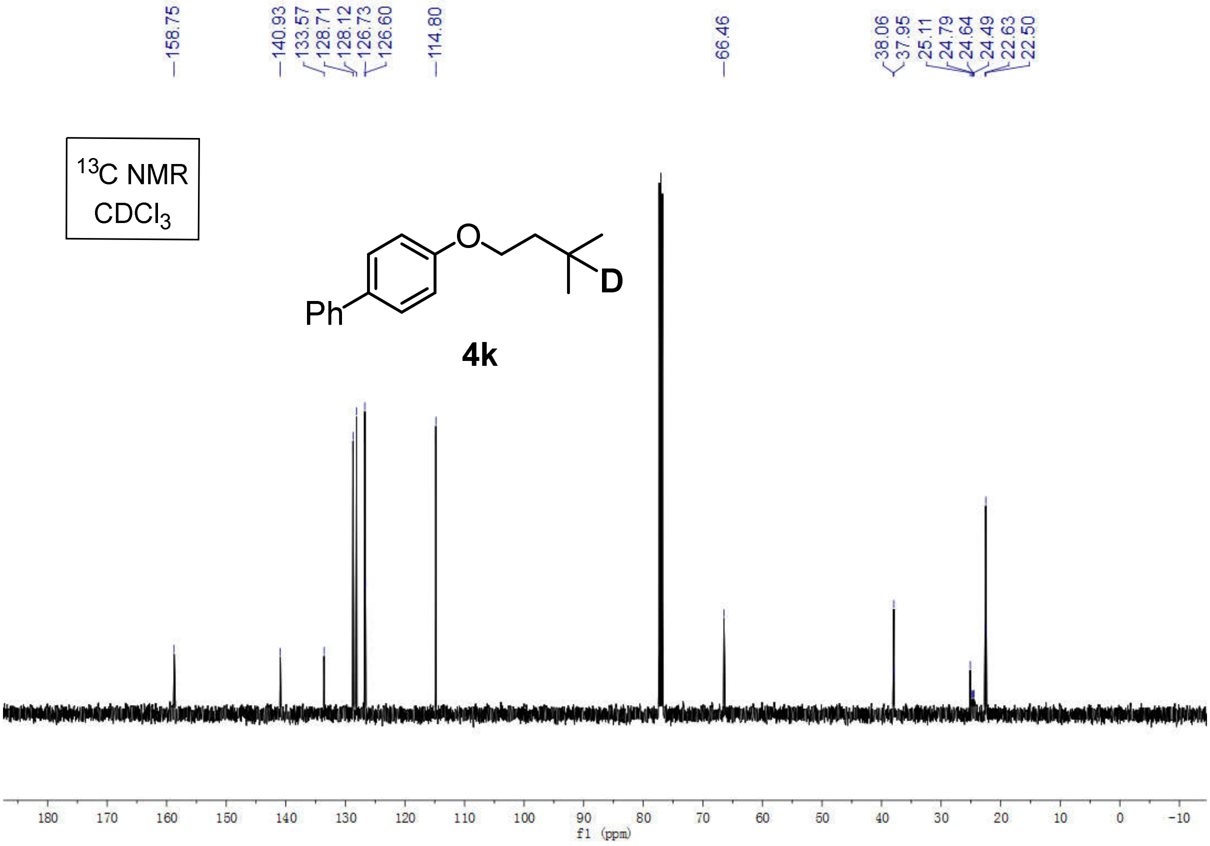


**Supplementary Fig. 81** $^{1}$H NMR, $^{2}$H NMR and $^{13}$C NMR spectra of the compound **4k**.

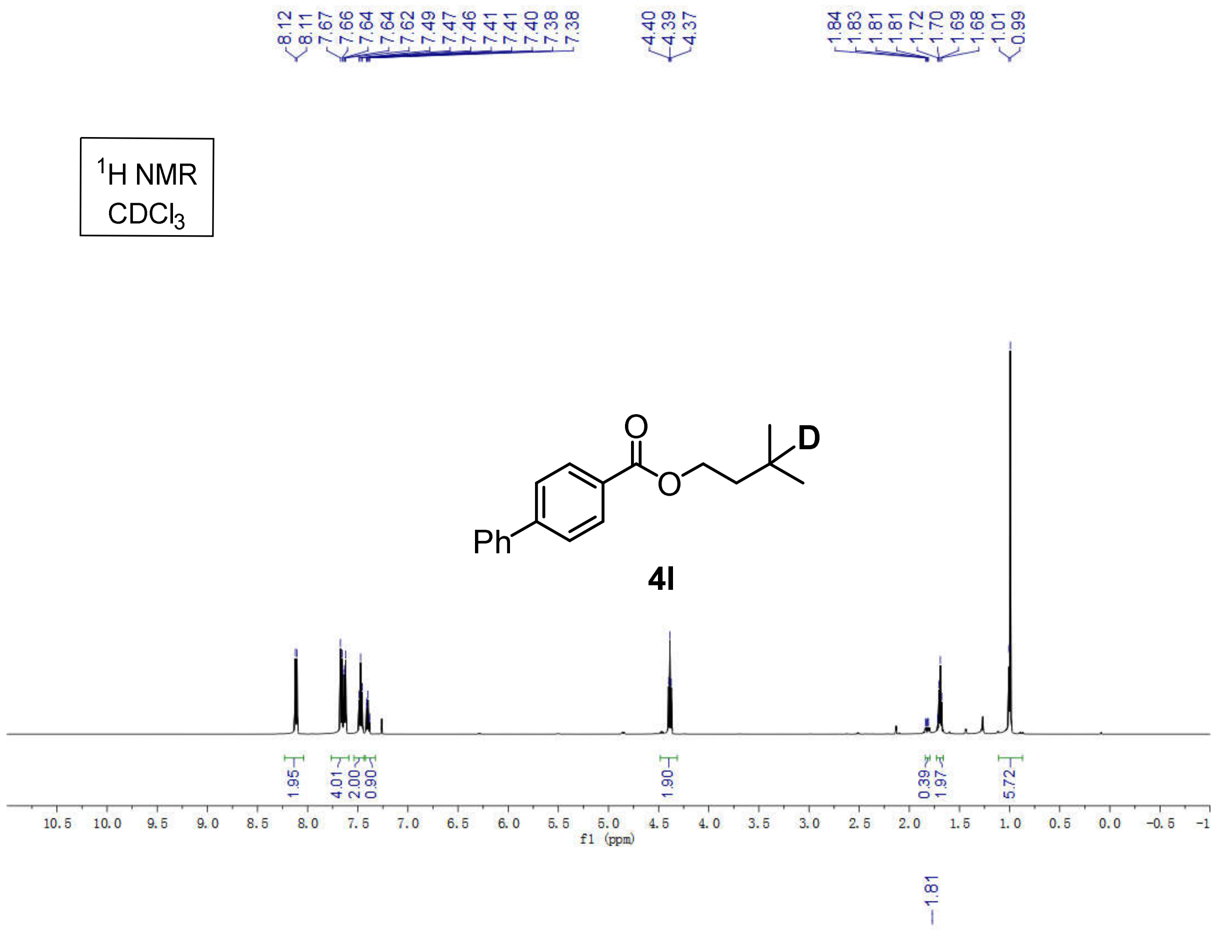
$^1H$ NMR
$CDCl_3$
Ph
O
O
D
4l
8.12
8.11
7.67
7.66
7.64
7.64
7.62
7.49
7.47
7.46
7.41
7.41
7.40
7.38
7.38
4.40
4.39
4.37
1.84
1.83
1.81
1.81
1.72
1.70
1.69
1.68
1.01
0.99
1.95
4.01
2.00
0.90
1.90
0.39
1.97
5.72
f1 (ppm)
1.81

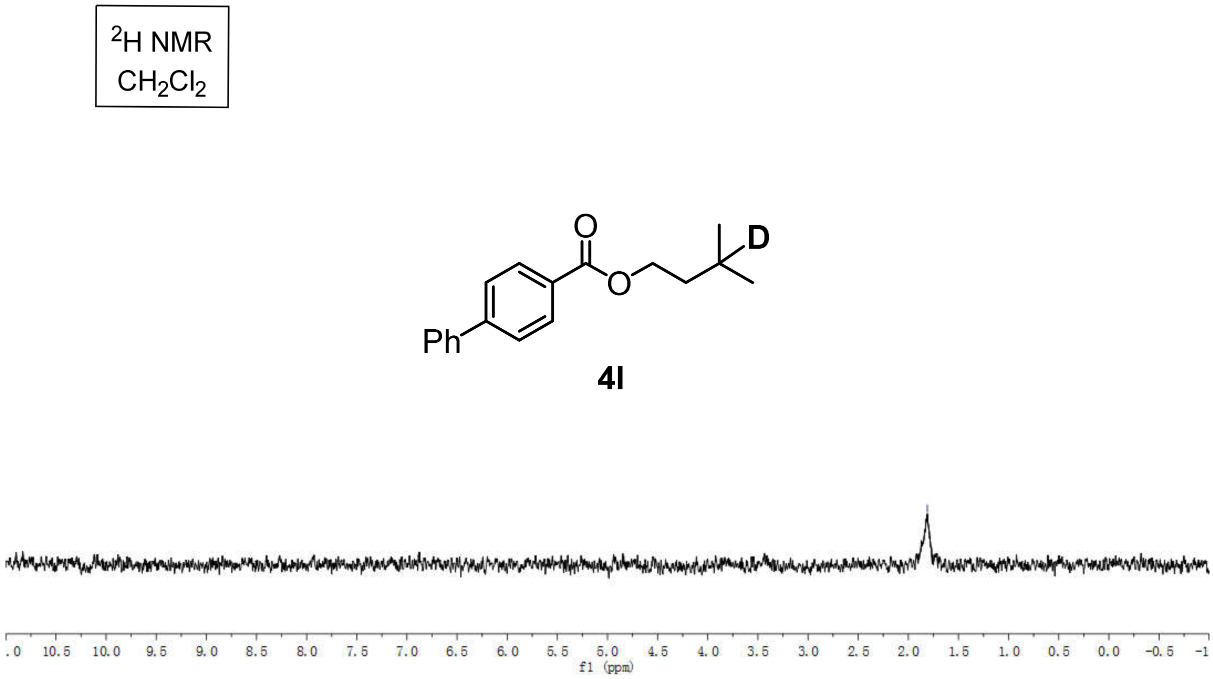
$^2H$ NMR
$CH_2Cl_2$
Ph
O
O
D
4l
f1 (ppm)

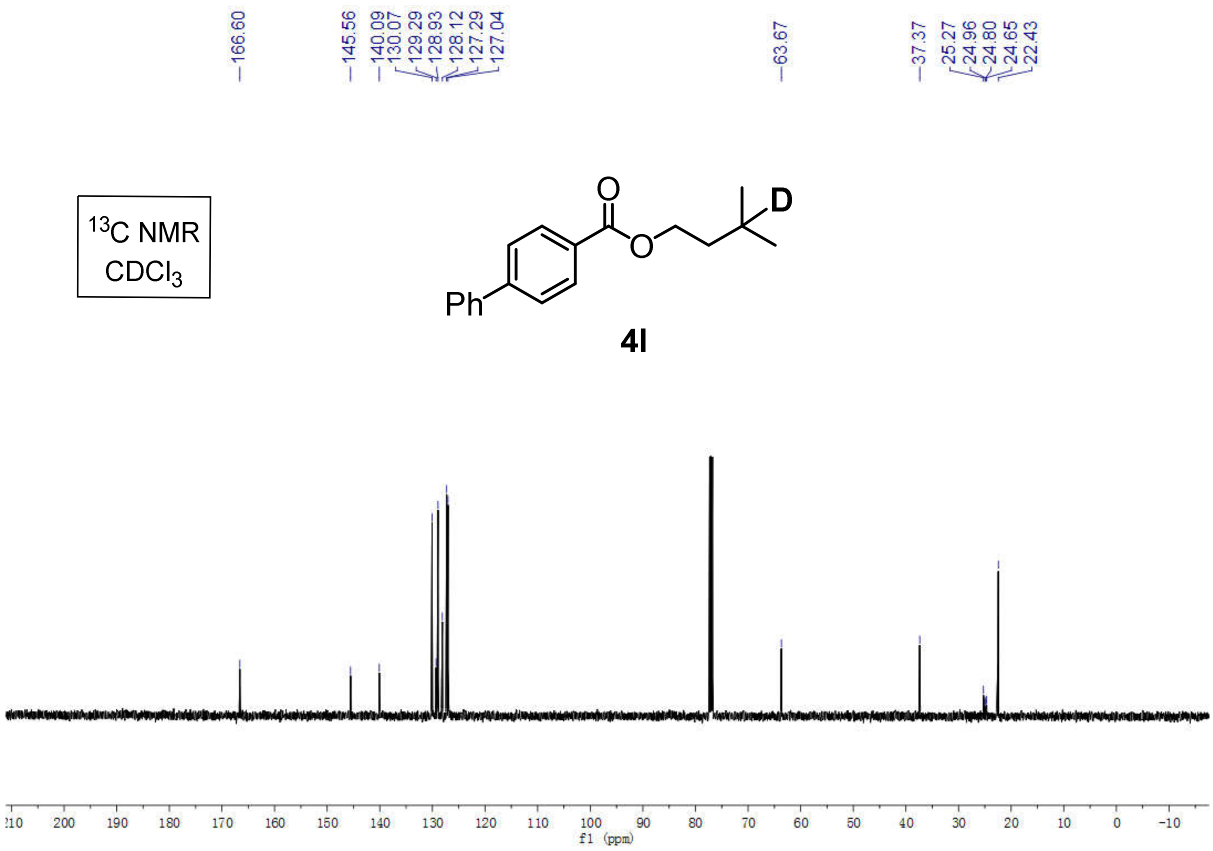


**Supplementary Fig. 82** $^{1}$H NMR, $^{2}$H NMR and $^{13}$C NMR spectra of the compound **4l**.

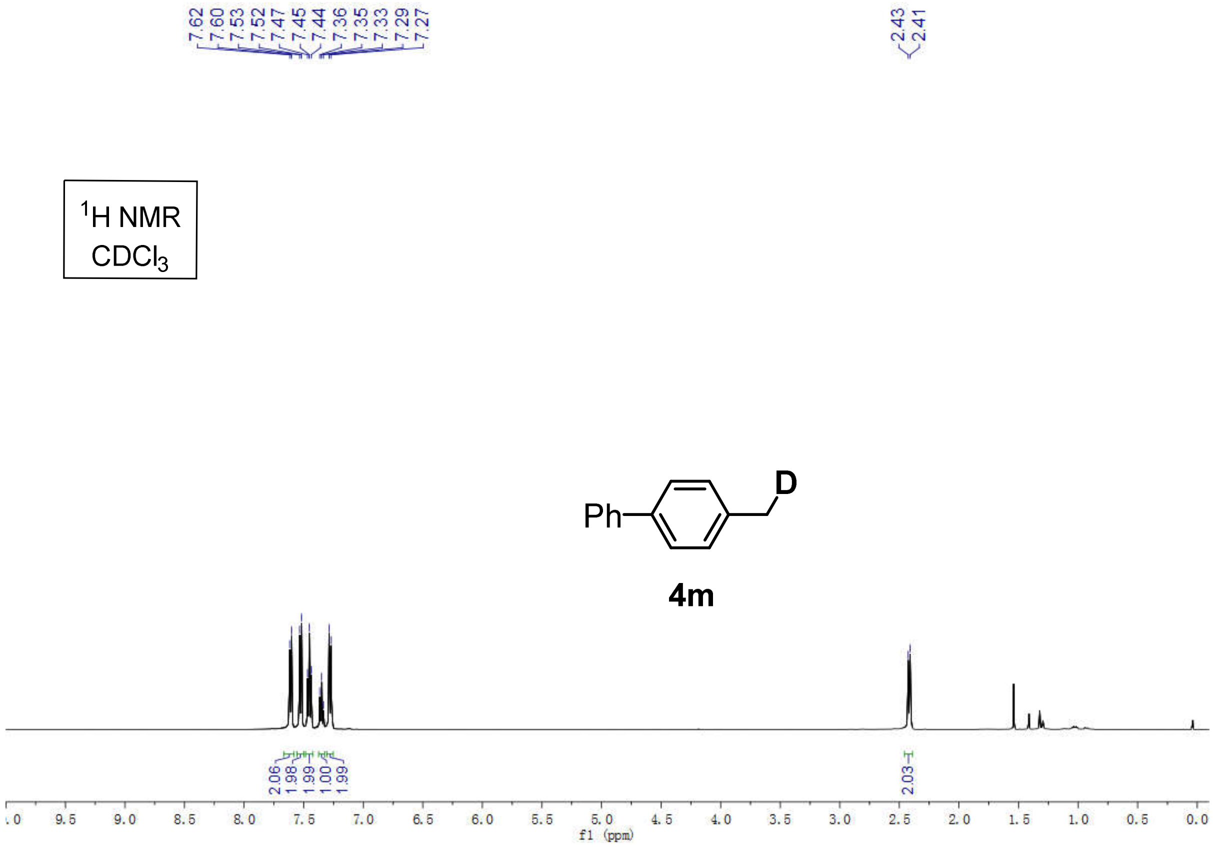
7.62
7.60
7.53
7.52
7.47
7.45
7.44
7.36
7.35
7.33
7.29
7.27
2.43
2.41
1H NMR
CDCl3
Ph
D
4m
2.06
1.98
1.99
1.00
1.99
2.03
f1 (ppm)

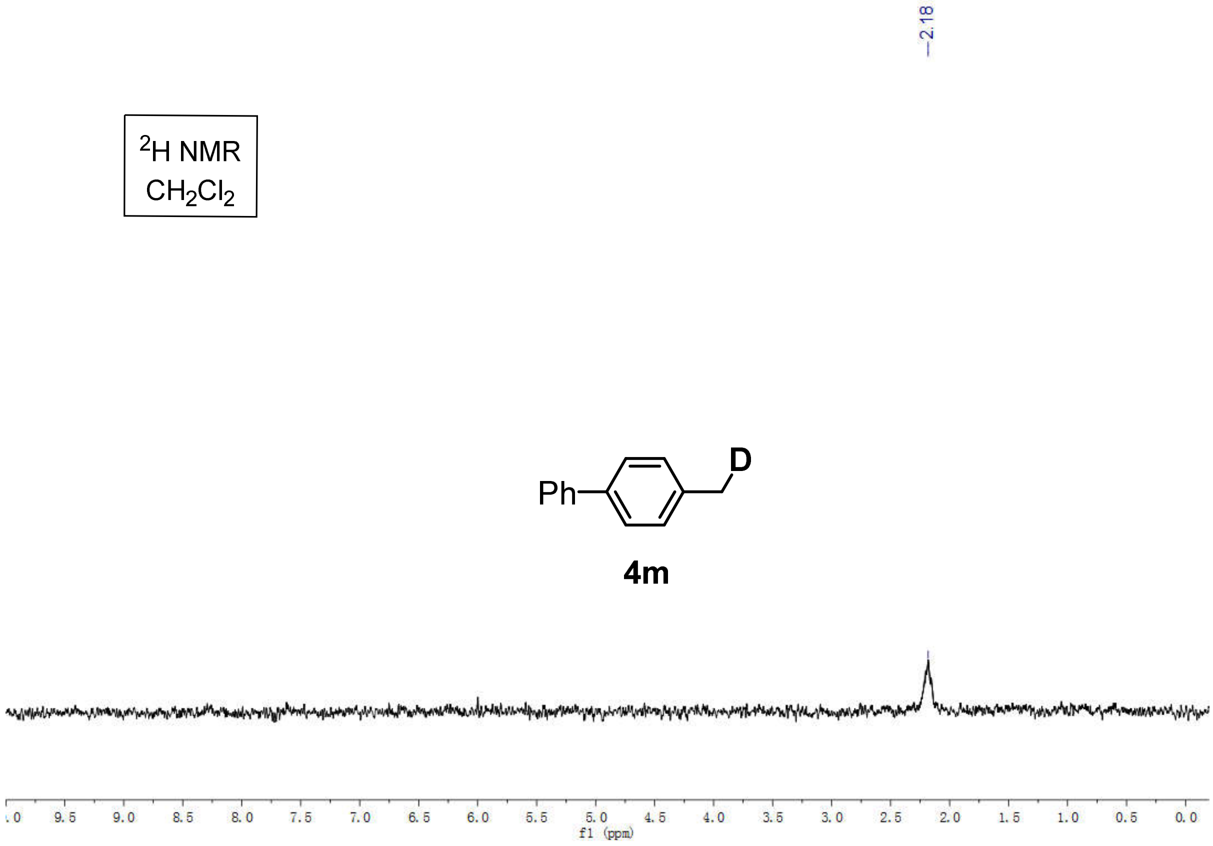
2.18
2H NMR
CH2Cl2
Ph
D
4m
f1 (ppm)

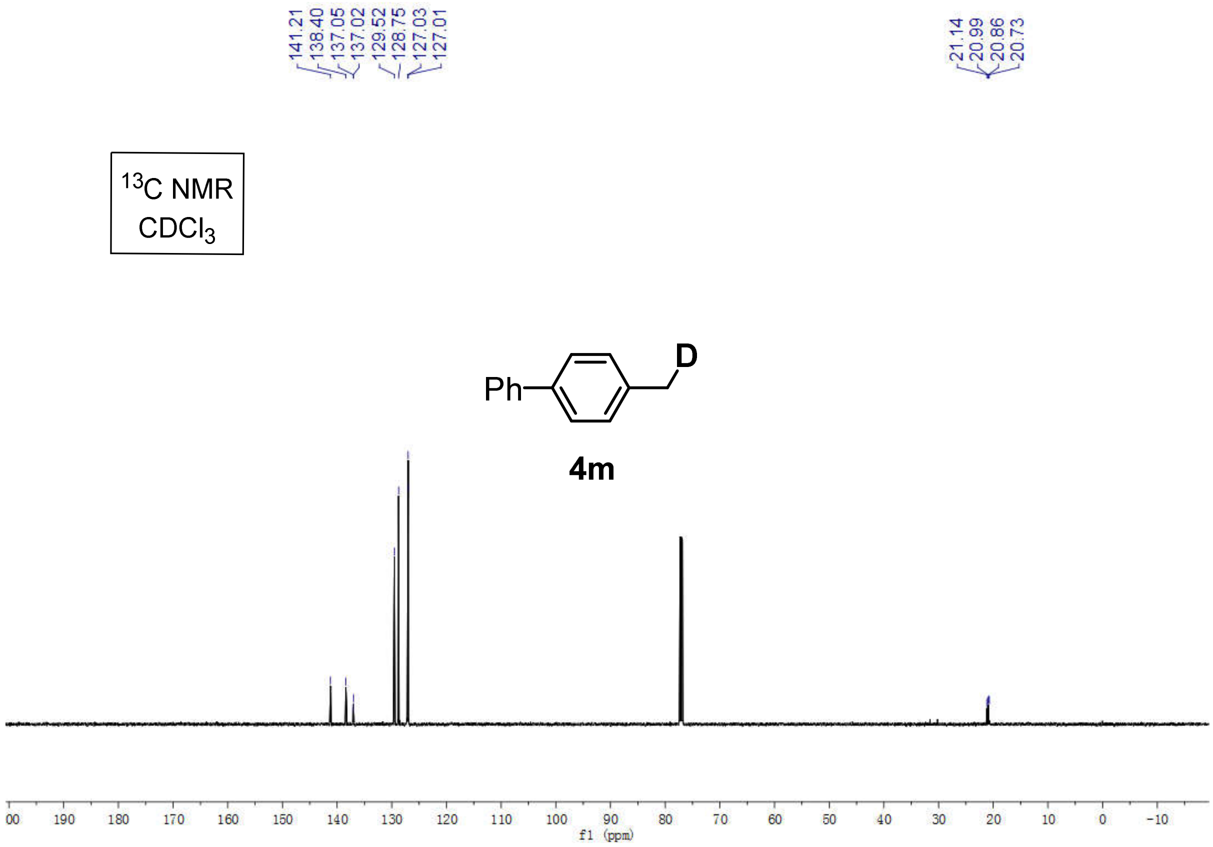


**Supplementary Fig. 83** $^{1}$H NMR, $^{2}$H NMR and $^{13}$C NMR spectra of the compound **4m**.

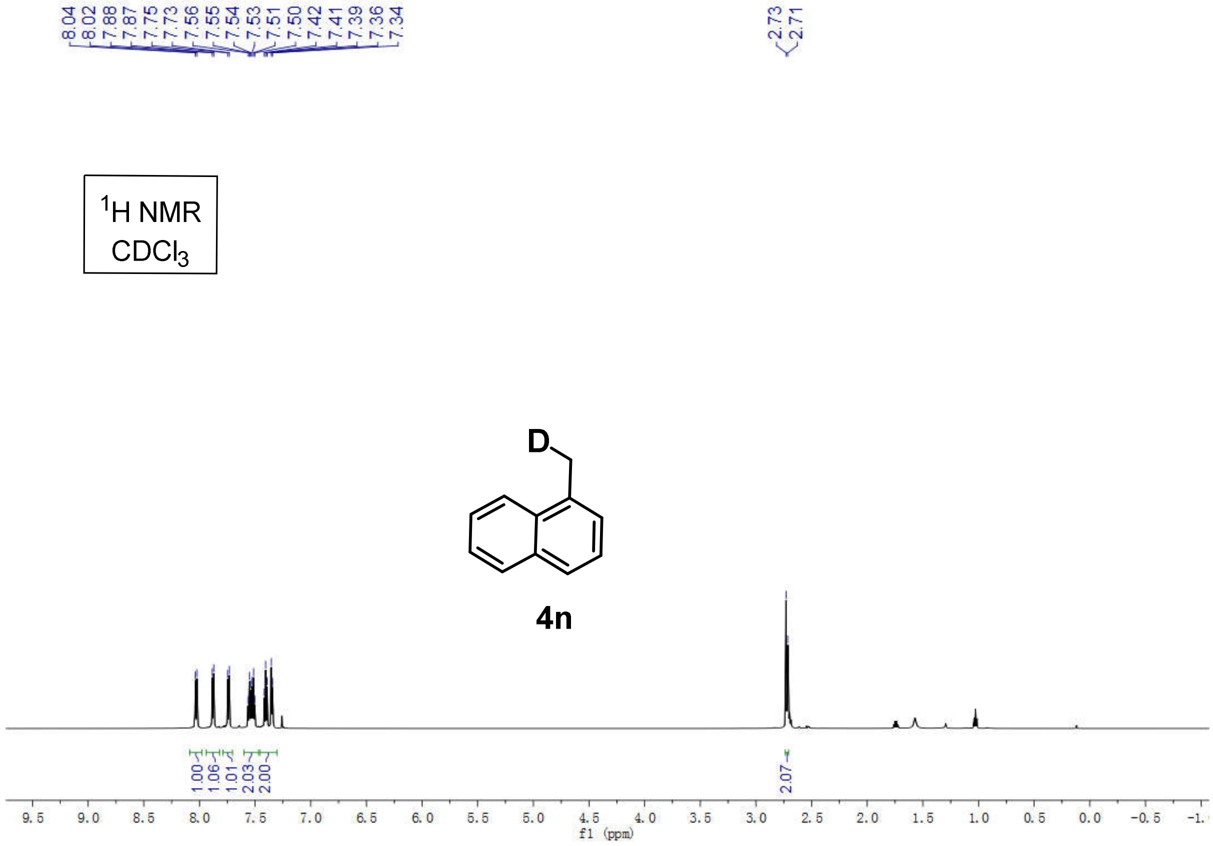
$^1$H NMR
CDCl$_3$
4n
8.04 8.02 7.88 7.87 7.75 7.73 7.56 7.55 7.54 7.53 7.51 7.50 7.42 7.41 7.39 7.36 7.34
2.73 2.71
1.00 1.06 1.01 2.03 2.00
2.07
f1 (ppm)

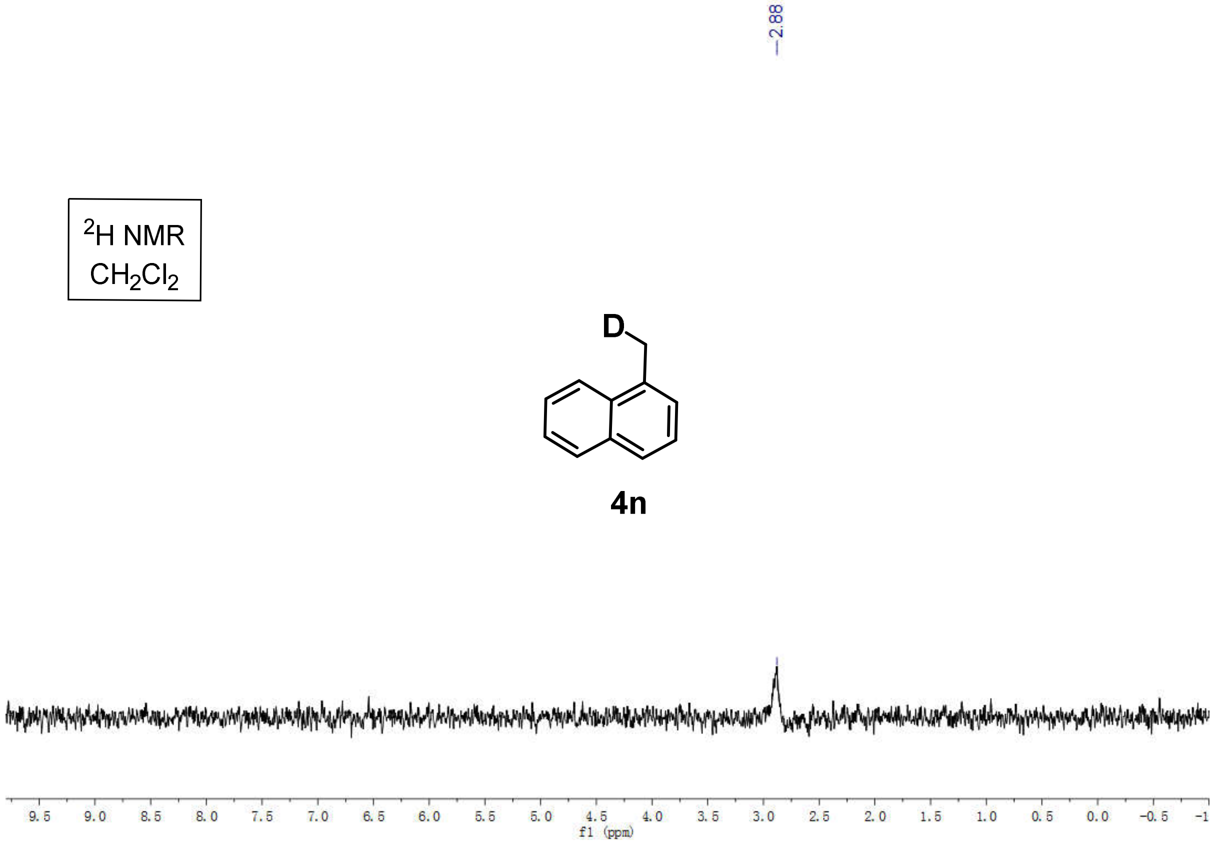
$^2$H NMR
CH$_2$Cl$_2$
4n
2.88
f1 (ppm)

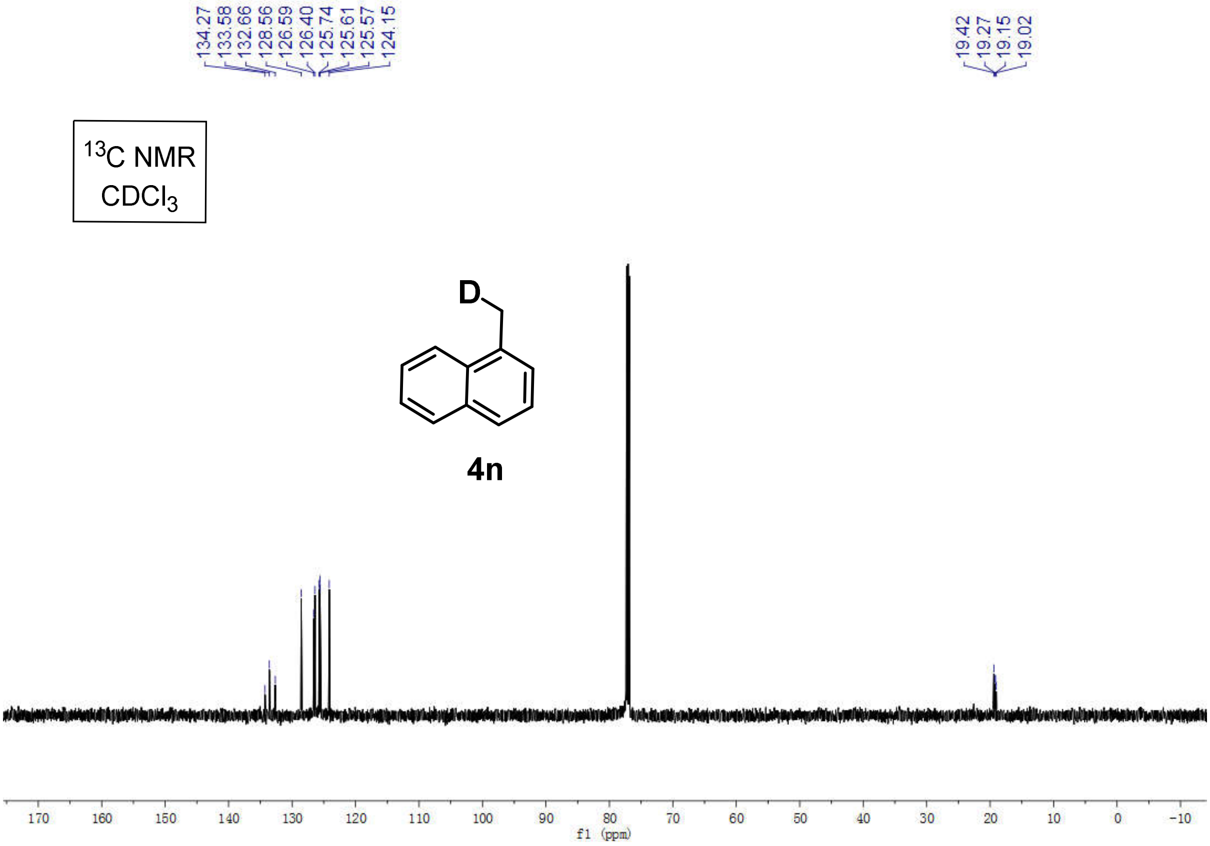


**Supplementary Fig. 84** $^{1}$H NMR, $^{2}$H NMR and $^{13}$C NMR spectra of the compound **4n**.

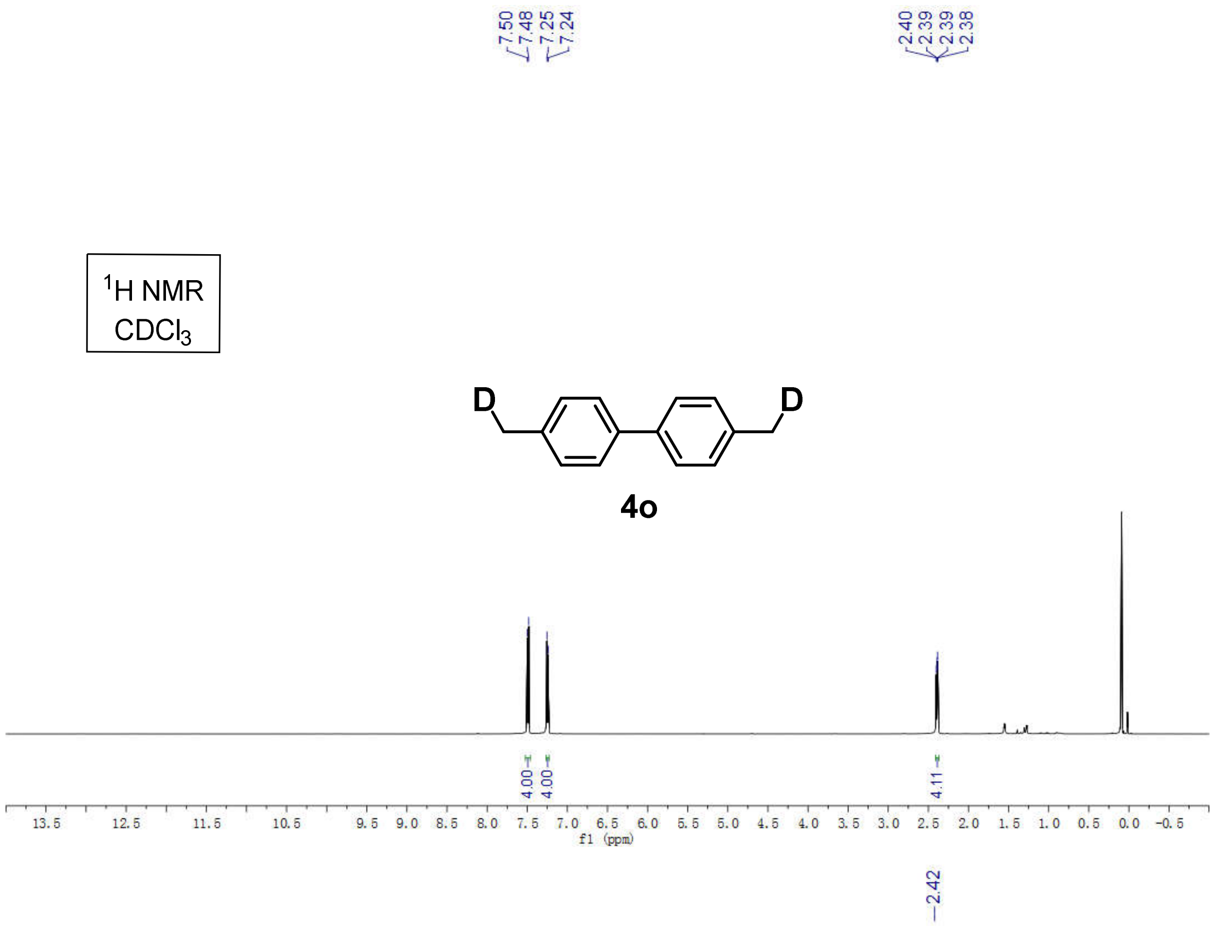
7.50
7.48
7.25
7.24
2.40
2.39
2.39
2.38
$^1$H NMR
$CDCl_3$
D
D
4o
4.00
4.00
4.11
13.5 12.5 11.5 10.5 9.5 9.0 8.5 8.0 7.5 7.0 6.5 6.0 5.5 5.0 4.5 4.0 3.5 3.0 2.5 2.0 1.5 1.0 0.5 0.0 -0.5
f1 (ppm)

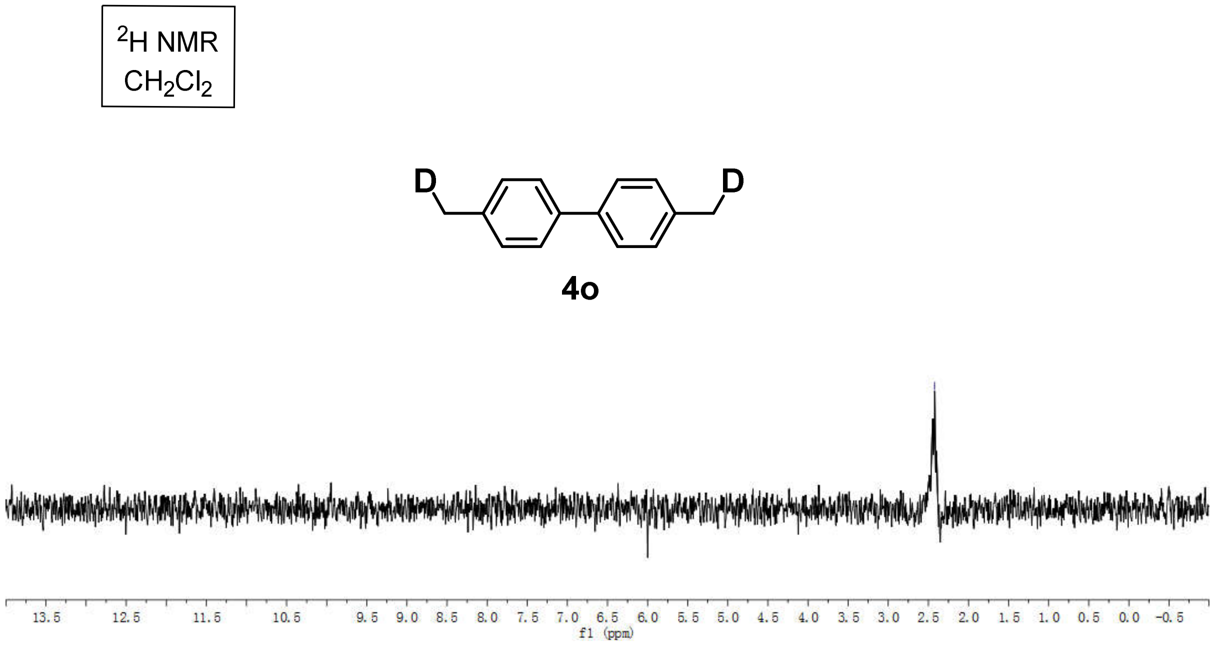
2.42
$^2$H NMR
$CH_2Cl_2$
D
D
4o
13.5 12.5 11.5 10.5 9.5 9.0 8.5 8.0 7.5 7.0 6.5 6.0 5.5 5.0 4.5 4.0 3.5 3.0 2.5 2.0 1.5 1.0 0.5 0.0 -0.5
f1 (ppm)

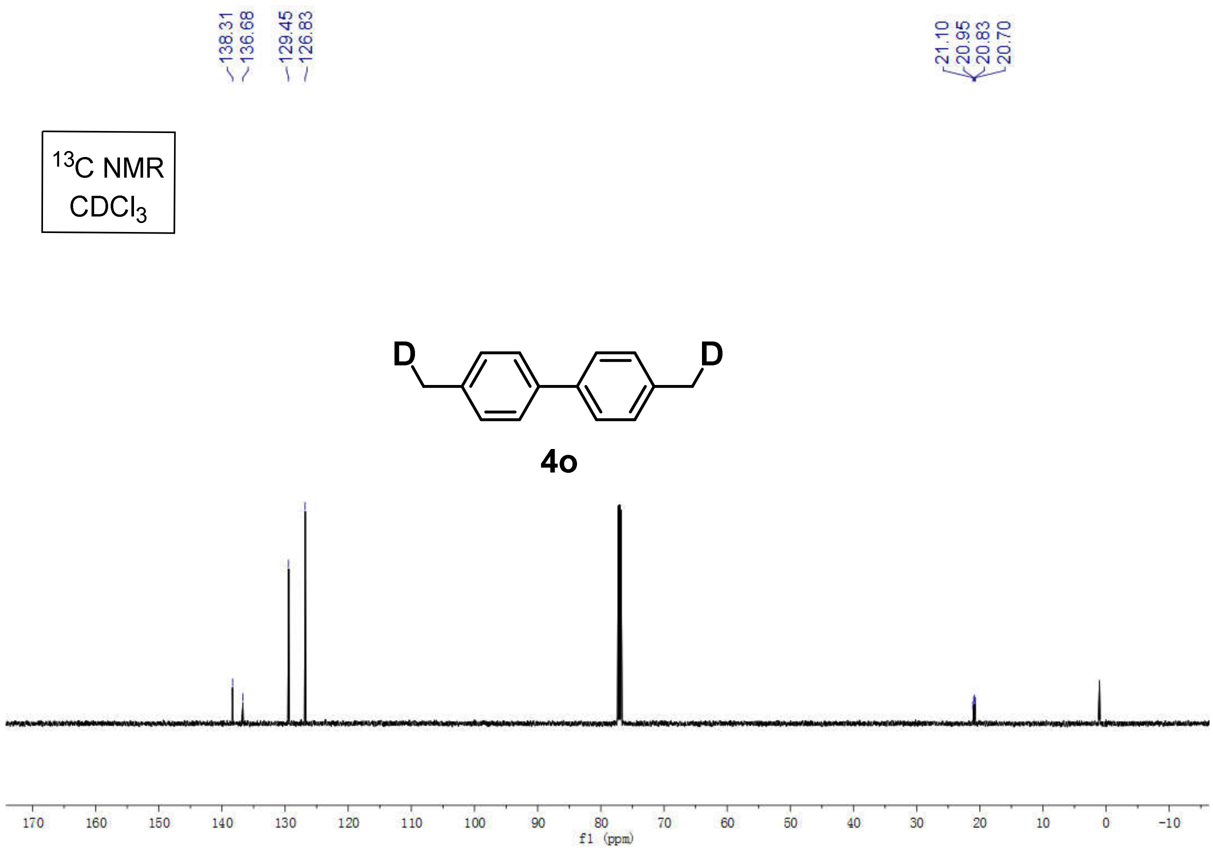


**Supplementary Fig. 85** $^{1}$H NMR, $^{2}$H NMR and $^{13}$C NMR spectra of the compound **4o**.

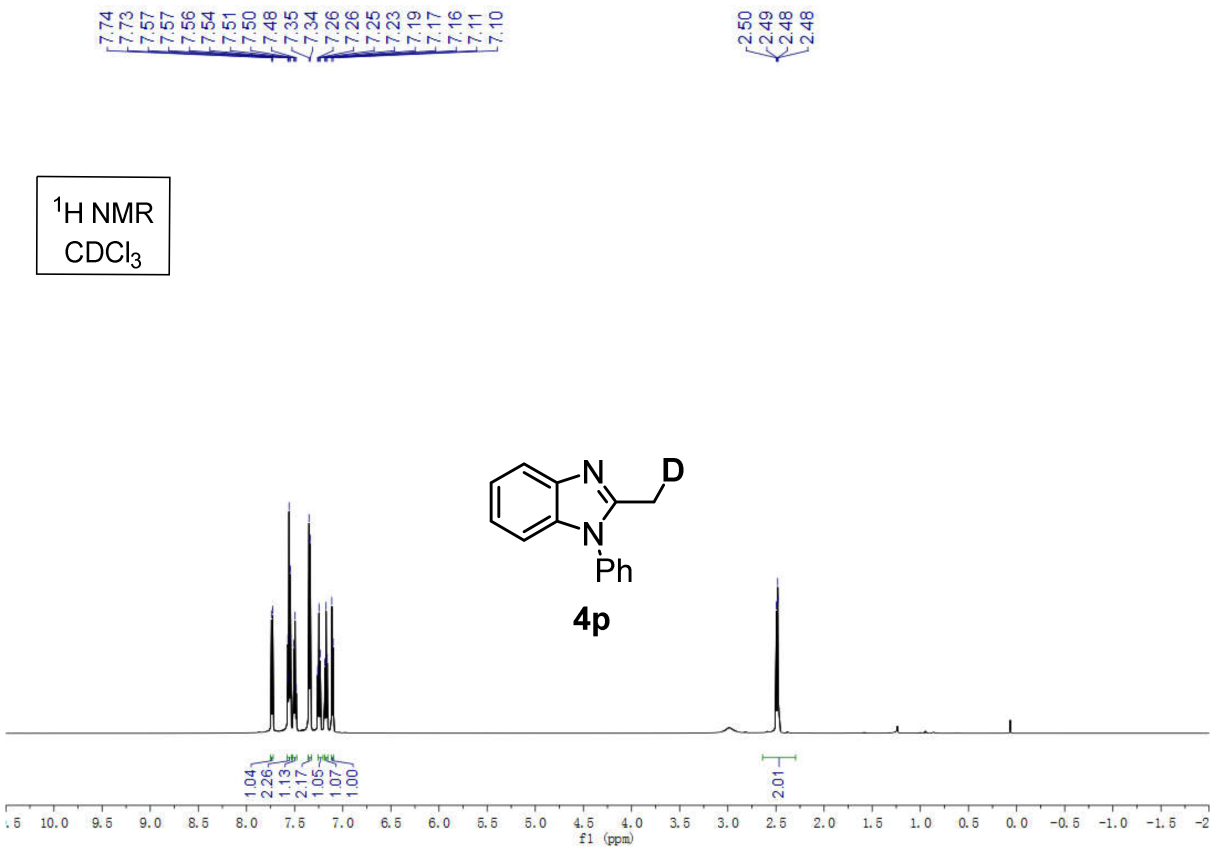
7.74
7.73
7.57
7.57
7.56
7.54
7.51
7.50
7.48
7.35
7.34
7.26
7.26
7.25
7.23
7.19
7.17
7.16
7.11
7.10
2.50
2.49
2.48
2.48
$^1$H NMR
$CDCl_3$
N
N
D
Ph
4p
1.04
2.26
1.13
2.17
1.05
1.07
1.00
2.01
f1 (ppm)

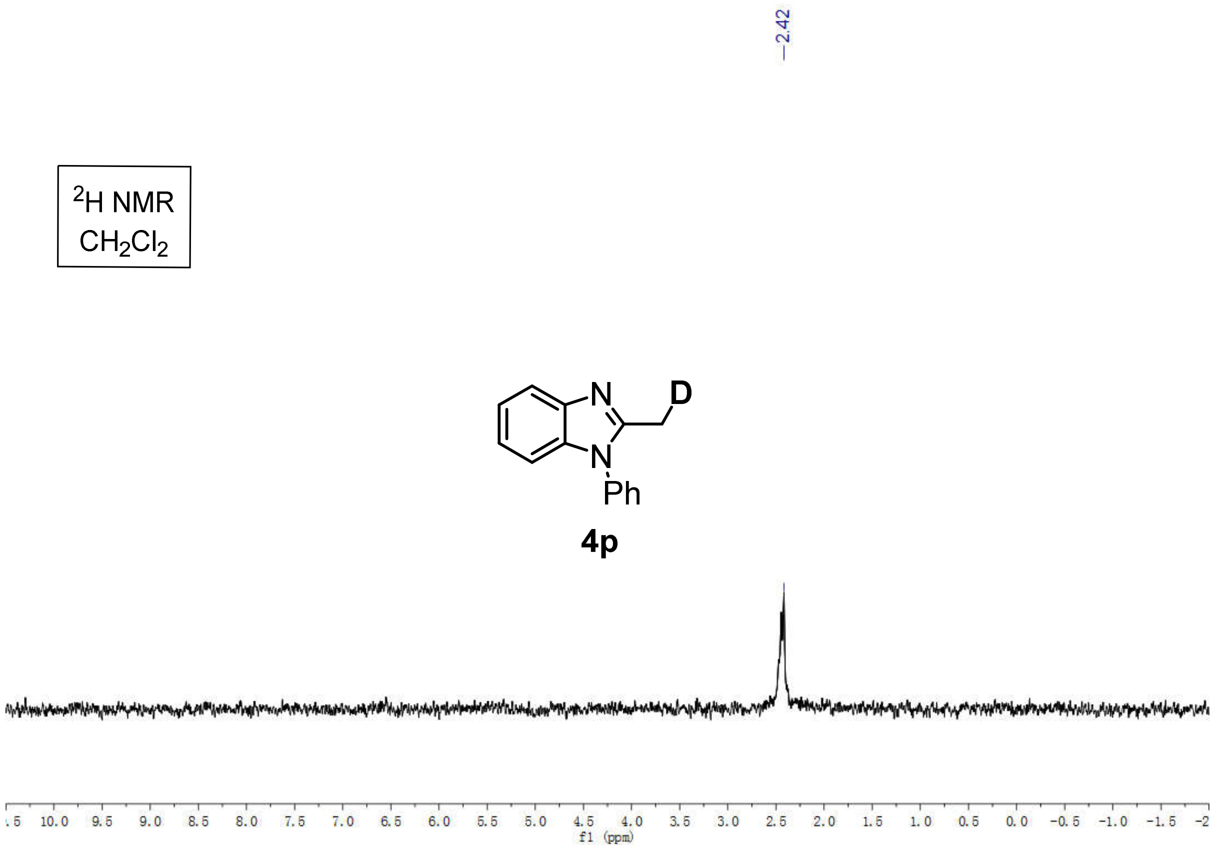
2.42
$^2$H NMR
$CH_2Cl_2$
N
N
D
Ph
4p
f1 (ppm)

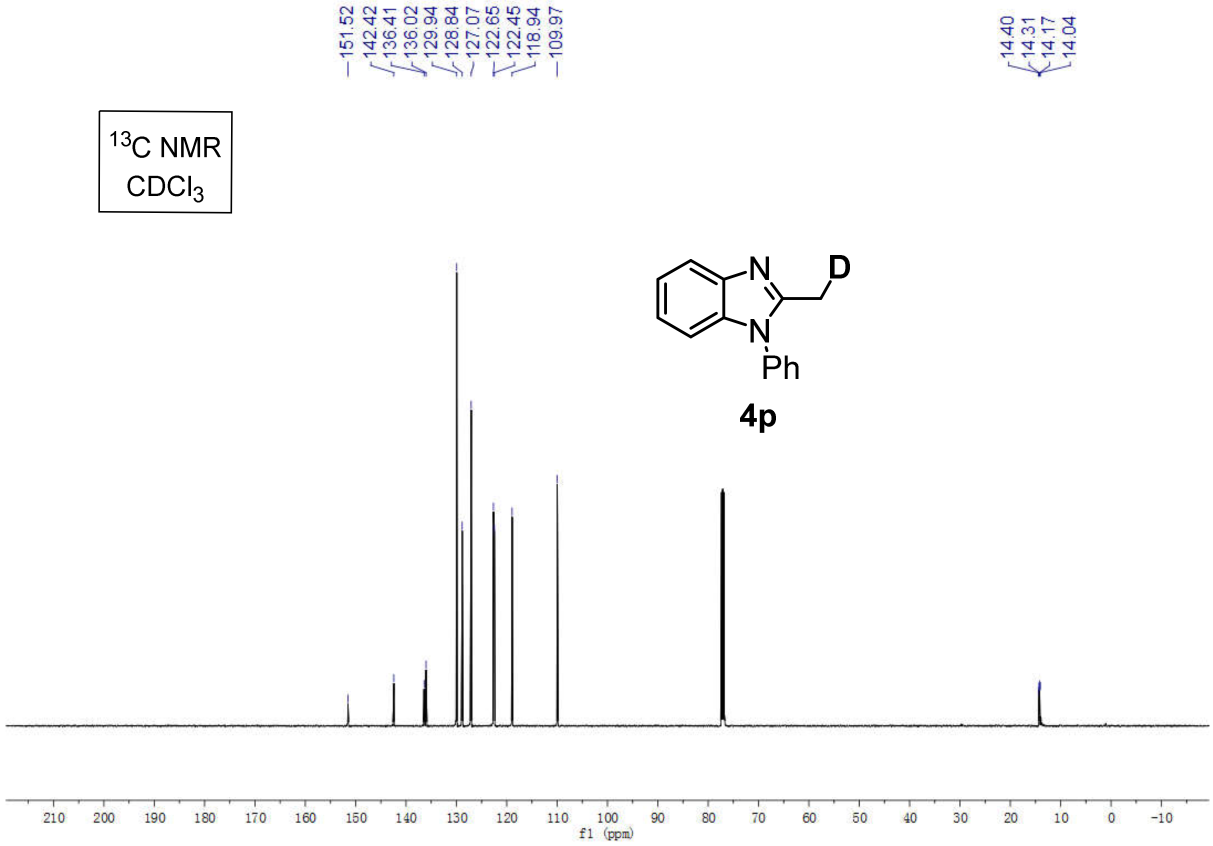


**Supplementary Fig. 86** $^{1}$H NMR, $^{2}$H NMR and $^{13}$C NMR spectra of the compound **4p**.

$^1$H NMR
$CDCl_3$

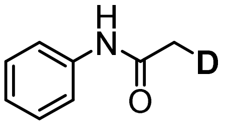


**4q**

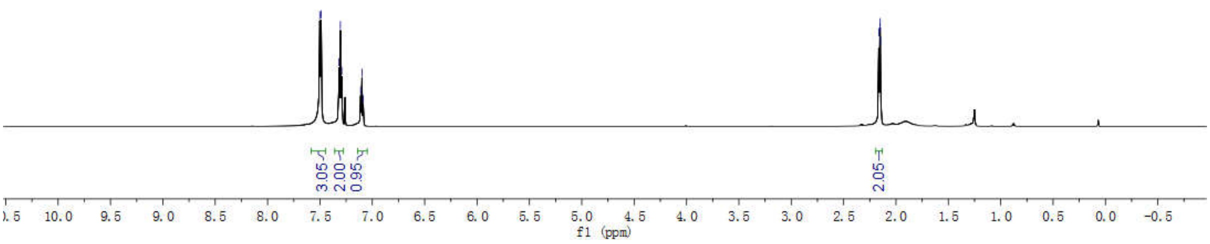




$^2$H NMR
$CH_2Cl_2$

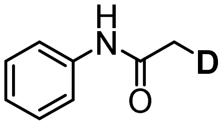


**4q**

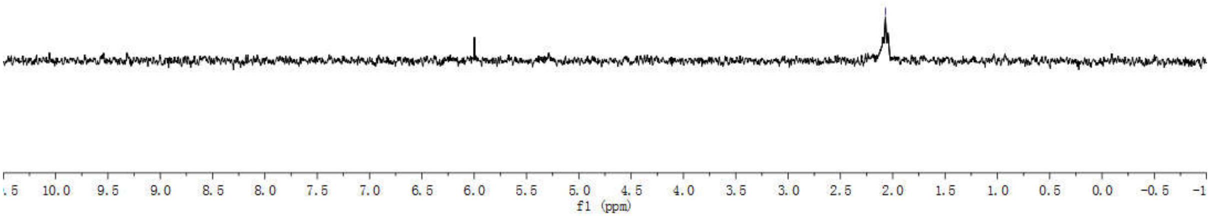

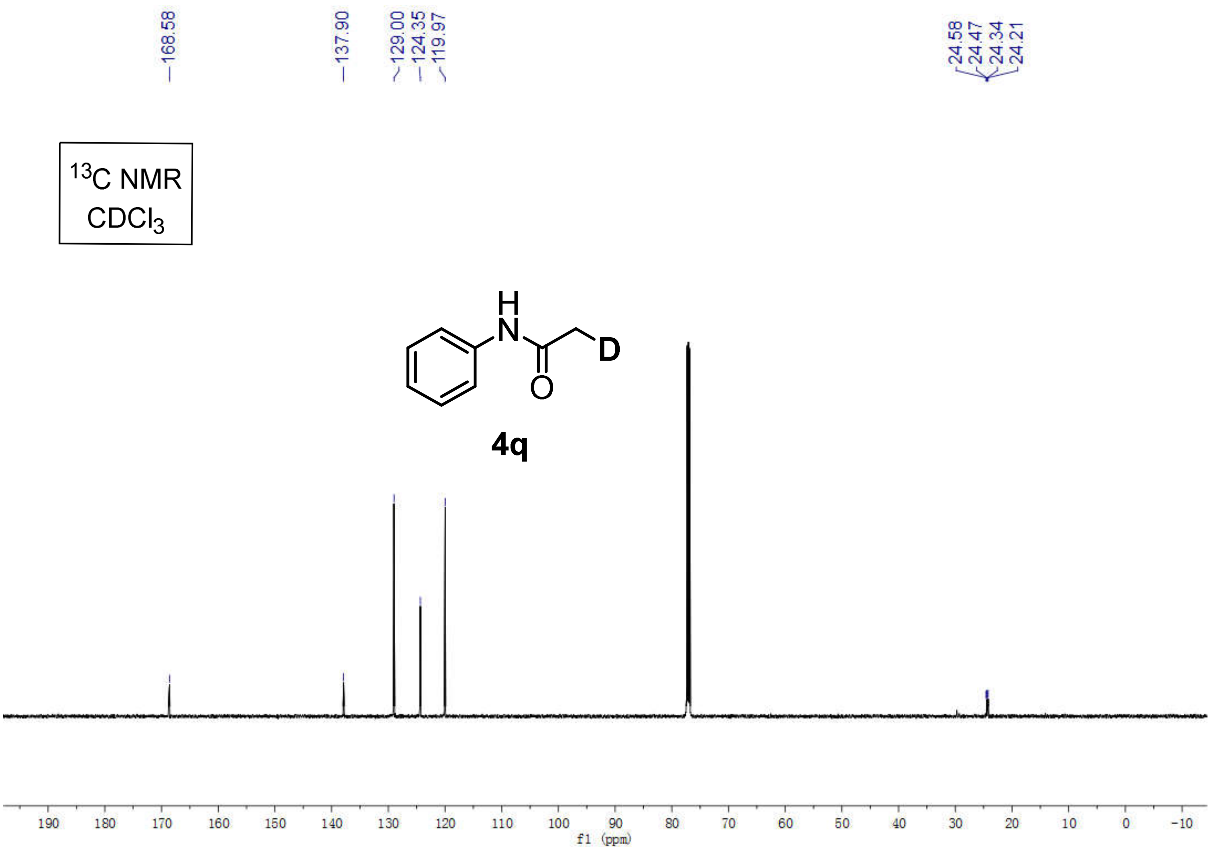


**Supplementary Fig. 87** $^{1}$H NMR, $^{2}$H NMR and $^{13}$C NMR spectra of the compound **4q**.

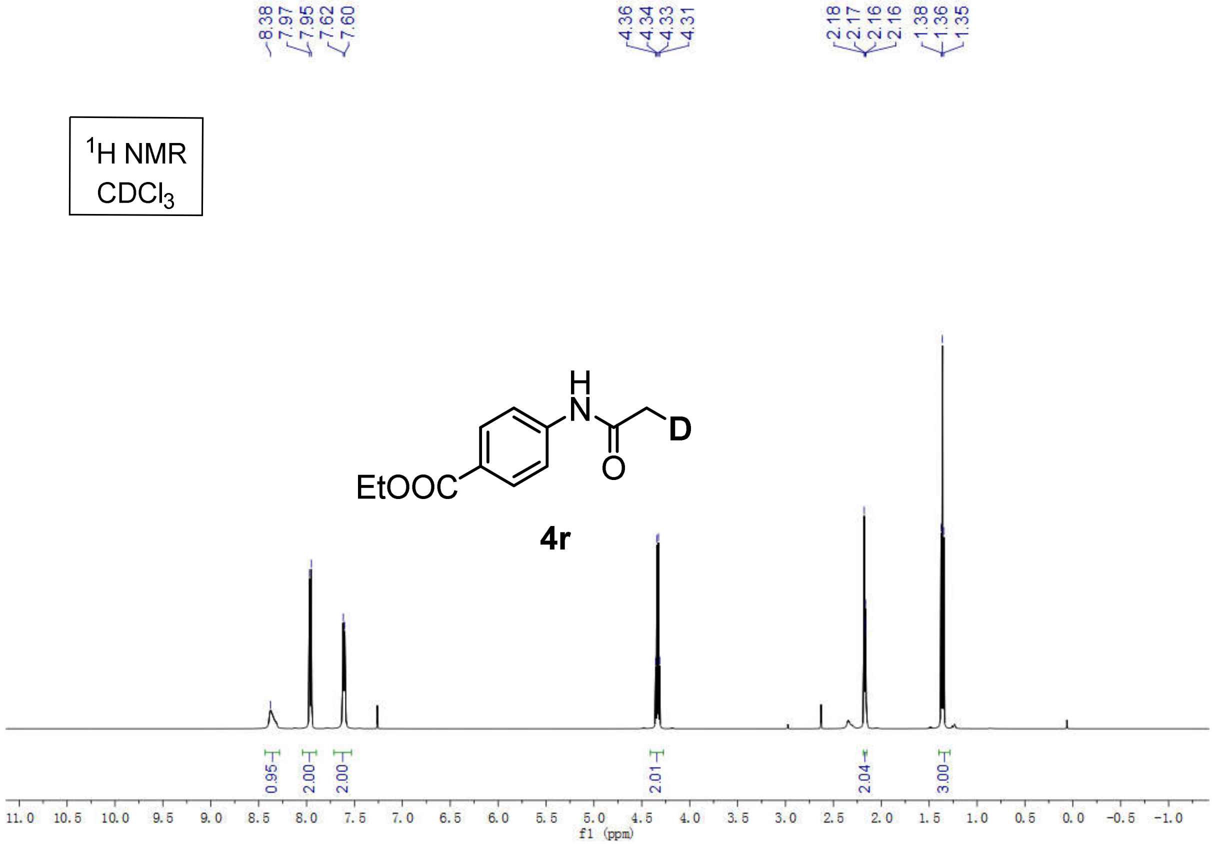

8.38
7.97
7.95
7.62
7.60
4.36
4.34
4.33
4.31
2.18
2.17
2.16
2.16
1.38
1.36
1.35
<sup></sup>
1H NMR
CDCl3
EtOOC
H
N
O
D
4r
0.95
2.00
2.00
2.01
2.04
3.00
f1 (ppm)


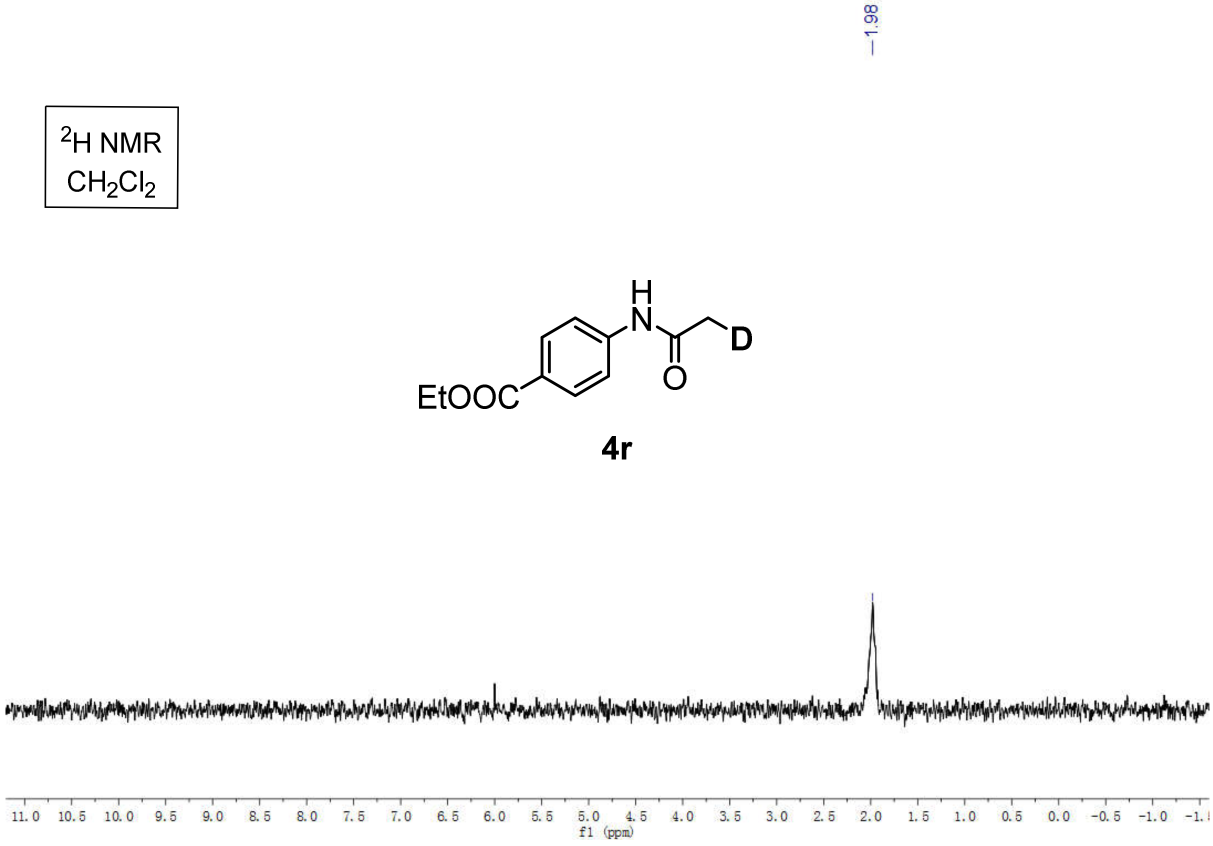

1.98
2H NMR
CH2Cl2
EtOOC
H
N
O
D
4r
f1 (ppm)

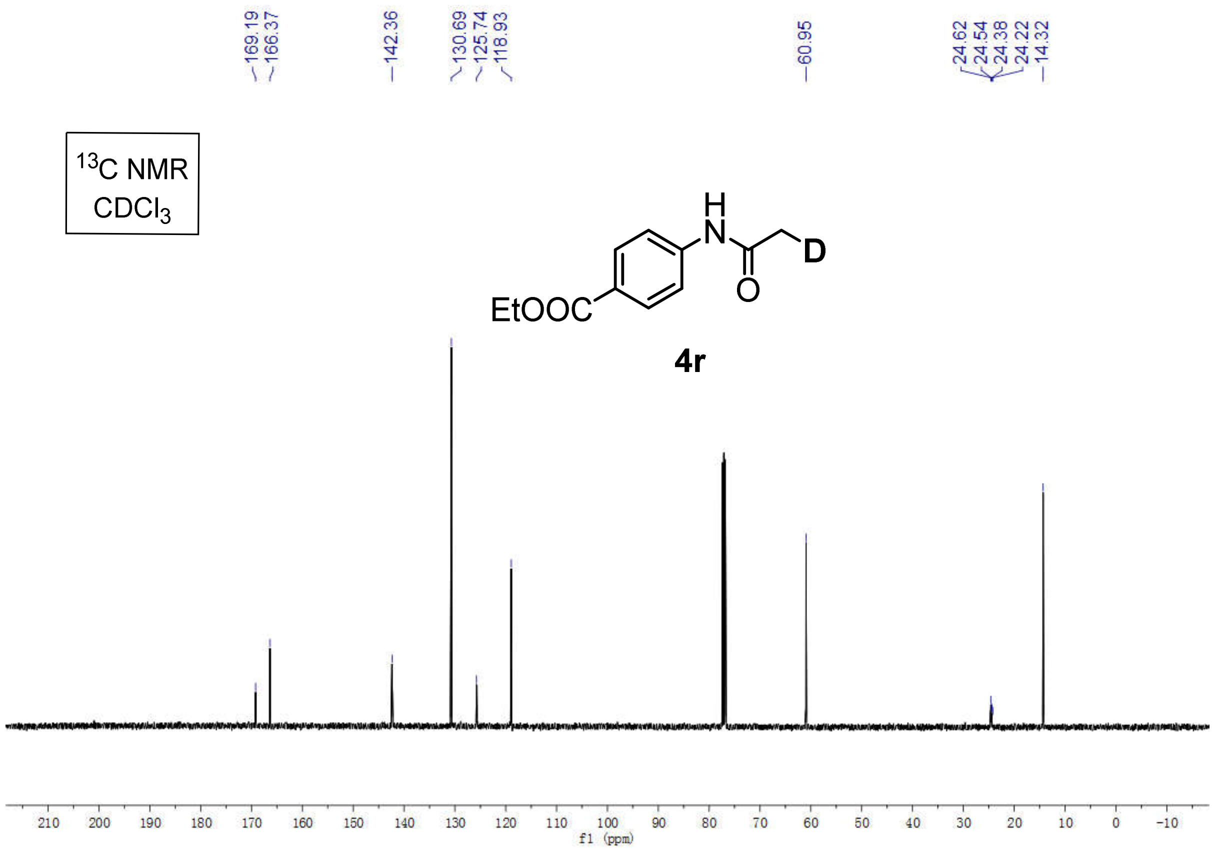


**Supplementary Fig. 88** $^{1}$H NMR, $^{2}$H NMR and $^{13}$C NMR spectra of the compound **4r**.

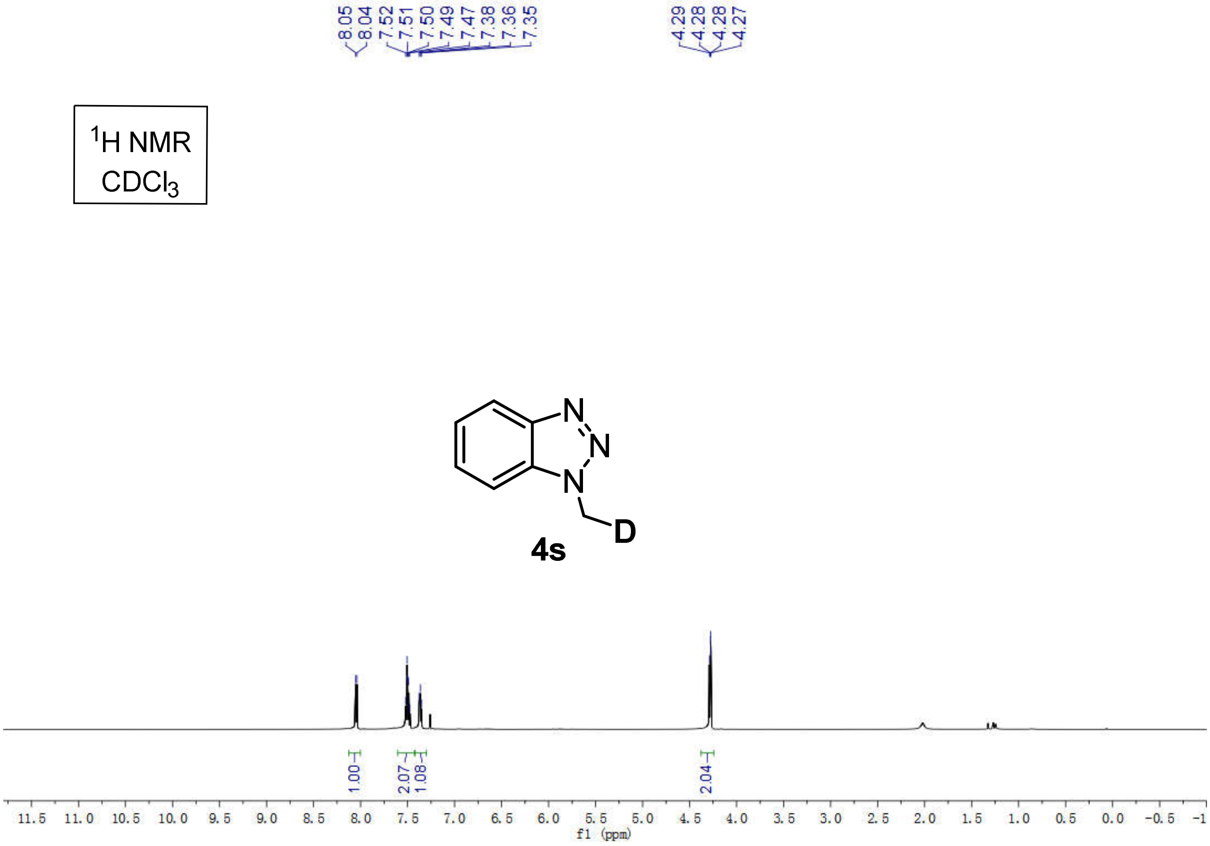
8.05
8.04
7.52
7.51
7.50
7.49
7.47
7.38
7.36
7.35
4.29
4.28
4.28
4.27
$^1$H NMR
$CDCl_3$
4s
D
1.00
2.07
1.08
2.04
11.5 11.0 10.5 10.0 9.5 9.0 8.5 8.0 7.5 7.0 6.5 6.0 5.5 5.0 4.5 4.0 3.5 3.0 2.5 2.0 1.5 1.0 0.5 0.0 -0.5 -1
f1 (ppm)

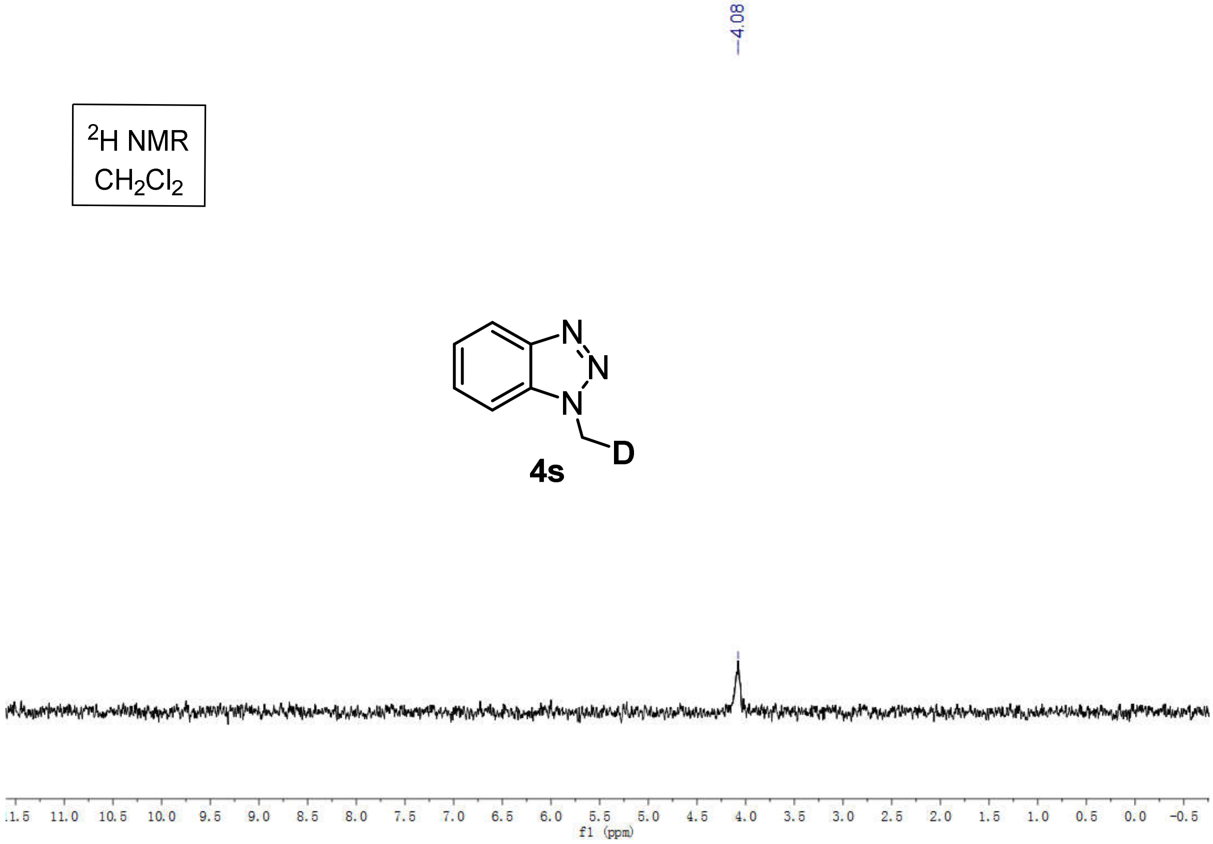
4.08
$^2$H NMR
$CH_2Cl_2$
4s
D
11.5 11.0 10.5 10.0 9.5 9.0 8.5 8.0 7.5 7.0 6.5 6.0 5.5 5.0 4.5 4.0 3.5 3.0 2.5 2.0 1.5 1.0 0.5 0.0 -0.5
f1 (ppm)

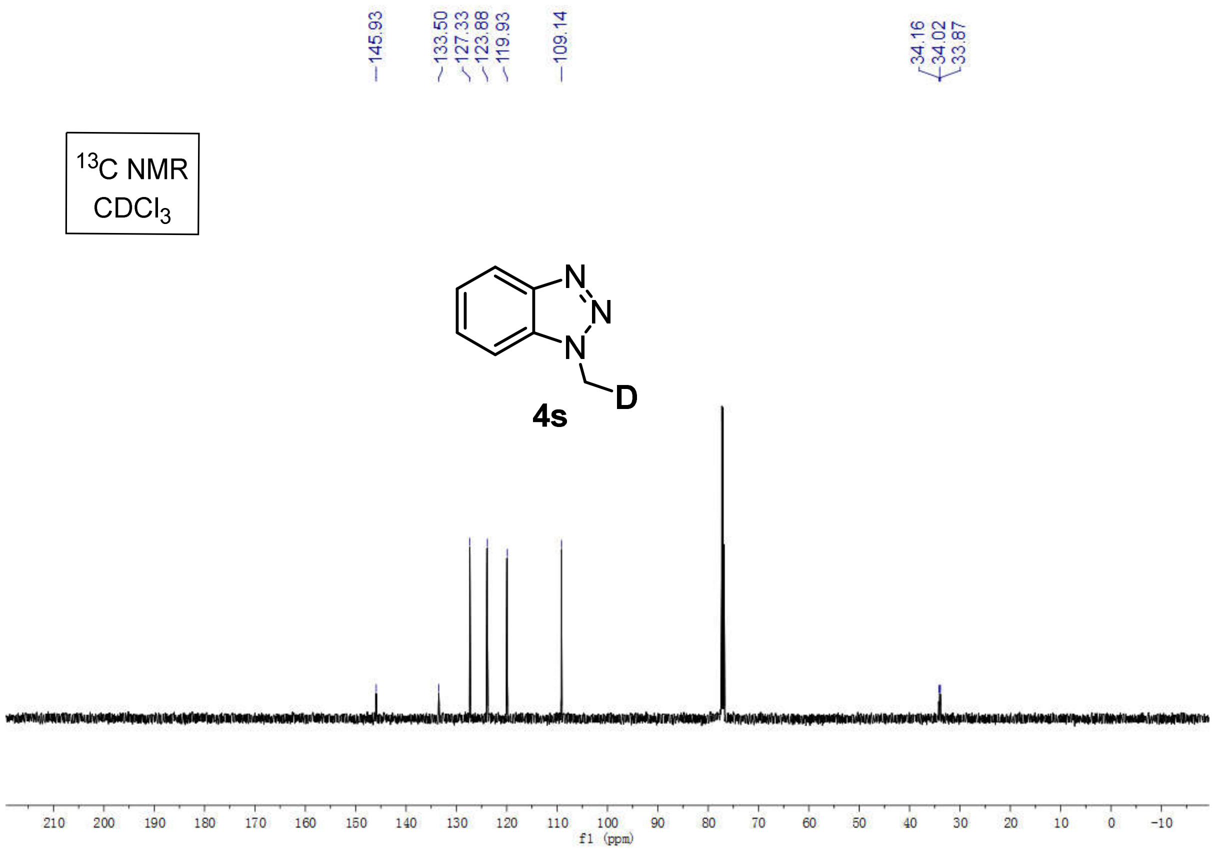


**Supplementary Fig. 89** $^1$H NMR, $^2$H NMR and $^{13}$C NMR spectra of the compound **4s**.

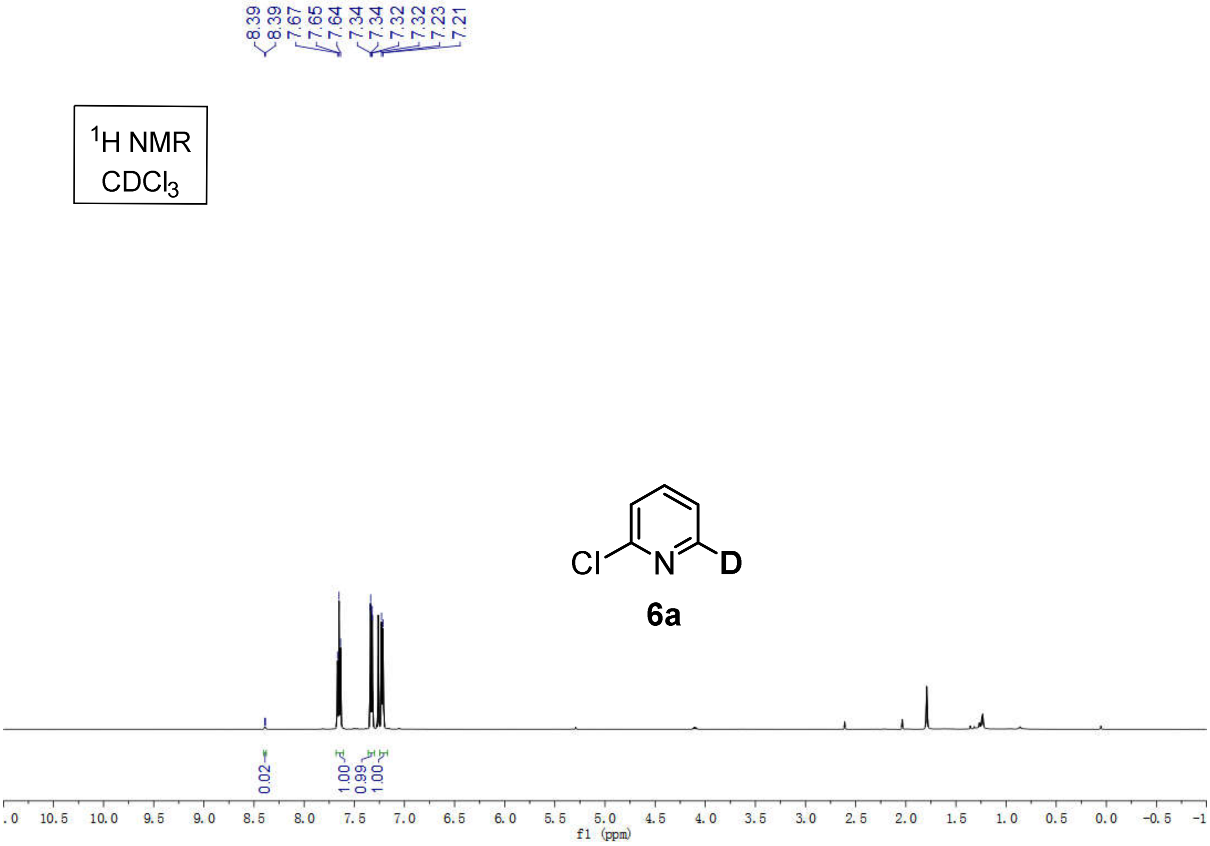
$^1H$ NMR
$CDCl_3$
8.39 8.39 7.67 7.65 7.64 7.34 7.34 7.32 7.32 7.23 7.21
Cl N D
6a
0.02 1.00 0.99 1.00
f1 (ppm)

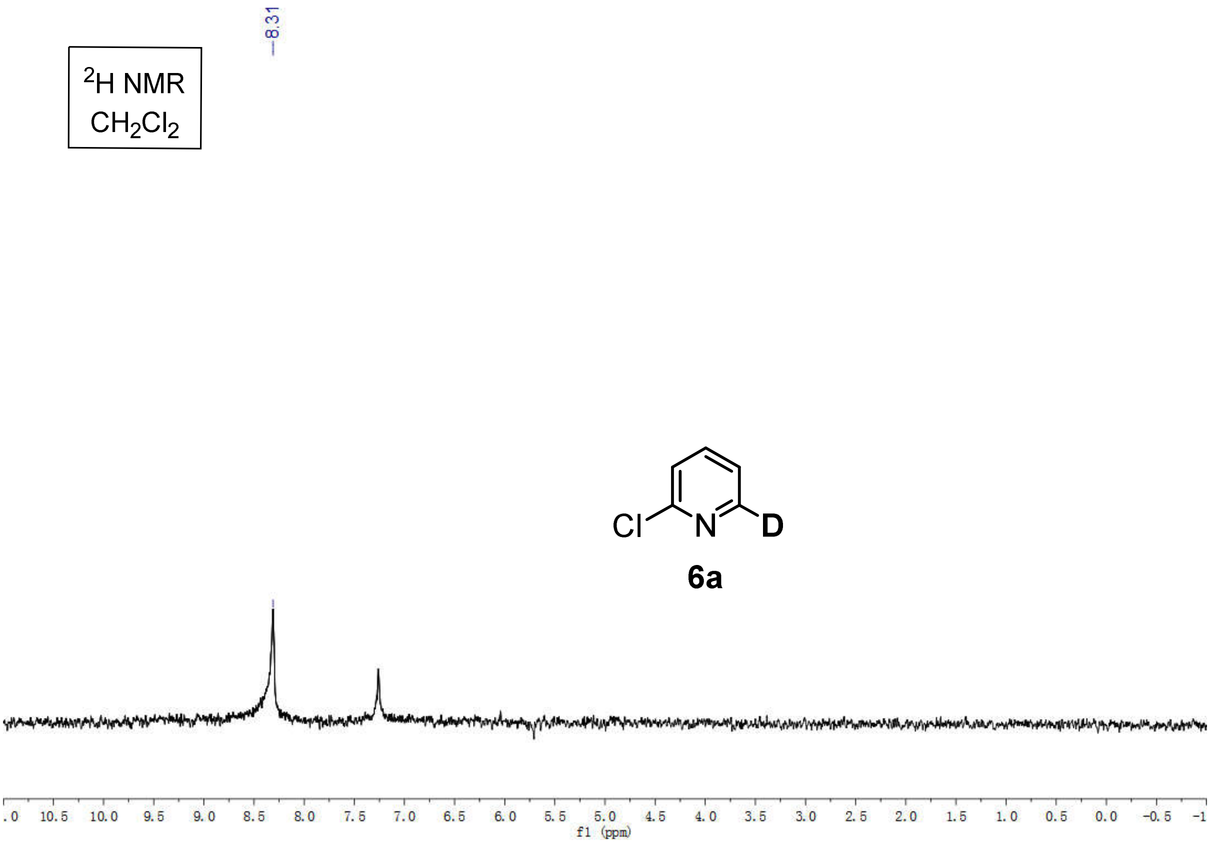
$^2H$ NMR
$CH_2Cl_2$
8.31
Cl N D
6a
f1 (ppm)

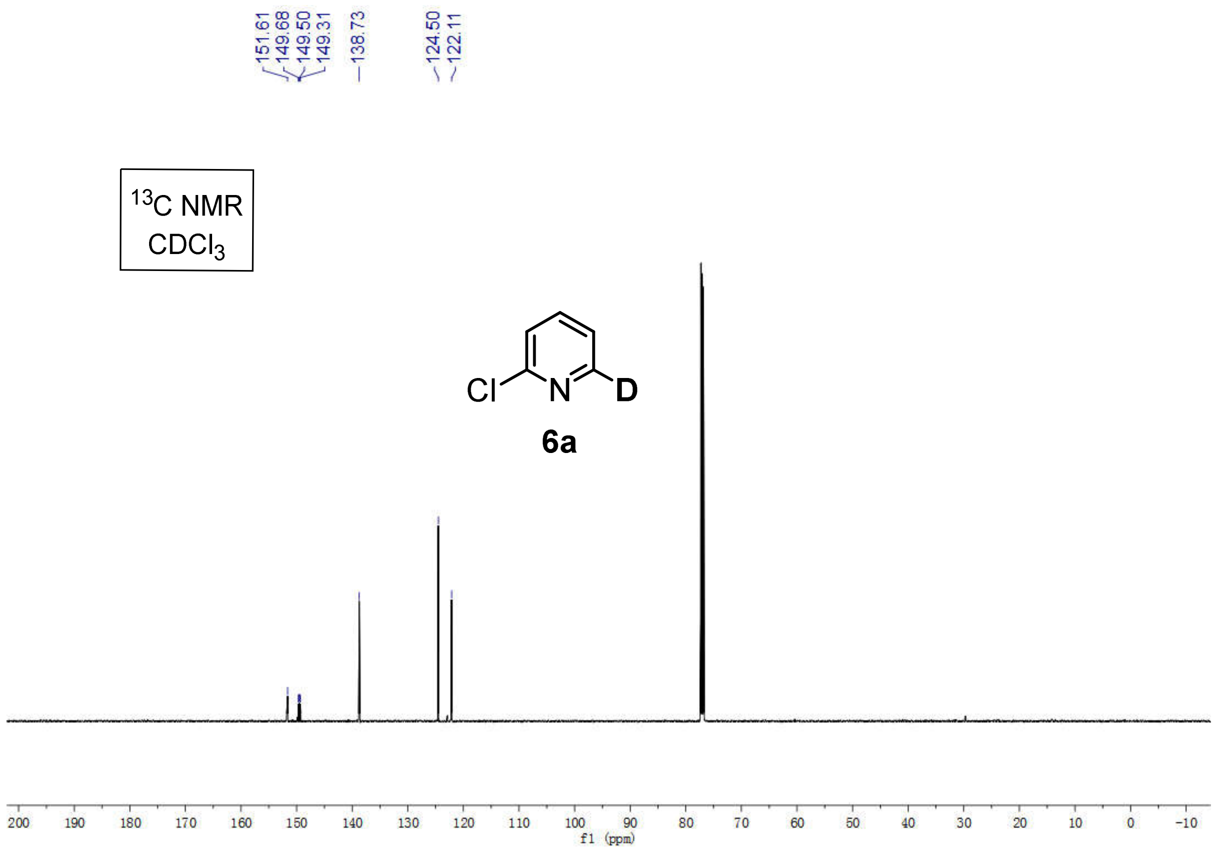


**Supplementary Fig. 90** $^{1}$H NMR, $^{2}$H NMR and $^{13}$C NMR spectra of the compound **6a**.

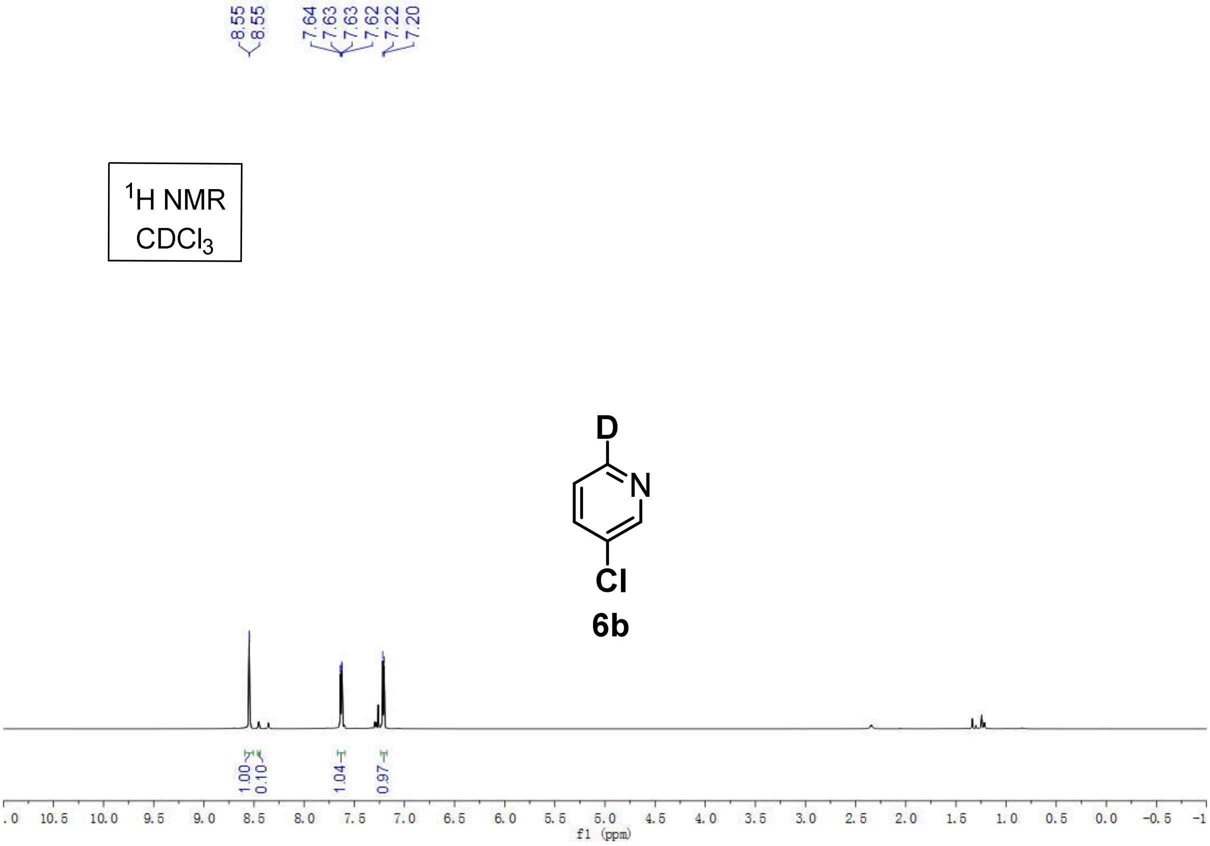

8.55
8.55
7.64
7.63
7.63
7.62
7.22
7.20
1H NMR
CDCl3
D
N
Cl
6b
1.00
0.10
1.04
0.97
f1 (ppm)


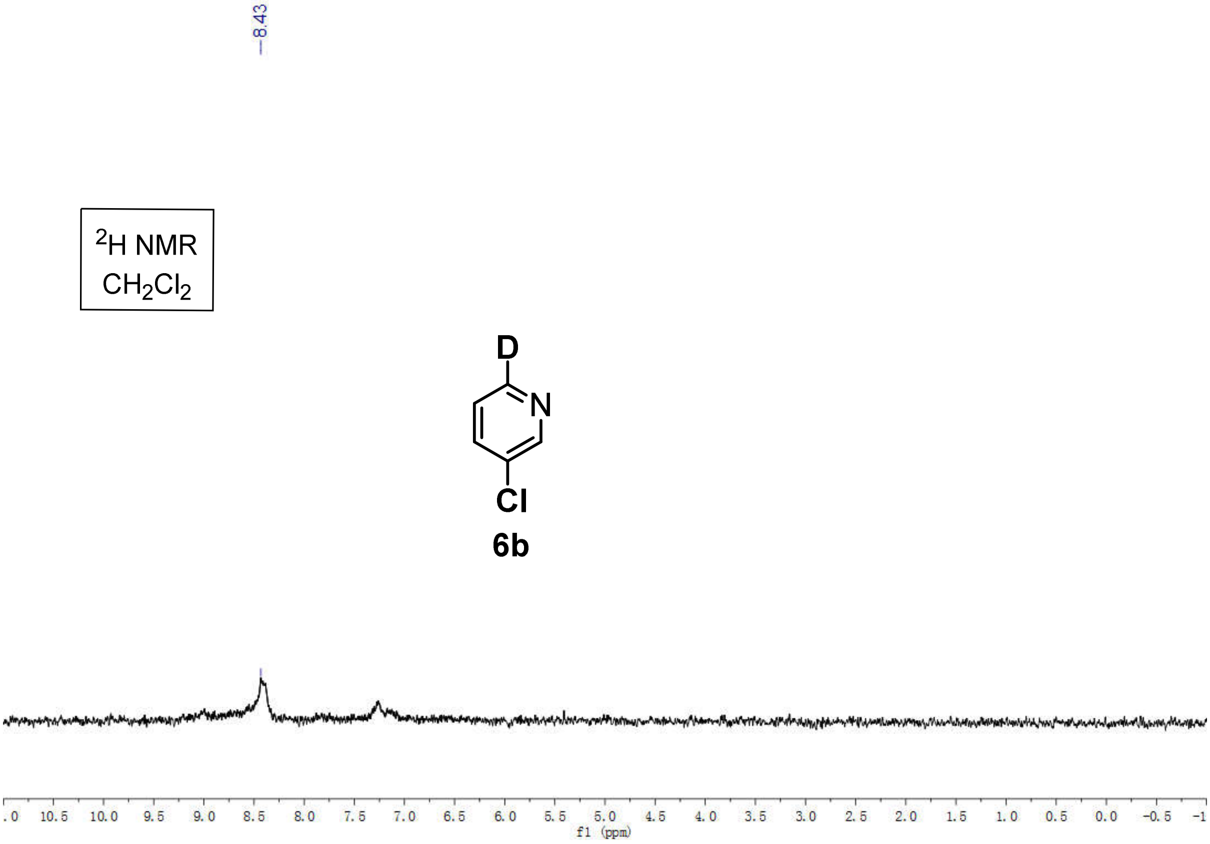

8.43
2H NMR
CH2Cl2
D
N
Cl
6b
f1 (ppm)

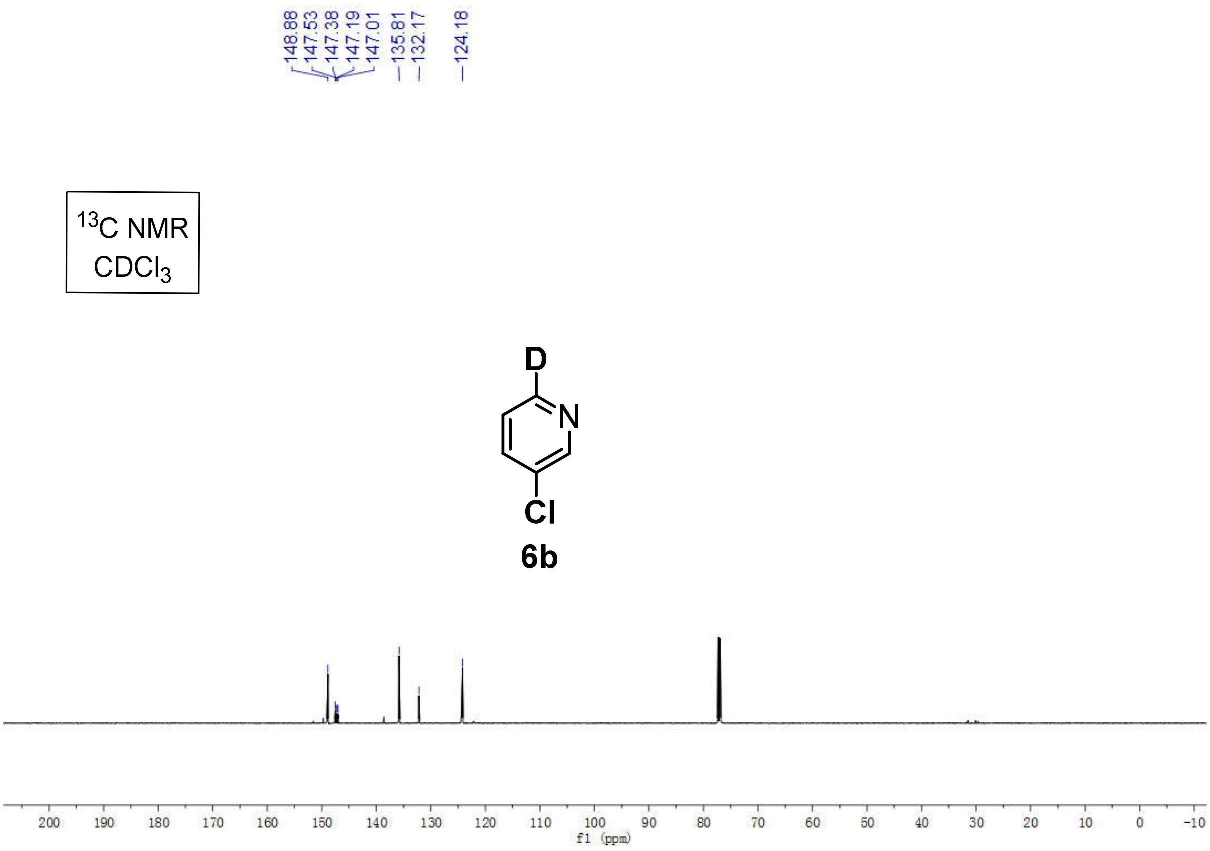


**Supplementary Fig. 91** $^{1}$H NMR, $^{2}$H NMR and $^{13}$C NMR spectra of the compound **6b**.

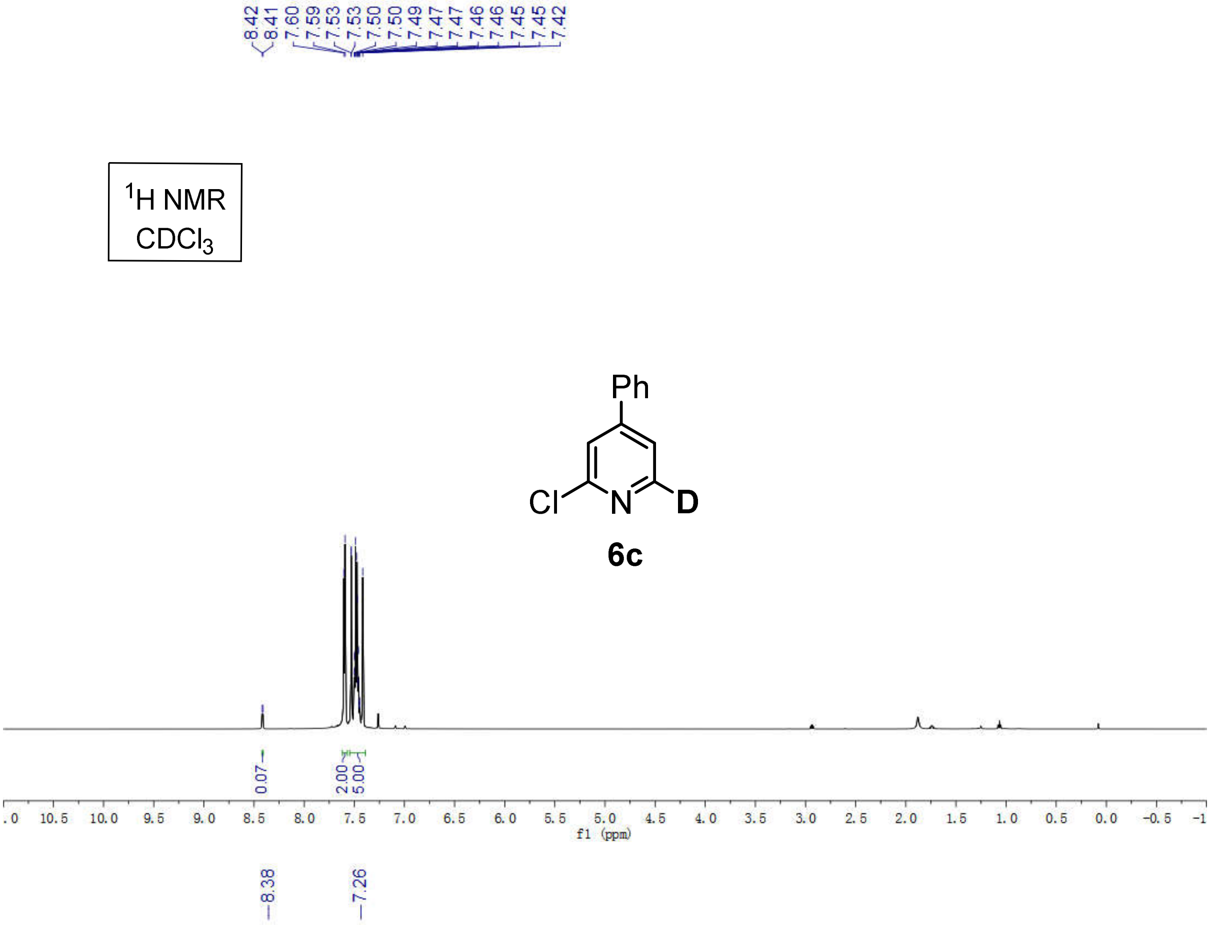
8.42
8.41
7.60
7.59
7.53
7.53
7.50
7.50
7.49
7.47
7.47
7.46
7.46
7.45
7.45
7.42
$^1$H NMR
$CDCl_3$
Ph
Cl
N
D
6c
0.07
2.00
5.00
f1 (ppm)
8.38
7.26

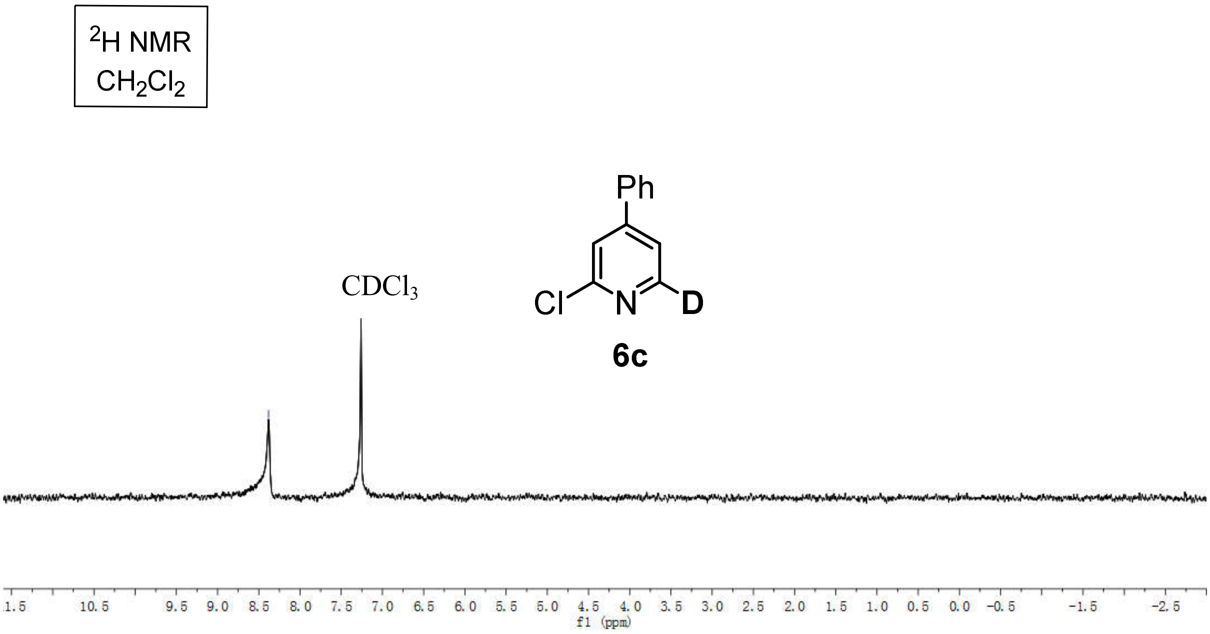
$^2$H NMR
$CH_2Cl_2$
$CDCl_3$
Ph
Cl
N
D
6c
f1 (ppm)

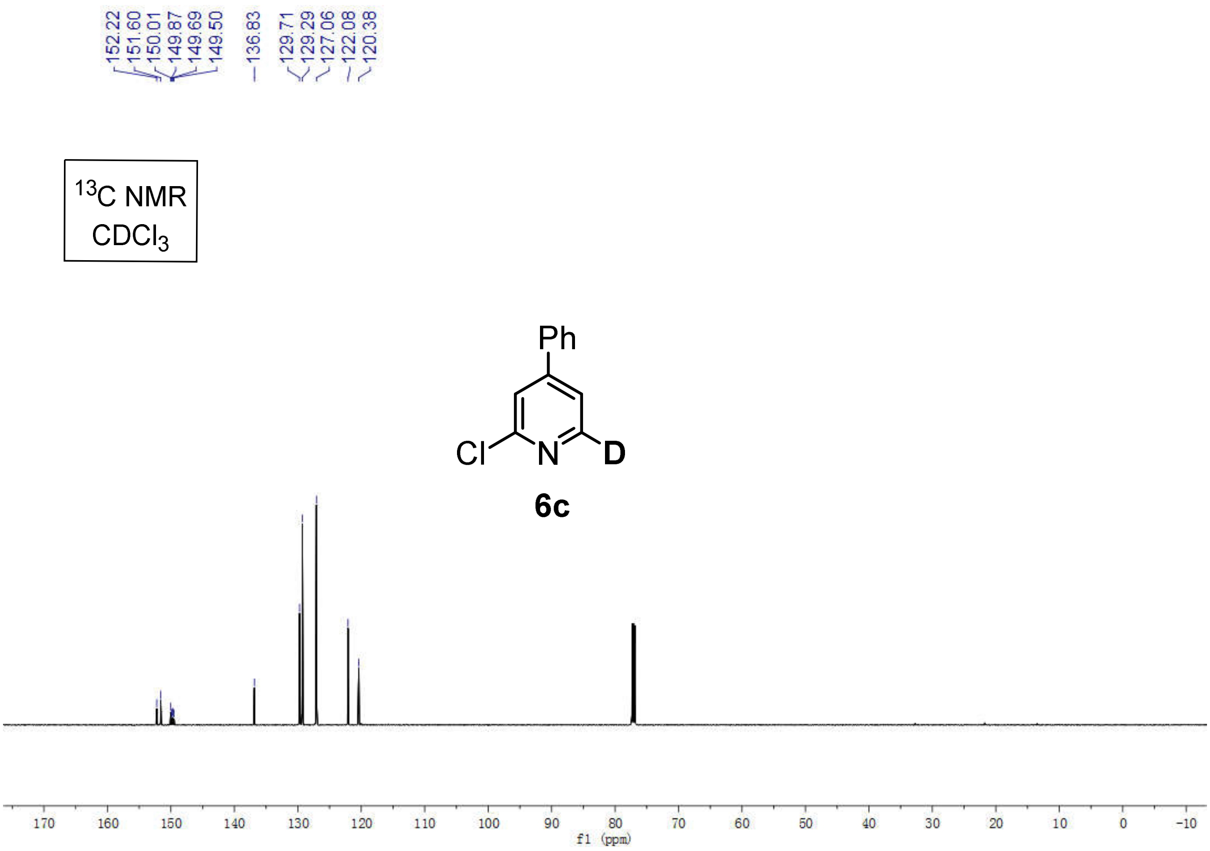


**Supplementary Fig. 92** $^{1}H$ NMR, $^{2}H$ NMR and $^{13}C$ NMR spectra of the compound **6c**.

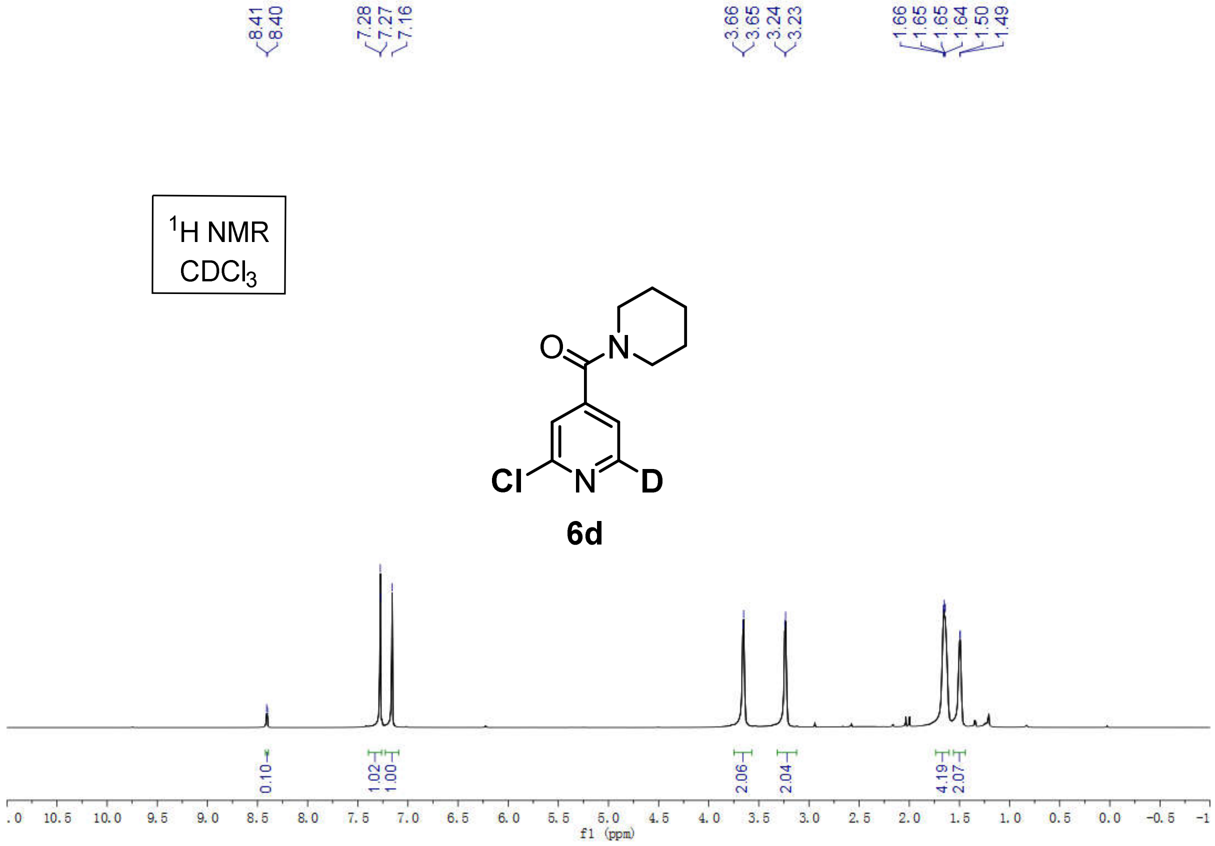

8.41
8.40
7.28
7.27
7.16
3.66
3.65
3.24
3.23
1.66
1.65
1.65
1.64
1.50
1.49
$^1$H NMR
$CDCl_3$
O
N
Cl
N
D
6d
0.10
1.02
1.00
2.06
2.04
4.19
2.07
f1 (ppm)


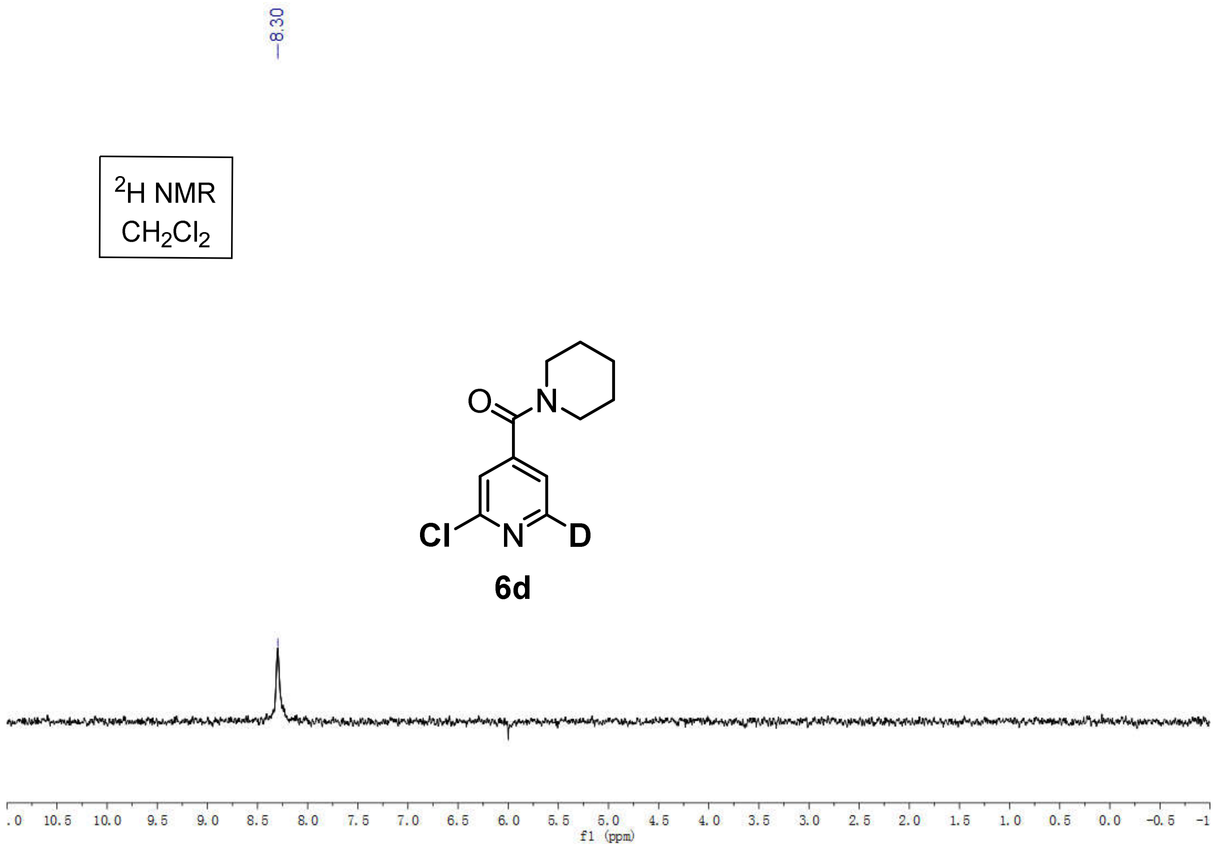

8.30
$^2$H NMR
$CH_2Cl_2$
O
N
Cl
N
D
6d
f1 (ppm)

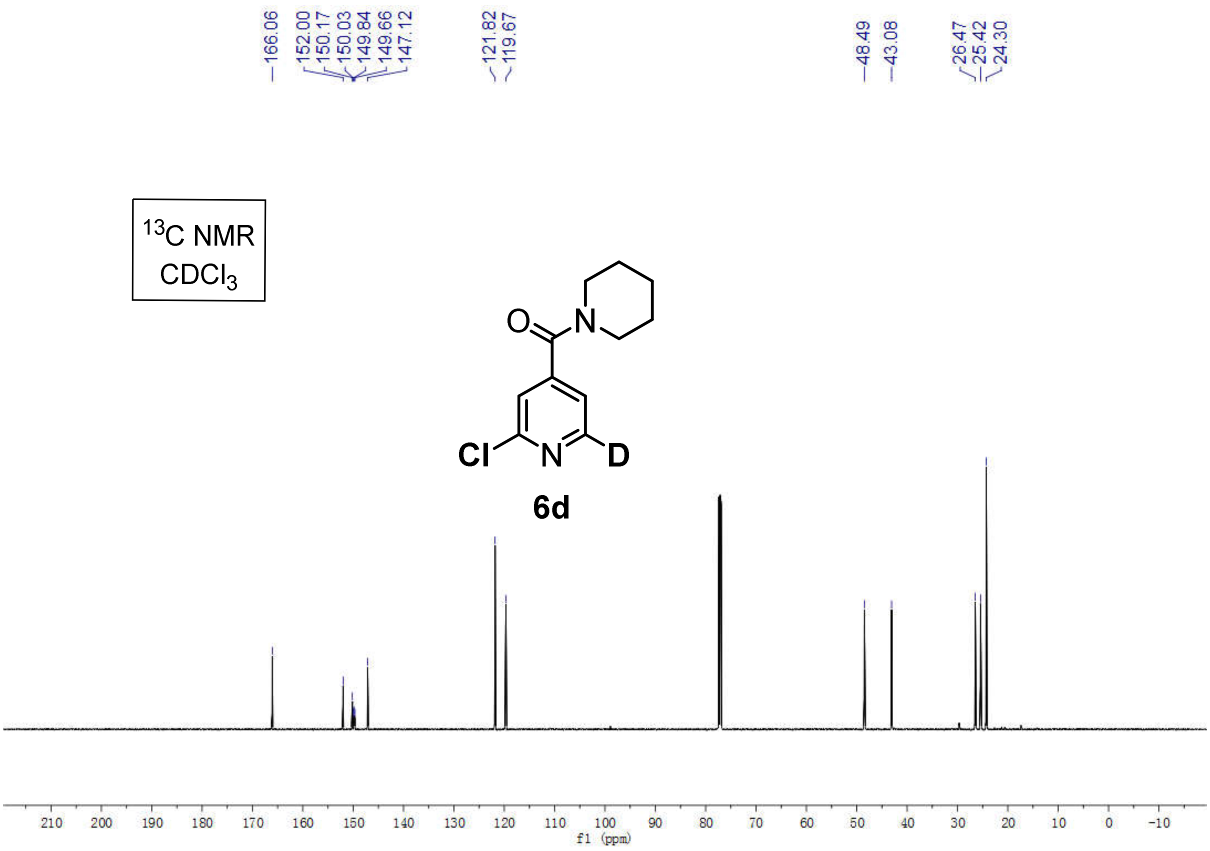


**Supplementary Fig. 93** $^{1}$H NMR, $^{2}$H NMR and $^{13}$C NMR spectra of the compound **6d**.

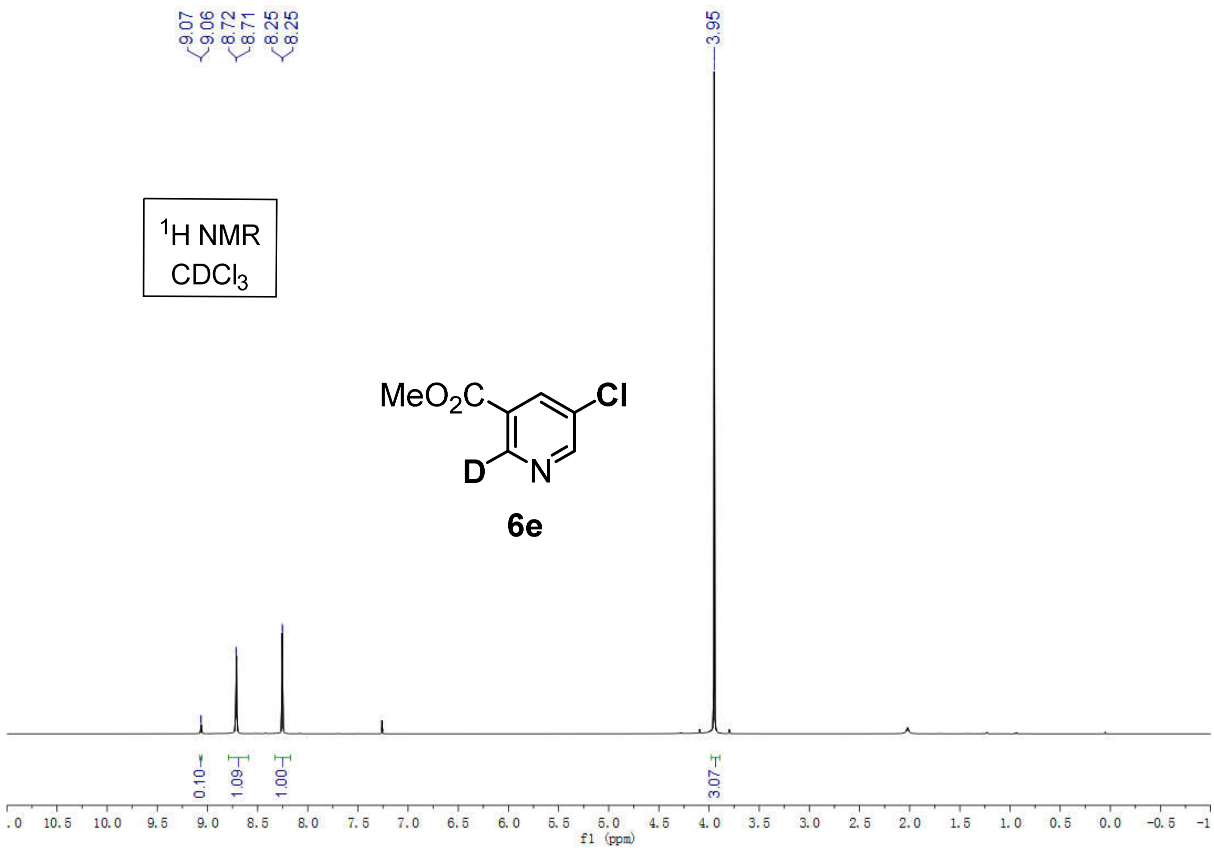
9.07
9.06
8.72
8.71
8.25
8.25
3.95
$^1$H NMR
$CDCl_3$
$MeO_2C$
Cl
D
N
6e
0.10
1.09
1.00
3.07
f1 (ppm)

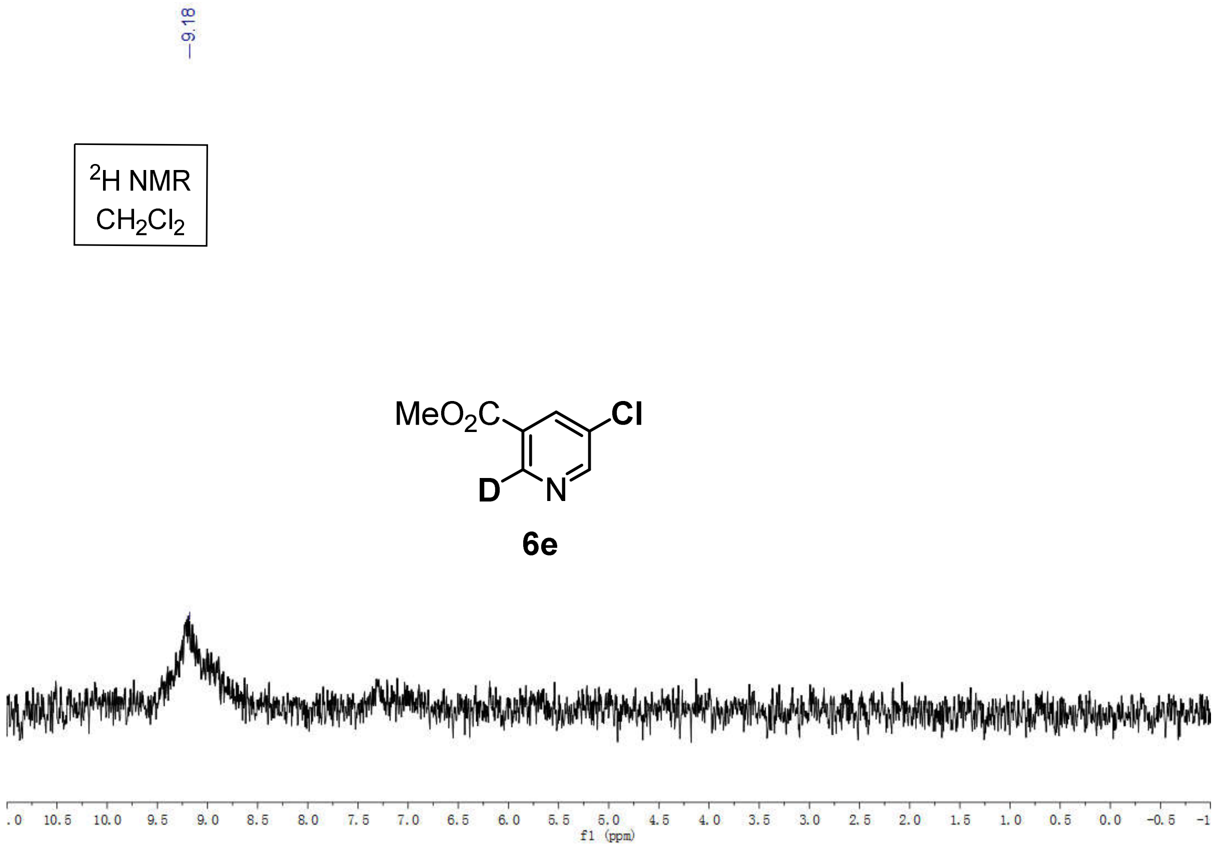
9.18
$^2$H NMR
$CH_2Cl_2$
$MeO_2C$
Cl
D
N
6e
f1 (ppm)

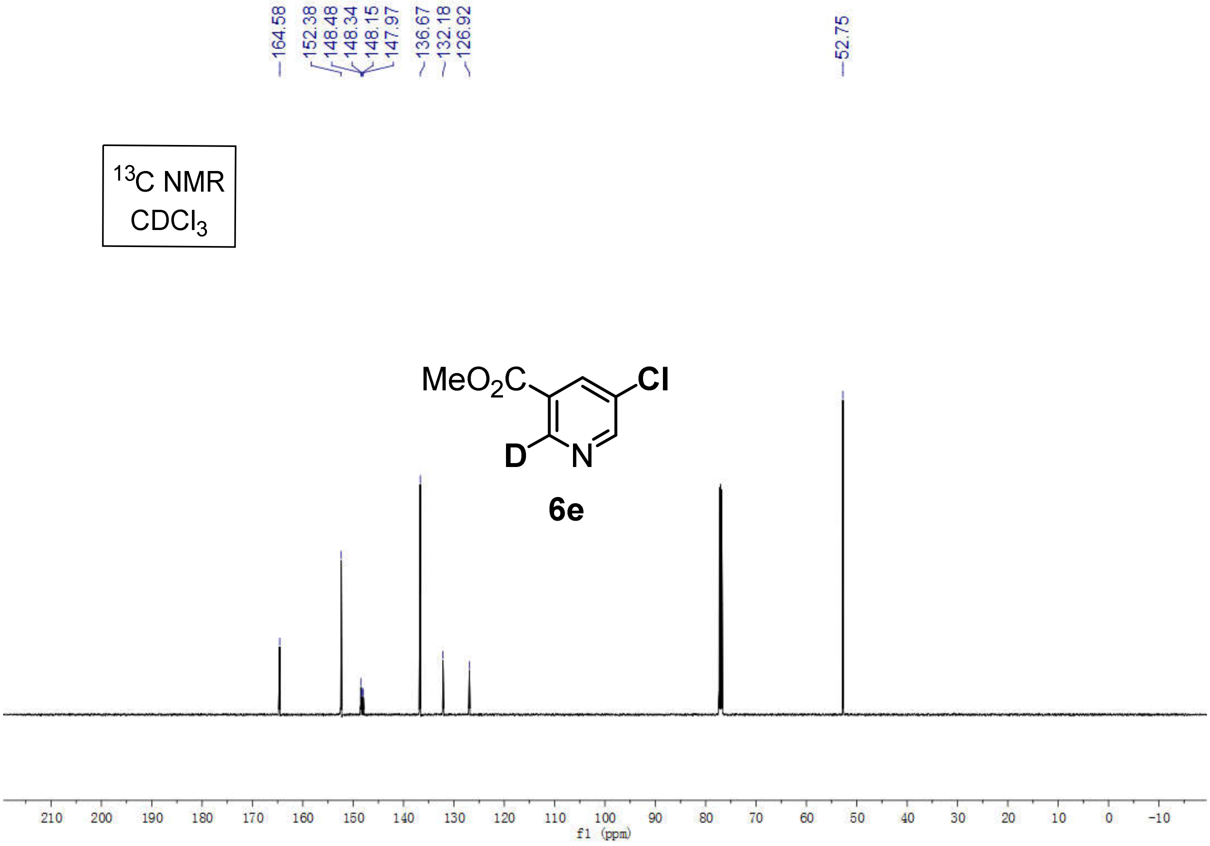


**Supplementary Fig. 94** $^{1}$H NMR, $^{2}$H NMR and $^{13}$C NMR spectra of the compound **6e**.

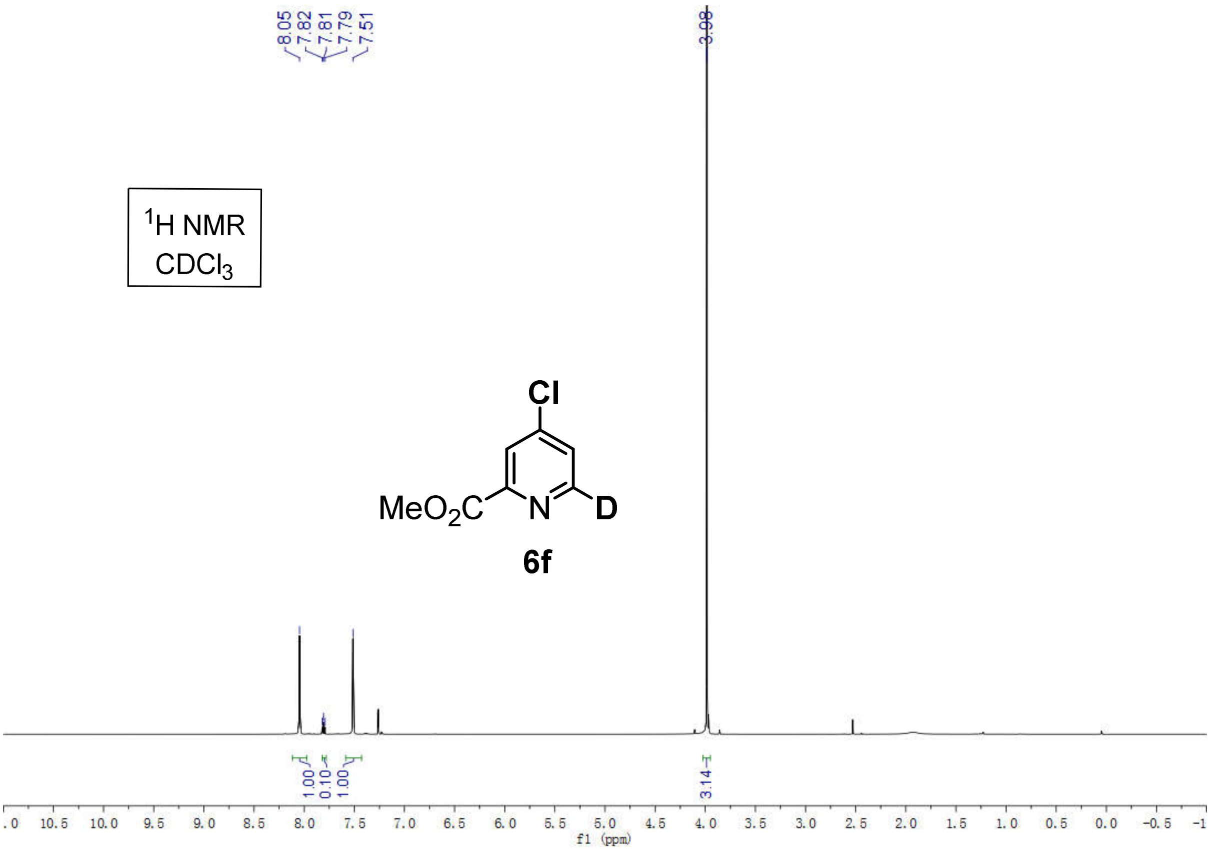
8.05
7.82
7.81
7.79
7.51
3.98
$^1$H NMR
$CDCl_3$
Cl
$MeO_2C$
N
D
6f
1.00
0.10
1.00
3.14
f1 (ppm)

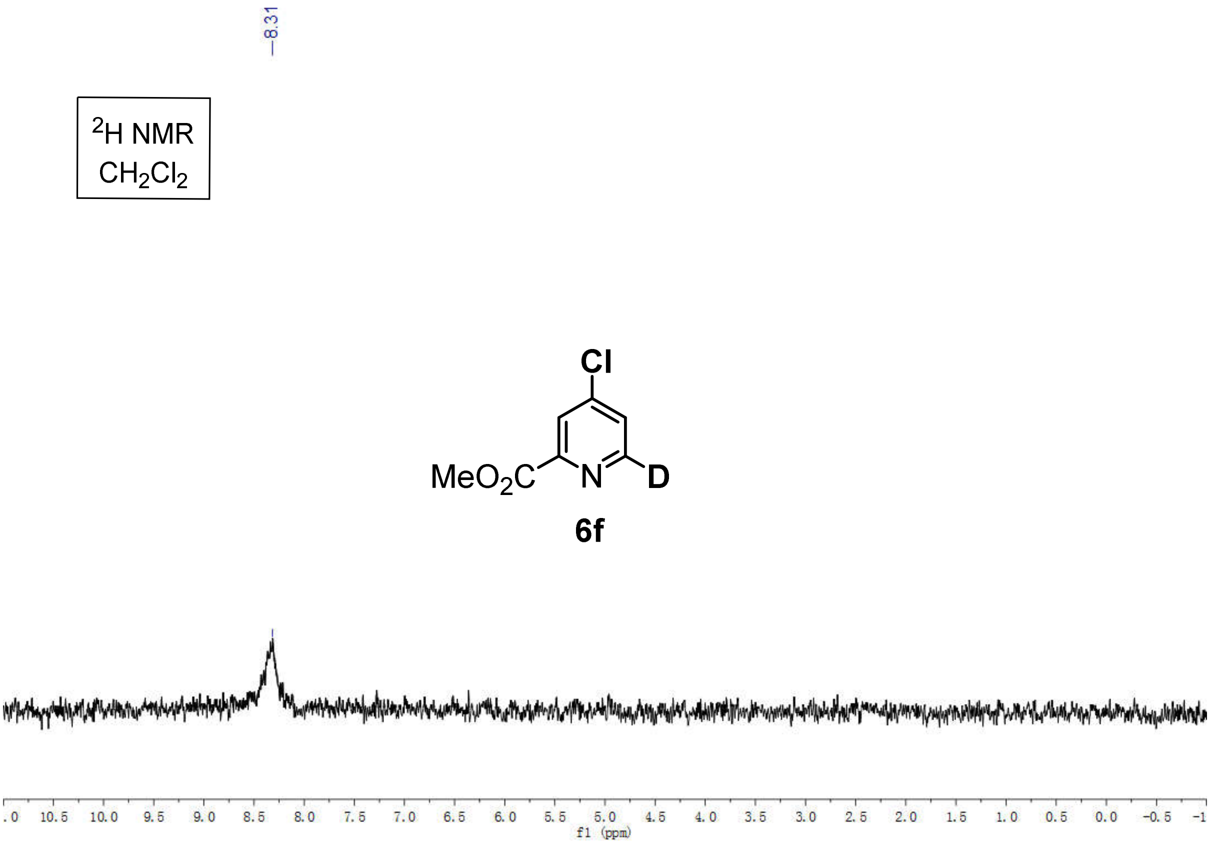
8.31
$^2$H NMR
$CH_2Cl_2$
Cl
$MeO_2C$
N
D
6f
f1 (ppm)

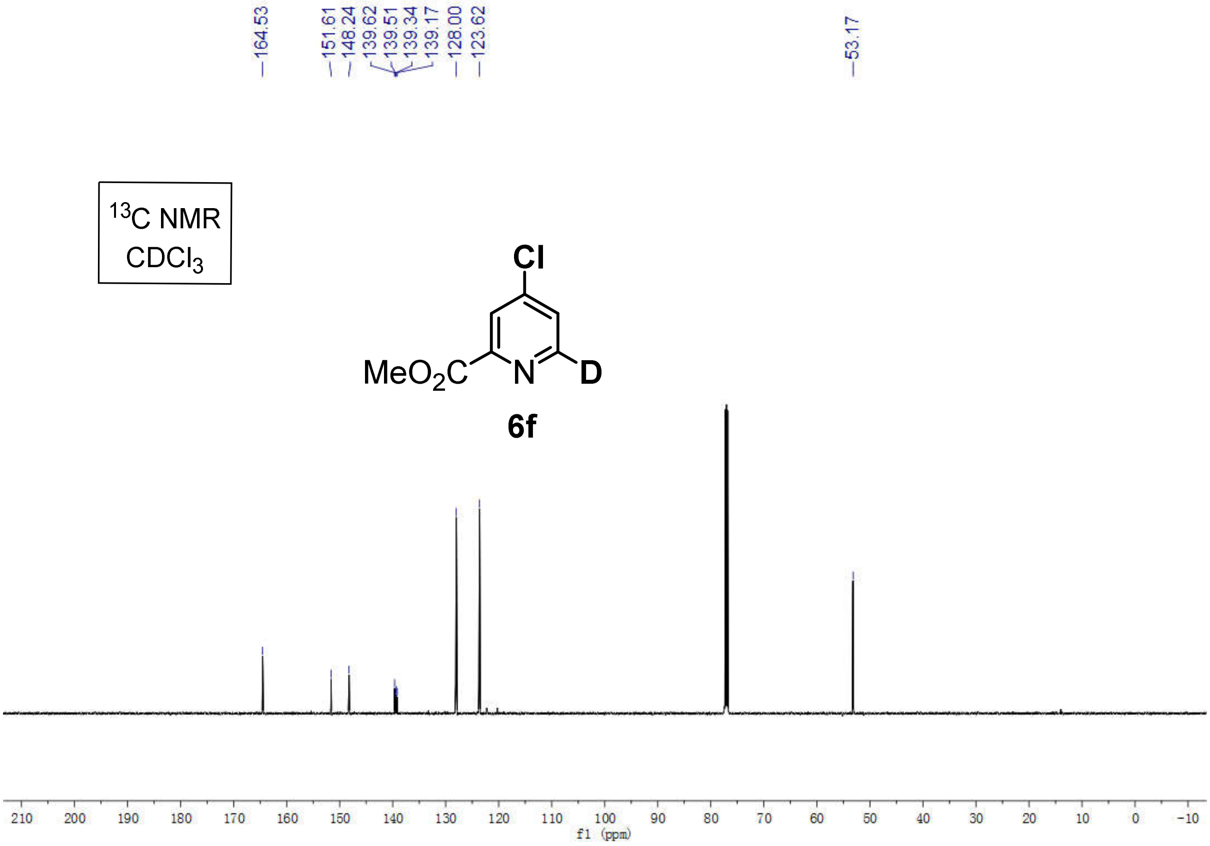


**Supplementary Fig. 95** $^{1}$H NMR, $^{2}$H NMR and $^{13}$C NMR spectra of the compound **6f**.

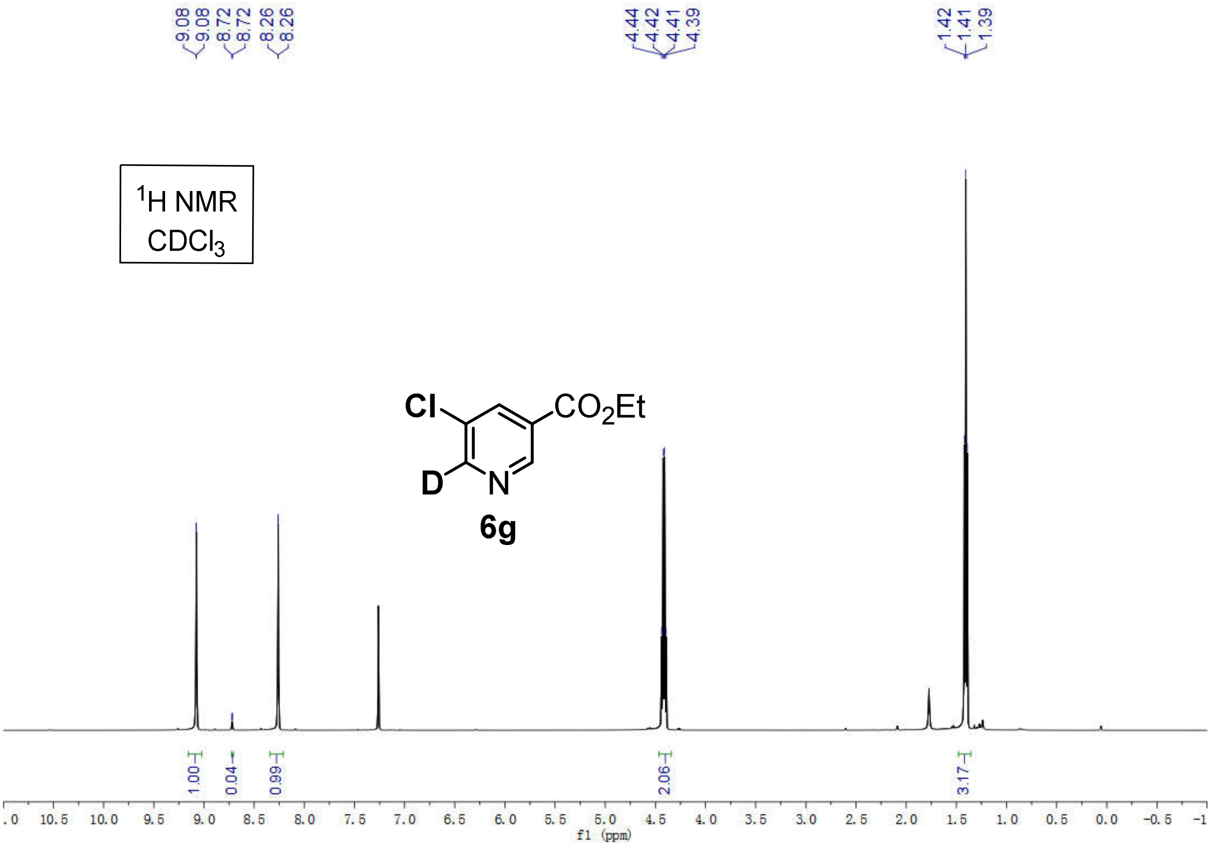
9.08
9.08
8.72
8.72
8.26
8.26
4.44
4.42
4.41
4.39
1.42
1.41
1.39
$^1$H NMR
$CDCl_3$
Cl
$CO_2Et$
D
N
6g
1.00
0.04
0.99
2.06
3.17
.0 10.5 10.0 9.5 9.0 8.5 8.0 7.5 7.0 6.5 6.0 5.5 5.0 4.5 4.0 3.5 3.0 2.5 2.0 1.5 1.0 0.5 0.0 −0.5 −1
f1 (ppm)

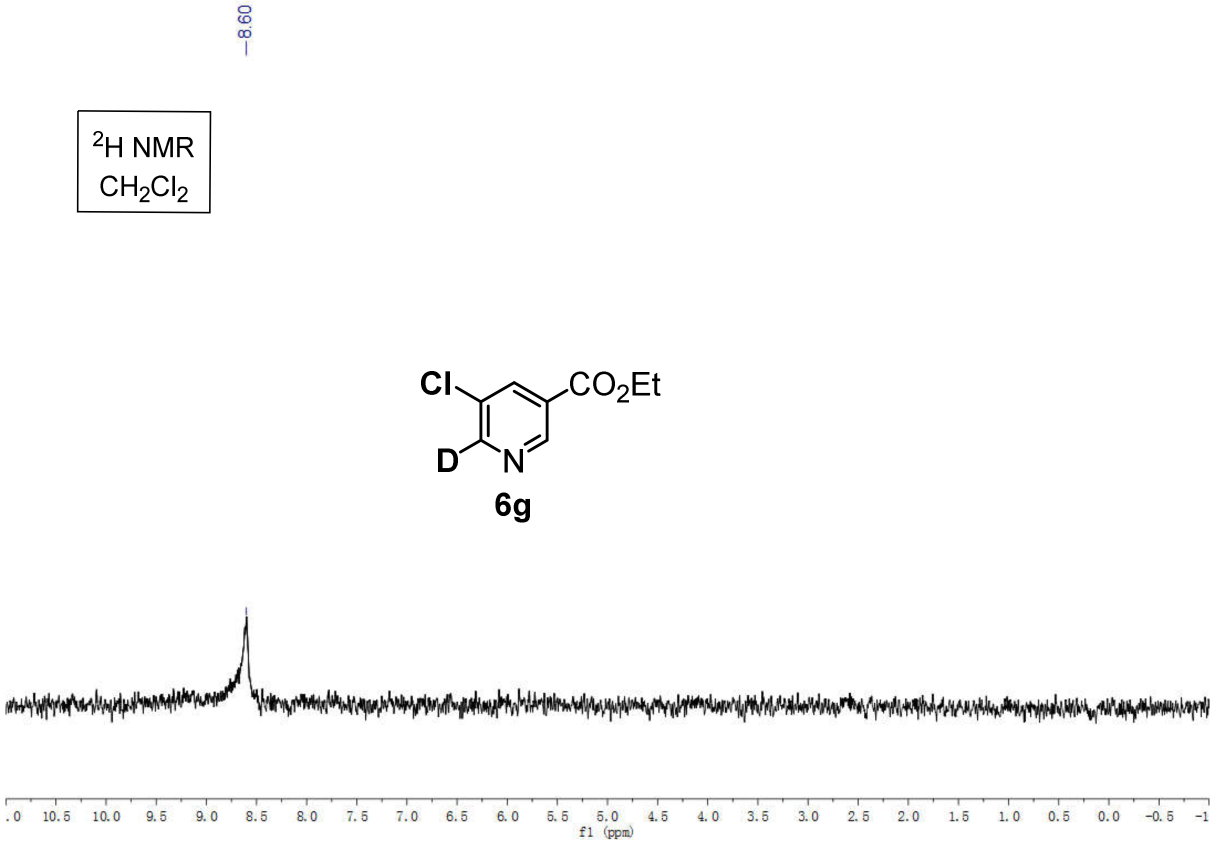
8.60
$^2$H NMR
$CH_2Cl_2$
Cl
$CO_2Et$
D
N
6g
.0 10.5 10.0 9.5 9.0 8.5 8.0 7.5 7.0 6.5 6.0 5.5 5.0 4.5 4.0 3.5 3.0 2.5 2.0 1.5 1.0 0.5 0.0 −0.5 −1
f1 (ppm)

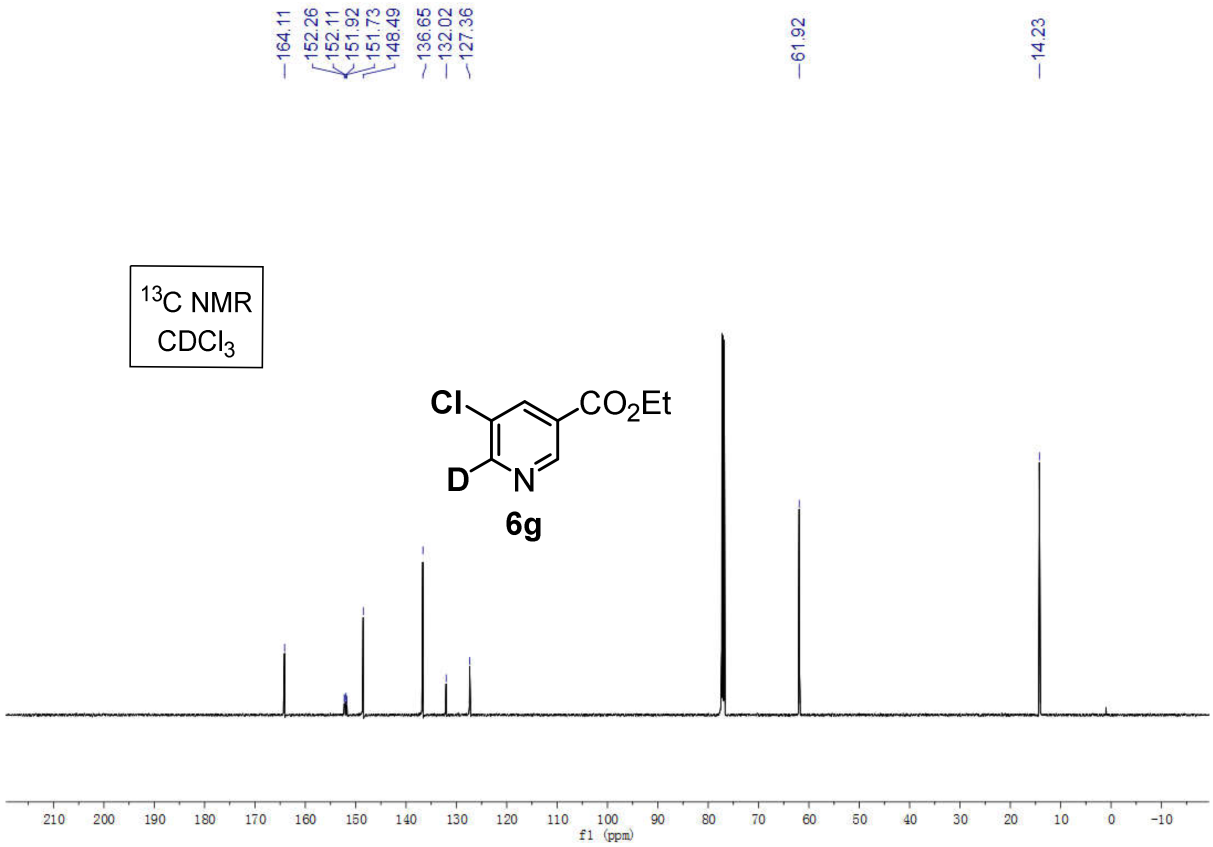


**Supplementary Fig. 96** $^{1}$H NMR, $^{2}$H NMR and $^{13}$C NMR spectra of the compound **6g**.

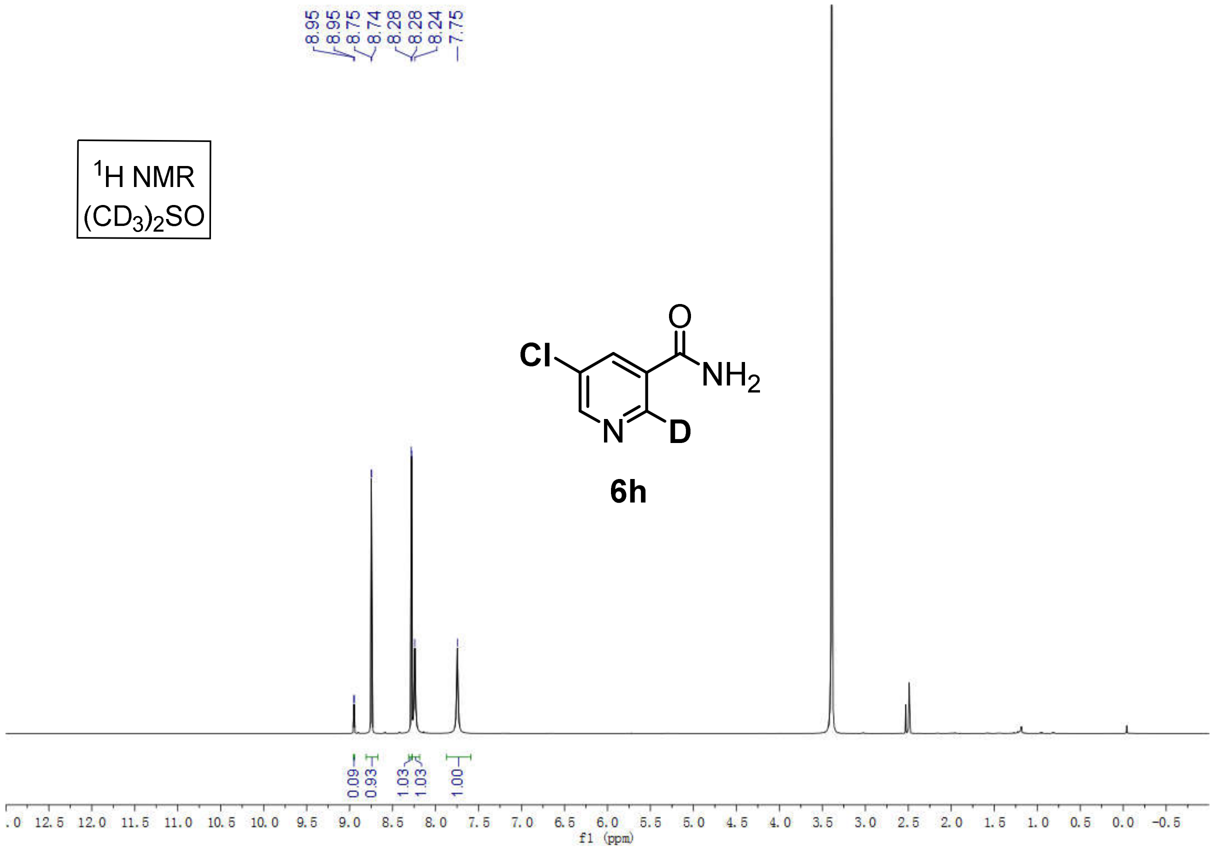

8.95
8.95
8.75
8.74
8.28
8.28
8.24
7.75
$^1$H NMR
$(CD_3)_2SO$
Cl
O
NH2
N
D
6h
0.09
0.93
1.03
1.03
1.00
f1 (ppm)


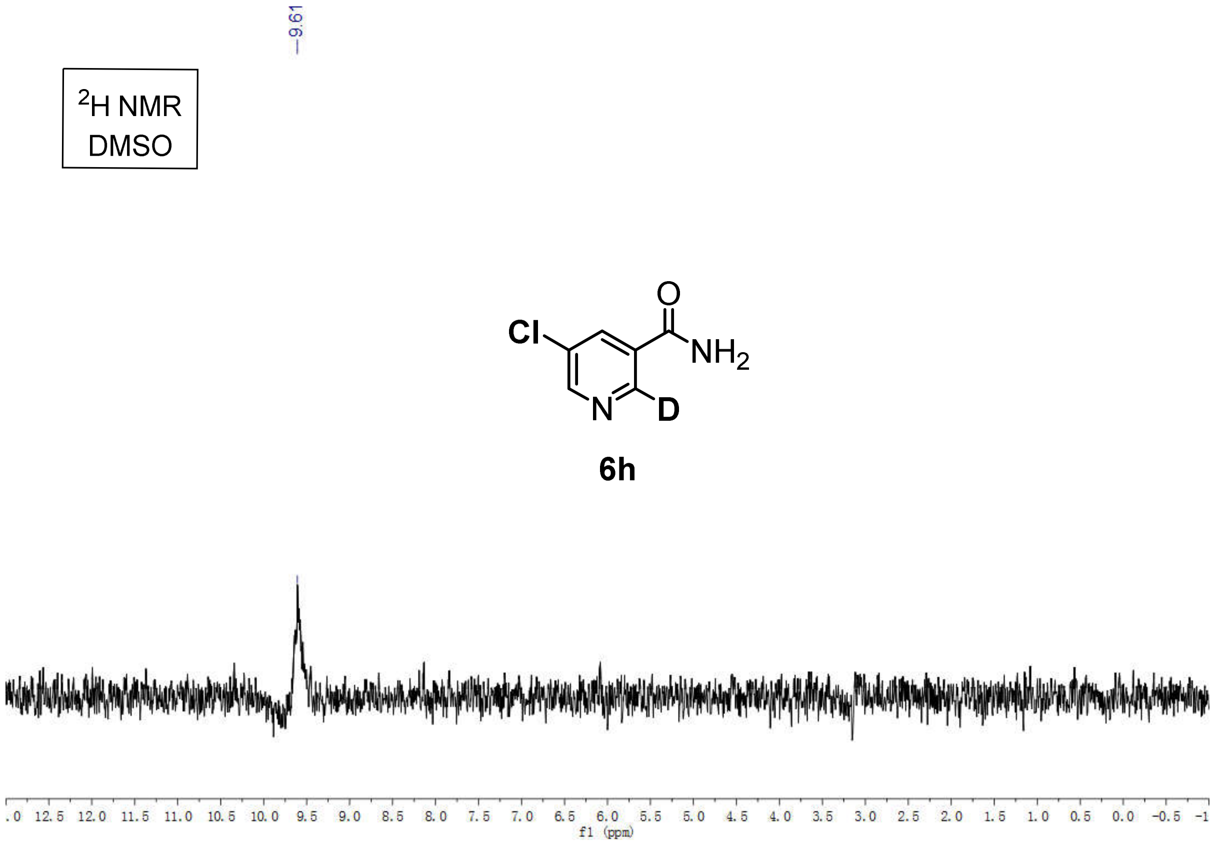

9.61
$^2$H NMR
DMSO
Cl
O
NH2
N
D
6h
f1 (ppm)

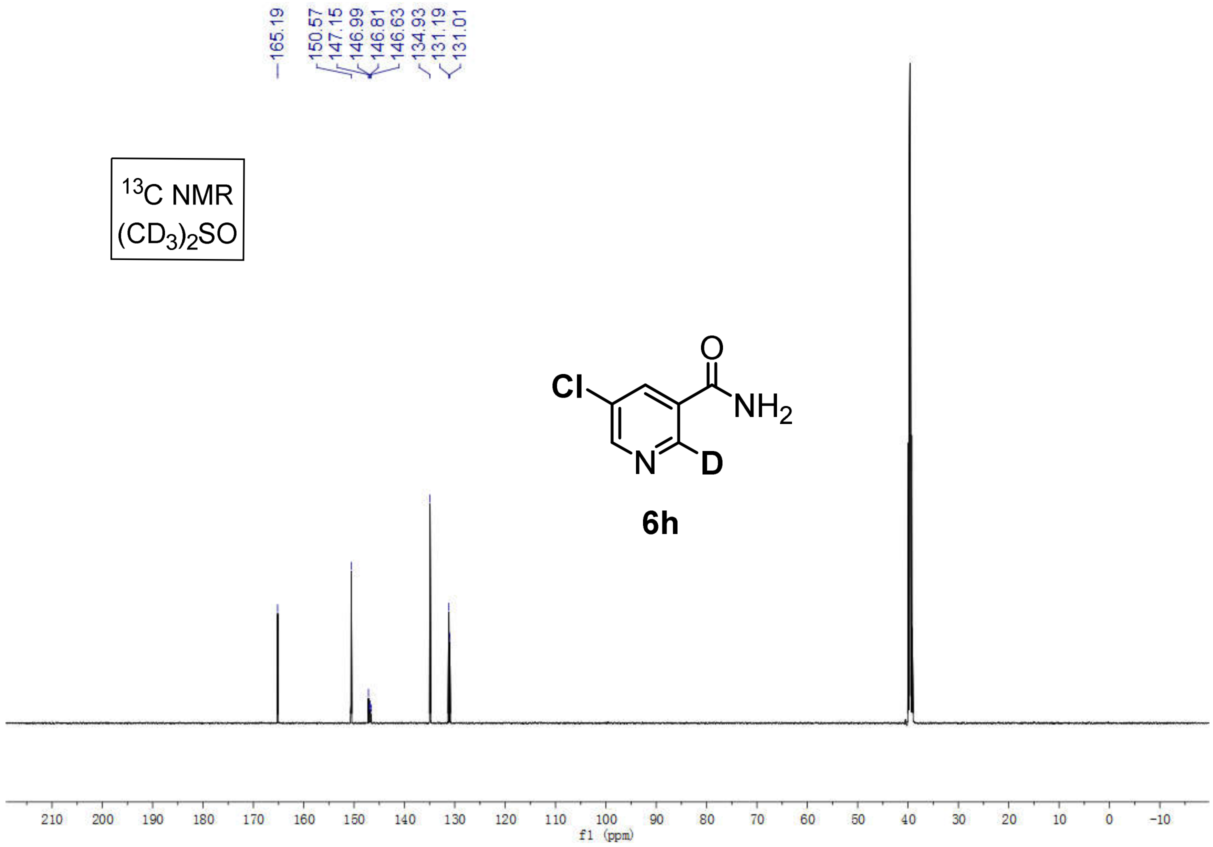


**Supplementary Fig. 97** $^{1}$H NMR, $^{2}$H NMR and $^{13}$C NMR spectra of the compound **6h**.

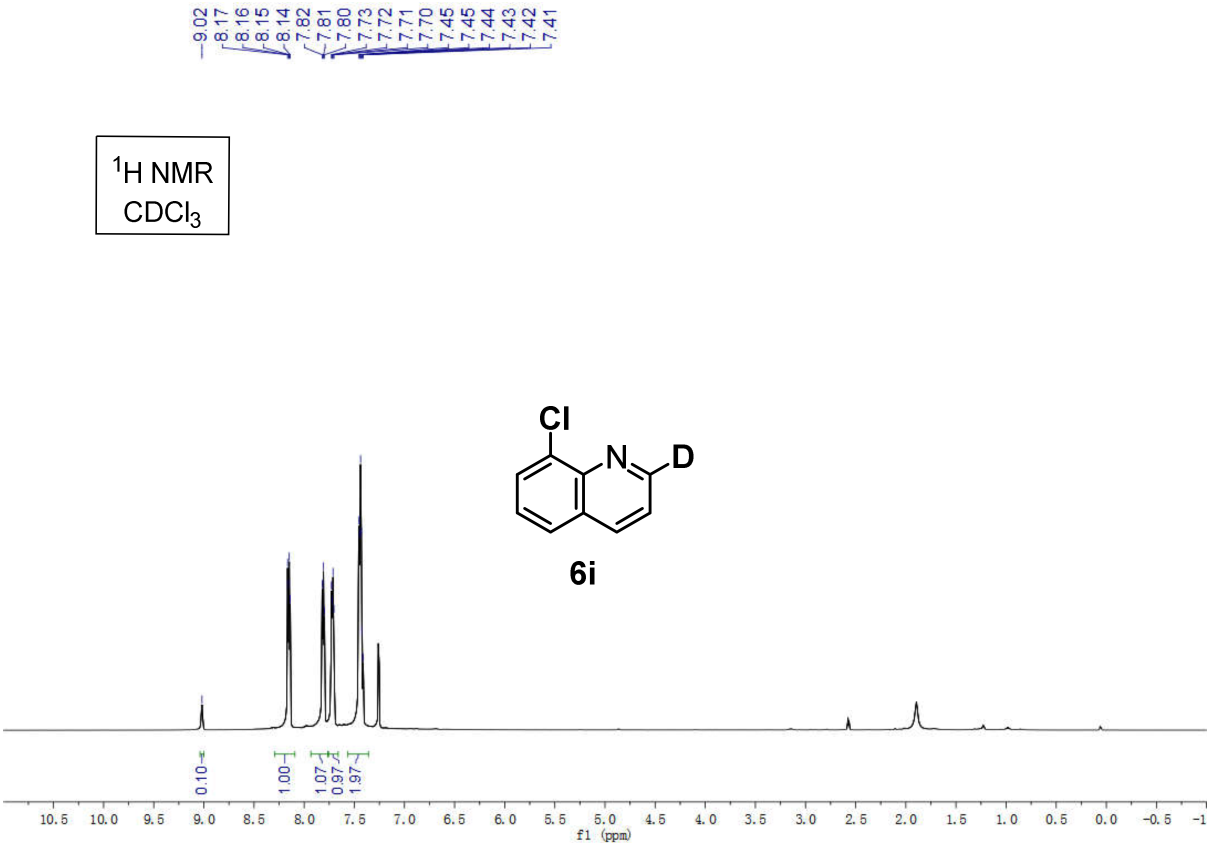
9.02
8.17
8.16
8.15
8.14
7.82
7.81
7.80
7.73
7.72
7.71
7.70
7.45
7.45
7.44
7.43
7.42
7.41
$^1$H NMR
$CDCl_3$
Cl
N
D
6i
0.10
1.00
1.07
0.97
1.97
f1 (ppm)

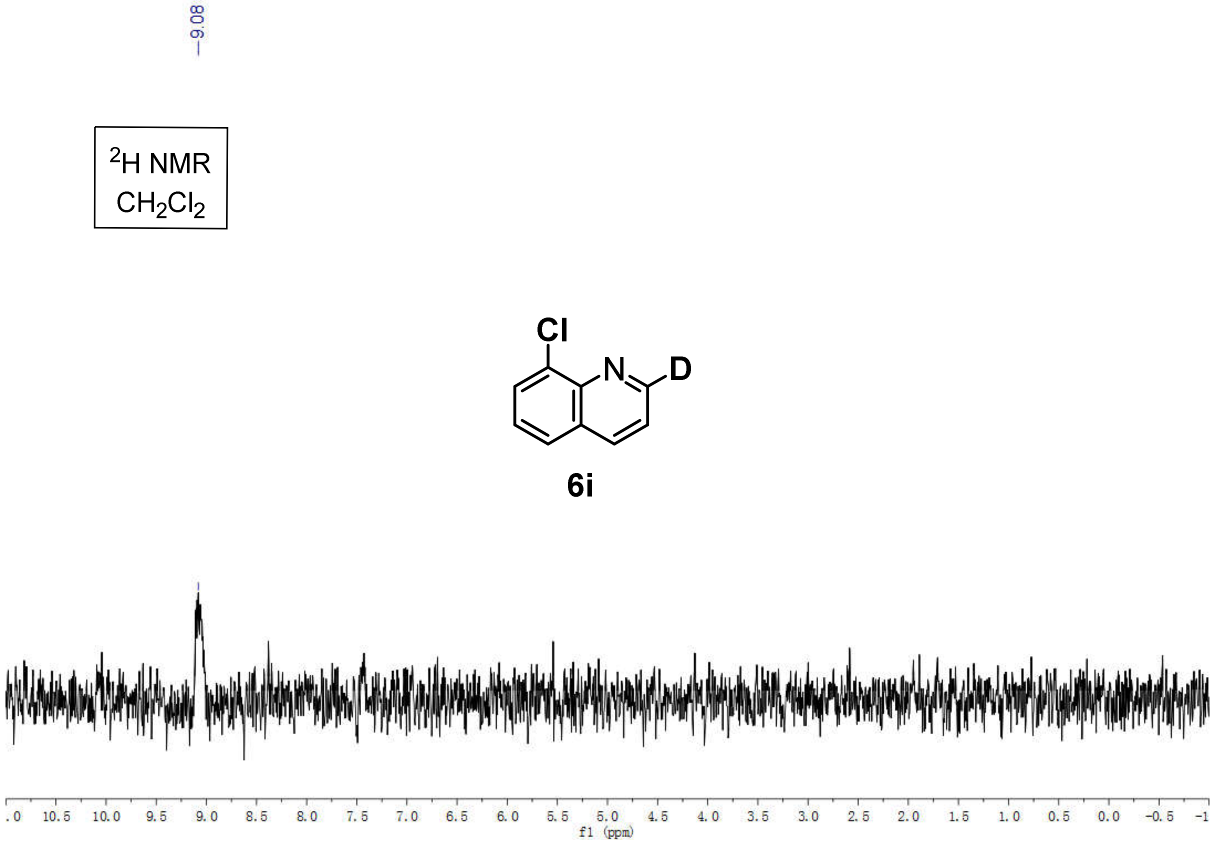
9.08
$^2$H NMR
$CH_2Cl_2$
Cl
N
D
6i
f1 (ppm)

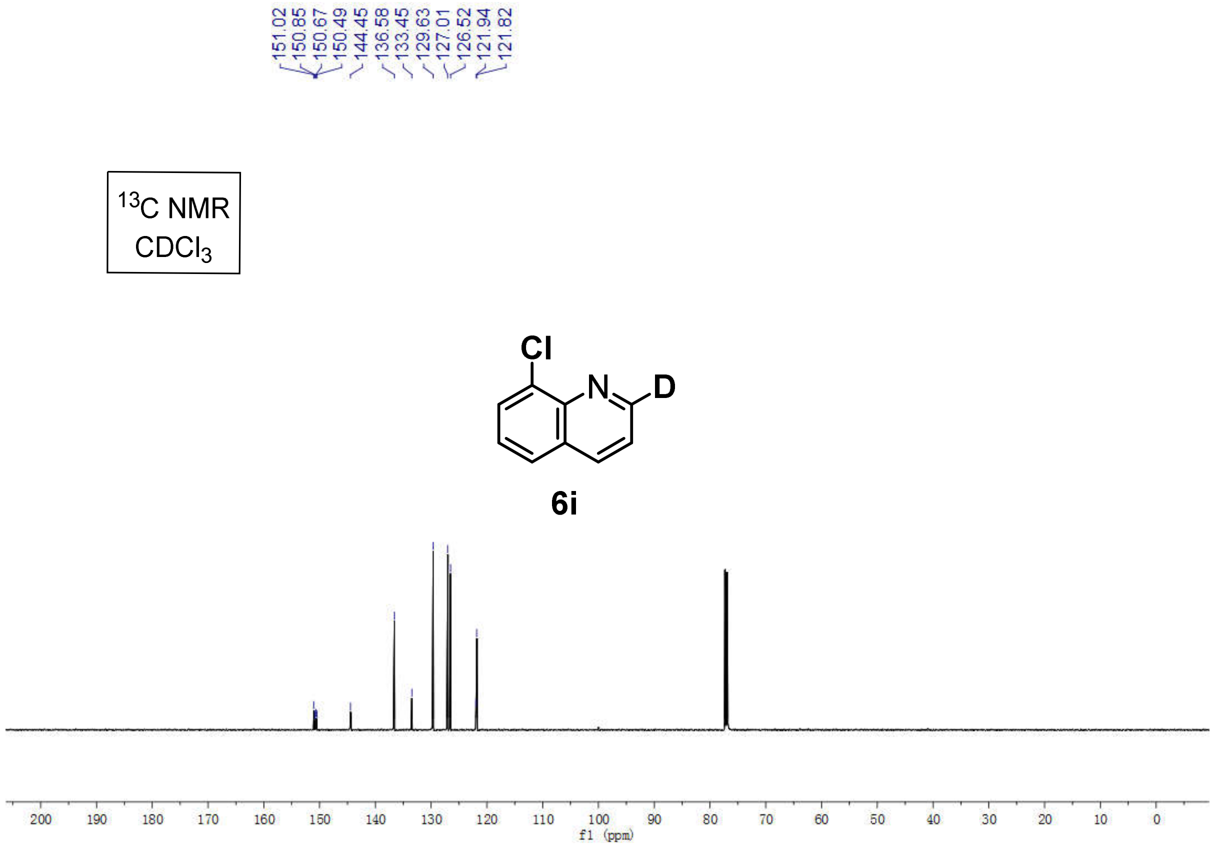


**Supplementary Fig. 98** $^{1}$H NMR, $^{2}$H NMR and $^{13}$C NMR spectra of the compound **6i**.

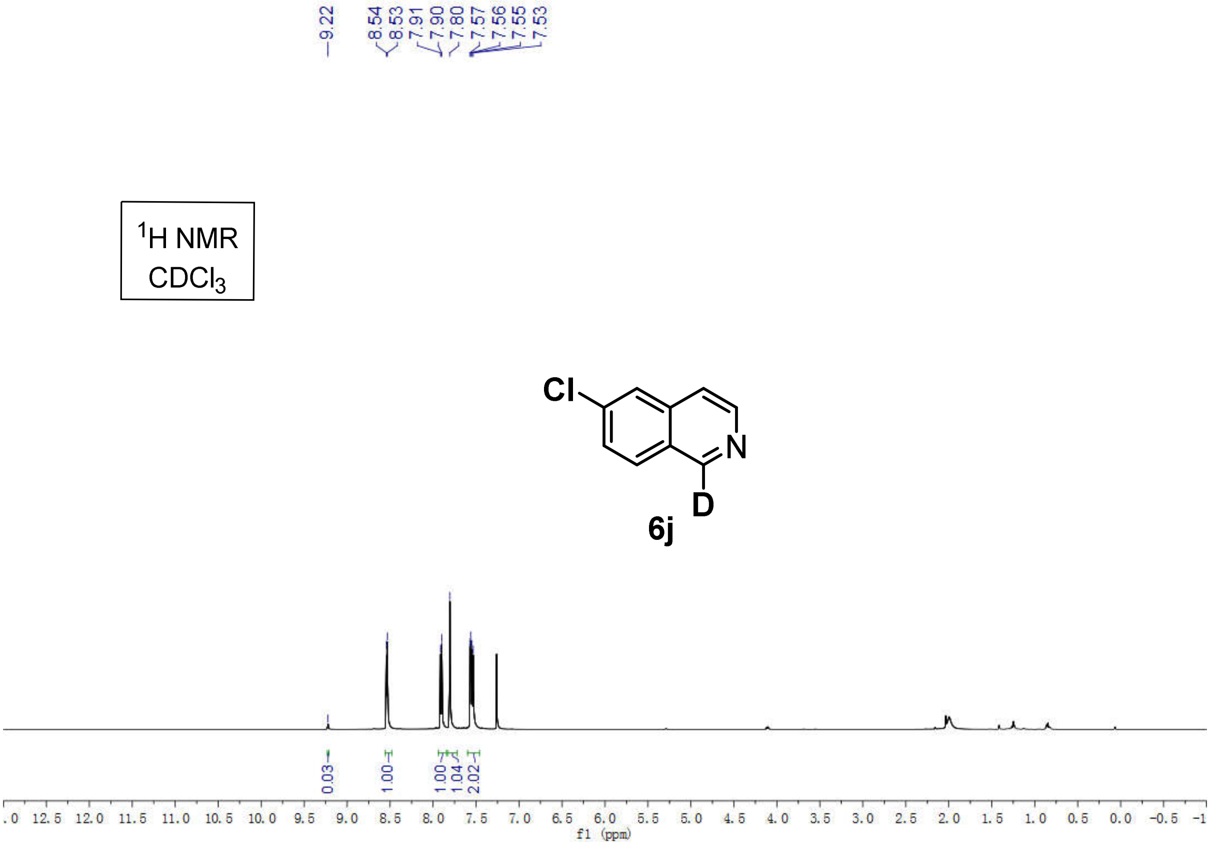
9.22
8.54
8.53
7.91
7.90
7.80
7.57
7.56
7.55
7.53
1H NMR
CDCl3
Cl
N
D
6j
0.03
1.00
1.00
1.04
2.02
f1 (ppm)

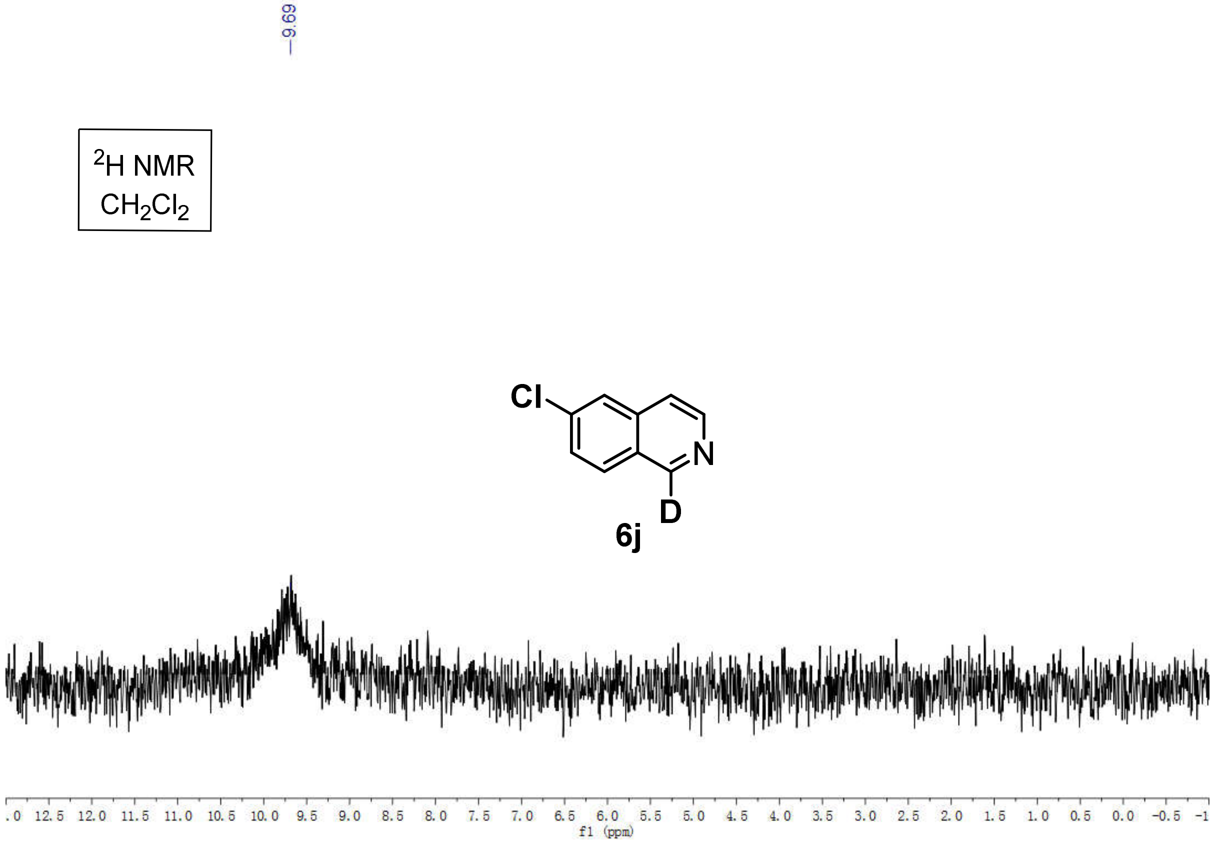
9.69
2H NMR
CH2Cl2
Cl
N
D
6j
f1 (ppm)

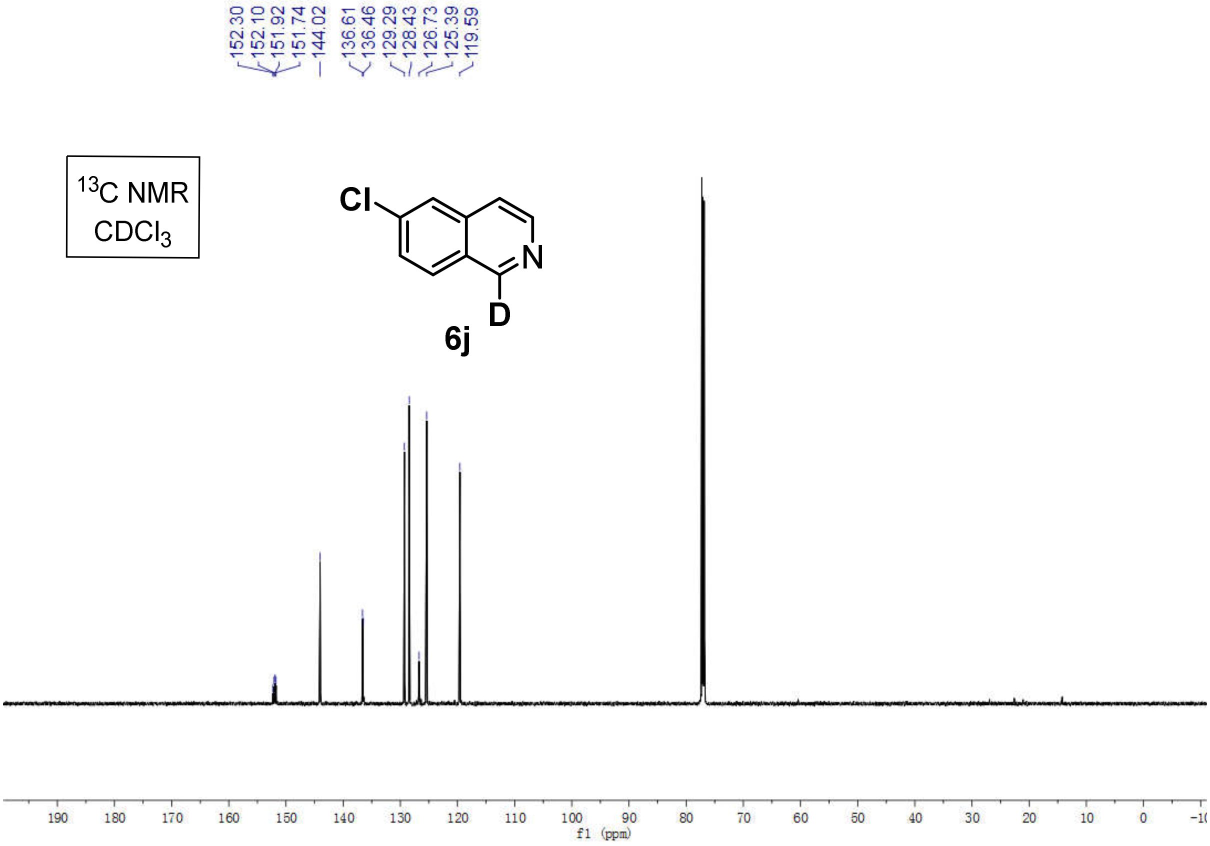


**Supplementary Fig. 99** $^{1}$H NMR, $^{2}$H NMR and $^{13}$C NMR spectra of the compound **6j**.

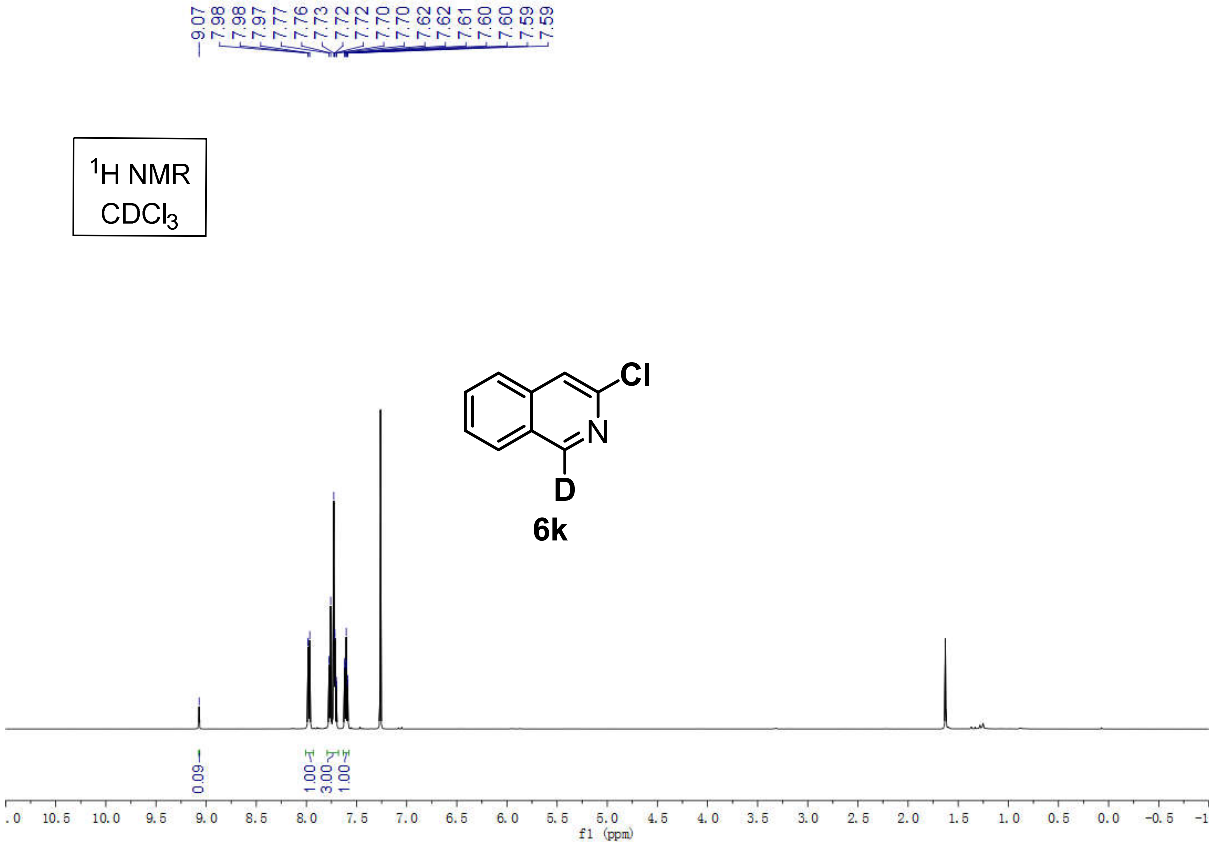
$^1$H NMR
$CDCl_3$
Cl
N
D
6k
f1 (ppm)

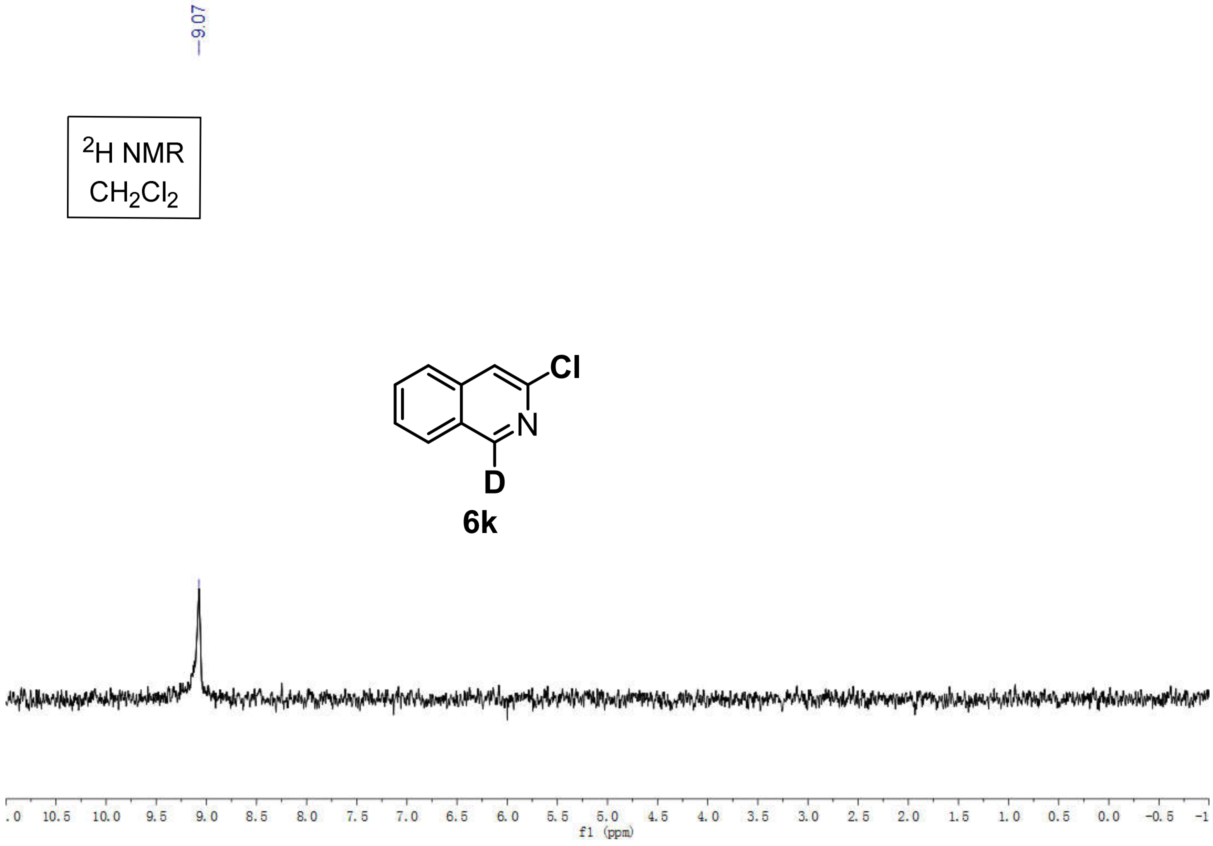
$^2$H NMR
$CH_2Cl_2$
Cl
N
D
6k
f1 (ppm)

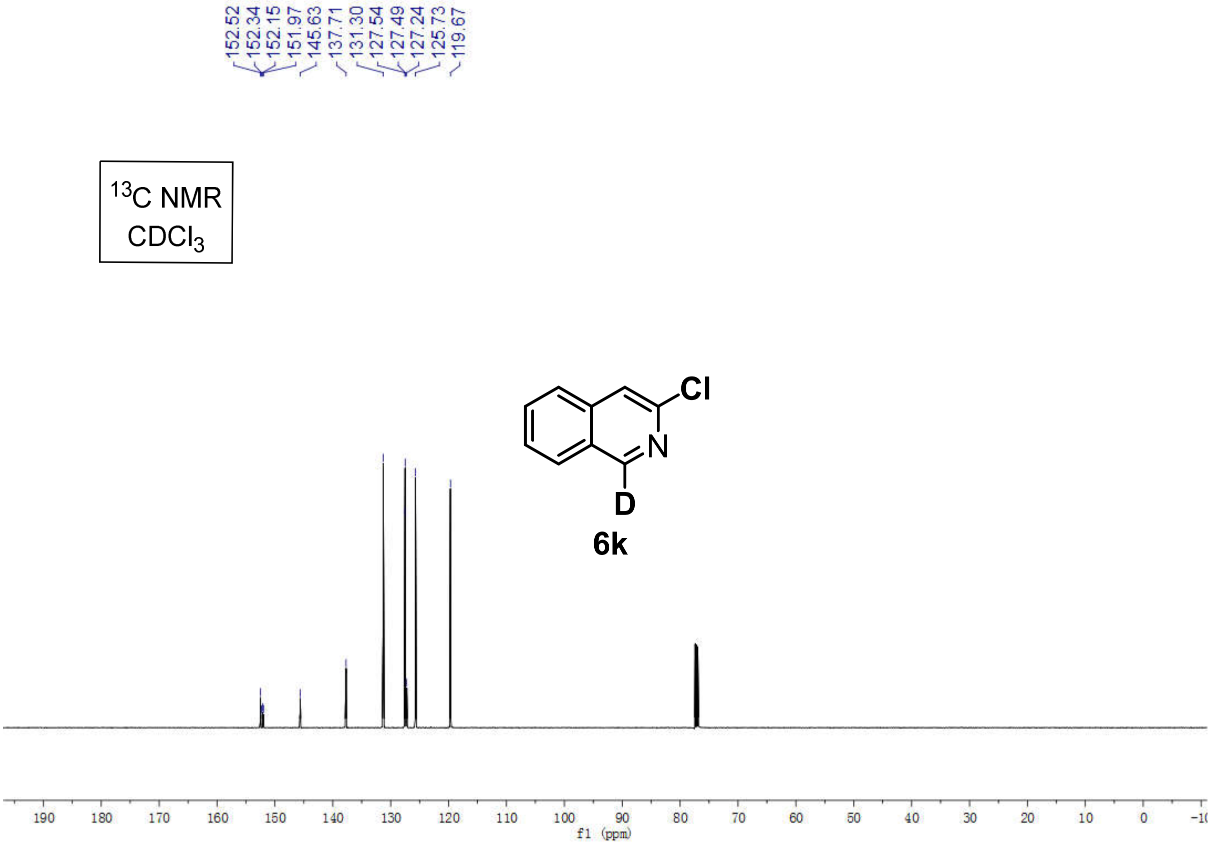


**Supplementary Fig. 100** $^1$H NMR, $^2$H NMR and $^{13}$C NMR spectra of the compound **6k**.

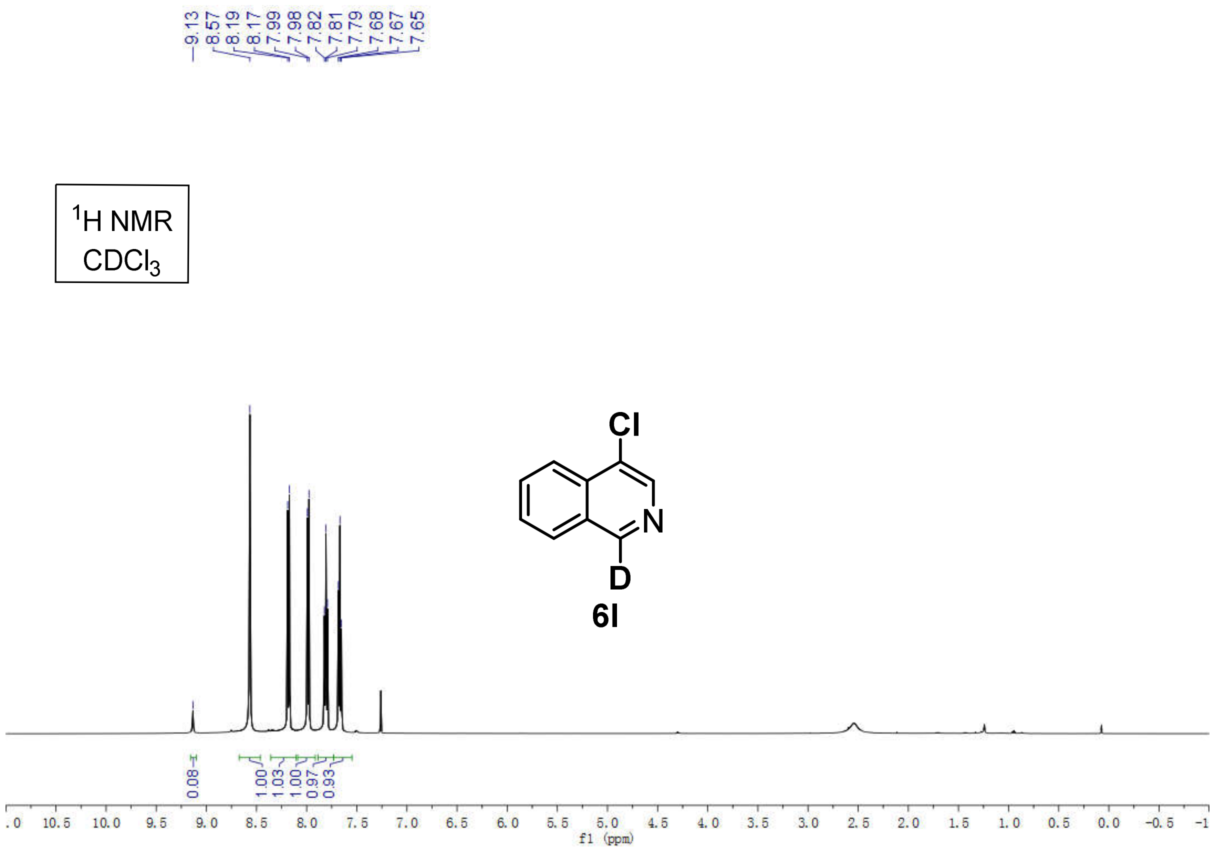

9.13
8.57
8.19
8.17
7.99
7.98
7.82
7.81
7.79
7.68
7.67
7.65
$^1$H NMR
$CDCl_3$
Cl
N
D
6l
0.08
1.00
1.03
1.00
0.97
0.93
f1 (ppm)


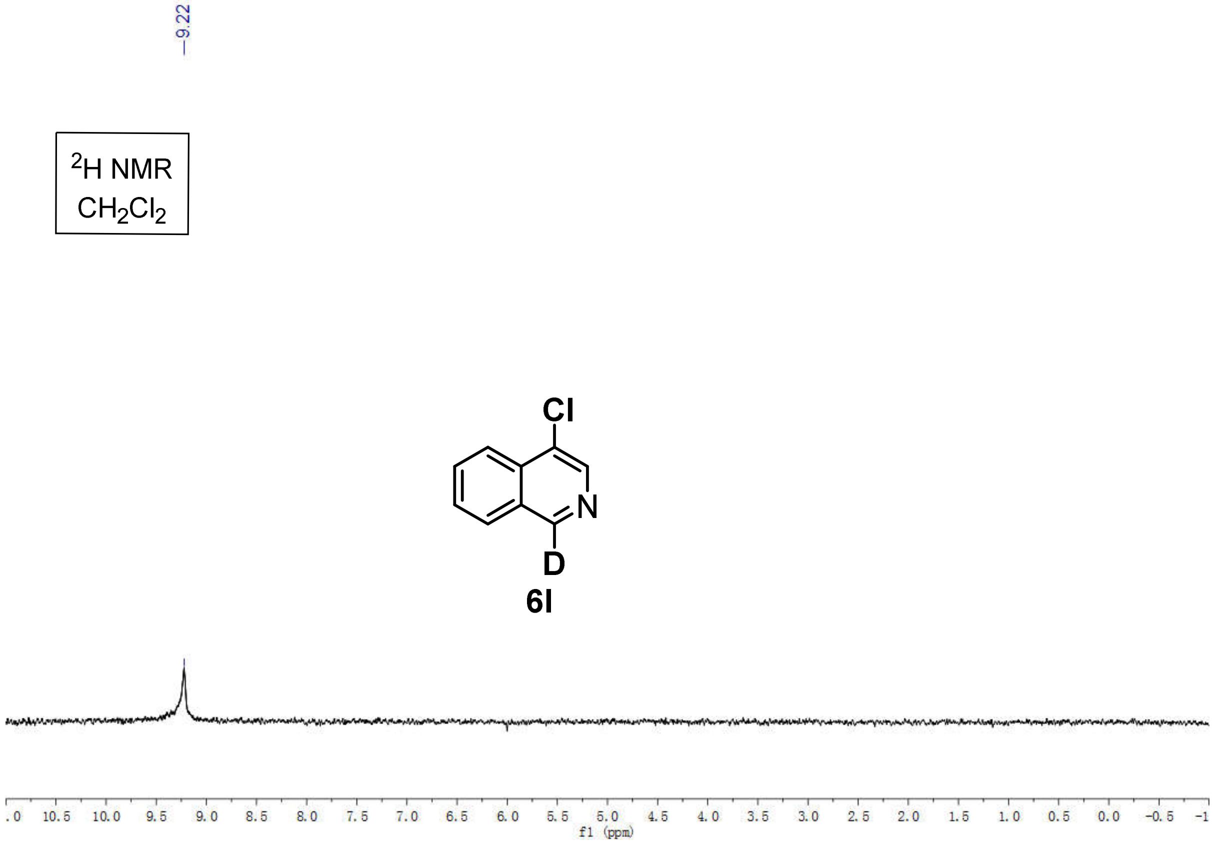

9.22
$^2$H NMR
$CH_2Cl_2$
Cl
N
D
6l
f1 (ppm)

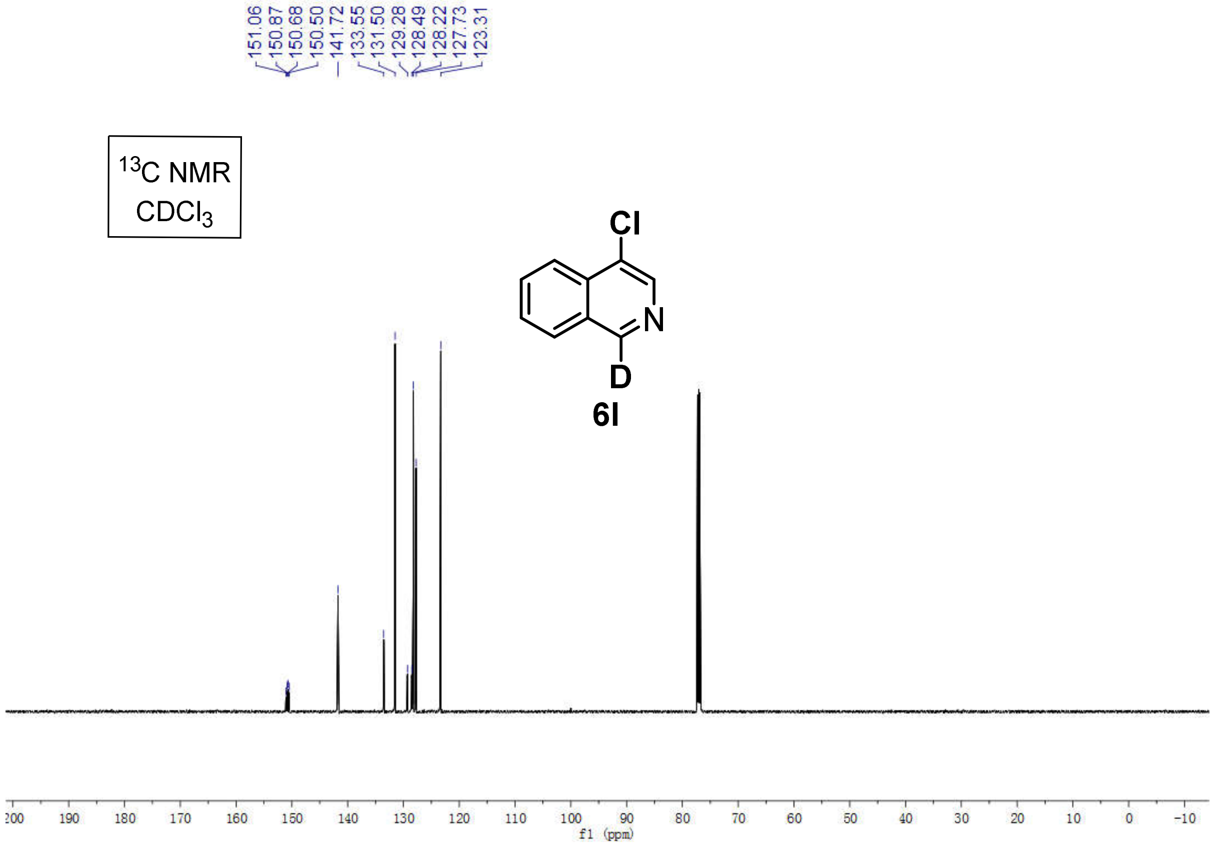


**Supplementary Fig. 101** $^1$H NMR, $^2$H NMR and $^{13}$C NMR spectra of the compound **6l**.

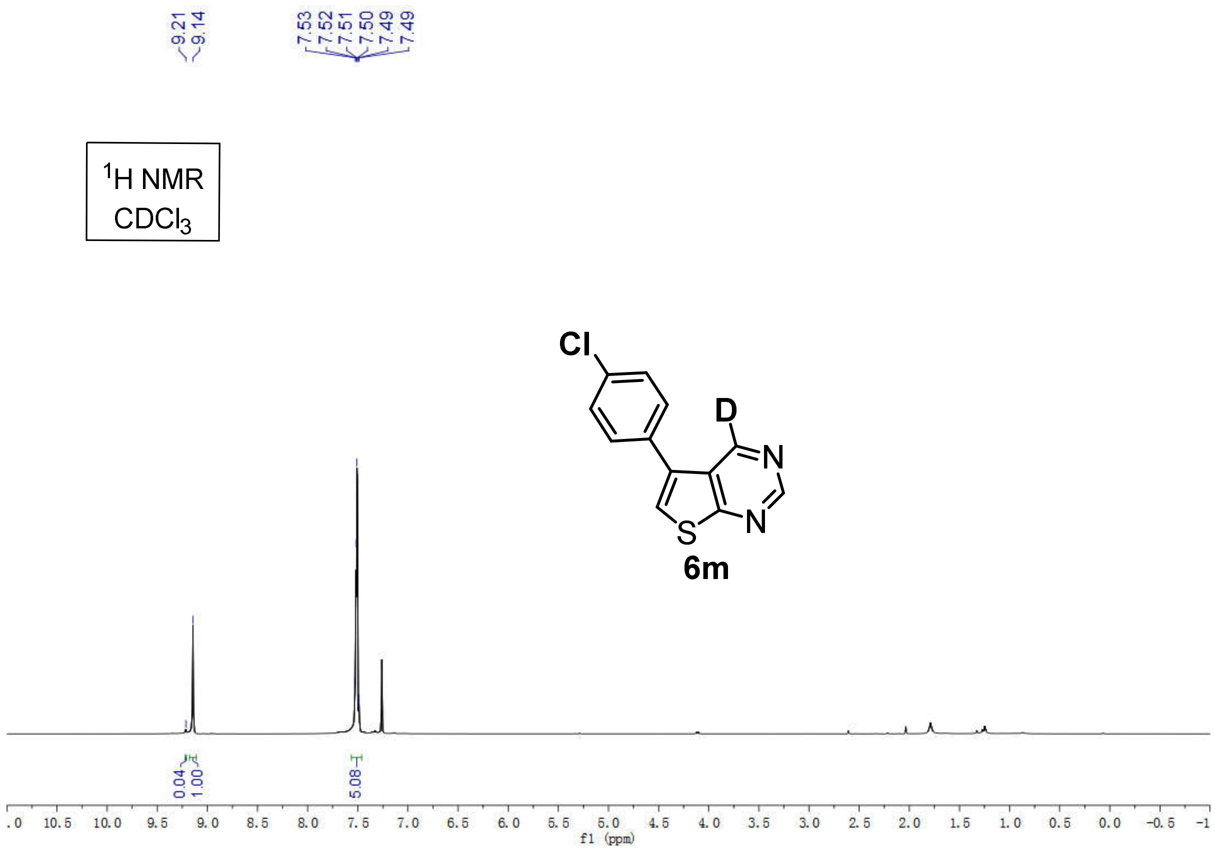
9.21
9.14
7.53
7.52
7.51
7.50
7.49
7.49
$^1$H NMR
$CDCl_3$
Cl
D
N
N
S
6m
0.04
1.00
5.08
f1 (ppm)

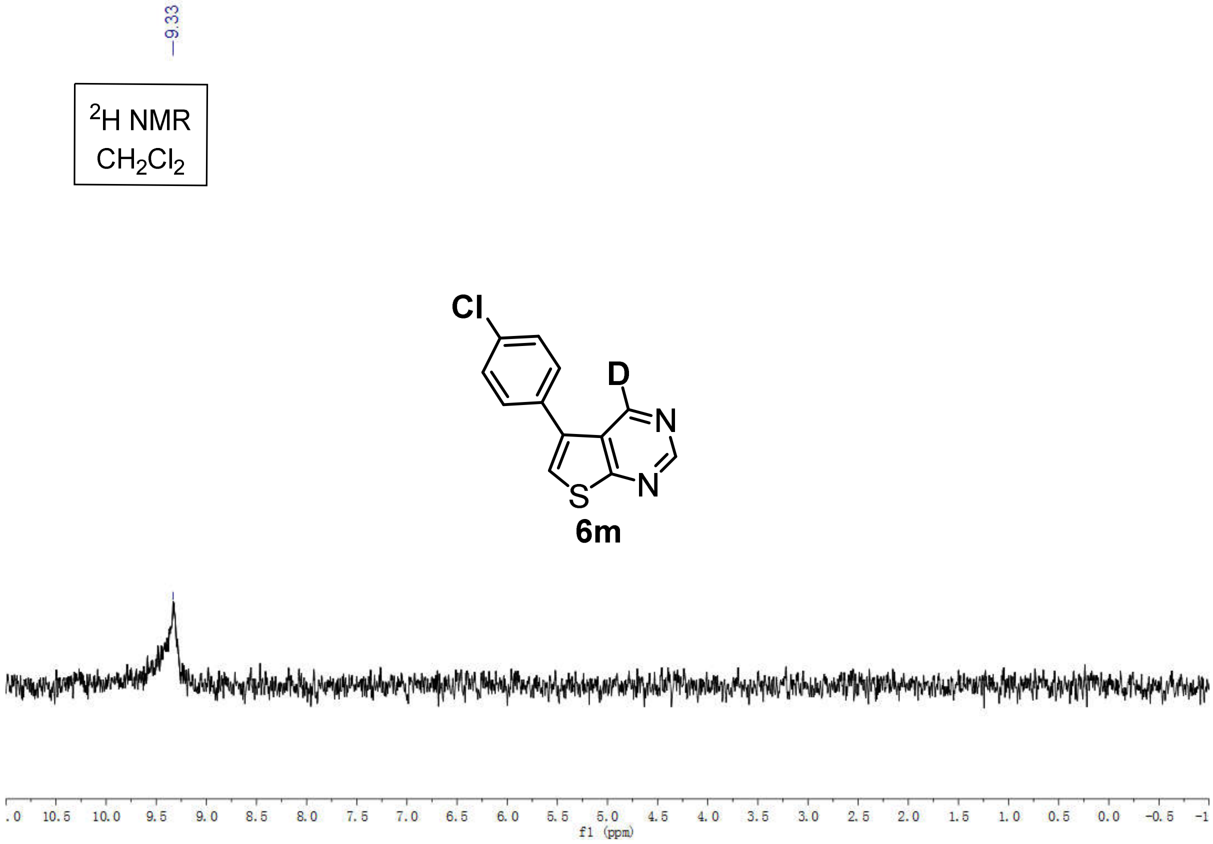
9.33
$^2$H NMR
$CH_2Cl_2$
Cl
D
N
N
S
6m
f1 (ppm)

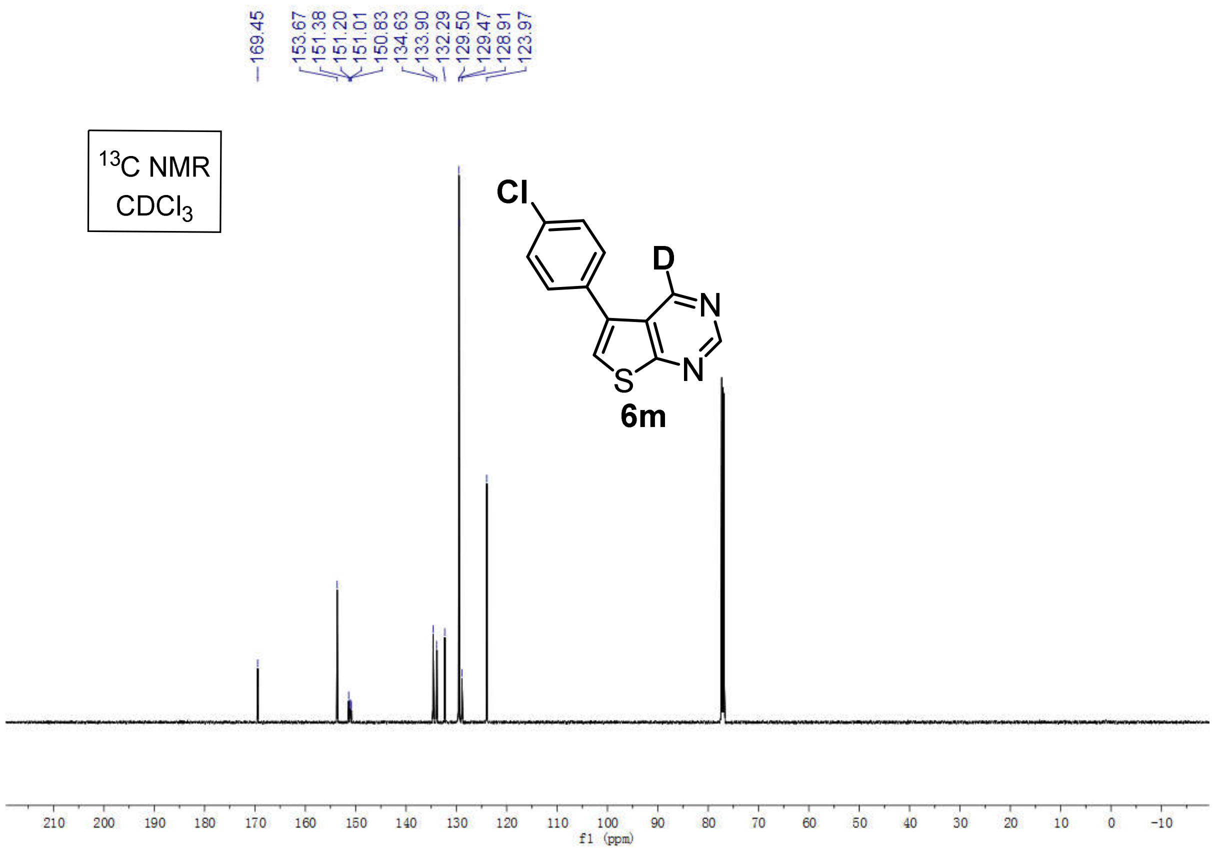


**Supplementary Fig. 102** $^1$H NMR, $^2$H NMR and $^{13}$C NMR spectra of the compound **6m**.

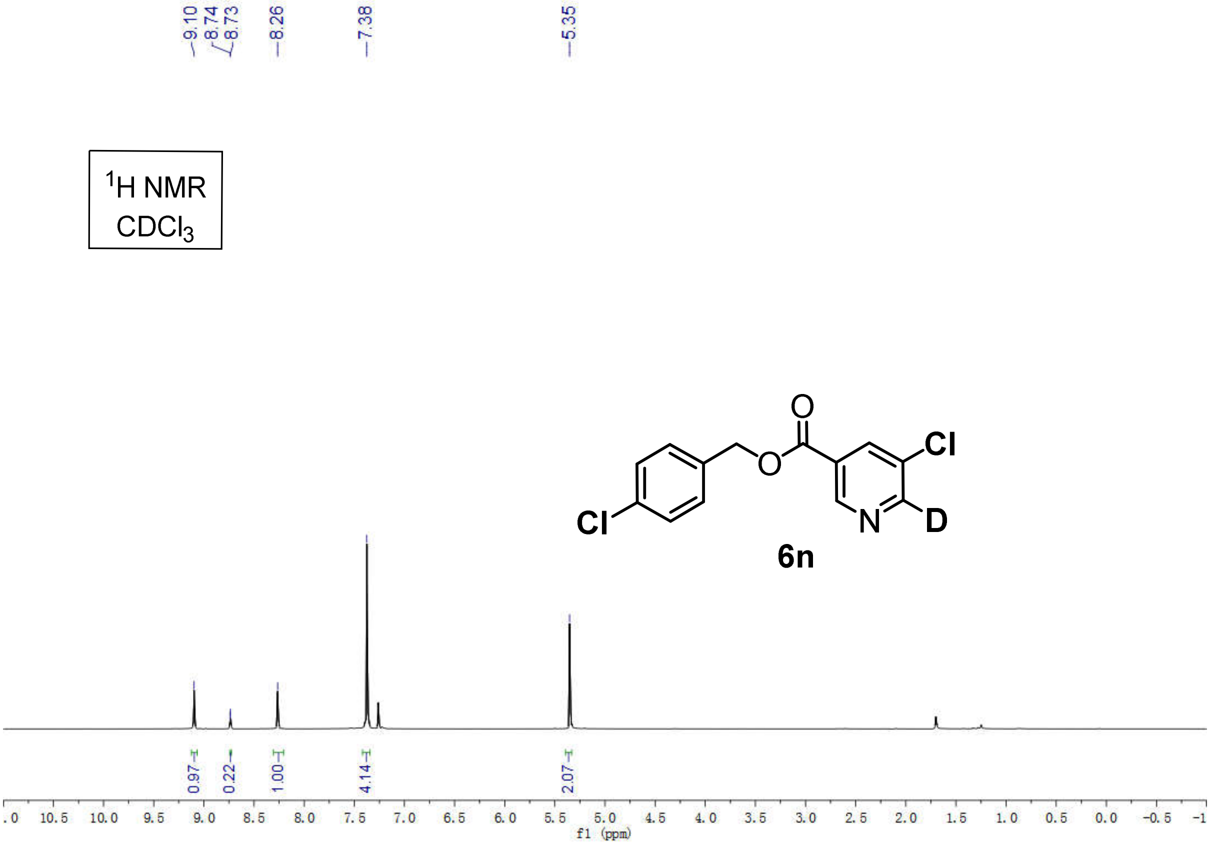

9.10
8.74
8.73
8.26
7.38
5.35
1H NMR
CDCl3
Cl
O
O
Cl
N
D
6n
0.97
0.22
1.00
4.14
2.07
f1 (ppm)


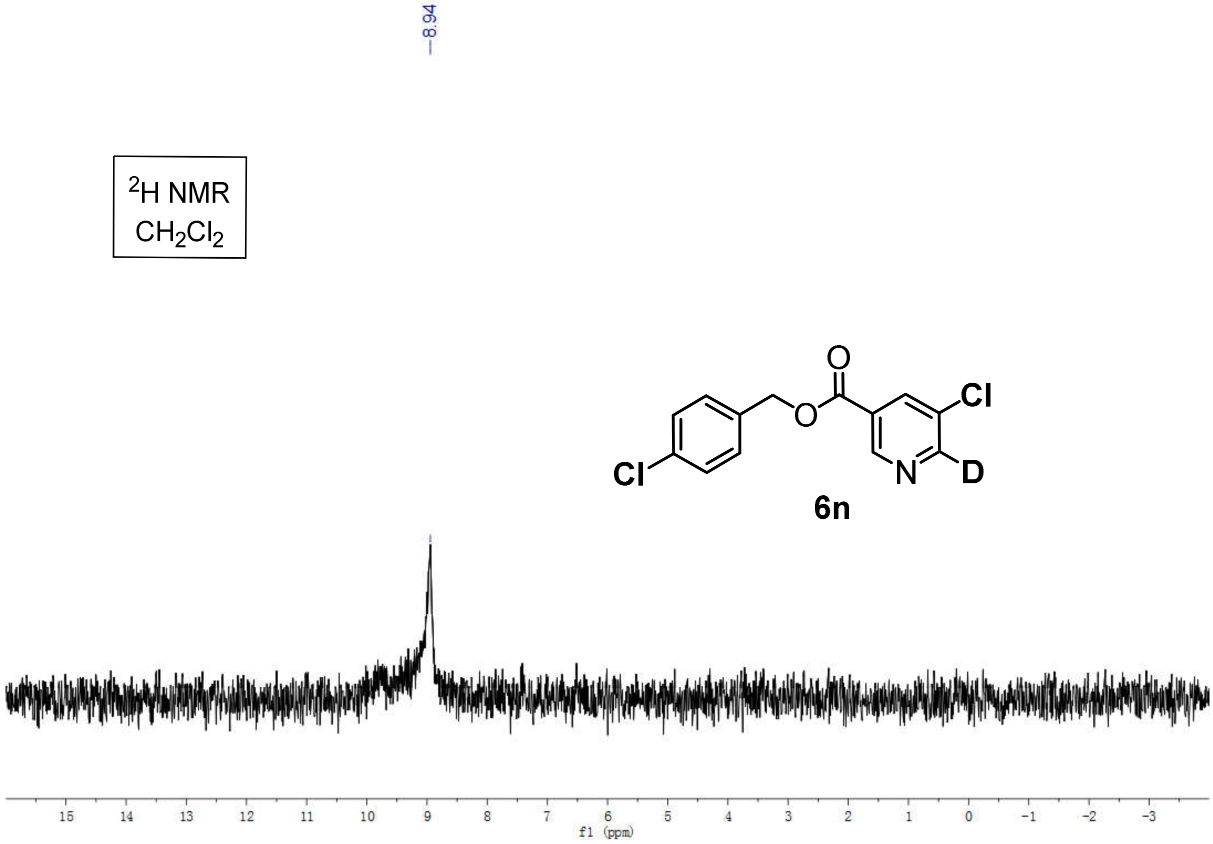

8.94
2H NMR
CH2Cl2
Cl
O
O
Cl
N
D
6n
f1 (ppm)

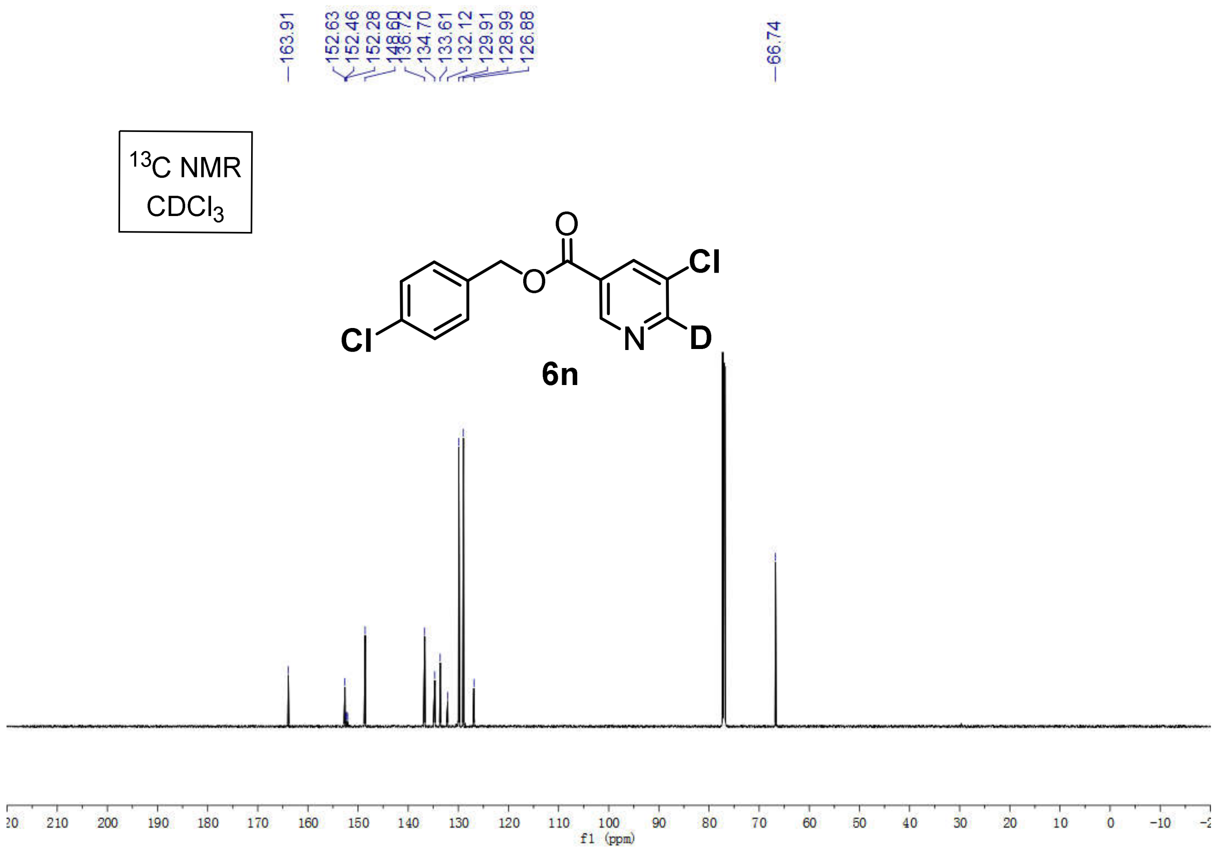


**Supplementary Fig. 103** $^{1}H$ NMR, $^{2}H$ NMR and $^{13}C$ NMR spectra of the compound **6n**.

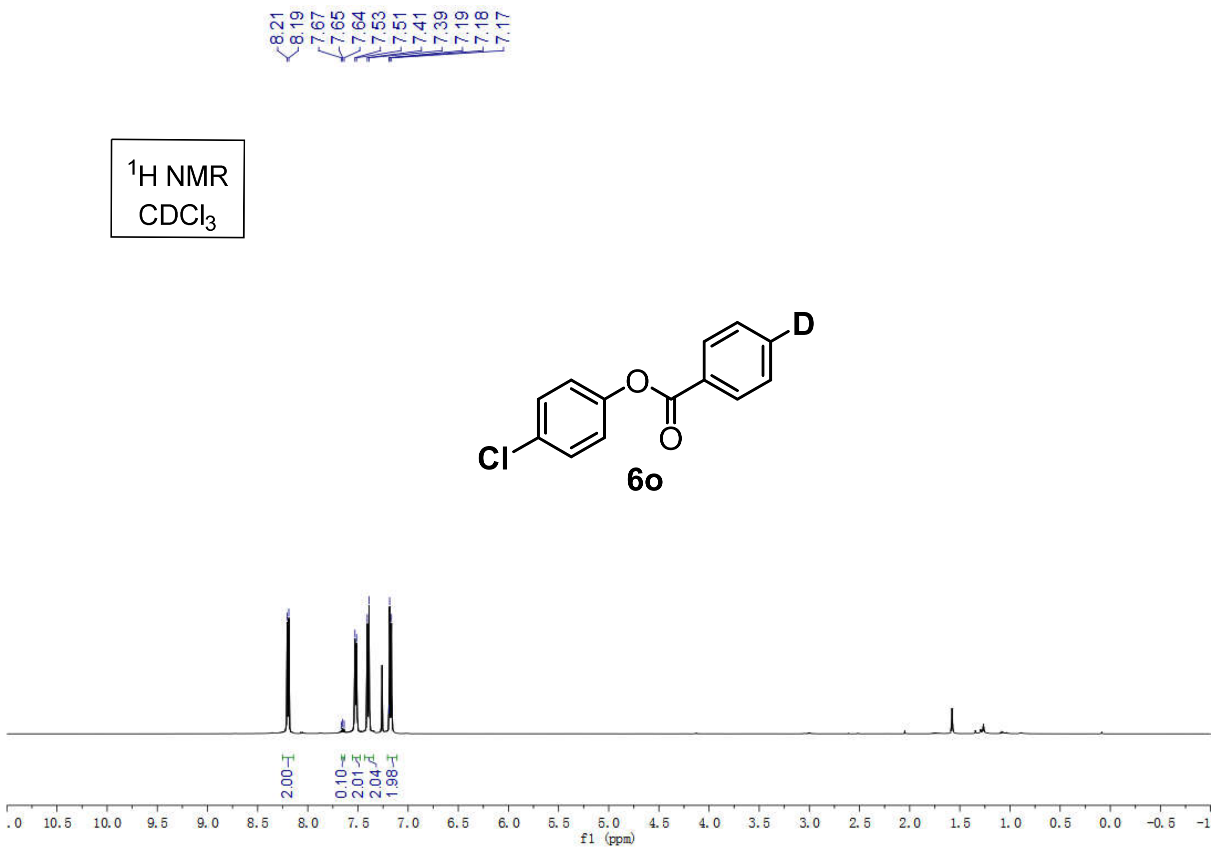
8.21
8.19
7.67
7.65
7.64
7.53
7.51
7.41
7.39
7.19
7.18
7.17
$^1$H NMR
$CDCl_3$
D
O
O
Cl
6o
2.00
0.10
2.01
2.04
1.98
f1 (ppm)

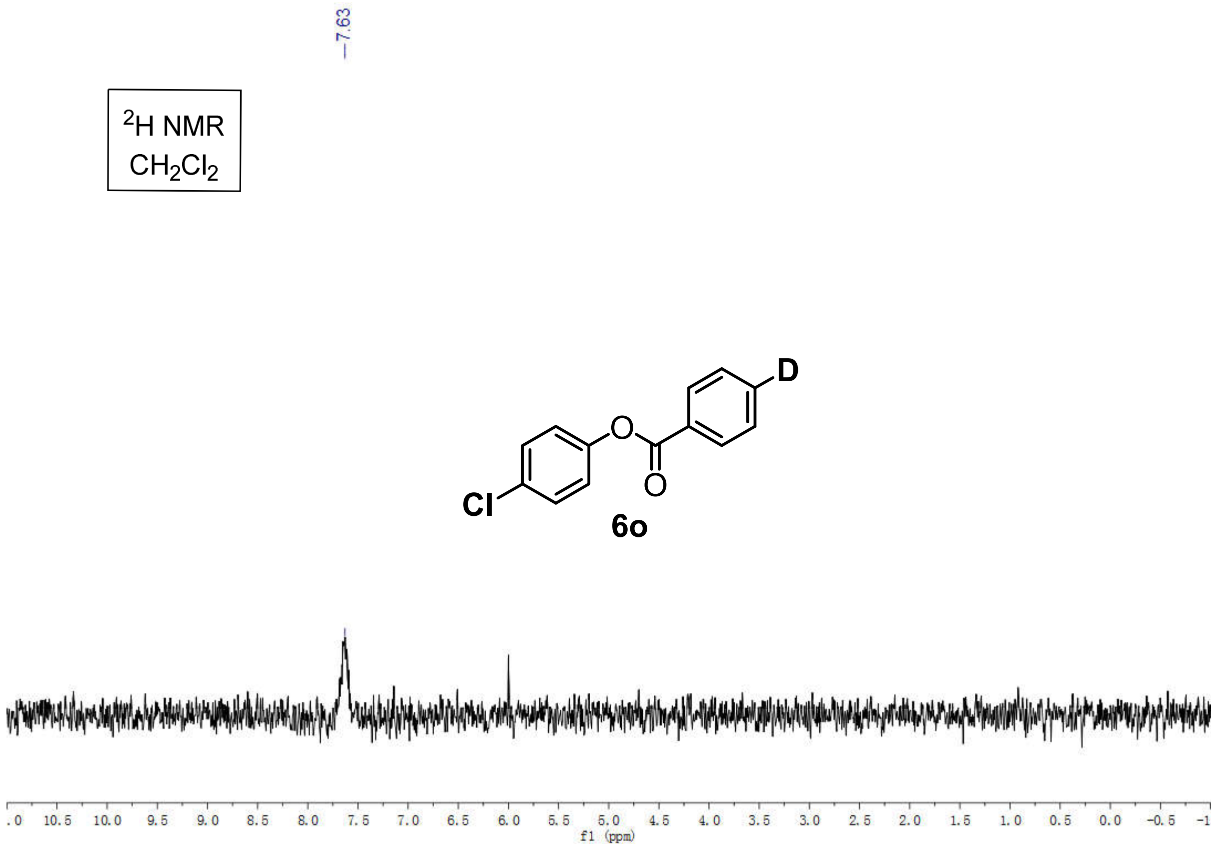
7.63
$^2$H NMR
$CH_2Cl_2$
D
O
O
Cl
6o
f1 (ppm)

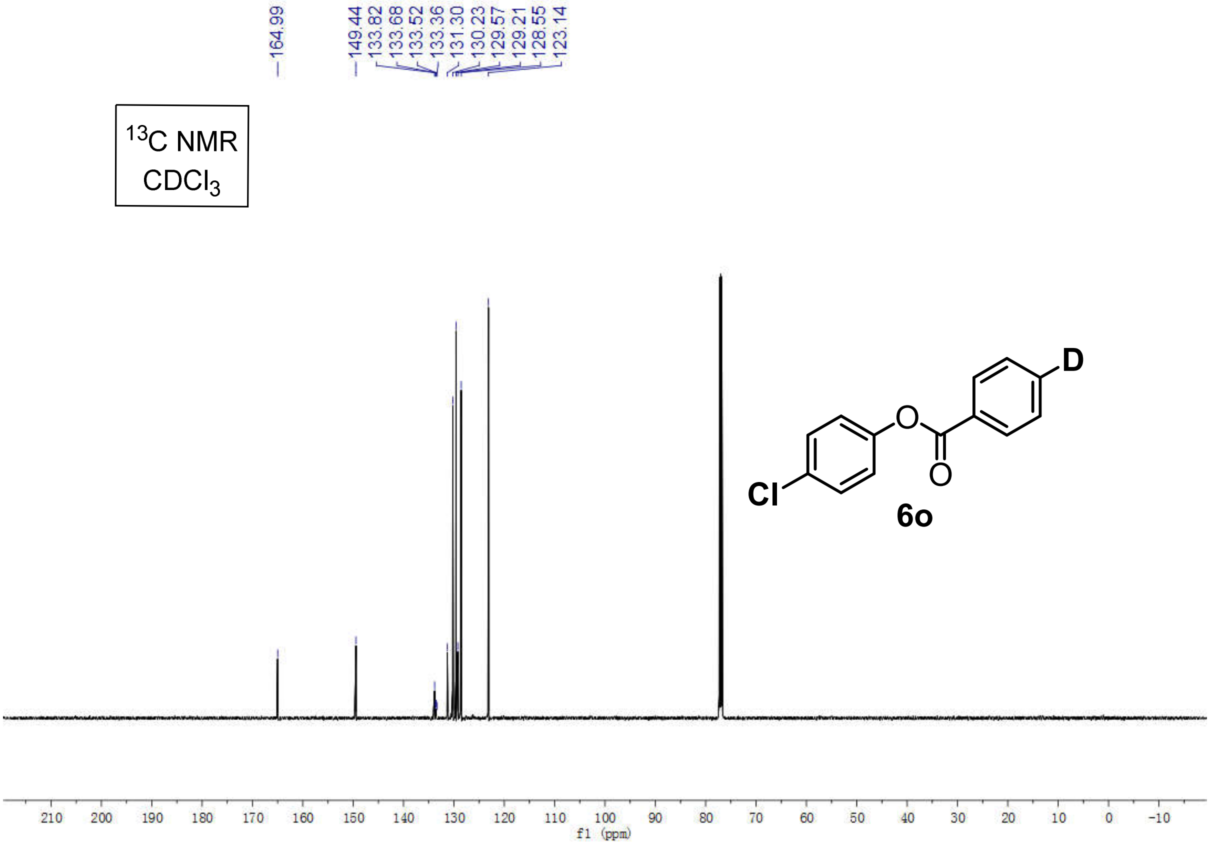


**Supplementary Fig. 104** $^{1}$H NMR, $^{2}$H NMR and $^{13}$C NMR spectra of the compound **6o**.

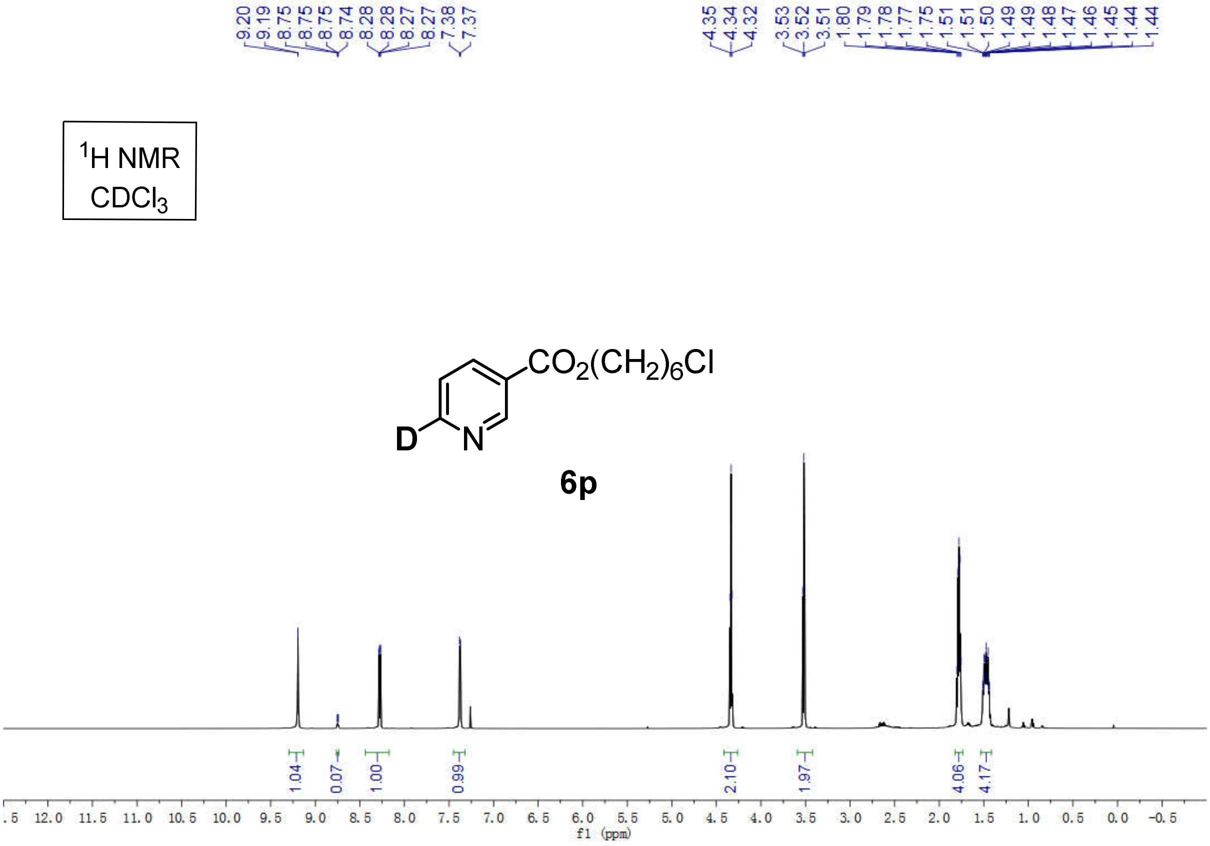
9.20 9.19 8.75 8.75 8.74 8.28 8.28 8.27 8.27 7.38 7.37
4.35 4.34 4.32 3.53 3.52 3.51 1.80 1.79 1.78 1.77 1.75 1.51 1.51 1.50 1.49 1.48 1.47 1.46 1.45 1.44 1.44
1H NMR
CDCl3
CO2(CH2)6Cl
D
N
6p
1.04 0.07 1.00 0.99 2.10 1.97 4.06 4.17
f1 (ppm)

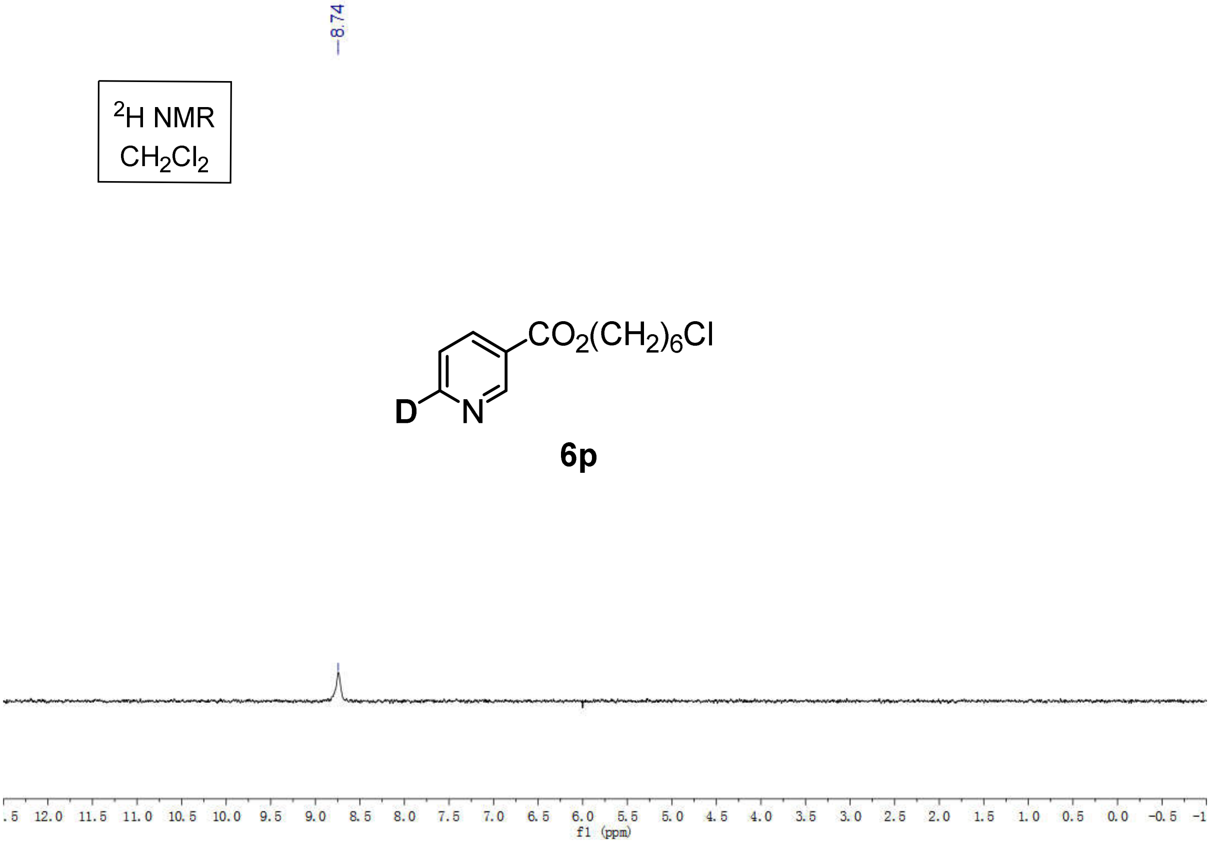
8.74
2H NMR
CH2Cl2
CO2(CH2)6Cl
D
N
6p
f1 (ppm)

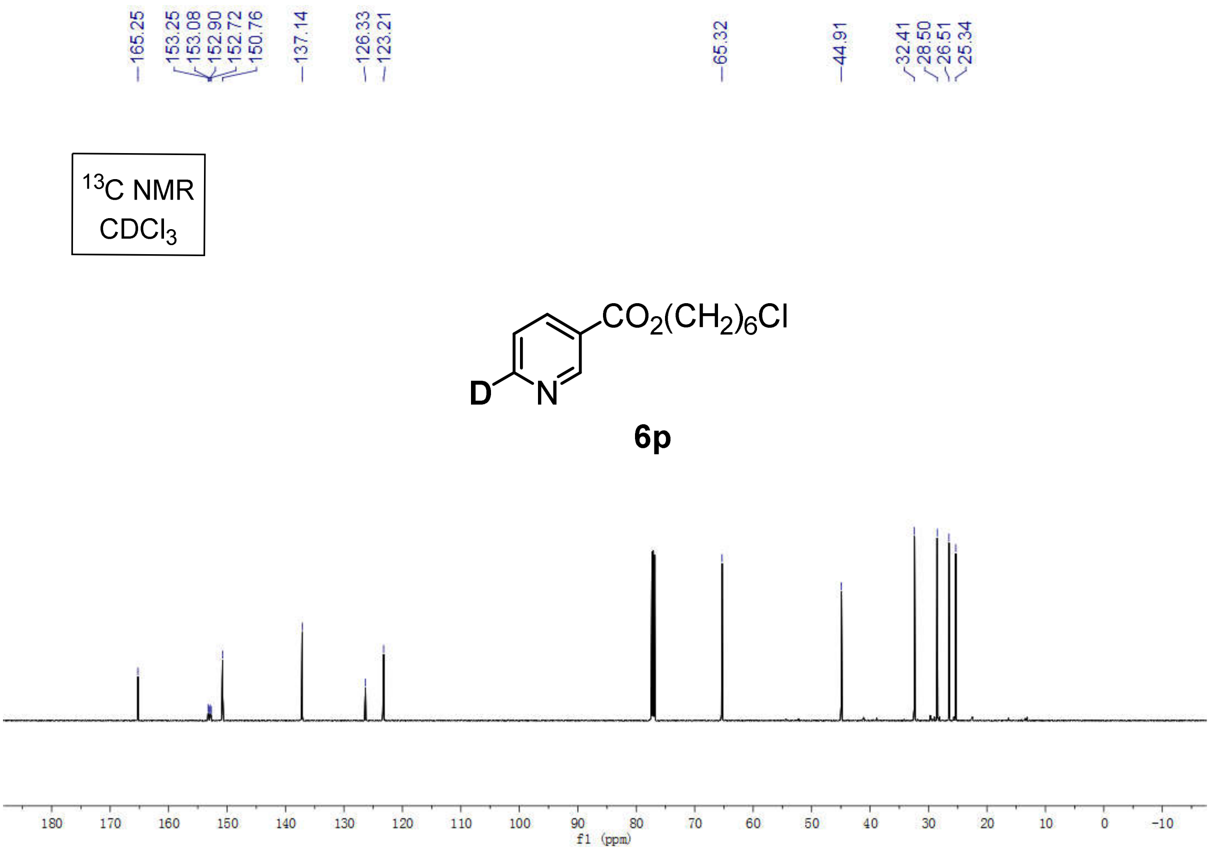


**Supplementary Fig. 105** $^1$H NMR, $^2$H NMR and $^{13}$C NMR spectra of the compound **6p**.

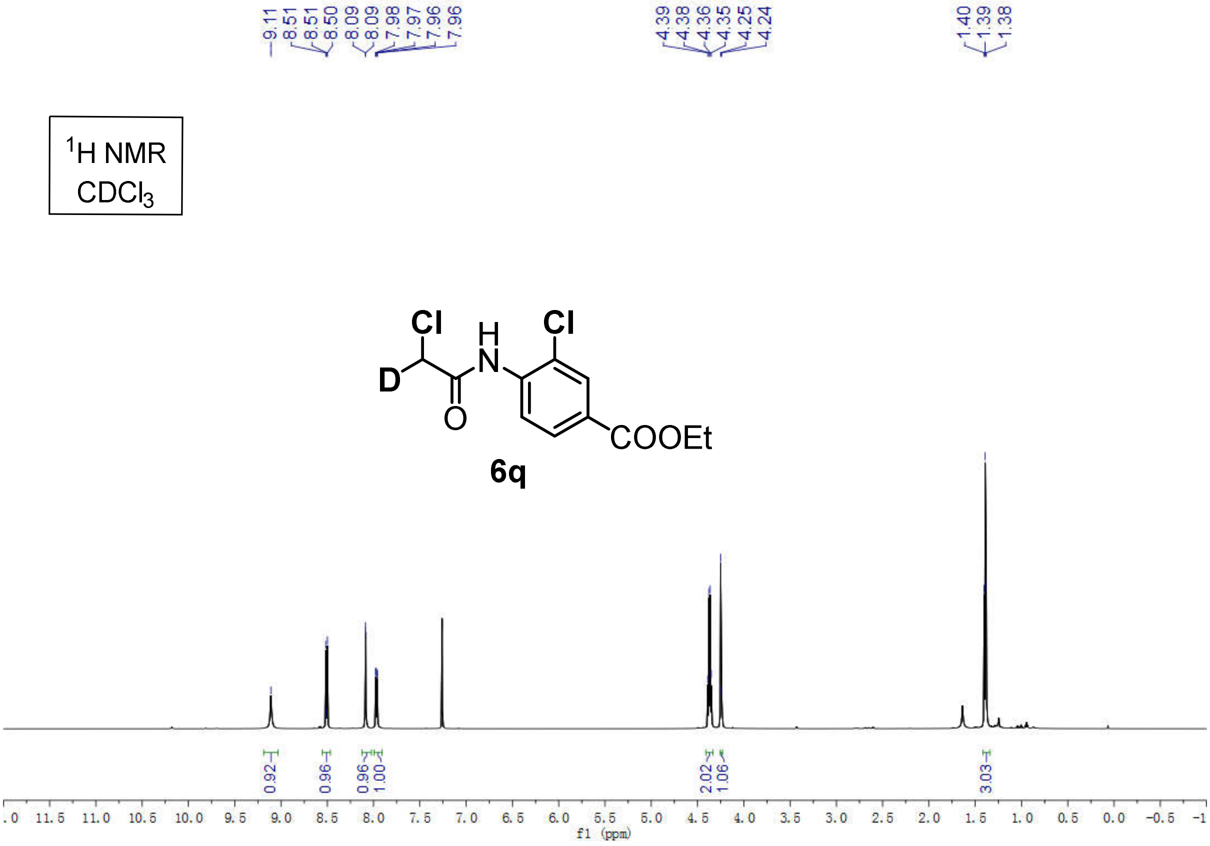

9.11
8.51
8.51
8.50
8.09
8.09
7.98
7.97
7.96
7.96
4.39
4.38
4.36
4.35
4.25
4.24
1.40
1.39
1.38
$^1$H NMR
$CDCl_3$
Cl
H
N
Cl
D
O
COOEt
6q
0.92
0.96
0.96
1.00
2.02
1.06
3.03
f1 (ppm)


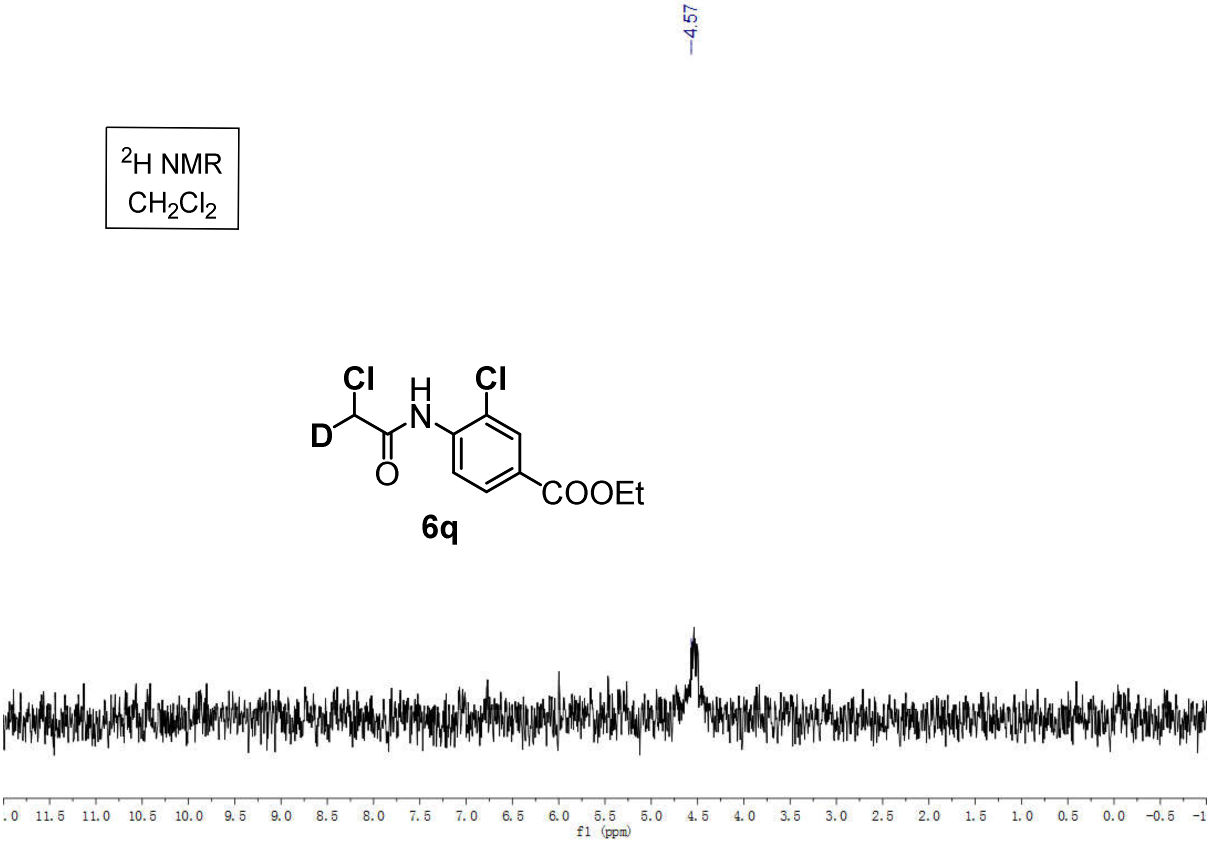

4.57
$^2$H NMR
$CH_2Cl_2$
Cl
H
N
Cl
D
O
COOEt
6q
f1 (ppm)

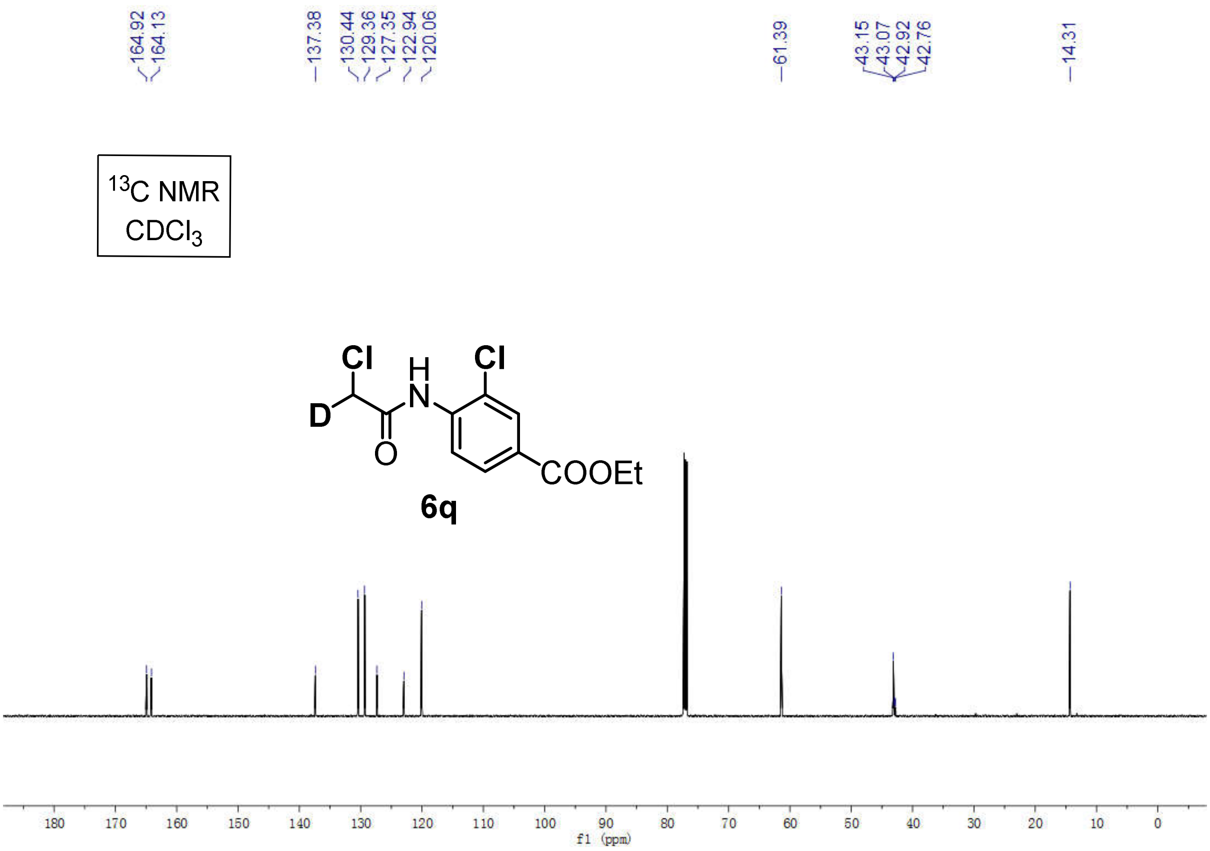


**Supplementary Fig. 106** $^{1}$H NMR, $^{2}$H NMR and $^{13}$C NMR spectra of the compound **6q**.

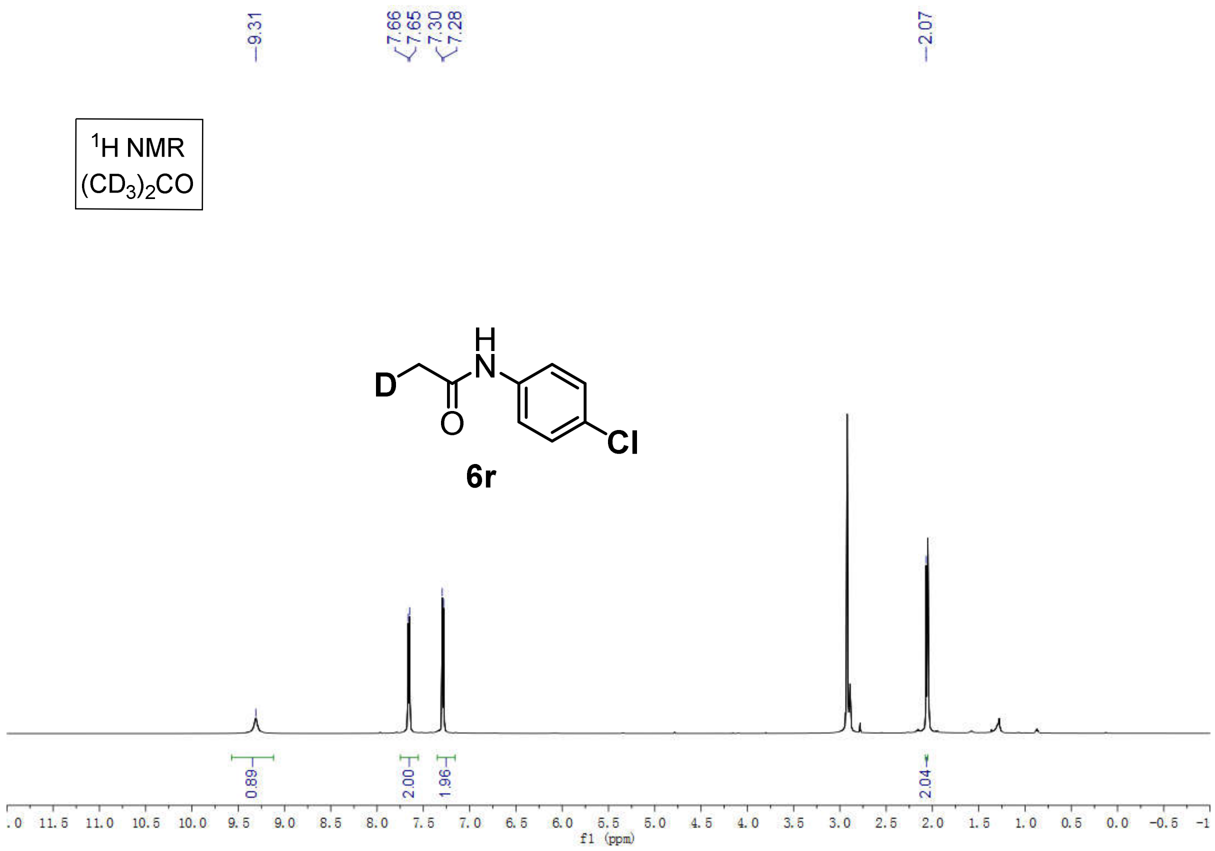
9.31
7.66
7.65
7.30
7.28
2.07
1H NMR
(CD3)2CO
D
H
N
O
Cl
6r
0.89
2.00
1.96
2.04
.0 11.5 11.0 10.5 10.0 9.5 9.0 8.5 8.0 7.5 7.0 6.5 6.0 5.5 5.0 4.5 4.0 3.5 3.0 2.5 2.0 1.5 1.0 0.5 0.0 -0.5 -1
f1 (ppm)

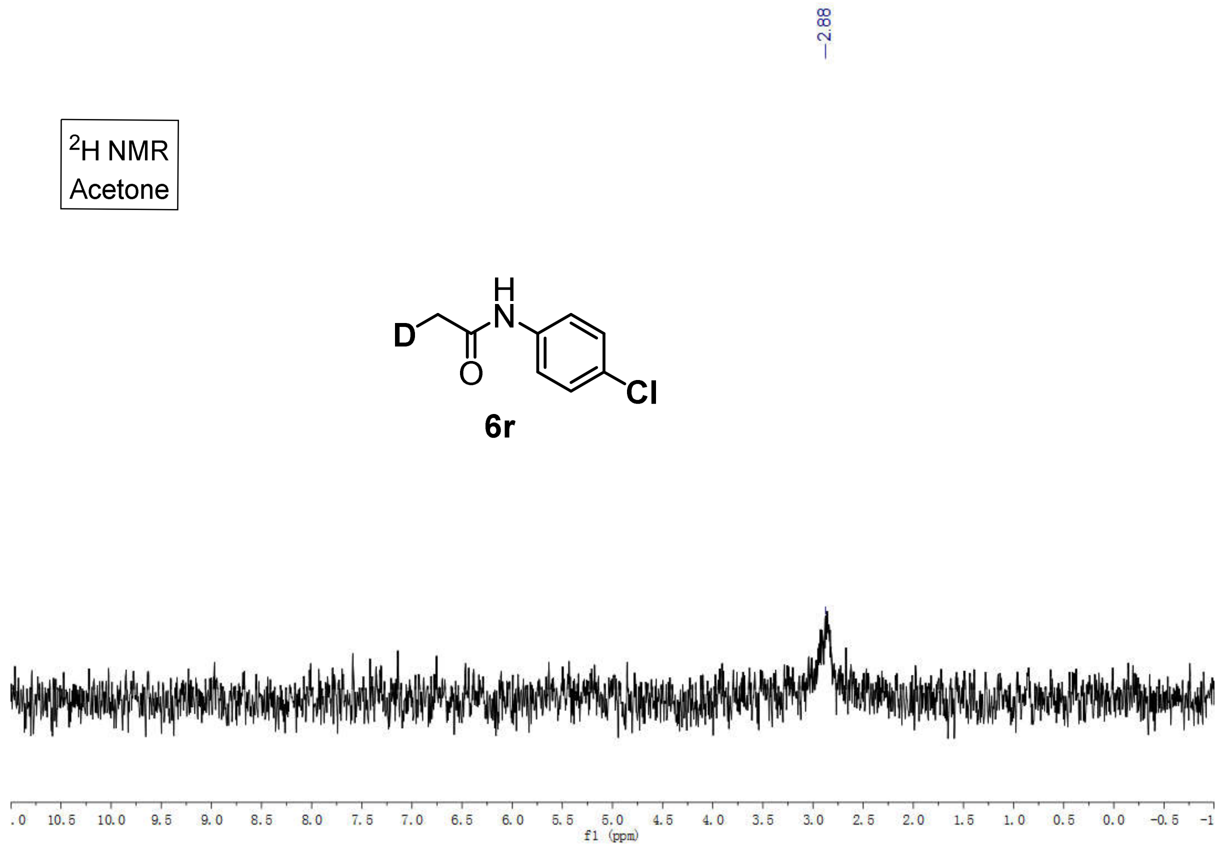
2.88
2H NMR
Acetone
D
H
N
O
Cl
6r
.0 10.5 10.0 9.5 9.0 8.5 8.0 7.5 7.0 6.5 6.0 5.5 5.0 4.5 4.0 3.5 3.0 2.5 2.0 1.5 1.0 0.5 0.0 -0.5 -1
f1 (ppm)

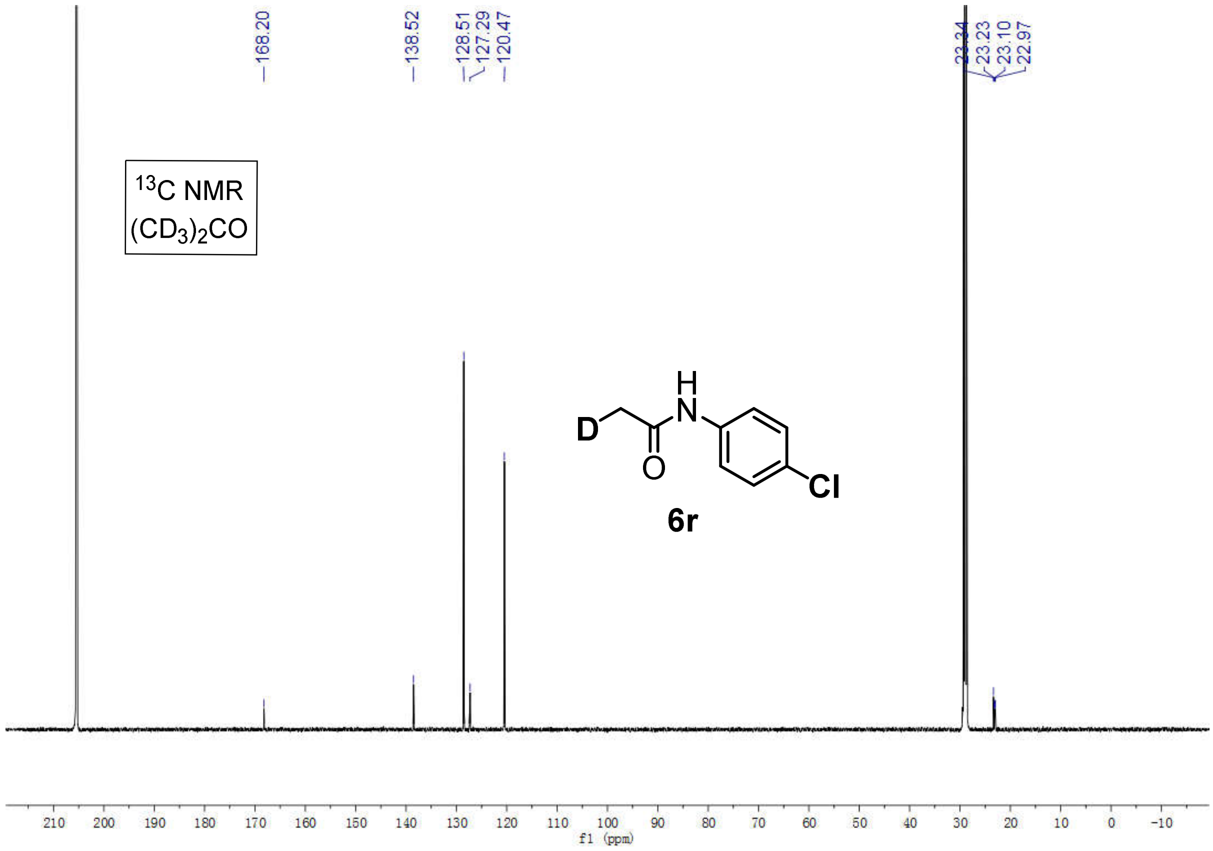


**Supplementary Fig. 107** $^{1}$H NMR, $^{2}$H NMR and $^{13}$C NMR spectra of the compound **6r**.

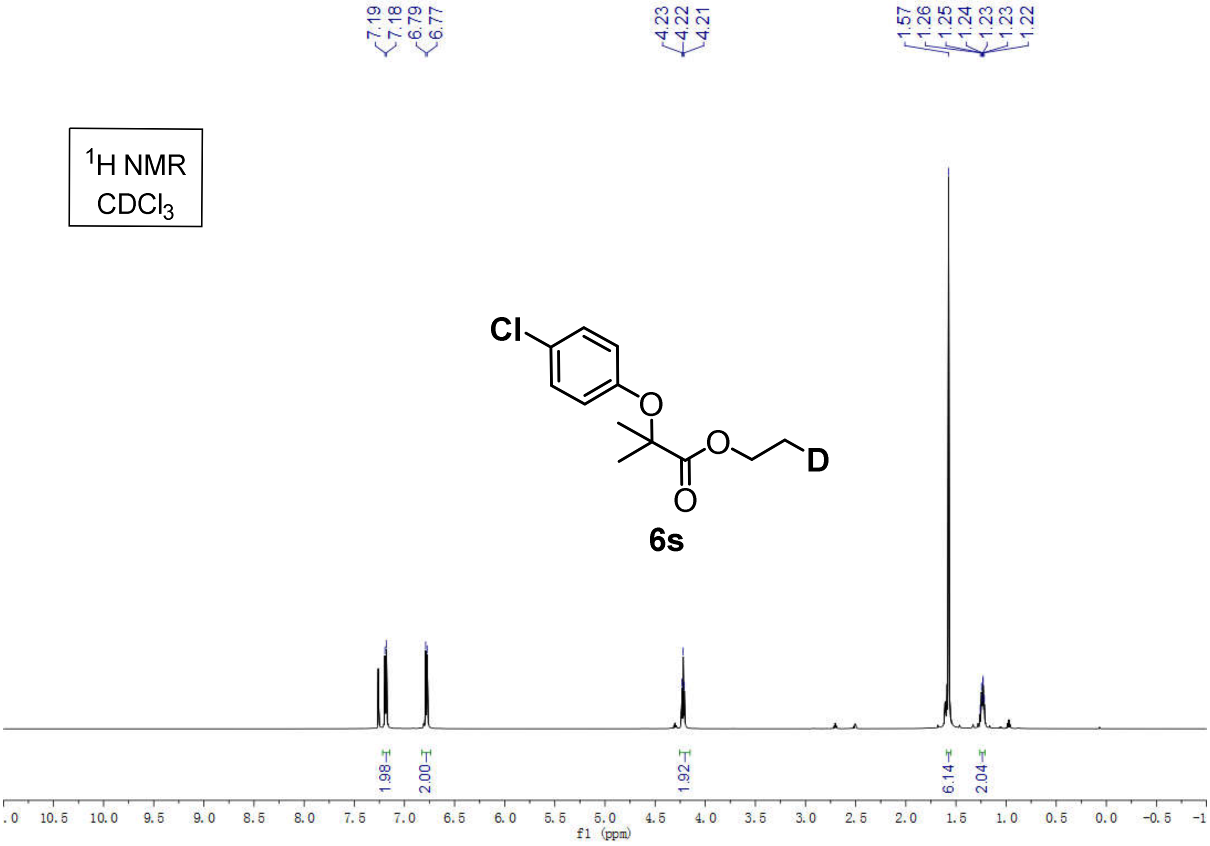
7.19
7.18
6.79
6.77
4.23
4.22
4.21
1.57
1.26
1.25
1.24
1.23
1.23
1.22
1H NMR
CDCl3
Cl
O
O
D
O
6s
1.98
2.00
1.92
6.14
2.04
f1 (ppm)

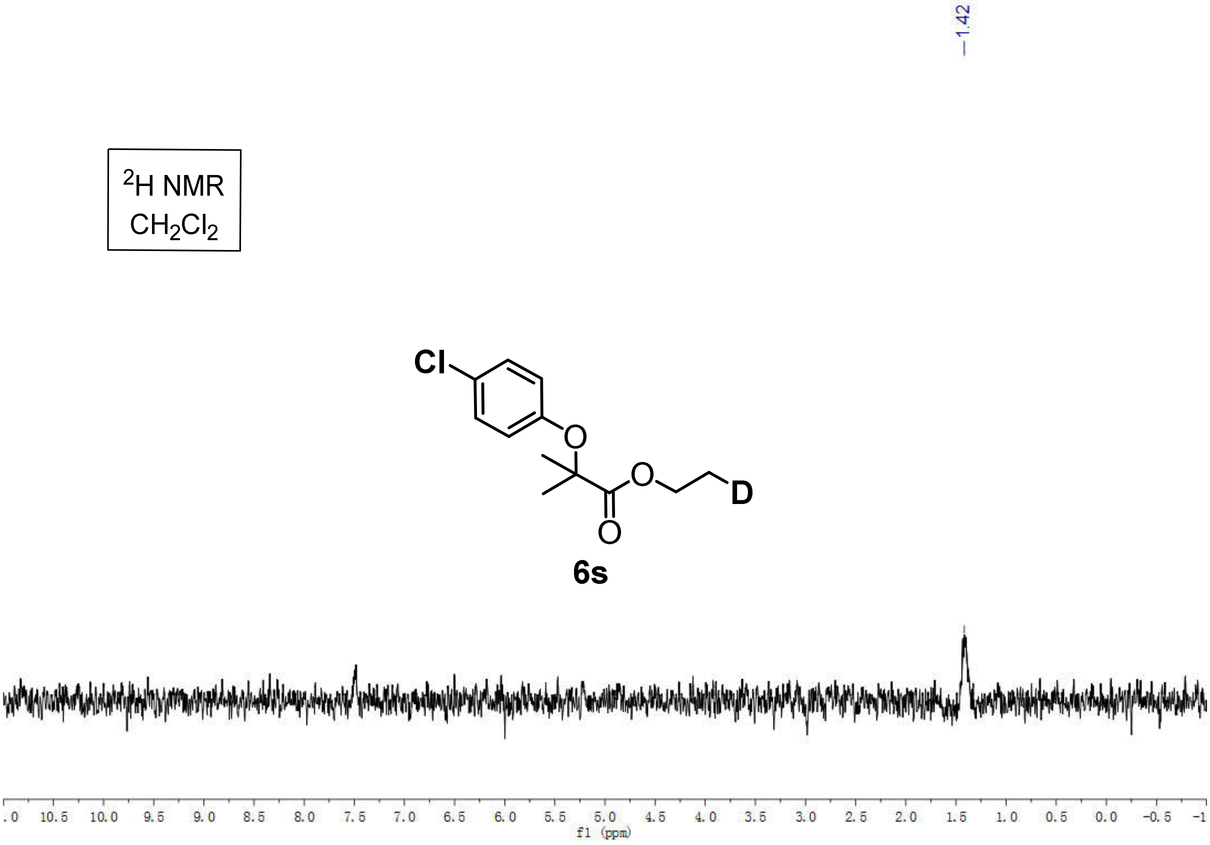
1.42
2H NMR
CH2Cl2
Cl
O
O
D
O
6s
f1 (ppm)

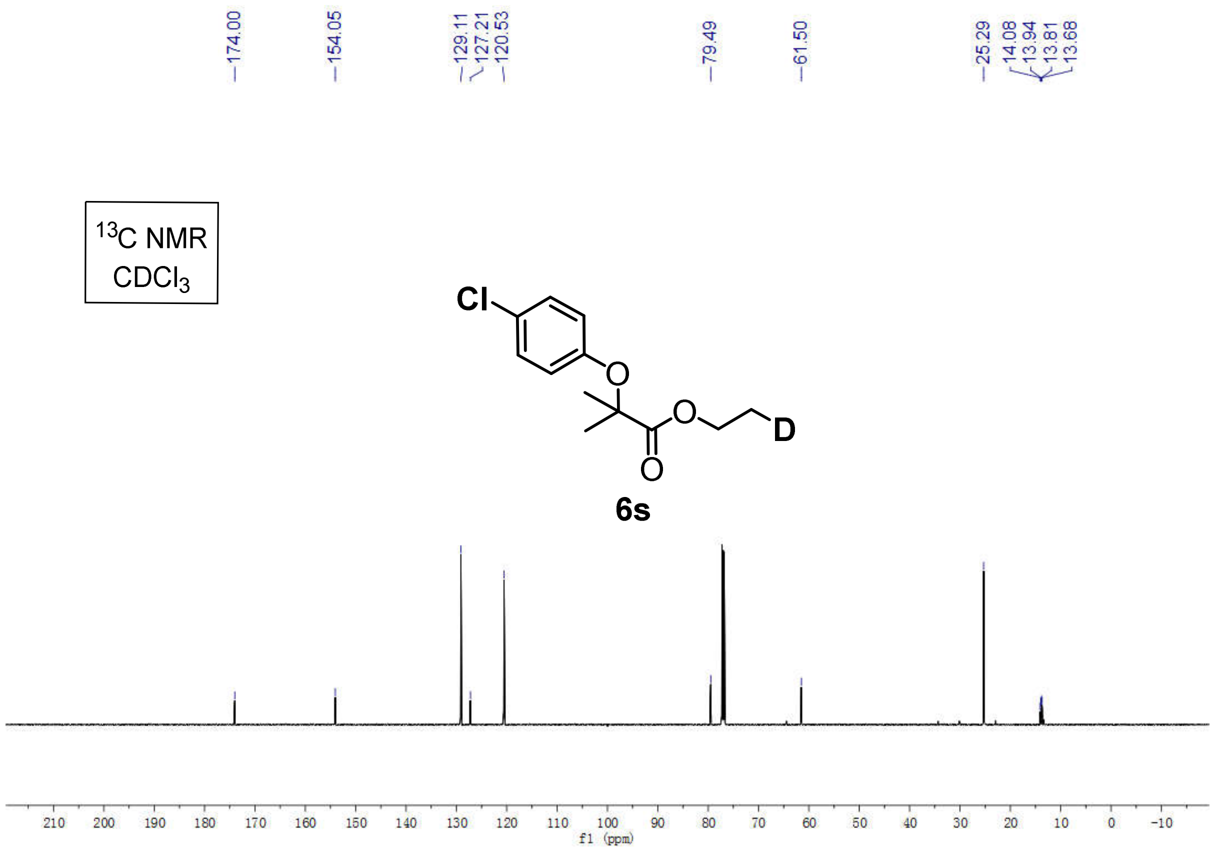


**Supplementary Fig. 108** $^{1}$H NMR, $^{2}$H NMR and $^{13}$C NMR spectra of the compound **6s**.

$^{1}H$ NMR
$CDCl_3$

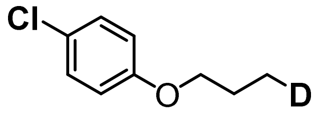

**6t**

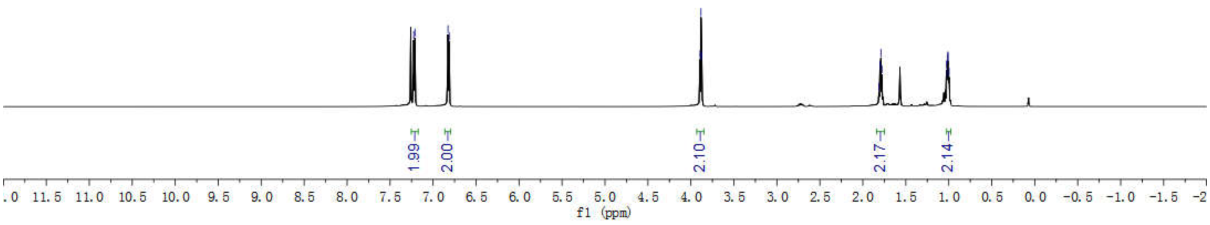


$^{2}H$ NMR
$CH_2Cl_2$

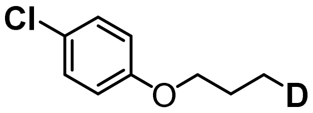

**6t**

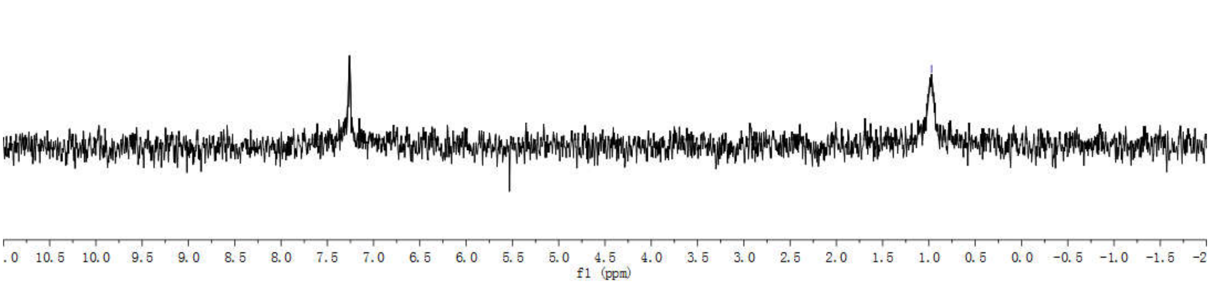

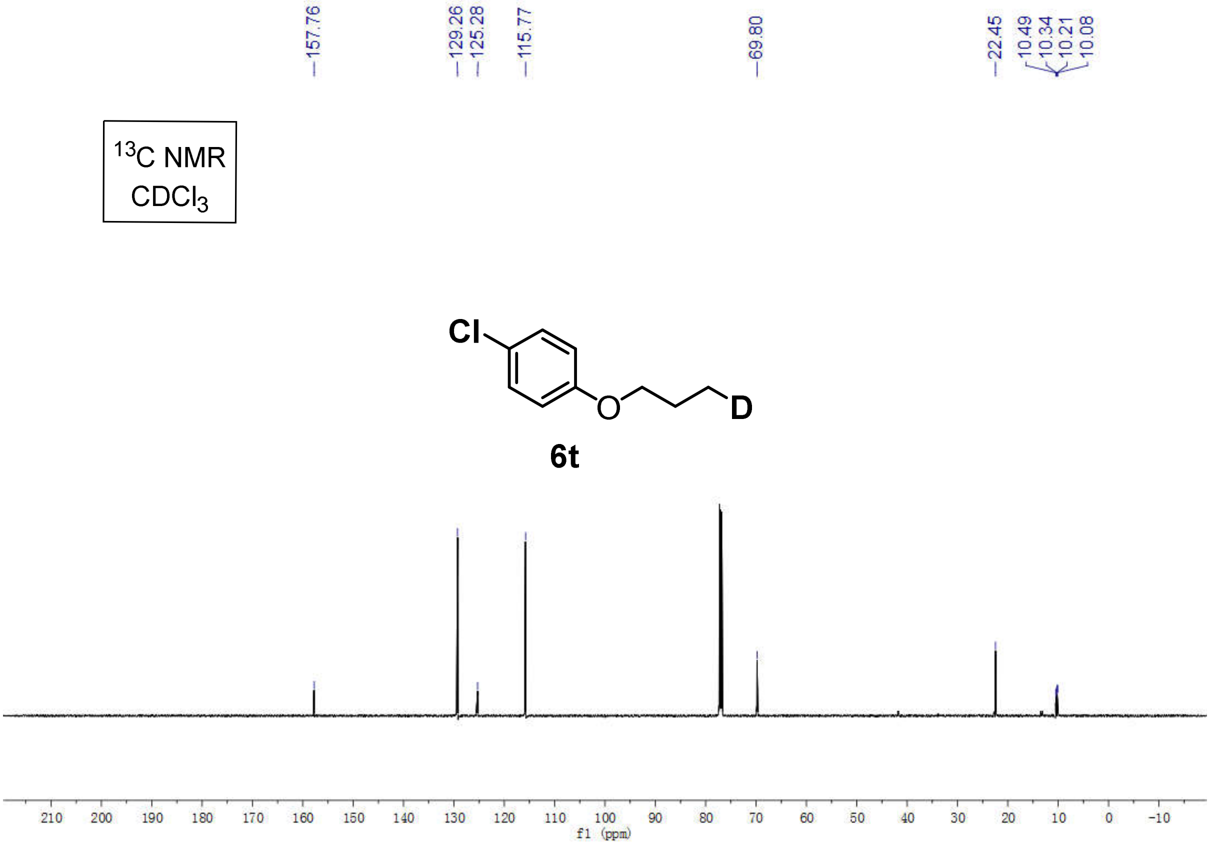


**Supplementary Fig. 109** $^{1}$H NMR, $^{2}$H NMR and $^{13}$C NMR spectra of the compound **6t**.

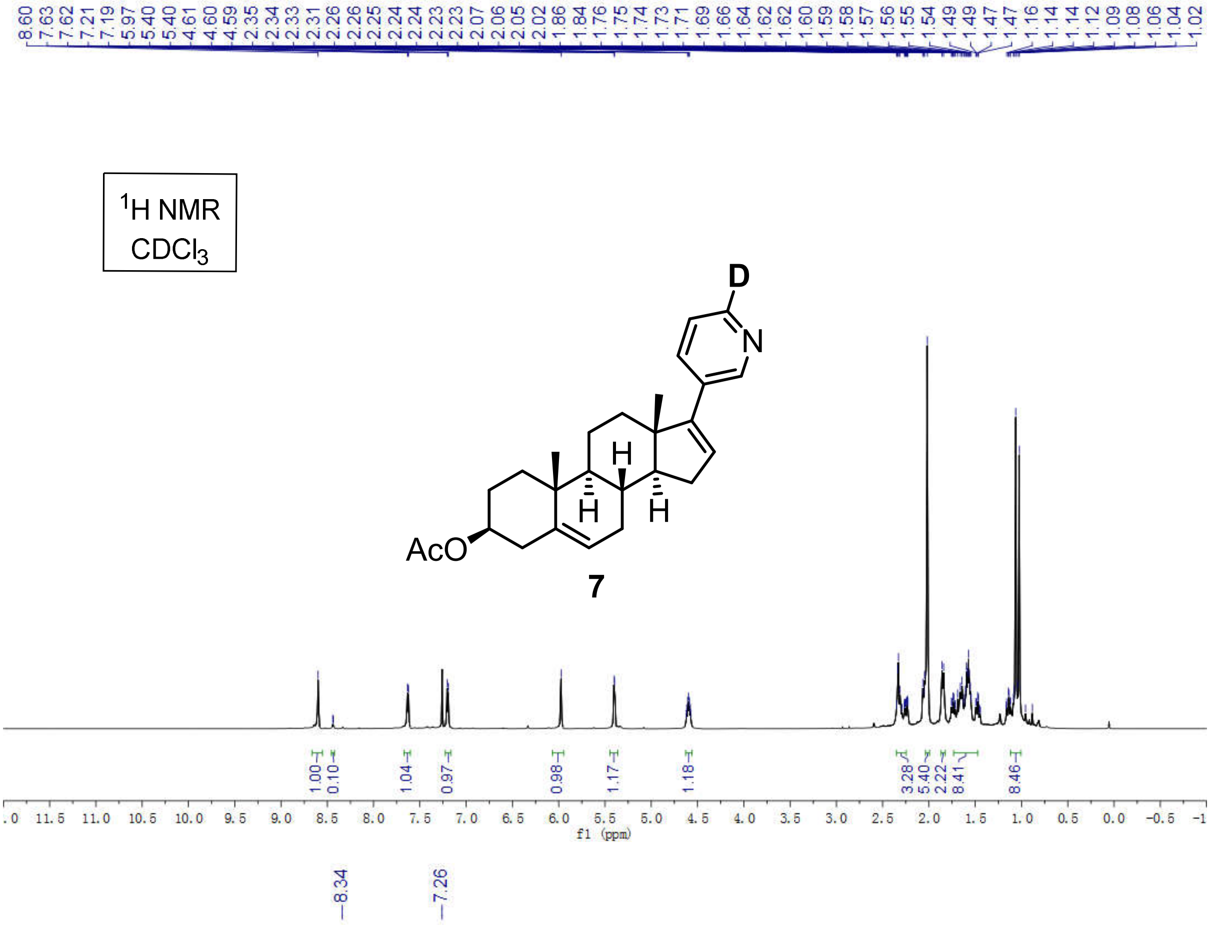
8.60 7.63 7.62 7.21 7.19 5.97 5.40 5.40 4.61 4.60 4.59 2.35 2.34 2.33 2.31 2.26 2.26 2.25 2.24 2.24 2.23 2.23 2.07 2.06 2.05 2.02 1.86 1.84 1.76 1.75 1.74 1.73 1.71 1.69 1.66 1.64 1.62 1.62 1.60 1.59 1.58 1.57 1.56 1.55 1.54 1.49 1.49 1.47 1.47 1.16 1.14 1.14 1.12 1.09 1.08 1.06 1.04 1.02
$^1$H NMR
$CDCl_3$
D
N
H
H
H
AcO
7
1.00 0.10 1.04 0.97 0.98 1.17 1.18 3.28 5.40 2.22 8.41 8.46
f1 (ppm)
8.34
7.26

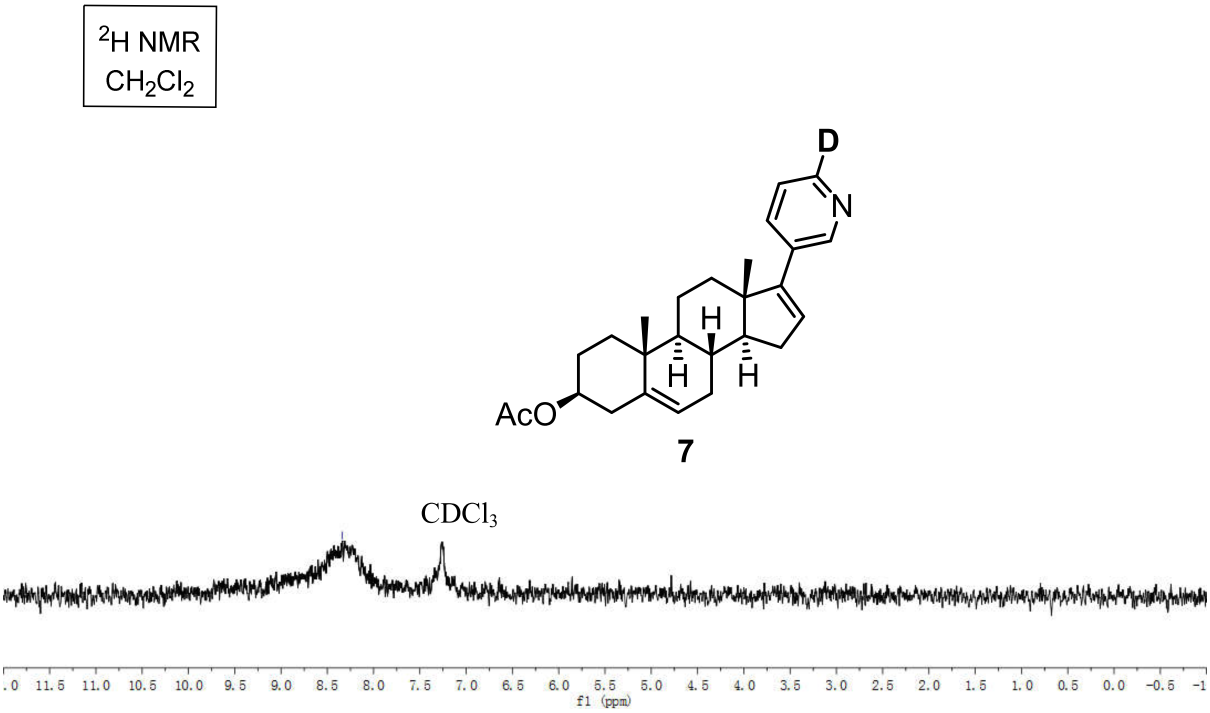
$^2$H NMR
$CH_2Cl_2$
D
N
H
H
H
AcO
7
$CDCl_3$
f1 (ppm)

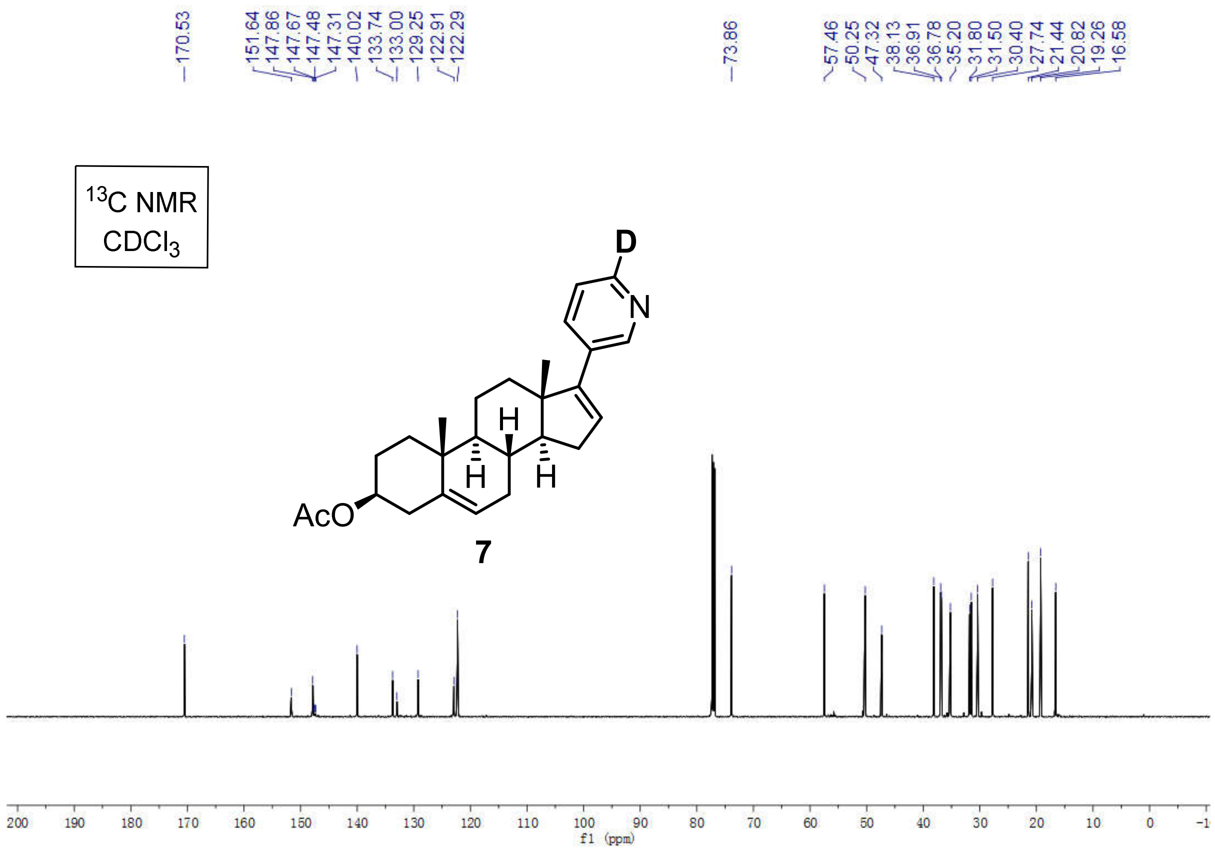


**Supplementary Fig. 110** $^{1}$H NMR, $^{2}$H NMR and $^{13}$C NMR spectra of the compound **7**.

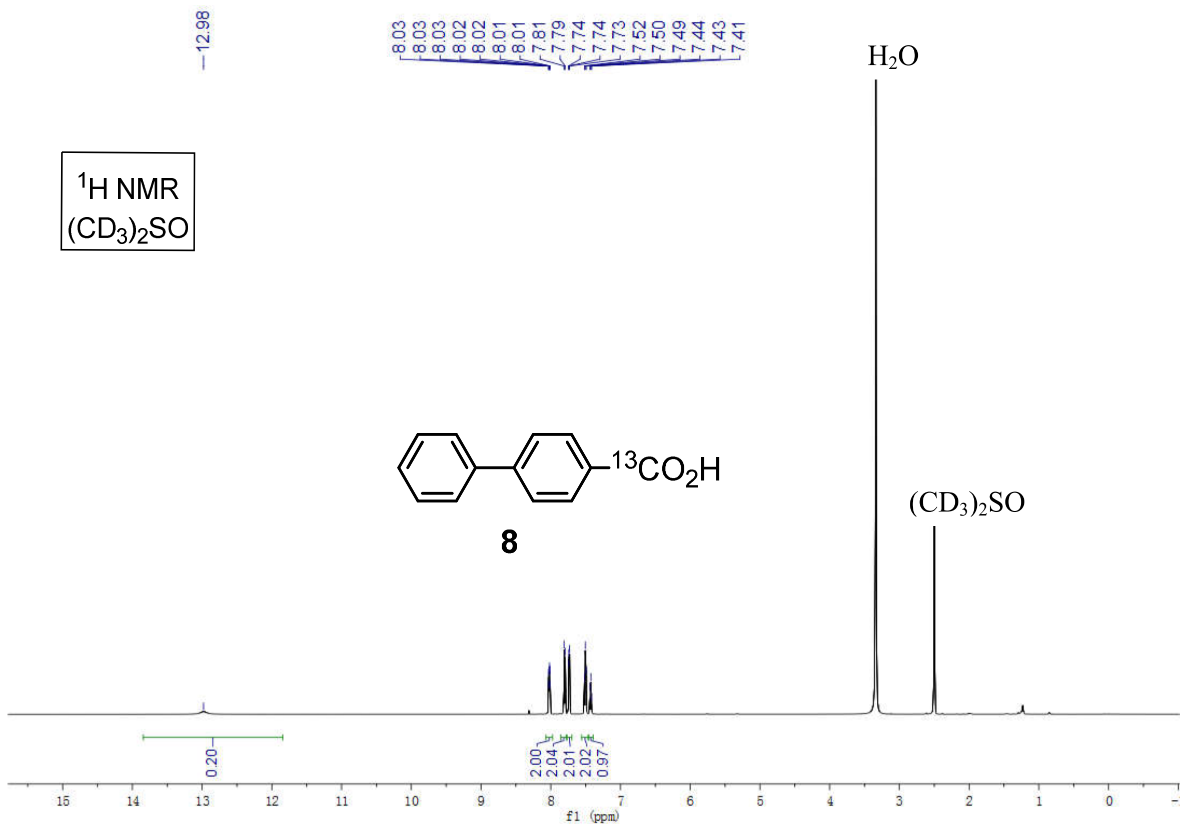


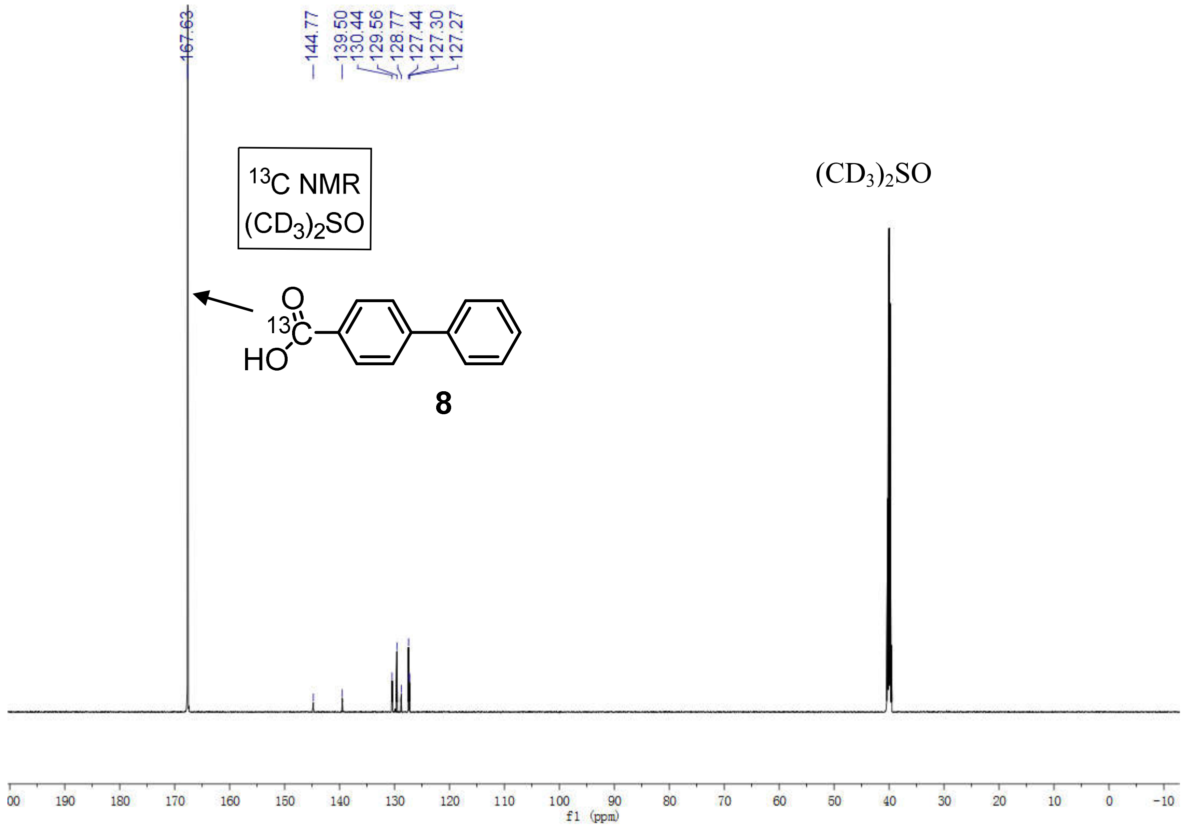


**Supplementary Fig. 111** $^{1}$H NMR and $^{13}$C NMR spectra of the compound **8**.

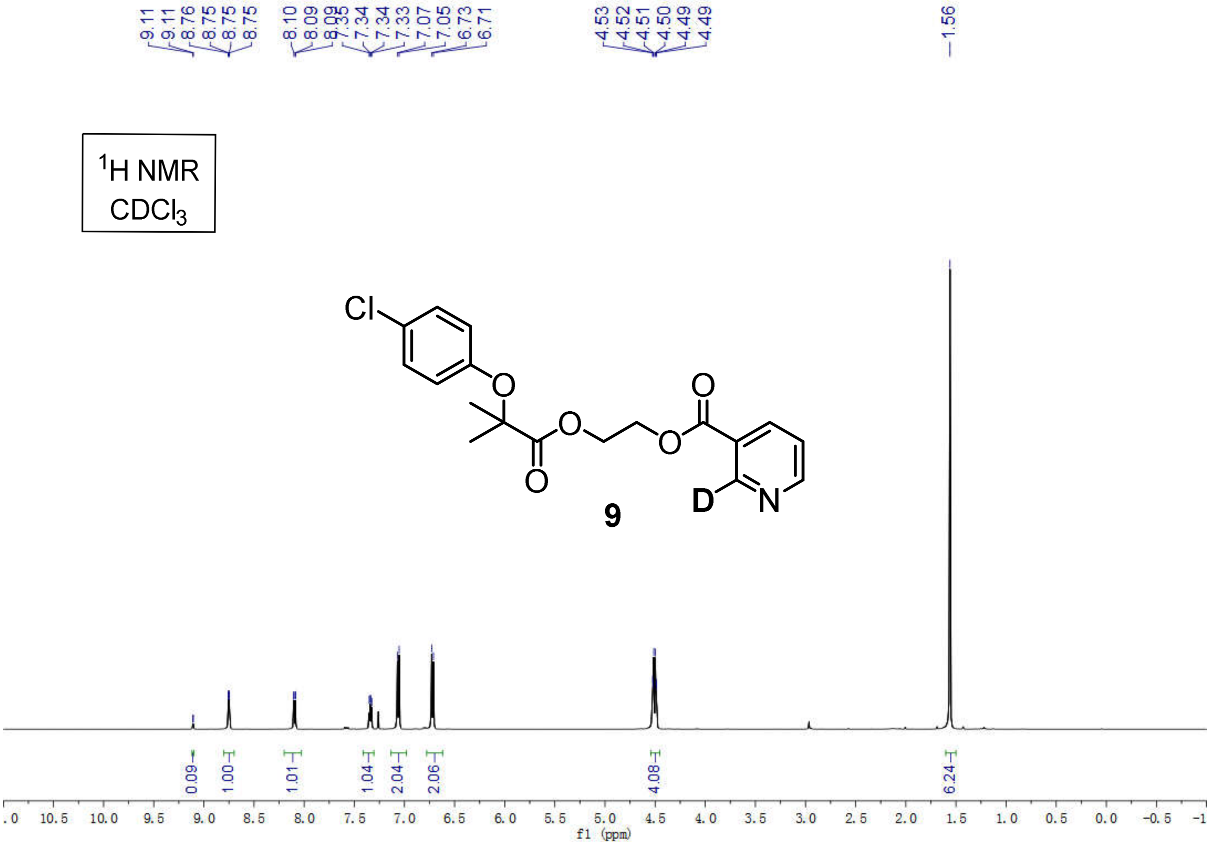
9.11
9.11
8.76
8.75
8.75
8.75
8.10
8.09
8.09
7.35
7.34
7.34
7.33
7.07
7.05
6.73
6.71
4.53
4.52
4.51
4.50
4.49
4.49
1.56
1H NMR
CDCl3
Cl
O
O
O
O
O
D
N
9
0.09
1.00
1.01
1.04
2.04
2.06
4.08
6.24
f1 (ppm)

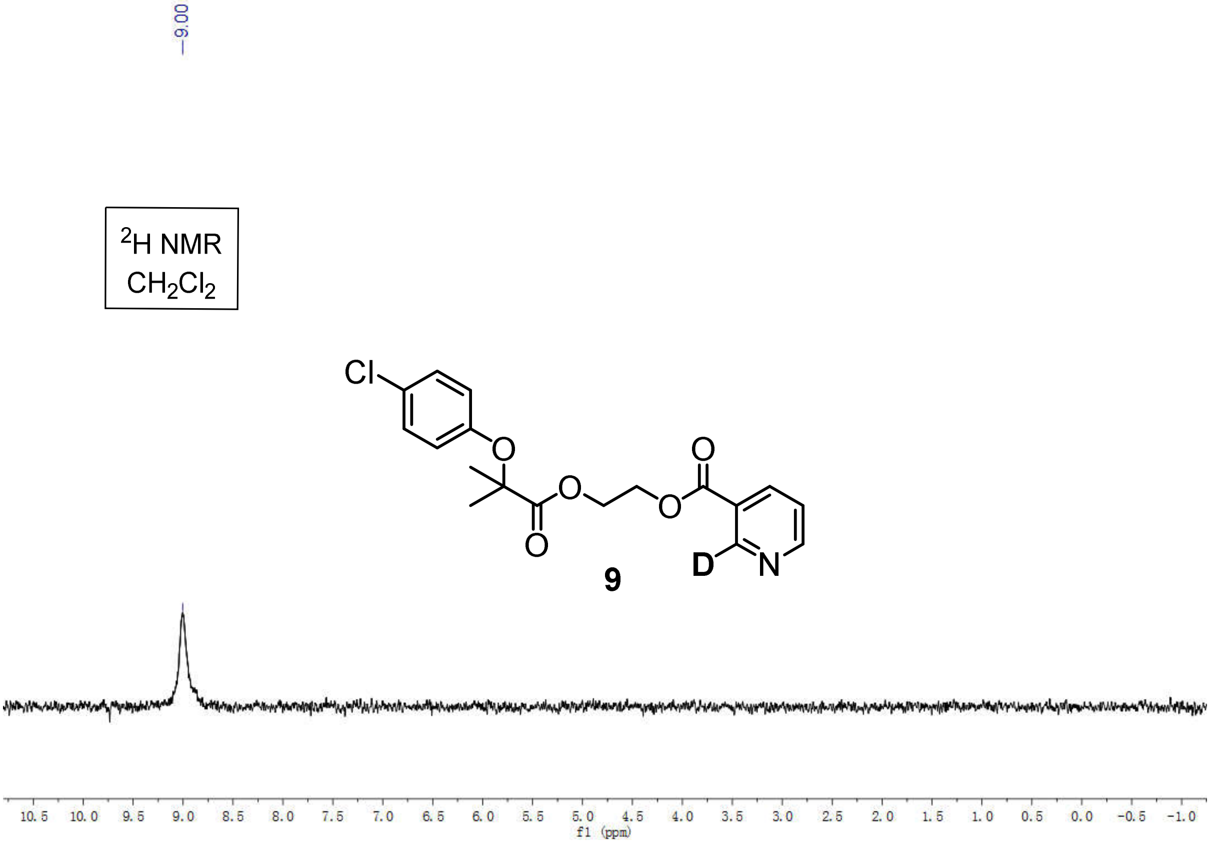
9.00
2H NMR
CH2Cl2
Cl
O
O
O
O
O
D
N
9
f1 (ppm)

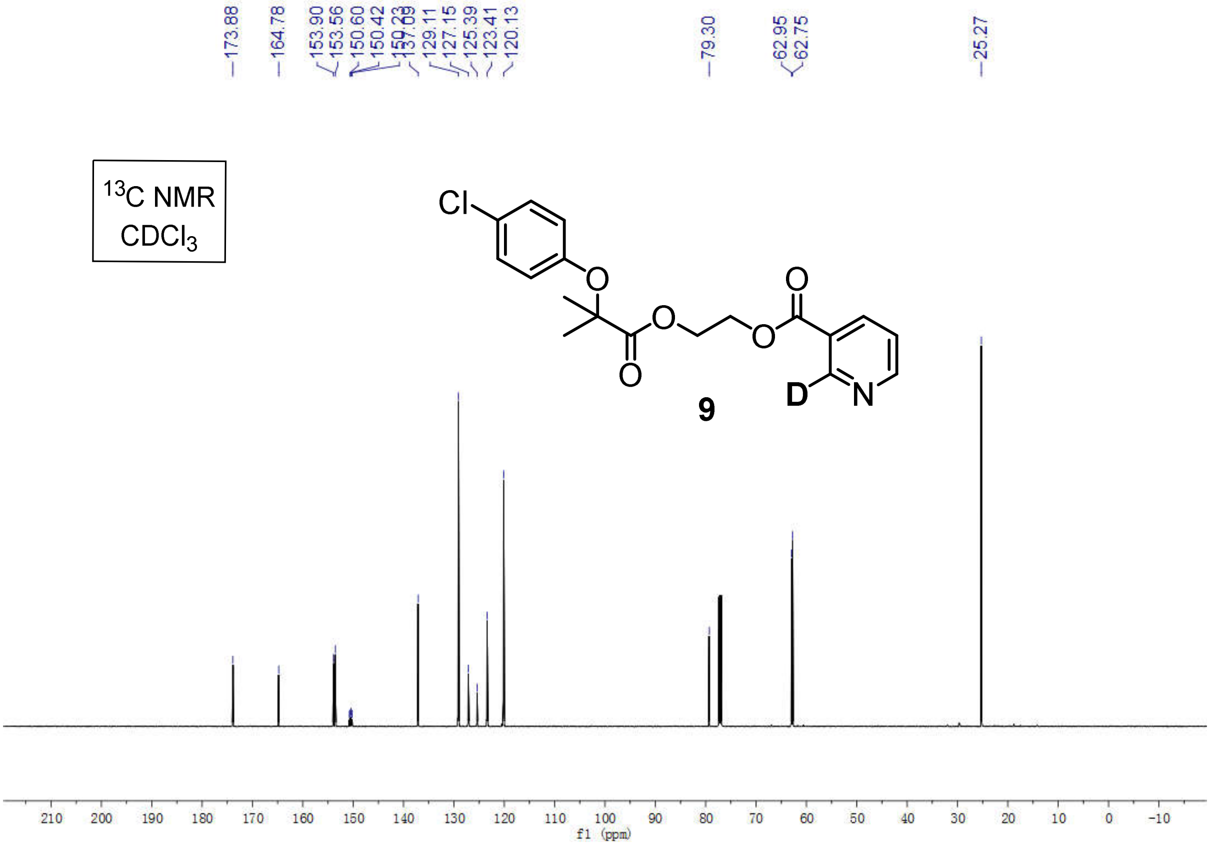


**Supplementary Fig. 112** $^{1}H$ NMR, $^{2}H$ NMR and $^{13}C$ NMR spectra of the compound **9**.

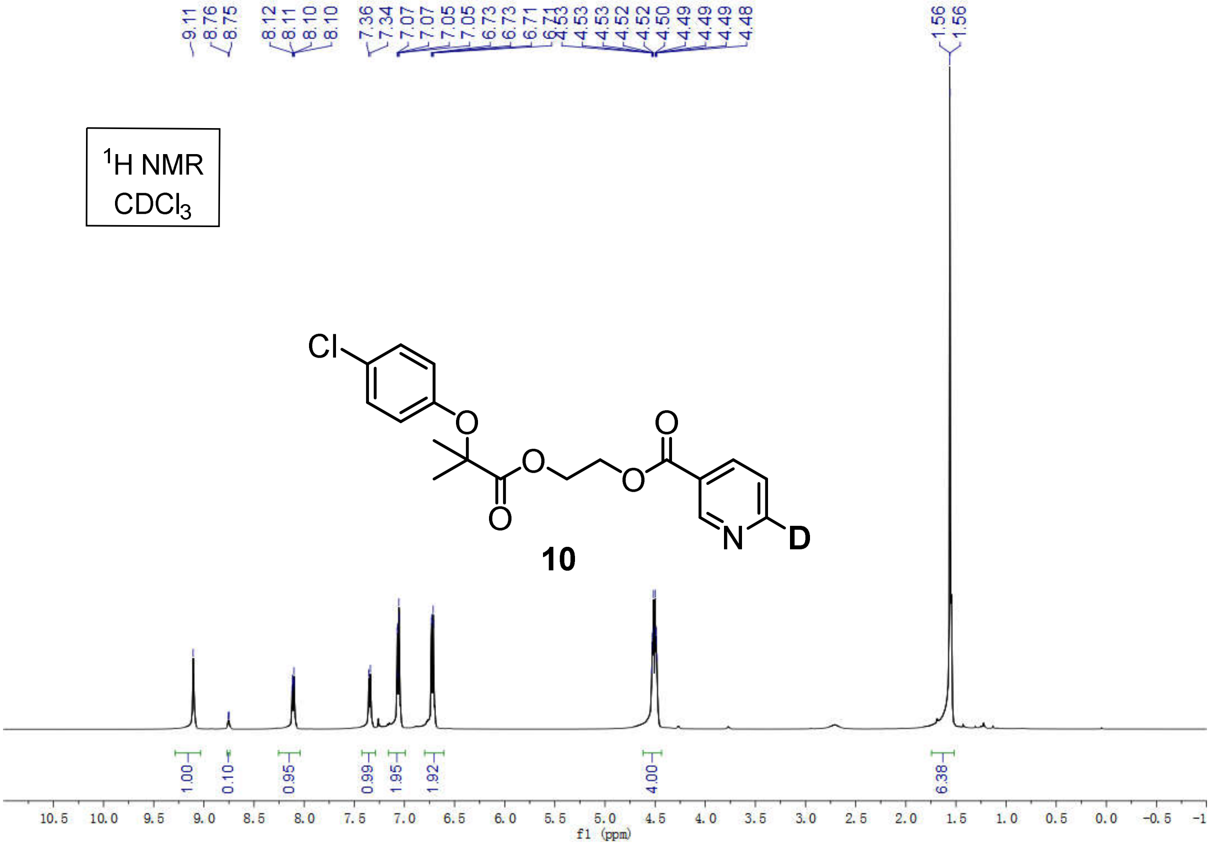
9.11 8.76 8.75 8.12 8.11 8.10 8.10 7.36 7.34 7.07 7.07 7.05 7.05 6.73 6.73 6.71 6.71 4.53 4.53 4.52 4.52 4.50 4.49 4.49 4.49 4.48 1.56 1.56
$^1$H NMR
$CDCl_3$
Cl
O
O
O
O
O
N
D
10
1.00 0.10 0.95 0.99 1.95 1.92 4.00 6.38
f1 (ppm)

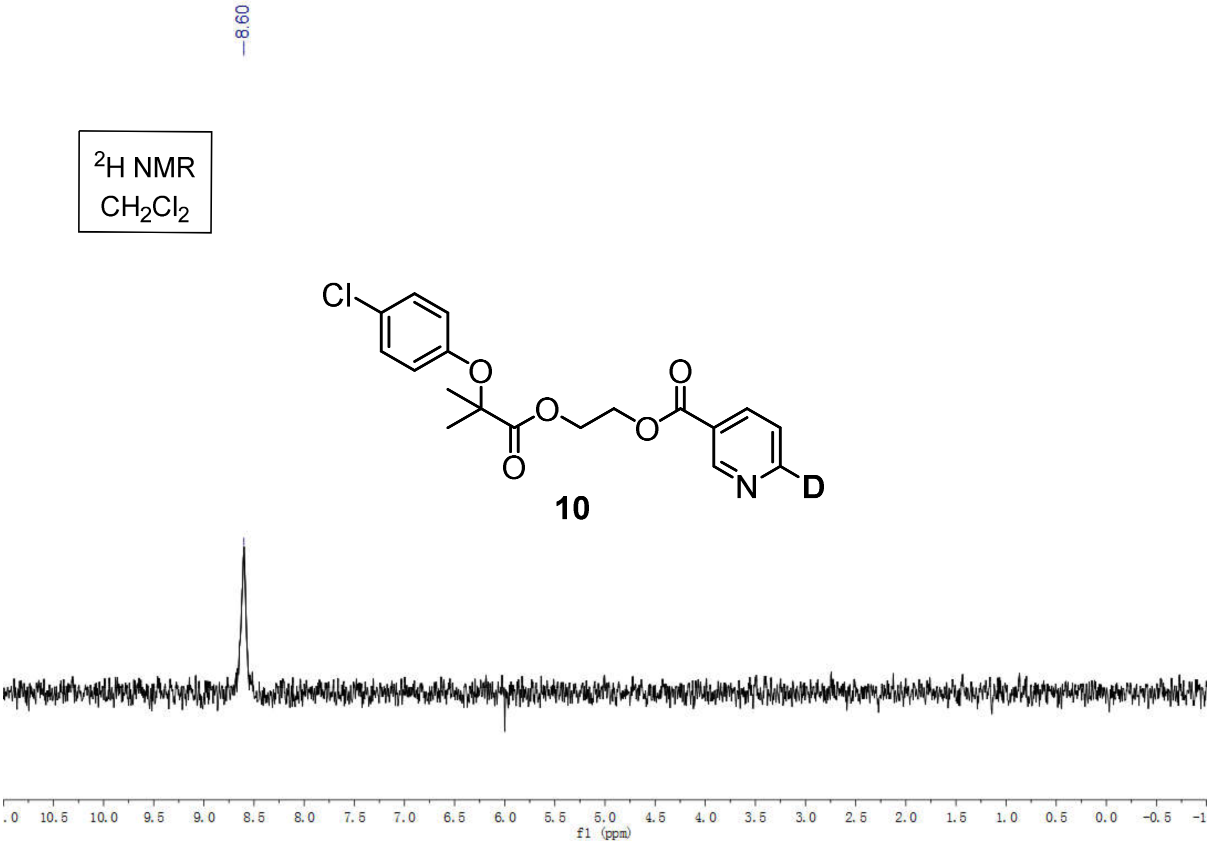
8.60
$^2$H NMR
$CH_2Cl_2$
Cl
O
O
O
O
O
N
D
10
f1 (ppm)

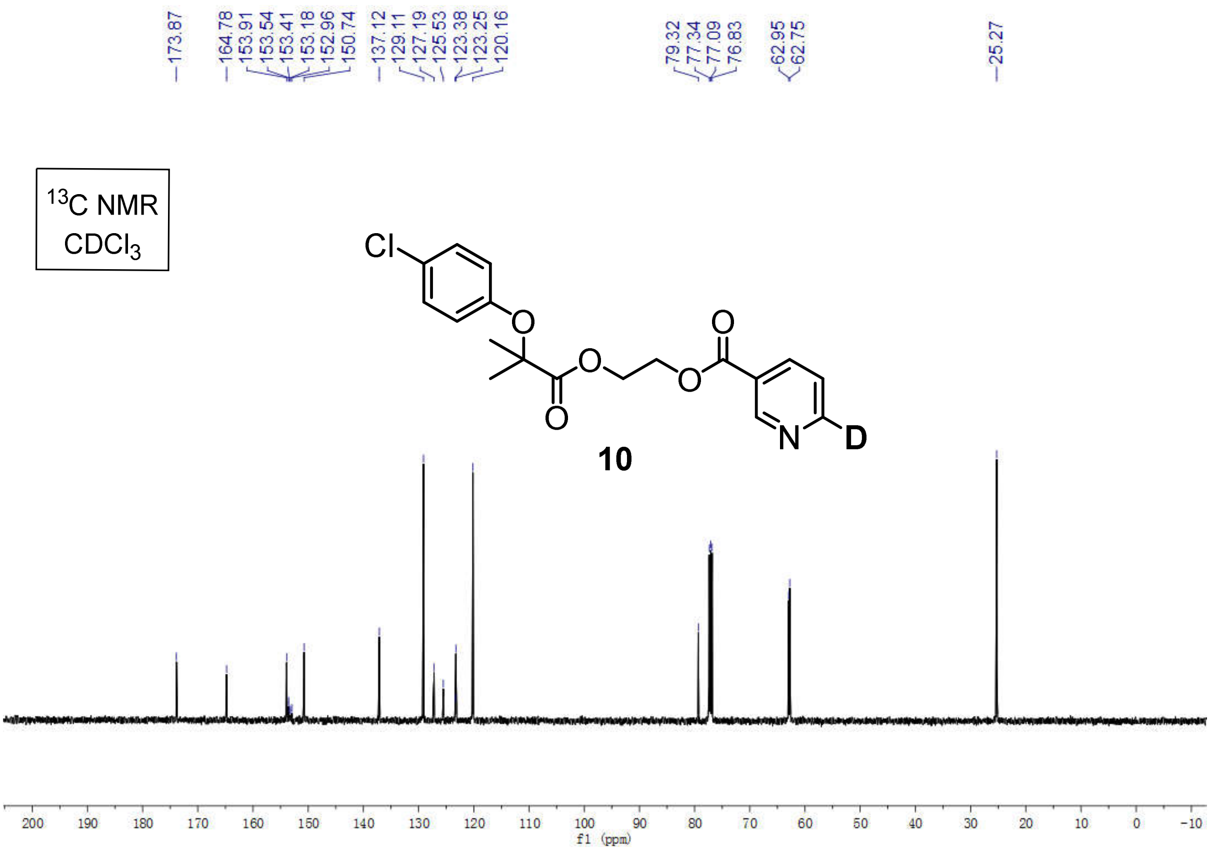


**Supplementary Fig. 113** $^{1}$H NMR, $^{2}$H NMR and $^{13}$C NMR spectra of the compound **10**.

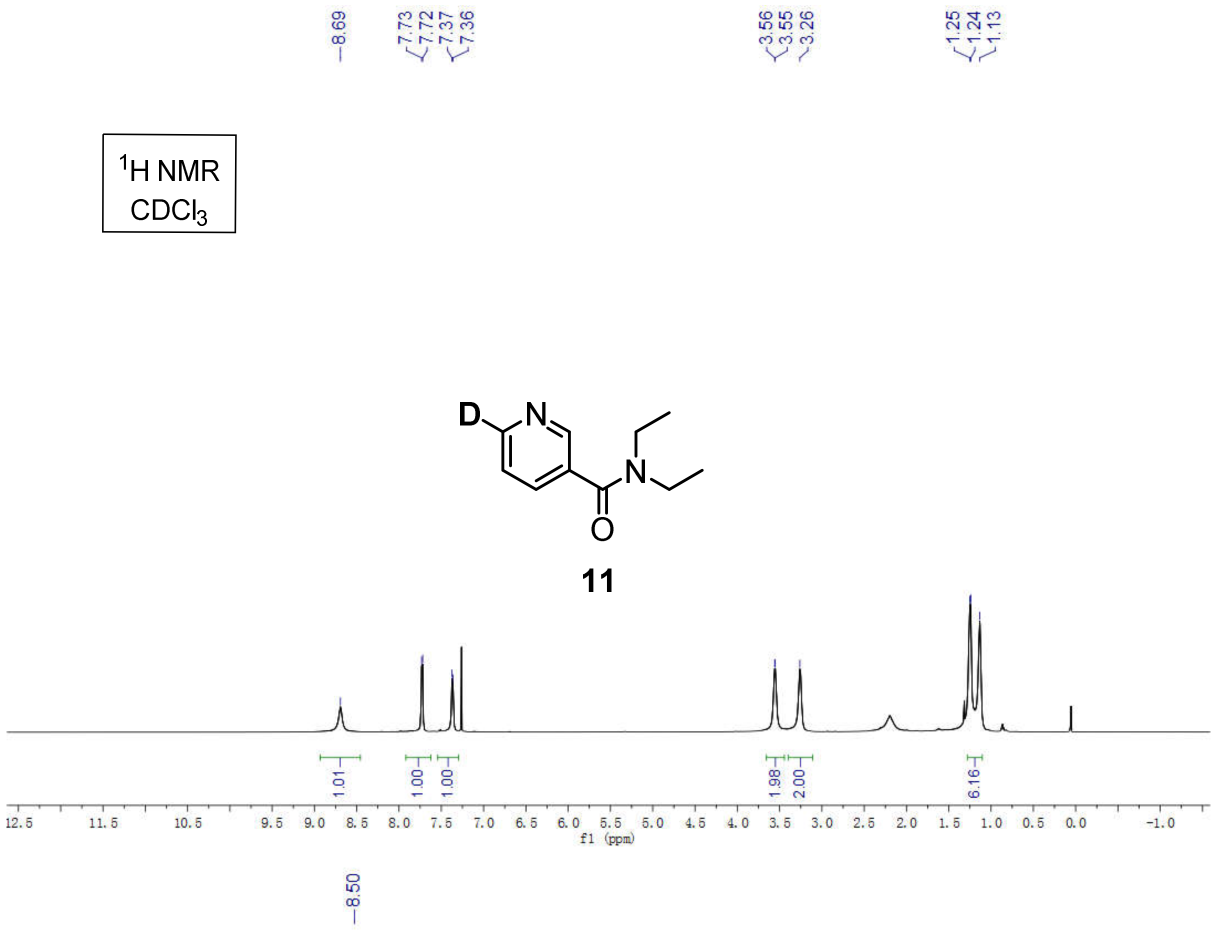
8.69
7.73
7.72
7.37
7.36
3.56
3.55
3.26
1.25
1.24
1.13
$^{1}$H NMR
$CDCl_3$
D
N
N
O
11
1.01
1.00
1.00
1.98
2.00
6.16
12.5 11.5 10.5 9.5 9.0 8.5 8.0 7.5 7.0 6.5 6.0 5.5 5.0 4.5 4.0 3.5 3.0 2.5 2.0 1.5 1.0 0.5 0.0 -1.0
f1 (ppm)

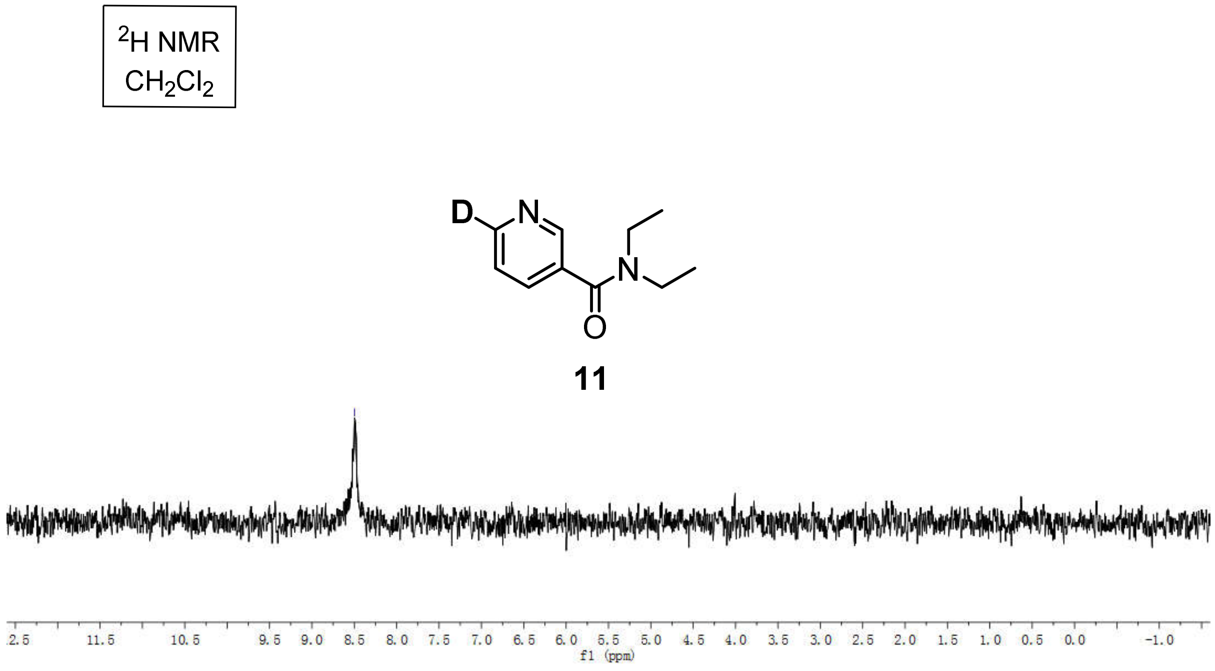
8.50
$^{2}$H NMR
$CH_2Cl_2$
D
N
N
O
11
2.5 11.5 10.5 9.5 9.0 8.5 8.0 7.5 7.0 6.5 6.0 5.5 5.0 4.5 4.0 3.5 3.0 2.5 2.0 1.5 1.0 0.5 0.0 -1.0
f1 (ppm)

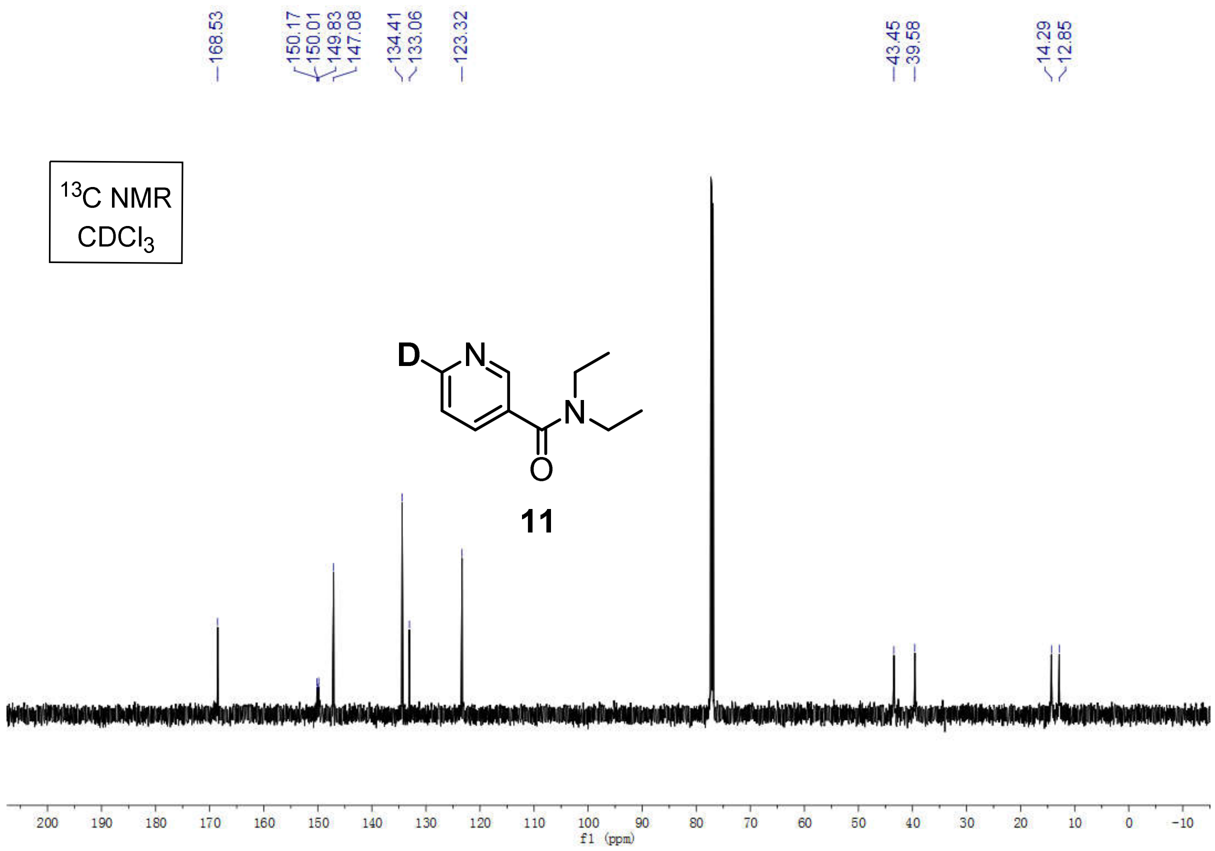


**Supplementary Fig. 114** $^1$H NMR, $^2$H NMR and $^{13}$C NMR spectra of the compound **44**.

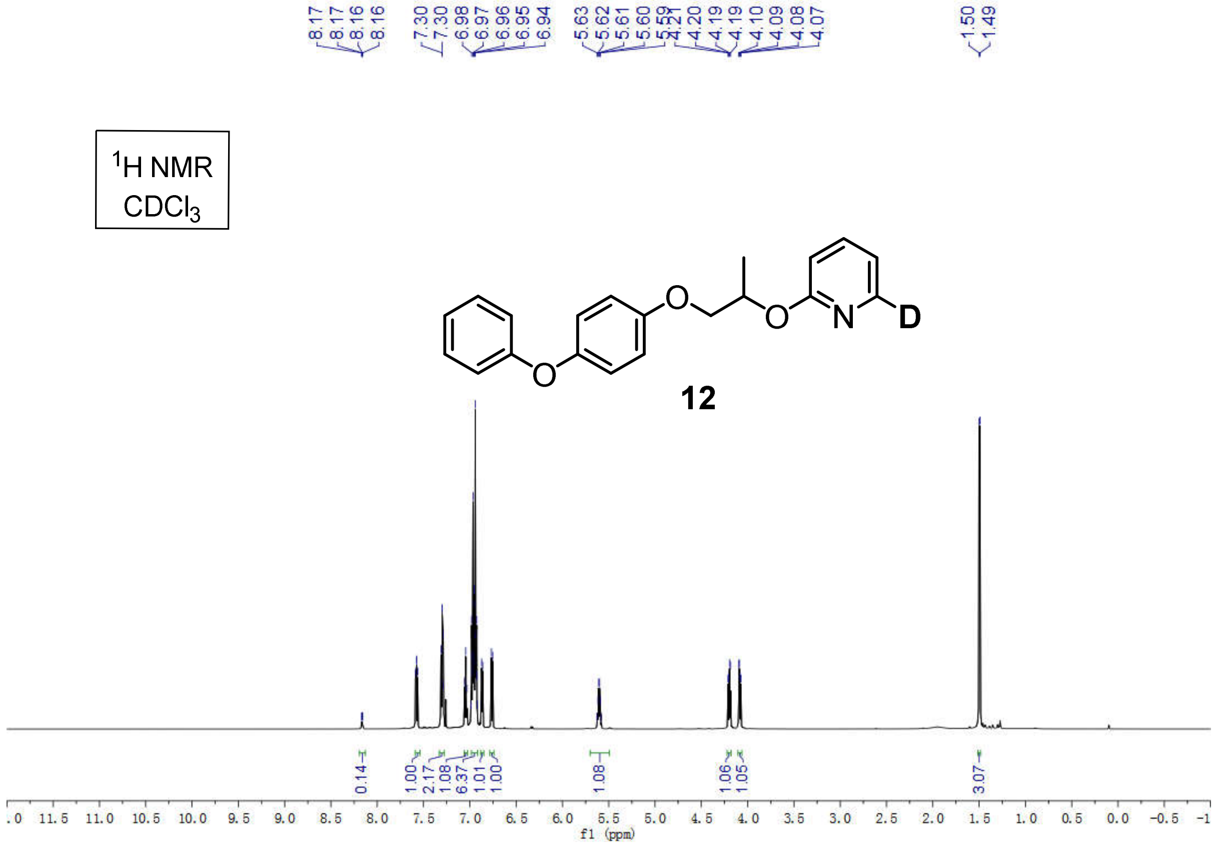

8.17
8.17
8.16
8.16
7.30
7.30
6.98
6.97
6.96
6.95
6.94
5.63
5.62
5.61
5.60
5.59
4.21
4.20
4.19
4.19
4.10
4.09
4.08
4.07
1.50
1.49
$^1$H NMR
$CDCl_3$
O
O
O
N
D
12
0.14
1.00
2.17
1.08
6.37
1.01
1.00
1.08
1.06
1.05
3.07
f1 (ppm)


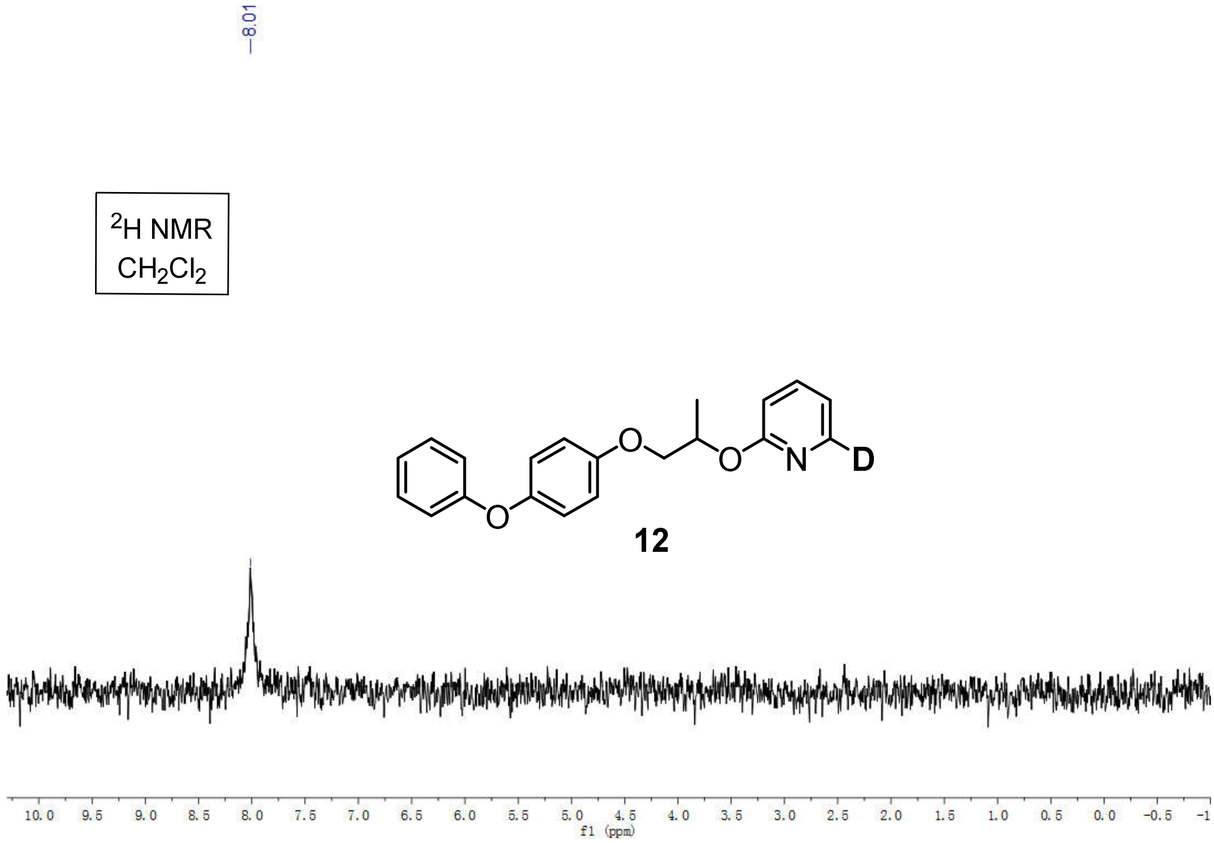

8.01
$^2$H NMR
$CH_2Cl_2$
O
O
O
N
D
12
f1 (ppm)

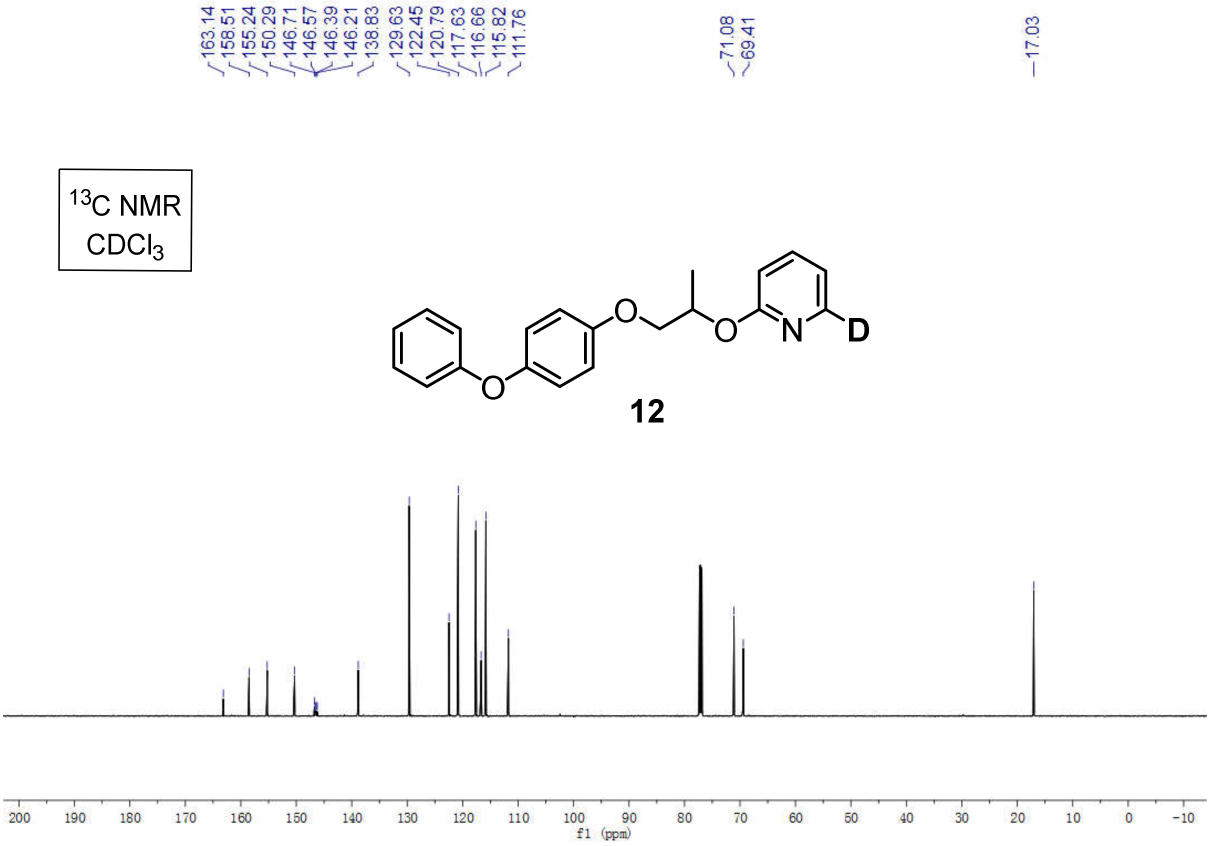


**Supplementary Fig. 115** $^{1}$H NMR, $^{2}$H NMR and $^{13}$C NMR spectra of the compound **12**.

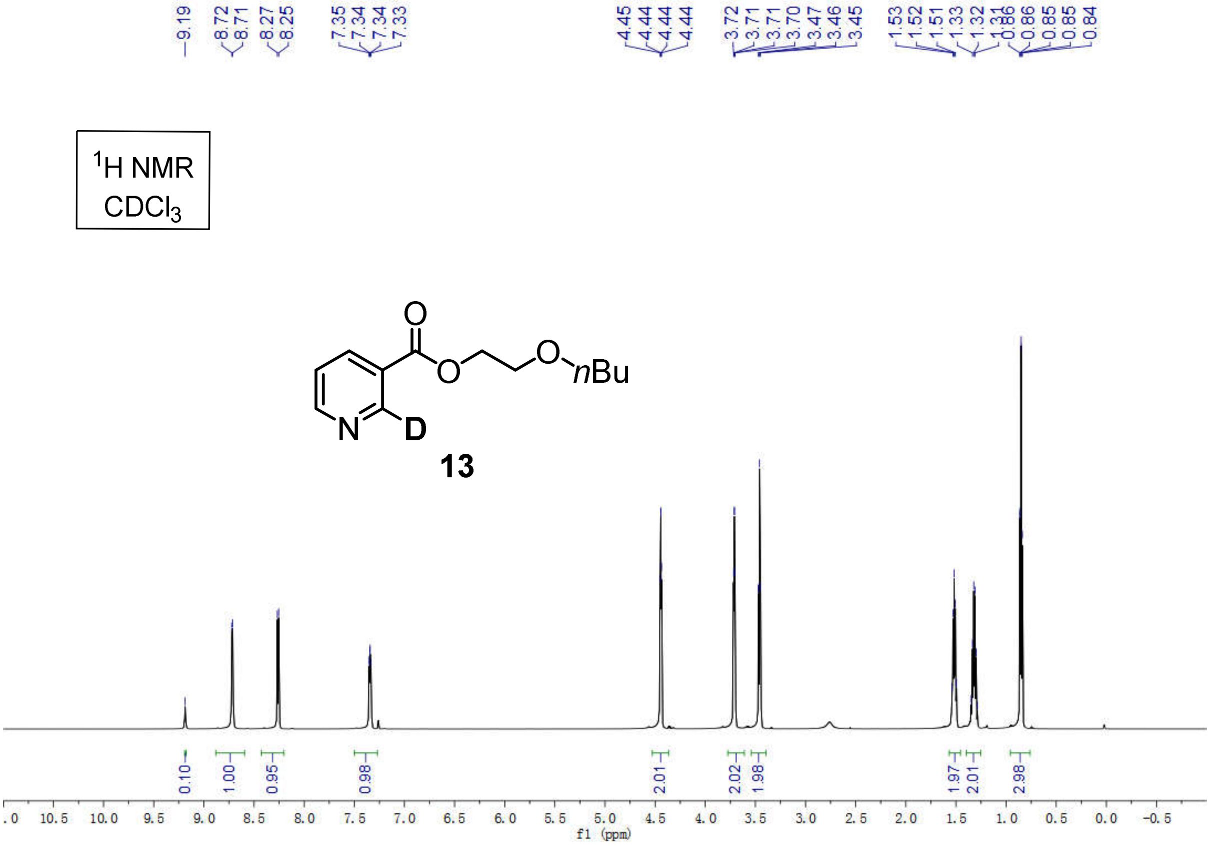

9.19
8.72
8.71
8.27
8.25
7.35
7.34
7.34
7.33
4.45
4.44
4.44
4.44
3.72
3.71
3.71
3.70
3.47
3.46
3.45
1.53
1.52
1.51
1.33
1.32
1.31
0.86
0.85
0.85
0.84
1H NMR
CDCl3
O
O
O
nBu
N
D
13
0.10
1.00
0.95
0.98
2.01
2.02
1.98
1.97
2.01
2.98
f1 (ppm)


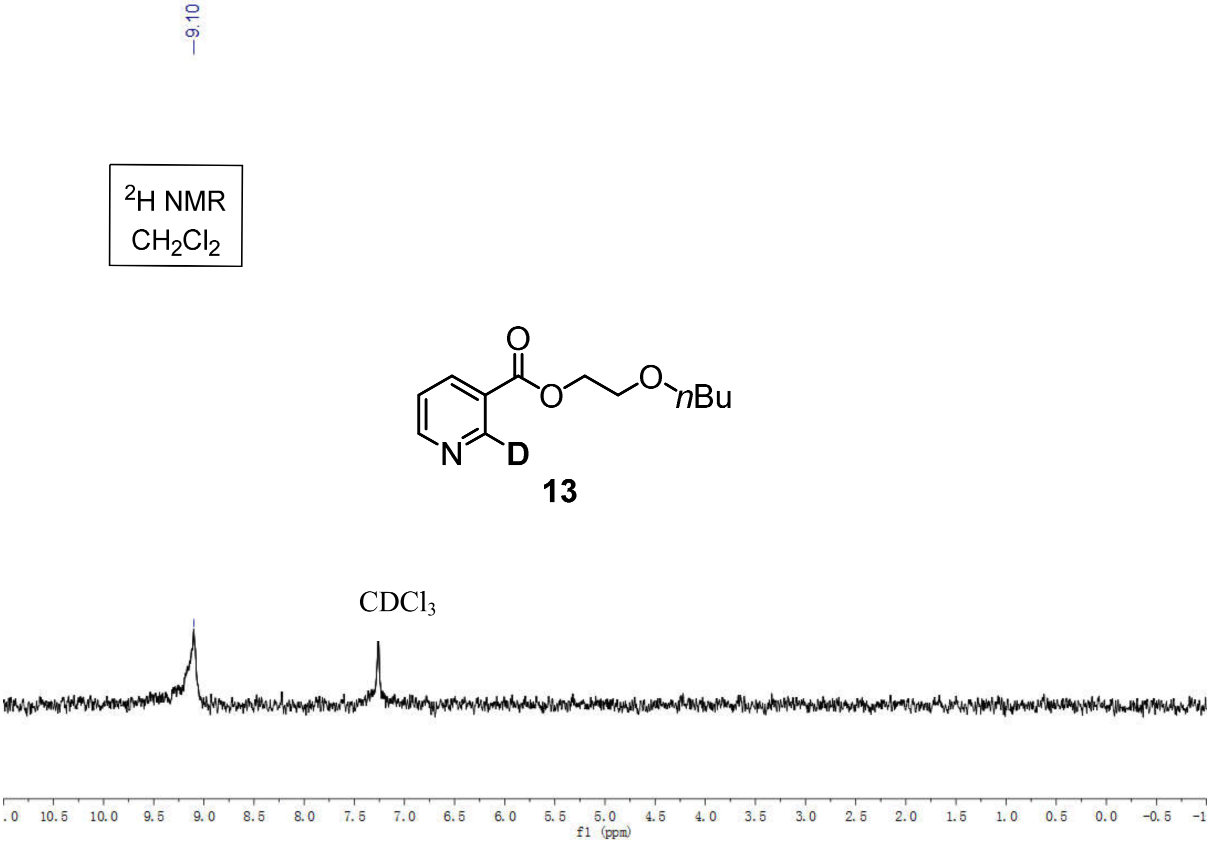

9.10
2H NMR
CH2Cl2
O
O
O
nBu
N
D
13
CDCl3
f1 (ppm)

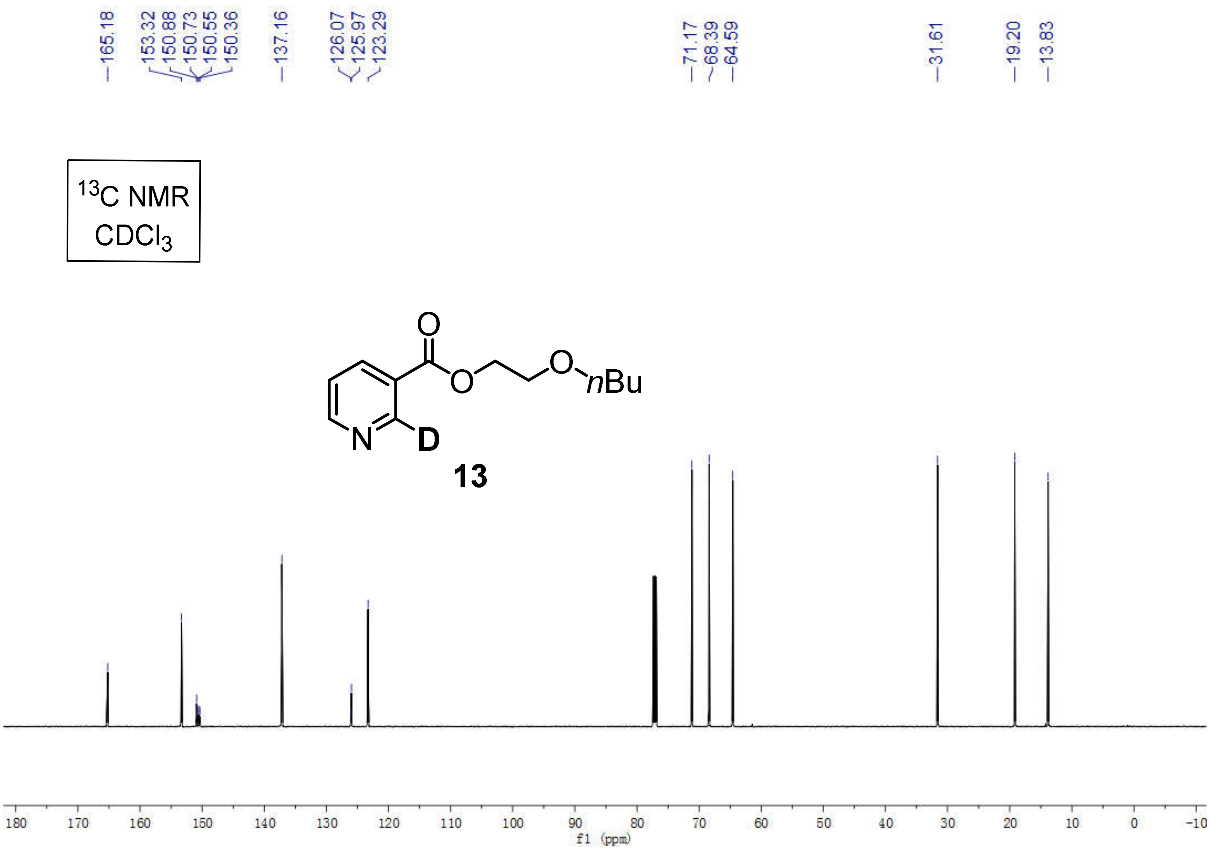


**Supplementary Fig. 116** $^{1}H$ NMR, $^{2}H$ NMR and $^{13}C$ NMR spectra of the compound **13**.

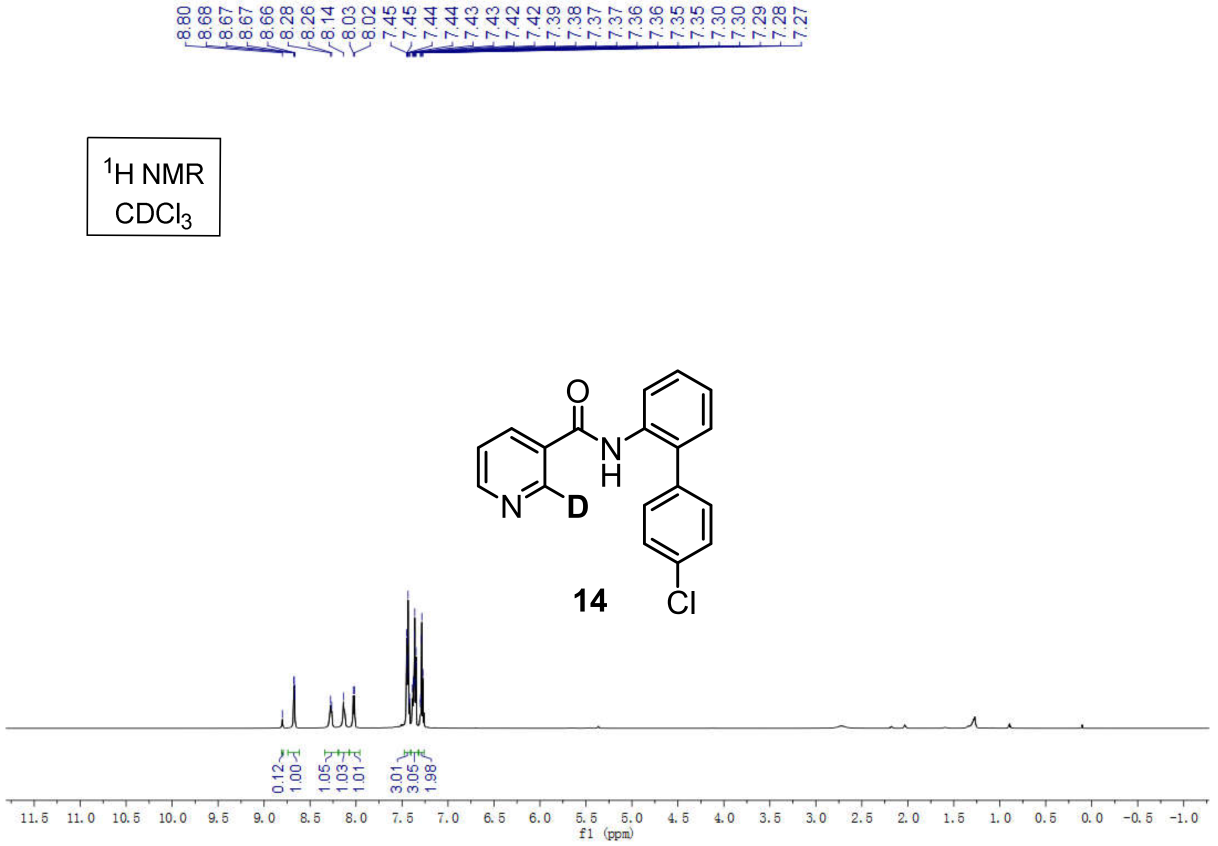

$^1$H NMR
$CDCl_3$
14
8.80 8.68 8.67 8.67 8.66 8.28 8.26 8.14 8.03 8.02 7.45 7.45 7.44 7.44 7.43 7.43 7.42 7.42 7.39 7.38 7.37 7.37 7.36 7.36 7.35 7.35 7.30 7.30 7.29 7.28 7.27
0.12 1.00 1.05 1.03 1.01 3.01 3.05 1.98
f1 (ppm)


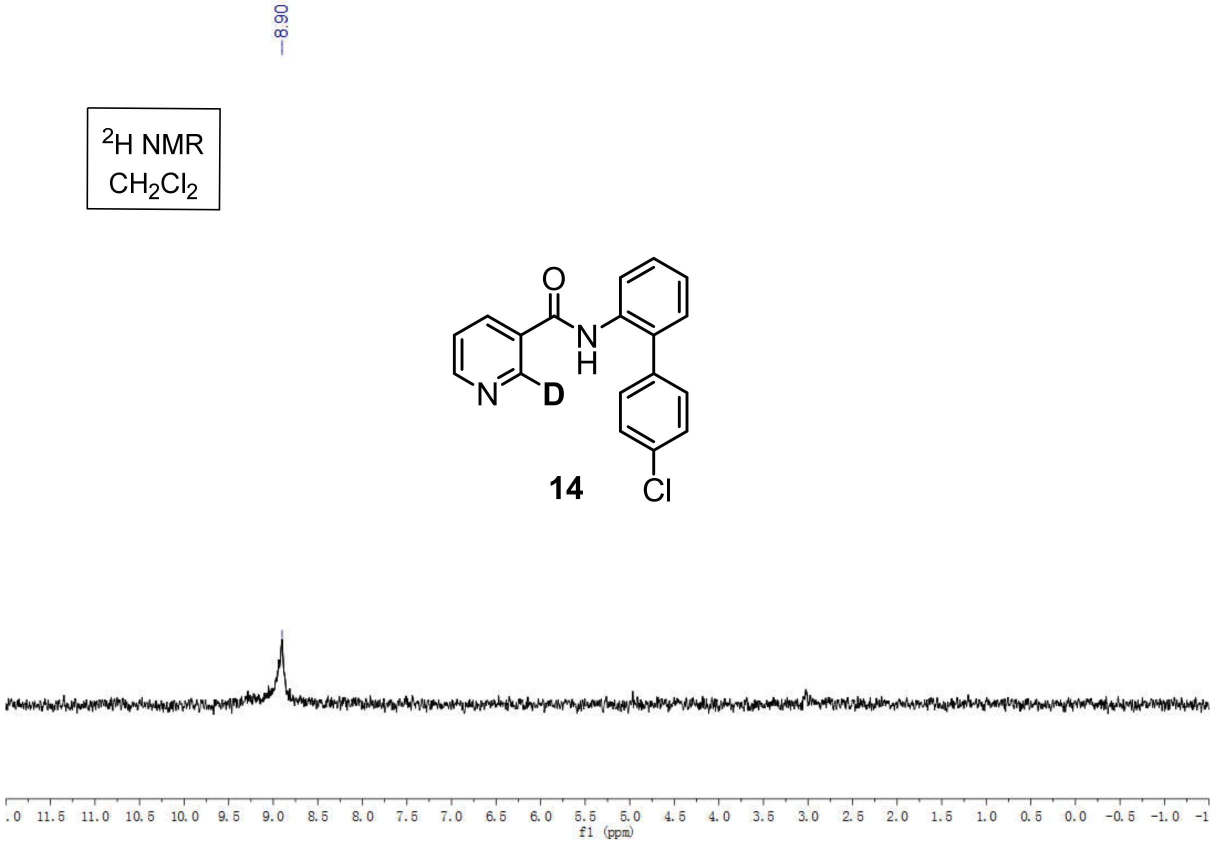

$^2$H NMR
$CH_2Cl_2$
14
8.90
f1 (ppm)

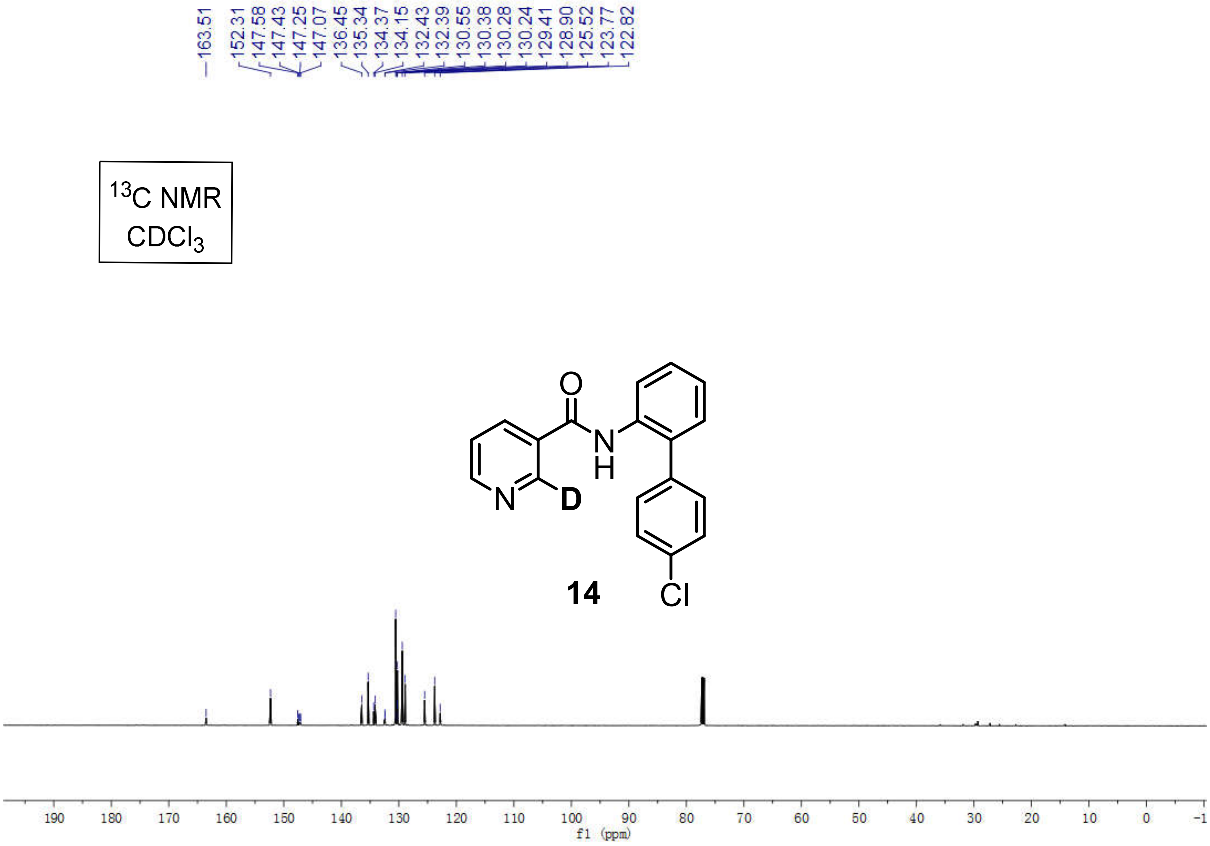


**Supplementary Fig. 117** $^{1}$H NMR, $^{2}$H NMR and $^{13}$C NMR spectra of the compound **14**.

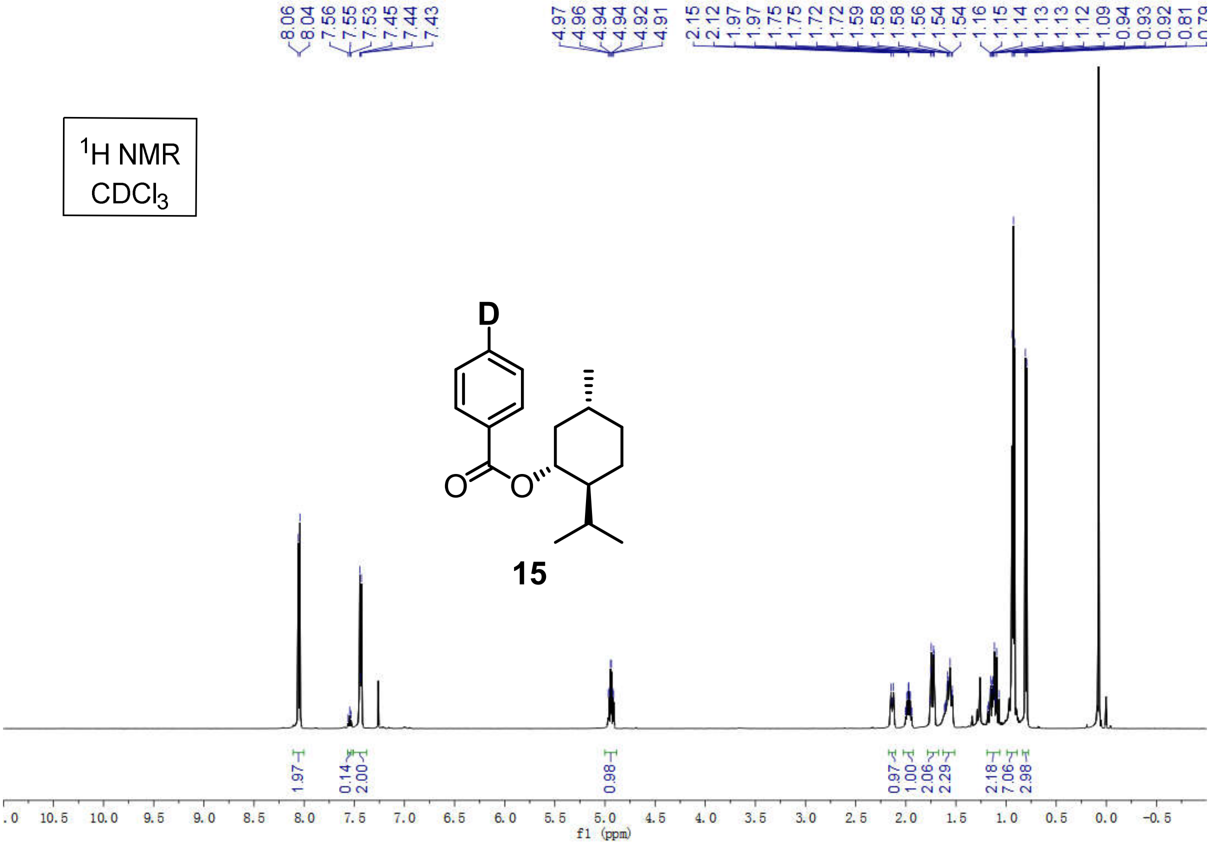

$^1$H NMR
$CDCl_3$
D
O
O
15
8.06
8.04
7.56
7.55
7.53
7.45
7.44
7.43
4.97
4.96
4.94
4.94
4.92
4.91
2.15
2.12
1.97
1.97
1.75
1.75
1.72
1.72
1.59
1.58
1.58
1.56
1.54
1.54
1.16
1.15
1.14
1.13
1.13
1.12
1.09
0.94
0.93
0.92
0.81
1.97
0.14
2.00
0.98
0.97
1.00
2.06
2.29
2.18
7.06
2.98
f1 (ppm)


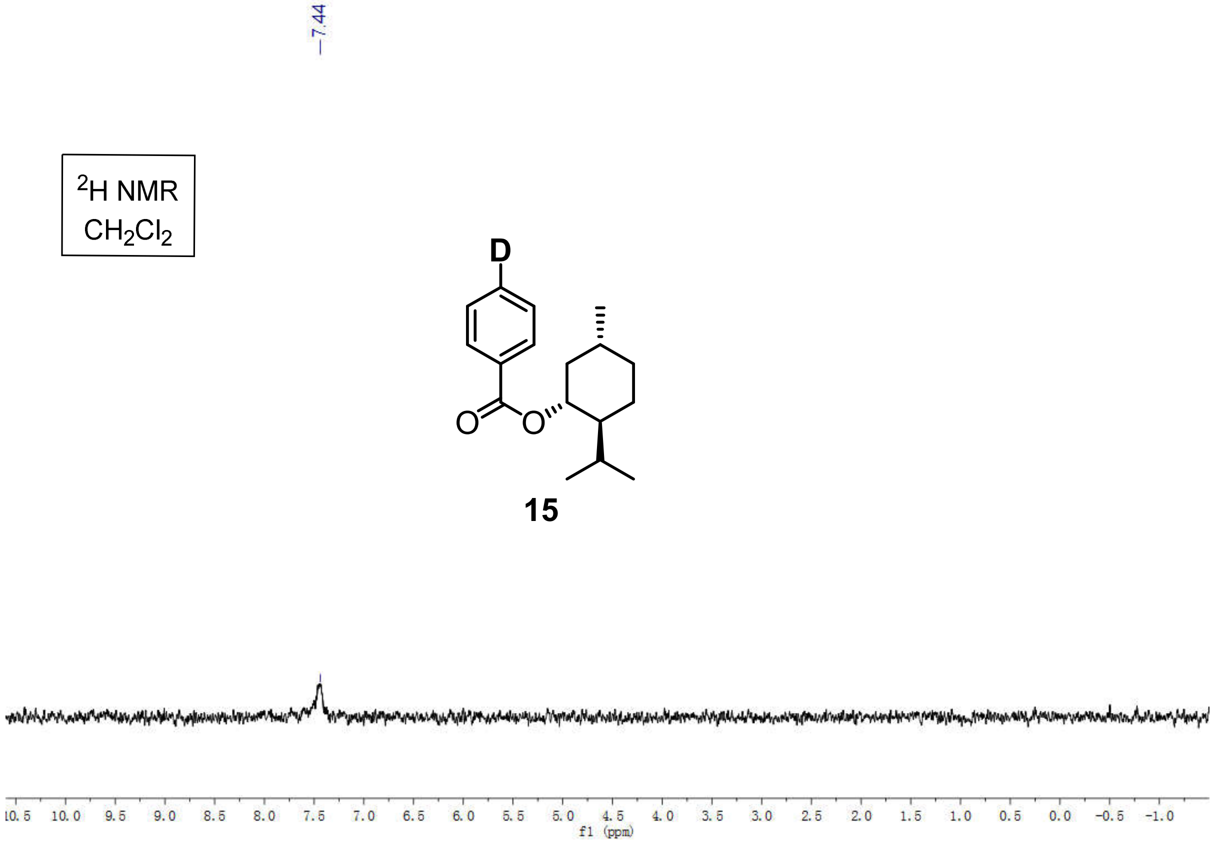

$^2$H NMR
$CH_2Cl_2$
D
O
O
15
7.44
f1 (ppm)

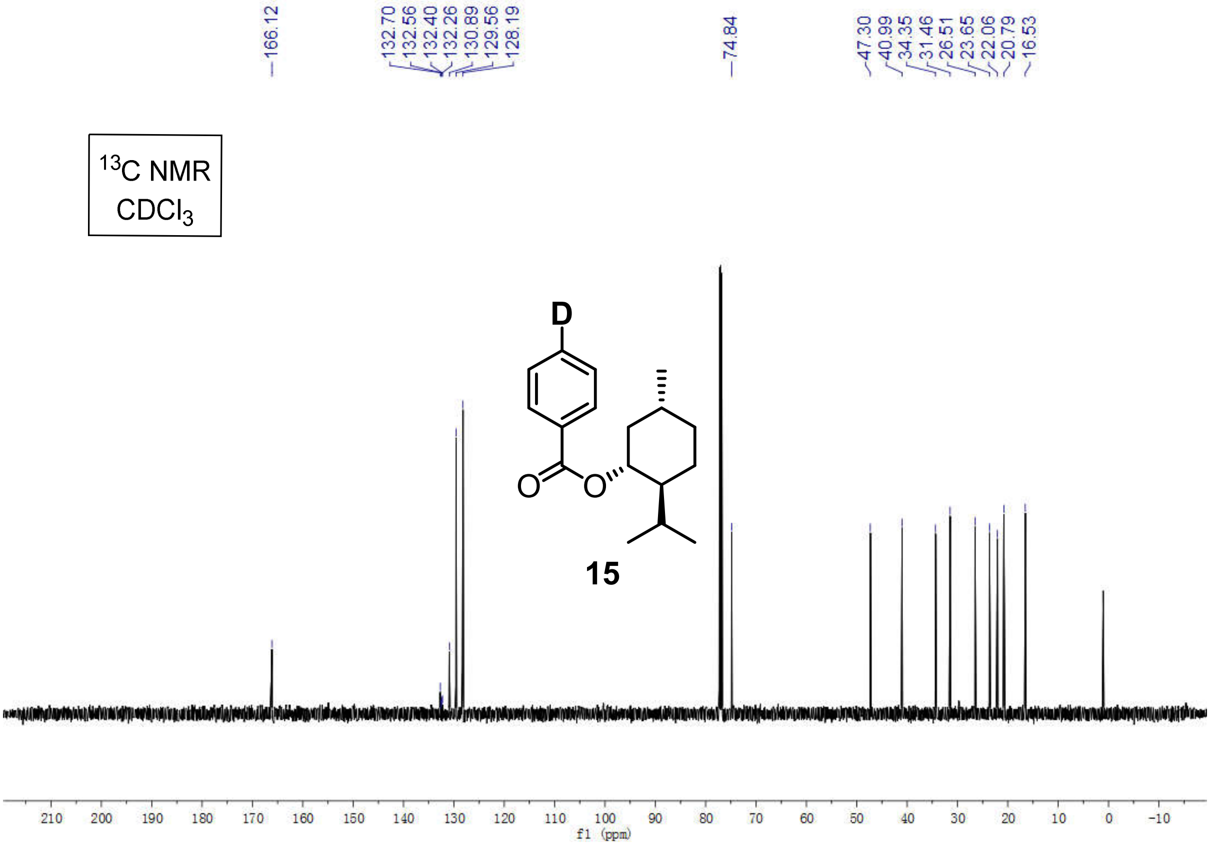


**Supplementary Fig. 118** $^{1}$H NMR, $^{2}$H NMR and $^{13}$C NMR spectra of the compound **15**.

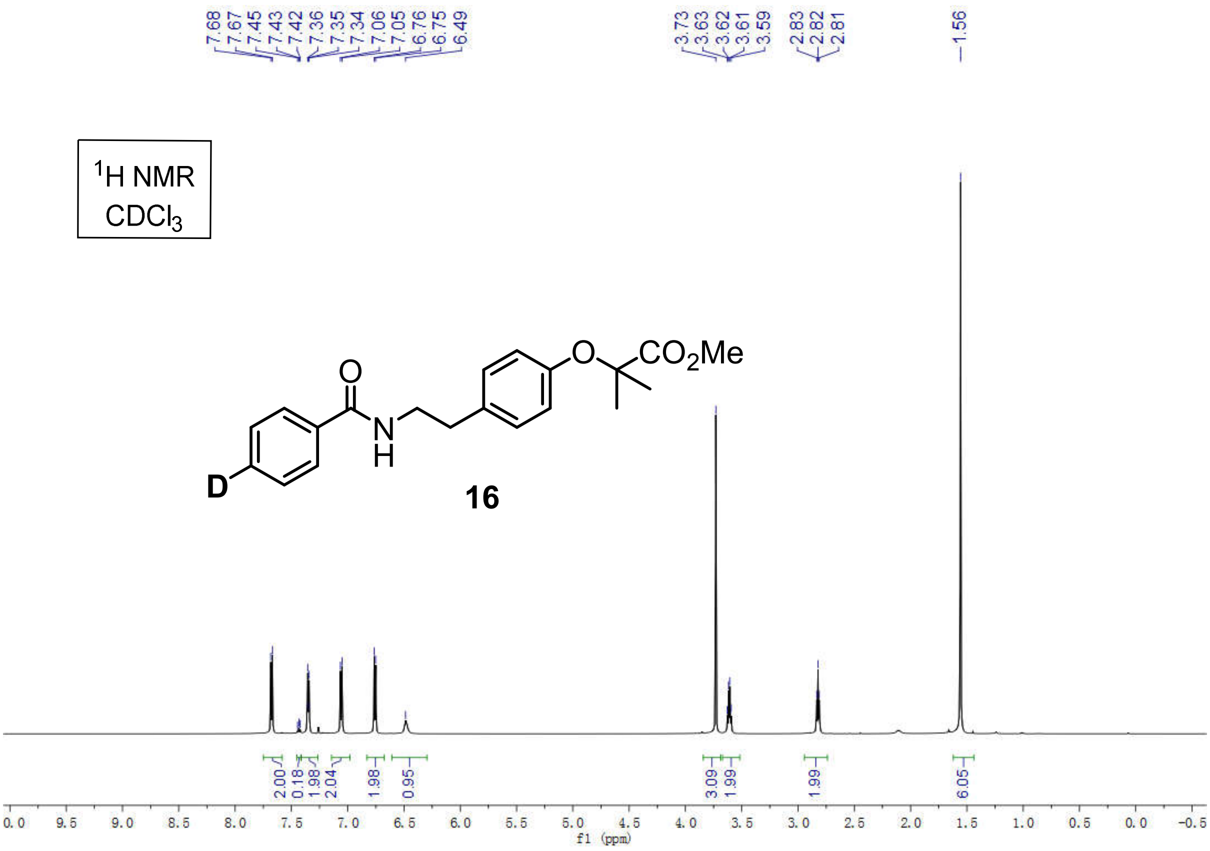

7.68
7.67
7.45
7.43
7.42
7.36
7.35
7.34
7.06
7.05
6.76
6.75
6.49
3.73
3.63
3.62
3.61
3.59
2.83
2.82
2.81
1.56
$^1$H NMR
$CDCl_3$
$CO_2Me$
O
N
H
D
16
2.00
0.18
1.98
2.04
1.98
0.95
3.09
1.99
1.99
6.05
f1 (ppm)


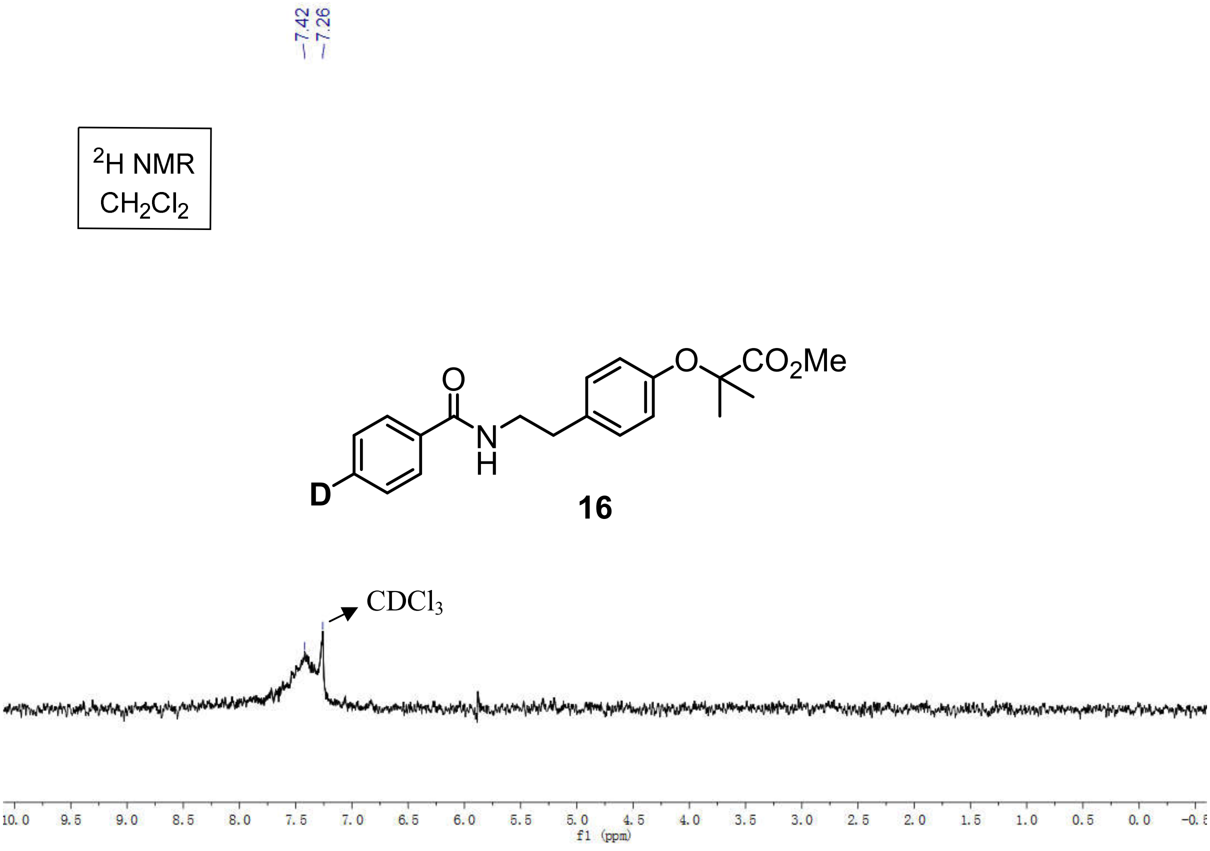

7.42
7.26
$^2$H NMR
$CH_2Cl_2$
$CO_2Me$
O
N
H
D
16
$CDCl_3$
f1 (ppm)

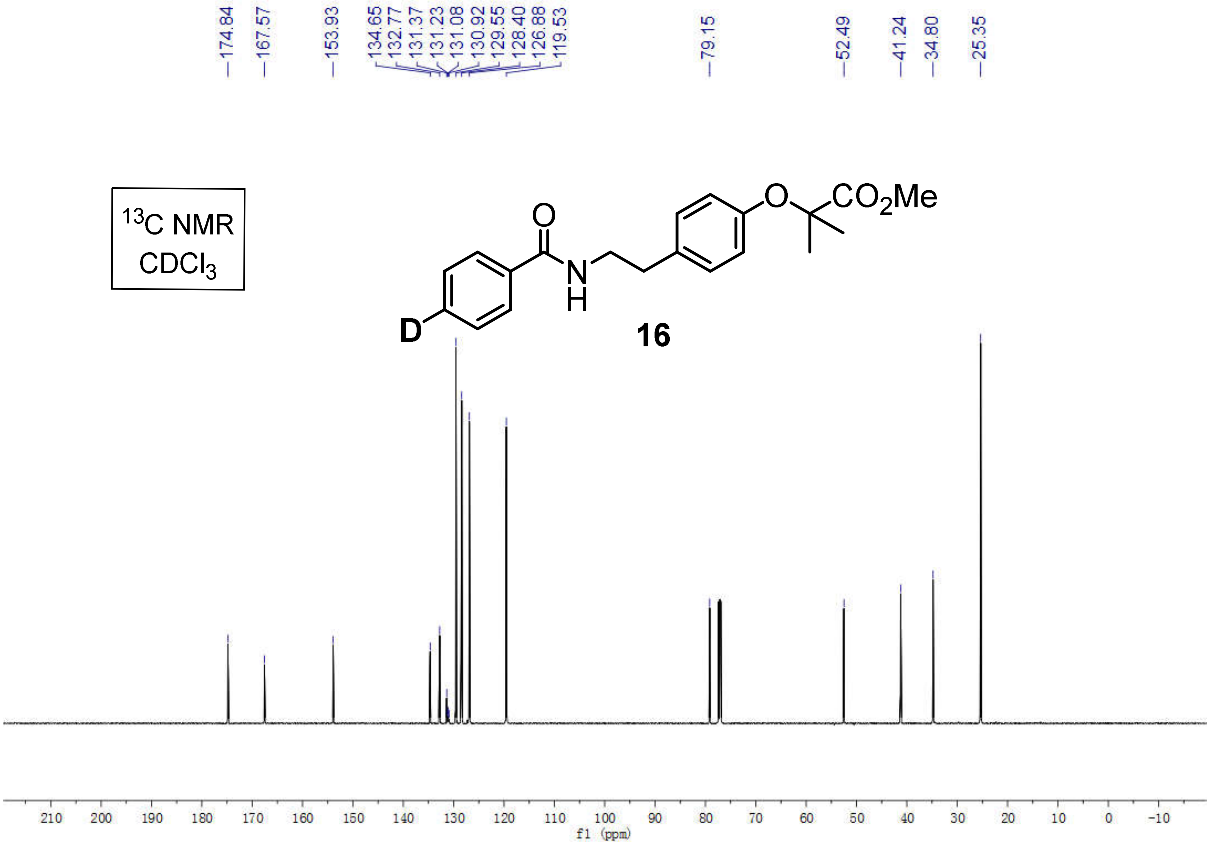


**Supplementary Fig. 119** $^{1}$H NMR, $^{2}$H NMR and $^{13}$C NMR spectra of the compound **16**.

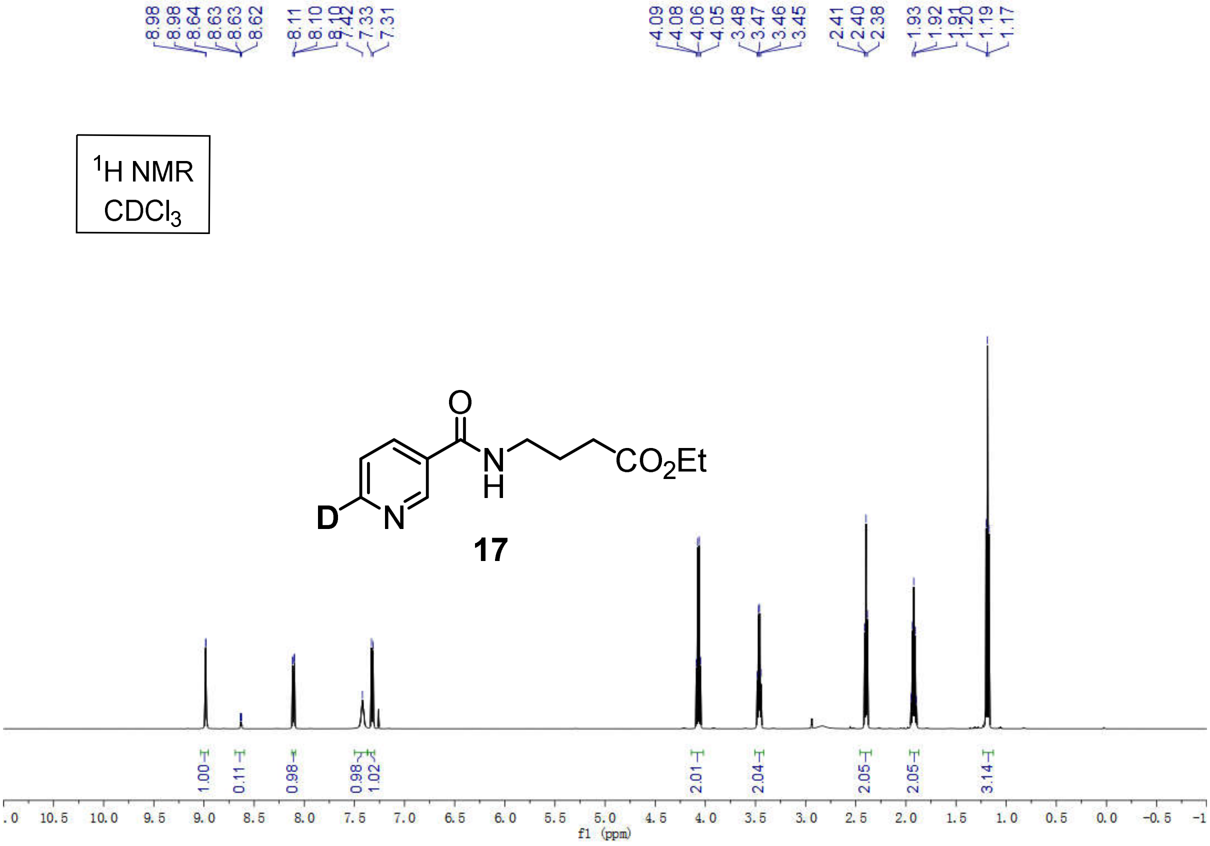
8.98 8.98 8.64 8.63 8.63 8.62 8.11 8.10 8.10 7.42 7.33 7.31
4.09 4.08 4.06 4.05 3.48 3.47 3.46 3.45 2.41 2.40 2.38 1.93 1.92 1.91 1.20 1.19 1.17
1H NMR
CDCl3
O
N
H
CO2Et
D
N
17
1.00 0.11 0.98 0.98 1.02 2.01 2.04 2.05 2.05 3.14
f1 (ppm)

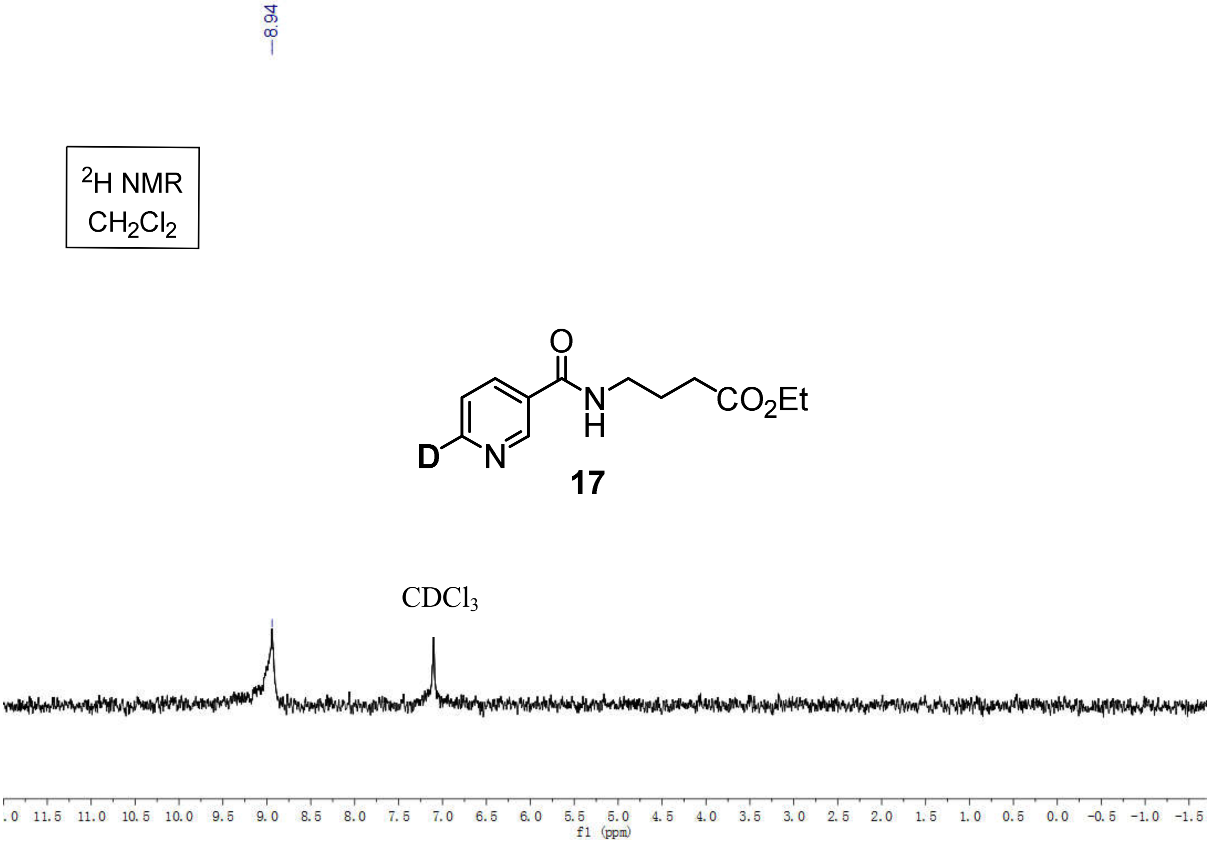
8.94
2H NMR
CH2Cl2
O
N
H
CO2Et
D
N
17
CDCl3
f1 (ppm)

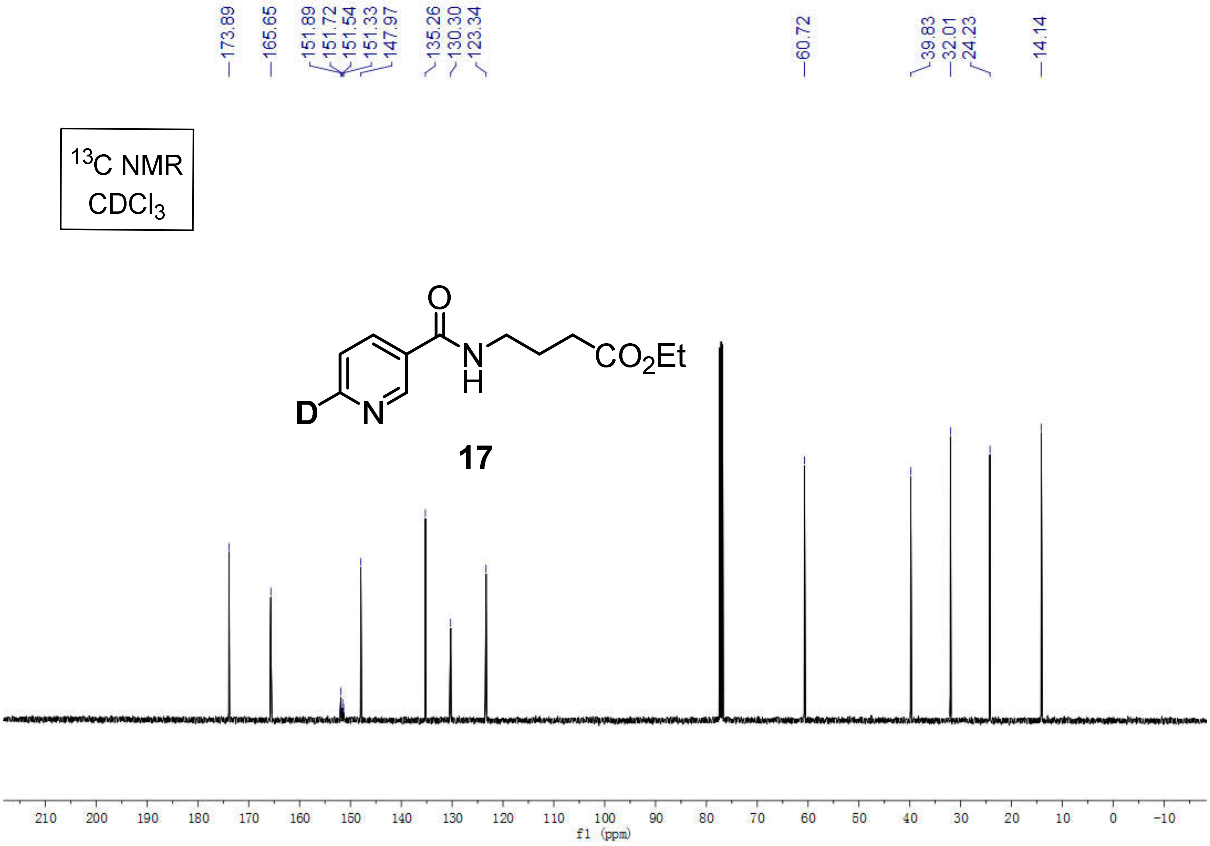


**Supplementary Fig. 120** $^{1}$H NMR, $^{2}$H NMR and $^{13}$C NMR spectra of the compound **17**.

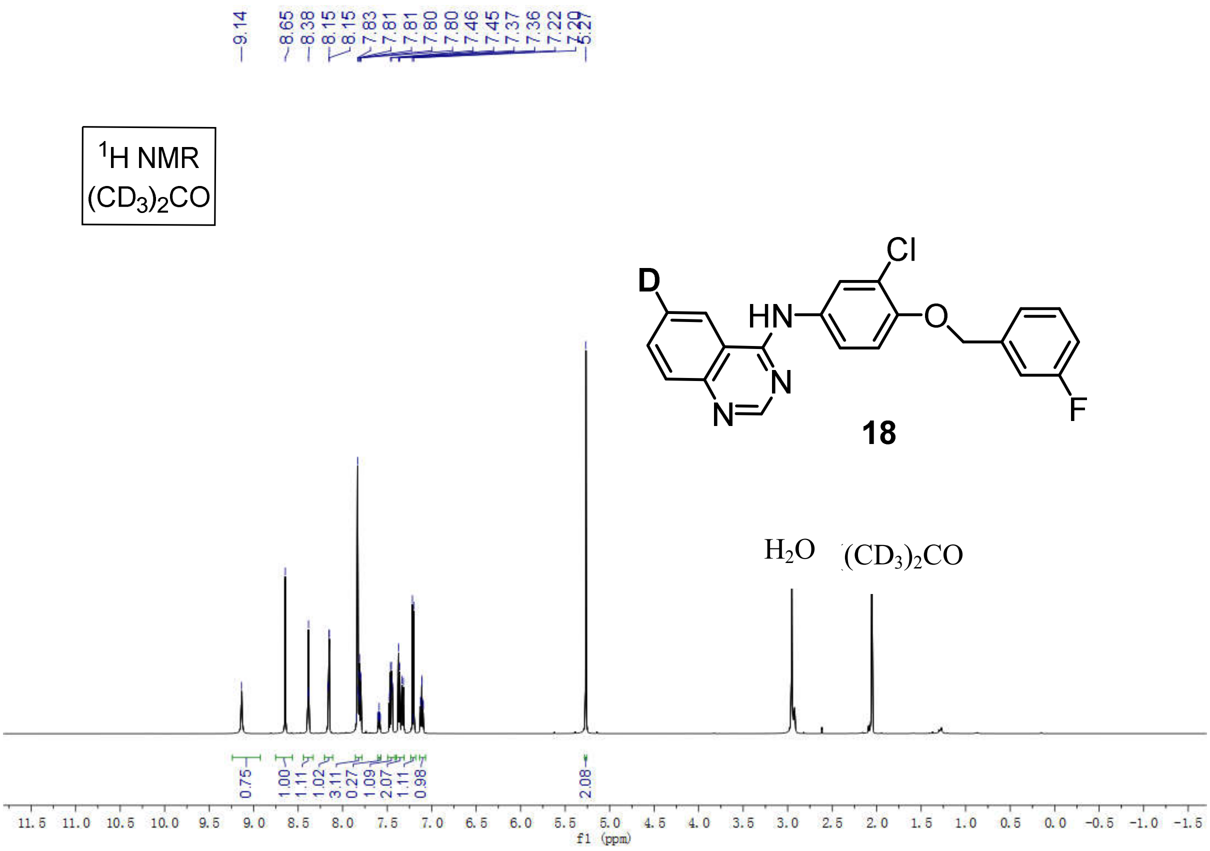
1H NMR
(CD3)2CO
9.14
8.65
8.38
8.15
8.15
7.83
7.81
7.81
7.80
7.80
7.46
7.45
7.37
7.36
7.22
7.20
5.27
D
HN
Cl
O
N
N
F
18
H2O
(CD3)2CO
0.75
1.00
1.11
1.02
3.11
0.27
1.09
2.07
1.11
0.98
2.08
f1 (ppm)

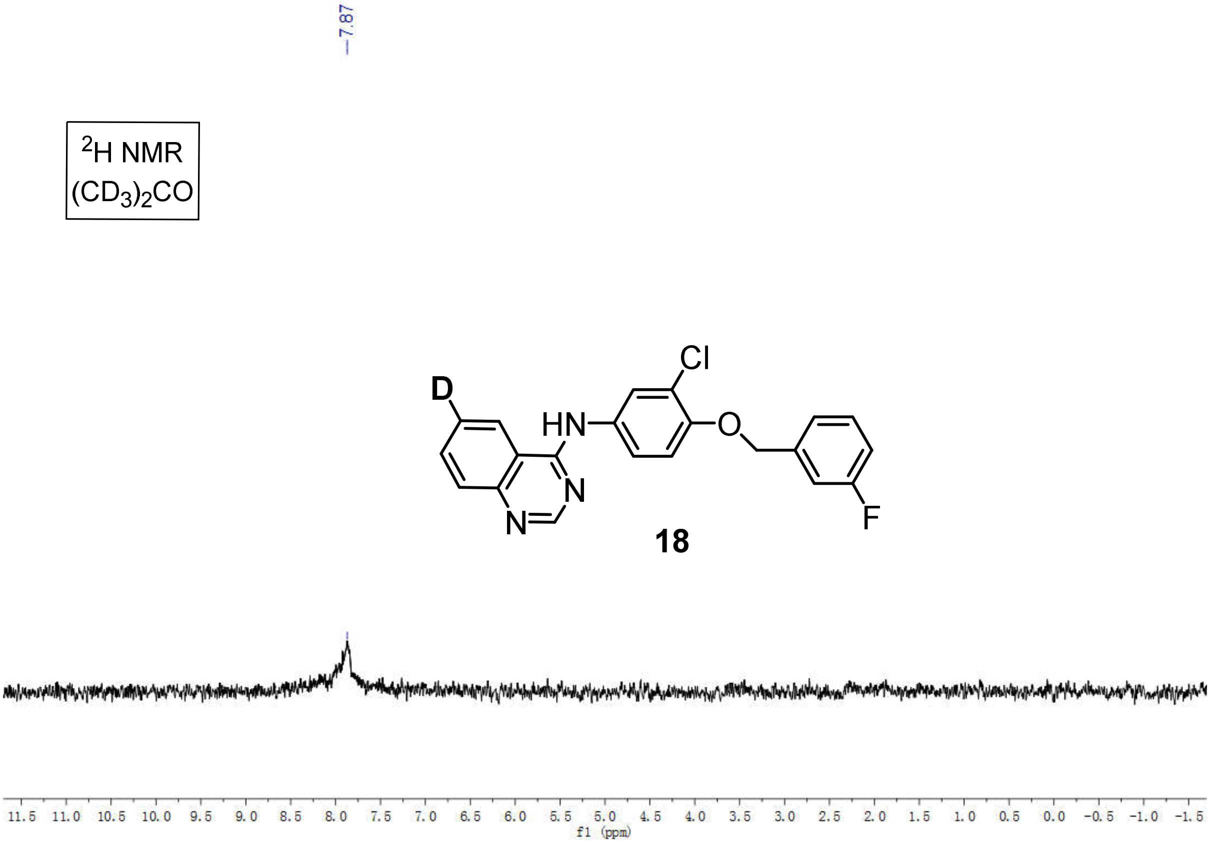
7.87
2H NMR
(CD3)2CO
D
HN
Cl
O
N
N
F
18
f1 (ppm)

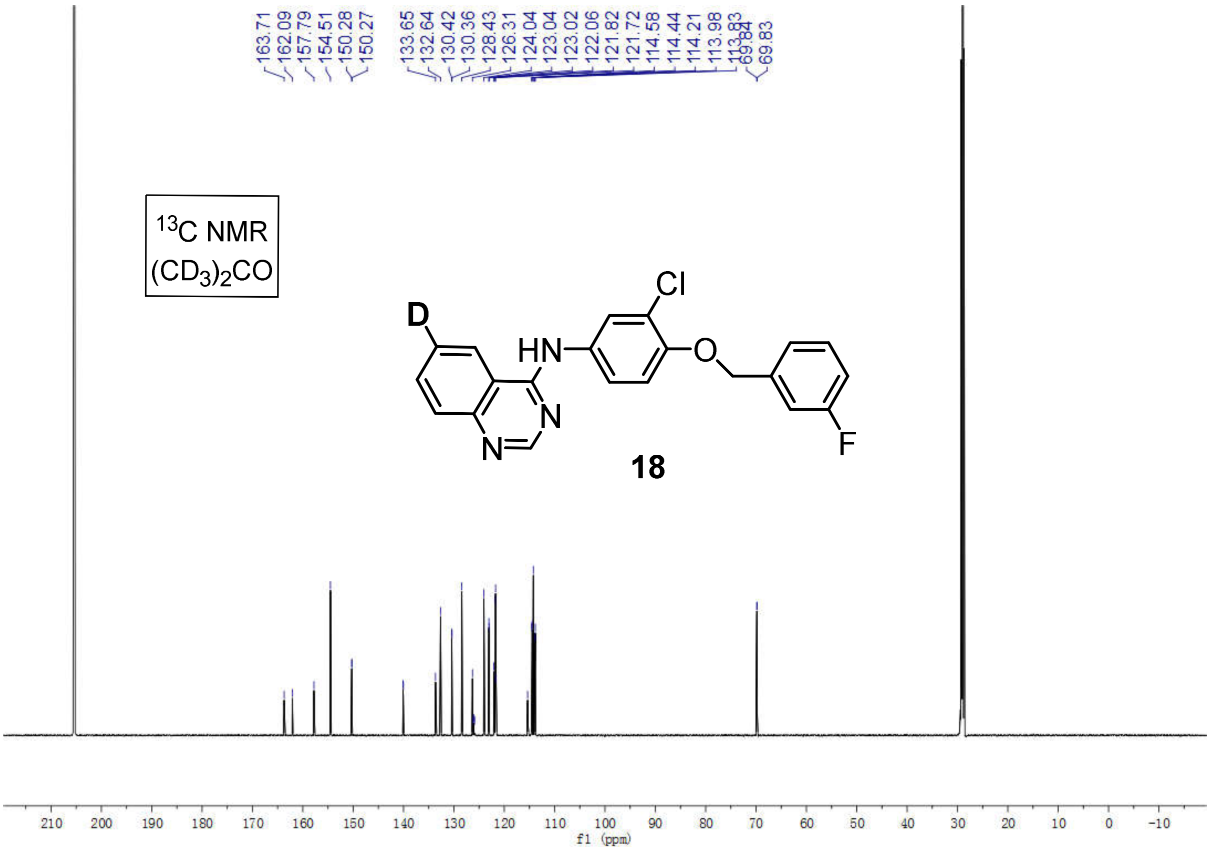


**Supplementary Fig. 121** $^{1}$H NMR, $^{2}$H NMR and $^{13}$C NMR spectra of the compound **18**.

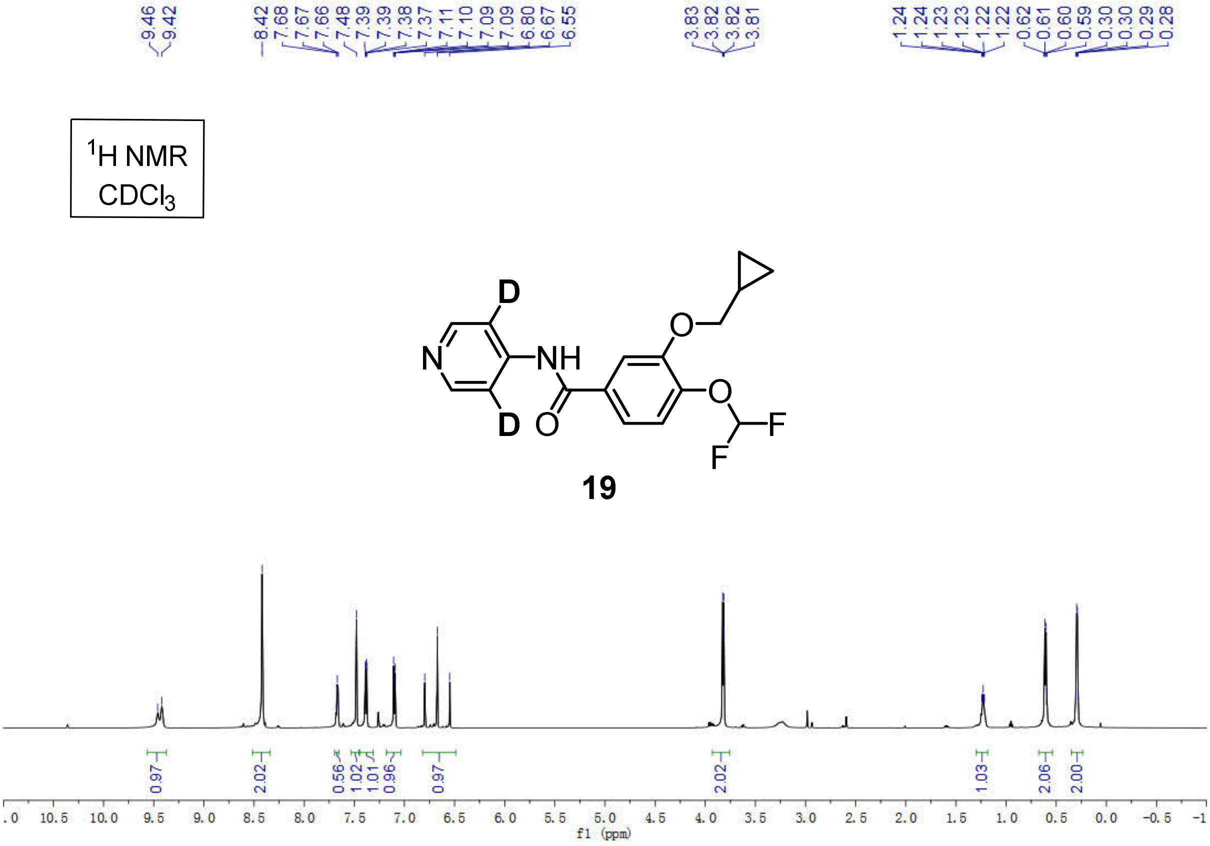
1H NMR
CDCl3
19
f1 (ppm)

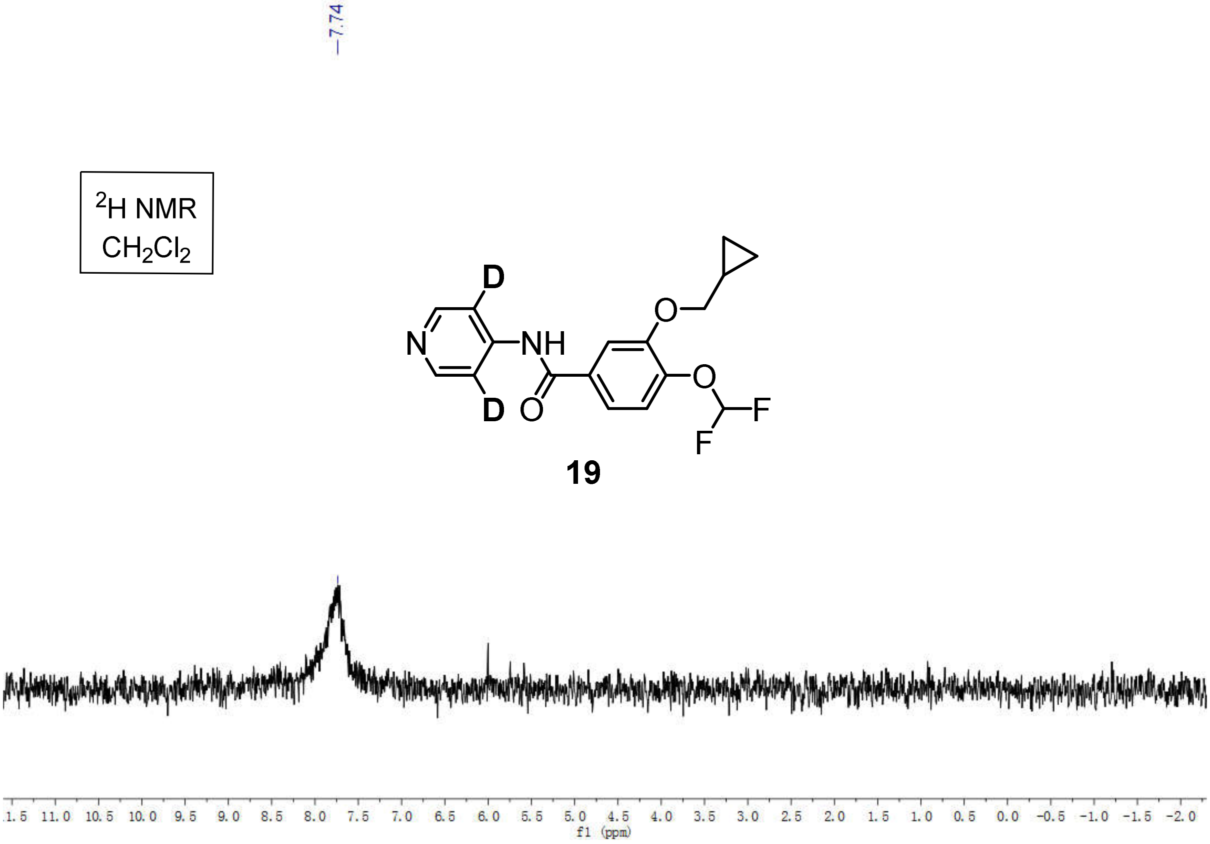
7.74
2H NMR
CH2Cl2
19
f1 (ppm)

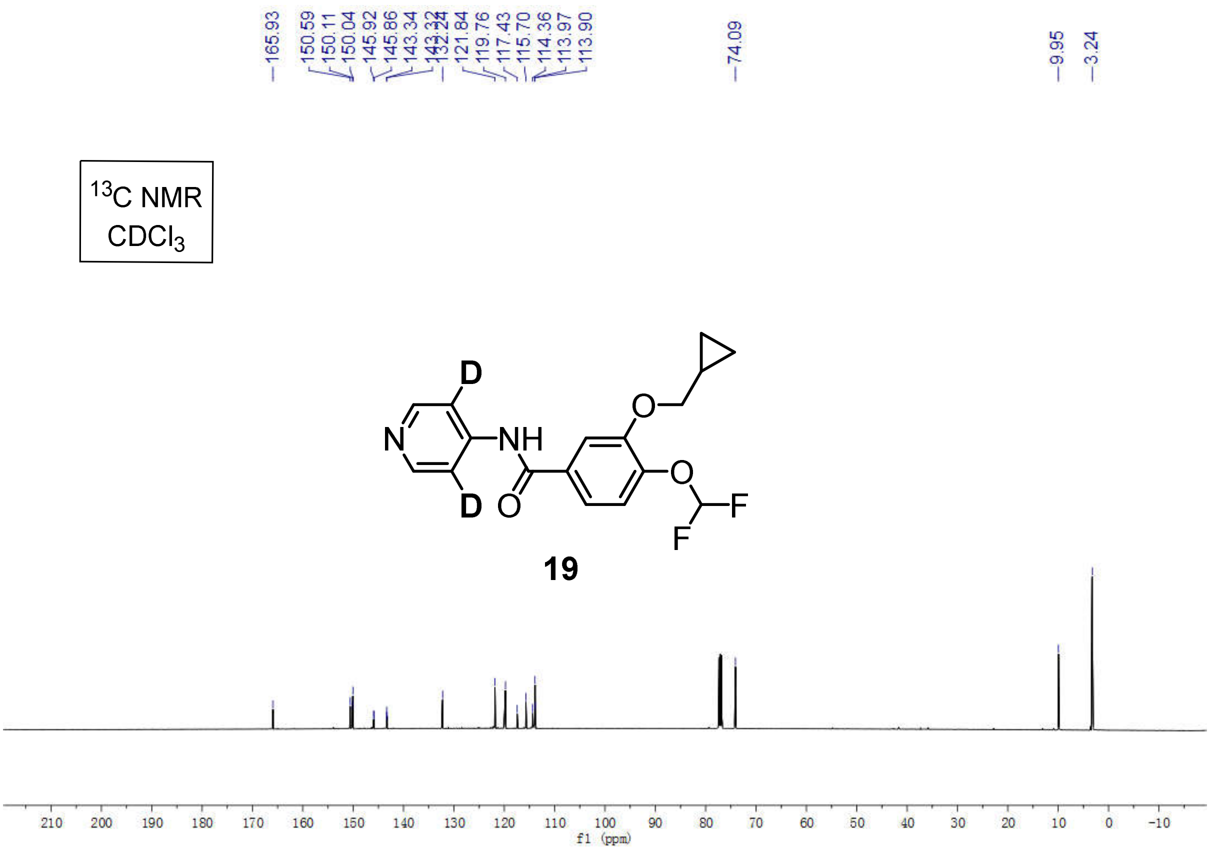


**Supplementary Fig. 122** $^{1}$H NMR, $^{2}$H NMR and $^{13}$C NMR spectra of the compound **19**.

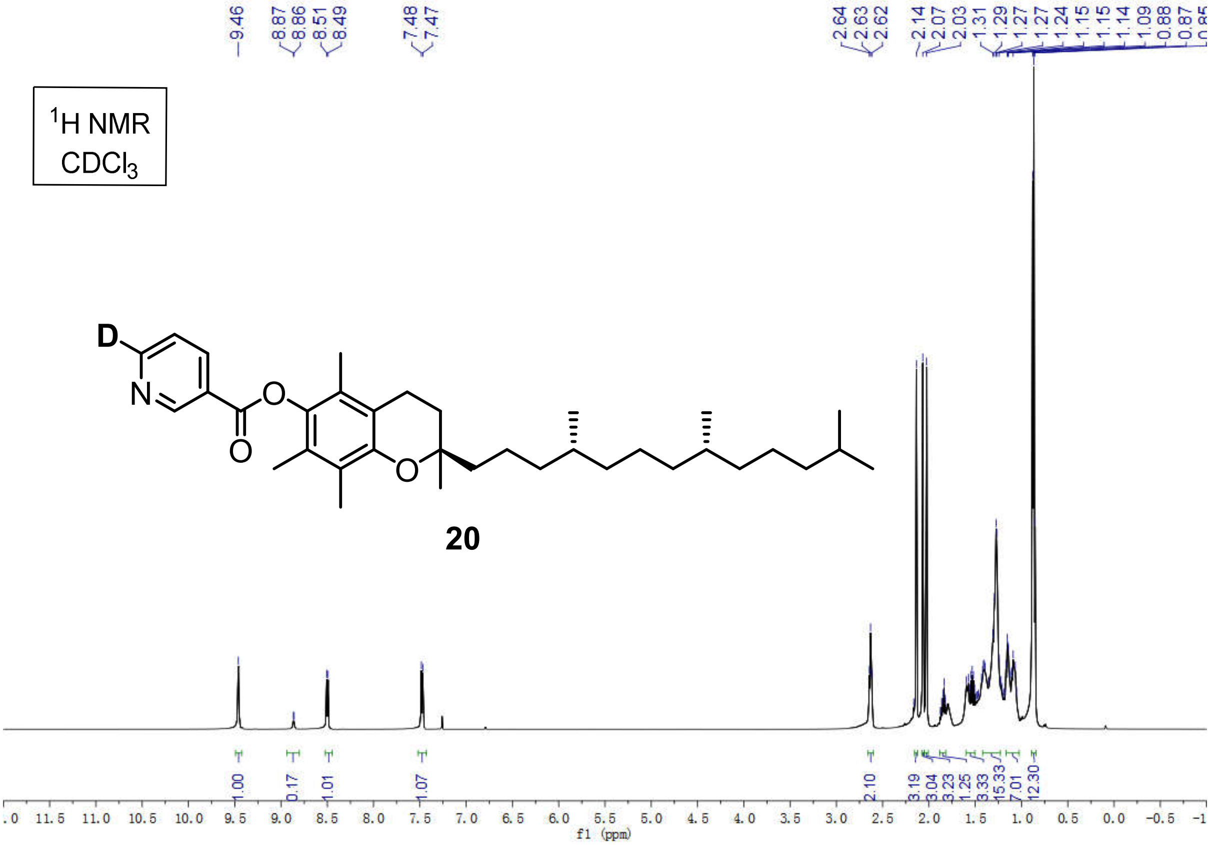

9.46
8.87
8.86
8.51
8.49
7.48
7.47
2.64
2.63
2.62
2.14
2.07
2.03
1.31
1.29
1.27
1.27
1.24
1.15
1.15
1.14
1.09
0.88
0.87
1H NMR
CDCl3
D
N
O
O
O
20
1.00
0.17
1.01
1.07
2.10
3.19
3.04
3.23
1.25
3.33
15.33
7.01
12.30
f1 (ppm)


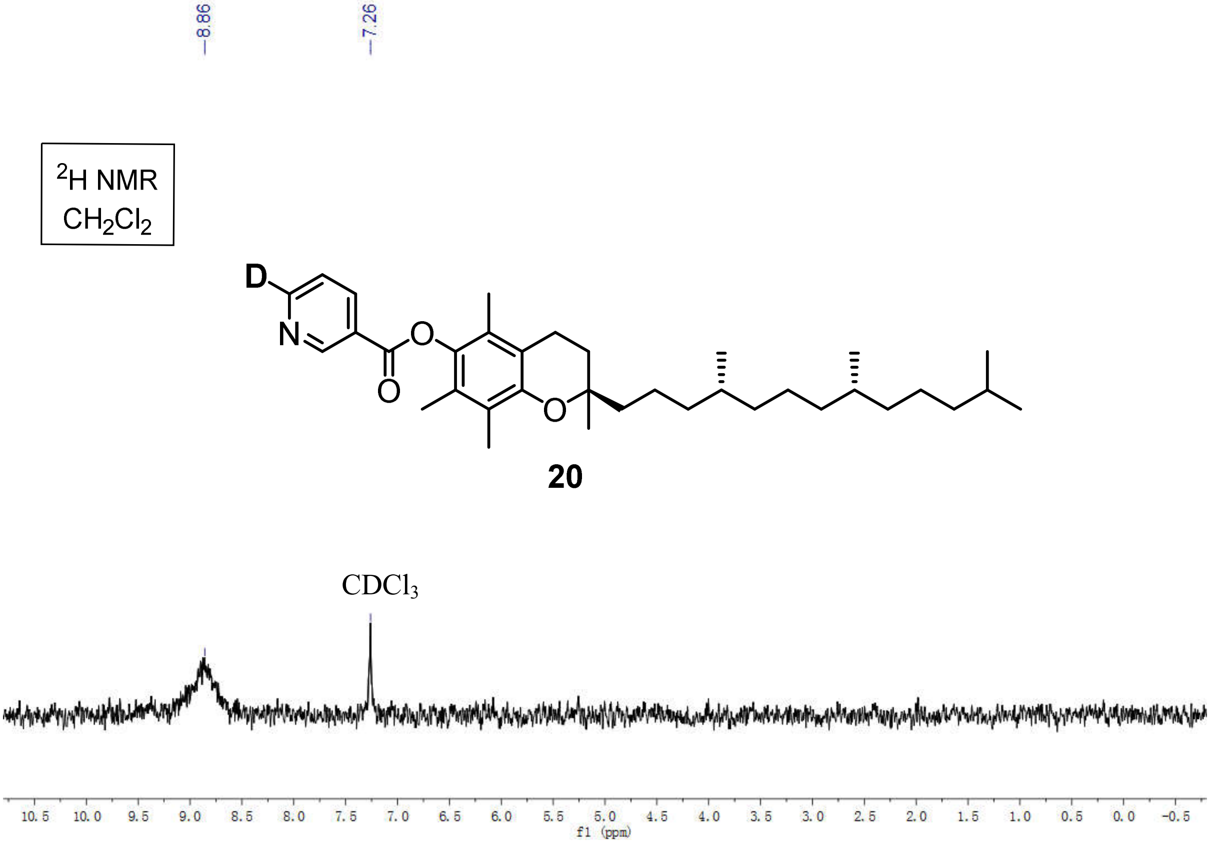

8.86
7.26
2H NMR
CH2Cl2
D
N
O
O
O
20
CDCl3
f1 (ppm)

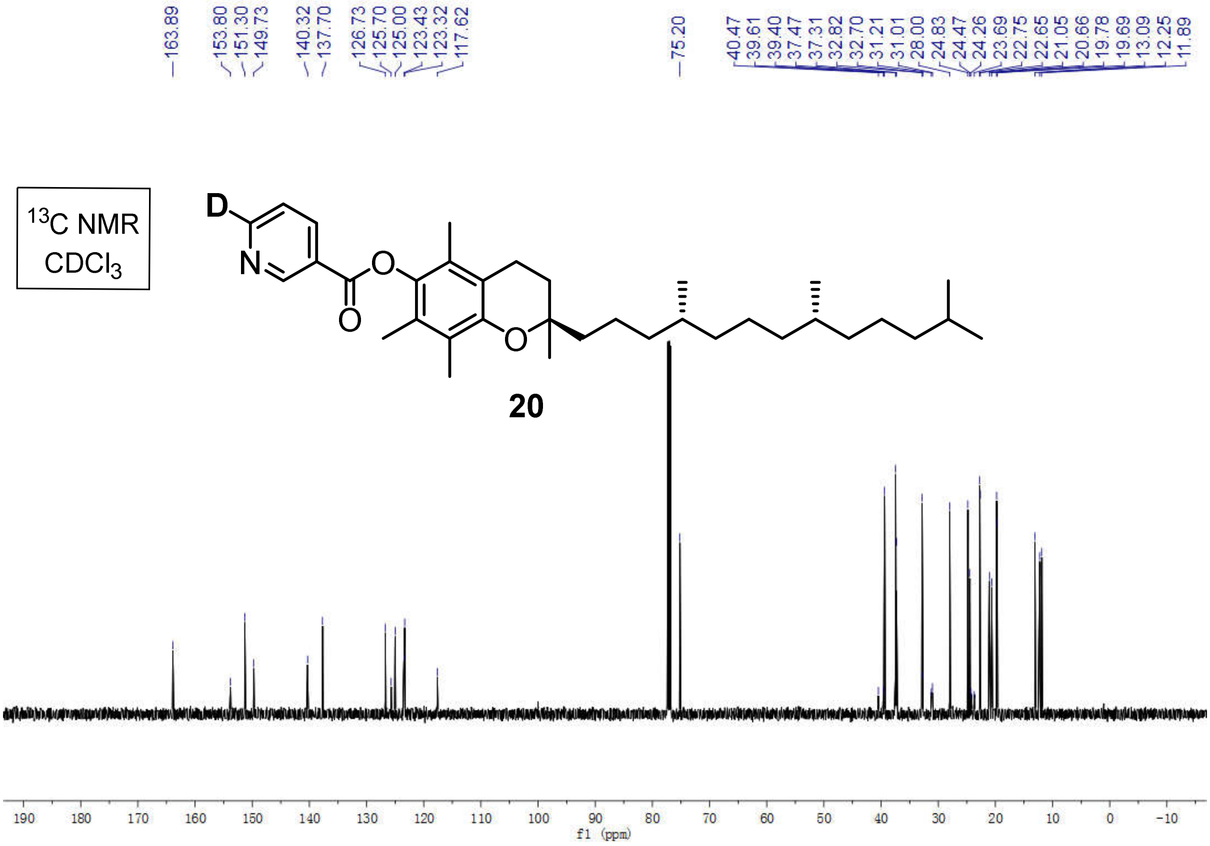


**Supplementary Fig. 123** $^{1}$H NMR, $^{2}$H NMR and $^{13}$C NMR spectra of the compound **20**.

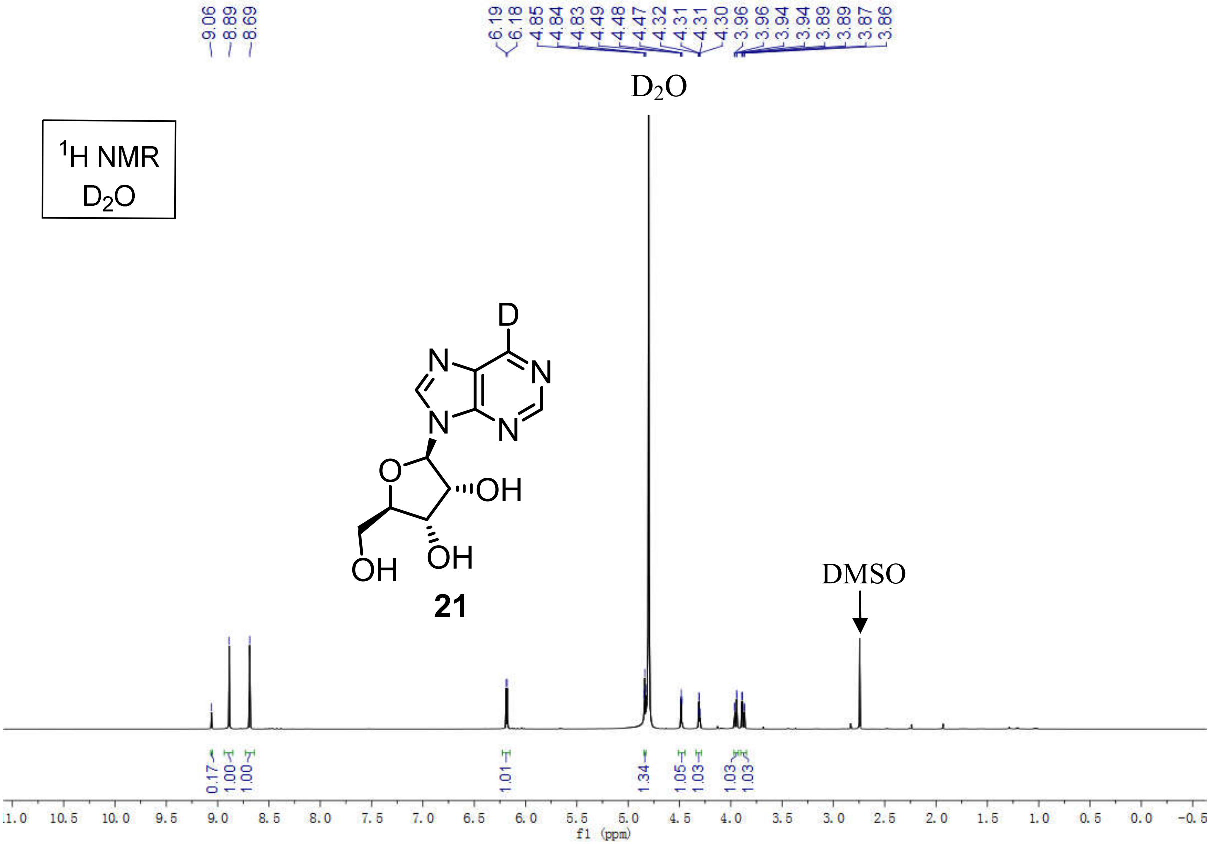
1H NMR
D2O
D2O
DMSO
21
f1 (ppm)

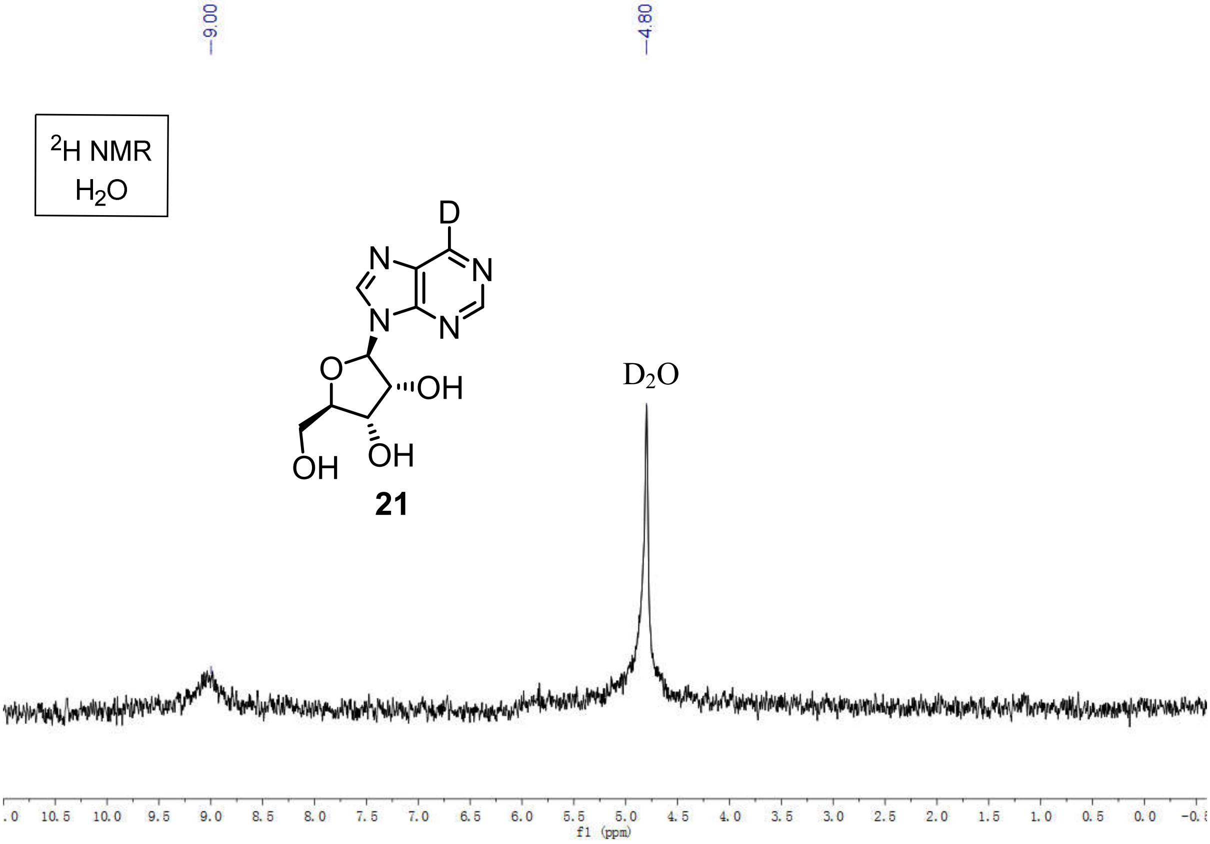
2H NMR
H2O
D2O
21
f1 (ppm)

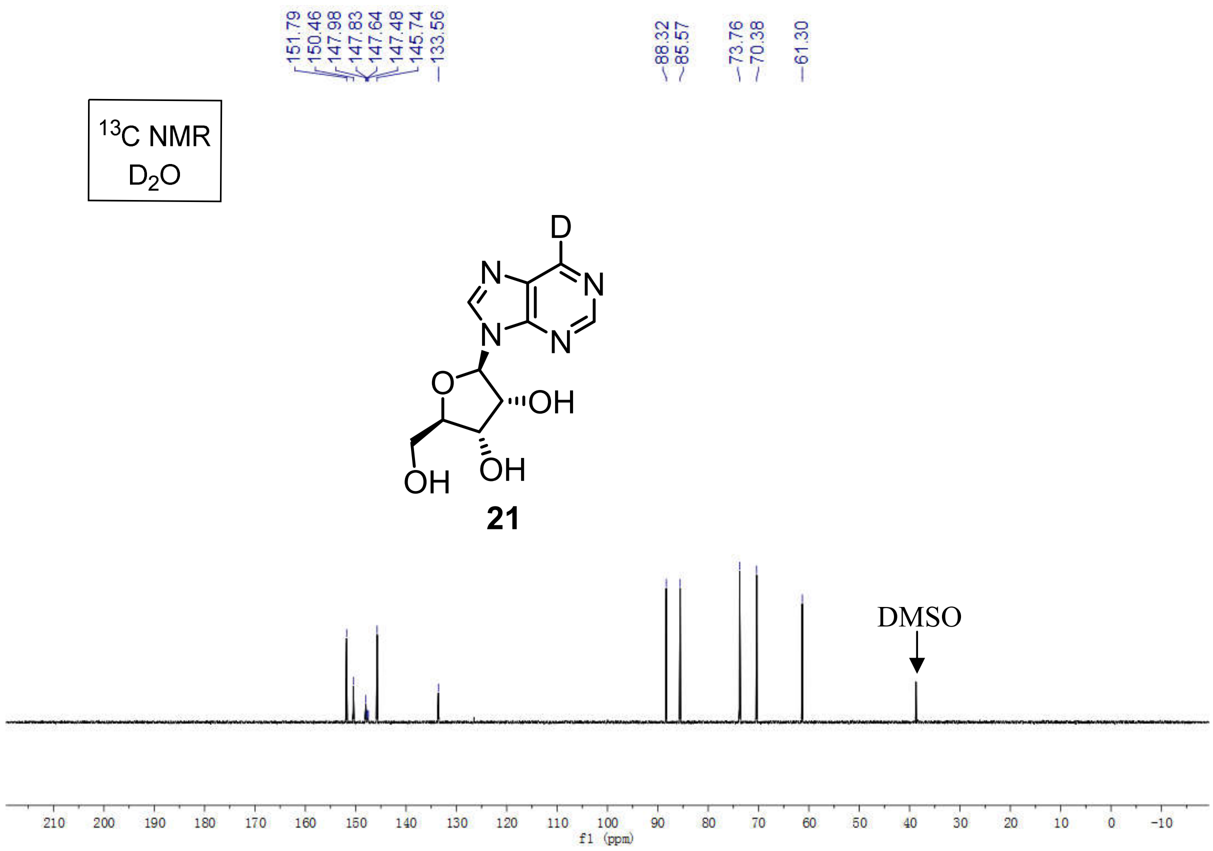


**Supplementary Fig. 124** $^{1}$H NMR, $^{2}$H NMR and $^{13}$C NMR spectra of the compound **21**.

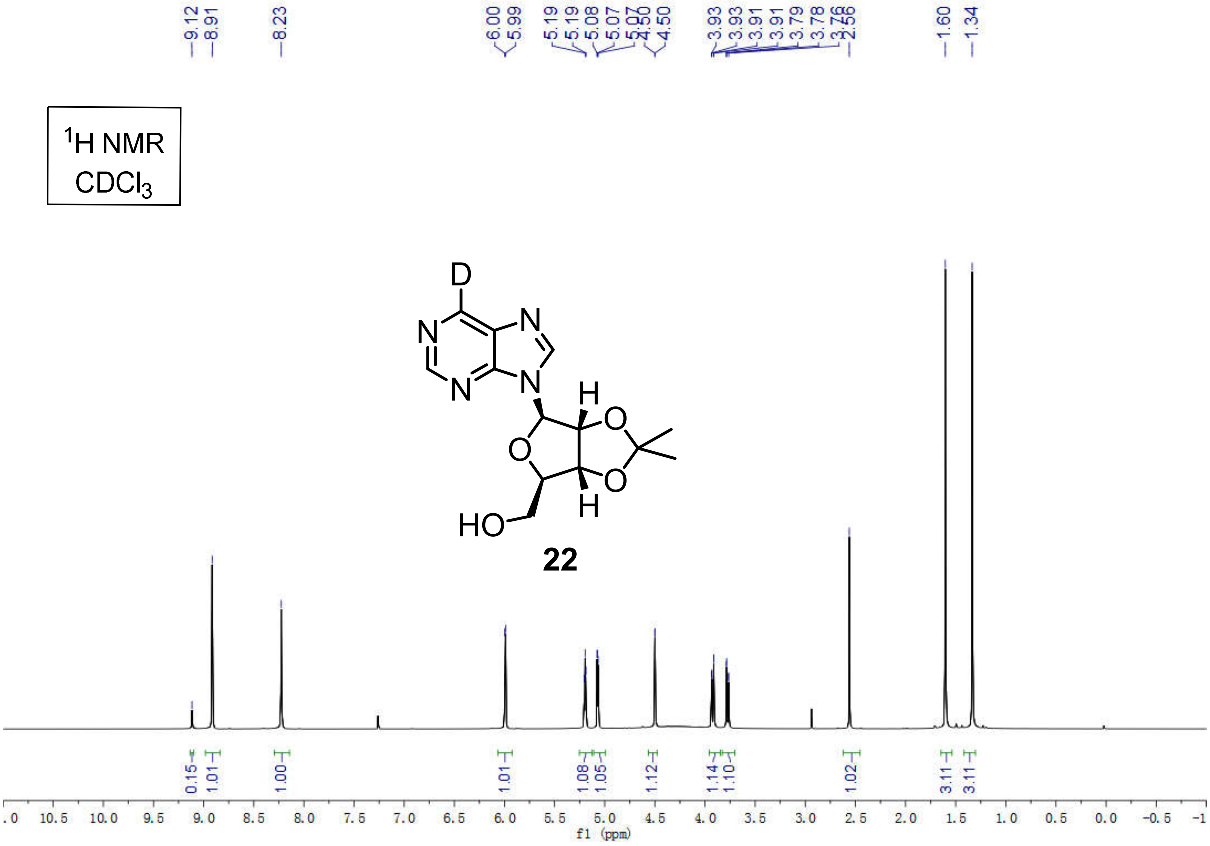

9.12
8.91
8.23
6.00
5.99
5.19
5.19
5.08
5.07
5.07
4.50
4.50
3.93
3.93
3.91
3.91
3.79
3.78
3.76
2.56
1.60
1.34
$^1$H NMR
$CDCl_3$
22
0.15
1.01
1.00
1.01
1.08
1.05
1.12
1.14
1.10
1.02
3.11
3.11
f1 (ppm)


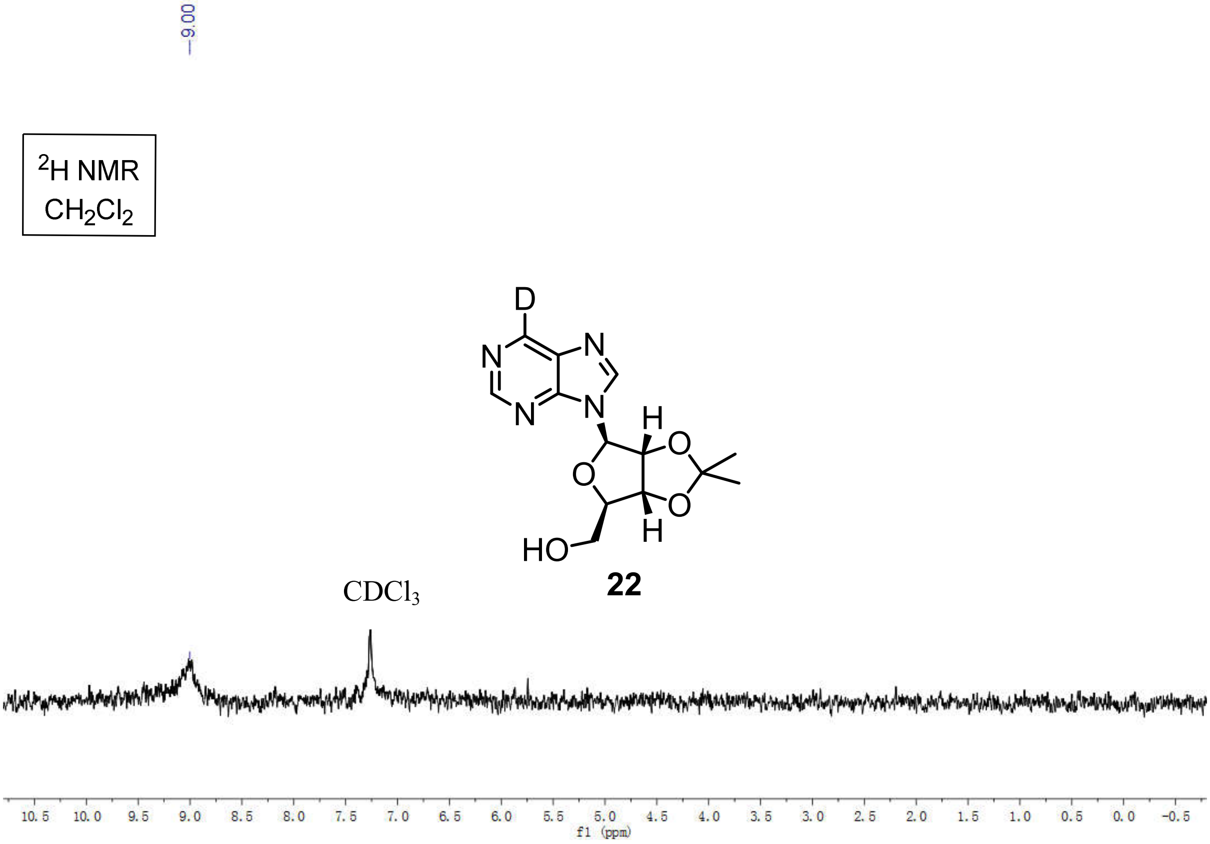

9.00
$^2$H NMR
$CH_2Cl_2$
22
$CDCl_3$
f1 (ppm)

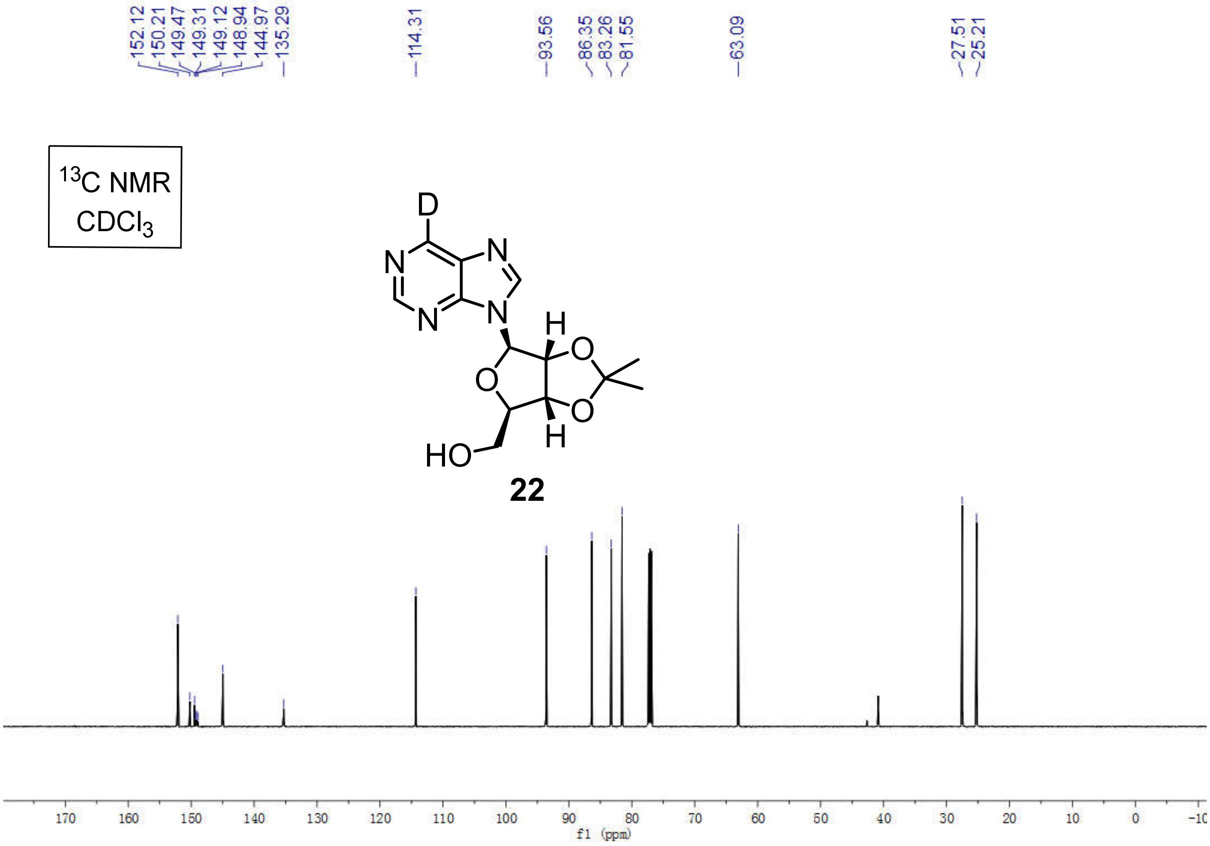


**Supplementary Fig. 125** $^{1}$H NMR, $^{2}$H NMR and $^{13}$C NMR spectra of the compound **22**.

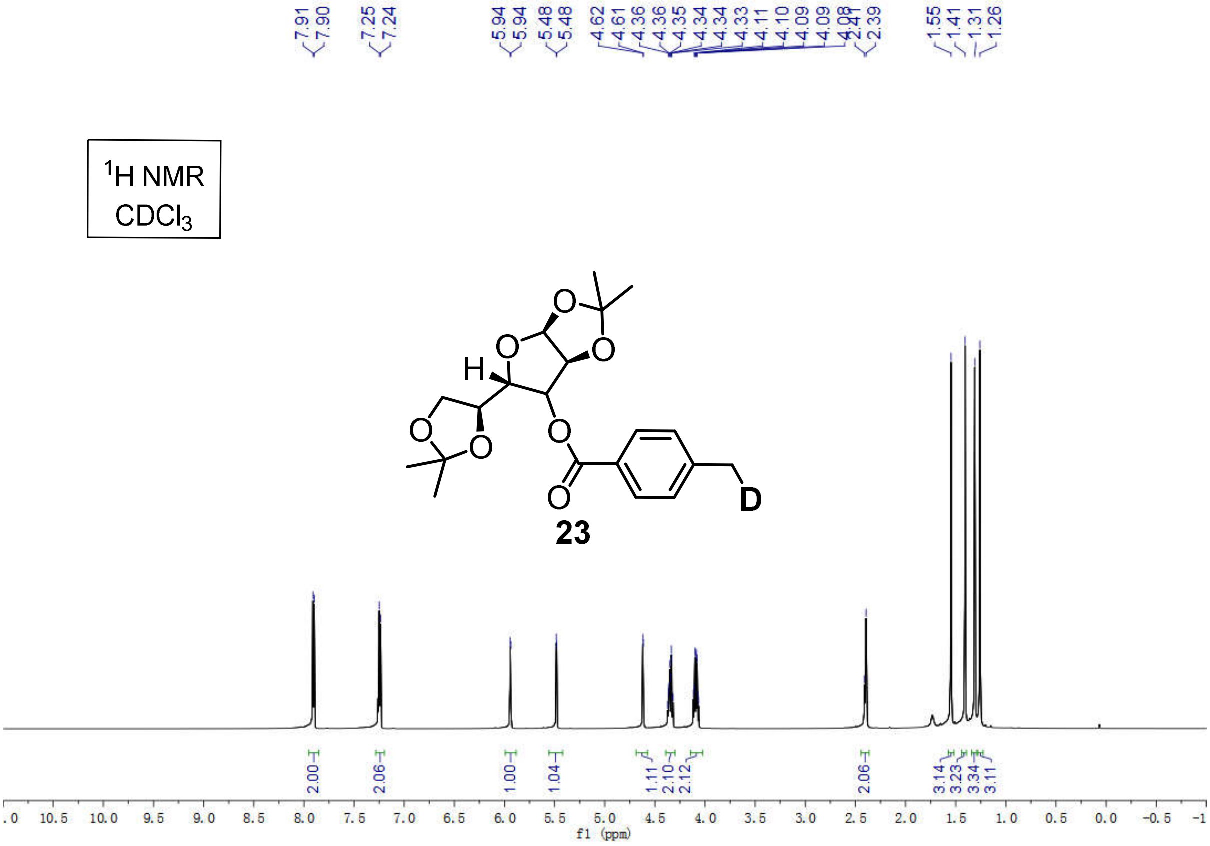

7.91
7.90
7.25
7.24
5.94
5.94
5.48
5.48
4.62
4.61
4.36
4.36
4.35
4.34
4.34
4.33
4.11
4.10
4.09
4.09
4.08
2.41
2.39
1.55
1.41
1.31
1.26
$^1$H NMR
$CDCl_3$
O
H
D
23
2.00
2.06
1.00
1.04
1.11
2.10
2.12
2.06
3.14
3.23
3.34
3.11
f1 (ppm)


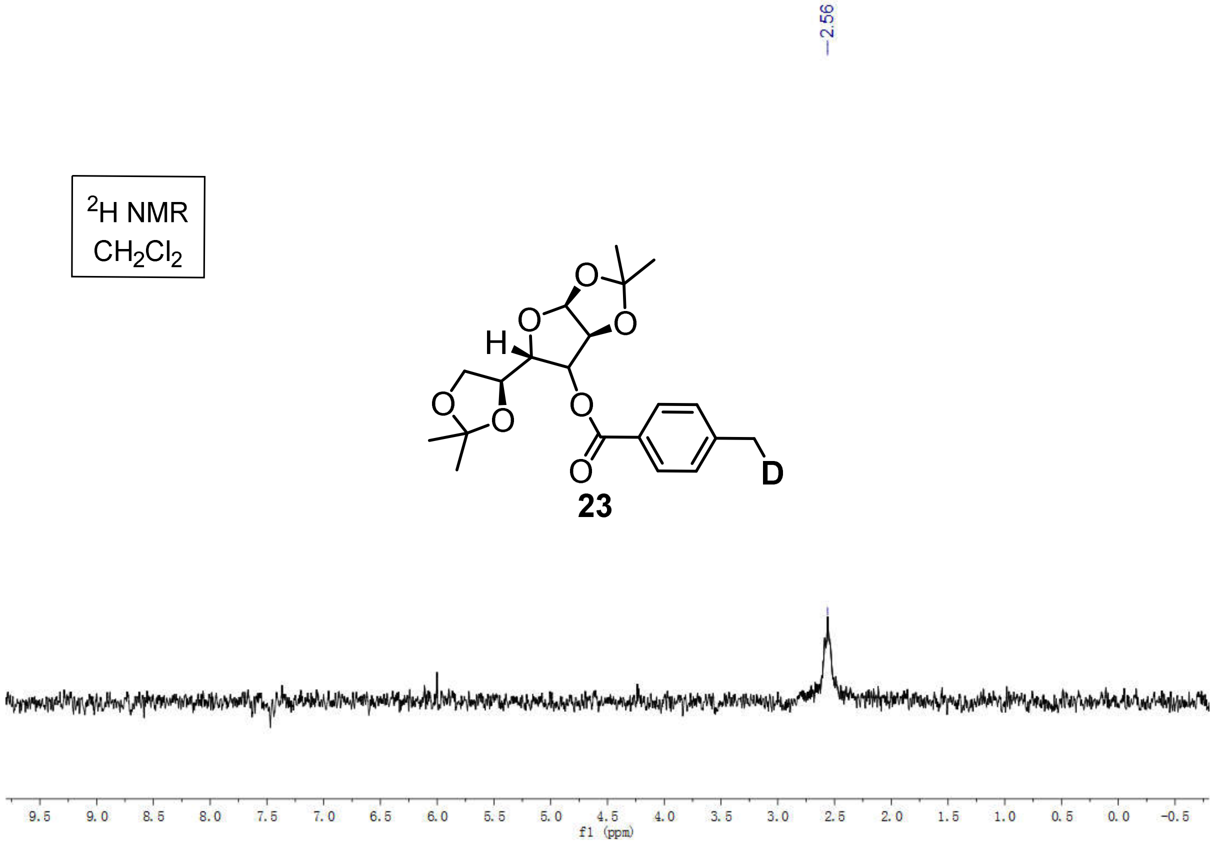

2.56
$^2$H NMR
$CH_2Cl_2$
O
H
D
23
f1 (ppm)

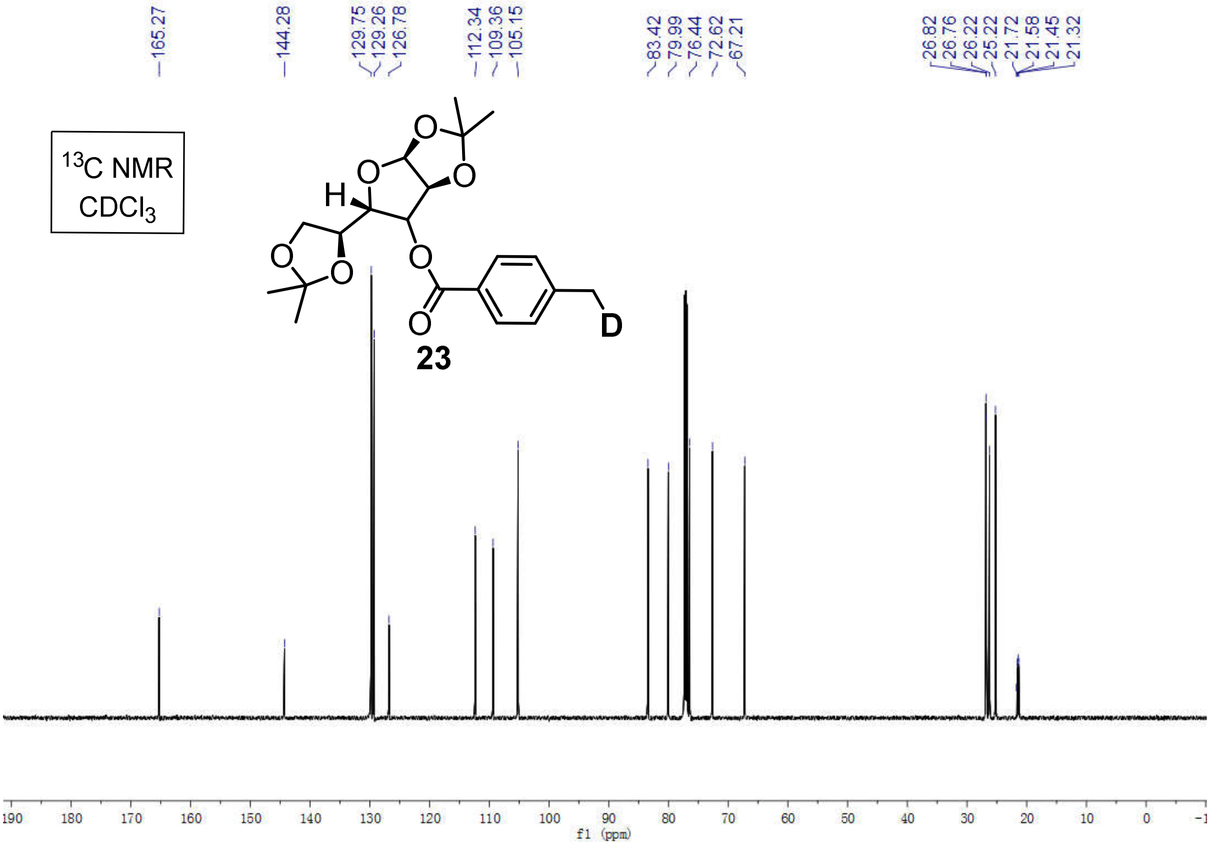


**Supplementary Fig. 126** $^{1}$H NMR, $^{2}$H NMR and $^{13}$C NMR spectra of the compound **23**.

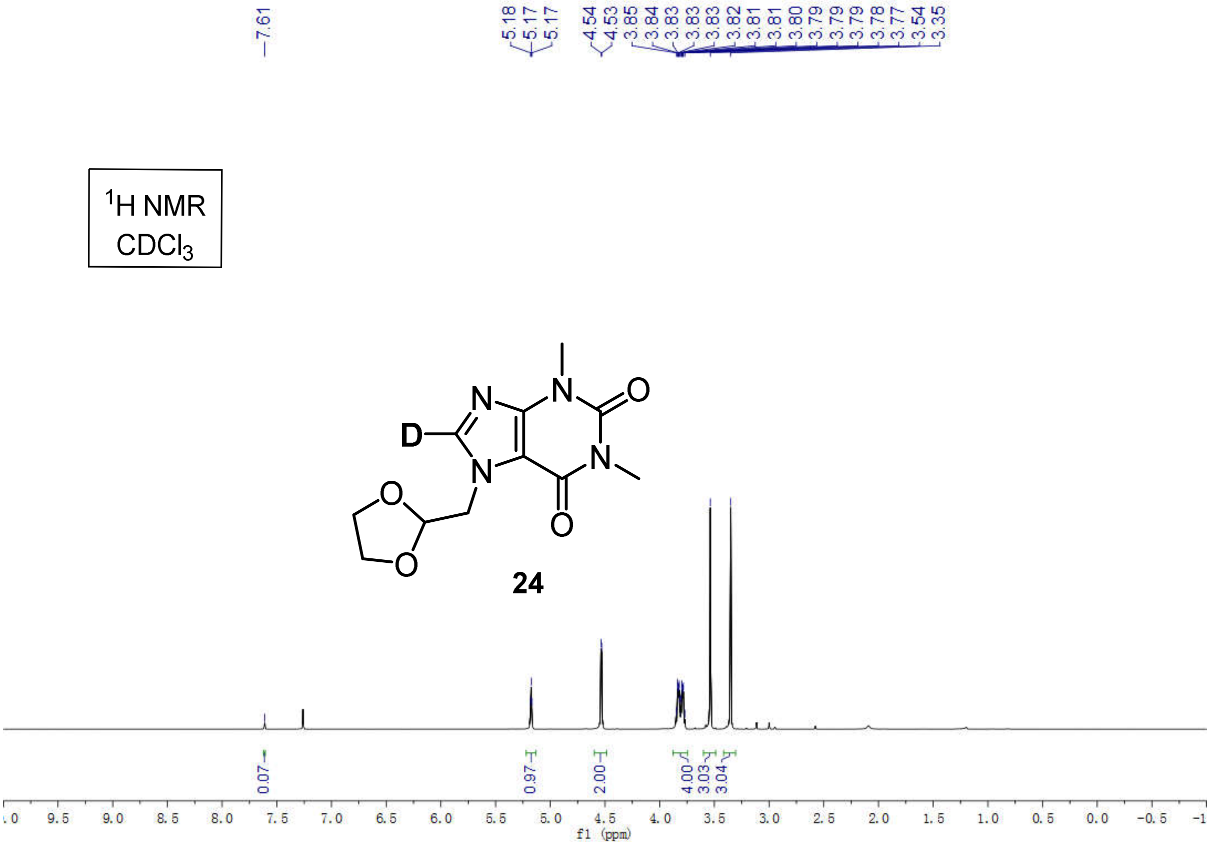
$^1$H NMR
$CDCl_3$
24
7.61
5.18 5.17 5.17
4.54 4.53 3.85 3.84 3.83 3.83 3.83 3.82 3.81 3.81 3.80 3.79 3.79 3.79 3.78 3.77 3.54 3.35
0.07
0.97
2.00
4.00 3.03 3.04
f1 (ppm)

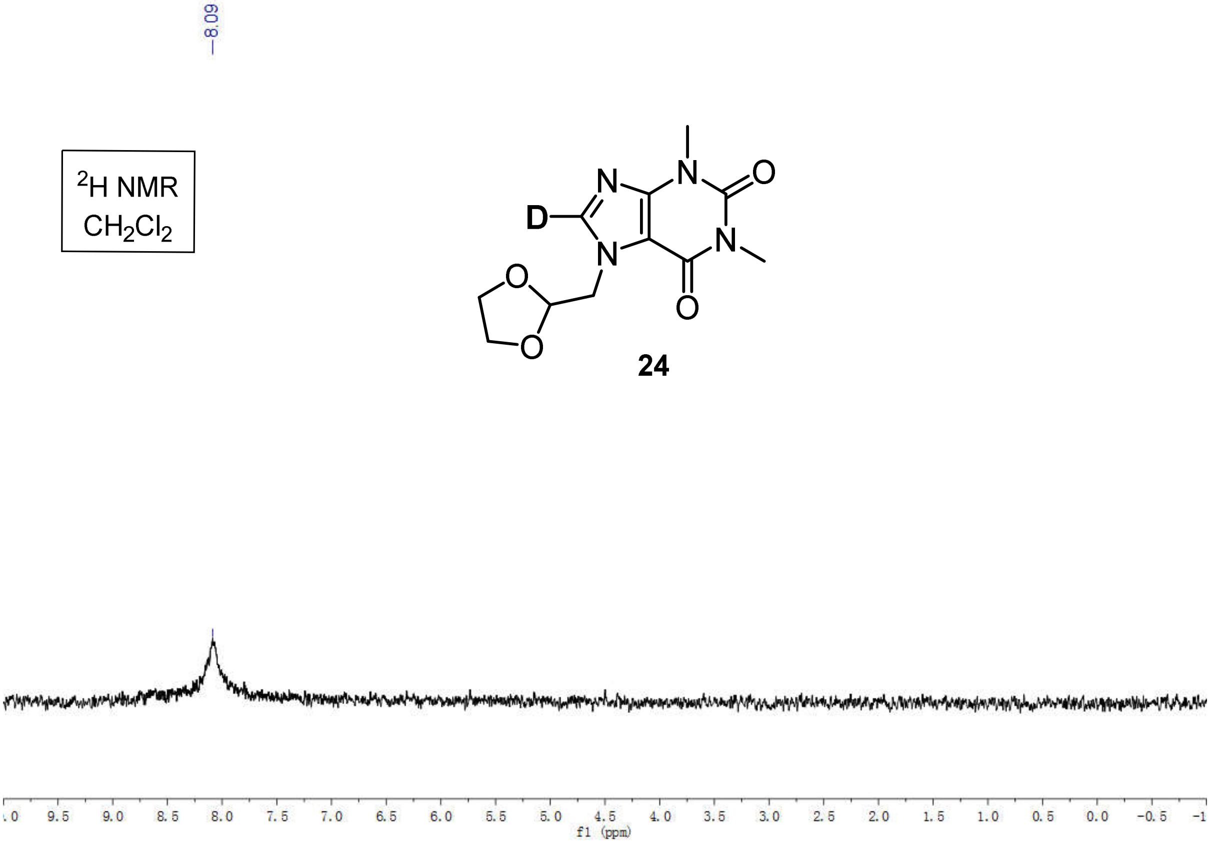
$^2$H NMR
$CH_2Cl_2$
24
8.09
f1 (ppm)

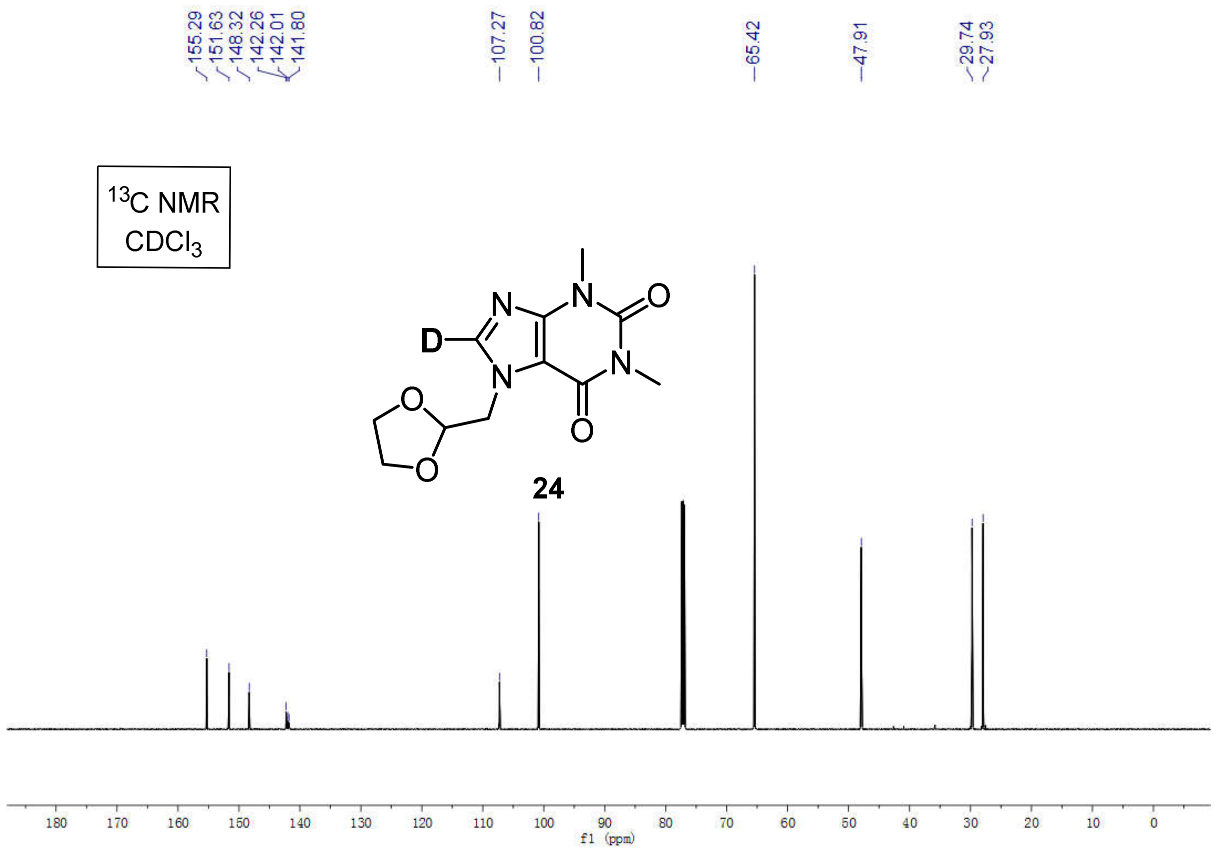


**Supplementary Fig. 127** $^{1}$H NMR, $^{2}$H NMR and $^{13}$C NMR spectra of the compound **24**.

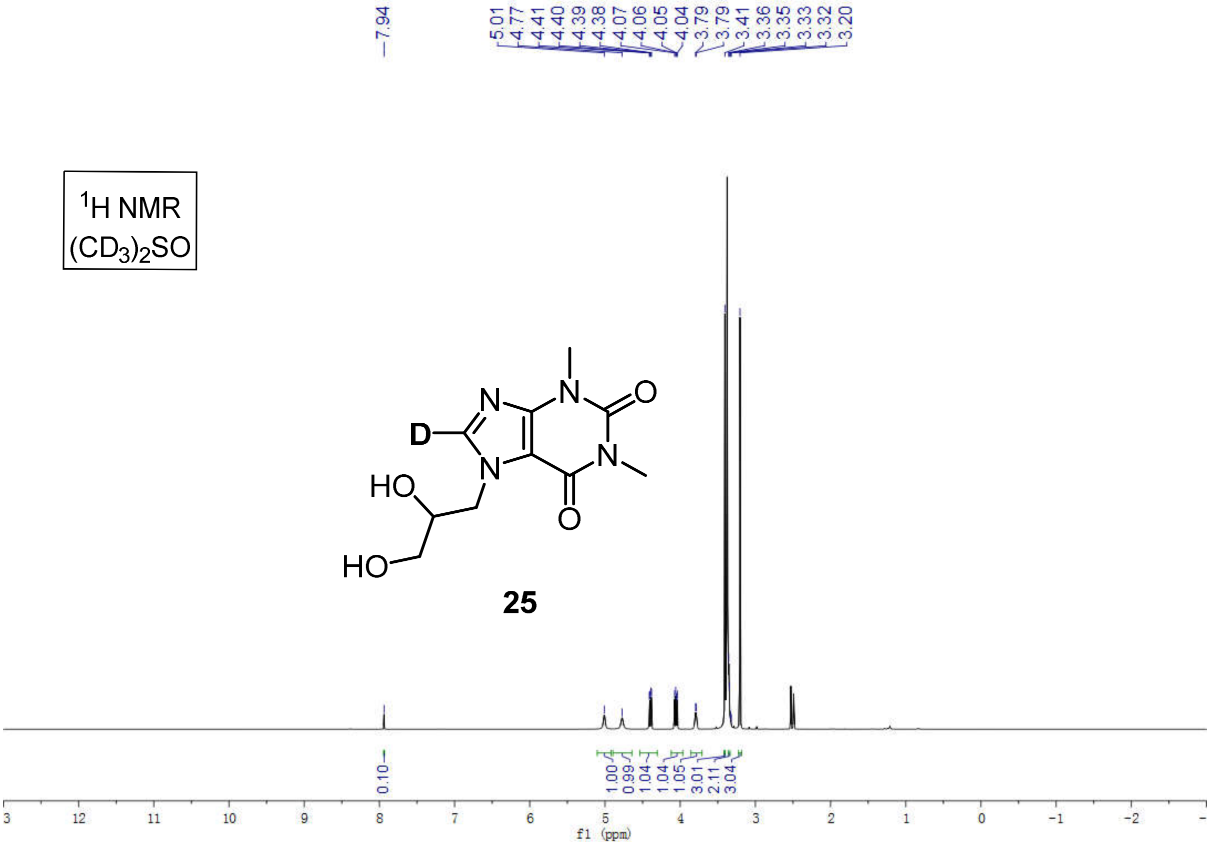

1H NMR
(CD3)2SO
25
f1 (ppm)


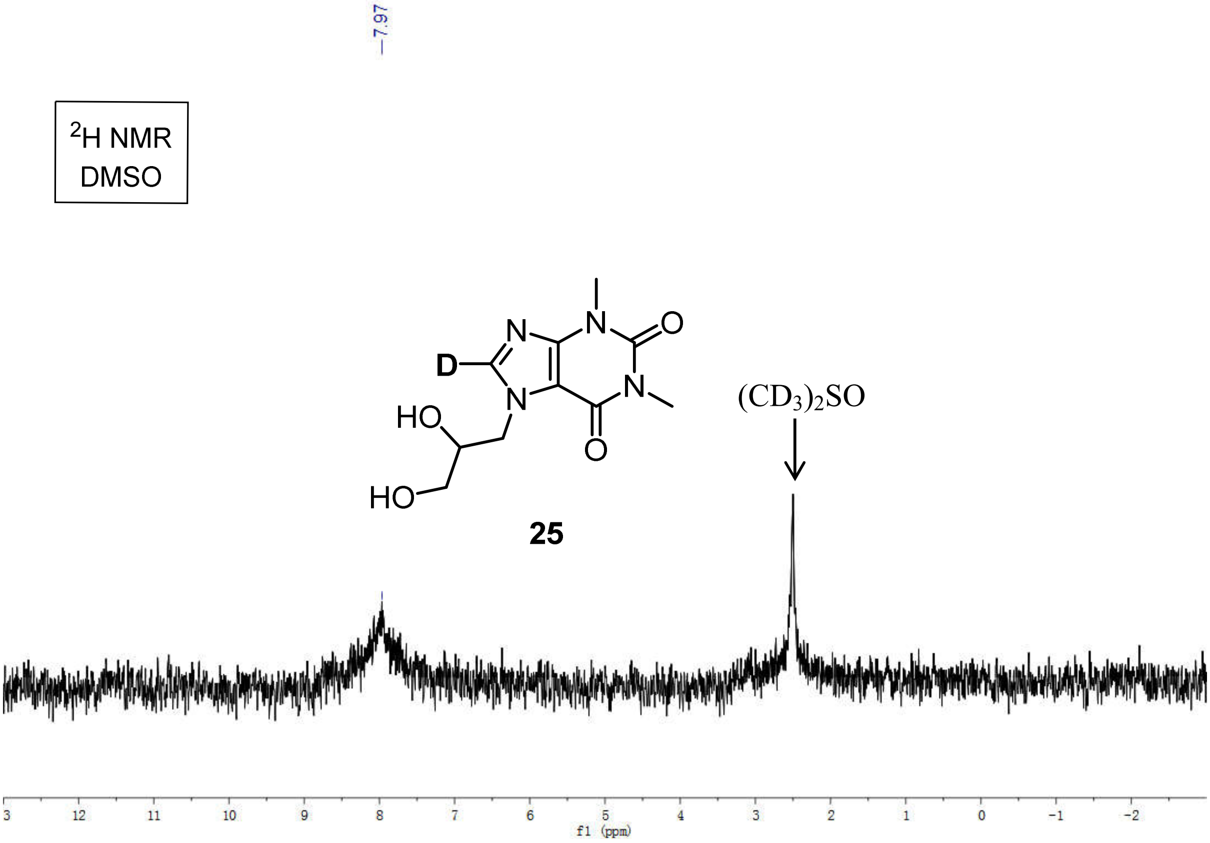

2H NMR
DMSO
25
(CD3)2SO
f1 (ppm)

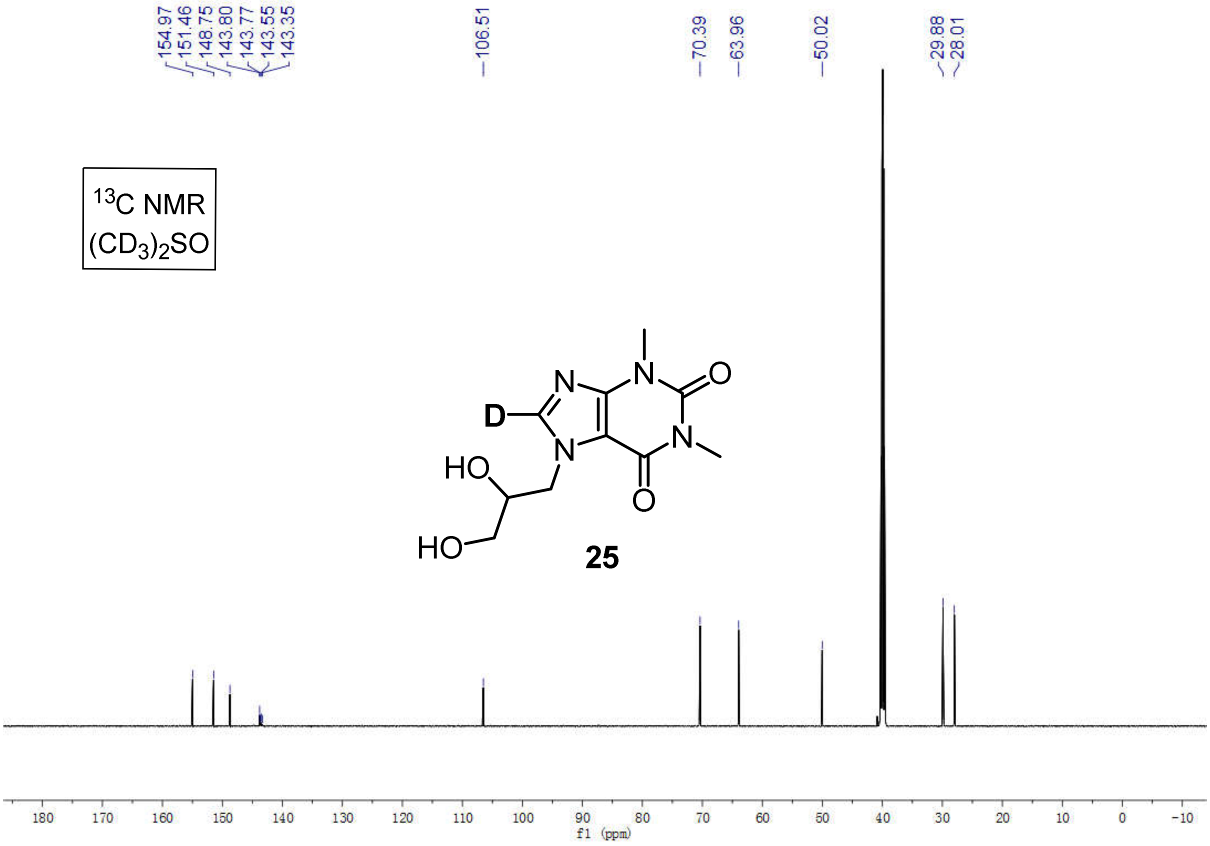


**Supplementary Fig. 128** $^{1}$H NMR, $^{2}$H NMR and $^{13}$C NMR spectra of the compound **25**.

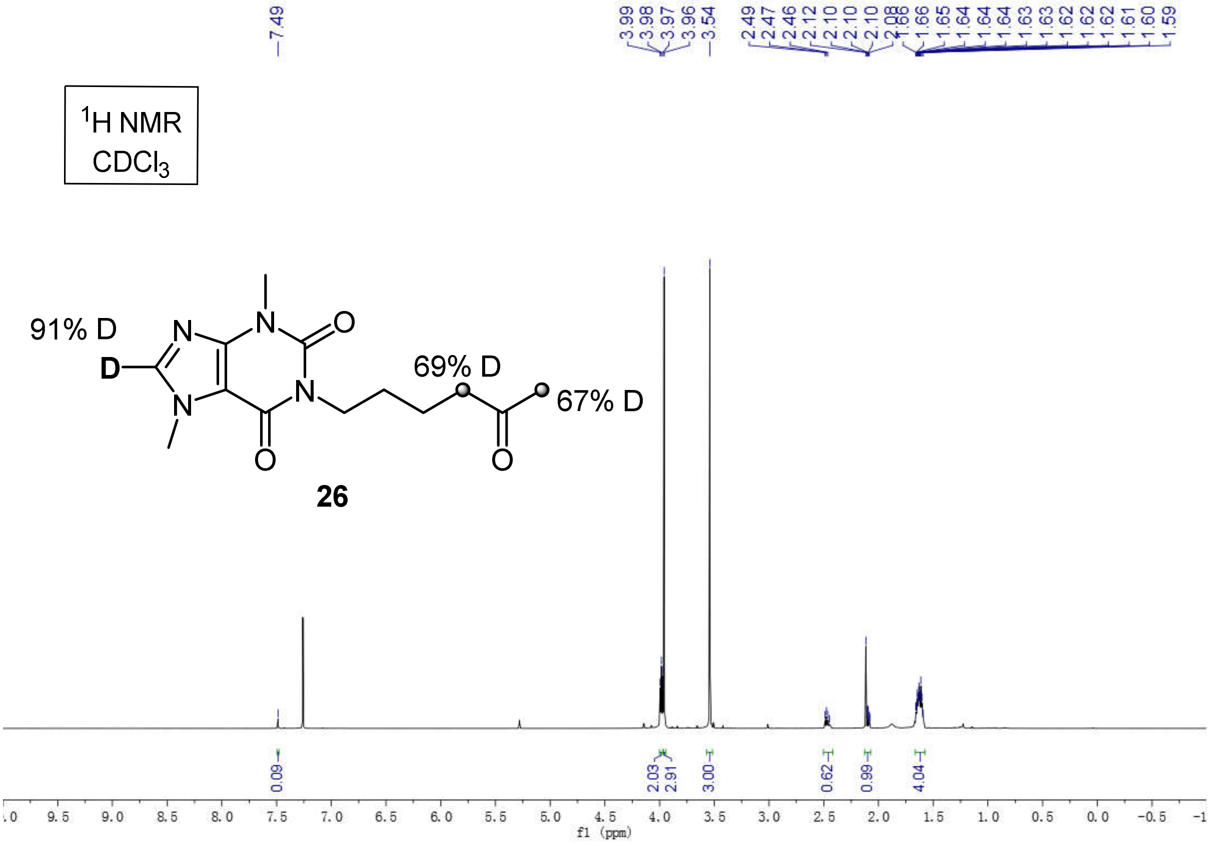

1H NMR
CDCl3
91% D
D
69% D
67% D
26
f1 (ppm)


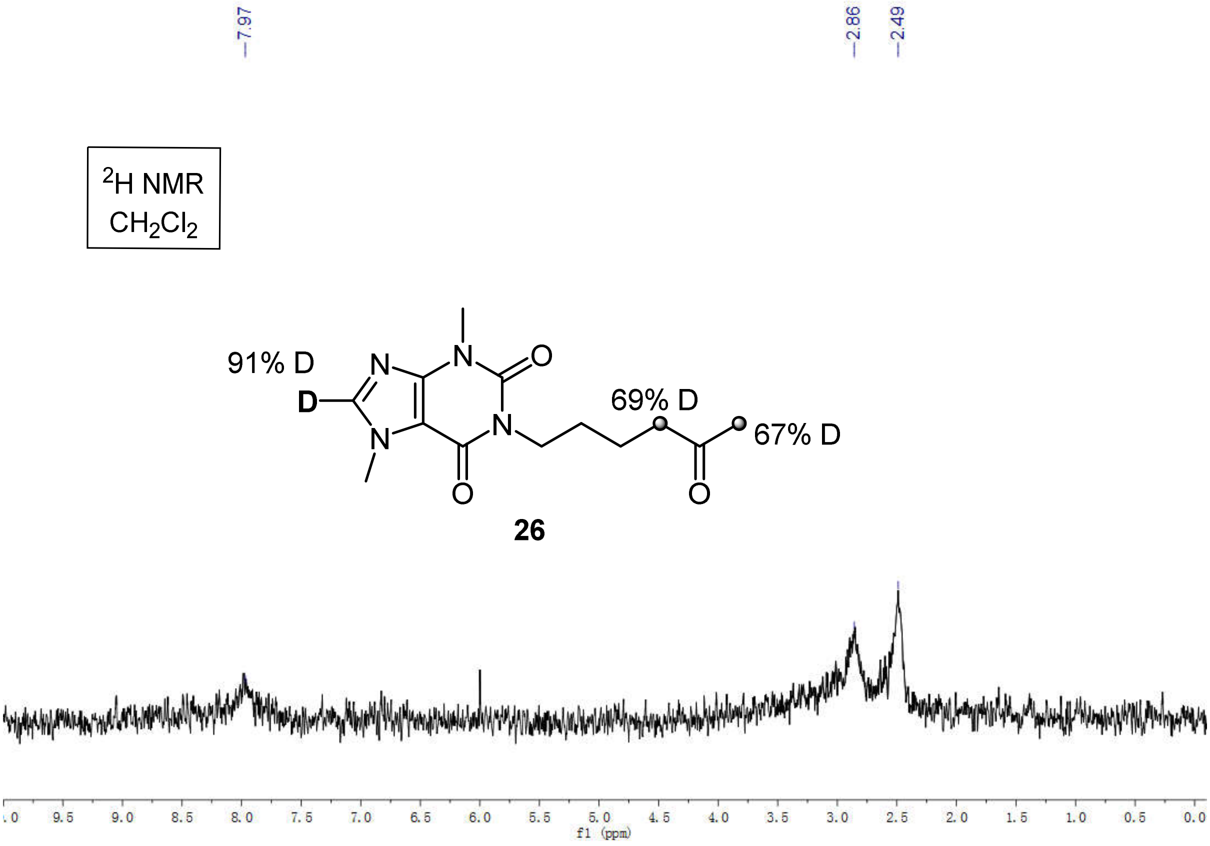

2H NMR
CH2Cl2
91% D
D
69% D
67% D
26
f1 (ppm)

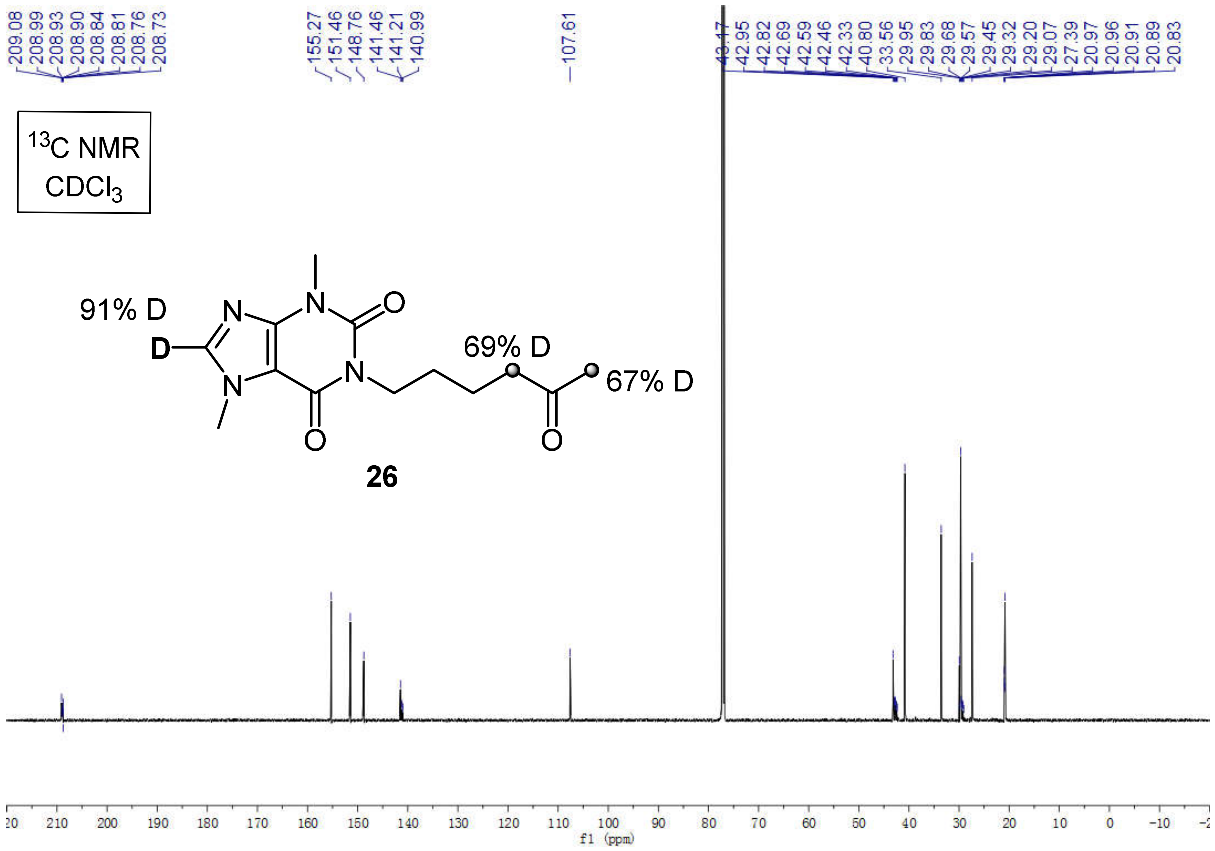


**Supplementary Fig. 129** $^{1}$H NMR, $^{2}$H NMR and $^{13}$C NMR spectra of the compound **26**.

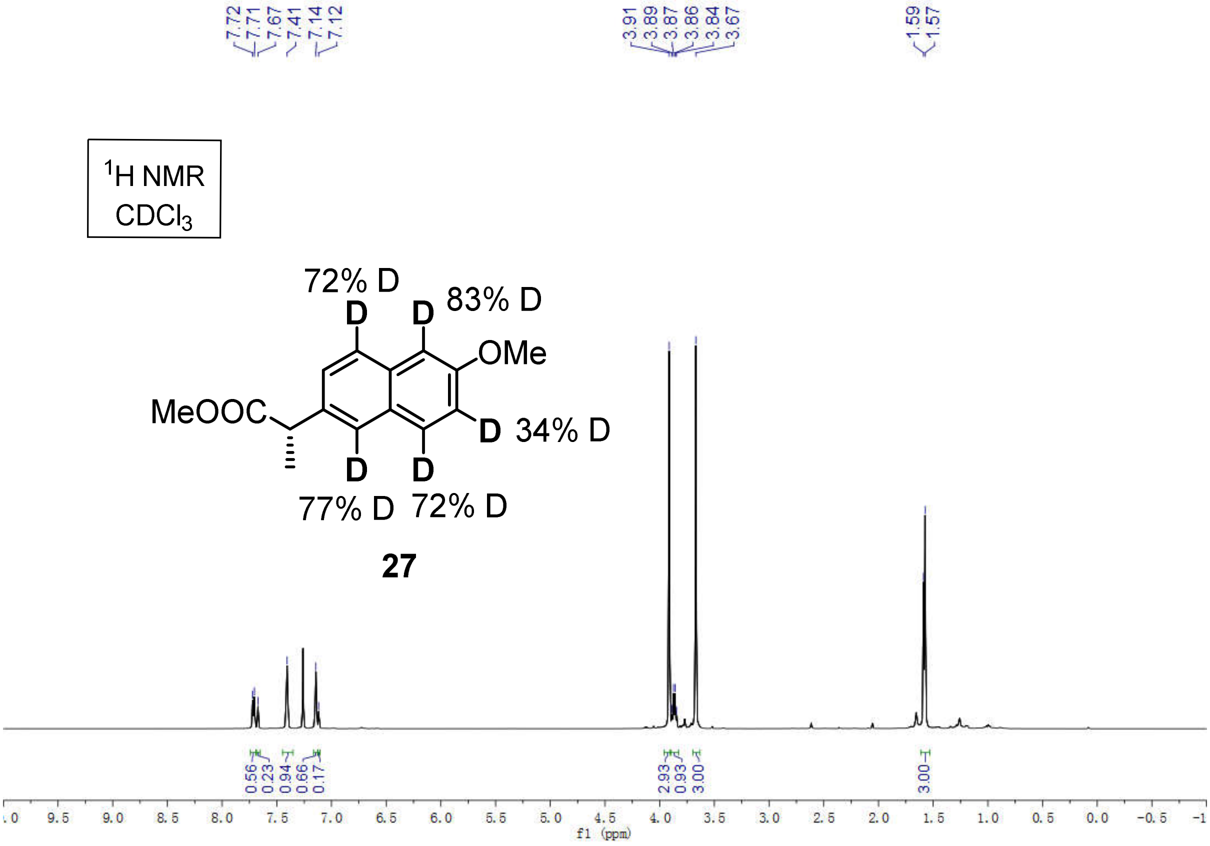
7.72
7.71
7.67
7.41
7.14
7.12
3.91
3.89
3.87
3.86
3.84
3.67
1.59
1.57
1H NMR
CDCl3
72% D
83% D
OMe
MeOOC
D
34% D
77% D
72% D
27
0.56
0.23
0.94
0.66
0.17
2.93
0.93
3.00
3.00
f1 (ppm)

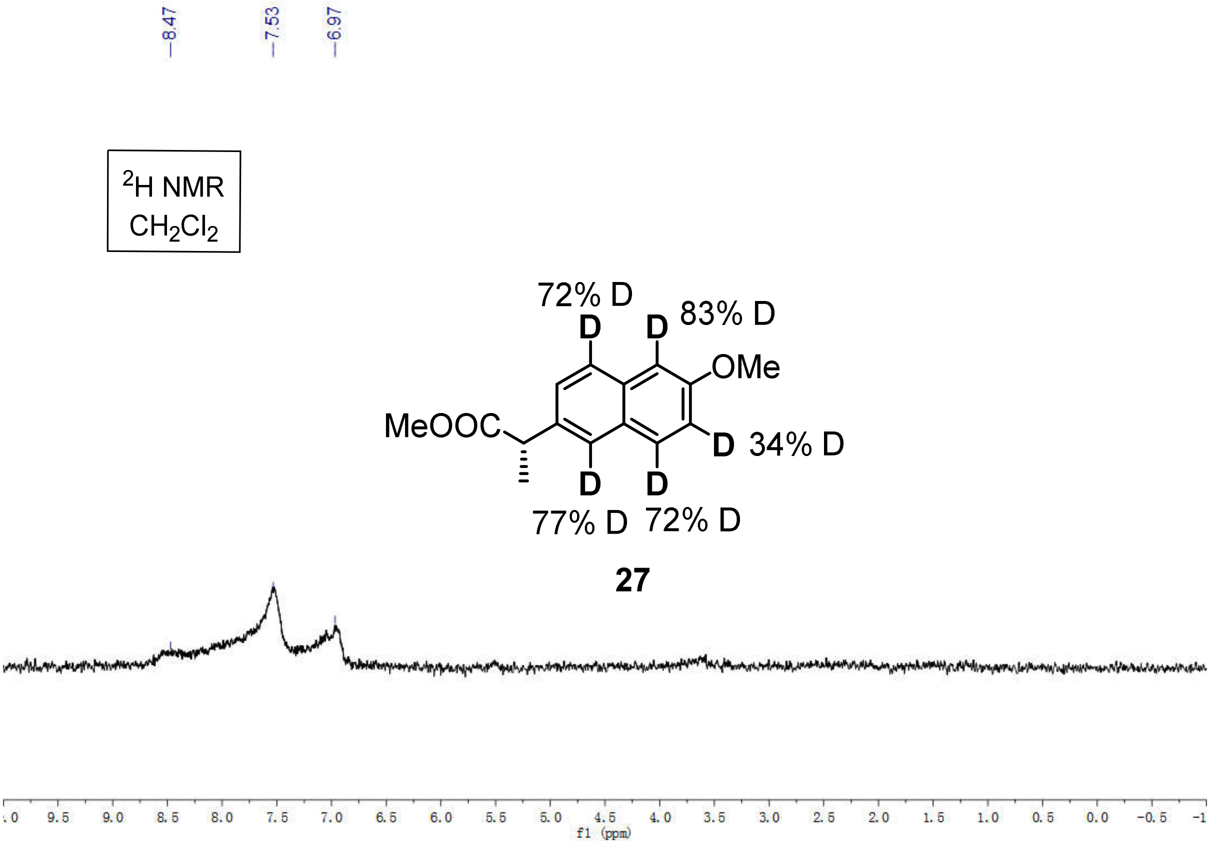
8.47
7.53
6.97
2H NMR
CH2Cl2
72% D
83% D
OMe
MeOOC
D
34% D
77% D
72% D
27
f1 (ppm)

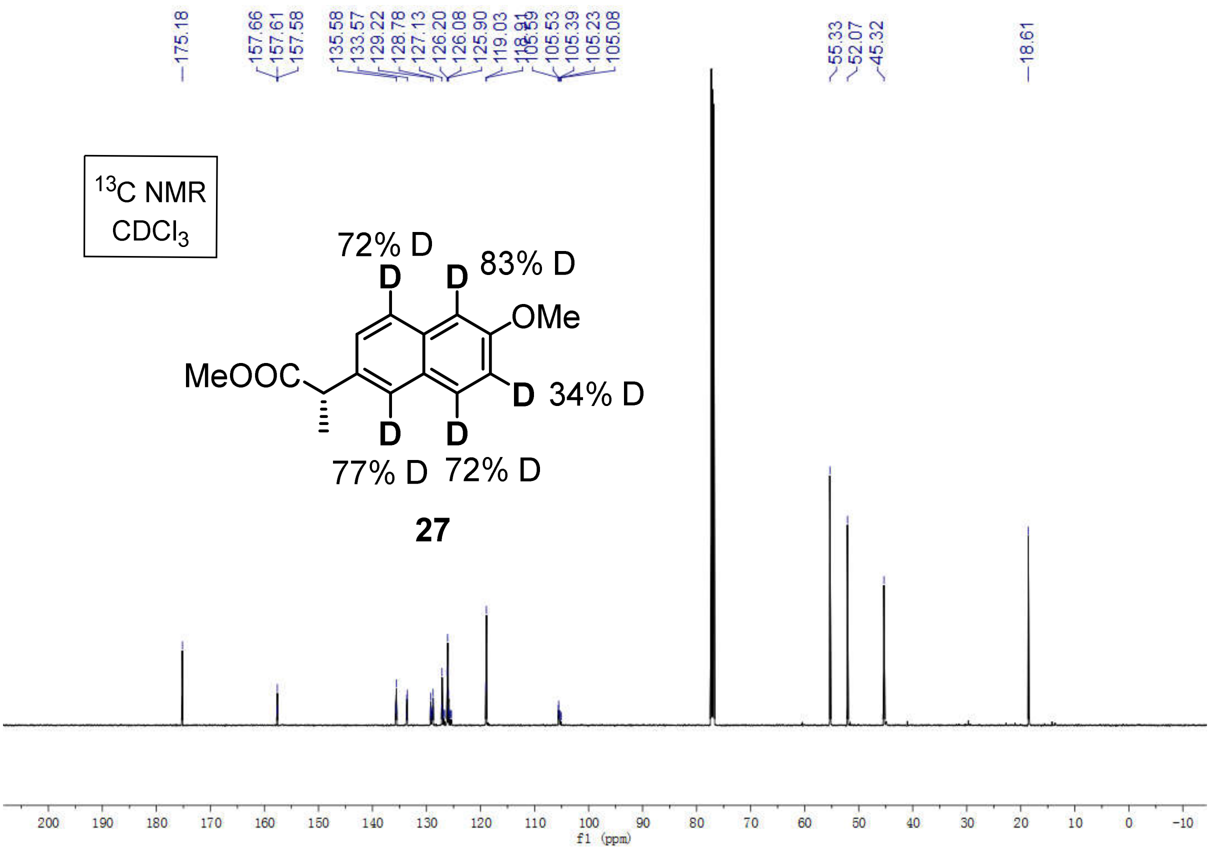


**Supplementary Fig. 130** $^{1}$H NMR, $^{2}$H NMR and $^{13}$C NMR spectra of the compound **27**.

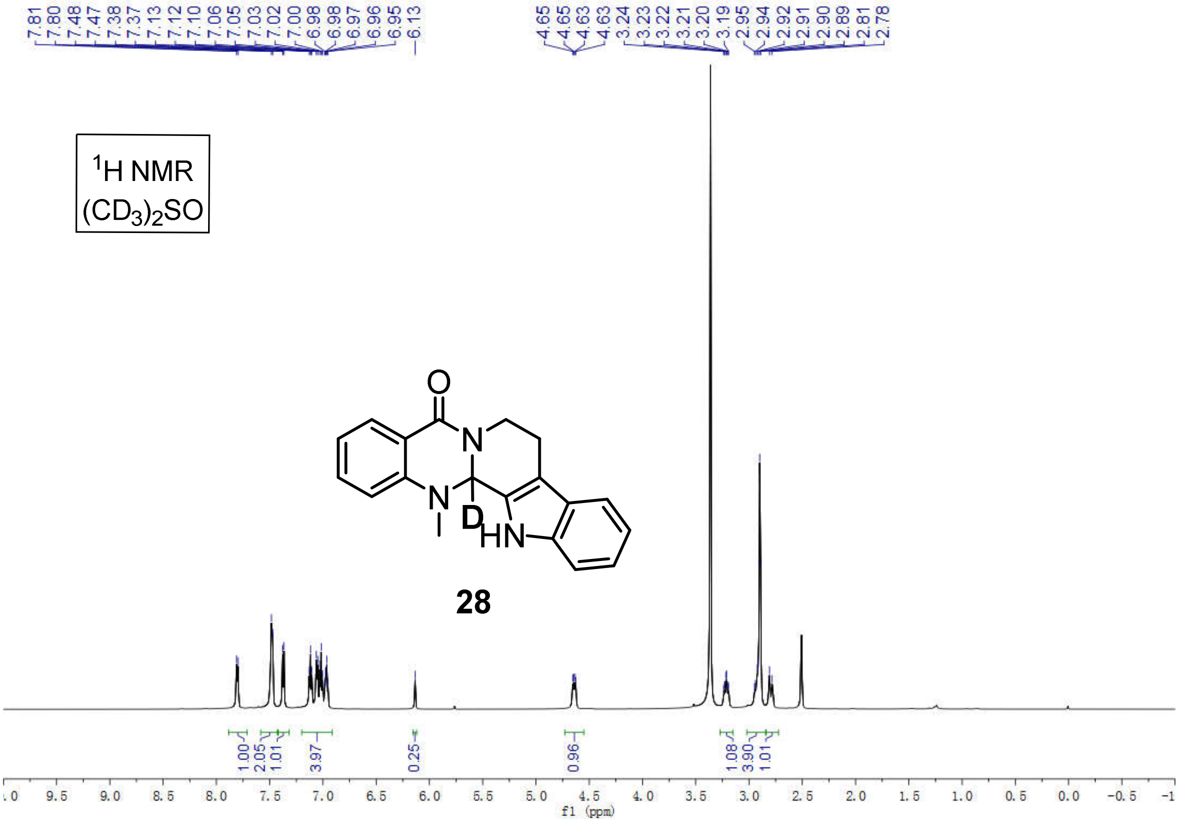


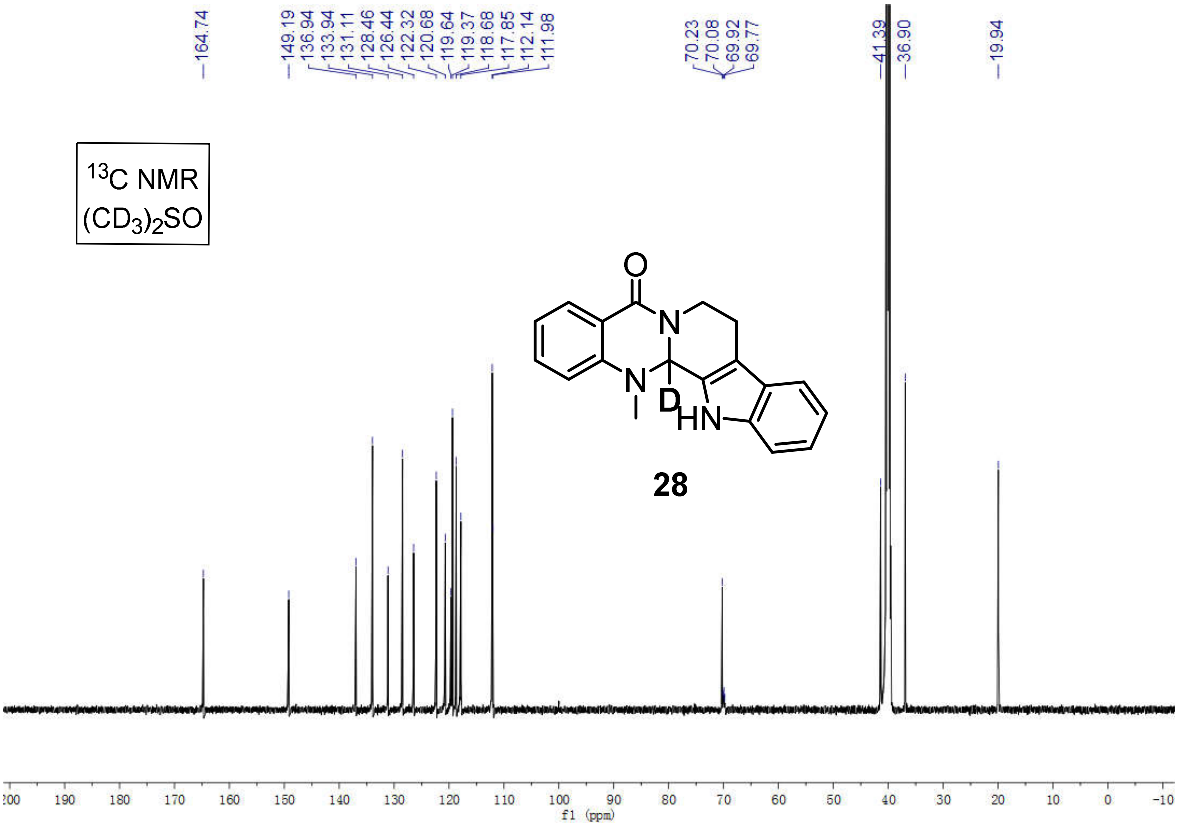


**Supplementary Fig. 131** $^{1}$H NMR, $^{2}$H NMR and $^{13}$C NMR spectra of the compound **28**.

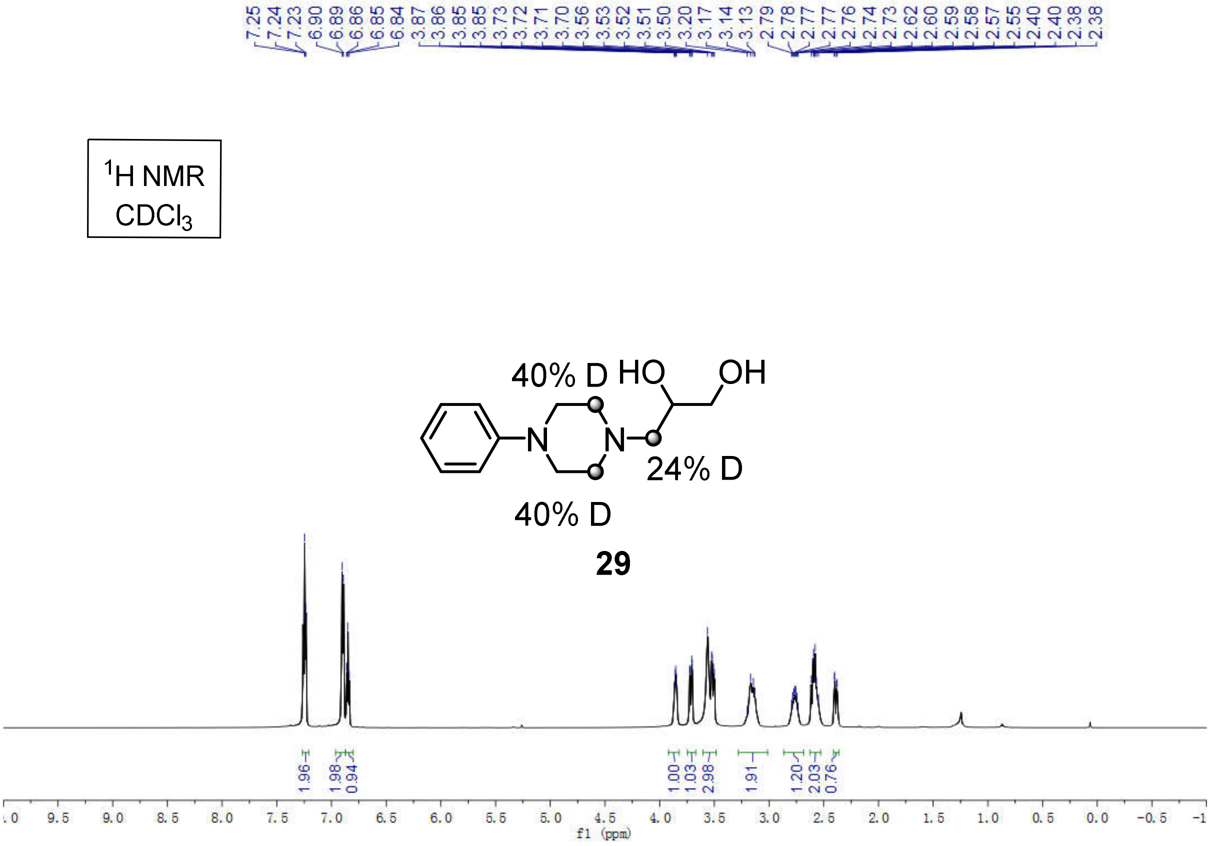

7.25
7.24
7.23
6.90
6.89
6.86
6.85
6.84
3.87
3.86
3.85
3.85
3.73
3.72
3.71
3.70
3.56
3.53
3.52
3.51
3.50
3.20
3.17
3.14
3.13
2.79
2.78
2.77
2.77
2.76
2.74
2.73
2.62
2.60
2.59
2.58
2.57
2.55
2.40
2.40
2.38
2.38
$^1$H NMR
$CDCl_3$
40% D
HO
OH
N
N
24% D
40% D
29
1.96
1.98
0.94
1.00
1.03
2.98
1.91
1.20
2.03
0.76
f1 (ppm)


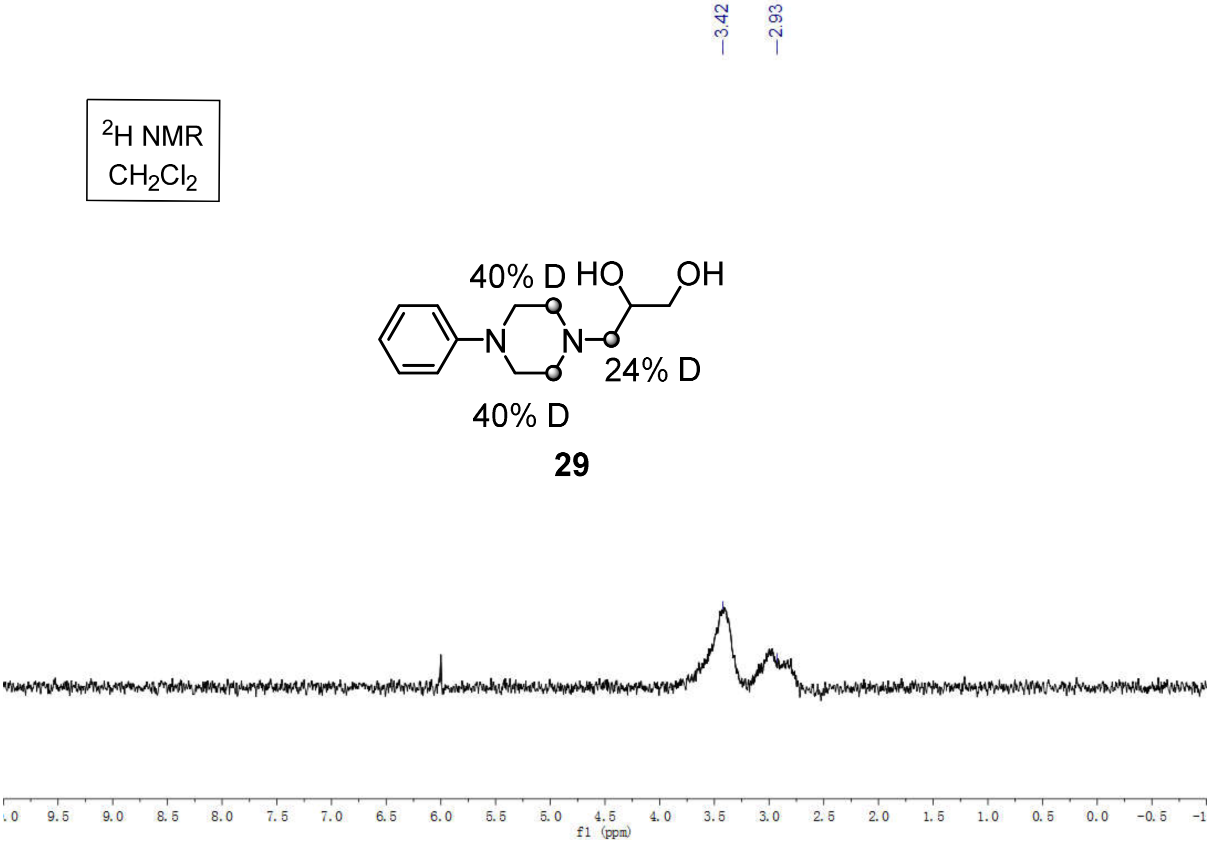

3.42
2.93
$^2$H NMR
$CH_2Cl_2$
40% D
HO
OH
N
N
24% D
40% D
29
f1 (ppm)

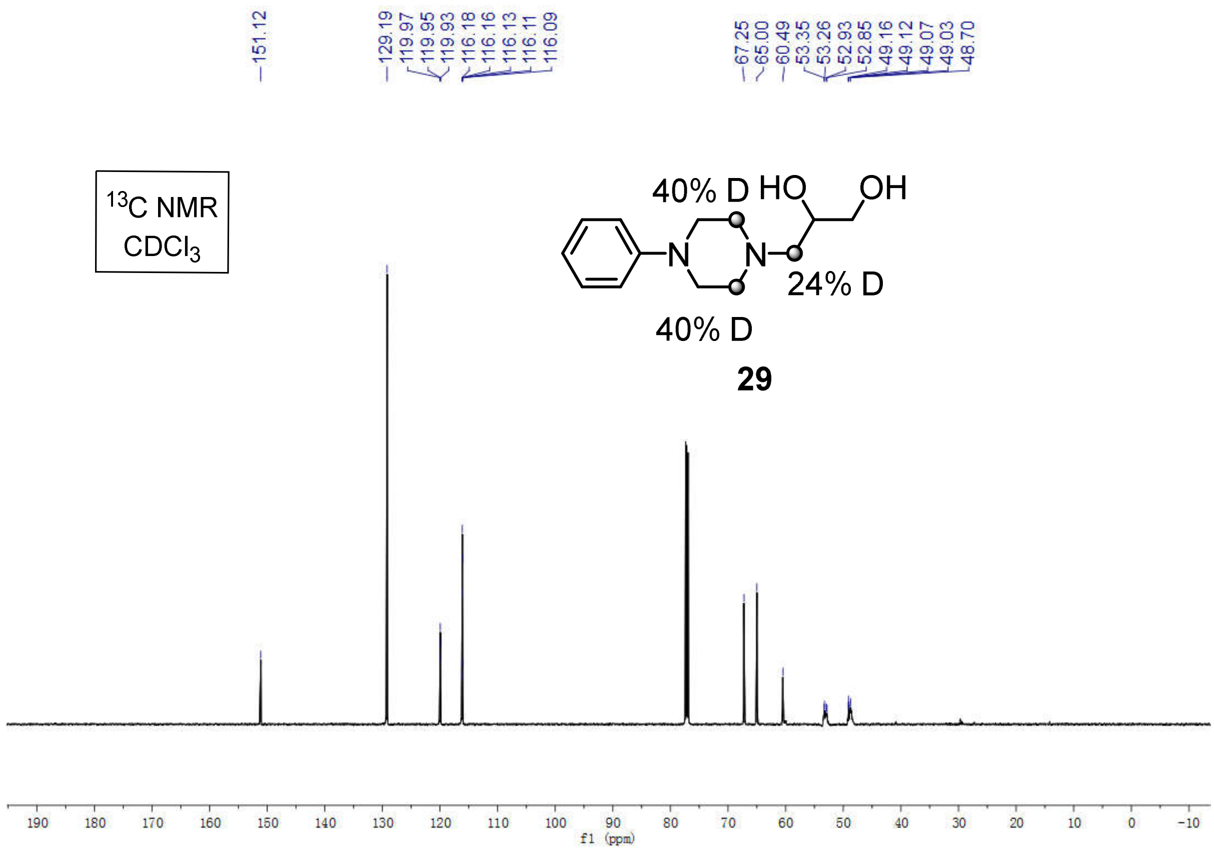


**Supplementary Fig. 132** $^{1}$H NMR, $^{2}$H NMR and $^{13}$C NMR spectra of the compound **29**.

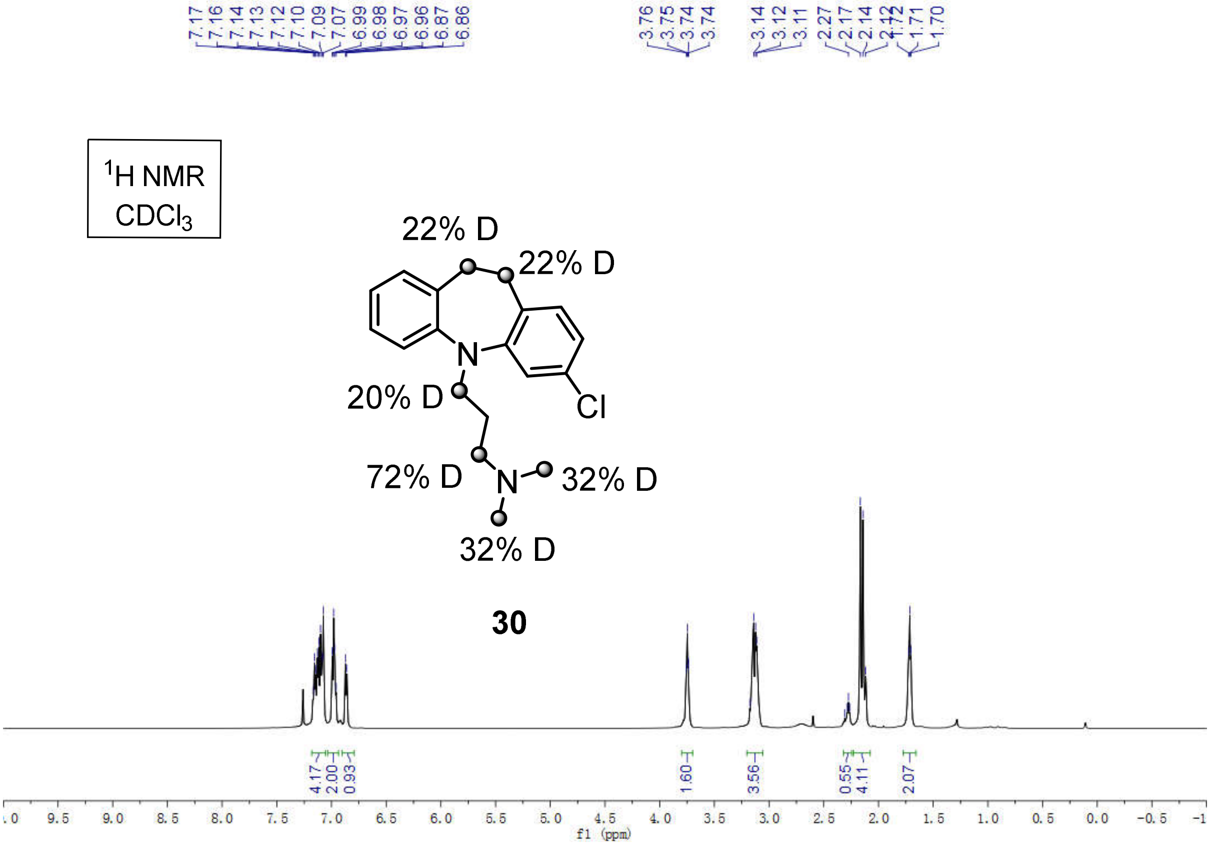
7.17 7.16 7.14 7.13 7.12 7.10 7.09 7.07 6.99 6.98 6.97 6.96 6.87 6.86
3.76 3.75 3.74 3.74
3.14 3.12 3.11 2.27 2.17 2.14 2.12 1.72 1.71 1.70
1H NMR
CDCl3
22% D
22% D
20% D
Cl
72% D
32% D
32% D
30
4.17 2.00 0.93
1.60
3.56
0.55 4.11
2.07
f1 (ppm)

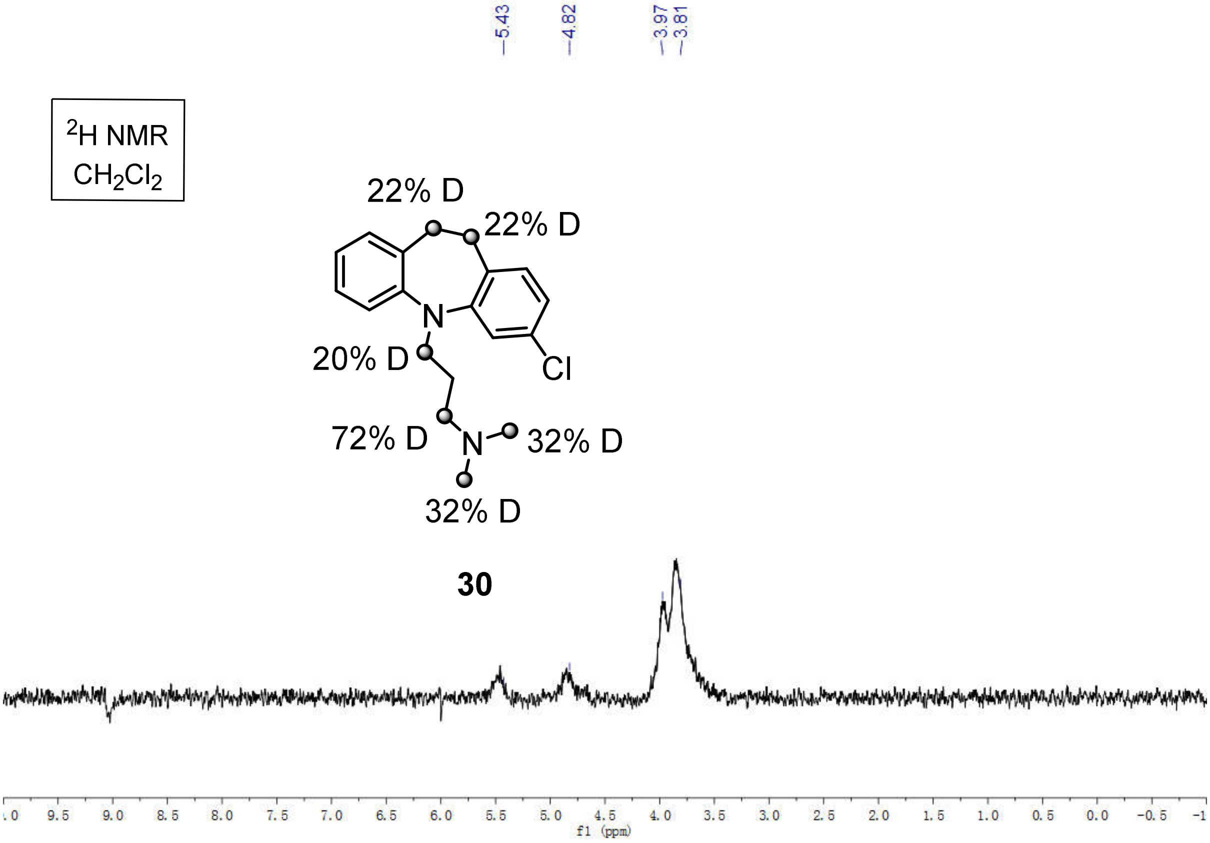
5.43
4.82
3.97 3.81
2H NMR
CH2Cl2
22% D
22% D
20% D
Cl
72% D
32% D
32% D
30
f1 (ppm)

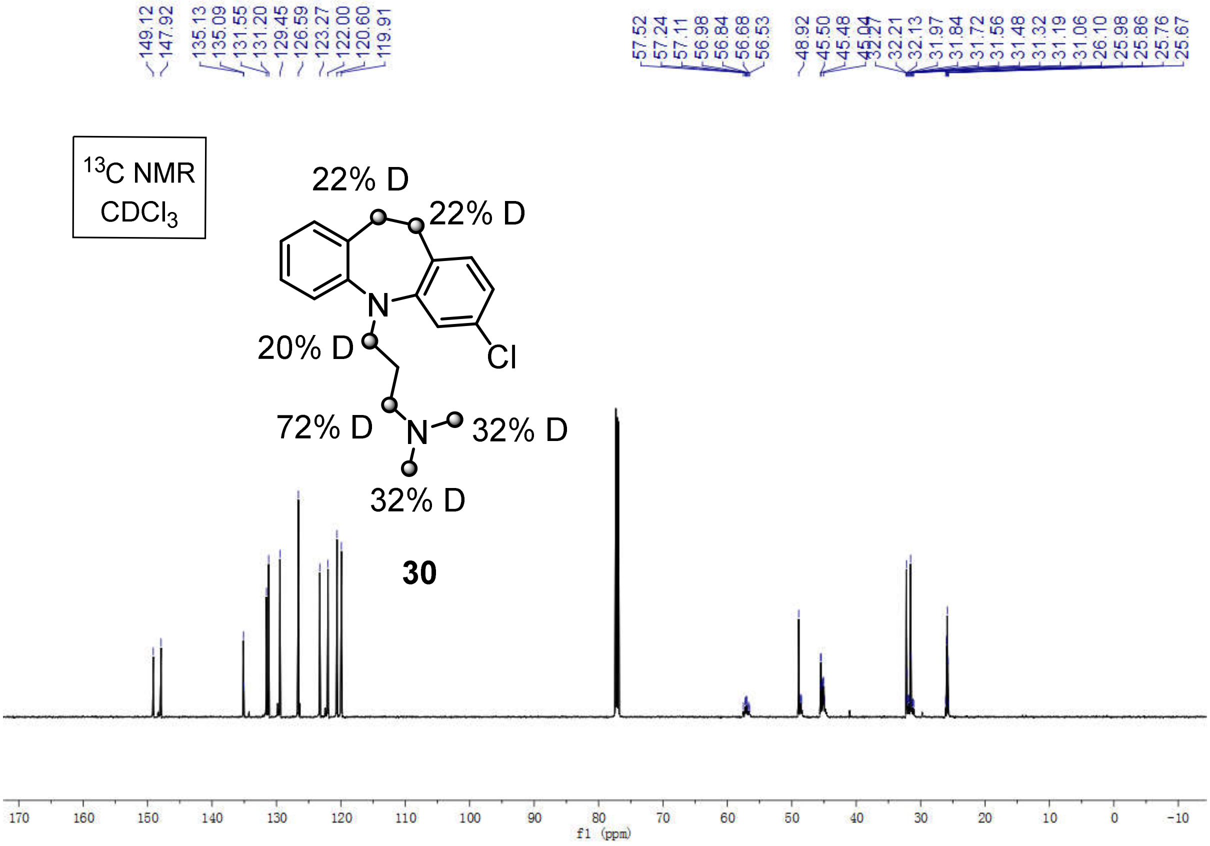


**Supplementary Fig. 133** $^{1}$H NMR, $^{2}$H NMR and $^{13}$C NMR spectra of the compound **30**.

## Supplementary References


1. Vishnoi, P., Sen, S., Patwari, G. N. & Murugavel, R. Charge transfer aided selective sensing and capture of picric acid by triphenylbenzenes. *New J. Chem.* **39**, 886–892 (2015).
2. Sasaki, S., Suzuki, S., Sameera, W. M. C., Igawa, K., Morokuma, K. & Konishi, G. Highly twisted *N*,*N*-dialkylamines as a design strategy to tune simple aromatic hydrocarbons as steric environment-sensitive fluorophores. *J. Am. Chem. Soc.* **138**, 8194−8206 (2016).
3. Matsubara, R., Yabuta, T., Md Idros, U., Hayashi, M., Ema, F., Kobori, Y. & Sakata, K. UVA- and visible-light-mediated generation of carbon radicals from organochlorides using nonmetal photocatalyst. *J. Org. Chem.* **83**, 9381−9390 (2019).
4. Lee, K. C., Lee, S.-Y., Choe, Y. S & Chi, D. Y. Metabolic stability of [$^{18}$F]fluoroalkylbiphenyls. *Bull. Korean Chem. Soc.* **25**, 1225–1230 (2004).
5. Kigawa, H., Takamuku, S., Toki, S., Kimura, K., Takeda, S., Tsumori, K. & Sakurai, H. Intramolecular electron transfer and $S_N2$ reactions in the radical anions of 1-(4-biphenylyl)-ω-haloalkane studied by pulse radiolysis. *J. Am. Chem. Soc.* **103**, 5176−5179 (1981).
6. Villalpando, A., Ayala, C. E., Watson, C. B. & Kartika, R. Triphosgene-amine base promoted chlorination of unactivated aliphatic alcohols. *J. Org. Chem.* **78**, 3989−3996 (2013).
7. Neuvonen, H., Neuvonen, K. & Pasanen, P. Evidence of substituent-induced electronic interplay. Effect of the remote aromatic ring substituent of phenyl benzoates on the sensitivity of the carbonyl unit to electronic effects of phenyl or benzoyl ring substituents. *J. Org. Chem.* **69**, 3794−3800 (2004).
8. Iwasaki, T., Fukuoka, A., Yokoyama, W., Min, X., Hisaki, I., Yang, T., Ehara, M., Kuniyasu, H. & Kambe, N. Nickel-catalyzed coupling reaction of alkyl halides with aryl grignard reagents in the presence of 1,3-butadiene: mechanistic studies of four-component coupling and competing cross-coupling reactions. *Chem. Sci.* **9**, 2195−2211 (2018).
9. Li, T. W., Yang, Y. C., Cheng, C. M., Wang, D. C., Lu, A. J. & Zhao, Y. F. Design, synthesis and biological evaluation of multi-substituted *N*-phenyl-2,2-dichloro acetamide analogues as anti-cancer drugs. *Acta Pharm. Sin.* **47**, 354−363 (2012).

10. Hoffmann-Emery, F., Hilpert, H., Scalone, M. & Waldmeier, P. Efficient synthesis of novel NK1 receptor antagonists: selective 1,4-addition of Grignard reagents to 6-Chloronicotinic acid derivatives. *J. Org. Chem.* **71**, 2000−2008 (2006).
11. Dyck, J., Zavorine, S., Robertson, A. J., Capretta, A., Larichev, V., Britten, J. & McNulty, J. Dimethylmalonyltrialkylphosphoranes: probing the steric effect on phosphorus and its stereochemical consequence in esterification reactions of chiral secondary alcohols. *J. Organomet. Chem.* **690**, 2548−2552 (2005).
12. Margrey, K. A., Levens, A. & Nicewicz, D. A. Direct aryl C-H amination with primary amines using organic photoredox catalysis. *Angew. Chem. Int. Ed.* **56**, 15644−15648 (2017).
13. Frisch, M. J., Trucks, G. W., Schlegel, H. B., Scuseria, G. E., Robb, M. A., Cheeseman, J. R., Scalmani, G., Barone, V., Mennucci, B., Petersson, G. A., Nakatsuji, H., Caricato, M., Li, X., Hratchian, H. P., Izmaylov, A. F., Bloino, J., Zheng, G., Sonnenberg, J. L., Hada, M., Ehara, M., Toyota, K., Fukuda, R., Hasegawa, J., Ishida, M., Nakajima, T., Honda, Y., Kitao, O., Nakai, H., Vreven, T., Montgomery, J. A., Jr., Peralta, J. E., Ogliaro, F., Bearpark, M., Heyd, J. J., Brothers, E., Kudin, K. N., Staroverov, V. N., Keith, T., Kobayashi, R., Normand, J., Raghavachari, K., Rendell, A., Burant, J. C., Iyengar, S. S., Tomasi, J., Cossi, M., Rega, N., Millam, J. M., Klene, M., Knox, J. E., Cross, J. B., Bakken, V., Adamo, C., Jaramillo, J., Gomperts, R., Stratmann, R. E., Yazyev, O., Austin, A. J., Cammi, R., Pomelli, C., Ochterski, J. W., Martin, R. L., Morokuma, K., Zakrzewski, V. G., Voth, G. A., Salvador, P., Dannenberg, J. J., Dapprich, S., Daniels, A. D., Farkas, O., Foresman, J. B., Ortiz, J. V., Cioslowski, J. & Fox, D. J. Gaussian 09, Revision E.01 (Gaussian, Inc., Wallingford CT) (2013).
14. Y, Zhao., D. G. Truhlar. *Theor. Chem. Acc.* **120**, 215 (2008).
15. Ditchfield, R., Hehre, W. J. & Pople, J. A. Self-consistent molecular orbital methods. IX. An extended Gaussian-type basis for molecular-orbital studies of organic molecules. *J. Chem. Phys.* **54**, 724−728 (1971).
16. Hehre, W. J., Ditchfield, R. & Pople, J. A. Self-consistent molecular orbital methods. XII. further extensions of Gaussian-type basis sets for use in molecular orbital studies of organic molecules. *J. Chem. Phys.* **56**, 2257−2261 (1972).
17. Hariharan, P. C. & Pople, J. A. The influence of polarization functions on molecular orbital hydrogenation energies. *Theor. Chim. Acta.* **28**, 213−222 (1973).

18. Tomasi, J. & Persico, M. Molecular interactions in solution: an overview of methods based on continuous distributions of the solvent. *Chem. Rev.* **94**, 2027−2094 (1994).
19. Marenich, A. V., Cramer, C. J. & Truhlar, D. G. Universal solvation model based on solute electron density and on a continuum model of the solvent defined by the bulk dielectric constant and atomic surface tensions. *J. Phys. Chem. B.* **113**, 6378−6396 (2009).
